# Persistent inequalities in consultation incidence and prevalence of low back pain and osteoarthritis in England between 2004 and 2019

**DOI:** 10.1093/rap/rkac106

**Published:** 2022-12-02

**Authors:** Dahai Yu, Kelvin P Jordan, Ross Wilkie, James Bailey, Justine Fitzpatrick, Nuzhat Ali, Paul Niblett, George Peat

**Affiliations:** Primary Care Centre Versus Arthritis, School of Medicine, Keele University, Keele, UK; Primary Care Centre Versus Arthritis, School of Medicine, Keele University, Keele, UK; Primary Care Centre Versus Arthritis, School of Medicine, Keele University, Keele, UK; Primary Care Centre Versus Arthritis, School of Medicine, Keele University, Keele, UK; Department of Health and Social Care, Office for Health Improvement and Disparities, London, UK; Department of Health and Social Care, Office for Health Improvement and Disparities, London, UK; Department of Health and Social Care, Office for Health Improvement and Disparities, London, UK; Primary Care Centre Versus Arthritis, School of Medicine, Keele University, Keele, UK; Department of Allied Health Professions, College of Health, Wellbeing & Life Sciences, Sheffield Hallam University, Sheffield, UK

**Keywords:** low back pain, osteoarthritis, incidence, prevalence, deprivation, Clinical Practice Research Datalink, socioeconomic inequality, slope index of inequality, relative index of inequality

## Abstract

**Objective:**

We wanted to determine whether socioeconomic inequalities in primary care consultation rates for two major, disabling musculoskeletal conditions in England narrowed or widened between 2004 and 2019.

**Methods:**

We analysed data from Clinical Practice Research Datalink Aurum, a national general practice electronic health records database, linked to national deprivation ranking of each patient’s registered residential postcode. For each year, we estimated the age- and sex-standardized consultation incidence and prevalence for low back pain and OA for the most deprived 10% of neighbourhoods through to the least deprived 10%. We then calculated the slope index of inequality and relative index of inequality overall and by sex, age group and geographical region.

**Results:**

Inequalities in low back pain incidence and prevalence over socioeconomic status widened between 2004 and 2013 and stabilized between 2014 and 2019. Inequalities in OA incidence remained stable over socioeconomic status within the study period, whereas inequalities in OA prevalence widened markedly over socioeconomic status between 2004 and 2019. The widest gap in low back pain incidence and prevalence over socioeconomic status was observed in populations resident in northern English regions and London and in those of working age, peaking at 45–54 years.

**Conclusion:**

We found persistent, and generally increasing, socioeconomic inequalities in the rate of adults presenting to primary care in England with low back pain and OA between 2004 and 2019.

Key messages

- Socioeconomic inequalities in consultation rates for low back pain and OA persist and have increased in England since 2004.
- Inequalities are more common for low back pain and are wider among women, people of working age and in the north.

## Introduction

The rates of many non-communicable diseases are higher among disadvantaged and marginalized people and communities [1]. Musculoskeletal disorders, such as low back pain, neck pain and OA, are important and increasing causes of disability and societal costs in populations worldwide [2] and show the same pattern, in which the occurrence, severity and impact tend to be inversely related to socioeconomic position [3].

Evidence on the extent of socioeconomic inequalities in the prevalence of musculoskeletal pain disorders comes mainly from cross-sectional population surveys and, to a lesser extent, from cross-sectional analysis of single waves of longitudinal studies, including birth cohorts. Despite heterogeneous case definitions and methods, a consistent finding has emerged of higher prevalence of musculoskeletal pain [4], low back pain [5], hip or knee pain [6, 7], widespread pain [8] and chronic pain in general [9] among adults with lower individual socioeconomic position or living in more deprived neighbourhoods. Inequalities might be greater for some disorders (e.g. back pain) than others (e.g. self-reported and doctor-diagnosed OA) [4]. However, a paucity of repeated survey data on musculoskeletal pain means that it is unclear whether inequalities in musculoskeletal pain, severity and impact are widening or narrowing over time. In England, the current Public Health Outcomes Framework [10] includes one indicator on the prevalence of long-term musculoskeletal problems obtained from the national General Practice Patient Survey and available annually only from 2018. Understanding the long-term health inequalities might help the government’s place-based approaches to support the most deprived areas with the poorest health, in order to narrow the national health inequalities gap [11].

Continuous morbidity recording in primary care might offer an additional source of data to examine trends over time in the magnitude of inequalities at national and subnational levels. Using these data, investigators in other fields have reported growing inequalities by neighbourhood deprivation in the rates of multimorbidity [12], age at first presentation of heart failure [13] and incidence of fracture [14]. To our knowledge, a similar approach has not previously been applied to studying trends over time in inequalities for the most common, disabling musculoskeletal pain conditions. The objective of our study was to determine whether the rate of adults presenting to general practice for low back pain and OA differed by area-level deprivation and whether any such differences have widened or reduced between 2004 and 2019 in England.

## Methods

### Data sources and study population

Clinical Practice Research Datalink (CPRD) Aurum is a database including anonymized data from patient electronic health records in primary care on demographics, diagnoses, symptoms, prescriptions, referrals, immunizations, lifestyle factors, tests and results. Patient-level data linkage to national deprivation measures is used in this study. As of February 2021, CPRD Aurum included data on 39.7 million patients from 1489 practices, of whom 13.3 million currently contribute data (20% of the population of England) [15].

### Neighbourhood deprivation

We used the English index of multiple deprivation (IMD) 2015 rank as a composite measure of neighbourhood deprivation, which combines 37 indicators covering seven domains of material deprivation (health deprivation and disability; barriers to housing and services; employment deprivation; income deprivation; education, skills and training deprivation; crime; living environment deprivation) presented at the level of lower super output area (LSOA; areas with mean population size 1500, minimum 1000) [16, 17]. Our analyses were restricted to English practices in CPRD that consented to the linkage. Individual-level IMD linkage is available for those general practices that agreed to this linkage and where the individual themselves has not opted out, covering ∼70% of CPRD participants. IMD rank was categorized by decile score, where 1 = the least deprived 10% of neighbourhoods and 10 = the most deprived 10% [18].

### Case definitions

Case definitions and definitions of consultation incidence (new cases presenting to general practice) and prevalence (all cases presenting to general practice, including new and ongoing cases) matched those we used previously to determine overall trends in prevalence and incidence of low back pain and OA in CPRD [19]. In UK primary care, symptom and diagnosis problems were recorded using Read codes up to 2018, when SNOMED codes began to replace Read codes. High validity of diagnostic coding has been previously reported [20–22].

Cases of non-specific low back pain among those aged ≥15 years were defined as having at least one recorded coded event of low back pain in a calendar year. We applied a Read code list previously developed [19] to define low back pain. Cases of OA were defined as having at least one recorded clinical event of OA (based on Read codes starting N05 ‘Osteoarthritis and allied disorders’) among those aged ≥45 years in each calendar year.

### Defining the at-risk population

To estimate annual prevalence, the denominator population included all patients with a full registration history over the prior 3 calendar years. In the estimation of annual incidence, the denominator population was restricted to those with no recorded codes of the outcome of interest (low back pain or OA) over the previous 3 years. A 3-year look-back period has previously been shown to be optimal for common musculoskeletal disorders [23]. A period of <3 years might increase the risk of including prevalent cases as new cases, whereas a longer period might increase the risk of selection bias, because patients would need to have been registered at their practice for a longer time to be included in the study. The numerator population incorporated all patients in the denominator population who fulfilled our case definitions above [23].

### Statistical analysis

The annual age- and sex-standardized rates, stratified by deprivation, were estimated using the mid-2019 England population (ONS code: E92000001) as the standard, with 95% CIs estimated by Poisson regression for the whole English population and for the population in each English geographical region between 2004 and 2019. The annual age-standardized incidence and prevalence for men and women by deprivation status were also determined.

The annual incidence and prevalence population-weighted, regression-based slope index of inequality (SII) and relative index of inequality (RII) were estimated [24, 25] (Supplementary Technical Note, available at *Rheumatology Advances in Practice* online). A value of zero on the SII indicates no inequality. Positive values of the SII indicate a higher concentration of low back pain/OA among those in the most deprived areas, whereas negative values indicate a higher concentration among those in the least deprived areas. RII has the value one when there is no inequality. Values of the RII larger than one indicate a higher concentration of low back pain/OA in most deprived areas, whereas values smaller than one indicate a higher concentration in the least deprived areas. SIIs and RIIs were calculated using a standard analytical tool provided by the England Office for Health Improvement and Disparities. The confidence intervals for each SII and RII were estimated using bootstrapping with resampling 10 000 times. Stata MP Version 16.0 (Stata Corporation, College Station, Texas, USA) was used for data management and statistical analyses.

### Ethical approval

The study was approved by the Independent Scientific Advisory Committee for CPRD research (protocol reference: 20_054R). No further ethical permissions were required for the analyses of these anonymized patient-level data.

## Results

**Figure 1.**
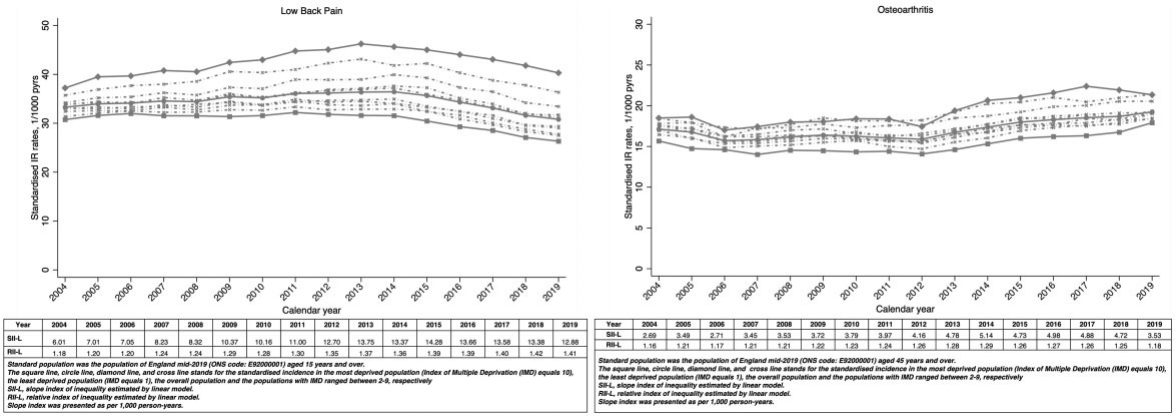
Standardized incidence of low back pain and OA by neighbourhood deprivation

Adults living in more deprived neighbourhoods had higher age- and sex-standardized incidence rates for low back pain than adults living in less deprived neighbourhoods. The gap between annual incidence rates in the most and least deprived neighbourhoods widened between 2004 and 2013, because incidence rates rose among the most deprived while remaining stable among the least deprived (SII rose from 6.01 to 13.75 per 1000 person-years, RII from 1.18 to 1.37). From 2014 to 2019, incidence rates fell across all groups, slightly narrowing the absolute inequality gap but not the relative index of inequality (SII in 2019 = 12.88 per 1000 person-years; RII = 1.41: Fig. 1; Supplementary Tables S1 and S2, available at *Rheumatology Advances in Practice* online). The same pattern was observed for the age- and sex-standardized prevalence of low back pain (Supplementary Fig. S1 and Supplementary Tables S1 and S2, available at *Rheumatology Advances in Practice* online).

Inequalities in the age- and sex-standardized incidence of OA increased between 2004 and 2014 (SII rose from 2.69 to 5.14 per 1000 person-years, RII from 1.16 to 1.29), then decreased to 2019 (SII fell from 5.14 to 3.53 per 1000 person-years, RII from 1.29 to 1.18; Fig. 1; Supplementary Tables S1 and S2, available at *Rheumatology Advances in Practice* online). A similar pattern was seen for age- and sex-standardized prevalence of OA, whereby both SII and RII increased between 2004 and 2016 before slightly decreasing to 2019 (Supplementary Fig. S1 and Supplementary Tables S1 and S2, available at *Rheumatology Advances in Practice* online).

**Figure 2.**
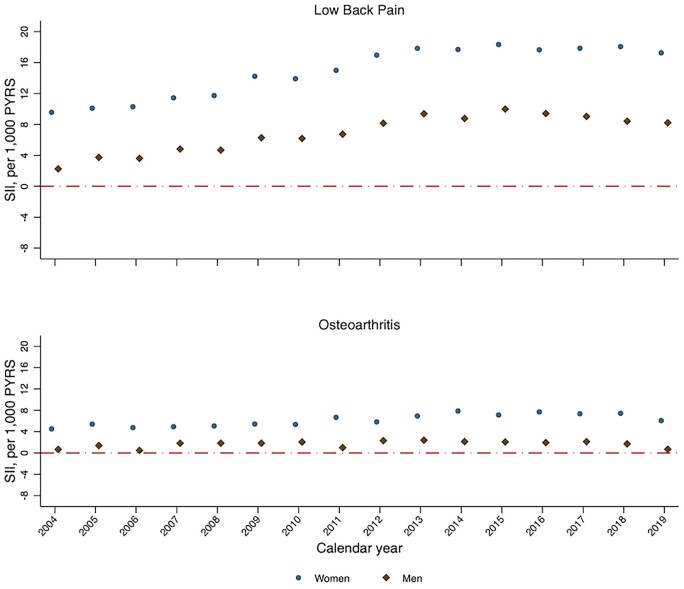
Slope index of inequality for sex-specific standardized incidence of low back pain and OA between 2004 and 2019 in England PYRS: person-years; SII: slope index of inequality

**Figure 3.**
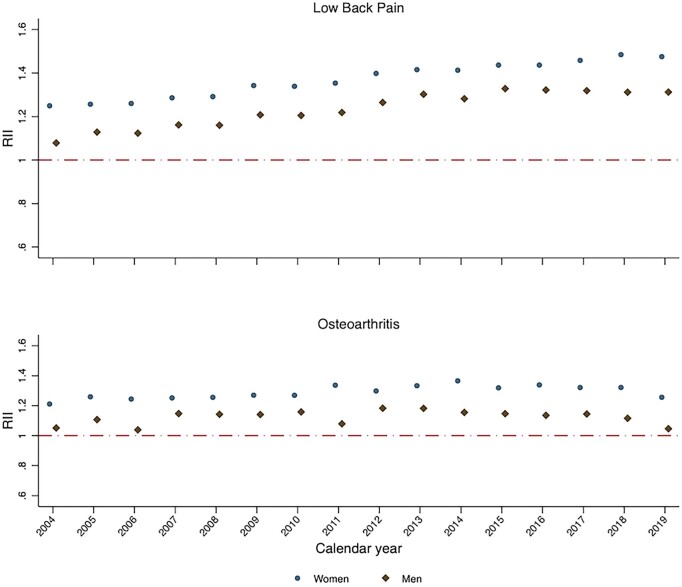
Relative index of inequality for sex-specific standardized incidence of low back pain and OA between 2004 and 2019 in England RII: relative index of inequality

In each year from 2004 to 2019, absolute and relative socioeconomic inequalities in the age-standardized incidence and prevalence of low back pain and OA were higher among women than among men (Figs 2 and 3; Supplementary Figs S2 and S3 and Supplementary Tables S2 and S3, available at *Rheumatology Advances in Practice* online). The trends across time in absolute and relative socioeconomic inequalities for men and for women were broadly similar, following the overall trend.

**Figure 4.**
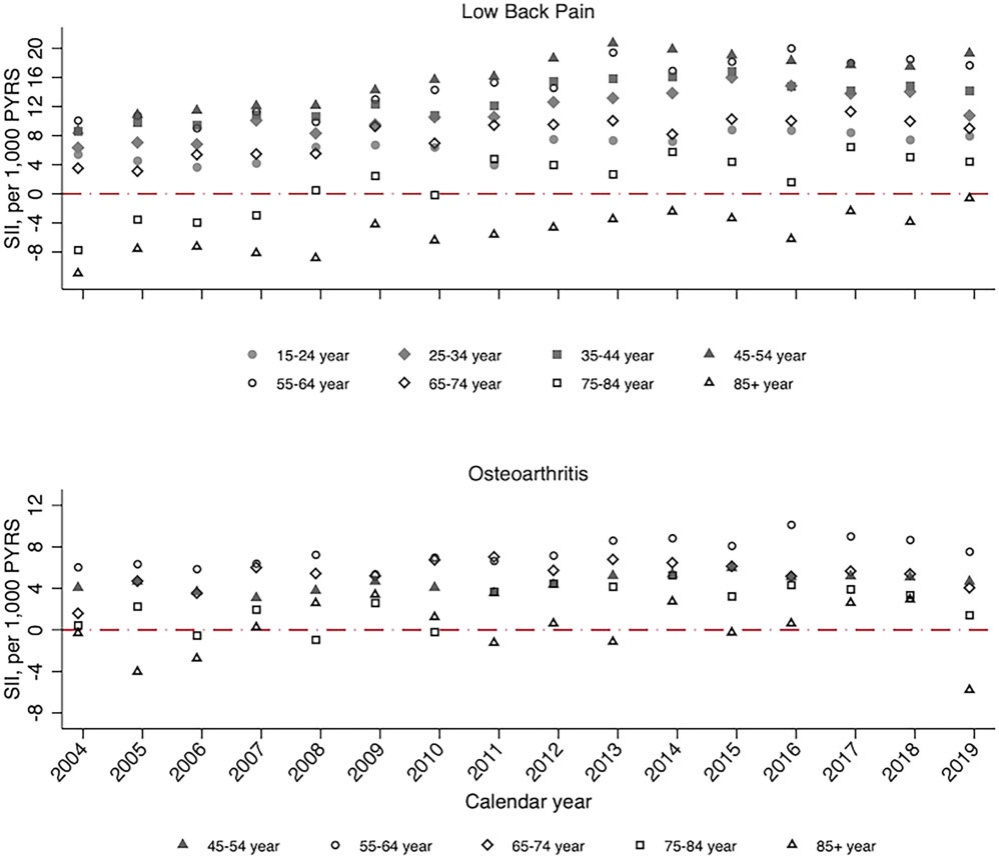
Slope index of inequality for age-stratified standardized incidence of low back pain and OA between 2004 and 2019 in England PYRS: person-years; SII: slope index of inequality

**Figure 5.**
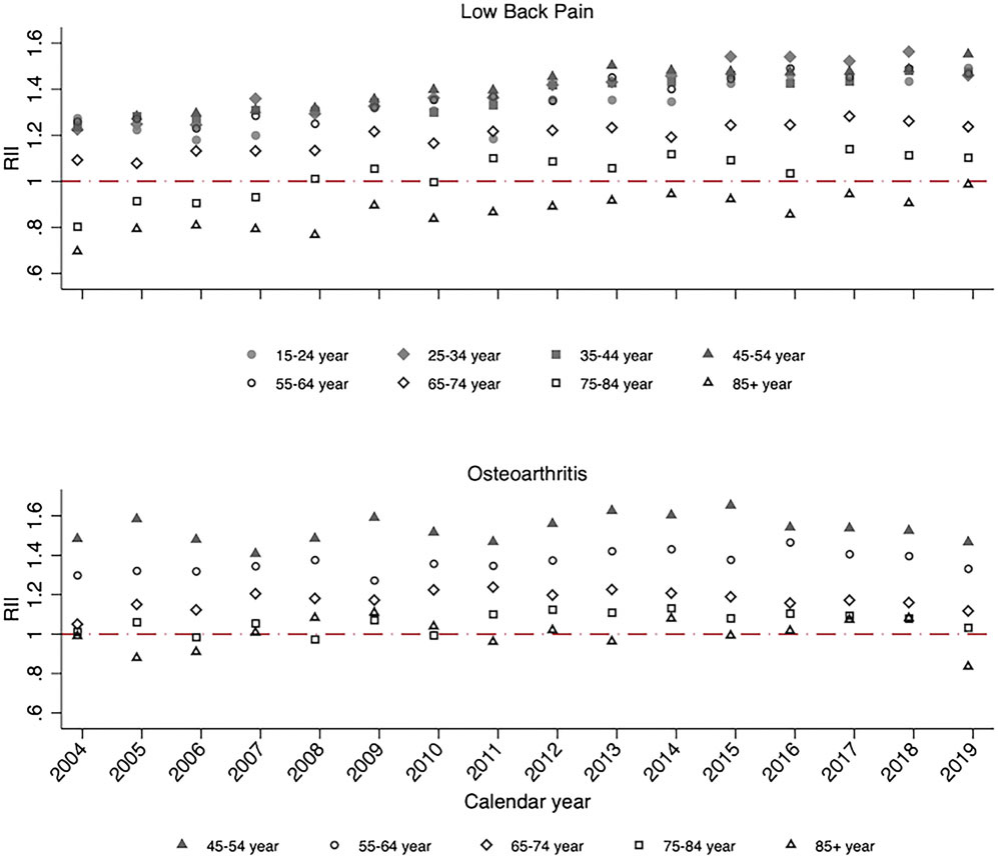
Relative index of inequality for age-stratified standardized incidence of low back pain and OA between 2004–2019 in England RII: relative index of inequality

In age-stratified analyses, socioeconomic inequalities for low back pain and OA incidence and prevalence rates were greatest in adults <65 years of age (Figs 4 and 5; Supplementary Figs S4 and S5 and Supplementary Tables S4 and S5, available at *Rheumatology Advances in Practice* online). Consistent with the overall trend over time, relative socioeconomic inequalities in low back pain incidence increased over time within all age groups from 15 to 64 years. Among 75- to 84-year-olds and those >85 years of age, a much greater increase in the incidence and prevalence of low back pain between 2004 and 2019 was seen among those living in the most deprived neighbourhoods compared with the least deprived. For OA, relative socioeconomic inequalities in both incidence and prevalence were associated with age group, with RII across 2004–2019 consistently being highest in the age category of 45–54 years and lowest in adults aged ≥75 years.

### Region-specific trends

**Figure 6.**
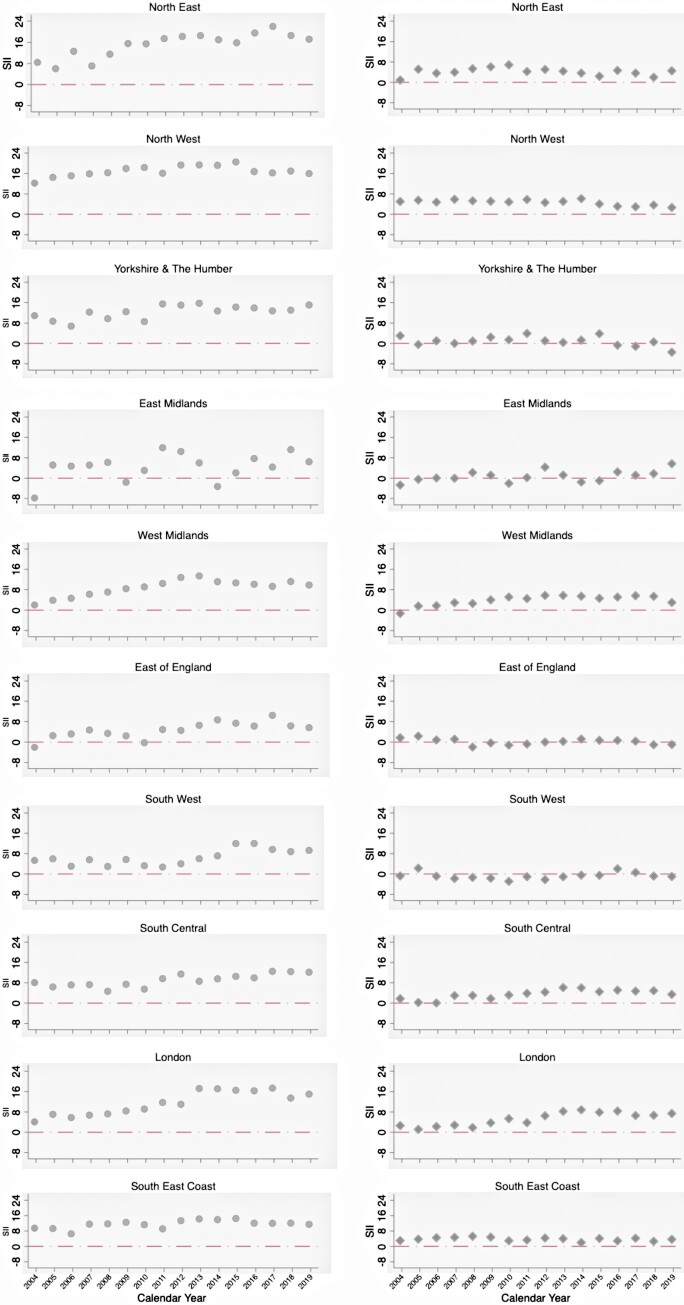
Slope index of inequality for standardized incidence of low back pain and OA between 2004 and 2019 in each of 10 English regions Dots and diamonds indicate SII for low back pain and OA, respectively. PYRS: person-years; SII: slope index of inequality

Similar trends of SIIs and RIIs for age- and sex-standardized incidence and prevalence by geographical region were identified, with generally greater socioeconomic inequalities in the North West and North East, for both low back pain and OA (Fig. 6; Supplementary Figures S6–S8 and Supplementary Tables S6 and S7, available at *Rheumatology Advances in Practice* online). Over the study period, the socioeconomic gap in incidence and prevalence widened in several regions, especially for low back pain. For example, in the North East, the estimate of SII for low back pain incidence widened from 8.48 in 2004 to 17.13 per 1000 person-years in 2019. In London, the corresponding increases were from 4.15 to 15.03 per 1000 person-years. In comparison, in South Central, SII increased less, from 8.13 to 12.09 per 1000 person-years. Under-representation of general practices from the East Midlands resulted in unstable region-specific estimates for that region.

## Discussion

### Main findings

Our descriptive study found evidence of persistent socioeconomic inequalities in the annual rate of recorded cases of low back pain and OA presenting to primary care in England over the 16-year period between 2004 and 2019. Consultation rates were, in general, between 15 and 55% higher among adults living in the most deprived decile of neighbourhoods compared with those living in the least deprived decile. Inequalities were generally greater for low back pain than for OA and were greatest among women, adults under the age of statutory retirement and in northern regions and London. Overall, absolute and relative inequalities widened in the period between 2004 and 2013, although this pattern was not observed consistently in stratified analyses. These inequalities have not reduced since 2013.

### Comparison with previous studies

Our estimates of the direction and magnitude of relative inequalities for these two common musculoskeletal conditions are broadly consistent with available national survey data from 2018–2020 on deprivation-specific prevalence of self-reported long-term back pain or joint pain and chronic pain. These sources respectively suggest a 20–30% and a 36% higher prevalence among adults living in the most deprived neighbourhoods. There are few published estimates of sex-, age- and region-specific inequalities for direct comparison. Our study found greater socioeconomic inequalities for low back pain and OA among women than among men. Higher levels of opportunistic consultation and coding of OA, especially among women living in more deprived settings, might contribute to this. Women have higher levels of multimorbidity [26] and more contacts with primary care [27], and there might be a stronger gradient in consultation rates by deprivation among women [28]. We are unaware of previous studies finding greater, and widening, inequalities in musculoskeletal conditions among young- and middle-aged adults, and this warrants further investigation. However, this pattern, and the absence or reversal of inequalities in old age, was also found for multimorbidity rates by an independent research group using the same data source [12].

Our study did not explore potential mechanisms underlying the observed inequalities, but future research to explicate further how exposure to inequitable social structures and systems becomes embodied as OA would be valuable. We hypothesize that persistent inequalities in the rate of new diagnoses of low back pain and OA are likely to arise, at least in part, from inequalities in the distribution of one or more key proximal causal exposures, including obesity, occupational physical exposures, injury, physical inactivity and mood. The causal action of some of these exposures begins earlier in life and might be cumulative over many years [29–32], implying the need for earlier and sustained equity-focused prevention to reduce the inequalities in OA incidence seen in middle age.

Relying on coded diagnoses in the primary care electronic health record to define a case of OA does not provide an unfiltered measure of disease incidence in the population; it also reflects the propensity to consult, access to primary care, and coding behaviours among primary health-care professionals. Inequalities by deprivation in these factors might also contribute to observed inequalities in consultation incidence and prevalence. It is interesting that the period during which we observed the clearest widening of inequalities in low back pain/OA incidence/prevalence coincided with when there appeared to have been success in achieving a more equitable supply of general practitioners [33, 34]. This apparent paradox could reflect better access or more complete problem coding in deprived areas when there is a greater supply of general practitioners.

### Strengths and limitations

Our study used established code lists and a recognized area-level measure of deprivation based on patients’ postcodes applied to a large primary care electronic health record database representative of the English population [35]. Individual-level measures of socioeconomic position, such as educational attainment, occupation or income, are not routinely recorded or available. The result is that our analyses are based on the marker ‘living in a deprived area’ rather than being socioeconomically disadvantaged. Moscrop *et al.* [36] argue that this can result in underestimation of ‘true’ socioeconomic inequalities and can obscure the real social determinants responsible for the observed inequalities. Underestimation of inequalities might also result from analytical decisions. We modelled the slope index of inequality and relative index of inequality as a linear function, hence assuming a linear relationship between indicator and population socioeconomic status. This might be suboptimal in situations where the relationship between indicator and deprivation is non-linear. Future methodological exploration of optimal models to fit for non-linear relationships are warranted. Owing to restricted access to clinical records and measurements in the denominator population, confounding effects from obesity and multimorbidity on the research findings were not explored further in the present study. Future research to test the effects of these confounders is warranted. We relied on a clinician-coded record of OA rather than need for radiographic evidence. Clinical guidance suggests that non-radiographic features alone are sufficient to make a diagnosis for OA [37, 38], and a previous study revealed good specificity of general practitioner-diagnosed OA [39]. Studies based on electronic health records might be subject to misclassification that has the potential to bias results. However, in the present study, the established codes list and methods used to estimate incidence and prevalence have been validated and yielded internationally comparable estimations [23].

### Conclusion

In conclusion, the present study found persistent and, in some cases, widening inequalities by deprivation in the rates of two of the most common, disabling musculoskeletal conditions presenting to primary care in England between 2004 and 2019.

## Supplementary Material

rkac106_Supplementary_Data

## Data Availability

Data may be obtained from a third party and are not publicly available. The data were obtained from the Clinical Practice Research Datalink (CPRD). CPRD data governance does not allow us to distribute patient data to other parties. Researchers may apply for data access at: http://www.CPRD.com/
