# Supplementary material for: Persistent inequalities in consultation incidence and prevalence of low back pain and osteoarthritis in England between 2004 and 2019
Source: Rheumatol Adv Pract. 2022 Dec 2;7(1):rkac106. doi: 10.1093/rap/rkac106 (PMC9800855; doi:10.1093/rap/rkac106)

Supplemental Figure S1. Standardised prevalence of low back pain and osteoarthritis in least and most deprived English population

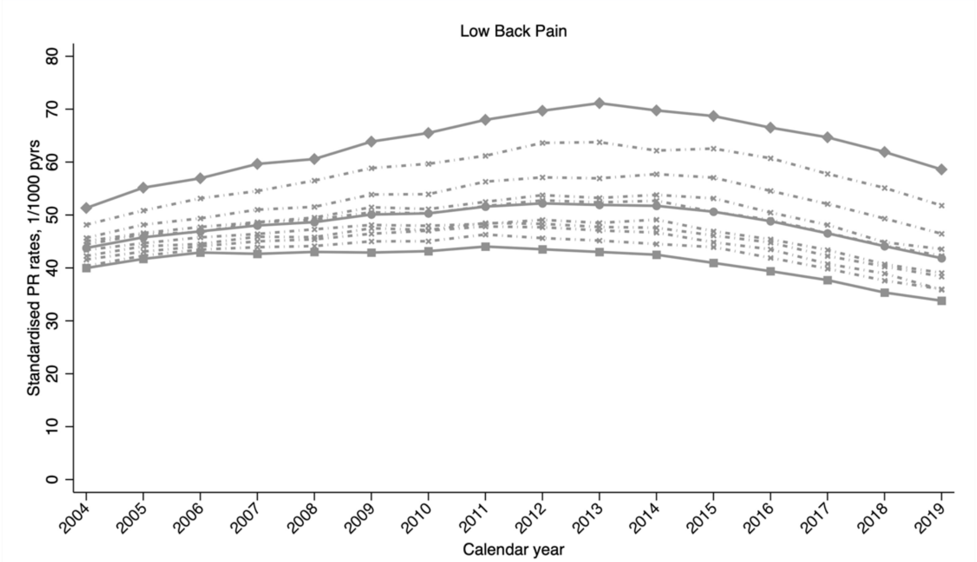

Year	2004	2005	2006	2007	2008	2009	2010	2011	2012	2013	2014	2015	2016	2017	2018	2019
SII-L	10.92	12.43	13.22	15.47	16.70	19.60	20.27	21.65	24.80	26.08	25.91	27.04	26.28	25.86	24.97	23.39
RII-L	1.25	1.27	1.28	1.32	1.34	1.38	1.40	1.41	1.47	1.49	1.49	1.52	1.53	1.54	1.55	1.55

Standard population was the population of England mid-2019 (ONS code: E92000001) aged 15 years and over.
The blue square line, black circle line, read diamond line, and grey cross line stands for the standardised incidence in the most deprived population (Index of Multiple Deprivation (IMD) equals 10), the least deprived population (IMD equals 1), the overall population and the populations with IMD ranged between 2-9, respectively
SII-L, slope index of inequality estimated by linear model.
RII-L, relative index of inequality estimated by linear model.
Slope index was presented as per 1,000 person-years.

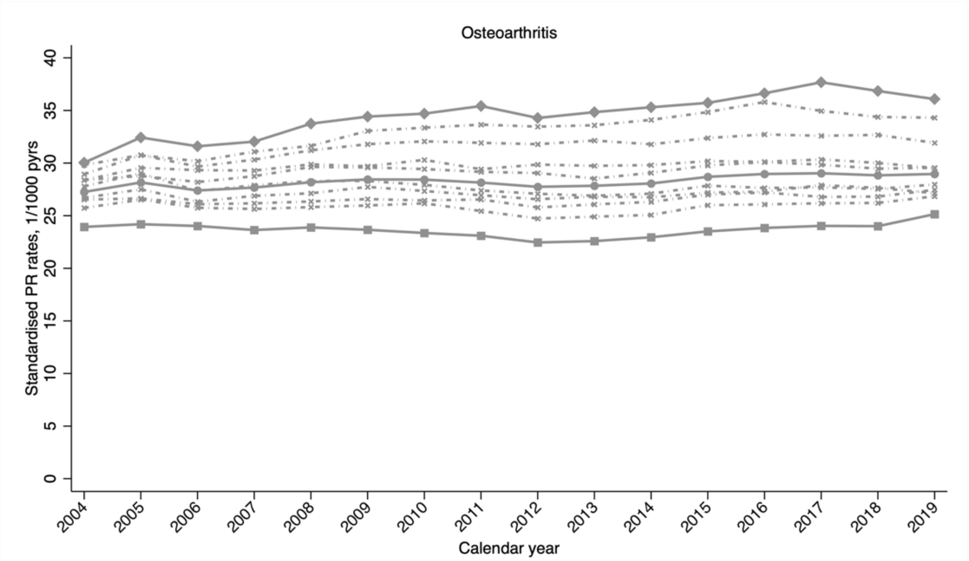

Year	2004	2005	2006	2007	2008	2009	2010	2011	2012	2013	2014	2015	2016	2017	2018	2019
SII-L	6.19	7.75	7.52	8.48	9.60	10.41	11.06	11.73	12.21	12.20	12.23	12.10	12.63	12.40	11.80	10.08
RII-L	1.22	1.27	1.27	1.30	1.33	1.36	1.38	1.40	1.43	1.42	1.42	1.41	1.42	1.41	1.40	1.34

Standard population was the population of England mid-2019 (ONS code: E92000001) aged 45 years and over.
The blue square line, black circle line, read diamond line, and grey cross line stands for the standardised incidence in the most deprived population (Index of Multiple Deprivation (IMD) equals 10), the least deprived population (IMD equals 1), the overall population and the populations with IMD ranged between 2-9, respectively
SII-L, slope index of inequality estimated by linear model.
RII-L, relative index of inequality estimated by linear model.
Slope index was presented as per 1,000 person-years.

Supplemental Figure S2. Slope index of inequality for sex-specific standardised prevalence of low back pain and osteoarthritis between 2004-2019 in England

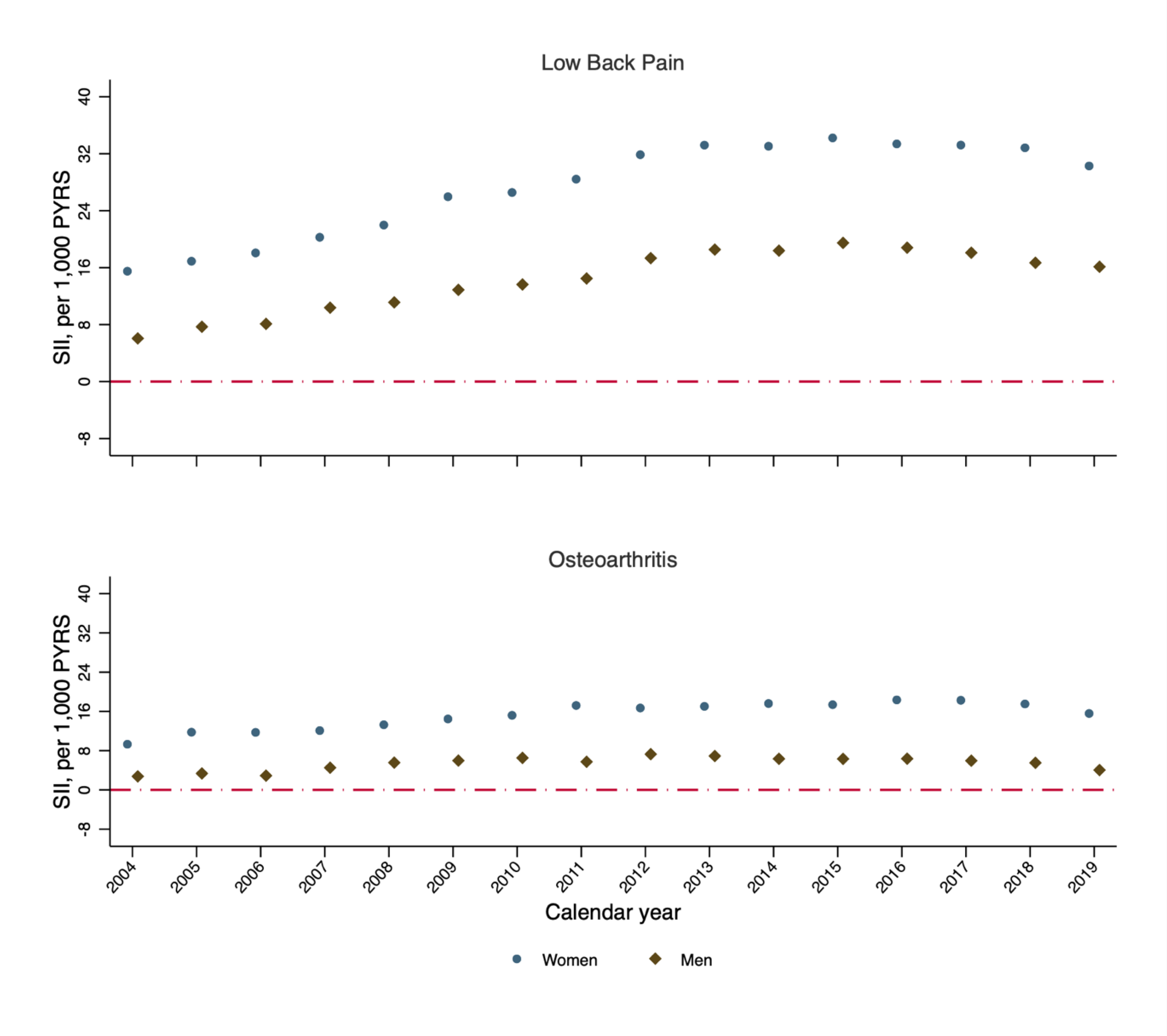

Supplemental Figure S3. Relative index of inequality for overall standardised prevalence of low back pain and osteoarthritis between 2004-2019 in England

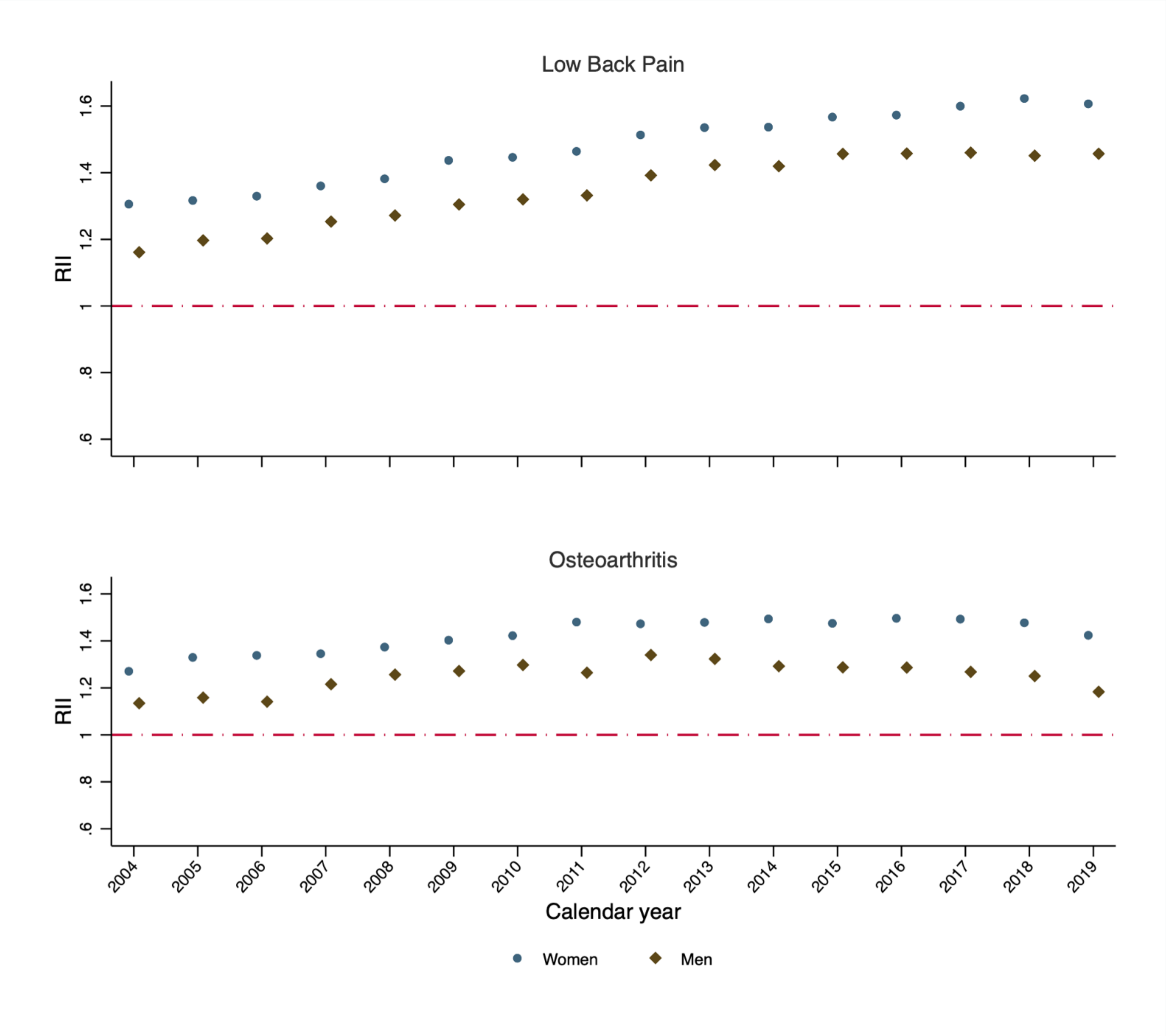

Supplemental Figure S4. Slope index of inequality for prevalence of low back pain and osteoarthritis by age-strata between 2004-2019

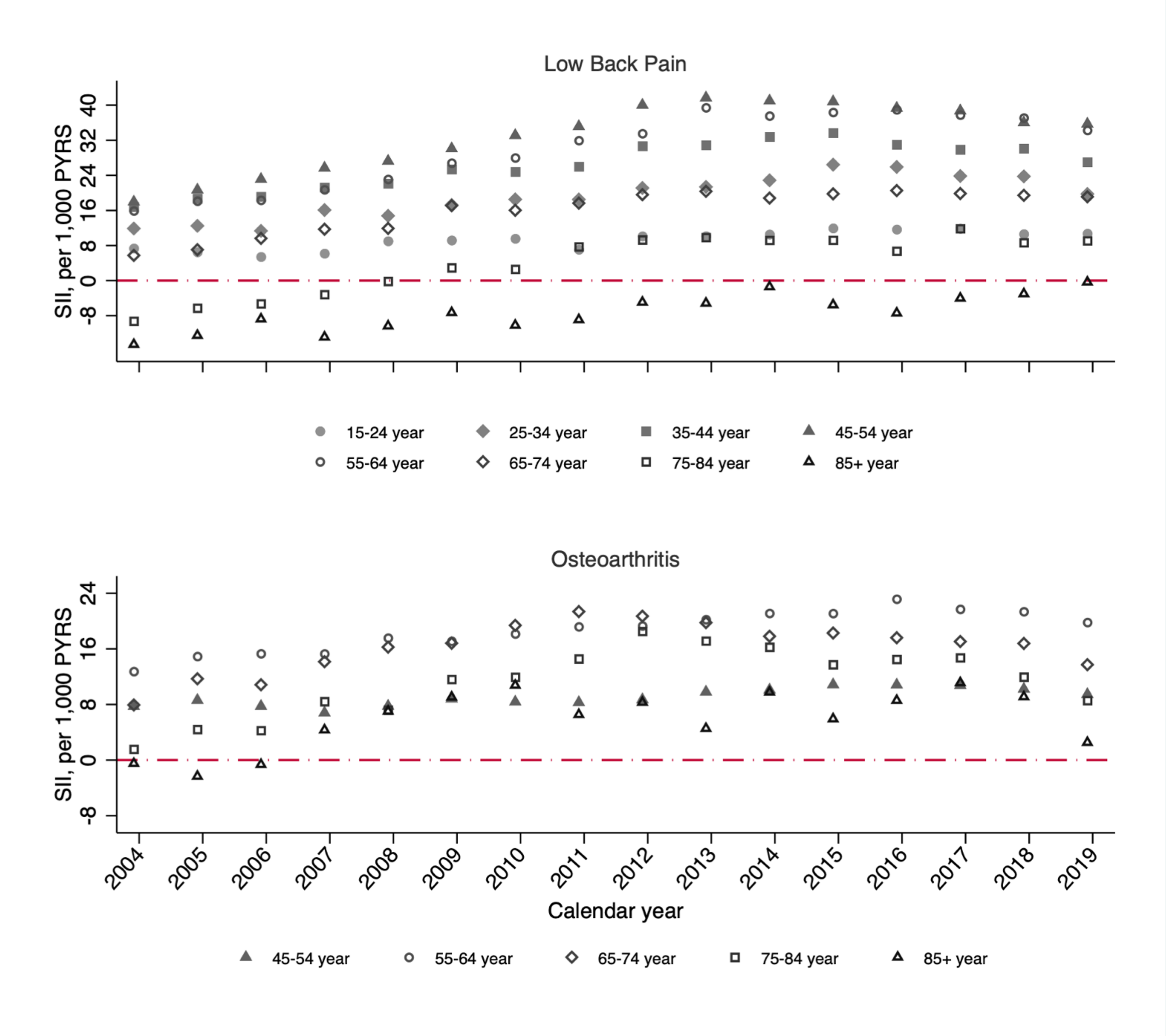

Supplemental Figure S5. Relative index of inequality for prevalence of low back pain and osteoarthritis by age-strata between 2004-2019

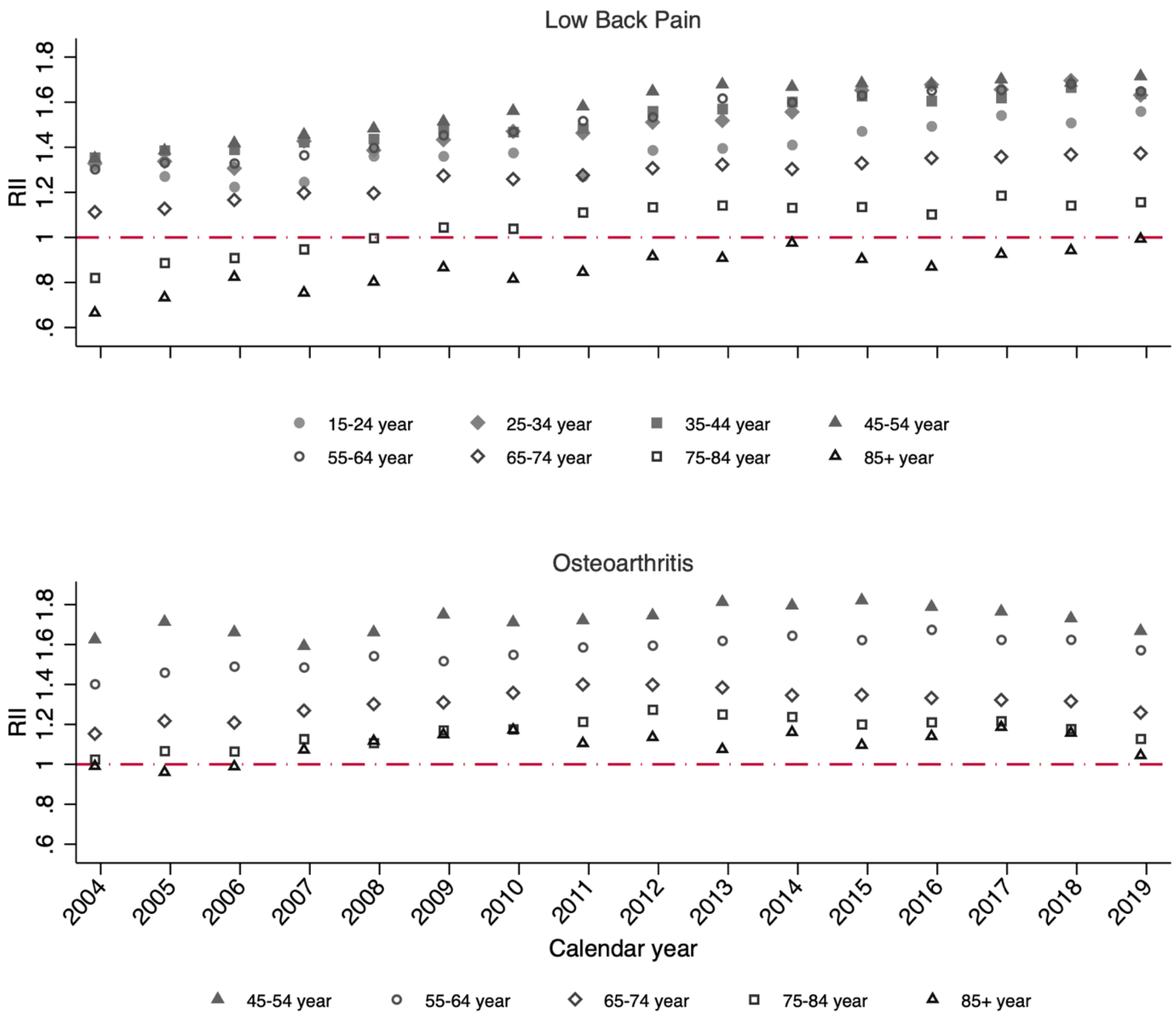

Supplemental Figure S6. Relative index of inequality of standardised incidence of low back pain and osteoarthritis by geographical region between 2004-2019
Dot and diamond indicate RII for low back pain and osteoarthritis, respectively.

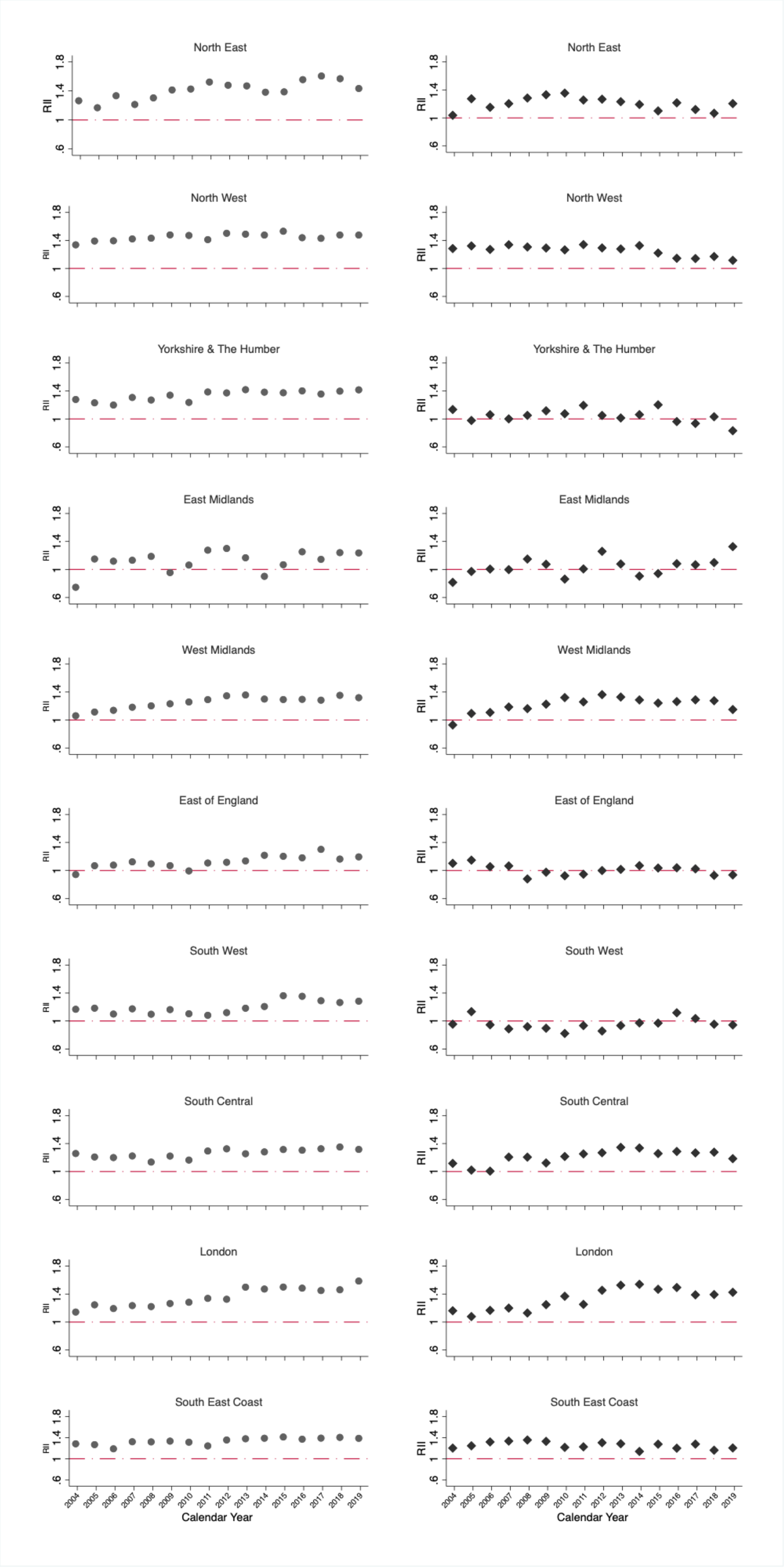

Supplemental Figure S7. Slope index of inequality of standardised prevalence of low back pain and osteoarthritis by geographical region between 2004-2019

Dot and diamond indicate SII for low back pain and osteoarthritis, respectively.

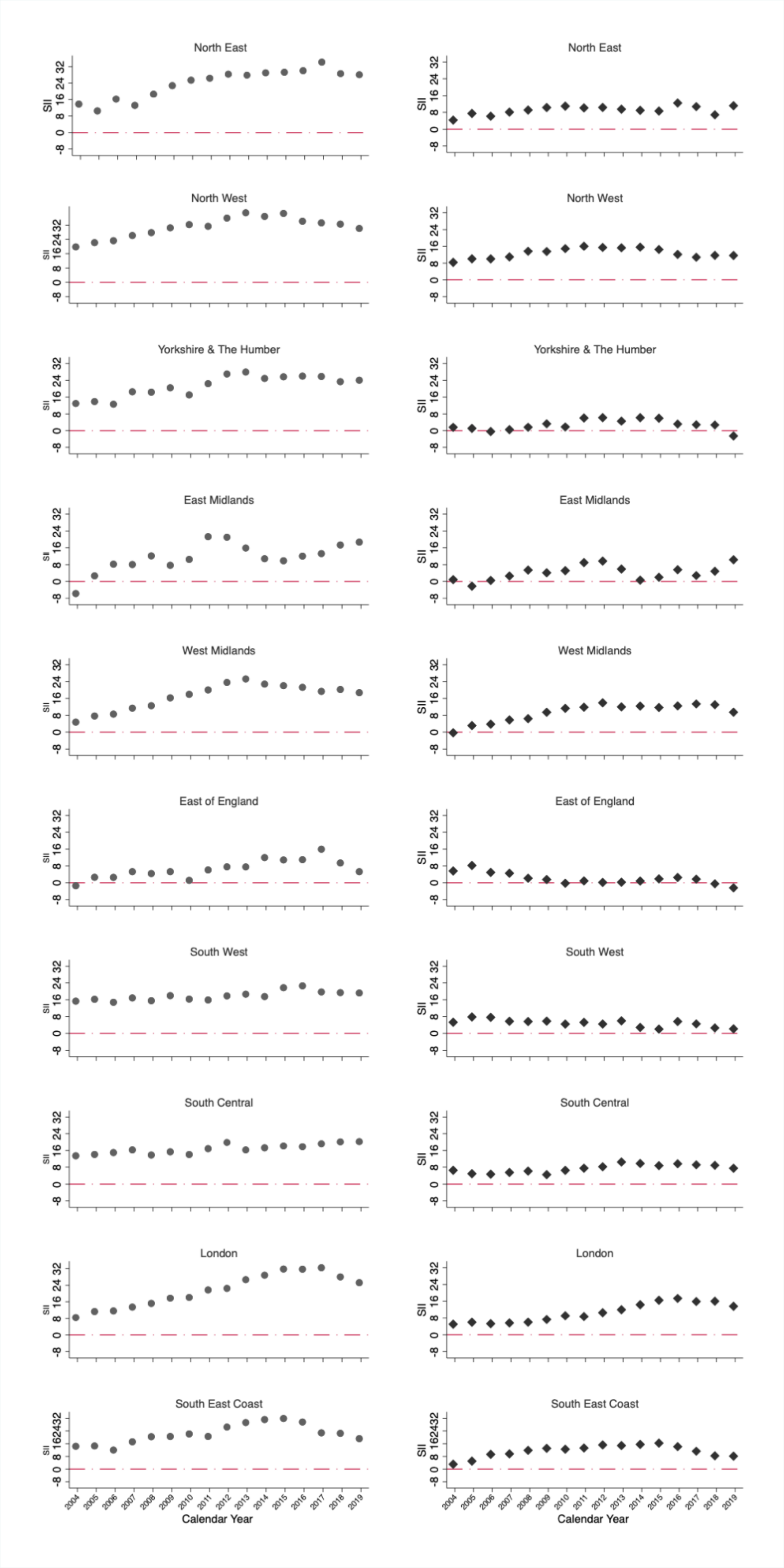

Supplemental Figure S8. Relative index of inequality of standardised prevalence of low back pain and osteoarthritis by geographical region between 2004-2019

Dot and diamond indicate RII for low back pain and osteoarthritis, respectively.

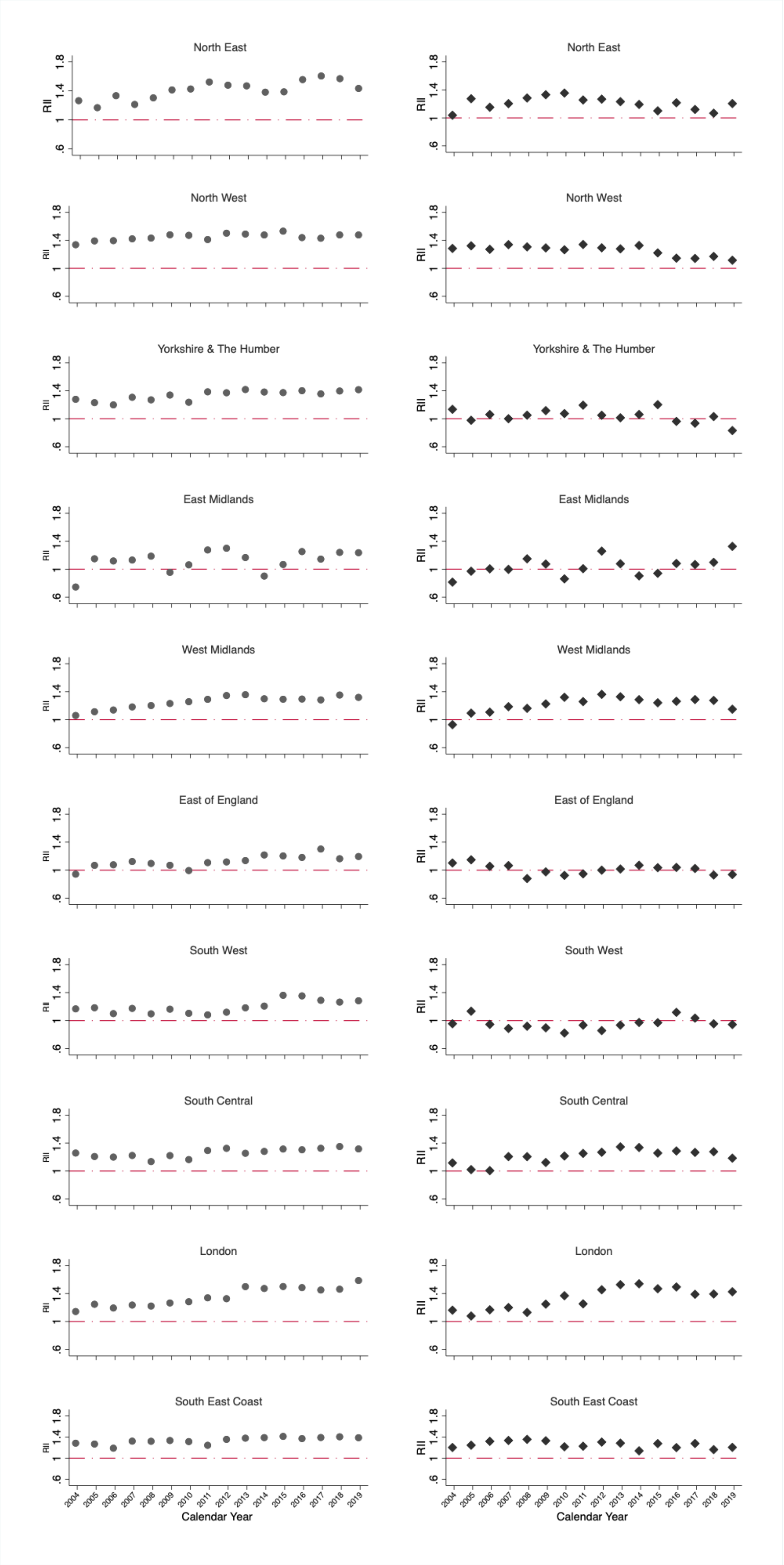

Supplement: rkac106_Supplementary_Data [file rkac106_supplementary_data.zip › 22-113 Supplementary Figures.pdf]
