# Supplementary material for: Persistent inequalities in consultation incidence and prevalence of low back pain and osteoarthritis in England between 2004 and 2019
Source: Rheumatol Adv Pract. 2022 Dec 2;7(1):rkac106. doi: 10.1093/rap/rkac106 (PMC9800855; doi:10.1093/rap/rkac106)
Supplement: rkac106_Supplementary_Data [file rkac106_supplementary_data.zip › 22-113 Supplementary Tables.pdf]

Supplementary Table S1. Standardised incidence and prevalence of low back pain and osteoarthritis by index of multiple derivation in England between 2004-2019

For low back pain: the standard population was the population of England 2019 (ONS code: E92000001) aged 15 years and over; for osteoarthritis: the standard population was the population of England 2019 (ONS code: E92000001) aged 45 years and over.
IMD indicates index of multiple deprivation STDIR indicates standardised incidence rates; STDPR indicates standardised prevalence; CI indicates confidence interval.

Occurrence	Condition	Calendar Year	IMD decile	Numerator	Denominator	STDIR/STDPR (95 % CI)
Incidence	Low back pain	2004	Least Deprivation	14732	463670.8	30.76 (30.71 to 30.81)
			IMD=2	14452	447360.5	31.39 (31.34 to 31.44)
			IMD=3	14255	428353.7	32.44 (32.38 to 32.49)
			IMD=4	12985	386326.7	32.97 (32.92 to 33.02)
			IMD=5	12468	371708	33.03 (32.98 to 33.08)
			IMD=6	12644	370589.1	33.75 (33.70 to 33.80)
			IMD=7	12755	375145.8	33.93 (33.88 to 33.98)
			IMD=8	10894	321701.3	34.21 (34.15 to 34.26)
			IMD=9	11993	342129.2	35.73 (35.67 to 35.78)
			Most Deprivation	11059	305397.1	37.22 (37.16 to 37.27)
		2005	Least Deprivation	15165	466933	31.60 (31.55 to 31.65)
			IMD=2	14749	448301.5	32.08 (32.03 to 32.14)
			IMD=3	14406	430716.1	32.57 (32.51 to 32.62)
			IMD=4	13122	387033.2	33.35 (33.30 to 33.41)
			IMD=5	12525	374392.4	32.94 (32.89 to 32.99)
			IMD=6	12877	371674.9	34.44 (34.39 to 34.49)
			IMD=7	12907	377825.2	34.22 (34.17 to 34.27)
			IMD=8	11267	323309.4	35.21 (35.16 to 35.27)
			IMD=9	12381	343151.1	36.91 (36.86 to 36.97)
			Most Deprivation	11768	307344	39.50 (39.44 to 39.56)
		2006	Least Deprivation	15631	474115.1	32.00 (31.95 to 32.05)
			IMD=2	15136	453369.3	32.50 (32.45 to 32.56)
			IMD=3	14518	435291.3	32.54 (32.49 to 32.59)
			IMD=4	13138	391469.3	32.91 (32.86 to 32.97)
			IMD=5	12834	379571	33.39 (33.34 to 33.44)
			IMD=6	12886	375549.7	34.02 (33.96 to 34.07)
			IMD=7	13041	381472.6	34.30 (34.24 to 34.35)
			IMD=8	11336	325679.9	35.40 (35.35 to 35.46)
			IMD=9	12704	346553.2	37.70 (37.64 to 37.75)
			Most Deprivation	11901	310994.2	39.68 (39.62 to 39.74)
		2007	Least Deprivation	15534	479128.3	31.54 (31.48 to 31.59)
			IMD=2	15206	458000.5	32.24 (32.19 to 32.30)
			IMD=3	14933	437479.9	33.27 (33.22 to 33.32)
			IMD=4	13576	394513	33.81 (33.76 to 33.87)
			IMD=5	12963	383182.5	33.38 (33.33 to 33.43)
			IMD=6	13474	378667.8	35.27 (35.21 to 35.32)
			IMD=7	13234	384717.3	34.57 (34.52 to 34.62)
			IMD=8	11725	328972.9	36.25 (36.20 to 36.31)

				IMD=9	12929	349562.4	38.00 (37.94 to 38.05)
				Most Deprivation	12338	313643.7	40.79 (40.73 to 40.84)
			2008	Least Deprivation	15887	488385.7	31.52 (31.47 to 31.57)
				IMD=2	15454	465760.7	32.28 (32.23 to 32.33)
				IMD=3	14928	445372	32.71 (32.66 to 32.76)
				IMD=4	13541	400522.1	33.26 (33.21 to 33.32)
				IMD=5	13351	389820	33.87 (33.82 to 33.93)
				IMD=6	13402	384791.8	34.69 (34.64 to 34.75)
				IMD=7	13465	393029.3	34.40 (34.35 to 34.45)
				IMD=8	11762	335432.7	35.78 (35.72 to 35.83)
				IMD=9	13300	355296	38.57 (38.52 to 38.63)
				Most Deprivation	12409	317540	40.54 (40.48 to 40.60)
			2009	Least Deprivation	15998	495295	31.36 (31.31 to 31.42)
				IMD=2	15829	470026.6	32.78 (32.73 to 32.83)
				IMD=3	15642	451988.1	33.69 (33.64 to 33.74)
				IMD=4	14157	405116	34.49 (34.44 to 34.54)
				IMD=5	13653	394518.1	34.25 (34.19 to 34.30)
				IMD=6	14131	389519.6	36.05 (35.99 to 36.10)
				IMD=7	14155	398226.2	35.80 (35.75 to 35.86)
				IMD=8	12432	339446.6	37.34 (37.29 to 37.40)
				IMD=9	14064	358875.5	40.57 (40.51 to 40.63)
				Most Deprivation	13024	318211	42.44 (42.38 to 42.50)
			2010	Least Deprivation	16509	505531	31.57 (31.52 to 31.62)
				IMD=2	16028	478665.9	32.66 (32.61 to 32.71)
				IMD=3	15956	460058.8	33.79 (33.74 to 33.84)
				IMD=4	14131	411531.5	33.78 (33.73 to 33.83)
				IMD=5	13675	401888.1	33.64 (33.59 to 33.69)
				IMD=6	14106	396391.7	35.31 (35.26 to 35.37)
				IMD=7	14144	405442.3	35.16 (35.10 to 35.21)
				IMD=8	12596	346543.7	37.08 (37.02 to 37.13)
				IMD=9	14257	364813.2	40.37 (40.31 to 40.43)
				Most Deprivation	13285	320972.4	42.98 (42.92 to 43.04)
			2011	Least Deprivation	17088	513255.5	32.20 (32.15 to 32.26)
				IMD=2	16662	484638.9	33.36 (33.31 to 33.41)
				IMD=3	16486	466337.9	34.48 (34.43 to 34.53)
				IMD=4	14821	417122.4	34.91 (34.86 to 34.96)
				IMD=5	14175	407020.7	34.38 (34.33 to 34.44)
				IMD=6	14666	401723	36.22 (36.17 to 36.28)
				IMD=7	14727	411358	35.97 (35.92 to 36.03)
				IMD=8	13388	351579.8	38.95 (38.89 to 39.00)
				IMD=9	14662	369172.5	41.00 (40.94 to 41.06)
				Most Deprivation	13867	323338	44.78 (44.72 to 44.84)
			2012	Least Deprivation	17110	518371.6	31.82 (31.76 to 31.87)
				IMD=2	16518	489718.6	32.74 (32.69 to 32.80)
				IMD=3	16331	469773.8	33.80 (33.74 to 33.85)

			IMD=4	14735	421692	34.32 (34.27 to 34.38)
			IMD=5	14448	411292.5	34.66 (34.61 to 34.71)
			IMD=6	14953	406157.7	36.63 (36.57 to 36.68)
			IMD=7	15246	415351.8	36.87 (36.82 to 36.93)
			IMD=8	13597	357107.2	38.90 (38.84 to 38.95)
			IMD=9	15336	373649.7	42.31 (42.25 to 42.37)
			Most Deprivation	14134	326808	45.06 (45.00 to 45.12)
		2013	Least Deprivation	17007	520281.2	31.61 (31.56 to 31.66)
			IMD=2	16601	491183.4	32.86 (32.80 to 32.91)
			IMD=3	16253	470231.6	33.61 (33.56 to 33.66)
			IMD=4	14895	422732.7	34.50 (34.45 to 34.55)
			IMD=5	14514	411797.9	34.78 (34.73 to 34.84)
			IMD=6	15016	404736	36.91 (36.86 to 36.97)
			IMD=7	15256	413148.6	37.15 (37.09 to 37.20)
			IMD=8	13598	356095.7	38.96 (38.90 to 39.02)
			IMD=9	15482	370296.2	43.13 (43.07 to 43.19)
			Most Deprivation	14388	323939.8	46.25 (46.18 to 46.31)
		2014	Least Deprivation	17135	523402.5	31.55 (31.50 to 31.60)
			IMD=2	16767	493747.7	32.90 (32.84 to 32.95)
			IMD=3	16590	472709.5	34.01 (33.96 to 34.07)
			IMD=4	14756	424619.3	34.06 (34.00 to 34.11)
			IMD=5	14719	414380.1	34.96 (34.91 to 35.01)
			IMD=6	15214	407081.1	37.19 (37.13 to 37.25)
			IMD=7	15520	415214.7	37.62 (37.57 to 37.68)
			IMD=8	14072	359386.9	39.95 (39.89 to 40.00)
			IMD=9	15176	373423.4	41.84 (41.78 to 41.90)
			Most Deprivation	14263	324783.8	45.64 (45.58 to 45.70)
		2015	Least Deprivation	16771	530584.2	30.46 (30.41 to 30.51)
			IMD=2	16768	498204.9	32.51 (32.46 to 32.56)
			IMD=3	15941	478032.7	32.39 (32.34 to 32.45)
			IMD=4	14597	429605.7	33.29 (33.24 to 33.34)
			IMD=5	14238	418975.8	33.47 (33.42 to 33.52)
			IMD=6	14868	412090.2	35.96 (35.90 to 36.01)
			IMD=7	15581	421210.7	37.28 (37.23 to 37.34)
			IMD=8	14109	366573.5	39.29 (39.23 to 39.34)
			IMD=9	15633	381719.2	42.24 (42.18 to 42.30)
			Most Deprivation	14329	330750.3	45.01 (44.95 to 45.07)
		2016	Least Deprivation	16553	540043.8	29.30 (29.25 to 29.35)
			IMD=2	16248	506711.8	30.87 (30.82 to 30.92)
			IMD=3	15882	486908.8	31.65 (31.60 to 31.70)
			IMD=4	14514	438028.4	32.45 (32.40 to 32.50)
			IMD=5	14106	428020.1	32.43 (32.37 to 32.48)
			IMD=6	14701	421236.9	34.71 (34.65 to 34.76)
			IMD=7	15047	431764.3	35.14 (35.09 to 35.20)
			IMD=8	13738	377402.3	37.32 (37.26 to 37.37)

			IMD=9	15318	393176.2	40.32 (40.27 to 40.38)
			Most Deprivation	14382	339785.2	44.04 (43.98 to 44.10)
		2017	Least Deprivation	16401	549686	28.53 (28.48 to 28.58)
			IMD=2	15861	515425.1	29.69 (29.64 to 29.74)
			IMD=3	15413	496699.7	30.06 (30.01 to 30.11)
			IMD=4	14166	446801.5	30.99 (30.94 to 31.04)
			IMD=5	14029	436030.9	31.61 (31.56 to 31.66)
			IMD=6	14419	430645.7	33.28 (33.23 to 33.34)
			IMD=7	14917	442415.7	33.96 (33.91 to 34.02)
			IMD=8	13775	388542.3	36.46 (36.40 to 36.51)
			IMD=9	15155	406056.6	38.79 (38.73 to 38.85)
			Most Deprivation	14425	349267.2	43.06 (43.00 to 43.12)
		2018	Least Deprivation	16013	563908.1	27.09 (27.04 to 27.14)
			IMD=2	15562	529716	28.27 (28.23 to 28.32)
			IMD=3	15294	513611.2	28.79 (28.74 to 28.84)
			IMD=4	14062	462940.8	29.66 (29.61 to 29.71)
			IMD=5	13574	451469.9	29.55 (29.50 to 29.60)
			IMD=6	14313	443641	32.04 (31.99 to 32.09)
			IMD=7	14458	458585.4	31.83 (31.78 to 31.89)
			IMD=8	13396	403525.1	34.21 (34.15 to 34.26)
			IMD=9	15361	421962.5	37.79 (37.73 to 37.85)
			Most Deprivation	14397	360205	41.80 (41.74 to 41.86)
		2019	Least Deprivation	15687	567554.6	26.33 (26.29 to 26.38)
			IMD=2	15247	531669.1	27.51 (27.46 to 27.56)
			IMD=3	14649	507951.6	27.76 (27.71 to 27.81)
			IMD=4	13806	459215.6	29.40 (29.35 to 29.45)
			IMD=5	13463	454569.1	29.03 (28.98 to 29.08)
			IMD=6	13993	446485.1	31.14 (31.09 to 31.19)
			IMD=7	14504	462843.2	31.71 (31.66 to 31.76)
			IMD=8	13134	404470	33.44 (33.39 to 33.50)
			IMD=9	14853	426488.5	36.35 (36.29 to 36.40)
			Most Deprivation	13568	351573.3	40.31 (40.25 to 40.36)
	Osteoarthritis	2004	Least Deprivation	5508	291893.2	15.67 (15.63 to 15.72)
			IMD=2	5615	275182.1	16.45 (16.41 to 16.50)
			IMD=3	5608	262455.4	17.08 (17.03 to 17.12)
			IMD=4	4869	229648.4	17.02 (16.98 to 17.07)
			IMD=5	4667	218909.3	16.93 (16.88 to 16.97)
			IMD=6	4823	215061.6	17.63 (17.58 to 17.68)
			IMD=7	4620	208001.7	17.48 (17.44 to 17.53)
			IMD=8	3963	169644.3	18.42 (18.37 to 18.46)
			IMD=9	3930	174998.8	17.82 (17.78 to 17.87)
			Most Deprivation	3619	155197.9	18.49 (18.45 to 18.54)
		2005	Least Deprivation	5252	296925.4	14.74 (14.70 to 14.78)
			IMD=2	5510	278304.1	16.07 (16.02 to 16.11)
			IMD=3	5567	265968	16.83 (16.78 to 16.87)

				IMD=4	4611	232661.3	15.96 (15.92 to 16.00)
				IMD=5	4802	221817.6	17.33 (17.28 to 17.37)
				IMD=6	4698	216872.4	17.20 (17.16 to 17.25)
				IMD=7	4564	210994.6	17.17 (17.12 to 17.21)
				IMD=8	3844	171099.6	17.93 (17.89 to 17.98)
				IMD=9	3949	176756.4	17.92 (17.87 to 17.97)
				Most Deprivation	3627	156477.4	18.61 (18.56 to 18.65)
			2006	Least Deprivation	5341	304738.7	14.60 (14.56 to 14.65)
				IMD=2	5222	285124.7	14.86 (14.82 to 14.90)
				IMD=3	5244	271794.2	15.56 (15.52 to 15.61)
				IMD=4	4505	237847.3	15.35 (15.31 to 15.40)
				IMD=5	4405	227189.5	15.60 (15.55 to 15.64)
				IMD=6	4499	220634.4	16.27 (16.22 to 16.31)
				IMD=7	4341	214501.6	16.20 (16.16 to 16.25)
				IMD=8	3476	173208.6	16.18 (16.13 to 16.22)
				IMD=9	3844	179325.1	17.34 (17.29 to 17.38)
				Most Deprivation	3332	158907.7	17.01 (16.96 to 17.06)
			2007	Least Deprivation	5241	311660.8	14.02 (13.98 to 14.06)
				IMD=2	5404	291325.6	15.05 (15.01 to 15.09)
				IMD=3	5369	276750.7	15.65 (15.61 to 15.69)
				IMD=4	4618	242565.5	15.39 (15.35 to 15.44)
				IMD=5	4559	231832.2	15.88 (15.83 to 15.92)
				IMD=6	4622	224836.5	16.48 (16.44 to 16.53)
				IMD=7	4387	218308.5	16.18 (16.13 to 16.22)
				IMD=8	3749	176352.1	17.25 (17.20 to 17.29)
				IMD=9	3821	182249.1	17.14 (17.09 to 17.18)
				Most Deprivation	3438	161475.3	17.44 (17.39 to 17.48)
			2008	Least Deprivation	5617	320623.8	14.53 (14.49 to 14.58)
				IMD=2	5603	299089.5	15.19 (15.14 to 15.23)
				IMD=3	5609	284359.9	15.94 (15.89 to 15.98)
				IMD=4	4794	248626.3	15.65 (15.61 to 15.69)
				IMD=5	4778	237693.1	16.27 (16.22 to 16.31)
				IMD=6	4863	230027.1	17.00 (16.95 to 17.04)
				IMD=7	4557	224185	16.41 (16.36 to 16.45)
				IMD=8	3822	180800.1	17.31 (17.26 to 17.36)
				IMD=9	4008	186298	17.70 (17.65 to 17.75)
				Most Deprivation	3582	164789.9	18.00 (17.95 to 18.04)
			2009	Least Deprivation	5756	328323.1	14.48 (14.44 to 14.52)
				IMD=2	5857	305450.5	15.51 (15.47 to 15.56)
				IMD=3	5923	291376.4	16.36 (16.32 to 16.41)
				IMD=4	5009	253868.3	15.99 (15.94 to 16.03)
				IMD=5	4874	242883.5	16.27 (16.23 to 16.32)
				IMD=6	4973	234120.8	17.16 (17.11 to 17.20)
				IMD=7	4589	228483.4	16.28 (16.24 to 16.33)
				IMD=8	3961	184198.1	17.77 (17.72 to 17.81)

				IMD=9	4208	189656.2	18.51 (18.46 to 18.55)
				Most Deprivation	3634	166598.9	18.04 (18.00 to 18.09)
			2010	Least Deprivation	5851	337299.4	14.34 (14.29 to 14.38)
				IMD=2	6081	313439.5	15.71 (15.66 to 15.75)
				IMD=3	5846	298358.2	15.83 (15.79 to 15.88)
				IMD=4	5072	260057.1	15.86 (15.82 to 15.91)
				IMD=5	4919	248936.7	16.06 (16.02 to 16.11)
				IMD=6	4903	239556.9	16.58 (16.53 to 16.62)
				IMD=7	4815	233852.7	16.81 (16.76 to 16.85)
				IMD=8	3935	188655.2	17.31 (17.27 to 17.36)
				IMD=9	4178	193576.5	18.14 (18.10 to 18.19)
				Most Deprivation	3719	169368.8	18.40 (18.35 to 18.45)
			2011	Least Deprivation	6042	345013.5	14.41 (14.37 to 14.45)
				IMD=2	5961	319834.4	14.99 (14.95 to 15.04)
				IMD=3	5906	304577.8	15.62 (15.58 to 15.67)
				IMD=4	5184	266107.4	15.84 (15.79 to 15.88)
				IMD=5	4923	254483.6	15.72 (15.68 to 15.77)
				IMD=6	4933	244411.2	16.37 (16.32 to 16.41)
				IMD=7	4724	238770.6	16.19 (16.14 to 16.23)
				IMD=8	4058	192275	17.58 (17.53 to 17.63)
				IMD=9	4219	197380.1	18.15 (18.10 to 18.20)
				Most Deprivation	3723	171683.9	18.37 (18.33 to 18.42)
			2012	Least Deprivation	6074	352210.3	14.08 (14.04 to 14.12)
				IMD=2	5986	326024.2	14.71 (14.67 to 14.75)
				IMD=3	6022	309780.1	15.57 (15.53 to 15.62)
				IMD=4	5177	271340.3	15.42 (15.37 to 15.46)
				IMD=5	5007	259219.7	15.63 (15.58 to 15.67)
				IMD=6	5013	248749.6	16.31 (16.27 to 16.36)
				IMD=7	4942	243207.2	16.60 (16.55 to 16.64)
				IMD=8	4128	196142.4	17.61 (17.56 to 17.66)
				IMD=9	4338	201017.5	18.22 (18.18 to 18.27)
				Most Deprivation	3603	174713.3	17.43 (17.39 to 17.48)
			2013	Least Deprivation	6402	355962.8	14.61 (14.57 to 14.66)
				IMD=2	6409	329205	15.54 (15.50 to 15.59)
				IMD=3	6452	312425.3	16.50 (16.46 to 16.55)
				IMD=4	5466	273324.9	16.11 (16.07 to 16.16)
				IMD=5	5330	261442.2	16.47 (16.43 to 16.52)
				IMD=6	5237	249988.2	16.95 (16.90 to 16.99)
				IMD=7	5127	244001.3	17.18 (17.13 to 17.23)
				IMD=8	4381	197521.7	18.49 (18.45 to 18.54)
				IMD=9	4595	201769.9	19.24 (19.19 to 19.28)
				Most Deprivation	3999	174873.4	19.40 (19.35 to 19.45)
			2014	Least Deprivation	6824	360115.8	15.33 (15.28 to 15.37)
				IMD=2	6722	332401.2	16.05 (16.01 to 16.10)
				IMD=3	6627	315476.5	16.72 (16.68 to 16.77)

			IMD=4	5781	275943.3	16.84 (16.80 to 16.89)
			IMD=5	5634	264112.4	17.20 (17.15 to 17.24)
			IMD=6	5547	252688.9	17.75 (17.70 to 17.80)
			IMD=7	5342	246484	17.71 (17.66 to 17.76)
			IMD=8	4494	200043.7	18.75 (18.70 to 18.79)
			IMD=9	4867	204139	20.24 (20.19 to 20.29)
			Most Deprivation	4265	176106	20.65 (20.60 to 20.70)
		2015	Least Deprivation	7239	364970.8	16.01 (15.97 to 16.06)
			IMD=2	7174	335916.5	16.93 (16.89 to 16.98)
			IMD=3	6949	319027.3	17.31 (17.26 to 17.35)
			IMD=4	6124	279227.9	17.63 (17.58 to 17.67)
			IMD=5	5852	267254.6	17.62 (17.58 to 17.67)
			IMD=6	5795	255416.3	18.34 (18.30 to 18.39)
			IMD=7	5667	249940.1	18.55 (18.50 to 18.59)
			IMD=8	4671	203769.6	19.24 (19.19 to 19.29)
			IMD=9	4994	207888.1	20.46 (20.41 to 20.51)
			Most Deprivation	4400	178899.1	21.00 (20.95 to 21.05)
		2016	Least Deprivation	7485	370902.4	16.22 (16.17 to 16.26)
			IMD=2	7471	341064.8	17.32 (17.27 to 17.37)
			IMD=3	7210	324208.2	17.61 (17.57 to 17.66)
			IMD=4	6233	284003.1	17.62 (17.57 to 17.66)
			IMD=5	6046	272295.6	17.85 (17.80 to 17.89)
			IMD=6	6027	260077.4	18.72 (18.68 to 18.77)
			IMD=7	5715	255256.8	18.36 (18.32 to 18.41)
			IMD=8	4908	208710.3	19.88 (19.83 to 19.93)
			IMD=9	5250	212620.6	21.02 (20.97 to 21.07)
			Most Deprivation	4594	182416.3	21.59 (21.53 to 21.64)
		2017	Least Deprivation	7654	375524.6	16.32 (16.28 to 16.37)
			IMD=2	7730	345165.5	17.63 (17.59 to 17.68)
			IMD=3	7682	328303.8	18.45 (18.40 to 18.49)
			IMD=4	6310	288320.1	17.49 (17.45 to 17.54)
			IMD=5	6169	276272.4	17.89 (17.84 to 17.93)
			IMD=6	6181	263857.3	18.93 (18.89 to 18.98)
			IMD=7	5980	259233.1	18.92 (18.88 to 18.97)
			IMD=8	5066	212951	20.03 (19.98 to 20.08)
			IMD=9	5217	216927.6	20.47 (20.42 to 20.52)
			Most Deprivation	4823	185322.1	22.39 (22.34 to 22.44)
		2018	Least Deprivation	8049	382238.7	16.75 (16.70 to 16.79)
			IMD=2	7959	351392.9	17.74 (17.69 to 17.78)
			IMD=3	7817	336276.2	18.23 (18.18 to 18.27)
			IMD=4	6665	295834.5	17.92 (17.87 to 17.97)
			IMD=5	6519	283514.6	18.33 (18.28 to 18.38)
			IMD=6	6209	269118.1	18.56 (18.51 to 18.60)
			IMD=7	6191	264683.7	19.12 (19.07 to 19.17)
			IMD=8	5283	218058.3	20.54 (20.49 to 20.59)

			IMD=9	5445	222350.3	20.97 (20.92 to 21.02)
			Most Deprivation	4821	188502	21.93 (21.88 to 21.98)
		2019	Least Deprivation	8659	381614.1	17.91 (17.86 to 17.95)
			IMD=2	8297	349221	18.48 (18.43 to 18.53)
			IMD=3	7752	329082	18.36 (18.31 to 18.40)
			IMD=4	6967	289809.5	19.02 (18.97 to 19.07)
			IMD=5	6797	282344.4	19.10 (19.05 to 19.15)
			IMD=6	6466	267471.3	19.37 (19.32 to 19.42)
			IMD=7	6167	262763.4	19.14 (19.09 to 19.19)
			IMD=8	5257	215572.8	20.55 (20.50 to 20.60)
			IMD=9	5546	221421.9	21.39 (21.34 to 21.44)
			Most Deprivation	4563	182148	21.34 (21.29 to 21.39)
Prevalence	Low back pain	2004	Least Deprivation	22245	531855	39.98 (39.93 to 40.04)
			IMD=2	21584	514185	40.36 (40.30 to 40.42)
			IMD=3	21172	491040	41.58 (41.52 to 41.64)
			IMD=4	19387	445391	42.27 (42.21 to 42.33)
			IMD=5	19077	428480	43.39 (43.33 to 43.45)
			IMD=6	19411	427665	44.46 (44.40 to 44.52)
			IMD=7	19749	433818	45.05 (44.99 to 45.12)
			IMD=8	16978	371639	45.68 (45.61 to 45.74)
			IMD=9	18873	395606	48.17 (48.11 to 48.23)
			Most Deprivation	17771	352775	51.33 (51.26 to 51.40)
		2005	Least Deprivation	23523	540212	41.71 (41.65 to 41.77)
			IMD=2	22837	519277	42.32 (42.26 to 42.38)
			IMD=3	22273	498591	43.10 (43.04 to 43.16)
			IMD=4	20399	450517	44.08 (44.02 to 44.14)
			IMD=5	19972	435535	44.69 (44.62 to 44.75)
			IMD=6	20522	434292	46.34 (46.28 to 46.40)
			IMD=7	20793	442383	46.61 (46.55 to 46.67)
			IMD=8	18215	378573	48.17 (48.11 to 48.24)
			IMD=9	20272	403684	50.85 (50.78 to 50.91)
			Most Deprivation	19549	362017	55.17 (55.10 to 55.23)
		2006	Least Deprivation	24746	551738	42.91 (42.85 to 42.97)
			IMD=2	23900	528845	43.47 (43.41 to 43.53)
			IMD=3	23253	508422	44.09 (44.03 to 44.15)
			IMD=4	21111	459597	44.53 (44.47 to 44.59)
			IMD=5	20905	445526	45.77 (45.71 to 45.84)
			IMD=6	21195	444147	46.78 (46.72 to 46.85)
			IMD=7	21793	452751	47.73 (47.67 to 47.79)
			IMD=8	18995	386668	49.37 (49.30 to 49.43)
			IMD=9	21583	412315	53.15 (53.08 to 53.22)
			Most Deprivation	20622	371084	56.94 (56.87 to 57.01)
		2007	Least Deprivation	25019	561210	42.66 (42.60 to 42.72)
			IMD=2	24630	537296	43.92 (43.86 to 43.98)

				IMD=3	24120	514839	45.03 (44.96 to 45.09)
				IMD=4	22023	466034	45.91 (45.85 to 45.97)
				IMD=5	21571	453029	46.42 (46.36 to 46.48)
				IMD=6	22291	449351	48.62 (48.55 to 48.68)
				IMD=7	22417	457645	48.65 (48.59 to 48.71)
				IMD=8	19896	392067	51.01 (50.94 to 51.07)
				IMD=9	22540	420255	54.53 (54.46 to 54.60)
				Most Deprivation	22022	378305	59.66 (59.59 to 59.73)
			2008	Least Deprivation	25677	569524	43.03 (42.97 to 43.09)
				IMD=2	25111	545740	44.14 (44.08 to 44.20)
				IMD=3	24673	522758	45.40 (45.34 to 45.46)
				IMD=4	22246	472028	45.82 (45.76 to 45.88)
				IMD=5	22309	459769	47.30 (47.24 to 47.37)
				IMD=6	22843	456603	49.12 (49.05 to 49.18)
				IMD=7	23241	466452	49.52 (49.46 to 49.58)
				IMD=8	20501	400659	51.54 (51.48 to 51.61)
				IMD=9	23770	427939	56.52 (56.45 to 56.59)
				Most Deprivation	22789	385538	60.58 (60.51 to 60.65)
			2009	Least Deprivation	26057	579604	42.91 (42.85 to 42.97)
				IMD=2	25955	553097	45.03 (44.97 to 45.09)
				IMD=3	25787	532961	46.46 (46.40 to 46.53)
				IMD=4	23384	479147	47.50 (47.44 to 47.57)
				IMD=5	23056	467334	48.13 (48.07 to 48.19)
				IMD=6	23884	463576	50.52 (50.45 to 50.58)
				IMD=7	24535	474932	51.47 (51.40 to 51.53)
				IMD=8	21783	407000	53.87 (53.81 to 53.94)
				IMD=9	25138	434757	58.86 (58.79 to 58.93)
				Most Deprivation	24240	388823	63.88 (63.80 to 63.95)
			2010	Least Deprivation	26880	591700	43.17 (43.11 to 43.23)
				IMD=2	26493	564088	45.05 (44.98 to 45.11)
				IMD=3	26701	544013	47.04 (46.98 to 47.11)
				IMD=4	23730	488212	47.15 (47.09 to 47.22)
				IMD=5	23492	477375	47.96 (47.90 to 48.03)
				IMD=6	24349	473956	50.31 (50.25 to 50.38)
				IMD=7	24934	485145	51.11 (51.05 to 51.18)
				IMD=8	22349	417024	53.94 (53.87 to 54.01)
				IMD=9	26051	444160	59.69 (59.62 to 59.76)
				Most Deprivation	25248	394869	65.51 (65.43 to 65.58)
			2011	Least Deprivation	27841	601593	44.04 (43.98 to 44.10)
				IMD=2	27685	571687	46.31 (46.25 to 46.38)
				IMD=3	27576	553196	47.84 (47.78 to 47.91)
				IMD=4	24821	496069	48.50 (48.43 to 48.56)
				IMD=5	24129	485483	48.35 (48.28 to 48.41)
				IMD=6	25425	481798	51.68 (51.62 to 51.75)
				IMD=7	26159	494735	52.49 (52.43 to 52.56)

			IMD=8	23755	425602	56.31 (56.24 to 56.38)
			IMD=9	27177	452129	61.19 (61.12 to 61.27)
			Most Deprivation	26477	399704	68.00 (67.92 to 68.07)
		2012	Least Deprivation	27856	606798	43.53 (43.47 to 43.59)
			IMD=2	27540	576802	45.64 (45.58 to 45.70)
			IMD=3	27734	557020	47.69 (47.62 to 47.75)
			IMD=4	24984	500991	48.34 (48.27 to 48.40)
			IMD=5	24672	488902	49.08 (49.02 to 49.14)
			IMD=6	26197	486484	52.77 (52.71 to 52.84)
			IMD=7	27019	498384	53.76 (53.70 to 53.83)
			IMD=8	24453	431430	57.12 (57.05 to 57.19)
			IMD=9	28639	457329	63.64 (63.57 to 63.71)
			Most Deprivation	27508	404001	69.71 (69.63 to 69.78)
		2013	Least Deprivation	27706	612234	43.02 (42.96 to 43.08)
			IMD=2	27511	582853	45.17 (45.11 to 45.23)
			IMD=3	27609	561719	47.09 (47.02 to 47.15)
			IMD=4	25007	505947	47.74 (47.68 to 47.81)
			IMD=5	24656	494149	48.52 (48.46 to 48.58)
			IMD=6	26230	491298	52.39 (52.33 to 52.46)
			IMD=7	27070	505033	53.24 (53.17 to 53.30)
			IMD=8	24795	439442	56.94 (56.87 to 57.01)
			IMD=9	29101	464240	63.78 (63.70 to 63.85)
			Most Deprivation	28382	408539	71.14 (71.07 to 71.22)
		2014	Least Deprivation	27584	615664	42.50 (42.44 to 42.56)
			IMD=2	27310	585770	44.52 (44.46 to 44.58)
			IMD=3	27587	564321	46.68 (46.62 to 46.75)
			IMD=4	25019	507951	47.59 (47.53 to 47.66)
			IMD=5	25111	495593	49.13 (49.06 to 49.19)
			IMD=6	26461	492012	52.70 (52.63 to 52.77)
			IMD=7	27327	503741	53.81 (53.74 to 53.87)
			IMD=8	25230	439484	57.73 (57.66 to 57.80)
			IMD=9	28416	462450	62.18 (62.11 to 62.25)
			Most Deprivation	27815	406646	69.77 (69.69 to 69.84)
		2015	Least Deprivation	26905	621294	40.95 (40.90 to 41.01)
			IMD=2	27163	589068	43.94 (43.88 to 44.00)
			IMD=3	26605	568234	44.86 (44.80 to 44.92)
			IMD=4	24414	511015	46.13 (46.07 to 46.19)
			IMD=5	24189	500352	46.94 (46.87 to 47.00)
			IMD=6	25631	497096	50.59 (50.53 to 50.66)
			IMD=7	27273	509631	53.16 (53.09 to 53.22)
			IMD=8	25350	446872	57.07 (57.00 to 57.13)
			IMD=9	29131	471644	62.56 (62.49 to 62.64)
			Most Deprivation	27889	413407	68.71 (68.63 to 68.78)
		2016	Least Deprivation	26358	629852	39.39 (39.33 to 39.44)
			IMD=2	26306	595989	41.91 (41.85 to 41.97)

			IMD=3	26161	575057	43.52 (43.46 to 43.58)
			IMD=4	24056	518422	44.80 (44.74 to 44.86)
			IMD=5	23728	507062	45.41 (45.34 to 45.47)
			IMD=6	25247	503896	49.11 (49.05 to 49.18)
			IMD=7	26350	518757	50.47 (50.41 to 50.54)
			IMD=8	24733	456621	54.57 (54.50 to 54.63)
			IMD=9	28838	481992	60.74 (60.67 to 60.81)
			Most Deprivation	27532	421607	66.52 (66.44 to 66.59)
		2017	Least Deprivation	25561	639042	37.70 (37.64 to 37.75)
			IMD=2	25296	604537	39.86 (39.81 to 39.92)
			IMD=3	24962	584540	40.77 (40.71 to 40.82)
			IMD=4	23105	527360	42.26 (42.20 to 42.32)
			IMD=5	23103	515831	43.40 (43.34 to 43.46)
			IMD=6	24466	513996	46.61 (46.54 to 46.67)
			IMD=7	25710	530274	48.15 (48.08 to 48.21)
			IMD=8	24220	469021	52.10 (52.03 to 52.16)
			IMD=9	28205	496301	57.79 (57.72 to 57.86)
			Most Deprivation	27472	432701	64.69 (64.61 to 64.76)
		2018	Least Deprivation	24547	653055	35.35 (35.30 to 35.40)
			IMD=2	24561	620355	37.61 (37.55 to 37.66)
			IMD=3	24565	602038	38.95 (38.89 to 39.01)
			IMD=4	22751	544631	40.28 (40.22 to 40.34)
			IMD=5	22312	531366	40.71 (40.65 to 40.76)
			IMD=6	23897	528901	44.26 (44.20 to 44.32)
			IMD=7	24765	549224	44.85 (44.79 to 44.91)
			IMD=8	23719	486073	49.34 (49.28 to 49.40)
			IMD=9	27902	514693	55.13 (55.06 to 55.19)
			Most Deprivation	27053	446205	61.91 (61.84 to 61.98)
		2019	Least Deprivation	23899	662925	33.81 (33.76 to 33.86)
			IMD=2	23924	630044	35.99 (35.94 to 36.05)
			IMD=3	23038	610421	35.88 (35.83 to 35.94)
			IMD=4	22021	553054	38.37 (38.31 to 38.43)
			IMD=5	21874	541755	39.06 (39.00 to 39.11)
			IMD=6	23190	537188	42.28 (42.22 to 42.34)
			IMD=7	24411	558689	43.57 (43.51 to 43.63)
			IMD=8	22711	495082	46.47 (46.40 to 46.53)
			IMD=9	26497	522202	51.80 (51.74 to 51.87)
			Most Deprivation	25165	438378	58.61 (58.54 to 58.68)
	Osteoarthritis	2004	Least Deprivation	9504	319889	23.94 (23.88 to 23.99)
			IMD=2	10047	304978	25.74 (25.68 to 25.79)
			IMD=3	10027	290635	26.70 (26.64 to 26.76)
			IMD=4	8693	254894	26.54 (26.48 to 26.59)
			IMD=5	8794	243730	27.79 (27.73 to 27.85)
			IMD=6	8951	239771	28.40 (28.34 to 28.46)
			IMD=7	8651	232448	28.38 (28.32 to 28.44)

			IMD=8	7409	189955	29.82 (29.76 to 29.88)
			IMD=9	7364	195923	28.94 (28.88 to 29.00)
			Most Deprivation	6759	173891	30.04 (29.98 to 30.10)
		2005	Least Deprivation	9794	326692	24.19 (24.14 to 24.25)
			IMD=2	10473	309044	26.53 (26.47 to 26.59)
			IMD=3	10511	296026	27.51 (27.45 to 27.57)
			IMD=4	8869	259220	26.65 (26.59 to 26.70)
			IMD=5	9323	248026	29.04 (28.98 to 29.10)
			IMD=6	9168	243545	28.78 (28.73 to 28.84)
			IMD=7	9147	237080	29.55 (29.49 to 29.61)
			IMD=8	7716	193028	30.77 (30.71 to 30.83)
			IMD=9	7898	199649	30.75 (30.69 to 30.81)
			Most Deprivation	7375	177184	32.44 (32.37 to 32.50)
		2006	Least Deprivation	9988	335959	24.02 (23.97 to 24.07)
			IMD=2	10419	317058	25.76 (25.70 to 25.81)
			IMD=3	10298	303650	26.33 (26.27 to 26.39)
			IMD=4	8867	265776	26.09 (26.03 to 26.15)
			IMD=5	8986	254849	27.34 (27.28 to 27.40)
			IMD=6	9150	249335	28.20 (28.14 to 28.26)
			IMD=7	9247	243079	29.33 (29.27 to 29.39)
			IMD=8	7531	196847	29.67 (29.61 to 29.73)
			IMD=9	7840	203364	30.18 (30.12 to 30.24)
			Most Deprivation	7253	180381	31.60 (31.54 to 31.66)
		2007	Least Deprivation	10079	344053	23.64 (23.59 to 23.69)
			IMD=2	10620	324401	25.64 (25.58 to 25.70)
			IMD=3	10727	309619	26.89 (26.83 to 26.95)
			IMD=4	9078	271304	26.18 (26.12 to 26.23)
			IMD=5	9351	260620	27.89 (27.83 to 27.95)
			IMD=6	9466	253554	28.76 (28.70 to 28.82)
			IMD=7	9329	246530	29.29 (29.23 to 29.35)
			IMD=8	7768	199961	30.33 (30.27 to 30.39)
			IMD=9	8170	207131	31.09 (31.03 to 31.15)
			Most Deprivation	7429	183695	32.04 (31.98 to 32.10)
		2008	Least Deprivation	10464	352095	23.89 (23.83 to 23.94)
			IMD=2	10947	331710	25.82 (25.76 to 25.87)
			IMD=3	11064	316631	27.13 (27.08 to 27.19)
			IMD=4	9308	276688	26.35 (26.30 to 26.41)
			IMD=5	9638	265850	28.30 (28.25 to 28.36)
			IMD=6	9905	258644	29.63 (29.57 to 29.69)
			IMD=7	9685	252027	29.88 (29.82 to 29.94)
			IMD=8	8102	204543	31.22 (31.16 to 31.28)
			IMD=9	8398	211360	31.64 (31.58 to 31.70)
			Most Deprivation	7908	187556	33.75 (33.69 to 33.81)
		2009	Least Deprivation	10680	361577	23.66 (23.61 to 23.72)
			IMD=2	11284	339232	25.97 (25.91 to 26.03)

				IMD=3	11625	325096	27.74 (27.68 to 27.79)
				IMD=4	9599	282840	26.58 (26.52 to 26.64)
				IMD=5	9839	271769	28.30 (28.24 to 28.36)
				IMD=6	10050	263684	29.58 (29.52 to 29.64)
				IMD=7	9782	257328	29.70 (29.64 to 29.76)
				IMD=8	8349	208717	31.80 (31.74 to 31.86)
				IMD=9	8861	215524	33.06 (33.00 to 33.12)
				Most Deprivation	8125	189853	34.42 (34.36 to 34.49)
			2010	Least Deprivation	10848	371627	23.35 (23.30 to 23.41)
				IMD=2	11686	348513	26.18 (26.12 to 26.23)
				IMD=3	11730	333371	27.35 (27.29 to 27.41)
				IMD=4	9778	290177	26.45 (26.39 to 26.51)
				IMD=5	9939	278931	27.93 (27.87 to 27.99)
				IMD=6	10201	270062	29.44 (29.38 to 29.50)
				IMD=7	10166	263749	30.30 (30.24 to 30.36)
				IMD=8	8565	213821	32.07 (32.01 to 32.13)
				IMD=9	9062	220262	33.36 (33.30 to 33.43)
				Most Deprivation	8257	193260	34.70 (34.64 to 34.77)
			2011	Least Deprivation	11047	380320	23.10 (23.05 to 23.15)
				IMD=2	11665	355947	25.44 (25.39 to 25.50)
				IMD=3	11854	340593	26.95 (26.89 to 27.00)
				IMD=4	10060	297041	26.54 (26.49 to 26.60)
				IMD=5	9984	285533	27.40 (27.34 to 27.46)
				IMD=6	10295	275485	29.17 (29.11 to 29.23)
				IMD=7	10061	269541	29.42 (29.36 to 29.48)
				IMD=8	8674	218322	31.93 (31.86 to 31.99)
				IMD=9	9256	224725	33.66 (33.59 to 33.72)
				Most Deprivation	8502	196216	35.43 (35.36 to 35.49)
			2012	Least Deprivation	11024	387192	22.46 (22.40 to 22.51)
				IMD=2	11602	361690	24.73 (24.68 to 24.79)
				IMD=3	11927	345441	26.57 (26.51 to 26.62)
				IMD=4	9997	302226	25.78 (25.73 to 25.84)
				IMD=5	10045	289378	27.08 (27.03 to 27.14)
				IMD=6	10432	279701	29.06 (29.00 to 29.12)
				IMD=7	10372	273226	29.86 (29.80 to 29.92)
				IMD=8	8756	221821	31.80 (31.73 to 31.86)
				IMD=9	9363	228153	33.47 (33.41 to 33.53)
				Most Deprivation	8318	198916	34.29 (34.23 to 34.35)
			2013	Least Deprivation	11329	393196	22.59 (22.53 to 22.64)
				IMD=2	11919	367340	24.91 (24.85 to 24.96)
				IMD=3	12253	350349	26.81 (26.75 to 26.86)
				IMD=4	10282	306458	26.08 (26.02 to 26.14)
				IMD=5	10147	293760	26.90 (26.84 to 26.95)
				IMD=6	10369	283194	28.54 (28.48 to 28.60)
				IMD=7	10488	277616	29.74 (29.68 to 29.80)

			IMD=8	9008	226117	32.14 (32.08 to 32.21)
			IMD=9	9526	232201	33.60 (33.53 to 33.66)
			Most Deprivation	8545	201571	34.84 (34.78 to 34.91)
		2014	Least Deprivation	11704	397875	22.95 (22.90 to 23.00)
			IMD=2	12144	370811	25.07 (25.02 to 25.13)
			IMD=3	12375	353792	26.75 (26.70 to 26.81)
			IMD=4	10483	309187	26.31 (26.26 to 26.37)
			IMD=5	10323	296359	27.10 (27.04 to 27.16)
			IMD=6	10652	285315	29.07 (29.01 to 29.13)
			IMD=7	10541	278648	29.81 (29.75 to 29.87)
			IMD=8	8975	227632	31.79 (31.73 to 31.85)
			IMD=9	9725	233219	34.11 (34.05 to 34.18)
			Most Deprivation	8635	201584	35.31 (35.24 to 35.37)
		2015	Least Deprivation	12196	403232	23.51 (23.46 to 23.56)
			IMD=2	12782	374915	26.00 (25.94 to 26.05)
			IMD=3	12745	357743	27.19 (27.13 to 27.25)
			IMD=4	10902	312758	27.00 (26.94 to 27.06)
			IMD=5	10764	300275	27.85 (27.80 to 27.91)
			IMD=6	11050	288832	29.78 (29.72 to 29.84)
			IMD=7	10823	282756	30.19 (30.13 to 30.25)
			IMD=8	9261	231687	32.38 (32.32 to 32.45)
			IMD=9	10072	237740	34.85 (34.78 to 34.91)
			Most Deprivation	8897	205248	35.73 (35.66 to 35.79)
		2016	Least Deprivation	12620	410158	23.84 (23.78 to 23.89)
			IMD=2	13042	380272	26.08 (26.03 to 26.14)
			IMD=3	13005	363045	27.29 (27.24 to 27.35)
			IMD=4	11172	317733	27.21 (27.15 to 27.27)
			IMD=5	10873	305238	27.64 (27.58 to 27.70)
			IMD=6	11323	293323	30.10 (30.04 to 30.16)
			IMD=7	10971	287909	30.12 (30.06 to 30.18)
			IMD=8	9519	236435	32.73 (32.67 to 32.79)
			IMD=9	10540	242279	35.81 (35.74 to 35.87)
			Most Deprivation	9263	208672	36.64 (36.57 to 36.70)
		2017	Least Deprivation	12981	416491	24.03 (23.98 to 24.09)
			IMD=2	13329	386349	26.18 (26.12 to 26.24)
			IMD=3	13558	369066	27.91 (27.85 to 27.96)
			IMD=4	11233	323533	26.79 (26.73 to 26.84)
			IMD=5	11136	310998	27.69 (27.63 to 27.75)
			IMD=6	11451	298855	29.85 (29.79 to 29.91)
			IMD=7	11271	293462	30.35 (30.29 to 30.41)
			IMD=8	9697	242023	32.60 (32.53 to 32.66)
			IMD=9	10528	248320	34.96 (34.89 to 35.02)
			Most Deprivation	9687	212938	37.68 (37.62 to 37.75)
		2018	Least Deprivation	13331	425300	24.01 (23.95 to 24.06)
			IMD=2	13765	395703	26.22 (26.16 to 26.27)

			IMD=3	13917	379848	27.64 (27.58 to 27.70)
			IMD=4	11647	333420	26.82 (26.76 to 26.87)
			IMD=5	11435	319687	27.55 (27.49 to 27.61)
			IMD=6	11639	306807	29.47 (29.41 to 29.53)
			IMD=7	11480	301798	30.01 (29.95 to 30.07)
			IMD=8	10001	249244	32.68 (32.62 to 32.74)
			IMD=9	10676	255978	34.39 (34.32 to 34.45)
			Most Deprivation	9711	218105	36.86 (36.79 to 36.92)
		2019	Least Deprivation	14265	431098	25.15 (25.09 to 25.20)
			IMD=2	14375	400774	26.85 (26.79 to 26.90)
			IMD=3	13903	384144	27.14 (27.09 to 27.20)
			IMD=4	12116	336671	27.46 (27.41 to 27.52)
			IMD=5	11856	324774	27.97 (27.91 to 28.03)
			IMD=6	11812	309663	29.55 (29.49 to 29.61)
			IMD=7	11415	304090	29.54 (29.48 to 29.60)
			IMD=8	9882	251645	31.92 (31.86 to 31.99)
			IMD=9	10707	257211	34.32 (34.25 to 34.38)
			Most Deprivation	9356	213456	36.09 (36.02 to 36.15)

Supplementary Table S2. Slope index of inequality (SII) and relative index of inequality (RII) for standardised incidence and prevalence of low back pain and osteoarthritis by overall and sex between 2004-2019

SII indicates slope index of inequality; RII indicates relative index of inequality; CI indicates confidence interval.

	SII for incidence (95% CI), per 1,000 person years			RII for incidence (95% CI)		
	Overall	Men	Women	Overall	Men	Women
	Low back pain					
2004	6.01 (5.95-6.06)	2.26 (2.19-2.33)	9.59 (9.51-9.67)	1.179 (1.177-1.180)	1.079 (1.076-1.081)	1.250 (1.248-1.252)
2005	7.01 (6.96-7.06)	3.74 (3.67-3.81)	10.12 (10.04-10.20)	1.204 (1.203-1.206)	1.128 (1.126-1.131)	1.257 (1.255-1.259)
2006	7.05 (7.00-7.10)	3.61 (3.54-3.68)	10.31 (10.23-10.40)	1.204 (1.203-1.206)	1.123 (1.121-1.126)	1.261 (1.259-1.263)
2007	8.23 (8.18-8.29)	4.82 (4.75-4.89)	11.46 (11.38-11.54)	1.235 (1.234-1.237)	1.162 (1.159-1.164)	1.287 (1.285-1.289)
2008	8.32 (8.26-8.37)	4.68 (4.61-4.75)	11.76 (11.67-11.84)	1.239 (1.237-1.240)	1.160 (1.158-1.162)	1.292 (1.290-1.294)
2009	10.37 (10.31-10.42)	6.28 (6.21-6.35)	14.24 (14.16-14.33)	1.288 (1.287-1.290)	1.208 (1.205-1.210)	1.343 (1.341-1.345)
2010	10.16 (10.11-10.22)	6.19 (6.12-6.26)	13.93 (13.85-14.02)	1.285 (1.283-1.286)	1.205 (1.203-1.207)	1.340 (1.338-1.342)
2011	11.00 (10.94-11.05)	6.74 (6.66-6.81)	15.01 (14.93-15.10)	1.300 (1.298-1.301)	1.219 (1.216-1.221)	1.354 (1.352-1.356)
2012	12.70 (12.64-12.76)	8.16 (8.08-8.23)	16.98 (16.90-17.07)	1.345(1.344-1.347)	1.265 (1.262-1.267)	1.399 (1.397-1.401)
2013	13.75 (13.70-13.81)	9.39 (9.31-9.46)	17.86 (17.78-17.95)	1.371 (1.369-1.372)	1.302 (1.300-1.305)	1.416 (1.414-1.418)
2014	13.37 (13.31-13.42)	8.77 (8.70-8.84)	17.72 (17.64-17.80)	1.360 (1.359-1.362)	1.282 (1.279-1.284)	1.414 (1.412-1.416)
2015	14.28 (14.23-14.34)	9.98 (9.91-10.05)	18.36 (18.27-18.44)	1.394 (1.392-1.395)	1.328 (1.326-1.331)	1.437 (1.435-1.439)
2016	13.66 (13.60-13.71)	9.41 (9.34-9.48)	17.67 (17.59-17.75)	1.391 (1.390-1.393)	1.322 (1.320-1.324)	1.437 (1.435-1.439)
2017	13.58 (13.53-13.63)	9.04 (8.97-9.11)	17.87 (17.79-17.95)	1.402 (1.401-1.404)	1.319 (1.316-1.321)	1.459 (1.457-1.461)
2018	13.38 (13.33-13.43)	8.41 (8.34-8.48)	18.08 (18.01-18.16)	1.416 (1.414-1.417)	1.312 (1.310-1.315)	1.485 (1.483-1.488)
2019	12.88 (12.82-12.93)	8.21 (8.14-8.28)	17.28 (17.20-17.36)	1.410 (1.409-1.412)	1.312 (1.310-1.315)	1.476 (1.474-1.478)
	Osteoarthritis					
2004	2.69 (2.64-2.74)	0.66 (0.61-0.72)	4.55 (4.48-4.62)	1.155 (1.153-1.158)	1.051 (1.047-1.056)	1.212 (1.209-1.216)
2005	3.49 (3.45-3.54)	1.38 (1.32-1.44)	5.44 (5.37-5.51)	1.205 (1.202-1.208)	1.107 (1.103-1.111)	1.261 (1.257-1.264)
2006	2.71 (2.67-2.75)	0.47 (0.42-0.53)	4.78 (4.71-4.85)	1.170 (1.167-1.173)	1.039 (1.034-1.043)	1.245 (1.242-1.249)
2007	3.45 (3.41-3.50)	1.82 (1.77-1.88)	4.96 (4.89-5.03)	1.214 (1.212-1.217)	1.148 (1.143-1.152)	1.253 (1.250-1.257)
2008	3.53 (3.49-3.57)	1.83 (1.77-1.88)	5.10 (5.03-5.17)	1.215 (1.212-1.217)	1.143 (1.138-1.147)	1.257 (1.253-1.260)
2009	3.72 (3.68-3.77)	1.84 (1.78-1.89)	5.46 (5.39-5.53)	1.223 (1.220-1.226)	1.141 (1.137-1.145)	1.271 (1.268-1.275)
2010	3.79 (3.74-3.83)	2.05 (1.99-2.10)	5.39 (5.32-5.46)	1.229 (1.226-1.232)	1.158 (1.154-1.163)	1.270 (1.267-1.274)
2011	3.97 (3.93-4.02)	1.00 (0.94-1.05)	6.71 (6.64-6.78)	1.243 (1.240-1.246)	1.079 (1.075-1.084)	1.338 (1.335-1.341)
2012	4.16 (4.11-4.20)	2.30 (2.25-2.36)	5.87 (5.80-5.93)	1.257 (1.254-1.259)	1.183 (1.179-1.188)	1.299 (1.296-1.303)
2013	4.78 (4.73-4.82)	2.39 (2.33-2.45)	6.96 (6.90-7.03)	1.279 (1.277-1.282)	1.182 (1.178-1.187)	1.334 (1.331-1.338)
2014	5.14 (5.10-5.19)	2.13 (2.07-2.19)	7.90 (7.83-7.97)	1.289 (1.287-1.292)	1.155 (1.151-1.159)	1.367 (1.364-1.370)
2015	4.73 (4.68-4.78)	2.07 (2.01-2.12)	7.16 (7.09-7.23)	1.258 (1.255-1.260)	1.147 (1.143-1.151)	1.321 (1.317-1.324)
2016	4.98 (4.93-5.02)	1.94 (1.88-2.00)	7.73 (7.66-7.81)	1.265 (1.264-1.269)	1.136 (1.132-1.140)	1.340 (1.337-1.343)
2017	4.88 (4.84-4.93)	2.11 (2.05-2.17)	7.39 (7.32-7.46)	1.258 (1.256-1.261)	1.145 (1.141-1.149)	1.322 (1.319-1.326)
2018	4.72 (4.67-4.77)	1.70 (1.64-1.76)	7.47 (7.40-7.54)	1.248 (1.245-1.251)	1.116 (1.112-1.120)	1.323 (1.320-1.326)
2019	3.53 (3.48-3.58)	0.69 (0.63-0.75)	6.11 (6.03-6.18)	1.181 (1.178-1.183)	1.046 (1.042-1.051)	1.257 (1.254-1.260)
	SII for prevalence (95% CI), per 1,000 person years			RII for prevalence (95% CI)		
	Overall	Men	Women	Overall	Men	Women
	Low back pain					
2004	10.92 (10.86-10.98)	6.07 (5.99-6.15)	15.54 (15.45-15.63)	1.246 (1.245-1.248)	1.161 (1.159-1.163)	1.306 (1.305-1.308)
2005	12.43 (12.37-12.49)	7.70 (7.62-7.78)	16.95 (16.85-17.04)	1.268 (1.267-1.269)	1.197 (1.195-1.199)	1.317 (1.316-1.319)
2006	13.22 (13.16-13.29)	8.11 (8.02-8.19)	18.10 (18.01-18.20)	1.278 (1.277-1.279)	1.203 (1.201-1.205)	1.330 (1.329-1.332)
2007	15.47 (15.4-15.53)	10.38 (10.3-10.46)	20.31 (20.21-20.40)	1.317 (1.316-1.319)	1.253 (1.251-1.255)	1.361 (1.359-1.363)
2008	16.7 (16.64-16.77)	11.13 (11.05-11.21)	22.02 (21.92-22.12)	1.338 (1.337-1.339)	1.272 (1.270-1.274)	1.383 (1.381-1.384)
2009	19.6 (19.53-19.66)	12.89 (12.80-12.97)	26.00 (25.90-26.10)	1.384 (1.383-1.386)	1.305 (1.303-1.307)	1.438 (1.436-1.439)
2010	20.27 (20.21-20.34)	13.64 (13.56-13.73)	26.60 (26.50-26.70)	1.396 (1.394-1.397)	1.320 (1.318-1.322)	1.447 (1.445-1.449)
2011	21.65 (21.58-21.71)	14.48 (14.40-14.57)	28.47 (28.37-28.57)	1.411 (1.410-1.413)	1.332 (1.330-1.334)	1.465 (1.463-1.466)
2012	24.8 (24.73-24.87)	17.34 (17.25-17.43)	31.90 (31.80-32.00)	1.465 (1.464-1.466)	1.392 (1.390-1.394)	1.514 (1.513-1.516)
2013	26.08 (26.01-26.14)	18.54 (18.45-18.63)	33.25 (33.15-33.35)	1.491 (1.490-1.492)	1.423 (1.421-1.425)	1.536 (1.535-1.538)
2014	25.91 (25.85-25.98)	18.39 (18.30-18.48)	33.09 (32.99-33.19)	1.490 (1.489-1.491)	1.420 (1.418-1.422)	1.538 (1.536-1.539)
2015	27.04 (26.98-27.11)	19.48 (19.39-19.56)	34.26 (34.16-34.36)	1.523 (1.522-1.525)	1.457 (1.455-1.458)	1.568 (1.566-1.569)
2016	26.28 (26.22-26.35)	18.81 (18.72-18.89)	33.41 (33.32-33.51)	1.527 (1.526-1.529)	1.458 (1.456-1.460)	1.574 (1.572-1.575)
2017	25.86 (25.79-25.92)	18.10 (18.02-18.18)	33.25 (33.16-33.35)	1.544 (1.543-1.546)	1.460 (1.458-1.462)	1.601 (1.599-1.602)
2018	24.97 (24.91-25.03)	16.69 (16.61-16.77)	32.87 (32.78-32.96)	1.555 (1.553-1.556)	1.451 (1.449-1.453)	1.624 (1.622-1.625)
2019	23.39 (23.33-23.45)	16.13 (16.05-16.21)	30.32 (30.23-30.41)	1.547 (1.546-1.549)	1.457 (1.455-1.459)	1.607 (1.606-1.609)
	Osteoarthritis					
2004	6.19 (6.14-6.25)	2.76 (2.69-2.83)	9.37 (9.28-9.46)	1.223 (1.221-1.226)	1.135 (1.131-1.138)	1.272 (1.269-1.275)
2005	7.75 (7.69-7.81)	3.36 (3.29-3.43)	11.82 (11.73-11.91)	1.270 (1.268-1.272)	1.158 (1.155-1.162)	1.331 (1.328-1.333)

2006	7.52 (7.46-7.57)	2.91 (2.84-2.99)	11.78 (11.69-11.87)	1.269 (1.267-1.271)	1.141 (1.138-1.145)	1.339 (1.337-1.342)
2007	8.48 (8.42-8.54)	4.53 (4.46-4.60)	12.15 (12.06-12.24)	1.300 (1.298-1.302)	1.216 (1.212-1.219)	1.347 (1.344-1.349)
2008	9.6 (9.54-9.66)	5.56 (5.49-5.64)	13.34 (13.25-13.43)	1.332 (1.330-1.334)	1.256 (1.253-1.260)	1.375 (1.372-1.377)
2009	10.41 (10.35-10.47)	5.98 (5.91-6.05)	14.52 (14.43-14.61)	1.356 (1.354-1.359)	1.272 (1.268-1.275)	1.404 (1.402-1.407)
2010	11.06 (11-11.12)	6.53 (6.46-6.60)	15.26 (15.17-15.36)	1.378 (1.376-1.380)	1.298 (1.294-1.301)	1.424 (1.421-1.426)
2011	11.73 (11.67-11.78)	5.74 (5.66-5.81)	17.26 (17.17-17.36)	1.404 (1.402-1.406)	1.265 (1.261-1.268)	1.481 (1.479-1.484)
2012	12.21 (12.15-12.27)	7.29 (7.22-7.37)	16.75 (16.66-16.84)	1.426 (1.424-1.428)	1.340 (1.337-1.343)	1.474 (1.471-1.476)
2013	12.2 (12.14-12.26)	6.91 (6.84-6.99)	17.08 (16.98-17.17)	1.425 (1.423-1.427)	1.323 (1.320-1.327)	1.480 (1.477-1.482)
2014	12.23 (12.17-12.29)	6.34 (6.27-6.42)	17.67 (17.58-17.76)	1.422 (1.420-1.424)	1.292 (1.289-1.296)	1.495 (1.492-1.497)
2015	12.1 (12.04-12.16)	6.33 (6.25-6.40)	17.43 (17.34-17.52)	1.409 (1.407-1.411)	1.287 (1.284-1.291)	1.476 (1.473-1.478)
2016	12.63 (12.57-12.69)	6.37 (6.30-6.45)	18.40 (18.30-18.49)	1.423 (1.421-1.425)	1.287 (1.283-1.290)	1.497 (1.495-1.500)
2017	12.4 (12.34-12.46)	5.96 (5.88-6.03)	18.33 (18.23-18.42)	1.415 (1.413-1.417)	1.268 (1.265-1.271)	1.494 (1.492-1.496)
2018	11.8 (11.74-11.86)	5.53 (5.45-5.60)	17.57 (17.48-17.67)	1.398 (1.396-1.400)	1.250 (1.247-1.254)	1.478 (1.476-1.481)
2019	10.08 (10.02-10.14)	4.05 (3.97-4.12)	15.63 (15.54-15.73)	1.340 (1.338-1.342)	1.183 (1.18-1.187)	1.425 (1.422-1.427)

Supplementary Table S3. Standardised incidence and prevalence of low back pain and osteoarthritis by index of multiple derivation in men and women between 2004-2019

For low back pain: the standard population was the population of England 2019 (ONS code: E92000001) aged 15 years and over; for osteoarthritis: the standard population was the population of England 2019 (ONS code: E92000001) aged 45 years and over.
IMD indicates index of multiple deprivation STDIR indicates standardised incidence rates; STDPR indicates standardised prevalence.

Year	IMD decile	Incidence						Prevalence					
		Men			Women			Men			Women		
		Numerator	Denominator	STDIR (95 % CI)	Numerator	Denominator	STDIR (95 % CI)	Numerator	Denominator	STDIR (95 % CI)	Numerator	Denominator	STDIR (95 % CI)
**Low back pain**													
2004	Least Deprivation	6668	235172.2	27.37 (27.30 to 27.43)	8064	228498.7	34.02 (33.95 to 34.09)	9753	266478	34.87 (34.79 to 34.94)	12492	265377	44.91 (44.82 to 44.99)
	IMD=2	6434	226846.5	27.58 (27.51 to 27.65)	8018	220514	35.05 (34.97 to 35.13)	9410	257439	35.20 (35.13 to 35.28)	12174	256746	45.32 (45.24 to 45.41)
	IMD=3	6428	217100.2	28.81 (28.74 to 28.88)	7827	211253.5	35.93 (35.85 to 36.00)	9312	245604	36.54 (36.46 to 36.61)	11860	245436	46.44 (46.35 to 46.52)
	IMD=4	5852	197259.5	29.08 (29.01 to 29.15)	7133	189067.2	36.71 (36.64 to 36.79)	8584	224657	37.07 (36.99 to 37.15)	10803	220734	47.27 (47.19 to 47.36)
	IMD=5	5501	189211.8	28.58 (28.51 to 28.65)	6967	182496.2	37.31 (37.23 to 37.39)	8300	215050	37.56 (37.48 to 37.64)	10777	213430	48.99 (48.90 to 49.08)
	IMD=6	5488	188958	28.61 (28.54 to 28.68)	7156	181631.1	38.69 (38.61 to 38.77)	8293	214490	37.82 (37.74 to 37.90)	11118	213175	50.85 (50.76 to 50.94)
	IMD=7	5633	192203.7	29.11 (29.04 to 29.18)	7122	182942.1	38.56 (38.49 to 38.64)	8490	218578	38.28 (38.20 to 38.36)	11259	215240	51.57 (51.48 to 51.66)
	IMD=8	4617	165259	28.06 (27.99 to 28.13)	6277	156442.3	40.11 (40.03 to 40.20)	7107	187690	37.67 (37.59 to 37.75)	9871	183949	53.38 (53.29 to 53.48)
	IMD=9	5129	176957.7	29.30 (29.23 to 29.37)	6864	165171.5	41.91 (41.83 to 42.00)	7919	200883	39.46 (39.37 to 39.54)	10954	194723	56.55 (56.46 to 56.65)
	Most Deprivation	4762	158553.5	30.51 (30.44 to 30.59)	6297	146843.6	43.67 (43.58 to 43.75)	7462	179932	41.79 (41.70 to 41.87)	10309	172843	60.51 (60.41 to 60.61)
2005	Least Deprivation	6725	236933.4	27.50 (27.43 to 27.57)	8440	229999.6	35.55 (35.47 to 35.62)	10185	270550	35.99 (35.91 to 36.06)	13338	269662	47.22 (47.13 to 47.30)
	IMD=2	6602	227598.8	28.14 (28.07 to 28.21)	8147	220702.7	35.88 (35.80 to 35.95)	9957	260240	36.81 (36.73 to 36.89)	12880	259037	47.62 (47.53 to 47.71)
	IMD=3	6379	218598.2	28.34 (28.27 to 28.40)	8027	212117.9	36.64 (36.56 to 36.71)	9573	249525	36.93 (36.85 to 37.01)	12700	249066	49.04 (48.95 to 49.13)
	IMD=4	5829	197780.7	28.81 (28.74 to 28.88)	7293	189252.5	37.73 (37.65 to 37.81)	8871	226948	37.86 (37.78 to 37.94)	11528	223569	50.07 (49.98 to 50.16)
	IMD=5	5547	191189.7	28.46 (28.39 to 28.53)	6978	183202.7	37.25 (37.18 to 37.33)	8648	218795	38.43 (38.35 to 38.51)	11324	216740	50.70 (50.61 to 50.79)
	IMD=6	5603	190209.1	29.17 (29.10 to 29.24)	7274	181465.7	39.50 (39.42 to 39.58)	8729	218362	39.15 (39.07 to 39.23)	11793	215930	53.26 (53.17 to 53.35)
	IMD=7	5668	194380.2	29.11 (29.04 to 29.18)	7239	183445	39.13 (39.05 to 39.21)	8839	223449	39.14 (39.06 to 39.22)	11954	218934	53.80 (53.70 to 53.89)
	IMD=8	4838	166937.7	29.17 (29.10 to 29.24)	6429	156371.7	41.03 (40.94 to 41.11)	7663	191740	39.90 (39.82 to 39.98)	10552	186833	56.13 (56.04 to 56.23)
	IMD=9	5292	178297.5	30.17 (30.10 to 30.24)	7089	164853.6	43.40 (43.31 to 43.48)	8432	205331	41.35 (41.26 to 41.43)	11840	198353	59.99 (59.89 to 60.09)
	Most Deprivation	5117	160241	32.51 (32.44 to 32.59)	6651	147102.9	46.22 (46.13 to 46.31)	8249	184759	45.06 (44.97 to 45.15)	11300	177258	64.89 (64.79 to 64.99)

2006	Least Deprivation	6963	240899.1	27.83 (27.76 to 27.90)	8668	233216	36.00 (35.93 to 36.08)	10732	276117	37.09 (37.01 to 37.17)	14014	275621	48.50 (48.42 to 48.59)
	IMD=2	6725	230492.1	28.26 (28.19 to 28.33)	8411	222877.2	36.58 (36.51 to 36.66)	10344	264709	37.52 (37.44 to 37.60)	13556	264136	49.19 (49.10 to 49.28)
	IMD=3	6529	221173.5	28.70 (28.63 to 28.77)	7989	214117.8	36.23 (36.15 to 36.31)	10136	254356	38.26 (38.18 to 38.34)	13117	254066	49.70 (49.61 to 49.79)
	IMD=4	5858	200187.3	28.68 (28.61 to 28.75)	7280	191282	36.99 (36.91 to 37.06)	9207	231256	38.54 (38.45 to 38.62)	11904	228341	50.30 (50.21 to 50.39)
	IMD=5	5683	194338.6	28.73 (28.66 to 28.80)	7151	185232.4	37.87 (37.79 to 37.95)	8951	224059	38.84 (38.76 to 38.92)	11954	221467	52.45 (52.35 to 52.54)
	IMD=6	5598	192636.2	28.71 (28.64 to 28.78)	7288	182913.5	39.12 (39.04 to 39.20)	8922	223491	39.07 (38.99 to 39.15)	12273	220656	54.20 (54.11 to 54.30)
	IMD=7	5631	196932.8	28.56 (28.49 to 28.63)	7410	184539.7	39.82 (39.74 to 39.90)	9263	229066	40.06 (39.98 to 40.14)	12530	223685	55.11 (55.01 to 55.20)
	IMD=8	4898	168736.9	29.42 (29.35 to 29.49)	6438	156943	41.16 (41.08 to 41.24)	7954	196090	40.65 (40.56 to 40.73)	11041	190578	57.76 (57.66 to 57.86)
	IMD=9	5518	180616.8	31.15 (31.07 to 31.22)	7186	165936.3	44.00 (43.92 to 44.09)	9087	209865	43.72 (43.64 to 43.81)	12496	202450	62.22 (62.12 to 62.32)
	Most Deprivation	5143	162743.8	32.36 (32.29 to 32.44)	6758	148250.4	46.73 (46.64 to 46.81)	8614	189567	46.12 (46.03 to 46.21)	12008	181517	67.36 (67.25 to 67.46)
2007	Least Deprivation	6907	243803.6	27.30 (27.24 to 27.37)	8627	235324.7	35.61 (35.53 to 35.68)	10725	281134	36.34 (36.26 to 36.42)	14294	280076	48.75 (48.66 to 48.84)
	IMD=2	6761	233188.4	28.11 (28.04 to 28.18)	8445	224812.1	36.22 (36.14 to 36.30)	10619	269067	37.85 (37.77 to 37.93)	14011	268229	49.76 (49.67 to 49.85)
	IMD=3	6669	222695.9	29.01 (28.94 to 29.08)	8264	214784	37.37 (37.29 to 37.44)	10415	257790	38.66 (38.58 to 38.74)	13705	257049	51.15 (51.06 to 51.24)
	IMD=4	6171	201884.5	29.89 (29.82 to 29.97)	7405	192628.5	37.58 (37.50 to 37.66)	9646	234568	39.84 (39.75 to 39.92)	12377	231466	51.75 (51.66 to 51.85)
	IMD=5	5790	196548.2	28.91 (28.84 to 28.98)	7173	186634.3	37.68 (37.60 to 37.75)	9259	228090	39.43 (39.35 to 39.52)	12312	224939	53.14 (53.05 to 53.23)
	IMD=6	6023	194657.5	30.58 (30.51 to 30.65)	7451	184010.3	39.78 (39.70 to 39.86)	9465	226258	40.92 (40.83 to 41.00)	12826	223093	56.03 (55.93 to 56.13)
	IMD=7	5854	199076.8	29.37 (29.30 to 29.44)	7380	185640.5	39.57 (39.49 to 39.65)	9608	231991	41.00 (40.91 to 41.08)	12809	225654	56.01 (55.92 to 56.11)
	IMD=8	5065	171078.2	29.93 (29.86 to 30.00)	6660	157894.7	42.33 (42.25 to 42.42)	8382	199099	42.24 (42.15 to 42.32)	11514	192968	59.45 (59.35 to 59.55)
	IMD=9	5520	182575.1	30.82 (30.74 to 30.89)	7409	166987.3	44.91 (44.82 to 44.99)	9359	214116	44.29 (44.20 to 44.38)	13181	206139	64.38 (64.28 to 64.48)
	Most Deprivation	5420	164583.3	33.73 (33.65 to 33.81)	6918	149060.4	47.57 (47.49 to 47.66)	9225	193240	48.49 (48.40 to 48.58)	12797	185065	70.40 (70.29 to 70.50)
2008	Least Deprivation	6997	248907.6	27.00 (26.93 to 27.07)	8890	239478.1	35.88 (35.80 to 35.95)	10904	285461	36.22 (36.14 to 36.30)	14773	284063	49.58 (49.49 to 49.67)
	IMD=2	6938	237434.3	28.24 (28.17 to 28.31)	8516	228326.4	36.17 (36.09 to 36.25)	10882	273507	37.99 (37.91 to 38.07)	14229	272233	50.05 (49.96 to 50.14)
	IMD=3	6583	227105.2	28.04 (27.97 to 28.11)	8345	218266.8	37.21 (37.13 to 37.29)	10488	261845	38.31 (38.23 to 38.39)	14185	260913	52.22 (52.13 to 52.31)
	IMD=4	6004	205192.7	28.62 (28.55 to 28.69)	7537	195329.4	37.73 (37.65 to 37.81)	9603	237713	39.11 (39.03 to 39.20)	12643	234315	52.27 (52.18 to 52.36)
	IMD=5	5828	200193.3	28.62 (28.55 to 28.69)	7523	189626.7	38.93 (38.85 to 39.01)	9455	231570	39.65 (39.57 to 39.73)	12854	228199	54.67 (54.57 to 54.76)
	IMD=6	5820	197823.1	29.12 (29.05 to 29.19)	7582	186968.7	40.05 (39.97 to 40.13)	9489	229937	40.36 (40.28 to 40.44)	13354	226666	57.54 (57.45 to 57.64)
	IMD=7	5942	203446.5	29.18 (29.11 to 29.25)	7523	189582.8	39.42 (39.34 to 39.50)	9746	236216	40.94 (40.86 to 41.02)	13495	230236	57.78 (57.68 to 57.87)
	IMD=8	5090	174714.6	29.62 (29.55 to 29.69)	6672	160718.1	41.70 (41.62 to 41.78)	8527	203737	42.11 (42.03 to 42.20)	11974	196922	60.62 (60.52 to 60.72)
	IMD=9	5660	185824.3	31.14 (31.06 to 31.21)	7640	169471.7	45.73 (45.65 to 45.82)	9819	218048	45.64 (45.55 to 45.73)	13951	209891	66.98 (66.87 to 67.08)

	Most Deprivation	5305	166937.9	32.54 (32.47 to 32.62)	7104	150602.1	48.23 (48.14 to 48.32)	9440	197071	48.69 (48.60 to 48.78)	13349	188467	72.02 (71.92 to 72.13)
2009	Least Deprivation	7185	252332.1	27.46 (27.39 to 27.52)	8813	242962.9	35.13 (35.05 to 35.20)	11294	290354	36.97 (36.89 to 37.05)	14763	289250	48.61 (48.52 to 48.70)
	IMD=2	6948	239684.2	27.98 (27.91 to 28.05)	8881	230342.4	37.39 (37.32 to 37.47)	11046	277297	38.04 (37.96 to 38.12)	14909	275800	51.75 (51.66 to 51.84)
	IMD=3	6947	230596	29.07 (29.00 to 29.14)	8695	221392.1	38.13 (38.05 to 38.21)	11026	267078	39.30 (39.22 to 39.38)	14761	265883	53.36 (53.26 to 53.45)
	IMD=4	6247	207599.1	29.45 (29.38 to 29.52)	7910	197516.9	39.34 (39.26 to 39.42)	10097	241209	40.51 (40.42 to 40.59)	13287	237938	54.24 (54.14 to 54.33)
	IMD=5	5968	202850.8	28.97 (28.90 to 29.04)	7685	191667.3	39.33 (39.25 to 39.41)	9790	235431	40.43 (40.35 to 40.51)	13266	231903	55.54 (55.44 to 55.63)
	IMD=6	6209	200441.8	30.61 (30.53 to 30.68)	7922	189077.8	41.29 (41.20 to 41.37)	10048	233411	42.07 (41.99 to 42.16)	13836	230165	58.64 (58.55 to 58.74)
	IMD=7	6276	206296	30.53 (30.46 to 30.60)	7879	191930.2	40.88 (40.80 to 40.96)	10370	240600	42.87 (42.78 to 42.95)	14165	234332	59.74 (59.64 to 59.84)
	IMD=8	5387	176704.4	31.08 (31.00 to 31.15)	7045	162742.3	43.37 (43.29 to 43.46)	9075	206712	44.19 (44.10 to 44.27)	12708	200288	63.20 (63.09 to 63.30)
	IMD=9	5873	187972.1	32.13 (32.05 to 32.20)	8191	170903.4	48.70 (48.61 to 48.79)	10135	221264	46.48 (46.39 to 46.57)	15003	213493	70.77 (70.66 to 70.87)
	Most Deprivation	5619	167688.5	34.46 (34.38 to 34.53)	7405	150522.5	50.12 (50.03 to 50.21)	9995	198811	51.20 (51.11 to 51.30)	14245	190012	76.07 (75.96 to 76.18)
2010	Least Deprivation	7411	257076.2	27.69 (27.62 to 27.76)	9098	248454.8	35.31 (35.23 to 35.38)	11631	295906	37.13 (37.05 to 37.21)	15249	295794	48.98 (48.89 to 49.07)
	IMD=2	7139	243655.8	28.32 (28.25 to 28.39)	8889	235010	36.83 (36.76 to 36.91)	11396	282188	38.49 (38.41 to 38.58)	15097	281900	51.35 (51.26 to 51.44)
	IMD=3	7196	234494	29.67 (29.60 to 29.74)	8760	225564.8	37.75 (37.68 to 37.83)	11567	272277	40.47 (40.39 to 40.55)	15134	271736	53.37 (53.28 to 53.46)
	IMD=4	6238	210849.9	28.93 (28.86 to 29.00)	7893	200681.6	38.45 (38.37 to 38.53)	10143	245514	39.90 (39.82 to 39.98)	13587	242698	54.13 (54.04 to 54.23)
	IMD=5	5995	206408.9	28.56 (28.49 to 28.63)	7680	195479.2	38.53 (38.45 to 38.61)	9869	240145	39.92 (39.84 to 40.00)	13623	237230	55.70 (55.60 to 55.80)
	IMD=6	6185	203727.1	29.94 (29.87 to 30.01)	7921	192664.6	40.49 (40.41 to 40.57)	10288	238303	42.10 (42.02 to 42.19)	14061	235653	58.22 (58.12 to 58.31)
	IMD=7	6151	209808.5	29.48 (29.41 to 29.55)	7993	195633.8	40.62 (40.54 to 40.70)	10471	245604	42.33 (42.25 to 42.42)	14463	239541	59.56 (59.47 to 59.66)
	IMD=8	5355	180179.1	30.20 (30.13 to 30.28)	7241	166364.6	43.69 (43.61 to 43.78)	9166	211409	43.56 (43.47 to 43.65)	13183	205615	63.93 (63.82 to 64.03)
	IMD=9	6273	191095.7	33.87 (33.79 to 33.95)	7984	173717.5	46.62 (46.54 to 46.71)	10821	225807	48.71 (48.62 to 48.80)	15230	218353	70.25 (70.14 to 70.35)
	Most Deprivation	5730	169343.3	34.86 (34.79 to 34.94)	7555	151629	50.79 (50.70 to 50.88)	10453	202093	52.74 (52.64 to 52.83)	14795	192776	77.79 (77.68 to 77.91)
2011	Least Deprivation	7609	260153.9	28.01 (27.94 to 28.08)	9479	253101.7	36.24 (36.16 to 36.31)	12017	300055	37.83 (37.75 to 37.91)	15824	301538	50.02 (49.93 to 50.11)
	IMD=2	7335	245925.8	28.75 (28.68 to 28.82)	9327	238713.1	37.80 (37.72 to 37.88)	11862	285140	39.57 (39.48 to 39.65)	15823	286547	52.81 (52.71 to 52.90)
	IMD=3	7241	237297.9	29.46 (29.39 to 29.53)	9245	229040	39.31 (39.23 to 39.39)	11732	276450	40.37 (40.28 to 40.45)	15844	276746	55.04 (54.94 to 55.13)
	IMD=4	6454	213100.2	29.57 (29.50 to 29.64)	8367	204022.3	40.05 (39.97 to 40.13)	10507	248856	40.78 (40.69 to 40.86)	14314	247213	55.93 (55.83 to 56.02)
	IMD=5	6319	208444.8	29.74 (29.67 to 29.81)	7856	198575.9	38.84 (38.77 to 38.92)	10342	243639	41.15 (41.06 to 41.23)	13787	241844	55.27 (55.18 to 55.37)
	IMD=6	6368	206004.2	30.57 (30.50 to 30.65)	8298	195718.9	41.66 (41.57 to 41.74)	10643	241685	43.05 (42.97 to 43.14)	14782	240113	59.98 (59.88 to 60.08)
	IMD=7	6438	212268.1	30.41 (30.34 to 30.49)	8289	199089.9	41.32 (41.23 to 41.40)	10993	249888	43.64 (43.55 to 43.72)	15166	244847	61.01 (60.91 to 61.11)
	IMD=8	5732	182401.2	32.04 (31.97 to 32.12)	7656	169178.7	45.59 (45.50 to 45.68)	9791	215240	45.82 (45.73 to 45.91)	13964	210362	66.41 (66.30 to 66.51)

	IMD=9	6210	192853.1	33.06 (32.98 to 33.13)	8452	176319.4	48.65 (48.56 to 48.74)	11081	229485	49.07 (48.98 to 49.16)	16096	222644	72.86 (72.75 to 72.97)
	Most Deprivation	5915	170416.4	35.94 (35.86 to 36.02)	7952	152921.6	53.29 (53.19 to 53.38)	10820	204250	54.16 (54.07 to 54.26)	15657	195454	81.30 (81.19 to 81.42)
2012	Least Deprivation	7539	262453.1	27.52 (27.45 to 27.58)	9571	255918.5	35.95 (35.88 to 36.03)	11900	302188	37.21 (37.13 to 37.28)	15956	304610	49.61 (49.52 to 49.70)
	IMD=2	7301	248215.3	28.27 (28.20 to 28.34)	9217	241503.3	37.05 (36.97 to 37.13)	11876	287073	39.20 (39.12 to 39.28)	15664	289729	51.83 (51.74 to 51.93)
	IMD=3	7190	238678.6	29.04 (28.97 to 29.11)	9141	231095.3	38.38 (38.30 to 38.46)	11719	277826	40.07 (39.99 to 40.15)	16015	279194	55.01 (54.92 to 55.11)
	IMD=4	6425	215195	29.08 (29.01 to 29.15)	8310	206497	39.36 (39.28 to 39.44)	10584	250649	40.67 (40.59 to 40.75)	14400	250342	55.72 (55.62 to 55.81)
	IMD=5	6323	210165.1	29.48 (29.41 to 29.55)	8125	201127.4	39.64 (39.56 to 39.72)	10424	244642	41.28 (41.19 to 41.36)	14248	244260	56.59 (56.49 to 56.68)
	IMD=6	6525	208123.3	31.02 (30.95 to 31.09)	8428	198034.4	42.02 (41.93 to 42.10)	11047	243504	44.36 (44.27 to 44.44)	15150	242980	60.87 (60.77 to 60.97)
	IMD=7	6570	214327.7	30.61 (30.54 to 30.69)	8676	201024.1	42.89 (42.81 to 42.97)	11263	251138	44.38 (44.30 to 44.47)	15756	247246	62.79 (62.69 to 62.89)
	IMD=8	5801	185501.4	31.90 (31.83 to 31.97)	7796	171605.8	45.63 (45.54 to 45.71)	10090	217896	46.75 (46.66 to 46.84)	14363	213534	67.10 (67.00 to 67.21)
	IMD=9	6562	195340.4	34.59 (34.52 to 34.67)	8774	178309.3	49.74 (49.65 to 49.83)	11694	231683	51.30 (51.21 to 51.40)	16945	225646	75.51 (75.39 to 75.62)
	Most Deprivation	5982	172635.1	35.95 (35.87 to 36.03)	8152	154172.9	53.83 (53.74 to 53.92)	11239	206140	55.62 (55.52 to 55.72)	16269	197861	83.27 (83.15 to 83.38)
2013	Least Deprivation	7502	263529.7	27.19 (27.13 to 27.26)	9505	256751.5	35.85 (35.78 to 35.93)	11823	304863	36.57 (36.49 to 36.65)	15883	307371	49.23 (49.14 to 49.31)
	IMD=2	7255	248842	28.12 (28.05 to 28.19)	9346	242341.4	37.42 (37.34 to 37.49)	11672	290068	38.24 (38.16 to 38.32)	15839	292785	51.83 (51.74 to 51.93)
	IMD=3	7121	239133.6	28.84 (28.77 to 28.91)	9132	231097.9	38.20 (38.12 to 38.28)	11611	280062	39.51 (39.43 to 39.59)	15998	281657	54.38 (54.28 to 54.47)
	IMD=4	6529	215893.7	29.37 (29.30 to 29.44)	8366	206839	39.44 (39.36 to 39.52)	10612	253082	40.25 (40.16 to 40.33)	14395	252865	54.96 (54.86 to 55.05)
	IMD=5	6260	210433.9	29.10 (29.03 to 29.17)	8254	201364	40.25 (40.17 to 40.33)	10340	247338	40.40 (40.32 to 40.49)	14316	246811	56.33 (56.23 to 56.43)
	IMD=6	6573	207262	31.33 (31.26 to 31.41)	8443	197474	42.28 (42.20 to 42.36)	10998	246154	43.63 (43.54 to 43.71)	15232	245144	60.83 (60.73 to 60.93)
	IMD=7	6622	212908.9	31.17 (31.10 to 31.25)	8634	200239.7	42.89 (42.81 to 42.98)	11307	254713	43.98 (43.89 to 44.07)	15763	250320	62.14 (62.04 to 62.24)
	IMD=8	5853	184288.4	32.39 (32.32 to 32.47)	7745	171807.3	45.28 (45.20 to 45.37)	10232	222158	46.59 (46.50 to 46.68)	14563	217284	66.89 (66.79 to 67.00)
	IMD=9	6584	192404	35.18 (35.10 to 35.25)	8898	177892.1	50.78 (50.69 to 50.87)	11874	235170	51.29 (51.20 to 51.38)	17227	229070	75.79 (75.68 to 75.90)
	Most Deprivation	6113	171167.6	36.86 (36.78 to 36.94)	8275	152772.3	55.28 (55.18 to 55.37)	11535	208532	56.41 (56.31 to 56.51)	16847	200007	85.32 (85.20 to 85.43)
2014	Least Deprivation	7533	265074.3	27.23 (27.16 to 27.30)	9602	258328.3	35.71 (35.64 to 35.79)	11793	306324	36.35 (36.27 to 36.43)	15791	309340	48.42 (48.33 to 48.51)
	IMD=2	7448	250187.8	28.69 (28.62 to 28.76)	9319	243560	36.95 (36.87 to 37.03)	11641	291290	37.99 (37.90 to 38.07)	15669	294480	50.81 (50.72 to 50.90)
	IMD=3	7371	240679.9	29.50 (29.43 to 29.57)	9219	232029.5	38.36 (38.28 to 38.44)	11726	281532	39.61 (39.53 to 39.69)	15861	282789	53.49 (53.40 to 53.59)
	IMD=4	6499	217222.8	29.17 (29.10 to 29.24)	8257	207396.5	38.76 (38.68 to 38.84)	10614	254195	40.13 (40.05 to 40.22)	14405	253756	54.77 (54.68 to 54.86)
	IMD=5	6340	212283.4	29.14 (29.07 to 29.21)	8379	202096.7	40.56 (40.48 to 40.64)	10526	247996	40.89 (40.81 to 40.97)	14585	247597	57.05 (56.95 to 57.15)
	IMD=6	6616	209125.9	31.28 (31.21 to 31.36)	8598	197955.2	42.87 (42.79 to 42.96)	11065	246256	43.85 (43.76 to 43.94)	15396	245756	61.21 (61.11 to 61.31)
	IMD=7	6693	214795.4	31.23 (31.16 to 31.31)	8827	200419.3	43.77 (43.69 to 43.86)	11374	253485	44.43 (44.34 to 44.51)	15953	250256	62.83 (62.73 to 62.93)

	IMD=8	6125	186683.5	33.32 (33.25 to 33.40)	7947	172703.4	46.32 (46.23 to 46.41)	10535	221063	47.91 (47.82 to 48.00)	14695	218421	67.18 (67.07 to 67.28)
	IMD=9	6452	194676.8	34.02 (33.95 to 34.10)	8724	178746.6	49.36 (49.27 to 49.45)	11509	232735	50.06 (49.97 to 50.15)	16907	229715	73.84 (73.73 to 73.95)
	Most Deprivation	6164	171977.3	36.88 (36.80 to 36.96)	8099	152806.5	54.07 (53.97 to 54.16)	11360	207372	55.63 (55.53 to 55.72)	16455	199274	83.37 (83.26 to 83.49)
2015	Least Deprivation	7342	268787.8	25.98 (25.92 to 26.05)	9429	261796.4	34.77 (34.69 to 34.84)	11391	309077	34.55 (34.47 to 34.63)	15514	312217	47.12 (47.03 to 47.20)
	IMD=2	7421	252617.3	28.15 (28.08 to 28.22)	9347	245587.5	36.71 (36.63 to 36.78)	11591	292981	37.53 (37.45 to 37.61)	15572	296087	50.11 (50.02 to 50.20)
	IMD=3	7068	243676.8	27.94 (27.88 to 28.01)	8873	234356	36.67 (36.60 to 36.75)	11312	283735	37.97 (37.89 to 38.05)	15293	284499	51.48 (51.39 to 51.57)
	IMD=4	6492	220097.3	28.65 (28.58 to 28.72)	8105	209508.5	37.75 (37.67 to 37.83)	10434	256201	39.05 (38.97 to 39.13)	13980	254814	52.94 (52.85 to 53.04)
	IMD=5	6279	215041.6	28.55 (28.48 to 28.62)	7959	203934.2	38.21 (38.13 to 38.29)	10248	250773	39.44 (39.36 to 39.53)	13941	249579	54.15 (54.05 to 54.24)
	IMD=6	6349	212523.7	29.52 (29.45 to 29.60)	8519	199566.5	42.14 (42.06 to 42.23)	10572	249907	41.31 (41.22 to 41.39)	15059	247189	59.53 (59.43 to 59.62)
	IMD=7	6713	218799.8	30.77 (30.69 to 30.84)	8868	202410.9	43.55 (43.47 to 43.64)	11285	257695	43.39 (43.30 to 43.47)	15988	251936	62.55 (62.45 to 62.66)
	IMD=8	6053	191236.3	32.20 (32.12 to 32.27)	8056	175337.3	46.11 (46.02 to 46.19)	10454	226422	46.39 (46.30 to 46.47)	14896	220450	67.34 (67.24 to 67.45)
	IMD=9	6818	199736.1	35.12 (35.04 to 35.20)	8815	181983	49.08 (48.99 to 49.17)	11985	238692	50.91 (50.82 to 51.00)	17146	232952	73.78 (73.67 to 73.89)
	Most Deprivation	6217	175248.9	36.51 (36.43 to 36.59)	8112	155501.3	53.19 (53.10 to 53.29)	11427	210801	54.95 (54.85 to 55.04)	16462	202606	81.95 (81.83 to 82.06)
2016	Least Deprivation	7330	273300.3	25.37 (25.30 to 25.43)	9223	266743.5	33.09 (33.01 to 33.16)	11298	313203	33.70 (33.62 to 33.78)	15060	316649	44.86 (44.77 to 44.94)
	IMD=2	7066	256928.8	26.32 (26.25 to 26.38)	9182	249783.1	35.25 (35.18 to 35.33)	11069	296454	35.32 (35.24 to 35.39)	15237	299535	48.25 (48.16 to 48.34)
	IMD=3	6982	248094.9	27.04 (26.97 to 27.11)	8900	238813.9	36.09 (36.01 to 36.16)	11051	287077	36.57 (36.49 to 36.65)	15110	287980	50.21 (50.12 to 50.30)
	IMD=4	6418	224316.5	27.85 (27.78 to 27.92)	8096	213711.9	36.88 (36.80 to 36.96)	10179	260070	37.60 (37.52 to 37.68)	13877	258352	51.73 (51.63 to 51.82)
	IMD=5	6144	219693.3	27.24 (27.17 to 27.31)	7962	208326.7	37.42 (37.34 to 37.50)	9955	254466	37.69 (37.61 to 37.77)	13773	252596	52.83 (52.74 to 52.92)
	IMD=6	6456	217570.9	29.29 (29.22 to 29.36)	8245	203666	39.92 (39.83 to 40.00)	10658	253610	40.99 (40.91 to 41.07)	14589	250286	56.93 (56.83 to 57.02)
	IMD=7	6574	224565.4	29.35 (29.28 to 29.42)	8473	207198.9	40.71 (40.63 to 40.80)	11017	262598	41.52 (41.44 to 41.60)	15333	256159	59.08 (58.98 to 59.18)
	IMD=8	5872	197453.2	30.35 (30.28 to 30.42)	7866	179949.1	44.02 (43.94 to 44.11)	10208	231876	44.31 (44.23 to 44.40)	14525	224745	64.43 (64.33 to 64.53)
	IMD=9	6592	205964.1	33.02 (32.94 to 33.09)	8726	187212.2	47.35 (47.27 to 47.44)	11769	244266	48.93 (48.84 to 49.02)	17069	237726	72.10 (71.99 to 72.20)
	Most Deprivation	6216	180172.6	35.64 (35.56 to 35.72)	8166	159612.6	52.12 (52.03 to 52.21)	11239	215246	52.91 (52.82 to 53.01)	16293	206361	79.60 (79.49 to 79.72)
2017	Least Deprivation	7299	277699.3	24.95 (24.89 to 25.02)	9102	271986.7	31.97 (31.90 to 32.04)	11002	317488	32.45 (32.37 to 32.52)	14559	321554	42.75 (42.66 to 42.83)
	IMD=2	7007	261369.7	25.73 (25.66 to 25.80)	8854	254055.3	33.51 (33.44 to 33.58)	10783	300677	34.01 (33.93 to 34.09)	14513	303860	45.50 (45.41 to 45.58)
	IMD=3	6816	252707.3	25.89 (25.83 to 25.96)	8597	243992.4	34.07 (34.00 to 34.15)	10708	291729	34.77 (34.70 to 34.85)	14254	292811	46.53 (46.44 to 46.62)
	IMD=4	6209	228708.6	26.31 (26.24 to 26.38)	7957	218092.9	35.49 (35.42 to 35.57)	9779	264528	35.45 (35.38 to 35.53)	13326	262832	48.81 (48.72 to 48.90)
	IMD=5	6275	223692.8	27.30 (27.24 to 27.37)	7754	212338	35.75 (35.67 to 35.82)	9900	258768	36.78 (36.70 to 36.86)	13203	257063	49.76 (49.67 to 49.85)
	IMD=6	6263	222254.8	27.81 (27.74 to 27.88)	8156	208390.9	38.54 (38.47 to 38.62)	10259	258723	38.64 (38.56 to 38.72)	14207	255273	54.27 (54.18 to 54.37)

	IMD=7	6560	230543.2	28.50 (28.43 to 28.57)	8357	211872.5	39.22 (39.14 to 39.30)	10814	268925	39.78 (39.70 to 39.87)	14896	261349	56.19 (56.09 to 56.29)
	IMD=8	5955	203621.3	29.98 (29.90 to 30.05)	7820	184920.9	42.69 (42.61 to 42.77)	10019	238706	42.28 (42.20 to 42.37)	14201	230315	61.54 (61.44 to 61.64)
	IMD=9	6482	212835.2	31.44 (31.36 to 31.51)	8673	193221.4	45.87 (45.78 to 45.95)	11507	251809	46.32 (46.24 to 46.41)	16698	244492	68.82 (68.71 to 68.92)
	Most Deprivation	6257	185061.2	34.95 (34.88 to 35.03)	8168	164206	50.87 (50.77 to 50.96)	11261	221068	51.62 (51.52 to 51.71)	16211	211633	77.26 (77.15 to 77.37)
2018	Least Deprivation	7177	284590.9	23.92 (23.86 to 23.99)	8836	279317.2	30.14 (30.07 to 30.21)	10712	324338	30.92 (30.85 to 30.99)	13835	328717	39.61 (39.53 to 39.69)
	IMD=2	6797	268294.2	24.11 (24.04 to 24.17)	8765	261421.8	32.28 (32.21 to 32.36)	10281	308574	31.41 (31.34 to 31.49)	14280	311781	43.57 (43.48 to 43.65)
	IMD=3	6884	261081	25.25 (25.19 to 25.32)	8410	252530.2	32.20 (32.13 to 32.27)	10572	300599	33.29 (33.22 to 33.37)	13993	301439	44.39 (44.31 to 44.48)
	IMD=4	6253	236715.3	25.59 (25.52 to 25.65)	7809	226225.5	33.58 (33.51 to 33.65)	9702	273184	34.02 (33.94 to 34.10)	13049	271447	46.30 (46.21 to 46.39)
	IMD=5	5888	231270	24.76 (24.70 to 24.83)	7686	220199.9	34.15 (34.08 to 34.23)	9402	266565	33.86 (33.78 to 33.94)	12910	264801	47.29 (47.20 to 47.38)
	IMD=6	6331	228581.9	27.31 (27.25 to 27.38)	7982	215059	36.59 (36.51 to 36.67)	10104	266306	36.94 (36.86 to 37.01)	13793	262595	51.30 (51.21 to 51.39)
	IMD=7	6226	238894.6	26.15 (26.08 to 26.21)	8232	219690.8	37.31 (37.23 to 37.39)	10251	278776	36.40 (36.32 to 36.48)	14514	270448	52.98 (52.89 to 53.08)
	IMD=8	5743	211423.2	27.89 (27.82 to 27.95)	7653	192101.9	40.29 (40.21 to 40.37)	9727	247540	39.63 (39.55 to 39.72)	13992	238533	58.68 (58.58 to 58.78)
	IMD=9	6474	221146.3	30.22 (30.15 to 30.29)	8887	200816.2	45.07 (44.99 to 45.16)	11229	261403	43.54 (43.45 to 43.63)	16673	253290	66.28 (66.17 to 66.38)
	Most Deprivation	6184	190508.5	33.69 (33.61 to 33.77)	8213	169696.4	49.60 (49.51 to 49.69)	10974	228015	48.83 (48.74 to 48.92)	16079	218190	74.50 (74.39 to 74.61)
2019	Least Deprivation	6835	286122.2	22.62 (22.55 to 22.68)	8852	281432.4	29.91 (29.84 to 29.98)	10142	329097	28.85 (28.77 to 28.92)	13757	333828	38.59 (38.51 to 38.67)
	IMD=2	6792	269113.1	23.95 (23.89 to 24.02)	8455	262556	30.93 (30.86 to 31.01)	10195	313372	30.62 (30.55 to 30.69)	13729	316672	41.16 (41.08 to 41.25)
	IMD=3	6686	258202	24.75 (24.69 to 24.82)	7963	249749.6	30.65 (30.58 to 30.73)	10069	304986	31.16 (31.09 to 31.24)	12969	305435	40.42 (40.34 to 40.50)
	IMD=4	6124	234937.8	25.22 (25.15 to 25.28)	7682	224277.8	33.42 (33.34 to 33.49)	9454	277528	32.59 (32.52 to 32.67)	12567	275526	43.93 (43.85 to 44.02)
	IMD=5	5922	233109	24.66 (24.60 to 24.73)	7541	221460.1	33.24 (33.16 to 33.31)	9240	272207	32.53 (32.46 to 32.61)	12634	269548	45.34 (45.25 to 45.42)
	IMD=6	6019	230542.7	25.76 (25.69 to 25.82)	7974	215942.4	36.32 (36.24 to 36.40)	9709	271007	34.90 (34.82 to 34.97)	13481	266181	49.39 (49.30 to 49.48)
	IMD=7	6302	241606.7	26.23 (26.16 to 26.30)	8202	221236.4	36.99 (36.91 to 37.07)	10142	284252	35.42 (35.34 to 35.49)	14269	274437	51.42 (51.33 to 51.51)
	IMD=8	5703	212323.8	27.52 (27.46 to 27.59)	7431	192146.2	39.14 (39.06 to 39.22)	9477	252552	37.87 (37.79 to 37.95)	13234	242530	54.73 (54.64 to 54.83)
	IMD=9	6385	224398.8	29.57 (29.50 to 29.64)	8468	202089.7	42.86 (42.78 to 42.95)	10868	265931	41.63 (41.55 to 41.72)	15629	256271	61.59 (61.49 to 61.69)
	Most Deprivation	5782	185626.3	32.04 (31.97 to 32.11)	7786	165947	48.26 (48.17 to 48.35)	10233	223710	46.30 (46.21 to 46.39)	14932	214668	70.46 (70.35 to 70.57)
**Osteoarthritis**													
2004	Least Deprivation	2106	143837.9	12.10 (12.04 to 12.15)	3402	148055.3	19.01 (18.94 to 19.08)	3468	155307	18.06 (17.99 to 18.13)	6036	164582	29.41 (29.33 to 29.49)
	IMD=2	2124	134526.8	12.74 (12.68 to 12.79)	3491	140655.3	19.91 (19.84 to 19.98)	3646	146680	19.59 (19.52 to 19.66)	6401	158298	31.46 (31.38 to 31.55)
	IMD=3	2097	128276.9	13.15 (13.09 to 13.21)	3511	134178.5	20.74 (20.67 to 20.81)	3670	139648	20.60 (20.53 to 20.67)	6357	150987	32.38 (32.29 to 32.47)
	IMD=4	1779	112584.9	12.77 (12.72 to 12.83)	3090	117063.5	20.98 (20.91 to 21.05)	3113	122870	19.98 (19.91 to 20.06)	5580	132024	32.64 (32.55 to 32.72)

	IMD=5	1739	107411.4	12.92 (12.86 to 12.97)	2928	111497.9	20.66 (20.60 to 20.73)	3145	117518	20.86 (20.78 to 20.93)	5649	126212	34.25 (34.16 to 34.34)
	IMD=6	1777	105179.8	13.45 (13.39 to 13.51)	3046	109881.8	21.52 (21.45 to 21.59)	3145	114874	21.26 (21.18 to 21.33)	5806	124897	35.05 (34.96 to 35.14)
	IMD=7	1711	101640	13.41 (13.35 to 13.47)	2909	106361.6	21.28 (21.21 to 21.35)	3063	111245	21.46 (21.39 to 21.54)	5588	121203	34.82 (34.73 to 34.91)
	IMD=8	1386	83155.31	13.36 (13.31 to 13.42)	2577	86488.95	23.12 (23.05 to 23.19)	2518	91108	21.75 (21.68 to 21.83)	4891	98847	37.34 (37.24 to 37.43)
	IMD=9	1355	86462.84	12.69 (12.64 to 12.75)	2575	88535.95	22.60 (22.53 to 22.67)	2452	94551	20.55 (20.48 to 20.62)	4912	101372	36.76 (36.67 to 36.86)
	Most Deprivation	1219	77688.09	12.43 (12.38 to 12.49)	2400	77509.83	24.14 (24.06 to 24.21)	2197	84920	20.19 (20.12 to 20.26)	4562	88971	39.22 (39.13 to 39.32)
2005	Least Deprivation	2023	146428	11.45 (11.39 to 11.50)	3229	150497.4	17.81 (17.74 to 17.87)	3608	158576	18.40 (18.33 to 18.47)	6186	168116	29.59 (29.51 to 29.67)
	IMD=2	2123	136354.8	12.63 (12.57 to 12.68)	3387	141949.3	19.27 (19.21 to 19.34)	3844	148810	20.40 (20.33 to 20.47)	6629	160234	32.24 (32.15 to 32.32)
	IMD=3	2114	130289.6	13.08 (13.02 to 13.13)	3453	135678.4	20.32 (20.26 to 20.39)	3819	142395	21.02 (20.94 to 21.09)	6692	153631	33.56 (33.47 to 33.64)
	IMD=4	1787	114341.6	12.63 (12.57 to 12.69)	2824	118319.7	19.06 (18.99 to 19.13)	3281	125038	20.67 (20.60 to 20.74)	5588	134182	32.21 (32.12 to 32.30)
	IMD=5	1846	109190.1	13.61 (13.55 to 13.67)	2956	112627.5	20.79 (20.72 to 20.86)	3384	119704	22.12 (22.04 to 22.19)	5939	128322	35.49 (35.40 to 35.58)
	IMD=6	1713	106585.7	12.85 (12.80 to 12.91)	2985	110286.7	21.25 (21.18 to 21.33)	3190	117076	21.22 (21.14 to 21.29)	5978	126469	35.83 (35.74 to 35.92)
	IMD=7	1689	103538.9	13.09 (13.03 to 13.14)	2875	107455.7	20.97 (20.90 to 21.04)	3195	113878	21.95 (21.87 to 22.02)	5952	123202	36.64 (36.55 to 36.73)
	IMD=8	1368	84198.1	13.24 (13.18 to 13.29)	2476	86901.54	22.31 (22.24 to 22.38)	2604	92747	22.31 (22.23 to 22.38)	5112	100281	38.66 (38.57 to 38.76)
	IMD=9	1395	87697.4	12.96 (12.90 to 13.02)	2554	89059.02	22.54 (22.47 to 22.61)	2660	96571	21.95 (21.88 to 22.02)	5238	103078	38.95 (38.86 to 39.05)
	Most Deprivation	1280	78623.85	13.08 (13.02 to 13.13)	2347	77853.58	23.76 (23.69 to 23.83)	2360	86666	21.50 (21.42 to 21.57)	5015	90518	42.63 (42.53 to 42.73)
2006	Least Deprivation	2105	150576.2	11.55 (11.49 to 11.60)	3236	154162.5	17.45 (17.39 to 17.51)	3675	163238	18.17 (18.11 to 18.24)	6313	172721	29.47 (29.39 to 29.55)
	IMD=2	2086	139865.7	12.05 (11.99 to 12.10)	3136	145259	17.48 (17.41 to 17.54)	3896	152718	20.13 (20.06 to 20.20)	6523	164340	31.00 (30.91 to 31.08)
	IMD=3	2040	133381.2	12.37 (12.31 to 12.43)	3204	138413	18.54 (18.48 to 18.61)	3820	146187	20.50 (20.43 to 20.57)	6478	157463	31.76 (31.68 to 31.85)
	IMD=4	1704	117102.8	11.81 (11.76 to 11.86)	2801	120744.5	18.66 (18.59 to 18.72)	3213	128280	19.80 (19.73 to 19.87)	5654	137496	31.95 (31.87 to 32.04)
	IMD=5	1723	112240.7	12.39 (12.33 to 12.44)	2682	114948.8	18.59 (18.52 to 18.65)	3341	123311	21.27 (21.20 to 21.34)	5645	131538	32.99 (32.90 to 33.08)
	IMD=6	1682	108696.4	12.40 (12.34 to 12.45)	2817	111937.9	19.87 (19.80 to 19.94)	3209	120091	20.87 (20.80 to 20.94)	5941	129244	35.03 (34.94 to 35.12)
	IMD=7	1588	105585.4	12.16 (12.10 to 12.21)	2753	108916.2	19.97 (19.91 to 20.04)	3232	116966	21.76 (21.69 to 21.83)	6015	126113	36.39 (36.29 to 36.48)
	IMD=8	1242	85566.16	11.88 (11.83 to 11.94)	2234	87642.46	20.18 (20.11 to 20.25)	2507	94812	21.13 (21.06 to 21.20)	5024	102035	37.62 (37.53 to 37.71)
	IMD=9	1361	89206.6	12.46 (12.41 to 12.52)	2483	90118.53	21.88 (21.81 to 21.95)	2570	98512	20.91 (20.83 to 20.98)	5270	104852	38.82 (38.73 to 38.92)
	Most Deprivation	1182	79993.23	11.99 (11.94 to 12.05)	2150	78914.43	21.68 (21.61 to 21.76)	2358	88239	21.27 (21.20 to 21.34)	4895	92142	41.22 (41.12 to 41.32)
2007	Least Deprivation	1974	154206.8	10.58 (10.53 to 10.63)	3267	157454	17.22 (17.16 to 17.28)	3628	167394	17.44 (17.38 to 17.51)	6451	176659	29.41 (29.33 to 29.50)
	IMD=2	2123	142999	12.01 (11.95 to 12.06)	3281	148326.6	17.88 (17.82 to 17.94)	3932	156407	19.81 (19.74 to 19.88)	6688	167994	31.07 (30.98 to 31.15)
	IMD=3	2094	136189.3	12.41 (12.35 to 12.47)	3275	140561.4	18.67 (18.60 to 18.73)	3965	149406	20.79 (20.72 to 20.86)	6762	160213	32.58 (32.49 to 32.66)

	IMD=4	1826	119644.6	12.30 (12.25 to 12.36)	2792	122920.9	18.27 (18.21 to 18.34)	3407	131153	20.52 (20.45 to 20.59)	5671	140151	31.45 (31.36 to 31.53)
	IMD=5	1767	114854.5	12.43 (12.38 to 12.49)	2792	116977.8	19.09 (19.02 to 19.15)	3430	126436	21.30 (21.23 to 21.38)	5921	134184	34.03 (33.94 to 34.12)
	IMD=6	1792	111089	12.98 (12.92 to 13.04)	2830	113747.5	19.74 (19.68 to 19.81)	3405	122342	21.81 (21.74 to 21.89)	6061	131212	35.23 (35.14 to 35.32)
	IMD=7	1579	107682.2	11.88 (11.83 to 11.94)	2808	110626.3	20.18 (20.11 to 20.24)	3208	118847	21.30 (21.23 to 21.38)	6121	127683	36.73 (36.64 to 36.82)
	IMD=8	1402	87355.09	13.28 (13.22 to 13.34)	2347	88997.01	20.95 (20.88 to 21.01)	2671	96467	22.32 (22.24 to 22.39)	5097	103494	37.79 (37.70 to 37.89)
	IMD=9	1344	90921.83	12.21 (12.15 to 12.26)	2477	91327.32	21.73 (21.65 to 21.80)	2702	100563	21.73 (21.66 to 21.80)	5468	106568	39.80 (39.71 to 39.90)
	Most Deprivation	1278	81447.34	12.92 (12.87 to 12.98)	2160	80027.92	21.64 (21.57 to 21.71)	2494	89960	22.32 (22.24 to 22.39)	4935	93735	41.10 (41.00 to 41.20)
2008	Least Deprivation	2196	158817.3	11.35 (11.30 to 11.41)	3421	161806.5	17.50 (17.44 to 17.56)	3848	171487	17.98 (17.91 to 18.05)	6616	180608	29.39 (29.31 to 29.48)
	IMD=2	2254	146947	12.34 (12.28 to 12.39)	3349	152142.5	17.84 (17.78 to 17.90)	4123	160185	20.19 (20.12 to 20.26)	6824	171525	31.06 (30.97 to 31.14)
	IMD=3	2231	139966	12.83 (12.77 to 12.89)	3378	144393.9	18.83 (18.76 to 18.90)	4127	152915	21.10 (21.02 to 21.17)	6937	163716	32.76 (32.67 to 32.84)
	IMD=4	1877	122713.7	12.35 (12.30 to 12.41)	2917	125912.5	18.72 (18.66 to 18.79)	3490	134057	20.51 (20.44 to 20.58)	5818	142631	31.80 (31.71 to 31.88)
	IMD=5	1868	117776.6	12.82 (12.76 to 12.87)	2910	119916.5	19.48 (19.42 to 19.55)	3536	129076	21.56 (21.48 to 21.63)	6102	136774	34.59 (34.50 to 34.68)
	IMD=6	1868	113676.4	13.25 (13.19 to 13.31)	2995	116350.7	20.49 (20.42 to 20.56)	3568	125029	22.37 (22.30 to 22.45)	6337	133615	36.39 (36.30 to 36.49)
	IMD=7	1767	110674.3	12.95 (12.90 to 13.01)	2790	113510.8	19.62 (19.56 to 19.69)	3505	121619	22.78 (22.71 to 22.86)	6180	130408	36.50 (36.40 to 36.59)
	IMD=8	1440	89686.77	13.33 (13.27 to 13.38)	2382	91113.35	21.02 (20.95 to 21.09)	2809	98974	23.02 (22.94 to 23.10)	5293	105569	38.86 (38.76 to 38.95)
	IMD=9	1475	93005.84	13.20 (13.14 to 13.25)	2533	93292.18	21.90 (21.82 to 21.97)	2855	102687	22.68 (22.61 to 22.76)	5543	108673	39.98 (39.88 to 40.08)
	Most Deprivation	1325	83175.23	13.16 (13.11 to 13.22)	2257	81614.66	22.50 (22.43 to 22.57)	2711	92038	23.85 (23.78 to 23.93)	5197	95518	42.97 (42.87 to 43.07)
2009	Least Deprivation	2293	162445.2	11.47 (11.41 to 11.52)	3463	165877.9	17.28 (17.22 to 17.35)	3962	176011	17.92 (17.85 to 17.99)	6718	185566	29.01 (28.93 to 29.10)
	IMD=2	2344	150093	12.54 (12.48 to 12.60)	3513	155357.5	18.28 (18.22 to 18.35)	4271	163954	20.38 (20.31 to 20.46)	7013	175278	31.17 (31.09 to 31.26)
	IMD=3	2367	143286.4	13.23 (13.17 to 13.28)	3556	148089.9	19.28 (19.22 to 19.35)	4384	157108	21.71 (21.64 to 21.78)	7241	167988	33.35 (33.26 to 33.44)
	IMD=4	1985	125331	12.70 (12.65 to 12.76)	3024	128537.3	19.05 (18.98 to 19.11)	3626	137183	20.73 (20.66 to 20.81)	5973	145657	32.02 (31.94 to 32.11)
	IMD=5	1930	120347.6	12.96 (12.90 to 13.01)	2944	122536	19.36 (19.30 to 19.43)	3646	131989	21.72 (21.65 to 21.79)	6193	139780	34.43 (34.34 to 34.52)
	IMD=6	1930	115727.4	13.47 (13.41 to 13.53)	3043	118393.4	20.60 (20.53 to 20.67)	3679	127572	22.62 (22.55 to 22.70)	6371	136112	36.07 (35.98 to 36.16)
	IMD=7	1774	112879.3	12.80 (12.75 to 12.86)	2815	115604.1	19.53 (19.46 to 19.59)	3552	124384	22.65 (22.57 to 22.72)	6230	132944	36.27 (36.18 to 36.36)
	IMD=8	1484	91542.65	13.55 (13.49 to 13.61)	2477	92655.47	21.70 (21.63 to 21.77)	2930	101168	23.61 (23.53 to 23.69)	5419	107549	39.43 (39.33 to 39.53)
	IMD=9	1534	94890.79	13.65 (13.59 to 13.71)	2674	94765.38	23.03 (22.96 to 23.11)	2985	104911	23.40 (23.32 to 23.47)	5876	110613	42.06 (41.96 to 42.16)
	Most Deprivation	1354	84132.2	13.38 (13.32 to 13.43)	2280	82466.68	22.39 (22.32 to 22.47)	2783	93198	24.35 (24.27 to 24.43)	5342	96655	43.80 (43.70 to 43.90)
2010	Least Deprivation	2321	166537.9	11.36 (11.31 to 11.41)	3530	170761.5	17.11 (17.04 to 17.17)	4059	180613	17.84 (17.77 to 17.91)	6789	191014	28.49 (28.41 to 28.57)
	IMD=2	2401	153862.3	12.55 (12.49 to 12.60)	3680	159577.3	18.65 (18.59 to 18.72)	4374	168315	20.30 (20.23 to 20.38)	7312	180198	31.65 (31.56 to 31.73)

	IMD=3	2320	146844.5	12.67 (12.62 to 12.73)	3526	151513.7	18.78 (18.71 to 18.85)	4342	161116	20.99 (20.92 to 21.06)	7388	172255	33.27 (33.18 to 33.36)
	IMD=4	2052	128307.8	12.92 (12.86 to 12.98)	3020	131749.3	18.61 (18.54 to 18.67)	3734	140723	20.85 (20.78 to 20.92)	6044	149454	31.67 (31.58 to 31.76)
	IMD=5	1961	123320.5	12.89 (12.84 to 12.95)	2958	125616.2	19.02 (18.95 to 19.09)	3710	135619	21.56 (21.49 to 21.63)	6229	143312	33.87 (33.78 to 33.96)
	IMD=6	1921	118362.3	13.10 (13.04 to 13.16)	2982	121194.7	19.82 (19.75 to 19.88)	3706	130664	22.30 (22.23 to 22.38)	6495	139398	36.09 (36.00 to 36.18)
	IMD=7	1795	115559.6	12.72 (12.66 to 12.78)	3020	118293.1	20.61 (20.55 to 20.68)	3560	127538	22.28 (22.20 to 22.35)	6606	136211	37.78 (37.69 to 37.88)
	IMD=8	1481	93752.13	13.25 (13.19 to 13.31)	2454	94903.07	21.10 (21.03 to 21.17)	2983	103771	23.60 (23.53 to 23.68)	5582	110050	39.96 (39.86 to 40.06)
	IMD=9	1567	96896.88	13.71 (13.66 to 13.77)	2611	96679.59	22.27 (22.20 to 22.34)	3095	107318	23.90 (23.82 to 23.98)	5967	112944	42.18 (42.08 to 42.28)
	Most Deprivation	1411	85599.08	13.77 (13.72 to 13.83)	2308	83769.7	22.71 (22.63 to 22.78)	2889	95059	24.97 (24.89 to 25.05)	5368	98201	43.77 (43.67 to 43.87)
2011	Least Deprivation	2434	169895.4	11.61 (11.55 to 11.66)	3608	175118.1	17.02 (16.96 to 17.08)	4175	184484	17.85 (17.78 to 17.92)	6872	195836	27.99 (27.91 to 28.07)
	IMD=2	2439	156719.7	12.37 (12.32 to 12.43)	3522	163114.7	17.43 (17.37 to 17.50)	4485	171754	20.22 (20.15 to 20.29)	7180	184193	30.31 (30.23 to 30.39)
	IMD=3	2385	149660.4	12.72 (12.66 to 12.77)	3521	154917.3	18.33 (18.27 to 18.40)	4480	164580	21.05 (20.98 to 21.12)	7374	176013	32.44 (32.35 to 32.52)
	IMD=4	2069	131118.2	12.75 (12.70 to 12.81)	3115	134989.1	18.71 (18.64 to 18.77)	3836	144042	20.87 (20.80 to 20.94)	6224	152999	31.83 (31.74 to 31.92)
	IMD=5	1958	125846.1	12.62 (12.57 to 12.68)	2965	128637.4	18.61 (18.55 to 18.68)	3731	138679	21.19 (21.11 to 21.26)	6253	146854	33.18 (33.10 to 33.27)
	IMD=6	1925	120468	12.92 (12.86 to 12.97)	3008	123943.3	19.59 (19.52 to 19.65)	3771	133122	22.25 (22.17 to 22.32)	6524	142363	35.62 (35.53 to 35.71)
	IMD=7	1790	117967.2	12.43 (12.37 to 12.48)	2934	120803.4	19.69 (19.62 to 19.76)	3579	130476	21.90 (21.82 to 21.97)	6482	139065	36.43 (36.34 to 36.52)
	IMD=8	1500	95556.96	13.19 (13.13 to 13.24)	2558	96718.03	21.67 (21.60 to 21.74)	3010	105961	23.34 (23.26 to 23.41)	5664	112361	39.93 (39.83 to 40.03)
	IMD=9	1456	98908.09	12.64 (12.59 to 12.70)	2763	98471.98	23.28 (23.21 to 23.35)	2998	109715	22.90 (22.82 to 22.97)	6258	115010	43.68 (43.58 to 43.78)
	Most Deprivation	1305	86740.88	12.70 (12.65 to 12.76)	2418	84943	23.65 (23.58 to 23.73)	2848	96511	24.40 (24.32 to 24.48)	5654	99705	45.70 (45.60 to 45.80)
2012	Least Deprivation	2398	173251.5	11.10 (11.05 to 11.15)	3676	178958.8	16.86 (16.80 to 16.92)	4068	187564	16.93 (16.86 to 16.99)	6956	199628	27.61 (27.53 to 27.69)
	IMD=2	2359	159520.6	11.70 (11.65 to 11.75)	3627	166503.6	17.51 (17.45 to 17.57)	4377	174168	19.28 (19.21 to 19.35)	7225	187522	29.81 (29.73 to 29.89)
	IMD=3	2398	152110.9	12.46 (12.41 to 12.52)	3624	157669.2	18.47 (18.41 to 18.54)	4481	166748	20.59 (20.52 to 20.66)	7446	178693	32.13 (32.05 to 32.22)
	IMD=4	2088	133618.5	12.51 (12.45 to 12.56)	3089	137721.7	18.13 (18.07 to 18.20)	3813	146374	20.26 (20.18 to 20.33)	6184	155852	30.93 (30.85 to 31.02)
	IMD=5	1948	128074.2	12.18 (12.13 to 12.24)	3059	131145.5	18.83 (18.77 to 18.90)	3714	140365	20.64 (20.57 to 20.71)	6331	149013	33.09 (33.00 to 33.17)
	IMD=6	1929	122589.6	12.68 (12.63 to 12.74)	3084	126160	19.69 (19.63 to 19.76)	3827	135066	22.16 (22.08 to 22.23)	6605	144635	35.48 (35.39 to 35.57)
	IMD=7	1908	120234	12.99 (12.94 to 13.05)	3034	122973.1	19.96 (19.89 to 20.02)	3728	132217	22.43 (22.35 to 22.50)	6644	141009	36.79 (36.70 to 36.88)
	IMD=8	1551	97600.67	13.39 (13.33 to 13.45)	2577	98541.76	21.54 (21.47 to 21.61)	3062	107642	23.42 (23.35 to 23.50)	5694	114179	39.60 (39.50 to 39.69)
	IMD=9	1601	100882.5	13.46 (13.40 to 13.52)	2737	100135	22.66 (22.59 to 22.73)	3164	111438	23.59 (23.51 to 23.66)	6199	116715	42.67 (42.58 to 42.77)
	Most Deprivation	1353	88457.01	12.93 (12.88 to 12.99)	2250	86256.27	21.62 (21.55 to 21.69)	2856	97941	24.16 (24.09 to 24.24)	5462	100975	43.73 (43.62 to 43.83)
2013	Least Deprivation	2491	175055.6	11.34 (11.29 to 11.40)	3911	180907.2	17.66 (17.60 to 17.72)	4171	190414	16.97 (16.90 to 17.03)	7158	202782	27.82 (27.74 to 27.90)

	IMD=2	2559	161111.9	12.49 (12.44 to 12.55)	3850	168093.1	18.38 (18.32 to 18.45)	4487	176922	19.34 (19.27 to 19.41)	7432	190418	30.10 (30.02 to 30.18)
	IMD=3	2562	153586.9	13.14 (13.08 to 13.19)	3890	158838.4	19.64 (19.57 to 19.71)	4653	169220	20.97 (20.90 to 21.04)	7600	181129	32.24 (32.16 to 32.33)
	IMD=4	2189	134662.9	12.97 (12.91 to 13.03)	3277	138661.9	19.04 (18.98 to 19.11)	3937	148503	20.51 (20.44 to 20.58)	6345	157955	31.27 (31.18 to 31.35)
	IMD=5	1984	129218.9	12.26 (12.20 to 12.31)	3346	132223.2	20.40 (20.33 to 20.47)	3663	142522	19.96 (19.89 to 20.03)	6484	151238	33.36 (33.27 to 33.45)
	IMD=6	2093	123219.2	13.65 (13.59 to 13.71)	3144	126768.9	20.02 (19.95 to 20.09)	3880	136931	22.12 (22.05 to 22.19)	6489	146263	34.51 (34.42 to 34.60)
	IMD=7	2000	120594.8	13.53 (13.47 to 13.59)	3127	123406.4	20.58 (20.51 to 20.65)	3797	134543	22.41 (22.33 to 22.48)	6691	143073	36.58 (36.49 to 36.67)
	IMD=8	1573	98000.05	13.43 (13.38 to 13.49)	2808	99521.61	23.21 (23.13 to 23.28)	3064	109791	22.92 (22.85 to 23.00)	5944	116326	40.73 (40.64 to 40.83)
	IMD=9	1667	100853	13.95 (13.89 to 14.01)	2928	100916.9	24.16 (24.08 to 24.23)	3193	113517	23.35 (23.28 to 23.43)	6333	118684	43.14 (43.04 to 43.24)
	Most Deprivation	1454	88740.2	13.81 (13.75 to 13.87)	2545	86133.19	24.60 (24.53 to 24.68)	2919	99534	24.32 (24.25 to 24.40)	5626	102037	44.64 (44.54 to 44.74)
2014	Least Deprivation	2787	177094.1	12.48 (12.43 to 12.54)	4037	183021.7	17.97 (17.91 to 18.04)	4456	192672	17.82 (17.75 to 17.89)	7248	205203	27.73 (27.65 to 27.81)
	IMD=2	2721	162609.8	13.10 (13.04 to 13.15)	4001	169791.5	18.81 (18.74 to 18.87)	4667	178609	19.84 (19.77 to 19.91)	7477	192202	29.94 (29.86 to 30.03)
	IMD=3	2652	155127.2	13.36 (13.31 to 13.42)	3975	160349.4	19.85 (19.78 to 19.92)	4731	171007	20.99 (20.92 to 21.07)	7644	182785	32.12 (32.03 to 32.20)
	IMD=4	2278	136019.4	13.29 (13.23 to 13.35)	3503	139923.9	20.15 (20.08 to 20.22)	4008	149864	20.62 (20.55 to 20.69)	6475	159323	31.61 (31.53 to 31.70)
	IMD=5	2258	130750.9	13.75 (13.69 to 13.81)	3376	133361.5	20.41 (20.34 to 20.47)	3897	143835	20.97 (20.90 to 21.04)	6426	152524	32.81 (32.72 to 32.90)
	IMD=6	2178	124859.8	13.96 (13.90 to 14.02)	3369	127829.1	21.29 (21.22 to 21.36)	3976	138145	22.36 (22.28 to 22.43)	6676	147170	35.33 (35.24 to 35.42)
	IMD=7	2041	122058.8	13.57 (13.51 to 13.63)	3301	124425.2	21.57 (21.50 to 21.64)	3782	134937	22.20 (22.13 to 22.28)	6759	143711	36.90 (36.81 to 36.99)
	IMD=8	1706	99613.39	14.32 (14.26 to 14.38)	2788	100430.3	22.87 (22.80 to 22.95)	3119	110310	23.15 (23.07 to 23.23)	5856	117322	39.84 (39.74 to 39.93)
	IMD=9	1758	102468.5	14.53 (14.47 to 14.59)	3109	101670.5	25.55 (25.48 to 25.63)	3248	113532	23.63 (23.56 to 23.71)	6477	119687	43.87 (43.77 to 43.97)
	Most Deprivation	1538	89558.19	14.63 (14.57 to 14.69)	2727	86547.85	26.26 (26.18 to 26.34)	2935	99584	24.55 (24.47 to 24.62)	5700	102000	45.34 (45.23 to 45.44)
2015	Least Deprivation	2887	179698.7	12.70 (12.64 to 12.75)	4352	185272	19.10 (19.04 to 19.17)	4624	195425	18.14 (18.08 to 18.21)	7572	207807	28.51 (28.43 to 28.59)
	IMD=2	2814	164573.3	13.35 (13.29 to 13.41)	4360	171343.2	20.27 (20.20 to 20.34)	4829	180657	20.22 (20.15 to 20.29)	7953	194258	31.38 (31.30 to 31.47)
	IMD=3	2747	157188.5	13.66 (13.60 to 13.71)	4202	161838.7	20.70 (20.64 to 20.77)	4797	173094	20.96 (20.89 to 21.03)	7948	184649	33.00 (32.91 to 33.09)
	IMD=4	2502	137947.6	14.40 (14.34 to 14.46)	3622	141280.4	20.64 (20.57 to 20.70)	4158	151761	21.06 (20.99 to 21.14)	6744	160997	32.53 (32.45 to 32.62)
	IMD=5	2369	132370.1	14.18 (14.12 to 14.24)	3483	134884.5	20.83 (20.76 to 20.90)	4099	145910	21.68 (21.60 to 21.75)	6665	154365	33.61 (33.52 to 33.70)
	IMD=6	2257	126386.5	14.27 (14.22 to 14.33)	3538	129029.9	22.14 (22.06 to 22.21)	4092	140048	22.62 (22.55 to 22.70)	6958	148784	36.44 (36.35 to 36.53)
	IMD=7	2145	124089.6	13.98 (13.92 to 14.04)	3522	125850.6	22.80 (22.73 to 22.87)	3900	137285	22.41 (22.34 to 22.49)	6923	145471	37.44 (37.34 to 37.53)
	IMD=8	1733	101798.4	14.29 (14.23 to 14.35)	2938	101971.2	23.85 (23.77 to 23.92)	3227	112840	23.41 (23.34 to 23.49)	6034	118847	40.74 (40.64 to 40.84)
	IMD=9	1826	104793	14.90 (14.84 to 14.96)	3168	103095.1	25.63 (25.55 to 25.71)	3378	116362	24.18 (24.10 to 24.26)	6694	121378	44.78 (44.68 to 44.88)
	Most Deprivation	1587	91261.92	14.80 (14.74 to 14.86)	2813	87637.16	26.77 (26.69 to 26.85)	3006	101485	24.55 (24.48 to 24.63)	5891	103763	46.14 (46.04 to 46.24)

2016	Least Deprivation	3010	182651.9	12.95 (12.89 to 13.00)	4475	188250.5	19.26 (19.20 to 19.33)	4829	198751	18.53 (18.47 to 18.60)	7791	211407	28.78 (28.69 to 28.86)
	IMD=2	2927	167393	13.58 (13.53 to 13.64)	4544	173671.8	20.80 (20.73 to 20.87)	4875	183378	20.01 (19.94 to 20.08)	8167	196894	31.73 (31.65 to 31.82)
	IMD=3	2898	160011.6	14.08 (14.02 to 14.14)	4312	164196.5	20.90 (20.83 to 20.97)	4976	175903	21.33 (21.26 to 21.41)	8029	187142	32.85 (32.76 to 32.93)
	IMD=4	2503	140611.2	14.09 (14.03 to 14.15)	3730	143391.9	20.91 (20.84 to 20.98)	4318	154505	21.44 (21.36 to 21.51)	6854	163228	32.59 (32.50 to 32.67)
	IMD=5	2490	135055.5	14.58 (14.52 to 14.64)	3556	137240.1	20.89 (20.82 to 20.96)	4184	148504	21.68 (21.61 to 21.76)	6689	156734	33.19 (33.10 to 33.27)
	IMD=6	2423	129070.8	14.98 (14.92 to 15.04)	3604	131006.6	22.21 (22.14 to 22.28)	4274	142572	23.23 (23.16 to 23.31)	7049	150751	36.50 (36.40 to 36.59)
	IMD=7	2134	127051.7	13.64 (13.58 to 13.69)	3581	128205.1	22.77 (22.70 to 22.84)	3961	139992	22.36 (22.29 to 22.44)	7010	147917	37.36 (37.26 to 37.45)
	IMD=8	1780	104690	14.36 (14.30 to 14.42)	3128	104020.3	25.02 (24.95 to 25.10)	3254	115454	23.15 (23.07 to 23.22)	6265	120981	41.66 (41.56 to 41.75)
	IMD=9	1954	107637.7	15.40 (15.33 to 15.46)	3296	104983	26.25 (26.18 to 26.33)	3566	119004	24.83 (24.75 to 24.91)	6974	123275	46.03 (45.93 to 46.14)
	Most Deprivation	1629	93521.3	14.81 (14.75 to 14.87)	2965	88894.98	27.90 (27.82 to 27.98)	3102	103560	24.83 (24.75 to 24.91)	6161	105112	47.63 (47.53 to 47.74)
2017	Least Deprivation	3035	184999.6	12.83 (12.77 to 12.89)	4619	190525.1	19.58 (19.51 to 19.64)	4956	201896	18.62 (18.56 to 18.69)	8025	214595	29.07 (28.99 to 29.16)
	IMD=2	3108	169834.6	14.16 (14.10 to 14.22)	4622	175330.9	20.87 (20.80 to 20.94)	5069	186724	20.37 (20.30 to 20.44)	8260	199625	31.59 (31.50 to 31.68)
	IMD=3	3095	162256.9	14.76 (14.70 to 14.82)	4587	166046.9	21.88 (21.81 to 21.96)	5170	178977	21.69 (21.62 to 21.76)	8388	190089	33.70 (33.61 to 33.78)
	IMD=4	2572	142979.3	14.17 (14.11 to 14.22)	3738	145340.8	20.59 (20.52 to 20.66)	4361	157572	21.14 (21.07 to 21.21)	6872	165961	32.05 (31.96 to 32.14)
	IMD=5	2504	137135.3	14.41 (14.35 to 14.47)	3665	139137.1	21.13 (21.06 to 21.20)	4295	151534	21.74 (21.67 to 21.81)	6841	159464	33.24 (33.15 to 33.32)
	IMD=6	2510	131306.2	15.28 (15.21 to 15.34)	3671	132551.1	22.34 (22.27 to 22.41)	4285	145620	22.75 (22.67 to 22.82)	7166	153235	36.46 (36.37 to 36.55)
	IMD=7	2277	129440.4	14.30 (14.24 to 14.36)	3703	129792.7	23.23 (23.16 to 23.31)	4096	143071	22.60 (22.52 to 22.67)	7175	150391	37.57 (37.47 to 37.66)
	IMD=8	1898	107373.6	14.86 (14.79 to 14.92)	3168	105577.4	24.86 (24.78 to 24.93)	3350	118742	23.13 (23.06 to 23.21)	6347	123281	41.41 (41.31 to 41.51)
	IMD=9	1916	110215.6	14.70 (14.64 to 14.76)	3301	106712	25.85 (25.77 to 25.93)	3542	122321	23.97 (23.89 to 24.05)	6986	125999	45.19 (45.09 to 45.30)
	Most Deprivation	1770	95348.35	15.95 (15.89 to 16.02)	3053	89973.77	28.39 (28.30 to 28.47)	3235	106000	25.39 (25.31 to 25.47)	6452	106938	49.14 (49.03 to 49.24)
2018	Least Deprivation	3246	188441.6	13.38 (13.32 to 13.44)	4803	193797.1	19.89 (19.82 to 19.96)	5115	206189	18.67 (18.60 to 18.74)	8216	219111	28.98 (28.89 to 29.06)
	IMD=2	3215	172991	14.27 (14.21 to 14.33)	4744	178402	20.97 (20.90 to 21.04)	5257	191351	20.42 (20.35 to 20.49)	8508	204352	31.61 (31.53 to 31.70)
	IMD=3	3195	166392.7	14.76 (14.70 to 14.82)	4622	169883.5	21.46 (21.39 to 21.53)	5422	184405	21.91 (21.83 to 21.98)	8495	195443	32.98 (32.90 to 33.07)
	IMD=4	2657	146808.9	14.14 (14.08 to 14.20)	4008	149025.5	21.44 (21.37 to 21.51)	4452	162582	20.76 (20.69 to 20.83)	7195	170838	32.46 (32.37 to 32.55)
	IMD=5	2661	140845.3	14.80 (14.74 to 14.86)	3858	142669.2	21.62 (21.55 to 21.69)	4427	155932	21.62 (21.55 to 21.69)	7008	163755	33.07 (32.99 to 33.16)
	IMD=6	2476	134029.5	14.67 (14.61 to 14.73)	3733	135088.6	22.18 (22.11 to 22.25)	4360	149685	22.43 (22.36 to 22.51)	7279	157122	36.03 (35.94 to 36.12)
	IMD=7	2373	132470.7	14.46 (14.40 to 14.52)	3818	132213	23.46 (23.38 to 23.53)	4139	147515	22.07 (21.99 to 22.14)	7341	154283	37.42 (37.33 to 37.51)
	IMD=8	1969	110214.6	15.16 (15.10 to 15.22)	3314	107843.7	25.55 (25.47 to 25.63)	3485	122555	23.37 (23.30 to 23.45)	6516	126689	41.35 (41.26 to 41.45)
	IMD=9	2003	113135.3	15.10 (15.04 to 15.16)	3442	109215	26.45 (26.37 to 26.53)	3614	126270	23.68 (23.60 to 23.76)	7062	129708	44.36 (44.26 to 44.46)

	Most Deprivation	1773	97150.69	15.49 (15.43 to 15.56)	3048	91351.33	27.93 (27.85 to 28.01)	3289	108833	25.10 (25.02 to 25.18)	6422	109272	47.81 (47.70 to 47.91)
2019	Least Deprivation	3501	188150.3	14.32 (14.26 to 14.38)	5158	193463.8	21.25 (21.18 to 21.32)	5518	209089	19.71 (19.63 to 19.78)	8747	222009	30.22 (30.13 to 30.30)
	IMD=2	3340	172077.3	14.78 (14.72 to 14.84)	4957	177143.7	21.92 (21.85 to 21.99)	5497	193921	20.91 (20.84 to 20.99)	8878	206853	32.37 (32.29 to 32.46)
	IMD=3	3183	162965.4	14.91 (14.85 to 14.97)	4569	166116.6	21.56 (21.49 to 21.63)	5465	186729	21.65 (21.57 to 21.72)	8438	197415	32.27 (32.18 to 32.35)
	IMD=4	2808	144014	15.12 (15.06 to 15.18)	4159	145795.5	22.65 (22.58 to 22.72)	4686	164249	21.51 (21.43 to 21.58)	7430	172422	33.02 (32.93 to 33.10)
	IMD=5	2679	140627.3	14.84 (14.78 to 14.90)	4118	141717.1	23.07 (23.00 to 23.15)	4491	158813	21.39 (21.32 to 21.46)	7365	165961	34.10 (34.01 to 34.19)
	IMD=6	2561	133575.4	15.17 (15.11 to 15.23)	3905	133895.9	23.29 (23.22 to 23.36)	4400	151378	22.30 (22.23 to 22.38)	7412	158285	36.31 (36.22 to 36.40)
	IMD=7	2399	131911.6	14.67 (14.60 to 14.73)	3768	130851.8	23.31 (23.23 to 23.38)	4160	148970	21.88 (21.81 to 21.96)	7255	155120	36.67 (36.58 to 36.76)
	IMD=8	1992	109418.5	15.21 (15.15 to 15.27)	3265	106154.3	25.53 (25.45 to 25.61)	3470	124217	22.80 (22.73 to 22.88)	6412	127428	40.42 (40.33 to 40.52)
	IMD=9	2039	113001.9	15.31 (15.25 to 15.37)	3507	108420	27.05 (26.97 to 27.13)	3623	127198	23.49 (23.41 to 23.56)	7084	130013	44.40 (44.30 to 44.51)
	Most Deprivation	1668	94052.49	14.96 (14.90 to 15.02)	2895	88095.51	27.28 (27.20 to 27.36)	3183	106629	24.61 (24.53 to 24.69)	6173	106827	46.78 (46.67 to 46.88)

Supplementary Table S4. Standardised incidence and prevalence of low back pain and osteoarthritis by index of multiple derivation and age-strata between 2004-2019

For low back pain: the standard population was the population of England 2019 (ONS code: E92000001) aged 15 years and over; for osteoarthritis: the standard population was the population of England 2019 (ONS code: E92000001) aged 45 years and over.
IMD indicates index of multiple deprivation IR indicates incidence rates; PR indicates prevalence; CI indicates confidence interval.

Year	IMD decile	Low back pain						Osteoarthritis					
		Incidence			Prevalence			Incidence			Prevalence		
		Numerator	Denominator	IR (95 % CI)	Numerator	Denominator	PR (95 % CI)	Numerator	Denominator	IR (95 % CI)	Numerator	Denominator	PR (95 % CI)
**15-24 years**													
2004	Least Deprivation	696	44122.37	15.77 (14.60 to 16.95)	897	47997	18.69 (17.47 to 19.91)	-	-	-	-	-	-
	IMD=2	794	46780.73	16.97 (15.79 to 18.15)	1013	50947	19.88 (18.66 to 21.11)	-	-	-	-	-	-
	IMD=3	870	44623.09	19.50 (18.20 to 20.79)	1085	48461	22.39 (21.06 to 23.72)	-	-	-	-	-	-
	IMD=4	834	43498.65	19.17 (17.87 to 20.47)	1048	47642	22.00 (20.67 to 23.33)	-	-	-	-	-	-
	IMD=5	902	42635.59	21.16 (19.78 to 22.54)	1109	46545	23.83 (22.42 to 25.23)	-	-	-	-	-	-
	IMD=6	934	44272.06	21.10 (19.74 to 22.45)	1194	48056	24.85 (23.44 to 26.26)	-	-	-	-	-	-
	IMD=7	1023	49256.87	20.77 (19.50 to 22.04)	1302	53765	24.22 (22.90 to 25.53)	-	-	-	-	-	-
	IMD=8	917	46285.12	19.81 (18.53 to 21.09)	1188	50284	23.63 (22.28 to 24.97)	-	-	-	-	-	-
	IMD=9	1132	53618.21	21.11 (19.88 to 22.34)	1449	58194	24.90 (23.62 to 26.18)	-	-	-	-	-	-
	Most Deprivation	1121	51260.27	21.87 (20.59 to 23.15)	1483	55541	26.70 (25.34 to 28.06)	-	-	-	-	-	-
2005	Least Deprivation	807	45067.53	17.91 (16.67 to 19.14)	1007	49193	20.47 (19.21 to 21.73)	-	-	-	-	-	-
	IMD=2	880	47650.68	18.47 (17.25 to 19.69)	1099	52160	21.07 (19.82 to 22.32)	-	-	-	-	-	-
	IMD=3	874	45514.81	19.20 (17.93 to 20.48)	1155	49586	23.29 (21.95 to 24.64)	-	-	-	-	-	-
	IMD=4	926	44189.1	20.96 (19.61 to 22.31)	1178	48526	24.28 (22.89 to 25.66)	-	-	-	-	-	-
	IMD=5	872	43524.8	20.03 (18.70 to 21.36)	1133	47647	23.78 (22.39 to 25.16)	-	-	-	-	-	-
	IMD=6	962	45415.8	21.18 (19.84 to 22.52)	1228	49649	24.73 (23.35 to 26.12)	-	-	-	-	-	-
	IMD=7	1042	50388.78	20.68 (19.42 to 21.93)	1377	55594	24.77 (23.46 to 26.08)	-	-	-	-	-	-
	IMD=8	973	47517.15	20.48 (19.19 to 21.76)	1257	52031	24.16 (22.82 to 25.49)	-	-	-	-	-	-
	IMD=9	1162	54818.03	21.20 (19.98 to 22.42)	1506	60096	25.06 (23.79 to 26.33)	-	-	-	-	-	-
	Most Deprivation	1233	53045.99	23.24 (21.95 to 24.54)	1646	58085	28.34 (26.97 to 29.71)	-	-	-	-	-	-
2006	Least Deprivation	818	46495.38	17.59 (16.39 to 18.80)	1044	50666	20.61 (19.36 to 21.86)	-	-	-	-	-	-
	IMD=2	963	48783.34	19.74 (18.49 to 20.99)	1207	53480	22.57 (21.30 to 23.84)	-	-	-	-	-	-

	IMD=3	917	46585.79	19.68 (18.41 to 20.96)	1201	50924	23.58 (22.25 to 24.92)	-	-	-	-	-	-
	IMD=4	885	45241.32	19.56 (18.27 to 20.85)	1162	49995	23.24 (21.91 to 24.58)	-	-	-	-	-	-
	IMD=5	912	44601.84	20.45 (19.12 to 21.77)	1204	49026	24.56 (23.17 to 25.95)	-	-	-	-	-	-
	IMD=6	1000	46789.32	21.37 (20.05 to 22.70)	1299	51639	25.16 (23.79 to 26.52)	-	-	-	-	-	-
	IMD=7	1046	51839.86	20.18 (18.95 to 21.40)	1387	57705	24.04 (22.77 to 25.30)	-	-	-	-	-	-
	IMD=8	1044	48746.98	21.42 (20.12 to 22.72)	1324	53836	24.59 (23.27 to 25.92)	-	-	-	-	-	-
	IMD=9	1203	56242.32	21.39 (20.18 to 22.60)	1603	61950	25.88 (24.61 to 27.14)	-	-	-	-	-	-
	Most Deprivation	1195	54775.05	21.82 (20.58 to 23.05)	1628	60307	27.00 (25.68 to 28.31)	-	-	-	-	-	-
2007	Least Deprivation	911	47724.68	19.09 (17.85 to 20.33)	1141	52431	21.76 (20.50 to 23.02)	-	-	-	-	-	-
	IMD=2	954	49936.49	19.10 (17.89 to 20.32)	1251	54832	22.82 (21.55 to 24.08)	-	-	-	-	-	-
	IMD=3	997	47517.75	20.98 (19.68 to 22.28)	1271	52246	24.33 (22.99 to 25.66)	-	-	-	-	-	-
	IMD=4	915	46219.39	19.80 (18.51 to 21.08)	1222	51112	23.91 (22.57 to 25.25)	-	-	-	-	-	-
	IMD=5	958	45814.31	20.91 (19.59 to 22.23)	1289	50421	25.56 (24.17 to 26.96)	-	-	-	-	-	-
	IMD=6	1004	48081.33	20.88 (19.59 to 22.17)	1324	53034	24.97 (23.62 to 26.31)	-	-	-	-	-	-
	IMD=7	1169	52952.51	22.08 (20.81 to 23.34)	1522	58834	25.87 (24.57 to 27.17)	-	-	-	-	-	-
	IMD=8	1099	49704.06	22.11 (20.80 to 23.42)	1445	54930	26.31 (24.95 to 27.66)	-	-	-	-	-	-
	IMD=9	1318	57465.52	22.94 (21.70 to 24.17)	1750	63441	27.58 (26.29 to 28.88)	-	-	-	-	-	-
	Most Deprivation	1258	56257.96	22.36 (21.13 to 23.60)	1713	62145	27.56 (26.26 to 28.87)	-	-	-	-	-	-
2008	Least Deprivation	852	49342.11	17.27 (16.11 to 18.43)	1118	53909	20.74 (19.52 to 21.95)	-	-	-	-	-	-
	IMD=2	894	51363.05	17.41 (16.26 to 18.55)	1183	56270	21.02 (19.83 to 22.22)	-	-	-	-	-	-
	IMD=3	932	48985.74	19.03 (17.80 to 20.25)	1233	53645	22.98 (21.70 to 24.27)	-	-	-	-	-	-
	IMD=4	943	47467.29	19.87 (18.60 to 21.13)	1294	52254	24.76 (23.41 to 26.11)	-	-	-	-	-	-
	IMD=5	974	47232.4	20.62 (19.33 to 21.92)	1272	51843	24.54 (23.19 to 25.88)	-	-	-	-	-	-
	IMD=6	1087	49683.36	21.88 (20.58 to 23.18)	1435	54614	26.28 (24.92 to 27.63)	-	-	-	-	-	-
	IMD=7	1117	55002.59	20.31 (19.12 to 21.50)	1543	60941	25.32 (24.06 to 26.58)	-	-	-	-	-	-
	IMD=8	1081	51357.48	21.05 (19.79 to 22.30)	1478	56673	26.08 (24.75 to 27.41)	-	-	-	-	-	-
	IMD=9	1300	59067.56	22.01 (20.81 to 23.21)	1770	65169	27.16 (25.89 to 28.43)	-	-	-	-	-	-
	Most Deprivation	1379	57706.4	23.90 (22.64 to 25.16)	1922	63884	30.09 (28.74 to 31.43)	-	-	-	-	-	-
2009	Least Deprivation	921	50499.58	18.24 (17.06 to 19.42)	1175	55417	21.20 (19.99 to 22.42)	-	-	-	-	-	-

	IMD=2	963	52166.01	18.46 (17.29 to 19.63)	1276	57433	22.22 (21.00 to 23.44)	-	-	-	-	-	-
	IMD=3	955	50307.7	18.98 (17.78 to 20.19)	1267	55348	22.89 (21.63 to 24.15)	-	-	-	-	-	-
	IMD=4	993	48299.44	20.56 (19.28 to 21.84)	1334	53490	24.94 (23.60 to 26.28)	-	-	-	-	-	-
	IMD=5	1007	48149.93	20.91 (19.62 to 22.21)	1348	53204	25.34 (23.98 to 26.69)	-	-	-	-	-	-
	IMD=6	1123	50729.33	22.14 (20.84 to 23.43)	1473	56015	26.30 (24.95 to 27.64)	-	-	-	-	-	-
	IMD=7	1188	56141.43	21.16 (19.96 to 22.36)	1609	62667	25.68 (24.42 to 26.93)	-	-	-	-	-	-
	IMD=8	1172	52113.11	22.49 (21.20 to 23.78)	1569	57852	27.12 (25.78 to 28.46)	-	-	-	-	-	-
	IMD=9	1343	59891.34	22.42 (21.22 to 23.62)	1825	66401	27.48 (26.22 to 28.75)	-	-	-	-	-	-
	Most Deprivation	1454	58084.41	25.03 (23.75 to 26.32)	2002	64631	30.98 (29.62 to 32.33)	-	-	-	-	-	-
2010	Least Deprivation	930	51780.86	17.96 (16.81 to 19.11)	1204	56685	21.24 (20.04 to 22.44)	-	-	-	-	-	-
	IMD=2	1007	53142.44	18.95 (17.78 to 20.12)	1299	58635	22.15 (20.95 to 23.36)	-	-	-	-	-	-
	IMD=3	983	52112.5	18.86 (17.68 to 20.04)	1301	57287	22.71 (21.48 to 23.94)	-	-	-	-	-	-
	IMD=4	1003	49090.06	20.43 (19.17 to 21.70)	1353	54437	24.85 (23.53 to 26.18)	-	-	-	-	-	-
	IMD=5	1027	49068.43	20.93 (19.65 to 22.21)	1384	54117	25.57 (24.23 to 26.92)	-	-	-	-	-	-
	IMD=6	1098	51454.46	21.34 (20.08 to 22.60)	1496	56938	26.27 (24.94 to 27.61)	-	-	-	-	-	-
	IMD=7	1174	56755.8	20.69 (19.50 to 21.87)	1587	63100	25.15 (23.91 to 26.39)	-	-	-	-	-	-
	IMD=8	1120	53073.98	21.10 (19.87 to 22.34)	1558	58830	26.48 (25.17 to 27.80)	-	-	-	-	-	-
	IMD=9	1366	60383.05	22.62 (21.42 to 23.82)	1894	67037	28.25 (26.98 to 29.53)	-	-	-	-	-	-
	Most Deprivation	1467	58305.25	25.16 (23.87 to 26.45)	2038	64958	31.37 (30.01 to 32.74)	-	-	-	-	-	-
2011	Least Deprivation	1020	53033.33	19.23 (18.05 to 20.41)	1321	58101	22.74 (21.51 to 23.96)	-	-	-	-	-	-
	IMD=2	1093	53944.55	20.26 (19.06 to 21.46)	1412	59301	23.81 (22.57 to 25.05)	-	-	-	-	-	-
	IMD=3	1130	53094.47	21.28 (20.04 to 22.52)	1469	58824	24.97 (23.70 to 26.25)	-	-	-	-	-	-
	IMD=4	1055	49747.56	21.21 (19.93 to 22.49)	1406	55201	25.47 (24.14 to 26.80)	-	-	-	-	-	-
	IMD=5	1072	49686.49	21.58 (20.28 to 22.87)	1415	55040	25.71 (24.37 to 27.05)	-	-	-	-	-	-
	IMD=6	1107	52314	21.16 (19.91 to 22.41)	1529	57947	26.39 (25.06 to 27.71)	-	-	-	-	-	-
	IMD=7	1192	57711.55	20.65 (19.48 to 21.83)	1635	64431	25.38 (24.15 to 26.61)	-	-	-	-	-	-
	IMD=8	1215	53917.03	22.53 (21.27 to 23.80)	1693	59995	28.22 (26.87 to 29.56)	-	-	-	-	-	-
	IMD=9	1388	61087.08	22.72 (21.53 to 23.92)	1941	67926	28.58 (27.30 to 29.85)	-	-	-	-	-	-
	Most Deprivation	1402	58536.55	23.95 (22.70 to 25.20)	1959	65410	29.95 (28.62 to 31.28)	-	-	-	-	-	-

2012	Least Deprivation	966	54286.02	17.79 (16.67 to 18.92)	1288	59309	21.72 (20.53 to 22.90)	-	-	-	-	-	-
	IMD=2	1033	54857.65	18.83 (17.68 to 19.98)	1402	60150	23.31 (22.09 to 24.53)	-	-	-	-	-	-
	IMD=3	1011	53609.08	18.86 (17.70 to 20.02)	1374	59233	23.20 (21.97 to 24.42)	-	-	-	-	-	-
	IMD=4	999	50516.27	19.78 (18.55 to 21.00)	1361	56015	24.30 (23.01 to 25.59)	-	-	-	-	-	-
	IMD=5	1048	50324.69	20.82 (19.56 to 22.09)	1420	55597	25.54 (24.21 to 26.87)	-	-	-	-	-	-
	IMD=6	1164	53259.55	21.86 (20.60 to 23.11)	1591	58723	27.09 (25.76 to 28.42)	-	-	-	-	-	-
	IMD=7	1255	57941.9	21.66 (20.46 to 22.86)	1668	64386	25.91 (24.66 to 27.15)	-	-	-	-	-	-
	IMD=8	1196	54492.44	21.95 (20.70 to 23.19)	1663	60436	27.52 (26.19 to 28.84)	-	-	-	-	-	-
	IMD=9	1492	61653.38	24.20 (22.97 to 25.43)	2073	68270	30.36 (29.06 to 31.67)	-	-	-	-	-	-
	Most Deprivation	1452	58890.64	24.66 (23.39 to 25.92)	2047	65427	31.29 (29.93 to 32.64)	-	-	-	-	-	-
2013	Least Deprivation	1012	55417.39	18.26 (17.14 to 19.39)	1317	60788	21.67 (20.50 to 22.84)	-	-	-	-	-	-
	IMD=2	1040	55768.66	18.65 (17.52 to 19.78)	1419	61618	23.03 (21.83 to 24.23)	-	-	-	-	-	-
	IMD=3	967	54103.68	17.87 (16.75 to 19.00)	1341	60054	22.33 (21.13 to 23.53)	-	-	-	-	-	-
	IMD=4	989	51092.46	19.36 (18.15 to 20.56)	1356	56799	23.87 (22.60 to 25.14)	-	-	-	-	-	-
	IMD=5	1041	50484.17	20.62 (19.37 to 21.87)	1399	56163	24.91 (23.60 to 26.21)	-	-	-	-	-	-
	IMD=6	1132	53244.96	21.26 (20.02 to 22.50)	1556	59328	26.23 (24.92 to 27.53)	-	-	-	-	-	-
	IMD=7	1244	57924.49	21.48 (20.28 to 22.67)	1700	65147	26.09 (24.85 to 27.34)	-	-	-	-	-	-
	IMD=8	1137	54364.5	20.91 (19.70 to 22.13)	1591	61043	26.06 (24.78 to 27.34)	-	-	-	-	-	-
	IMD=9	1443	61514.52	23.46 (22.25 to 24.67)	1999	69302	28.84 (27.58 to 30.11)	-	-	-	-	-	-
	Most Deprivation	1467	58136.52	25.23 (23.94 to 26.52)	2121	65619	32.32 (30.95 to 33.70)	-	-	-	-	-	-
2014	Least Deprivation	1000	57133.46	17.50 (16.42 to 18.59)	1301	62403	20.85 (19.72 to 21.98)	-	-	-	-	-	-
	IMD=2	1108	57114.52	19.40 (18.26 to 20.54)	1475	63202	23.34 (22.15 to 24.53)	-	-	-	-	-	-
	IMD=3	1034	54998.49	18.80 (17.65 to 19.95)	1379	60761	22.70 (21.50 to 23.89)	-	-	-	-	-	-
	IMD=4	1029	51918.91	19.82 (18.61 to 21.03)	1416	57567	24.60 (23.32 to 25.88)	-	-	-	-	-	-
	IMD=5	983	50968.2	19.29 (18.08 to 20.49)	1330	56233	23.65 (22.38 to 24.92)	-	-	-	-	-	-
	IMD=6	1136	53449.57	21.25 (20.02 to 22.49)	1550	59222	26.17 (24.87 to 27.48)	-	-	-	-	-	-
	IMD=7	1221	58119.24	21.01 (19.83 to 22.19)	1689	64853	26.04 (24.80 to 27.29)	-	-	-	-	-	-
	IMD=8	1232	54824.76	22.47 (21.22 to 23.73)	1681	60930	27.59 (26.27 to 28.91)	-	-	-	-	-	-
	IMD=9	1391	61726.14	22.54 (21.35 to 23.72)	1944	68680	28.31 (27.05 to 29.56)	-	-	-	-	-	-

	Most Deprivation	1475	58045	25.41 (24.11 to 26.71)	2103	64739	32.48 (31.10 to 33.87)	-	-	-	-	-	-
2015	Least Deprivation	962	59991.32	16.04 (15.02 to 17.05)	1285	64979	19.78 (18.69 to 20.86)	-	-	-	-	-	-
	IMD=2	1082	58461.22	18.51 (17.41 to 19.61)	1443	64272	22.45 (21.29 to 23.61)	-	-	-	-	-	-
	IMD=3	1047	56791.9	18.44 (17.32 to 19.55)	1395	62420	22.35 (21.18 to 23.52)	-	-	-	-	-	-
	IMD=4	1000	53176.74	18.81 (17.64 to 19.97)	1364	58559	23.29 (22.06 to 24.53)	-	-	-	-	-	-
	IMD=5	1036	52055.53	19.90 (18.69 to 21.11)	1332	57218	23.28 (22.03 to 24.53)	-	-	-	-	-	-
	IMD=6	1164	54182.37	21.48 (20.25 to 22.72)	1559	59944	26.01 (24.72 to 27.30)	-	-	-	-	-	-
	IMD=7	1299	58896.51	22.06 (20.86 to 23.26)	1740	65485	26.57 (25.32 to 27.82)	-	-	-	-	-	-
	IMD=8	1286	55372.66	23.22 (21.96 to 24.49)	1760	61380	28.67 (27.33 to 30.01)	-	-	-	-	-	-
	IMD=9	1391	63111.11	22.04 (20.88 to 23.20)	1962	69918	28.06 (26.82 to 29.30)	-	-	-	-	-	-
	Most Deprivation	1487	58672.36	25.34 (24.06 to 26.63)	2098	65288	32.13 (30.76 to 33.51)	-	-	-	-	-	-
2016	Least Deprivation	968	63294.99	15.29 (14.33 to 16.26)	1283	68384	18.76 (17.74 to 19.79)	-	-	-	-	-	-
	IMD=2	942	60478.43	15.58 (14.58 to 16.57)	1284	66307	19.36 (18.31 to 20.42)	-	-	-	-	-	-
	IMD=3	991	58705.61	16.88 (15.83 to 17.93)	1343	64249	20.90 (19.79 to 22.02)	-	-	-	-	-	-
	IMD=4	948	54609.82	17.36 (16.25 to 18.46)	1320	59941	22.02 (20.83 to 23.21)	-	-	-	-	-	-
	IMD=5	978	53339.43	18.34 (17.19 to 19.48)	1330	58339	22.80 (21.57 to 24.02)	-	-	-	-	-	-
	IMD=6	1091	55185.17	19.77 (18.60 to 20.94)	1459	60667	24.05 (22.82 to 25.28)	-	-	-	-	-	-
	IMD=7	1198	60128.09	19.92 (18.80 to 21.05)	1636	66480	24.61 (23.42 to 25.80)	-	-	-	-	-	-
	IMD=8	1164	56653.75	20.55 (19.37 to 21.73)	1663	62366	26.67 (25.38 to 27.95)	-	-	-	-	-	-
	IMD=9	1337	64312.2	20.79 (19.67 to 21.90)	1878	70859	26.50 (25.30 to 27.70)	-	-	-	-	-	-
	Most Deprivation	1430	59682.47	23.96 (22.72 to 25.20)	1986	66003	30.09 (28.77 to 31.41)	-	-	-	-	-	-
2017	Least Deprivation	949	66753.63	14.22 (13.31 to 15.12)	1197	71973	16.63 (15.69 to 17.57)	-	-	-	-	-	-
	IMD=2	990	62734.47	15.78 (14.80 to 16.76)	1292	68474	18.87 (17.84 to 19.90)	-	-	-	-	-	-
	IMD=3	947	60419.62	15.67 (14.68 to 16.67)	1226	65960	18.59 (17.55 to 19.63)	-	-	-	-	-	-
	IMD=4	932	56456.49	16.51 (15.45 to 17.57)	1234	61805	19.97 (18.85 to 21.08)	-	-	-	-	-	-
	IMD=5	966	54299.64	17.79 (16.67 to 18.91)	1242	59344	20.93 (19.76 to 22.09)	-	-	-	-	-	-
	IMD=6	1027	56501.23	18.18 (17.06 to 19.29)	1371	61988	22.12 (20.95 to 23.29)	-	-	-	-	-	-
	IMD=7	1131	61345.3	18.44 (17.36 to 19.51)	1559	67746	23.01 (21.87 to 24.15)	-	-	-	-	-	-
	IMD=8	1119	58060.48	19.27 (18.14 to 20.40)	1511	63893	23.65 (22.46 to 24.84)	-	-	-	-	-	-

	IMD=9	1313	65833.78	19.94 (18.87 to 21.02)	1822	72391	25.17 (24.01 to 26.32)	-	-	-	-	-	-
	Most Deprivation	1427	60917.59	23.43 (22.21 to 24.64)	1956	67272	29.08 (27.79 to 30.36)	-	-	-	-	-	-
2018	Least Deprivation	920	71063.38	12.95 (12.11 to 13.78)	1191	76494	15.57 (14.69 to 16.45)	-	-	-	-	-	-
	IMD=2	976	65642.91	14.87 (13.94 to 15.80)	1274	71731	17.76 (16.79 to 18.74)	-	-	-	-	-	-
	IMD=3	966	62903.81	15.36 (14.39 to 16.33)	1270	68675	18.49 (17.48 to 19.51)	-	-	-	-	-	-
	IMD=4	1022	59002.49	17.32 (16.26 to 18.38)	1324	64577	20.50 (19.40 to 21.61)	-	-	-	-	-	-
	IMD=5	955	56145.74	17.01 (15.93 to 18.09)	1245	61344	20.30 (19.17 to 21.42)	-	-	-	-	-	-
	IMD=6	1000	57826.68	17.29 (16.22 to 18.36)	1321	63643	20.76 (19.64 to 21.88)	-	-	-	-	-	-
	IMD=7	1100	63749.53	17.26 (16.24 to 18.27)	1498	70766	21.17 (20.10 to 22.24)	-	-	-	-	-	-
	IMD=8	1064	60136.88	17.69 (16.63 to 18.76)	1482	66274	22.36 (21.22 to 23.50)	-	-	-	-	-	-
	IMD=9	1362	67854.76	20.07 (19.01 to 21.14)	1840	74760	24.61 (23.49 to 25.74)	-	-	-	-	-	-
	Most Deprivation	1292	62529.22	20.66 (19.54 to 21.79)	1854	69458	26.69 (25.48 to 27.91)	-	-	-	-	-	-
2019	Least Deprivation	932	73472.55	12.69 (11.87 to 13.50)	1153	80293	14.36 (13.53 to 15.19)	-	-	-	-	-	-
	IMD=2	910	66892.54	13.60 (12.72 to 14.49)	1189	74335	16.00 (15.09 to 16.90)	-	-	-	-	-	-
	IMD=3	853	62612.75	13.62 (12.71 to 14.54)	1111	70265	15.81 (14.88 to 16.74)	-	-	-	-	-	-
	IMD=4	888	58852.35	15.09 (14.10 to 16.08)	1169	66309	17.63 (16.62 to 18.64)	-	-	-	-	-	-
	IMD=5	902	56514.27	15.96 (14.92 to 17.00)	1178	62720	18.78 (17.71 to 19.85)	-	-	-	-	-	-
	IMD=6	949	58519.14	16.22 (15.19 to 17.25)	1275	65296	19.53 (18.45 to 20.60)	-	-	-	-	-	-
	IMD=7	1078	64394.8	16.74 (15.74 to 17.74)	1443	72294	19.96 (18.93 to 20.99)	-	-	-	-	-	-
	IMD=8	1095	60088.7	18.22 (17.14 to 19.30)	1454	67499	21.54 (20.43 to 22.65)	-	-	-	-	-	-
	IMD=9	1268	68332	18.56 (17.54 to 19.58)	1712	76059	22.51 (21.44 to 23.58)	-	-	-	-	-	-
	Most Deprivation	1213	60616.06	20.01 (18.89 to 21.14)	1669	67853	24.60 (23.42 to 25.78)	-	-	-	-	-	-
**25-34 years**													
2004	Least Deprivation	1304	50746.32	25.70 (24.30 to 27.09)	1845	57992	31.81 (30.36 to 33.27)	-	-	-	-	-	-
	IMD=2	1368	52515.18	26.05 (24.67 to 27.43)	1926	60004	32.10 (30.66 to 33.53)	-	-	-	-	-	-
	IMD=3	1367	51297.3	26.65 (25.24 to 28.06)	1941	58655	33.09 (31.62 to 34.56)	-	-	-	-	-	-
	IMD=4	1311	49488.57	26.49 (25.06 to 27.92)	1880	56938	33.02 (31.53 to 34.51)	-	-	-	-	-	-
	IMD=5	1337	48251.48	27.71 (26.22 to 29.19)	1972	55593	35.47 (33.91 to 37.04)	-	-	-	-	-	-
	IMD=6	1439	49636.77	28.99 (27.49 to 30.49)	2166	57342	37.77 (36.18 to 39.36)	-	-	-	-	-	-

	IMD=7	1510	54846.05	27.53 (26.14 to 28.92)	2262	63281	35.75 (34.27 to 37.22)	-	-	-	-	-	-
	IMD=8	1459	49831.01	29.28 (27.78 to 30.78)	2146	57217	37.51 (35.92 to 39.09)	-	-	-	-	-	-
	IMD=9	1597	53664.59	29.76 (28.30 to 31.22)	2474	62016	39.89 (38.32 to 41.46)	-	-	-	-	-	-
	Most Deprivation	1571	48153.51	32.62 (31.01 to 34.24)	2441	55298	44.14 (42.39 to 45.89)	-	-	-	-	-	-
2005	Least Deprivation	1346	49915.46	26.97 (25.52 to 28.41)	1897	57393	33.05 (31.57 to 34.54)	-	-	-	-	-	-
	IMD=2	1358	51879.12	26.18 (24.78 to 27.57)	1977	59782	33.07 (31.61 to 34.53)	-	-	-	-	-	-
	IMD=3	1309	51045.46	25.64 (24.25 to 27.03)	1954	58550	33.37 (31.89 to 34.85)	-	-	-	-	-	-
	IMD=4	1288	48675.83	26.46 (25.02 to 27.91)	1941	56434	34.39 (32.86 to 35.92)	-	-	-	-	-	-
	IMD=5	1291	48596.14	26.57 (25.12 to 28.02)	1990	56273	35.36 (33.81 to 36.92)	-	-	-	-	-	-
	IMD=6	1516	49453.57	30.66 (29.11 to 32.20)	2211	57506	38.45 (36.85 to 40.05)	-	-	-	-	-	-
	IMD=7	1524	54914.67	27.75 (26.36 to 29.15)	2291	63744	35.94 (34.47 to 37.41)	-	-	-	-	-	-
	IMD=8	1423	50072.8	28.42 (26.94 to 29.90)	2187	58092	37.65 (36.07 to 39.23)	-	-	-	-	-	-
	IMD=9	1624	54036.84	30.05 (28.59 to 31.52)	2574	63184	40.74 (39.16 to 42.31)	-	-	-	-	-	-
	Most Deprivation	1682	48546.77	34.65 (32.99 to 36.30)	2664	56788	46.91 (45.13 to 48.69)	-	-	-	-	-	-
2006	Least Deprivation	1298	49235.1	26.36 (24.93 to 27.80)	1918	56788	33.77 (32.26 to 35.29)	-	-	-	-	-	-
	IMD=2	1293	50953.07	25.38 (23.99 to 26.76)	1993	58968	33.80 (32.31 to 35.28)	-	-	-	-	-	-
	IMD=3	1315	50379.01	26.10 (24.69 to 27.51)	2004	58318	34.36 (32.86 to 35.87)	-	-	-	-	-	-
	IMD=4	1251	48118.59	26.00 (24.56 to 27.44)	1889	55980	33.74 (32.22 to 35.27)	-	-	-	-	-	-
	IMD=5	1338	48412.19	27.64 (26.16 to 29.12)	2017	56407	35.76 (34.20 to 37.32)	-	-	-	-	-	-
	IMD=6	1323	49167.81	26.91 (25.46 to 28.36)	2102	57769	36.39 (34.83 to 37.94)	-	-	-	-	-	-
	IMD=7	1519	54871.97	27.68 (26.29 to 29.07)	2332	64425	36.20 (34.73 to 37.67)	-	-	-	-	-	-
	IMD=8	1394	50222.8	27.76 (26.30 to 29.21)	2240	59079	37.92 (36.35 to 39.49)	-	-	-	-	-	-
	IMD=9	1734	55026.27	31.51 (30.03 to 33.00)	2694	64687	41.65 (40.07 to 43.22)	-	-	-	-	-	-
	Most Deprivation	1612	49576.02	32.52 (30.93 to 34.10)	2659	58562	45.40 (43.68 to 47.13)	-	-	-	-	-	-
2007	Least Deprivation	1213	48609.23	24.95 (23.55 to 26.36)	1833	56514	32.43 (30.95 to 33.92)	-	-	-	-	-	-
	IMD=2	1229	50373.48	24.40 (23.03 to 25.76)	1891	58508	32.32 (30.86 to 33.78)	-	-	-	-	-	-
	IMD=3	1262	49761.27	25.36 (23.96 to 26.76)	1916	57844	33.12 (31.64 to 34.61)	-	-	-	-	-	-
	IMD=4	1308	47706.05	27.42 (25.93 to 28.90)	2014	55876	36.04 (34.47 to 37.62)	-	-	-	-	-	-
	IMD=5	1265	48282.36	26.20 (24.76 to 27.64)	1985	56714	35.00 (33.46 to 36.54)	-	-	-	-	-	-

	IMD=6	1376	48995.8	28.08 (26.60 to 29.57)	2198	57764	38.05 (36.46 to 39.64)	-	-	-	-	-	-
	IMD=7	1539	54734.14	28.12 (26.71 to 29.52)	2381	64589	36.86 (35.38 to 38.34)	-	-	-	-	-	-
	IMD=8	1473	50660.16	29.08 (27.59 to 30.56)	2410	59667	40.39 (38.78 to 42.00)	-	-	-	-	-	-
	IMD=9	1740	55575.61	31.31 (29.84 to 32.78)	2797	66353	42.15 (40.59 to 43.72)	-	-	-	-	-	-
	Most Deprivation	1780	50264.51	35.41 (33.77 to 37.06)	2961	59929	49.41 (47.63 to 51.19)	-	-	-	-	-	-
2008	Least Deprivation	1233	48668.11	25.33 (23.92 to 26.75)	1848	56106	32.94 (31.44 to 34.44)	-	-	-	-	-	-
	IMD=2	1319	50653.82	26.04 (24.63 to 27.44)	1993	58582	34.02 (32.53 to 35.51)	-	-	-	-	-	-
	IMD=3	1311	50221.87	26.10 (24.69 to 27.52)	2032	58025	35.02 (33.50 to 36.54)	-	-	-	-	-	-
	IMD=4	1282	47844.26	26.80 (25.33 to 28.26)	1993	55832	35.70 (34.13 to 37.26)	-	-	-	-	-	-
	IMD=5	1345	48935.02	27.49 (26.02 to 28.95)	2084	57038	36.54 (34.97 to 38.11)	-	-	-	-	-	-
	IMD=6	1435	49837.19	28.79 (27.30 to 30.28)	2222	58511	37.98 (36.40 to 39.55)	-	-	-	-	-	-
	IMD=7	1581	56095.88	28.18 (26.79 to 29.57)	2482	65865	37.68 (36.20 to 39.17)	-	-	-	-	-	-
	IMD=8	1415	51847.37	27.29 (25.87 to 28.71)	2335	61198	38.15 (36.61 to 39.70)	-	-	-	-	-	-
	IMD=9	1771	57016.05	31.06 (29.61 to 32.51)	2933	67871	43.21 (41.65 to 44.78)	-	-	-	-	-	-
	Most Deprivation	1802	51059.98	35.29 (33.66 to 36.92)	3056	61277	49.87 (48.10 to 51.64)	-	-	-	-	-	-
2009	Least Deprivation	1275	48839.89	26.11 (24.67 to 27.54)	1891	56334	33.57 (32.05 to 35.08)	-	-	-	-	-	-
	IMD=2	1321	50654.12	26.08 (24.67 to 27.49)	2046	58757	34.82 (33.31 to 36.33)	-	-	-	-	-	-
	IMD=3	1325	50360.96	26.31 (24.89 to 27.73)	2079	58587	35.49 (33.96 to 37.01)	-	-	-	-	-	-
	IMD=4	1415	48415.62	29.23 (27.70 to 30.75)	2173	56652	38.36 (36.74 to 39.97)	-	-	-	-	-	-
	IMD=5	1394	49438.7	28.20 (26.72 to 29.68)	2183	57955	37.67 (36.09 to 39.25)	-	-	-	-	-	-
	IMD=6	1432	51099.09	28.02 (26.57 to 29.48)	2278	59809	38.09 (36.52 to 39.65)	-	-	-	-	-	-
	IMD=7	1703	57250.45	29.75 (28.33 to 31.16)	2741	67475	40.62 (39.10 to 42.14)	-	-	-	-	-	-
	IMD=8	1556	53110.64	29.30 (27.84 to 30.75)	2539	62716	40.48 (38.91 to 42.06)	-	-	-	-	-	-
	IMD=9	1912	58157.34	32.88 (31.40 to 34.35)	3107	69359	44.80 (43.22 to 46.37)	-	-	-	-	-	-
	Most Deprivation	1883	51974.79	36.23 (34.59 to 37.87)	3339	62561	53.37 (51.56 to 55.18)	-	-	-	-	-	-
2010	Least Deprivation	1232	49813.24	24.73 (23.35 to 26.11)	1837	57354	32.03 (30.56 to 33.49)	-	-	-	-	-	-
	IMD=2	1406	51671.13	27.21 (25.79 to 28.63)	2127	60066	35.41 (33.91 to 36.92)	-	-	-	-	-	-
	IMD=3	1410	51447.93	27.41 (25.98 to 28.84)	2145	60038	35.73 (34.22 to 37.24)	-	-	-	-	-	-
	IMD=4	1334	49299.59	27.06 (25.61 to 28.51)	2085	57883	36.02 (34.47 to 37.57)	-	-	-	-	-	-

	IMD=5	1377	50807.81	27.10 (25.67 to 28.53)	2202	59843	36.80 (35.26 to 38.33)	-	-	-	-	-	-
	IMD=6	1463	53176.55	27.51 (26.10 to 28.92)	2332	62572	37.27 (35.76 to 38.78)	-	-	-	-	-	-
	IMD=7	1727	59396.05	29.08 (27.70 to 30.45)	2788	70384	39.61 (38.14 to 41.08)	-	-	-	-	-	-
	IMD=8	1685	55628.85	30.29 (28.84 to 31.74)	2708	66031	41.01 (39.47 to 42.56)	-	-	-	-	-	-
	IMD=9	1957	60996.29	32.08 (30.66 to 33.51)	3315	72913	45.47 (43.92 to 47.01)	-	-	-	-	-	-
	Most Deprivation	2021	53550.12	37.74 (36.09 to 39.39)	3468	64984	53.37 (51.59 to 55.14)	-	-	-	-	-	-
2011	Least Deprivation	1271	50373.81	25.23 (23.84 to 26.62)	1921	58030	33.10 (31.62 to 34.58)	-	-	-	-	-	-
	IMD=2	1347	52245.91	25.78 (24.41 to 27.16)	2156	60624	35.56 (34.06 to 37.06)	-	-	-	-	-	-
	IMD=3	1422	52528.7	27.07 (25.66 to 28.48)	2204	61419	35.88 (34.39 to 37.38)	-	-	-	-	-	-
	IMD=4	1392	50259.41	27.70 (26.24 to 29.15)	2206	59218	37.25 (35.70 to 38.81)	-	-	-	-	-	-
	IMD=5	1445	51628.07	27.99 (26.55 to 29.43)	2260	61123	36.97 (35.45 to 38.50)	-	-	-	-	-	-
	IMD=6	1520	54444.74	27.92 (26.51 to 29.32)	2478	64450	38.45 (36.93 to 39.96)	-	-	-	-	-	-
	IMD=7	1730	61079.28	28.32 (26.99 to 29.66)	2844	72892	39.02 (37.58 to 40.45)	-	-	-	-	-	-
	IMD=8	1734	57176.15	30.33 (28.90 to 31.75)	2883	68397	42.15 (40.61 to 43.69)	-	-	-	-	-	-
	IMD=9	2076	62520.76	33.20 (31.78 to 34.63)	3512	75707	46.39 (44.86 to 47.92)	-	-	-	-	-	-
	Most Deprivation	2005	54874.11	36.54 (34.94 to 38.14)	3588	66853	53.67 (51.91 to 55.43)	-	-	-	-	-	-
2012	Least Deprivation	1270	50642.35	25.08 (23.70 to 26.46)	1906	58278	32.71 (31.24 to 34.17)	-	-	-	-	-	-
	IMD=2	1407	52950.72	26.57 (25.18 to 27.96)	2173	61469	35.35 (33.86 to 36.84)	-	-	-	-	-	-
	IMD=3	1438	53344.15	26.96 (25.56 to 28.35)	2291	62274	36.79 (35.28 to 38.30)	-	-	-	-	-	-
	IMD=4	1440	51330.93	28.05 (26.60 to 29.50)	2329	60343	38.60 (37.03 to 40.16)	-	-	-	-	-	-
	IMD=5	1508	53052.27	28.42 (26.99 to 29.86)	2404	62347	38.56 (37.02 to 40.10)	-	-	-	-	-	-
	IMD=6	1703	55997.5	30.41 (28.97 to 31.86)	2695	65829	40.94 (39.39 to 42.49)	-	-	-	-	-	-
	IMD=7	1879	62850.36	29.90 (28.54 to 31.25)	3050	74370	41.01 (39.56 to 42.47)	-	-	-	-	-	-
	IMD=8	1890	59658.22	31.68 (30.25 to 33.11)	3079	70732	43.53 (41.99 to 45.07)	-	-	-	-	-	-
	IMD=9	2241	64776.17	34.60 (33.16 to 36.03)	3785	77729	48.69 (47.14 to 50.25)	-	-	-	-	-	-
	Most Deprivation	2179	56902.04	38.29 (36.69 to 39.90)	3843	69019	55.68 (53.92 to 57.44)	-	-	-	-	-	-
2013	Least Deprivation	1304	50880.53	25.63 (24.24 to 27.02)	1987	58919	33.72 (32.24 to 35.21)	-	-	-	-	-	-
	IMD=2	1470	53293.37	27.58 (26.17 to 28.99)	2258	62531	36.11 (34.62 to 37.60)	-	-	-	-	-	-
	IMD=3	1484	53765.02	27.60 (26.20 to 29.01)	2352	63409	37.09 (35.59 to 38.59)	-	-	-	-	-	-

	IMD=4	1402	52011.93	26.96 (25.54 to 28.37)	2221	61560	36.08 (34.58 to 37.58)	-	-	-	-	-	-
	IMD=5	1531	53806.63	28.45 (27.03 to 29.88)	2398	64025	37.45 (35.96 to 38.95)	-	-	-	-	-	-
	IMD=6	1733	56423.56	30.71 (29.27 to 32.16)	2768	67846	40.80 (39.28 to 42.32)	-	-	-	-	-	-
	IMD=7	1898	63169.63	30.05 (28.69 to 31.40)	3081	76681	40.18 (38.76 to 41.60)	-	-	-	-	-	-
	IMD=8	1921	60306.5	31.85 (30.43 to 33.28)	3237	73708	43.92 (42.40 to 45.43)	-	-	-	-	-	-
	IMD=9	2283	64626.64	35.33 (33.88 to 36.78)	3899	80067	48.70 (47.17 to 50.23)	-	-	-	-	-	-
	Most Deprivation	2288	57540.49	39.76 (38.13 to 41.39)	4025	71040	56.66 (54.91 to 58.41)	-	-	-	-	-	-
2014	Least Deprivation	1282	50443.44	25.41 (24.02 to 26.81)	1950	58401	33.39 (31.91 to 34.87)	-	-	-	-	-	-
	IMD=2	1375	53253.85	25.82 (24.45 to 27.18)	2135	62415	34.21 (32.76 to 35.66)	-	-	-	-	-	-
	IMD=3	1425	53796.78	26.49 (25.11 to 27.86)	2248	63440	35.44 (33.97 to 36.90)	-	-	-	-	-	-
	IMD=4	1413	52129.09	27.11 (25.69 to 28.52)	2226	61570	36.15 (34.65 to 37.66)	-	-	-	-	-	-
	IMD=5	1523	54331.54	28.03 (26.62 to 29.44)	2445	64206	38.08 (36.57 to 39.59)	-	-	-	-	-	-
	IMD=6	1792	57455.14	31.19 (29.75 to 32.63)	2820	68331	41.27 (39.75 to 42.79)	-	-	-	-	-	-
	IMD=7	1956	64216.62	30.46 (29.11 to 31.81)	3122	76890	40.60 (39.18 to 42.03)	-	-	-	-	-	-
	IMD=8	2036	61392.75	33.16 (31.72 to 34.60)	3373	73755	45.73 (44.19 to 47.28)	-	-	-	-	-	-
	IMD=9	2255	66078.68	34.13 (32.72 to 35.53)	3843	80157	47.94 (46.43 to 49.46)	-	-	-	-	-	-
	Most Deprivation	2283	58538.42	39.00 (37.40 to 40.60)	4036	71514	56.44 (54.70 to 58.18)	-	-	-	-	-	-
2015	Least Deprivation	1234	50236.11	24.56 (23.19 to 25.93)	1806	57883	31.20 (29.76 to 32.64)	-	-	-	-	-	-
	IMD=2	1328	53653.04	24.75 (23.42 to 26.08)	2058	62385	32.99 (31.56 to 34.41)	-	-	-	-	-	-
	IMD=3	1374	54262.63	25.32 (23.98 to 26.66)	2212	63442	34.87 (33.41 to 36.32)	-	-	-	-	-	-
	IMD=4	1379	52714.71	26.16 (24.78 to 27.54)	2140	61452	34.82 (33.35 to 36.30)	-	-	-	-	-	-
	IMD=5	1460	55036.7	26.53 (25.17 to 27.89)	2369	64620	36.66 (35.18 to 38.14)	-	-	-	-	-	-
	IMD=6	1694	58608.78	28.90 (27.53 to 30.28)	2715	69309	39.17 (37.70 to 40.65)	-	-	-	-	-	-
	IMD=7	2035	65425.91	31.10 (29.75 to 32.46)	3282	77822	42.17 (40.73 to 43.62)	-	-	-	-	-	-
	IMD=8	2020	63441.05	31.84 (30.45 to 33.23)	3351	75786	44.22 (42.72 to 45.71)	-	-	-	-	-	-
	IMD=9	2364	68191.5	34.67 (33.27 to 36.06)	4058	82327	49.29 (47.77 to 50.81)	-	-	-	-	-	-
	Most Deprivation	2419	60352.45	40.08 (38.48 to 41.68)	4253	73281	58.04 (56.29 to 59.78)	-	-	-	-	-	-
2016	Least Deprivation	1066	50832.96	20.97 (19.71 to 22.23)	1613	58395	27.62 (26.27 to 28.97)	-	-	-	-	-	-
	IMD=2	1262	54788.64	23.03 (21.76 to 24.30)	1956	63412	30.85 (29.48 to 32.21)	-	-	-	-	-	-

	IMD=3	1367	55488.69	24.64 (23.33 to 25.94)	2127	64300	33.08 (31.67 to 34.49)	-	-	-	-	-	-
	IMD=4	1393	54100.01	25.75 (24.40 to 27.10)	2130	62772	33.93 (32.49 to 35.37)	-	-	-	-	-	-
	IMD=5	1420	56378.91	25.19 (23.88 to 26.50)	2277	65655	34.68 (33.26 to 36.11)	-	-	-	-	-	-
	IMD=6	1630	60696.12	26.86 (25.55 to 28.16)	2693	71032	37.91 (36.48 to 39.34)	-	-	-	-	-	-
	IMD=7	1913	67689.45	28.26 (26.99 to 29.53)	3103	79951	38.81 (37.45 to 40.18)	-	-	-	-	-	-
	IMD=8	1933	65847.75	29.36 (28.05 to 30.66)	3268	77983	41.91 (40.47 to 43.34)	-	-	-	-	-	-
	IMD=9	2244	71390.22	31.43 (30.13 to 32.73)	3896	85221	45.72 (44.28 to 47.15)	-	-	-	-	-	-
	Most Deprivation	2372	62691.18	37.84 (36.31 to 39.36)	4235	75624	56.00 (54.31 to 57.69)	-	-	-	-	-	-
2017	Least Deprivation	1106	51685.96	21.40 (20.14 to 22.66)	1651	59236	27.87 (26.53 to 29.22)	-	-	-	-	-	-
	IMD=2	1288	55833.61	23.07 (21.81 to 24.33)	1978	64357	30.73 (29.38 to 32.09)	-	-	-	-	-	-
	IMD=3	1353	57061.73	23.71 (22.45 to 24.97)	2046	65838	31.08 (29.73 to 32.42)	-	-	-	-	-	-
	IMD=4	1280	55235.11	23.17 (21.90 to 24.44)	1996	63806	31.28 (29.91 to 32.65)	-	-	-	-	-	-
	IMD=5	1409	57596.54	24.46 (23.19 to 25.74)	2224	66599	33.39 (32.01 to 34.78)	-	-	-	-	-	-
	IMD=6	1609	62452.08	25.76 (24.50 to 27.02)	2541	72767	34.92 (33.56 to 36.28)	-	-	-	-	-	-
	IMD=7	1857	70278.09	26.42 (25.22 to 27.63)	3015	82596	36.50 (35.20 to 37.81)	-	-	-	-	-	-
	IMD=8	1869	68119.31	27.44 (26.19 to 28.68)	3105	80448	38.60 (37.24 to 39.95)	-	-	-	-	-	-
	IMD=9	2286	74554.55	30.66 (29.41 to 31.92)	3884	88657	43.81 (42.43 to 45.19)	-	-	-	-	-	-
	Most Deprivation	2395	64916.68	36.89 (35.42 to 38.37)	4232	78432	53.96 (52.33 to 55.58)	-	-	-	-	-	-
2018	Least Deprivation	1048	52923.19	19.80 (18.60 to 21.00)	1541	60324	25.55 (24.27 to 26.82)	-	-	-	-	-	-
	IMD=2	1223	57703.3	21.19 (20.01 to 22.38)	1865	66457	28.06 (26.79 to 29.34)	-	-	-	-	-	-
	IMD=3	1339	59529.32	22.49 (21.29 to 23.70)	2028	68551	29.58 (28.30 to 30.87)	-	-	-	-	-	-
	IMD=4	1262	57675.74	21.88 (20.67 to 23.09)	1961	66442	29.51 (28.21 to 30.82)	-	-	-	-	-	-
	IMD=5	1357	59896.55	22.66 (21.45 to 23.86)	2115	69125	30.60 (29.29 to 31.90)	-	-	-	-	-	-
	IMD=6	1642	64957.55	25.28 (24.06 to 26.50)	2550	75407	33.82 (32.50 to 35.13)	-	-	-	-	-	-
	IMD=7	1704	73975.84	23.03 (21.94 to 24.13)	2775	86691	32.01 (30.82 to 33.20)	-	-	-	-	-	-
	IMD=8	1881	71230.05	26.41 (25.21 to 27.60)	3134	84010	37.31 (36.00 to 38.61)	-	-	-	-	-	-
	IMD=9	2289	78291.57	29.24 (28.04 to 30.43)	3849	92848	41.45 (40.15 to 42.76)	-	-	-	-	-	-
	Most Deprivation	2412	67169.39	35.91 (34.48 to 37.34)	4220	81301	51.91 (50.34 to 53.47)	-	-	-	-	-	-
2019	Least Deprivation	1021	53511.13	19.08 (17.91 to 20.25)	1472	61480	23.94 (22.72 to 25.17)	-	-	-	-	-	-

	IMD=2	1189	58360.16	20.37 (19.22 to 21.53)	1799	68091	26.42 (25.20 to 27.64)	-	-	-	-	-	-	
	IMD=3	1246	59426.03	20.97 (19.80 to 22.13)	1854	70203	26.41 (25.21 to 27.61)	-	-	-	-	-	-	
	IMD=4	1322	58026.67	22.78 (21.55 to 24.01)	1936	68479	28.27 (27.01 to 29.53)	-	-	-	-	-	-	
	IMD=5	1309	60701.71	21.56 (20.40 to 22.73)	2025	71057	28.50 (27.26 to 29.74)	-	-	-	-	-	-	
	IMD=6	1492	65673.66	22.72 (21.57 to 23.87)	2376	77129	30.81 (29.57 to 32.04)	-	-	-	-	-	-	
	IMD=7	1761	76209.51	23.11 (22.03 to 24.19)	2758	90045	30.63 (29.49 to 31.77)	-	-	-	-	-	-	
	IMD=8	1735	71655.09	24.21 (23.07 to 25.35)	2838	86166	32.94 (31.72 to 34.15)	-	-	-	-	-	-	
	IMD=9	2134	79909.2	26.71 (25.57 to 27.84)	3538	95139	37.19 (35.96 to 38.41)	-	-	-	-	-	-	
	Most Deprivation	2081	65611.47	31.72 (30.35 to 33.08)	3729	80136	46.53 (45.04 to 48.03)	-	-	-	-	-	-	
**35-44 years**														
2004	Least Deprivation	2823	92747.93	30.44 (29.31 to 31.56)	4224	105977	39.86 (38.66 to 41.06)	-	-	-	-	-	-	
	IMD=2	2788	85565.43	32.58 (31.37 to 33.79)	4168	98256	42.42 (41.13 to 43.71)	-	-	-	-	-	-	
	IMD=3	2684	81435.25	32.96 (31.71 to 34.21)	3982	93289	42.68 (41.36 to 44.01)	-	-	-	-	-	-	
	IMD=4	2485	74384.85	33.41 (32.09 to 34.72)	3784	85917	44.04 (42.64 to 45.45)	-	-	-	-	-	-	
	IMD=5	2415	71429.38	33.81 (32.46 to 35.16)	3770	82612	45.64 (44.18 to 47.09)	-	-	-	-	-	-	
	IMD=6	2471	71175.57	34.72 (33.35 to 36.09)	3894	82496	47.20 (45.72 to 48.68)	-	-	-	-	-	-	
	IMD=7	2553	72385.84	35.27 (33.90 to 36.64)	4110	84324	48.74 (47.25 to 50.23)	-	-	-	-	-	-	
	IMD=8	2325	63527.03	36.60 (35.11 to 38.09)	3785	74183	51.02 (49.40 to 52.65)	-	-	-	-	-	-	
	IMD=9	2590	67868.55	38.16 (36.69 to 39.63)	4253	79473	53.52 (51.91 to 55.12)	-	-	-	-	-	-	
	Most Deprivation	2289	58131.3	39.38 (37.76 to 40.99)	3832	68045	56.32 (54.53 to 58.10)	-	-	-	-	-	-	
2005	Least Deprivation	2882	92932.2	31.01 (29.88 to 32.14)	4444	106934	41.56 (40.34 to 42.78)	-	-	-	-	-	-	
	IMD=2	2667	85089.81	31.34 (30.15 to 32.53)	4139	98291	42.11 (40.83 to 43.39)	-	-	-	-	-	-	
	IMD=3	2642	81729.4	32.33 (31.09 to 33.56)	4064	94429	43.04 (41.71 to 44.36)	-	-	-	-	-	-	
	IMD=4	2483	74285.94	33.42 (32.11 to 34.74)	3902	86337	45.19 (43.78 to 46.61)	-	-	-	-	-	-	
	IMD=5	2386	71842.62	33.21 (31.88 to 34.54)	3861	83589	46.19 (44.73 to 47.65)	-	-	-	-	-	-	
	IMD=6	2406	71268.66	33.76 (32.41 to 35.11)	4010	83592	47.97 (46.49 to 49.46)	-	-	-	-	-	-	
	IMD=7	2546	72846.91	34.95 (33.59 to 36.31)	4251	85965	49.45 (47.96 to 50.94)	-	-	-	-	-	-	
	IMD=8	2403	63587.77	37.79 (36.28 to 39.30)	3971	75422	52.65 (51.01 to 54.29)	-	-	-	-	-	-	
	IMD=9	2581	67705.38	38.12 (36.65 to 39.59)	4480	80755	55.48 (53.85 to 57.10)	-	-	-	-	-	-	

	Most Deprivation	2401	58604.84	40.97 (39.33 to 42.61)	4180	69960	59.75 (57.94 to 61.56)	-	-	-	-	-	-
2006	Least Deprivation	2842	93729.55	30.32 (29.21 to 31.44)	4471	108325	41.27 (40.06 to 42.48)	-	-	-	-	-	-
	IMD=2	2782	85508.98	32.53 (31.33 to 33.74)	4348	99339	43.77 (42.47 to 45.07)	-	-	-	-	-	-
	IMD=3	2624	82204	31.92 (30.70 to 33.14)	4179	95530	43.75 (42.42 to 45.07)	-	-	-	-	-	-
	IMD=4	2537	74947.84	33.85 (32.53 to 35.17)	4081	87846	46.46 (45.03 to 47.88)	-	-	-	-	-	-
	IMD=5	2401	72655.98	33.05 (31.72 to 34.37)	3911	85244	45.88 (44.44 to 47.32)	-	-	-	-	-	-
	IMD=6	2461	71864.95	34.24 (32.89 to 35.60)	4110	85404	48.12 (46.65 to 49.60)	-	-	-	-	-	-
	IMD=7	2523	73529.6	34.31 (32.97 to 35.65)	4297	87542	49.09 (47.62 to 50.55)	-	-	-	-	-	-
	IMD=8	2296	64115.19	35.81 (34.35 to 37.28)	4021	76906	52.28 (50.67 to 53.90)	-	-	-	-	-	-
	IMD=9	2614	68364.55	38.24 (36.77 to 39.70)	4676	82314	56.81 (55.18 to 58.44)	-	-	-	-	-	-
	Most Deprivation	2467	59347.84	41.57 (39.93 to 43.21)	4435	71834	61.74 (59.92 to 63.56)	-	-	-	-	-	-
2007	Least Deprivation	2783	93237.59	29.85 (28.74 to 30.96)	4426	108212	40.90 (39.70 to 42.11)	-	-	-	-	-	-
	IMD=2	2772	85337.57	32.48 (31.27 to 33.69)	4421	99555	44.41 (43.10 to 45.72)	-	-	-	-	-	-
	IMD=3	2680	81154.66	33.02 (31.77 to 34.27)	4283	95130	45.02 (43.67 to 46.37)	-	-	-	-	-	-
	IMD=4	2483	74391.78	33.38 (32.06 to 34.69)	4061	87742	46.28 (44.86 to 47.71)	-	-	-	-	-	-
	IMD=5	2393	72189.32	33.15 (31.82 to 34.48)	3996	85274	46.86 (45.41 to 48.31)	-	-	-	-	-	-
	IMD=6	2534	71324.09	35.53 (34.14 to 36.91)	4297	84999	50.55 (49.04 to 52.07)	-	-	-	-	-	-
	IMD=7	2486	73514.09	33.82 (32.49 to 35.15)	4329	87692	49.37 (47.90 to 50.84)	-	-	-	-	-	-
	IMD=8	2356	64494.24	36.53 (35.06 to 38.01)	4126	77509	53.23 (51.61 to 54.86)	-	-	-	-	-	-
	IMD=9	2715	68540.05	39.61 (38.12 to 41.10)	4841	83330	58.09 (56.46 to 59.73)	-	-	-	-	-	-
	Most Deprivation	2530	59348.74	42.63 (40.97 to 44.29)	4683	72536	64.56 (62.71 to 66.41)	-	-	-	-	-	-
2008	Least Deprivation	2862	93145.84	30.73 (29.60 to 31.85)	4513	107414	42.02 (40.79 to 43.24)	-	-	-	-	-	-
	IMD=2	2736	85182.33	32.12 (30.92 to 33.32)	4342	99178	43.78 (42.48 to 45.08)	-	-	-	-	-	-
	IMD=3	2606	80927.73	32.20 (30.97 to 33.44)	4289	94457	45.41 (44.05 to 46.77)	-	-	-	-	-	-
	IMD=4	2426	74187.75	32.70 (31.40 to 34.00)	4068	87254	46.62 (45.19 to 48.06)	-	-	-	-	-	-
	IMD=5	2396	72152.24	33.21 (31.88 to 34.54)	4059	85038	47.73 (46.26 to 49.20)	-	-	-	-	-	-
	IMD=6	2440	71279.56	34.23 (32.87 to 35.59)	4235	84834	49.92 (48.42 to 51.42)	-	-	-	-	-	-
	IMD=7	2567	73773.4	34.80 (33.45 to 36.14)	4417	87619	50.41 (48.92 to 51.90)	-	-	-	-	-	-
	IMD=8	2314	65021.45	35.59 (34.14 to 37.04)	4126	78245	52.73 (51.12 to 54.34)	-	-	-	-	-	-
	IMD=9	2733	68604.57	39.84 (38.34 to 41.33)	5021	83539	60.10 (58.44 to 61.77)	-	-	-	-	-	-

	Most Deprivation	2520	59048.13	42.68 (41.01 to 44.34)	4795	72821	65.85 (63.98 to 67.71)	-	-	-	-	-	-
2009	Least Deprivation	2823	91833.95	30.74 (29.61 to 31.87)	4479	106276	42.14 (40.91 to 43.38)	-	-	-	-	-	-
	IMD=2	2731	83420.35	32.74 (31.51 to 33.97)	4426	97675	45.31 (43.98 to 46.65)	-	-	-	-	-	-
	IMD=3	2682	80233.26	33.43 (32.16 to 34.69)	4409	93930	46.94 (45.55 to 48.32)	-	-	-	-	-	-
	IMD=4	2458	73130.56	33.61 (32.28 to 34.94)	4175	86165	48.45 (46.98 to 49.92)	-	-	-	-	-	-
	IMD=5	2388	71551.31	33.37 (32.04 to 34.71)	4062	84406	48.12 (46.64 to 49.60)	-	-	-	-	-	-
	IMD=6	2534	70662.88	35.86 (34.46 to 37.26)	4412	84068	52.48 (50.93 to 54.03)	-	-	-	-	-	-
	IMD=7	2585	73483.06	35.18 (33.82 to 36.53)	4521	87462	51.69 (50.18 to 53.20)	-	-	-	-	-	-
	IMD=8	2423	64579.89	37.52 (36.03 to 39.01)	4379	77715	56.35 (54.68 to 58.02)	-	-	-	-	-	-
	IMD=9	2762	68369.55	40.40 (38.89 to 41.90)	5168	83473	61.91 (60.22 to 63.60)	-	-	-	-	-	-
	Most Deprivation	2648	57848.15	45.78 (44.03 to 47.52)	5086	71778	70.86 (68.91 to 72.80)	-	-	-	-	-	-
2010	Least Deprivation	2890	91386.45	31.62 (30.47 to 32.78)	4539	106034	42.81 (41.56 to 44.05)	-	-	-	-	-	-
	IMD=2	2731	82620.91	33.05 (31.81 to 34.29)	4492	96874	46.37 (45.01 to 47.73)	-	-	-	-	-	-
	IMD=3	2722	79389.21	34.29 (33.00 to 35.57)	4510	93317	48.33 (46.92 to 49.74)	-	-	-	-	-	-
	IMD=4	2393	72561.32	32.98 (31.66 to 34.30)	4051	85715	47.26 (45.81 to 48.72)	-	-	-	-	-	-
	IMD=5	2363	71293.82	33.14 (31.81 to 34.48)	4145	84484	49.06 (47.57 to 50.56)	-	-	-	-	-	-
	IMD=6	2546	70439.38	36.14 (34.74 to 37.55)	4433	84384	52.53 (50.99 to 54.08)	-	-	-	-	-	-
	IMD=7	2537	73474.77	34.53 (33.19 to 35.87)	4605	87912	52.38 (50.87 to 53.89)	-	-	-	-	-	-
	IMD=8	2449	64720.86	37.84 (36.34 to 39.34)	4467	78342	57.02 (55.35 to 58.69)	-	-	-	-	-	-
	IMD=9	2685	68424.52	39.24 (37.76 to 40.72)	5135	83948	61.17 (59.50 to 62.84)	-	-	-	-	-	-
	Most Deprivation	2597	57166.24	45.43 (43.68 to 47.18)	5162	71667	72.03 (70.06 to 73.99)	-	-	-	-	-	-
2011	Least Deprivation	2894	90217.62	32.08 (30.91 to 33.25)	4641	105142	44.14 (42.87 to 45.41)	-	-	-	-	-	-
	IMD=2	2744	81514.96	33.66 (32.40 to 34.92)	4508	95815	47.05 (45.68 to 48.42)	-	-	-	-	-	-
	IMD=3	2596	78317.16	33.15 (31.87 to 34.42)	4349	92360	47.09 (45.69 to 48.49)	-	-	-	-	-	-
	IMD=4	2415	71260.74	33.89 (32.54 to 35.24)	4079	84609	48.21 (46.73 to 49.69)	-	-	-	-	-	-
	IMD=5	2335	70265.61	33.23 (31.88 to 34.58)	4058	83787	48.43 (46.94 to 49.92)	-	-	-	-	-	-
	IMD=6	2590	69794.87	37.11 (35.68 to 38.54)	4451	83916	53.04 (51.48 to 54.60)	-	-	-	-	-	-
	IMD=7	2637	72797.36	36.22 (34.84 to 37.61)	4768	87871	54.26 (52.72 to 55.80)	-	-	-	-	-	-
	IMD=8	2438	64782.24	37.63 (36.14 to 39.13)	4457	78888	56.50 (54.84 to 58.16)	-	-	-	-	-	-
	IMD=9	2779	67772.56	41.00 (39.48 to 42.53)	5313	83771	63.42 (61.72 to 65.13)	-	-	-	-	-	-

	Most Deprivation	2632	56479.55	46.60 (44.82 to 48.38)	5255	71225	73.78 (71.79 to 75.78)	-	-	-	-	-	-
2012	Least Deprivation	2755	87654.26	31.43 (30.26 to 32.60)	4424	102019	43.36 (42.09 to 44.64)	-	-	-	-	-	-
	IMD=2	2564	79703.55	32.17 (30.92 to 33.41)	4262	93493	45.59 (44.22 to 46.95)	-	-	-	-	-	-
	IMD=3	2482	76508.6	32.44 (31.16 to 33.72)	4202	90072	46.65 (45.24 to 48.06)	-	-	-	-	-	-
	IMD=4	2429	69665.75	34.87 (33.48 to 36.25)	4067	82407	49.35 (47.84 to 50.87)	-	-	-	-	-	-
	IMD=5	2357	68736.95	34.29 (32.91 to 35.67)	4056	81580	49.72 (48.19 to 51.25)	-	-	-	-	-	-
	IMD=6	2470	68440.5	36.09 (34.67 to 37.51)	4424	82231	53.80 (52.21 to 55.39)	-	-	-	-	-	-
	IMD=7	2649	71744.39	36.92 (35.52 to 38.33)	4799	86402	55.54 (53.97 to 57.11)	-	-	-	-	-	-
	IMD=8	2530	64630.83	39.15 (37.62 to 40.67)	4587	78441	58.48 (56.78 to 60.17)	-	-	-	-	-	-
	IMD=9	2923	67290.33	43.44 (41.86 to 45.01)	5526	83177	66.44 (64.68 to 68.19)	-	-	-	-	-	-
	Most Deprivation	2688	56071.92	47.94 (46.13 to 49.75)	5398	70639	76.42 (74.38 to 78.46)	-	-	-	-	-	-
2013	Least Deprivation	2629	85057.06	30.91 (29.73 to 32.09)	4228	99331	42.56 (41.28 to 43.85)	-	-	-	-	-	-
	IMD=2	2427	77319.72	31.39 (30.14 to 32.64)	4045	91364	44.27 (42.91 to 45.64)	-	-	-	-	-	-
	IMD=3	2511	74069.65	33.90 (32.57 to 35.23)	4182	87907	47.57 (46.13 to 49.01)	-	-	-	-	-	-
	IMD=4	2288	67910.35	33.69 (32.31 to 35.07)	3915	81130	48.26 (46.74 to 49.77)	-	-	-	-	-	-
	IMD=5	2281	66692.34	34.20 (32.80 to 35.61)	3871	80201	48.27 (46.75 to 49.79)	-	-	-	-	-	-
	IMD=6	2471	65914.04	37.49 (36.01 to 38.97)	4424	80930	54.66 (53.05 to 56.28)	-	-	-	-	-	-
	IMD=7	2551	69103.7	36.92 (35.48 to 38.35)	4651	85589	54.34 (52.78 to 55.90)	-	-	-	-	-	-
	IMD=8	2457	62426.64	39.36 (37.80 to 40.91)	4500	78574	57.27 (55.60 to 58.94)	-	-	-	-	-	-
	IMD=9	2778	64200.12	43.27 (41.66 to 44.88)	5375	82670	65.02 (63.28 to 66.76)	-	-	-	-	-	-
	Most Deprivation	2583	54325.18	47.55 (45.71 to 49.38)	5418	70309	77.06 (75.01 to 79.11)	-	-	-	-	-	-
2014	Least Deprivation	2528	83129.33	30.41 (29.22 to 31.60)	4020	96985	41.45 (40.17 to 42.73)	-	-	-	-	-	-
	IMD=2	2514	75813.31	33.16 (31.86 to 34.46)	4026	89342	45.06 (43.67 to 46.45)	-	-	-	-	-	-
	IMD=3	2392	72618.88	32.94 (31.62 to 34.26)	4009	86328	46.44 (45.00 to 47.88)	-	-	-	-	-	-
	IMD=4	2198	66654.98	32.98 (31.60 to 34.35)	3768	79627	47.32 (45.81 to 48.83)	-	-	-	-	-	-
	IMD=5	2289	65888.02	34.74 (33.32 to 36.16)	3942	78795	50.03 (48.47 to 51.59)	-	-	-	-	-	-
	IMD=6	2426	65049.09	37.29 (35.81 to 38.78)	4315	79144	54.52 (52.89 to 56.15)	-	-	-	-	-	-
	IMD=7	2562	68315.06	37.50 (36.05 to 38.95)	4610	83350	55.31 (53.71 to 56.91)	-	-	-	-	-	-
	IMD=8	2518	62396.25	40.35 (38.78 to 41.93)	4587	77167	59.44 (57.72 to 61.16)	-	-	-	-	-	-
	IMD=9	2806	64025.11	43.83 (42.20 to 45.45)	5340	80394	66.42 (64.64 to 68.20)	-	-	-	-	-	-

	Most Deprivation	2515	53787.12	46.76 (44.93 to 48.59)	5254	68809	76.36 (74.29 to 78.42)	-	-	-	-	-	-
2015	Least Deprivation	2427	82088.35	29.57 (28.39 to 30.74)	3834	95200	40.27 (39.00 to 41.55)	-	-	-	-	-	-
	IMD=2	2399	74417.22	32.24 (30.95 to 33.53)	3946	87496	45.10 (43.69 to 46.51)	-	-	-	-	-	-
	IMD=3	2315	71558.12	32.35 (31.03 to 33.67)	3840	84629	45.37 (43.94 to 46.81)	-	-	-	-	-	-
	IMD=4	2119	66093.75	32.06 (30.70 to 33.43)	3606	78246	46.09 (44.58 to 47.59)	-	-	-	-	-	-
	IMD=5	2163	65317.82	33.12 (31.72 to 34.51)	3718	78239	47.52 (45.99 to 49.05)	-	-	-	-	-	-
	IMD=6	2356	65267.21	36.10 (34.64 to 37.56)	4139	79011	52.39 (50.79 to 53.98)	-	-	-	-	-	-
	IMD=7	2578	68890.09	37.42 (35.98 to 38.87)	4626	83568	55.36 (53.76 to 56.95)	-	-	-	-	-	-
	IMD=8	2583	63394.59	40.74 (39.17 to 42.32)	4715	78019	60.43 (58.71 to 62.16)	-	-	-	-	-	-
	IMD=9	2895	65296.3	44.34 (42.72 to 45.95)	5426	81659	66.45 (64.68 to 68.22)	-	-	-	-	-	-
	Most Deprivation	2459	54760.41	44.90 (43.13 to 46.68)	5208	69590	74.84 (72.81 to 76.87)	-	-	-	-	-	-
2016	Least Deprivation	2343	80465.88	29.12 (27.94 to 30.30)	3674	92915	39.54 (38.26 to 40.82)	-	-	-	-	-	-
	IMD=2	2252	73684.04	30.56 (29.30 to 31.83)	3630	85998	42.21 (40.84 to 43.58)	-	-	-	-	-	-
	IMD=3	2258	71129.35	31.74 (30.44 to 33.05)	3729	83463	44.68 (43.24 to 46.11)	-	-	-	-	-	-
	IMD=4	2094	66373.95	31.55 (30.20 to 32.90)	3528	77976	45.24 (43.75 to 46.74)	-	-	-	-	-	-
	IMD=5	2034	65959.88	30.84 (29.50 to 32.18)	3484	77830	44.76 (43.28 to 46.25)	-	-	-	-	-	-
	IMD=6	2246	65883.96	34.09 (32.68 to 35.50)	3961	78874	50.22 (48.66 to 51.78)	-	-	-	-	-	-
	IMD=7	2442	70222.64	34.78 (33.40 to 36.15)	4422	84417	52.38 (50.84 to 53.93)	-	-	-	-	-	-
	IMD=8	2429	65498.61	37.08 (35.61 to 38.56)	4466	79837	55.94 (54.30 to 57.58)	-	-	-	-	-	-
	IMD=9	2729	67590.02	40.38 (38.86 to 41.89)	5254	83633	62.82 (61.12 to 64.52)	-	-	-	-	-	-
	Most Deprivation	2565	56663	45.27 (43.52 to 47.02)	5155	71308	72.29 (70.32 to 74.27)	-	-	-	-	-	-
2017	Least Deprivation	2146	79539.57	26.98 (25.84 to 28.12)	3369	91342	36.88 (35.64 to 38.13)	-	-	-	-	-	-
	IMD=2	2118	73455.02	28.83 (27.61 to 30.06)	3449	85357	40.41 (39.06 to 41.76)	-	-	-	-	-	-
	IMD=3	2075	71693.58	28.94 (27.70 to 30.19)	3417	83676	40.84 (39.47 to 42.21)	-	-	-	-	-	-
	IMD=4	2019	66796.91	30.23 (28.91 to 31.54)	3389	78216	43.33 (41.87 to 44.79)	-	-	-	-	-	-
	IMD=5	2033	66986.32	30.35 (29.03 to 31.67)	3400	78890	43.10 (41.65 to 44.55)	-	-	-	-	-	-
	IMD=6	2137	67512.37	31.65 (30.31 to 33.00)	3762	80386	46.80 (45.30 to 48.29)	-	-	-	-	-	-
	IMD=7	2337	72183.17	32.38 (31.06 to 33.69)	4149	86470	47.98 (46.52 to 49.44)	-	-	-	-	-	-
	IMD=8	2422	68207.8	35.51 (34.09 to 36.92)	4442	82657	53.74 (52.16 to 55.32)	-	-	-	-	-	-
	IMD=9	2681	70812.51	37.86 (36.43 to 39.29)	5121	86933	58.91 (57.29 to 60.52)	-	-	-	-	-	-

	Most Deprivation	2485	59035.34	42.09 (40.44 to 43.75)	5108	74059	68.97 (67.08 to 70.86)	-	-	-	-	-	-
2018	Least Deprivation	2037	79555.45	25.60 (24.49 to 26.72)	3108	90937	34.18 (32.98 to 35.38)	-	-	-	-	-	-
	IMD=2	1993	74588.88	26.72 (25.55 to 27.89)	3172	86464	36.69 (35.41 to 37.96)	-	-	-	-	-	-
	IMD=3	1999	73156.26	27.33 (26.13 to 28.52)	3238	84964	38.11 (36.80 to 39.42)	-	-	-	-	-	-
	IMD=4	1880	68796.76	27.33 (26.09 to 28.56)	3103	80192	38.69 (37.33 to 40.06)	-	-	-	-	-	-
	IMD=5	1969	69428.61	28.36 (27.11 to 29.61)	3344	81210	41.18 (39.78 to 42.57)	-	-	-	-	-	-
	IMD=6	2100	70062.21	29.97 (28.69 to 31.26)	3653	83044	43.99 (42.56 to 45.42)	-	-	-	-	-	-
	IMD=7	2377	75284.05	31.57 (30.30 to 32.84)	4221	89969	46.92 (45.50 to 48.33)	-	-	-	-	-	-
	IMD=8	2279	72063.62	31.62 (30.33 to 32.92)	4212	86545	48.67 (47.20 to 50.14)	-	-	-	-	-	-
	IMD=9	2765	74761.7	36.98 (35.61 to 38.36)	5121	91107	56.21 (54.67 to 57.75)	-	-	-	-	-	-
	Most Deprivation	2571	61738.21	41.64 (40.03 to 43.25)	5102	77341	65.97 (64.16 to 67.78)	-	-	-	-	-	-
2019	Least Deprivation	1875	78299.15	23.95 (22.86 to 25.03)	2866	90054	31.83 (30.66 to 32.99)	-	-	-	-	-	-
	IMD=2	1946	74260.65	26.21 (25.04 to 27.37)	3101	86844	35.71 (34.45 to 36.96)	-	-	-	-	-	-
	IMD=3	1922	72500.27	26.51 (25.33 to 27.70)	3019	85809	35.18 (33.93 to 36.44)	-	-	-	-	-	-
	IMD=4	1894	68730.83	27.56 (26.32 to 28.80)	3055	81595	37.44 (36.11 to 38.77)	-	-	-	-	-	-
	IMD=5	1908	70525.71	27.05 (25.84 to 28.27)	3150	83204	37.86 (36.54 to 39.18)	-	-	-	-	-	-
	IMD=6	2111	71557.39	29.50 (28.24 to 30.76)	3595	85100	42.24 (40.86 to 43.63)	-	-	-	-	-	-
	IMD=7	2220	76713.28	28.94 (27.74 to 30.14)	3901	92260	42.28 (40.96 to 43.61)	-	-	-	-	-	-
	IMD=8	2307	73436.27	31.42 (30.13 to 32.70)	4042	89772	45.03 (43.64 to 46.41)	-	-	-	-	-	-
	IMD=9	2649	76612.23	34.58 (33.26 to 35.89)	4861	93793	51.83 (50.37 to 53.28)	-	-	-	-	-	-
	Most Deprivation	2500	61106.68	40.91 (39.31 to 42.52)	4791	76933	62.27 (60.51 to 64.04)	-	-	-	-	-	-
**45-54 years**													
2004	Least Deprivation	3002	89110.65	33.69 (32.48 to 34.89)	4533	101942	44.47 (43.17 to 45.76)	622	98185.07	6.33 (5.84 to 6.83)	871	101942	8.54 (7.98 to 9.11)
	IMD=2	2870	81095.41	35.39 (34.10 to 36.69)	4238	93169	45.49 (44.12 to 46.86)	642	89258.73	7.19 (5.64 to 7.75)	933	93169	10.01 (9.37 to 10.66)
	IMD=3	2719	75849.52	35.85 (34.50 to 37.19)	4075	86940	46.87 (45.43 to 48.31)	624	83354.48	7.49 (5.90 to 8.07)	929	86940	10.69 (10.00 to 11.37)
	IMD=4	2343	67393.56	34.77 (33.36 to 36.17)	3613	77680	46.51 (44.99 to 48.03)	544	74402.05	7.31 (5.70 to 7.93)	799	77680	10.29 (9.57 to 11.00)
	IMD=5	2249	63832.15	35.23 (33.78 to 36.69)	3541	73500	48.18 (46.59 to 49.76)	624	70219.27	8.89 (5.19 to 9.58)	930	73500	12.65 (11.84 to 13.47)
	IMD=6	2294	61276.33	37.44 (35.90 to 38.97)	3567	71141	50.14 (48.49 to 51.79)	562	68069.13	8.26 (5.57 to 8.94)	862	71141	12.12 (11.31 to 12.93)
	IMD=7	2269	60086	37.76 (36.21 to 39.32)	3727	69995	53.25 (51.54 to 54.96)	578	66862.46	8.64 (5.94 to 9.35)	899	69995	12.84 (12.00 to 13.68)
	IMD=8	1913	49849.04	38.38 (36.66 to 40.10)	3174	58383	54.37 (52.47 to 56.26)	527	55532.48	9.49 (5.68 to 10.30)	839	58383	14.37 (13.40 to 15.34)

	IMD=9	2201	52187.43	42.17 (40.41 to 43.94)	3606	61200	58.92 (57.00 to 60.84)	534	58011.67	9.21 (5.42 to 9.99)	899	61200	14.69 (13.73 to 15.65)
	Most Deprivation	1982	45751.04	43.32 (41.41 to 45.23)	3411	53928	63.25 (61.13 to 65.37)	560	51152.73	10.95 (5.04 to 11.85)	900	53928	16.69 (15.60 to 17.78)
2005	Least Deprivation	3049	90002.05	33.88 (32.67 to 35.08)	4717	103927	45.39 (44.09 to 46.68)	571	100081.6	5.71 (5.24 to 6.17)	848	103927	8.16 (7.61 to 8.71)
	IMD=2	2858	81185.33	35.20 (33.91 to 36.49)	4416	94134	46.91 (45.53 to 48.30)	593	90388.23	6.56 (5.03 to 7.09)	868	94134	9.22 (8.61 to 9.83)
	IMD=3	2780	76329.93	36.42 (35.07 to 37.77)	4328	88669	48.81 (47.36 to 50.26)	580	85022.65	6.82 (5.27 to 7.38)	879	88669	9.91 (9.26 to 10.57)
	IMD=4	2462	67738.34	36.35 (34.91 to 37.78)	3919	78907	49.67 (48.11 to 51.22)	540	75605.77	7.14 (5.54 to 7.74)	848	78907	10.75 (10.02 to 11.47)
	IMD=5	2319	64425.21	36.00 (34.53 to 37.46)	3767	75081	50.17 (48.57 to 51.77)	574	71692.23	8.01 (5.35 to 8.66)	904	75081	12.04 (11.26 to 12.83)
	IMD=6	2330	61685.11	37.77 (36.24 to 39.31)	3842	72617	52.91 (51.23 to 54.58)	551	69285.15	7.95 (5.29 to 8.62)	825	72617	11.36 (10.59 to 12.14)
	IMD=7	2326	60852.57	38.22 (36.67 to 39.78)	3920	71886	54.53 (52.82 to 56.24)	548	68482.42	8.00 (5.33 to 8.67)	906	71886	12.60 (11.78 to 13.42)
	IMD=8	2034	50671.24	40.14 (38.40 to 41.89)	3461	60192	57.50 (55.58 to 59.41)	508	57148.79	8.89 (5.12 to 9.66)	826	60192	13.72 (12.79 to 14.66)
	IMD=9	2344	52939.01	44.28 (42.48 to 46.07)	4003	63523	63.02 (61.06 to 64.97)	577	59966.61	9.62 (5.84 to 10.41)	971	63523	15.29 (14.32 to 16.25)
	Most Deprivation	2131	46587.17	45.74 (43.80 to 47.68)	3750	56204	66.72 (64.59 to 68.86)	569	53011.78	10.73 (5.85 to 11.62)	965	56204	17.17 (16.09 to 18.25)
2006	Least Deprivation	3222	92017.84	35.01 (33.81 to 36.22)	5075	107030	47.42 (46.11 to 48.72)	625	103075.1	6.06 (5.59 to 6.54)	900	107030	8.41 (7.86 to 8.96)
	IMD=2	2883	82838.54	34.80 (33.53 to 36.07)	4569	96790	47.21 (45.84 to 48.57)	595	92990.47	6.40 (5.88 to 6.91)	880	96790	9.09 (8.49 to 9.69)
	IMD=3	2782	77747.13	35.78 (34.45 to 37.11)	4477	91105	49.14 (47.70 to 50.58)	634	87391.77	7.25 (5.69 to 7.82)	942	91105	10.34 (9.68 to 11.00)
	IMD=4	2432	68948.55	35.27 (33.87 to 36.67)	4026	81174	49.60 (48.07 to 51.13)	501	77645.7	6.45 (5.89 to 7.02)	800	81174	9.86 (9.17 to 10.54)
	IMD=5	2513	65874.4	38.15 (36.66 to 39.64)	4077	77663	52.50 (50.88 to 54.11)	501	74118.8	6.76 (5.17 to 7.35)	801	77663	10.31 (9.60 to 11.03)
	IMD=6	2376	63059.22	37.68 (36.16 to 39.19)	3980	75138	52.97 (51.32 to 54.61)	560	71364.92	7.85 (5.20 to 8.50)	870	75138	11.58 (10.81 to 12.35)
	IMD=7	2406	62075.69	38.76 (37.21 to 40.31)	4240	74671	56.78 (55.07 to 58.49)	554	70677.24	7.84 (5.19 to 8.49)	929	74671	12.44 (11.64 to 13.24)
	IMD=8	2109	51523.48	40.93 (39.19 to 42.68)	3750	62286	60.21 (58.28 to 62.13)	486	58776.22	8.27 (5.53 to 9.00)	841	62286	13.50 (12.59 to 14.41)
	IMD=9	2304	54015.26	42.65 (40.91 to 44.40)	4266	65929	64.71 (62.76 to 66.65)	601	62192.44	9.66 (5.89 to 10.44)	1000	65929	15.17 (14.23 to 16.11)
	Most Deprivation	2315	47661.18	48.57 (46.59 to 50.55)	4181	58524	71.44 (69.28 to 73.61)	524	55215.6	9.49 (5.68 to 10.30)	926	58524	15.82 (14.80 to 16.84)
2007	Least Deprivation	3207	93912.3	34.15 (32.97 to 35.33)	5147	110017	46.78 (45.51 to 48.06)	637	105770.2	6.02 (5.55 to 6.49)	924	110017	8.40 (7.86 to 8.94)
	IMD=2	2930	84530.45	34.66 (33.41 to 35.92)	4728	99321	47.60 (46.25 to 48.96)	634	95252.28	6.66 (5.14 to 7.17)	931	99321	9.37 (8.77 to 9.98)
	IMD=3	2818	79215.89	35.57 (34.26 to 36.89)	4691	93466	50.19 (48.75 to 51.63)	657	89474.43	7.34 (5.78 to 7.90)	975	93466	10.43 (9.78 to 11.09)
	IMD=4	2594	70351.66	36.87 (35.45 to 38.29)	4263	83245	51.21 (49.67 to 52.75)	524	79593.65	6.58 (5.02 to 7.15)	779	83245	9.36 (8.70 to 10.02)
	IMD=5	2489	67350.87	36.96 (35.50 to 38.41)	4185	80111	52.24 (50.66 to 53.82)	558	76453.49	7.30 (5.69 to 7.90)	878	80111	10.96 (10.23 to 11.68)
	IMD=6	2476	64693.94	38.27 (36.76 to 39.78)	4210	77258	54.49 (52.85 to 56.14)	542	73610.59	7.36 (5.74 to 7.98)	870	77258	11.26 (10.51 to 12.01)
	IMD=7	2537	63656.56	39.85 (38.30 to 41.41)	4457	76674	58.13 (56.42 to 59.84)	524	72912.23	7.19 (5.57 to 7.80)	869	76674	11.33 (10.58 to 12.09)
	IMD=8	2201	52946.91	41.57 (39.83 to 43.31)	4001	64355	62.17 (60.24 to 64.10)	524	60938.27	8.60 (5.86 to 9.34)	852	64355	13.24 (12.35 to 14.13)

	IMD=9	2345	55565.52	42.20 (40.49 to 43.91)	4510	68519	65.82 (63.90 to 67.74)	573	64621.76	8.87 (5.14 to 9.59)	986	68519	14.39 (13.49 to 15.29)
	Most Deprivation	2331	48797.43	47.77 (45.83 to 49.71)	4505	60828	74.06 (71.90 to 76.22)	538	57331.68	9.38 (5.59 to 10.18)	934	60828	15.35 (14.37 to 16.34)
2008	Least Deprivation	3255	96965.23	33.57 (32.42 to 34.72)	5266	113146	46.54 (45.28 to 47.80)	690	109403.1	6.31 (5.84 to 6.78)	951	113146	8.41 (7.87 to 8.94)
	IMD=2	2968	86710.52	34.23 (33.00 to 35.46)	4767	101696	46.88 (45.54 to 48.21)	636	97905.52	6.50 (5.99 to 7.00)	920	101696	9.05 (8.46 to 9.63)
	IMD=3	2863	81751.49	35.02 (33.74 to 36.30)	4762	96188	49.51 (48.10 to 50.91)	618	92536.91	6.68 (5.15 to 7.20)	962	96188	10.00 (9.37 to 10.63)
	IMD=4	2578	72238.65	35.69 (34.31 to 37.06)	4281	85292	50.19 (48.69 to 51.70)	567	81973.16	6.92 (5.35 to 7.49)	849	85292	9.95 (9.28 to 10.62)
	IMD=5	2499	69455.17	35.98 (34.57 to 37.39)	4311	82354	52.35 (50.78 to 53.91)	582	78977.53	7.37 (5.77 to 7.97)	905	82354	10.99 (10.27 to 11.71)
	IMD=6	2481	66630.38	37.24 (35.77 to 38.70)	4429	79679	55.59 (53.95 to 57.22)	617	76116.98	8.11 (5.47 to 8.75)	965	79679	12.11 (11.35 to 12.88)
	IMD=7	2556	66059.23	38.69 (37.19 to 40.19)	4564	79412	57.47 (55.81 to 59.14)	627	75920.19	8.26 (5.61 to 8.91)	961	79412	12.10 (11.34 to 12.87)
	IMD=8	2246	55141.17	40.73 (39.05 to 42.42)	4181	67241	62.18 (60.29 to 64.06)	531	63815.51	8.32 (5.61 to 9.03)	897	67241	13.34 (12.47 to 14.21)
	IMD=9	2538	57721.82	43.97 (42.26 to 45.68)	4868	71446	68.14 (66.22 to 70.05)	649	67528.84	9.61 (5.87 to 10.35)	1047	71446	14.65 (13.77 to 15.54)
	Most Deprivation	2320	50618.9	45.83 (43.97 to 47.70)	4673	63549	73.53 (71.43 to 75.64)	578	59956.18	9.64 (5.85 to 10.43)	1023	63549	16.10 (15.11 to 17.08)
2009	Least Deprivation	3338	100078.4	33.35 (32.22 to 34.49)	5427	116959	46.40 (45.17 to 47.64)	649	112846.2	5.75 (5.31 to 6.19)	933	116959	7.98 (7.47 to 8.49)
	IMD=2	3252	89204.61	36.46 (35.20 to 37.71)	5142	104825	49.05 (47.71 to 50.39)	665	100862.1	6.59 (5.09 to 7.09)	942	104825	8.99 (8.41 to 9.56)
	IMD=3	3011	84342.42	35.70 (34.42 to 36.97)	5072	99383	51.03 (49.63 to 52.44)	638	95418.52	6.69 (5.17 to 7.21)	964	99383	9.70 (9.09 to 10.31)
	IMD=4	2782	74275.48	37.46 (36.06 to 38.85)	4625	87972	52.57 (51.06 to 54.09)	614	84437.95	7.27 (5.70 to 7.85)	870	87972	9.89 (9.23 to 10.55)
	IMD=5	2672	71590.34	37.32 (35.91 to 38.74)	4608	85078	54.16 (52.60 to 55.73)	565	81568.05	6.93 (5.36 to 7.50)	872	85078	10.25 (9.57 to 10.93)
	IMD=6	2713	68791.78	39.44 (37.95 to 40.92)	4669	82458	56.62 (55.00 to 58.25)	639	78779.28	8.11 (5.48 to 8.74)	996	82458	12.08 (11.33 to 12.83)
	IMD=7	2778	68289.91	40.68 (39.17 to 42.19)	4989	82290	60.63 (58.94 to 62.31)	632	78545.46	8.05 (5.42 to 8.67)	984	82290	11.96 (11.21 to 12.70)
	IMD=8	2448	57252.82	42.76 (41.06 to 44.45)	4561	70097	65.07 (63.18 to 66.96)	582	66503.69	8.75 (5.04 to 9.46)	953	70097	13.60 (12.73 to 14.46)
	IMD=9	2747	60010.13	45.78 (44.06 to 47.49)	5321	74551	71.37 (69.46 to 73.29)	684	70471.13	9.71 (5.98 to 10.43)	1135	74551	15.22 (14.34 to 16.11)
	Most Deprivation	2519	51812.27	48.62 (46.72 to 50.52)	5006	65452	76.48 (74.36 to 78.60)	663	61756.3	10.74 (5.92 to 11.55)	1113	65452	17.00 (16.01 to 18.00)
2010	Least Deprivation	3387	103935.3	32.59 (31.49 to 33.69)	5553	121308	45.78 (44.57 to 46.98)	734	117029.9	6.27 (5.82 to 6.73)	1019	121308	8.40 (7.88 to 8.92)
	IMD=2	3165	92352.43	34.27 (33.08 to 35.46)	5243	108571	48.29 (46.98 to 49.60)	690	104474.4	6.60 (5.11 to 7.10)	989	108571	9.11 (8.54 to 9.68)
	IMD=3	3137	87241.37	35.96 (34.70 to 37.22)	5332	103003	51.77 (50.38 to 53.15)	676	98841.13	6.84 (5.32 to 7.35)	994	103003	9.65 (9.05 to 10.25)
	IMD=4	2827	76859.52	36.78 (35.43 to 38.14)	4837	91214	53.03 (51.53 to 54.52)	580	87476.81	6.63 (5.09 to 7.17)	861	91214	9.44 (8.81 to 10.07)
	IMD=5	2674	74285.51	36.00 (34.63 to 37.36)	4635	88390	52.44 (50.93 to 53.95)	622	84666.57	7.35 (5.77 to 7.92)	933	88390	10.56 (9.88 to 11.23)
	IMD=6	2827	71039.7	39.79 (38.33 to 41.26)	4967	85573	58.04 (56.43 to 59.66)	652	81599.32	7.99 (5.38 to 8.60)	1027	85573	12.00 (11.27 to 12.74)
	IMD=7	2785	70832.39	39.32 (37.86 to 40.78)	5150	85766	60.05 (58.41 to 61.69)	708	81629.66	8.67 (5.03 to 9.31)	1089	85766	12.70 (11.94 to 13.45)
	IMD=8	2483	59500.53	41.73 (40.09 to 43.37)	4710	73062	64.47 (62.62 to 66.31)	606	69253.95	8.75 (5.05 to 9.45)	993	73062	13.59 (12.75 to 14.44)

	IMD=9	2974	62129.15	47.87 (46.15 to 49.59)	5779	77846	74.24 (72.32 to 76.15)	691	73434.61	9.41 (5.71 to 10.11)	1172	77846	15.06 (14.19 to 15.92)
	Most Deprivation	2562	53398.79	47.98 (46.12 to 49.84)	5442	68004	80.02 (77.90 to 82.15)	646	64014.64	10.09 (5.31 to 10.87)	1115	68004	16.40 (15.43 to 17.36)
2011	Least Deprivation	3604	106544.4	33.83 (32.72 to 34.93)	5760	124341	46.32 (45.13 to 47.52)	716	119803	5.98 (5.54 to 6.41)	967	124341	7.78 (7.29 to 8.27)
	IMD=2	3345	94465.09	35.41 (34.21 to 36.61)	5505	111231	49.49 (48.18 to 50.80)	745	106905.1	6.97 (5.47 to 7.47)	1049	111231	9.43 (8.86 to 10.00)
	IMD=3	3351	89021.63	37.64 (36.37 to 38.92)	5551	105524	52.60 (51.22 to 53.99)	668	101177.9	6.60 (5.10 to 7.10)	996	105524	9.44 (8.85 to 10.02)
	IMD=4	3007	79209.34	37.96 (36.61 to 39.32)	5104	94268	54.14 (52.66 to 55.63)	581	90354.42	6.43 (5.91 to 6.95)	878	94268	9.31 (8.70 to 9.93)
	IMD=5	2895	76391.42	37.90 (36.52 to 39.28)	4936	91274	54.08 (52.57 to 55.59)	608	87184.51	6.97 (5.42 to 7.53)	927	91274	10.16 (9.50 to 10.81)
	IMD=6	2875	72843.01	39.47 (38.03 to 40.91)	5136	88173	58.25 (56.66 to 59.84)	625	83998.31	7.44 (5.86 to 8.02)	1010	88173	11.45 (10.75 to 12.16)
	IMD=7	2894	72702.32	39.81 (38.36 to 41.26)	5425	88511	61.29 (59.66 to 62.92)	665	83950	7.92 (5.32 to 8.52)	1070	88511	12.09 (11.36 to 12.81)
	IMD=8	2725	60960.69	44.70 (43.02 to 46.38)	5114	75389	67.83 (65.98 to 69.69)	640	71248.59	8.98 (5.29 to 9.68)	1037	75389	13.76 (12.92 to 14.59)
	IMD=9	3082	64059.9	48.11 (46.41 to 49.81)	6051	80591	75.08 (73.19 to 76.97)	674	75971.37	8.87 (5.20 to 9.54)	1161	80591	14.41 (13.58 to 15.23)
	Most Deprivation	2798	54770.11	51.09 (49.19 to 52.98)	5862	70002	83.74 (81.60 to 85.88)	627	65811.67	9.53 (5.78 to 10.27)	1147	70002	16.39 (15.44 to 17.33)
2012	Least Deprivation	3570	108499.3	32.90 (31.82 to 33.98)	5722	126187	45.35 (44.17 to 46.52)	733	121907.2	6.01 (5.58 to 6.45)	1010	126187	8.00 (7.51 to 8.50)
	IMD=2	3392	95772.74	35.42 (34.23 to 36.61)	5576	112631	49.51 (48.21 to 50.81)	662	108605.6	6.10 (5.63 to 6.56)	981	112631	8.71 (8.16 to 9.25)
	IMD=3	3272	90190.38	36.28 (35.04 to 37.52)	5579	106818	52.23 (50.86 to 53.60)	697	102838.2	6.78 (5.27 to 7.28)	1031	106818	9.65 (9.06 to 10.24)
	IMD=4	3025	80813.84	37.43 (36.10 to 38.77)	5133	96161	53.38 (51.92 to 54.84)	664	92432.97	7.18 (5.64 to 7.73)	966	96161	10.05 (9.41 to 10.68)
	IMD=5	2905	77634.49	37.42 (36.06 to 38.78)	5054	92572	54.60 (53.09 to 56.10)	651	88829.18	7.33 (5.77 to 7.89)	992	92572	10.72 (10.05 to 11.38)
	IMD=6	3035	74283.23	40.86 (39.40 to 42.31)	5513	89824	61.38 (59.76 to 63.00)	665	85840.89	7.75 (5.16 to 8.34)	1034	89824	11.51 (10.81 to 12.21)
	IMD=7	3223	74014.57	43.55 (42.04 to 45.05)	5851	90036	64.99 (63.32 to 66.65)	703	85893.81	8.18 (5.58 to 8.79)	1110	90036	12.33 (11.60 to 13.05)
	IMD=8	2736	62497.53	43.78 (42.14 to 45.42)	5250	77254	67.96 (66.12 to 69.80)	615	73406.99	8.38 (5.72 to 9.04)	994	77254	12.87 (12.07 to 13.67)
	IMD=9	3167	65422.7	48.41 (46.72 to 50.09)	6448	82496	78.16 (76.25 to 80.07)	794	78071.46	10.17 (5.46 to 10.88)	1331	82496	16.13 (15.27 to 17.00)
	Most Deprivation	2910	55691.09	52.25 (50.35 to 54.15)	6210	71328	87.06 (84.90 to 89.23)	659	67438.82	9.77 (5.03 to 10.52)	1144	71328	16.04 (15.11 to 16.97)
2013	Least Deprivation	3526	108668.4	32.45 (31.38 to 33.52)	5700	127067	44.86 (43.69 to 46.02)	745	122226.2	6.10 (5.66 to 6.53)	1018	127067	8.01 (7.52 to 8.50)
	IMD=2	3337	96334.66	34.64 (33.46 to 35.81)	5540	113927	48.63 (47.35 to 49.91)	714	109228	6.54 (5.06 to 7.02)	993	113927	8.72 (8.17 to 9.26)
	IMD=3	3223	90869.56	35.47 (34.24 to 36.69)	5523	108184	51.05 (49.71 to 52.40)	728	103537.8	7.03 (5.52 to 7.54)	1049	108184	9.70 (9.11 to 10.28)
	IMD=4	3042	81065.18	37.53 (36.19 to 38.86)	5177	97178	53.27 (51.82 to 54.72)	703	92760.35	7.58 (5.02 to 8.14)	998	97178	10.27 (9.63 to 10.91)
	IMD=5	2947	77941.89	37.81 (36.45 to 39.18)	5124	93836	54.61 (53.11 to 56.10)	714	89248.35	8.00 (5.41 to 8.59)	1053	93836	11.22 (10.54 to 11.90)
	IMD=6	3040	74485.84	40.81 (39.36 to 42.26)	5411	91053	59.43 (57.84 to 61.01)	673	86241.63	7.80 (5.21 to 8.39)	1031	91053	11.32 (10.63 to 12.01)
	IMD=7	3079	74204.1	41.49 (40.03 to 42.96)	5833	91791	63.55 (61.92 to 65.18)	720	86450.74	8.33 (5.72 to 8.94)	1133	91791	12.34 (11.62 to 13.06)
	IMD=8	2875	62971.8	45.66 (43.99 to 47.32)	5471	79290	69.00 (67.17 to 70.83)	744	74170.89	10.03 (5.31 to 10.75)	1158	79290	14.60 (13.76 to 15.45)

	IMD=9	3231	65222.09	49.54 (47.83 to 51.25)	6698	84422	79.34 (77.44 to 81.24)	857	78243.95	10.95 (5.22 to 11.69)	1414	84422	16.75 (15.88 to 17.62)
	Most Deprivation	3013	55641.38	54.15 (52.22 to 56.08)	6404	72928	87.81 (85.66 to 89.96)	722	67980.51	10.62 (5.85 to 11.40)	1235	72928	16.93 (15.99 to 17.88)
2014	Least Deprivation	3612	109138.3	33.10 (32.02 to 34.17)	5718	127745	44.76 (43.60 to 45.92)	781	122807.1	6.36 (5.91 to 6.81)	1036	127745	8.11 (7.62 to 8.60)
	IMD=2	3262	96705.5	33.73 (32.57 to 34.89)	5390	114448	47.10 (45.84 to 48.35)	828	109569.4	7.56 (5.04 to 8.07)	1140	114448	9.96 (9.38 to 10.54)
	IMD=3	3343	91482.11	36.54 (35.30 to 37.78)	5595	109028	51.32 (49.97 to 52.66)	788	104099.7	7.57 (5.04 to 8.10)	1134	109028	10.40 (9.80 to 11.01)
	IMD=4	3049	81572.93	37.38 (36.05 to 38.70)	5293	97792	54.13 (52.67 to 55.58)	756	93268.05	8.11 (5.53 to 8.68)	1065	97792	10.89 (10.24 to 11.54)
	IMD=5	3075	78381.84	39.23 (37.84 to 40.62)	5309	94283	56.31 (54.79 to 57.82)	733	89847.59	8.16 (5.57 to 8.75)	1052	94283	11.16 (10.48 to 11.83)
	IMD=6	3085	74966.08	41.15 (39.70 to 42.60)	5568	91507	60.85 (59.25 to 62.45)	730	86984.34	8.39 (5.78 to 9.00)	1104	91507	12.06 (11.35 to 12.78)
	IMD=7	3127	74932.98	41.73 (40.27 to 43.19)	5843	92308	63.30 (61.68 to 64.92)	805	87505.77	9.20 (5.56 to 9.83)	1224	92308	13.26 (12.52 to 14.00)
	IMD=8	2896	63955.32	45.28 (43.63 to 46.93)	5525	80050	69.02 (67.20 to 70.84)	775	75474.85	10.27 (5.55 to 10.99)	1192	80050	14.89 (14.05 to 15.74)
	IMD=9	3248	66116.33	49.13 (47.44 to 50.82)	6558	84745	77.39 (75.51 to 79.26)	904	79502.52	11.37 (5.63 to 12.11)	1478	84745	17.44 (16.55 to 18.33)
	Most Deprivation	2966	55856.76	53.10 (51.19 to 55.01)	6303	72963	86.39 (84.25 to 88.52)	801	68563.31	11.68 (5.87 to 12.49)	1321	72963	18.11 (17.13 to 19.08)
2015	Least Deprivation	3508	109912	31.92 (30.86 to 32.97)	5533	128061	43.21 (42.07 to 44.34)	781	123348.2	6.33 (5.89 to 6.78)	1059	128061	8.27 (7.77 to 8.77)
	IMD=2	3360	97118.21	34.60 (33.43 to 35.77)	5408	114398	47.27 (46.01 to 48.53)	796	109743.9	7.25 (5.75 to 7.76)	1149	114398	10.04 (9.46 to 10.62)
	IMD=3	3197	92071.03	34.72 (33.52 to 35.93)	5379	109294	49.22 (47.90 to 50.53)	818	104541.7	7.82 (5.29 to 8.36)	1164	109294	10.65 (10.04 to 11.26)
	IMD=4	3009	82235.23	36.59 (35.28 to 37.90)	5165	98089	52.66 (51.22 to 54.09)	754	93808.34	8.04 (5.46 to 8.61)	1088	98089	11.09 (10.43 to 11.75)
	IMD=5	2878	78866.11	36.49 (35.16 to 37.83)	4968	94663	52.48 (51.02 to 53.94)	754	90212.63	8.36 (5.76 to 8.95)	1121	94663	11.84 (11.15 to 12.54)
	IMD=6	2903	75429.35	38.49 (37.09 to 39.89)	5323	91917	57.91 (56.36 to 59.47)	798	87533.56	9.12 (5.48 to 9.75)	1208	91917	13.14 (12.40 to 13.88)
	IMD=7	3095	75758.87	40.85 (39.41 to 42.29)	5773	93149	61.98 (60.38 to 63.57)	883	88411.33	9.99 (5.33 to 10.65)	1318	93149	14.15 (13.39 to 14.91)
	IMD=8	2882	65217.93	44.19 (42.58 to 45.80)	5518	81311	67.86 (66.07 to 69.65)	792	76795.38	10.31 (5.59 to 11.03)	1253	81311	15.41 (14.56 to 16.26)
	IMD=9	3276	67424.8	48.59 (46.92 to 50.25)	6692	86272	77.57 (75.71 to 79.43)	910	81006.92	11.23 (5.50 to 11.96)	1520	86272	17.62 (16.73 to 18.50)
	Most Deprivation	2924	56733.62	51.54 (49.67 to 53.41)	6224	74081	84.02 (81.93 to 86.10)	853	69565.84	12.26 (5.44 to 13.08)	1407	74081	18.99 (18.00 to 19.99)
2016	Least Deprivation	3372	111087.7	30.35 (29.33 to 31.38)	5364	128938	41.60 (40.49 to 42.71)	903	124234.4	7.27 (5.79 to 7.74)	1212	128938	9.40 (8.87 to 9.93)
	IMD=2	3261	97918.75	33.30 (32.16 to 34.45)	5287	114734	46.08 (44.84 to 47.32)	876	110314.7	7.94 (5.42 to 8.47)	1179	114734	10.28 (9.69 to 10.86)
	IMD=3	3235	92874.51	34.83 (33.63 to 36.03)	5318	109691	48.48 (47.18 to 49.78)	893	105252	8.48 (5.93 to 9.04)	1256	109691	11.45 (10.82 to 12.08)
	IMD=4	2902	83199.13	34.88 (33.61 to 36.15)	4972	98852	50.30 (48.90 to 51.70)	770	94736.91	8.13 (5.55 to 8.70)	1118	98852	11.31 (10.65 to 11.97)
	IMD=5	2789	80073.27	34.83 (33.54 to 36.12)	4910	95336	51.50 (50.06 to 52.94)	758	91248.9	8.31 (5.72 to 8.90)	1114	95336	11.68 (11.00 to 12.37)
	IMD=6	2909	77015.37	37.77 (36.40 to 39.14)	5220	92985	56.14 (54.62 to 57.66)	852	88800.18	9.59 (5.95 to 10.24)	1265	92985	13.60 (12.85 to 14.35)
	IMD=7	2988	77674.13	38.47 (37.09 to 39.85)	5507	94428	58.32 (56.78 to 59.86)	845	89913.32	9.40 (5.76 to 10.03)	1320	94428	13.98 (13.22 to 14.73)
	IMD=8	2802	67000.05	41.82 (40.27 to 43.37)	5439	82954	65.57 (63.82 to 67.31)	791	78674.13	10.05 (5.35 to 10.75)	1294	82954	15.60 (14.75 to 16.45)

	IMD=9	3256	69060.84	47.15 (45.53 to 48.77)	6623	87715	75.51 (73.69 to 77.32)	1012	82685.86	12.24 (5.49 to 12.99)	1694	87715	19.31 (18.39 to 20.23)
	Most Deprivation	2901	58120.96	49.91 (48.10 to 51.73)	6134	75159	81.61 (79.57 to 83.66)	873	70812	12.33 (5.51 to 13.15)	1481	75159	19.70 (18.70 to 20.71)
2017	Least Deprivation	3423	111529.1	30.69 (29.66 to 31.72)	5215	128946	40.44 (39.35 to 41.54)	869	124105.3	7.00 (5.54 to 7.47)	1198	128946	9.29 (8.76 to 9.82)
	IMD=2	3040	98390.07	30.90 (29.80 to 32.00)	4926	115027	42.82 (41.63 to 44.02)	937	110209.8	8.50 (5.96 to 9.05)	1282	115027	11.15 (10.54 to 11.76)
	IMD=3	2960	92951.84	31.84 (30.70 to 32.99)	4933	109456	45.07 (43.81 to 46.33)	943	104720.7	9.00 (5.43 to 9.58)	1310	109456	11.97 (11.32 to 12.62)
	IMD=4	2827	83713.32	33.77 (32.53 to 35.01)	4680	99184	47.19 (45.83 to 48.54)	778	94779.89	8.21 (5.63 to 8.79)	1140	99184	11.49 (10.83 to 12.16)
	IMD=5	2789	80665.45	34.57 (33.29 to 35.86)	4696	95795	49.02 (47.62 to 50.42)	796	91389.89	8.71 (5.10 to 9.32)	1156	95795	12.07 (11.37 to 12.76)
	IMD=6	2895	77589.63	37.31 (35.95 to 38.67)	5174	93571	55.29 (53.79 to 56.80)	832	88993.08	9.35 (5.71 to 9.98)	1287	93571	13.75 (13.00 to 14.51)
	IMD=7	3075	78617.88	39.11 (37.73 to 40.50)	5511	95470	57.72 (56.20 to 59.25)	893	90519.15	9.87 (5.22 to 10.51)	1425	95470	14.93 (14.15 to 15.70)
	IMD=8	2766	68440.88	40.41 (38.91 to 41.92)	5235	84504	61.95 (60.27 to 63.63)	865	79824.36	10.84 (5.11 to 11.56)	1360	84504	16.09 (15.24 to 16.95)
	IMD=9	3050	70735.38	43.12 (41.59 to 44.65)	6240	89477	69.74 (68.01 to 71.47)	987	84014.35	11.75 (5.02 to 12.48)	1681	89477	18.79 (17.89 to 19.69)
	Most Deprivation	2902	59226.19	49.00 (47.22 to 50.78)	6147	76372	80.49 (78.48 to 82.50)	907	71597.72	12.67 (5.84 to 13.49)	1538	76372	20.14 (19.13 to 21.14)
2018	Least Deprivation	3208	112190.5	28.59 (27.60 to 29.58)	4872	129226	37.70 (36.64 to 38.76)	937	124106	7.55 (5.07 to 8.03)	1253	129226	9.70 (9.16 to 10.23)
	IMD=2	3049	98854.94	30.84 (29.75 to 31.94)	4777	115317	41.42 (40.25 to 42.60)	909	110055.3	8.26 (5.72 to 8.80)	1256	115317	10.89 (10.29 to 11.49)
	IMD=3	2917	94275.76	30.94 (29.82 to 32.06)	4812	110513	43.54 (42.31 to 44.77)	855	105387.1	8.11 (5.57 to 8.66)	1263	110513	11.43 (10.80 to 12.06)
	IMD=4	2752	85081.7	32.35 (31.14 to 33.55)	4603	100239	45.92 (44.59 to 47.25)	837	95511.53	8.76 (5.17 to 9.36)	1217	100239	12.14 (11.46 to 12.82)
	IMD=5	2665	81635.66	32.65 (31.41 to 33.88)	4512	96552	46.73 (45.37 to 48.09)	803	91882.04	8.74 (5.13 to 9.34)	1196	96552	12.39 (11.69 to 13.09)
	IMD=6	2873	78512.74	36.59 (35.25 to 37.93)	4987	94603	52.72 (51.25 to 54.18)	872	89451.17	9.75 (5.10 to 10.40)	1312	94603	13.87 (13.12 to 14.62)
	IMD=7	2848	79578.18	35.79 (34.47 to 37.10)	5131	96488	53.18 (51.72 to 54.63)	929	90919.33	10.22 (5.56 to 10.87)	1394	96488	14.45 (13.69 to 15.21)
	IMD=8	2670	69845.97	38.23 (36.78 to 39.68)	5022	85892	58.47 (56.85 to 60.09)	869	80845.09	10.75 (5.03 to 11.46)	1367	85892	15.92 (15.07 to 16.76)
	IMD=9	3203	72362.96	44.26 (42.73 to 45.80)	6183	91246	67.76 (66.07 to 69.45)	998	85232.95	11.71 (5.98 to 12.44)	1678	91246	18.39 (17.51 to 19.27)
	Most Deprivation	2802	60187.89	46.55 (44.83 to 48.28)	5767	77317	74.59 (72.66 to 76.51)	896	72021.73	12.44 (5.63 to 13.26)	1532	77317	19.81 (18.82 to 20.81)
2019	Least Deprivation	3059	110032.9	27.80 (26.82 to 28.79)	4666	127814	36.51 (35.46 to 37.55)	980	120992	8.10 (5.59 to 8.61)	1315	127814	10.29 (9.73 to 10.84)
	IMD=2	2745	96634	28.41 (27.34 to 29.47)	4416	113940	38.76 (37.61 to 39.90)	941	106937.4	8.80 (5.24 to 9.36)	1306	113940	11.46 (10.84 to 12.08)
	IMD=3	2667	90987.63	29.31 (28.20 to 30.42)	4299	109137	39.39 (38.21 to 40.57)	878	100951.1	8.70 (5.12 to 9.27)	1279	109137	11.72 (11.08 to 12.36)
	IMD=4	2542	81965.8	31.01 (29.81 to 32.22)	4272	98827	43.23 (41.93 to 44.52)	784	91529.23	8.57 (5.97 to 9.17)	1189	98827	12.03 (11.35 to 12.71)
	IMD=5	2611	80009.14	32.63 (31.38 to 33.89)	4327	95824	45.16 (43.81 to 46.50)	851	89507.34	9.51 (5.87 to 10.15)	1229	95824	12.83 (12.11 to 13.54)
	IMD=6	2656	77249.77	34.38 (33.07 to 35.69)	4581	93915	48.78 (47.37 to 50.19)	885	87355.04	10.13 (5.46 to 10.80)	1321	93915	14.07 (13.31 to 14.82)
	IMD=7	2838	78237.33	36.27 (34.94 to 37.61)	4925	95666	51.48 (50.04 to 52.92)	853	88805.68	9.61 (5.96 to 10.25)	1325	95666	13.85 (13.10 to 14.60)
	IMD=8	2609	68657.59	38.00 (36.54 to 39.46)	4819	85693	56.24 (54.65 to 57.82)	850	78744.41	10.79 (5.07 to 11.52)	1376	85693	16.06 (15.21 to 16.91)

	IMD=9	3084	71566.02	43.09 (41.57 to 44.61)	5793	90437	64.06 (62.41 to 65.71)	1012	83581.77	12.11 (5.36 to 12.85)	1630	90437	18.02 (17.15 to 18.90)
	Most Deprivation	2733	57589.13	47.46 (45.68 to 49.24)	5467	74404	73.48 (71.53 to 75.42)	891	68320.82	13.04 (5.19 to 13.90)	1512	74404	20.32 (19.30 to 21.35)
**55-64 years**													
2004	Least Deprivation	2863	83823.13	34.16 (32.90 to 35.41)	4456	96441	46.20 (44.85 to 47.56)	1532	89594.09	17.10 (16.24 to 17.96)	2424	96441	25.13 (24.13 to 26.14)
	IMD=2	2699	77932.43	34.63 (33.33 to 35.94)	4092	89894	45.52 (44.13 to 46.92)	1526	82603.58	18.47 (17.55 to 19.40)	2515	89894	27.98 (26.88 to 29.07)
	IMD=3	2688	73982.84	36.33 (34.96 to 37.71)	4096	84971	48.20 (46.73 to 49.68)	1464	78339.07	18.69 (17.73 to 19.65)	2417	84971	28.45 (27.31 to 29.58)
	IMD=4	2469	63885.89	38.65 (37.12 to 40.17)	3729	73942	50.43 (48.81 to 52.05)	1345	67796.92	19.84 (18.78 to 20.90)	2165	73942	29.28 (28.05 to 30.51)
	IMD=5	2254	59421.03	37.93 (36.37 to 39.50)	3521	68807	51.17 (49.48 to 52.86)	1213	63006.54	19.25 (18.17 to 20.34)	2152	68807	31.28 (29.95 to 32.60)
	IMD=6	2195	57118.21	38.43 (36.82 to 40.04)	3457	66197	52.22 (50.48 to 53.96)	1208	60642.82	19.92 (18.80 to 21.04)	2102	66197	31.75 (30.40 to 33.11)
	IMD=7	2102	54188.52	38.79 (37.13 to 40.45)	3299	62975	52.39 (50.60 to 54.17)	1171	57428.91	20.39 (19.22 to 21.56)	2042	62975	32.43 (31.02 to 33.83)
	IMD=8	1790	43199.56	41.44 (39.52 to 43.36)	2823	50421	55.99 (53.92 to 58.05)	1013	45841.48	22.10 (20.74 to 23.46)	1742	50421	34.55 (32.93 to 36.17)
	IMD=9	1812	43472.09	41.68 (39.76 to 43.60)	2932	50878	57.63 (55.54 to 59.71)	979	46156.1	21.21 (19.88 to 22.54)	1760	50878	34.59 (32.98 to 36.21)
	Most Deprivation	1726	37884.39	45.56 (43.41 to 47.71)	2853	44719	63.80 (61.46 to 66.14)	1004	40288	24.92 (23.38 to 26.46)	1807	44719	40.41 (38.54 to 42.27)
2005	Least Deprivation	2936	85020.75	34.53 (33.28 to 35.78)	4594	98688	46.55 (45.20 to 47.90)	1472	91401.34	16.10 (15.28 to 16.93)	2423	98688	24.55 (23.57 to 25.53)
	IMD=2	2840	79027.7	35.94 (34.62 to 37.26)	4468	91681	48.73 (47.31 to 50.16)	1487	84214.42	17.66 (16.76 to 18.55)	2553	91681	27.85 (26.77 to 28.93)
	IMD=3	2785	74686.14	37.29 (35.90 to 38.67)	4349	86784	50.11 (48.62 to 51.60)	1514	79762.37	18.98 (18.03 to 19.94)	2563	86784	29.53 (28.39 to 30.68)
	IMD=4	2425	64778.13	37.44 (35.95 to 38.93)	3888	75730	51.34 (49.73 to 52.95)	1291	69353.84	18.61 (17.60 to 19.63)	2224	75730	29.37 (28.15 to 30.59)
	IMD=5	2299	60272.72	38.14 (36.58 to 39.70)	3711	70550	52.60 (50.91 to 54.29)	1260	64485.75	19.54 (18.46 to 20.62)	2250	70550	31.89 (30.57 to 33.21)
	IMD=6	2182	57759.32	37.78 (36.19 to 39.36)	3587	67964	52.78 (51.05 to 54.51)	1213	61965.42	19.58 (18.47 to 20.68)	2196	67964	32.31 (30.96 to 33.66)
	IMD=7	2211	54971.31	40.22 (38.54 to 41.90)	3649	64947	56.18 (54.36 to 58.01)	1189	58949.52	20.17 (19.02 to 21.32)	2156	64947	33.20 (31.80 to 34.60)
	IMD=8	1783	43557.59	40.93 (39.03 to 42.83)	2952	51701	57.10 (55.04 to 59.16)	967	46695.23	20.71 (19.40 to 22.01)	1832	51701	35.43 (33.81 to 37.06)
	IMD=9	1890	43728.22	43.22 (41.27 to 45.17)	3188	52267	60.99 (58.88 to 63.11)	1024	47074.36	21.75 (20.42 to 23.09)	1930	52267	36.93 (35.28 to 38.57)
	Most Deprivation	1837	37900.43	48.47 (46.25 to 50.69)	3142	45728	68.71 (66.31 to 71.11)	961	40811.13	23.55 (22.06 to 25.04)	1914	45728	41.86 (39.98 to 43.73)
2006	Least Deprivation	3130	86893.14	36.02 (34.76 to 37.28)	4914	101510	48.41 (47.06 to 49.76)	1463	93894.94	15.58 (14.78 to 16.38)	2389	101510	23.53 (22.59 to 24.48)
	IMD=2	2953	80585.21	36.64 (35.32 to 37.97)	4742	94177	50.35 (48.92 to 51.79)	1442	86457.16	16.68 (15.82 to 17.54)	2550	94177	27.08 (26.03 to 28.13)
	IMD=3	2751	76153.35	36.12 (34.77 to 37.47)	4537	89418	50.74 (49.26 to 52.22)	1323	81888.28	16.16 (15.29 to 17.03)	2388	89418	26.71 (25.63 to 27.78)
	IMD=4	2446	66269.19	36.91 (35.45 to 38.37)	3975	78179	50.84 (49.26 to 52.43)	1259	71379.73	17.64 (16.66 to 18.61)	2236	78179	28.60 (27.42 to 29.79)
	IMD=5	2273	61677.21	36.85 (35.34 to 38.37)	3816	72844	52.39 (50.72 to 54.05)	1181	66307.83	17.81 (16.80 to 18.83)	2152	72844	29.54 (28.29 to 30.79)
	IMD=6	2184	58518.57	37.32 (35.76 to 38.89)	3759	69749	53.89 (52.17 to 55.62)	1207	63103.33	19.13 (18.05 to 20.21)	2205	69749	31.61 (30.29 to 32.93)
	IMD=7	2202	55880.95	39.41 (37.76 to 41.05)	3828	67025	57.11 (55.30 to 58.92)	1123	60374.04	18.60 (17.51 to 19.69)	2213	67025	33.02 (31.64 to 34.39)

	IMD=8	1837	44050.27	41.70 (39.80 to 43.61)	3108	53183	58.44 (56.39 to 60.49)	913	47745.2	19.12 (17.88 to 20.36)	1802	53183	33.88 (32.32 to 35.45)
	IMD=9	1947	44064.72	44.19 (42.22 to 46.15)	3440	53577	64.21 (62.06 to 66.35)	991	47934.66	20.67 (19.39 to 21.96)	1934	53577	36.10 (34.49 to 37.71)
	Most Deprivation	1767	38068.43	46.42 (44.25 to 48.58)	3285	46907	70.03 (67.64 to 72.43)	910	41712.6	21.82 (20.40 to 23.23)	1912	46907	40.76 (38.93 to 42.59)
2007	Least Deprivation	2999	87521.14	34.27 (33.04 to 35.49)	4888	102768	47.56 (46.23 to 48.90)	1391	94923.41	14.65 (13.88 to 15.42)	2348	102768	22.85 (21.92 to 23.77)
	IMD=2	2928	80990.92	36.15 (34.84 to 37.46)	4853	95309	50.92 (49.49 to 52.35)	1448	87442.13	16.56 (15.71 to 17.41)	2551	95309	26.77 (25.73 to 27.80)
	IMD=3	2792	76400.28	36.54 (35.19 to 37.90)	4606	90424	50.94 (49.47 to 52.41)	1411	82681.98	17.07 (16.17 to 17.96)	2592	90424	28.66 (27.56 to 29.77)
	IMD=4	2556	66708.26	38.32 (36.83 to 39.80)	4178	79222	52.74 (51.14 to 54.34)	1348	72326.83	18.64 (17.64 to 19.63)	2363	79222	29.83 (28.62 to 31.03)
	IMD=5	2311	62146.68	37.19 (35.67 to 38.70)	3948	73967	53.38 (51.71 to 55.04)	1212	67165.91	18.04 (17.03 to 19.06)	2248	73967	30.39 (29.14 to 31.65)
	IMD=6	2397	59004.26	40.62 (39.00 to 42.25)	4005	70540	56.78 (55.02 to 58.53)	1199	63961.16	18.75 (17.68 to 19.81)	2219	70540	31.46 (30.15 to 32.77)
	IMD=7	2148	56214.73	38.21 (36.59 to 39.83)	3831	67605	56.67 (54.87 to 58.46)	1172	61074.83	19.19 (18.09 to 20.29)	2192	67605	32.42 (31.07 to 33.78)
	IMD=8	1794	44362.14	40.44 (38.57 to 42.31)	3131	53883	58.11 (56.07 to 60.14)	959	48431.81	19.80 (18.55 to 21.05)	1831	53883	33.98 (32.42 to 35.54)
	IMD=9	1973	44137.31	44.70 (42.73 to 46.67)	3593	54392	66.06 (63.90 to 68.22)	976	48531.57	20.11 (18.85 to 21.37)	1969	54392	36.20 (34.60 to 37.80)
	Most Deprivation	1870	38388.34	48.71 (46.50 to 50.92)	3512	47951	73.24 (70.82 to 75.66)	924	42557.22	21.71 (20.31 to 23.11)	1950	47951	40.67 (38.86 to 42.47)
2008	Least Deprivation	3114	88231.77	35.29 (34.05 to 36.53)	4965	103109	48.15 (46.81 to 49.49)	1507	95811.05	15.73 (14.93 to 16.52)	2466	103109	23.92 (22.97 to 24.86)
	IMD=2	2976	81801.15	36.38 (35.07 to 37.69)	4920	96212	51.14 (49.71 to 52.57)	1500	88768.34	16.90 (16.04 to 17.75)	2606	96212	27.09 (26.05 to 28.13)
	IMD=3	2821	77141.7	36.57 (35.22 to 37.92)	4759	91112	52.23 (50.75 to 53.72)	1452	83692.35	17.35 (16.46 to 18.24)	2549	91112	27.98 (26.89 to 29.06)
	IMD=4	2529	67464.76	37.49 (36.03 to 38.95)	4113	80031	51.39 (49.82 to 52.96)	1280	73352.89	17.45 (16.49 to 18.41)	2284	80031	28.54 (27.37 to 29.71)
	IMD=5	2385	62833.14	37.96 (36.43 to 39.48)	4067	74700	54.44 (52.77 to 56.12)	1315	68228.28	19.27 (18.23 to 20.32)	2276	74700	30.47 (29.22 to 31.72)
	IMD=6	2320	59589.12	38.93 (37.35 to 40.52)	4074	71246	57.18 (55.43 to 58.94)	1234	64869.79	19.02 (17.96 to 20.08)	2316	71246	32.51 (31.18 to 33.83)
	IMD=7	2307	57008.55	40.47 (38.82 to 42.12)	4115	68496	60.08 (58.24 to 61.91)	1207	62143.55	19.42 (18.33 to 20.52)	2330	68496	34.02 (32.64 to 35.40)
	IMD=8	1899	45100.83	42.11 (40.21 to 44.00)	3432	54864	62.55 (60.46 to 64.65)	1056	49381.93	21.38 (20.09 to 22.67)	1988	54864	36.24 (34.64 to 37.83)
	IMD=9	1965	44790.28	43.87 (41.93 to 45.81)	3797	55428	68.50 (66.32 to 70.68)	1086	49515.54	21.93 (20.63 to 23.24)	2128	55428	38.39 (36.76 to 40.02)
	Most Deprivation	1773	38965.13	45.50 (43.38 to 47.62)	3566	48932	72.88 (70.48 to 75.27)	1003	43422.53	23.10 (21.67 to 24.53)	2104	48932	43.00 (41.16 to 44.84)
2009	Least Deprivation	3015	87890.66	34.30 (33.08 to 35.53)	4918	103171	47.67 (46.34 to 49.00)	1624	95522.7	17.00 (16.17 to 17.83)	2540	103171	24.62 (23.66 to 25.58)
	IMD=2	2876	81161.98	35.44 (34.14 to 36.73)	4801	95911	50.06 (48.64 to 51.47)	1562	88273.05	17.70 (16.82 to 18.57)	2615	95911	27.26 (26.22 to 28.31)
	IMD=3	2927	77110.78	37.96 (36.58 to 39.33)	4819	91376	52.74 (51.25 to 54.23)	1607	83830.04	19.17 (18.23 to 20.11)	2769	91376	30.30 (29.17 to 31.43)
	IMD=4	2526	67313.09	37.53 (36.06 to 38.99)	4269	80041	53.34 (51.74 to 54.94)	1350	73341.45	18.41 (17.43 to 19.39)	2341	80041	29.25 (28.06 to 30.43)
	IMD=5	2403	62962.01	38.17 (36.64 to 39.69)	4149	75120	55.23 (53.55 to 56.91)	1331	68569.59	19.41 (18.37 to 20.45)	2350	75120	31.28 (30.02 to 32.55)
	IMD=6	2390	59520.4	40.15 (38.54 to 41.76)	4162	71498	58.21 (56.44 to 59.98)	1252	64944.88	19.28 (18.21 to 20.35)	2342	71498	32.76 (31.43 to 34.08)
	IMD=7	2273	57126.3	39.79 (38.15 to 41.42)	4129	69148	59.71 (57.89 to 61.53)	1196	62539.35	19.12 (18.04 to 20.21)	2325	69148	33.62 (32.26 to 34.99)

	IMD=8	1901	45320.41	41.95 (40.06 to 43.83)	3509	55358	63.39 (61.29 to 65.48)	1048	49708.8	21.08 (19.81 to 22.36)	2027	55358	36.62 (35.02 to 38.21)
	IMD=9	2166	45136.8	47.99 (45.97 to 50.01)	4041	56277	71.81 (69.59 to 74.02)	1135	50200.23	22.61 (21.29 to 23.92)	2228	56277	39.59 (37.95 to 41.23)
	Most Deprivation	1871	39182.73	47.75 (45.59 to 49.91)	3815	49642	76.85 (74.41 to 79.29)	991	43858.89	22.60 (21.19 to 24.00)	2162	49642	43.55 (41.72 to 45.39)
2010	Least Deprivation	3025	88579.35	34.15 (32.93 to 35.37)	4986	103991	47.95 (46.62 to 49.28)	1514	96062.02	15.76 (14.97 to 16.55)	2508	103991	24.12 (23.17 to 25.06)
	IMD=2	2929	81982.8	35.73 (34.43 to 37.02)	4866	96859	50.24 (48.83 to 51.65)	1603	88967.09	18.02 (17.14 to 18.90)	2691	96859	27.78 (26.73 to 28.83)
	IMD=3	2969	77771.91	38.18 (36.80 to 39.55)	5019	92594	54.20 (52.70 to 55.70)	1484	84725.01	17.52 (16.62 to 18.41)	2683	92594	28.98 (27.88 to 30.07)
	IMD=4	2490	67972.86	36.63 (35.19 to 38.07)	4304	81058	53.10 (51.51 to 54.68)	1358	74094.67	18.33 (17.35 to 19.30)	2367	81058	29.20 (28.02 to 30.38)
	IMD=5	2357	63931.33	36.87 (35.38 to 38.36)	4167	76576	54.42 (52.76 to 56.07)	1304	69573.21	18.74 (17.73 to 19.76)	2368	76576	30.92 (29.68 to 32.17)
	IMD=6	2349	60381.33	38.90 (37.33 to 40.48)	4170	72865	57.23 (55.49 to 58.97)	1272	66119.09	19.24 (18.18 to 20.30)	2376	72865	32.61 (31.30 to 33.92)
	IMD=7	2337	58036.16	40.27 (38.64 to 41.90)	4271	70460	60.62 (58.80 to 62.43)	1251	63600.02	19.67 (18.58 to 20.76)	2416	70460	34.29 (32.92 to 35.66)
	IMD=8	1982	46302.71	42.81 (40.92 to 44.69)	3651	56840	64.23 (62.15 to 66.32)	1087	50923.26	21.35 (20.08 to 22.61)	2133	56840	37.53 (35.93 to 39.12)
	IMD=9	2210	45805.73	48.25 (46.24 to 50.26)	4163	57522	72.37 (70.17 to 74.57)	1104	51214.61	21.56 (20.28 to 22.83)	2266	57522	39.39 (37.77 to 41.02)
	Most Deprivation	2028	39829.27	50.92 (48.70 to 53.13)	4074	50768	80.25 (77.78 to 82.71)	1087	44785.16	24.27 (22.83 to 25.71)	2243	50768	44.18 (42.35 to 46.01)
2011	Least Deprivation	3089	87762.64	35.20 (33.96 to 36.44)	4983	102971	48.39 (47.05 to 49.74)	1512	95225.59	15.88 (15.08 to 16.68)	2432	102971	23.62 (22.68 to 24.56)
	IMD=2	2961	81215.87	36.46 (35.15 to 37.77)	4978	96098	51.80 (50.36 to 53.24)	1518	88114.91	17.23 (16.36 to 18.09)	2589	96098	26.94 (25.90 to 27.98)
	IMD=3	2924	77292	37.83 (36.46 to 39.20)	4981	92107	54.08 (52.58 to 55.58)	1473	84268.28	17.48 (16.59 to 18.37)	2641	92107	28.67 (27.58 to 29.77)
	IMD=4	2603	67509.67	38.56 (37.08 to 40.04)	4383	80492	54.45 (52.84 to 56.06)	1347	73559.79	18.31 (17.33 to 19.29)	2324	80492	28.87 (27.70 to 30.05)
	IMD=5	2361	63852.04	36.98 (35.48 to 38.47)	4226	76605	55.17 (53.50 to 56.83)	1268	69591.55	18.22 (17.22 to 19.22)	2268	76605	29.61 (28.39 to 30.82)
	IMD=6	2474	60253.38	41.06 (39.44 to 42.68)	4436	72832	60.91 (59.11 to 62.70)	1265	65977.6	19.17 (18.12 to 20.23)	2337	72832	32.09 (30.79 to 33.39)
	IMD=7	2409	58314.59	41.31 (39.66 to 42.96)	4395	70968	61.93 (60.10 to 63.76)	1157	64043.86	18.07 (17.02 to 19.11)	2324	70968	32.75 (31.42 to 34.08)
	IMD=8	2113	46429.15	45.51 (43.57 to 47.45)	3940	57380	68.67 (66.52 to 70.81)	1075	51287.23	20.96 (19.71 to 22.21)	2145	57380	37.38 (35.80 to 38.96)
	IMD=9	2190	46331.18	47.27 (45.29 to 49.25)	4376	58436	74.89 (72.67 to 77.10)	1129	51959.36	21.73 (20.46 to 23.00)	2320	58436	39.70 (38.09 to 41.32)
	Most Deprivation	2152	40097.01	53.67 (51.40 to 55.94)	4357	51394	84.78 (82.26 to 87.29)	1085	45274.83	23.96 (22.54 to 25.39)	2352	51394	45.76 (43.91 to 47.61)
2012	Least Deprivation	3119	86157.36	36.20 (34.93 to 37.47)	5023	101165	49.65 (48.28 to 51.02)	1439	93861.08	15.33 (14.54 to 16.12)	2340	101165	23.13 (22.19 to 24.07)
	IMD=2	2923	80026.66	36.53 (35.20 to 37.85)	4886	94517	51.69 (50.24 to 53.14)	1489	87013.7	17.11 (16.24 to 17.98)	2518	94517	26.64 (25.60 to 27.68)
	IMD=3	2873	75930.88	37.84 (36.45 to 39.22)	4950	90487	54.70 (53.18 to 56.23)	1480	83063.34	17.82 (16.91 to 18.73)	2539	90487	28.06 (26.97 to 29.15)
	IMD=4	2525	66379.16	38.04 (36.56 to 39.52)	4339	79119	54.84 (53.21 to 56.47)	1257	72598.68	17.31 (16.36 to 18.27)	2214	79119	27.98 (26.82 to 29.15)
	IMD=5	2395	63169.7	37.91 (36.40 to 39.43)	4201	75467	55.67 (53.98 to 57.35)	1304	68994.55	18.90 (17.87 to 19.93)	2285	75467	30.28 (29.04 to 31.52)
	IMD=6	2481	59719.98	41.54 (39.91 to 43.18)	4437	72229	61.43 (59.62 to 63.24)	1232	65561.98	18.79 (17.74 to 19.84)	2351	72229	32.55 (31.23 to 33.86)
	IMD=7	2428	57972.54	41.88 (40.22 to 43.55)	4475	70526	63.45 (61.59 to 65.31)	1237	64003.84	19.33 (18.25 to 20.40)	2318	70526	32.87 (31.53 to 34.21)

	IMD=8	2082	46239.68	45.03 (43.09 to 46.96)	4021	57307	70.17 (68.00 to 72.33)	1108	51462.98	21.53 (20.26 to 22.80)	2181	57307	38.06 (36.46 to 39.66)
	IMD=9	2270	46356.83	48.97 (46.95 to 50.98)	4549	58642	77.57 (75.32 to 79.83)	1166	52314.02	22.29 (21.01 to 23.57)	2324	58642	39.63 (38.02 to 41.24)
	Most Deprivation	2073	40409.42	51.30 (49.09 to 53.51)	4473	52038	85.96 (83.44 to 88.48)	1025	46007.93	22.28 (20.91 to 23.64)	2247	52038	43.18 (41.39 to 44.97)
2013	Least Deprivation	2994	86015.17	34.81 (33.56 to 36.05)	4870	101493	47.98 (46.64 to 49.33)	1542	93836.46	16.43 (15.61 to 17.25)	2360	101493	23.25 (22.31 to 24.19)
	IMD=2	2896	79367.29	36.49 (35.16 to 37.82)	4782	94380	50.67 (49.23 to 52.10)	1539	86603.97	17.77 (16.88 to 18.66)	2537	94380	26.88 (25.83 to 27.93)
	IMD=3	2844	75278.54	37.78 (36.39 to 39.17)	4942	90358	54.69 (53.17 to 56.22)	1540	82577.09	18.65 (17.72 to 19.58)	2507	90358	27.75 (26.66 to 28.83)
	IMD=4	2556	66003.8	38.73 (37.22 to 40.23)	4337	79215	54.75 (53.12 to 56.38)	1353	72344.02	18.70 (17.71 to 19.70)	2242	79215	28.30 (27.13 to 29.47)
	IMD=5	2581	62957.04	41.00 (39.41 to 42.58)	4439	75876	58.50 (56.78 to 60.22)	1277	69026.99	18.50 (17.49 to 19.51)	2215	75876	29.19 (27.98 to 30.41)
	IMD=6	2483	59396.5	41.80 (40.16 to 43.45)	4485	72604	61.77 (59.97 to 63.58)	1376	65519.78	21.00 (19.89 to 22.11)	2369	72604	32.63 (31.32 to 33.94)
	IMD=7	2523	57660.74	43.76 (42.05 to 45.46)	4573	71097	64.32 (62.46 to 66.18)	1274	63896.49	19.94 (18.84 to 21.03)	2381	71097	33.49 (32.14 to 34.83)
	IMD=8	2099	46293.98	45.34 (43.40 to 47.28)	4129	58179	70.97 (68.81 to 73.14)	1162	51660.43	22.49 (21.20 to 23.79)	2211	58179	38.00 (36.42 to 39.59)
	IMD=9	2448	46585.97	52.55 (50.47 to 54.63)	4877	59874	81.45 (79.17 to 83.74)	1270	52878.1	24.02 (22.70 to 25.34)	2401	59874	40.10 (38.50 to 41.70)
	Most Deprivation	2258	40377.05	55.92 (53.62 to 58.23)	4810	52822	91.06 (88.49 to 93.63)	1206	46351.61	26.02 (24.55 to 27.49)	2373	52822	44.92 (43.12 to 46.73)
2014	Least Deprivation	3052	86476.59	35.29 (34.04 to 36.54)	4885	102139	47.83 (46.49 to 49.17)	1500	94408.2	15.89 (15.08 to 16.69)	2348	102139	22.99 (22.06 to 23.92)
	IMD=2	2846	79723.52	35.70 (34.39 to 37.01)	4644	94736	49.02 (47.61 to 50.43)	1552	86871.51	17.87 (16.98 to 18.75)	2505	94736	26.44 (25.41 to 27.48)
	IMD=3	2942	75139.05	39.15 (37.74 to 40.57)	4839	90118	53.70 (52.18 to 55.21)	1539	82400.28	18.68 (17.74 to 19.61)	2487	90118	27.60 (26.51 to 28.68)
	IMD=4	2532	66138.85	38.28 (36.79 to 39.77)	4326	79562	54.37 (52.75 to 55.99)	1396	72508.77	19.25 (18.24 to 20.26)	2247	79562	28.24 (27.07 to 29.41)
	IMD=5	2388	63378.8	37.68 (36.17 to 39.19)	4239	76449	55.45 (53.78 to 57.12)	1366	69602.23	19.63 (18.59 to 20.67)	2302	76449	30.11 (28.88 to 31.34)
	IMD=6	2499	59719.77	41.85 (40.20 to 43.49)	4524	73087	61.90 (60.10 to 63.70)	1337	66131.78	20.22 (19.13 to 21.30)	2400	73087	32.84 (31.52 to 34.15)
	IMD=7	2552	57951.55	44.04 (42.33 to 45.75)	4647	71583	64.92 (63.05 to 66.78)	1317	64548.82	20.40 (19.30 to 21.51)	2390	71583	33.39 (32.05 to 34.73)
	IMD=8	2166	46575.46	46.51 (44.55 to 48.46)	4203	58680	71.63 (69.46 to 73.79)	1167	52219.76	22.35 (21.07 to 23.63)	2174	58680	37.05 (35.49 to 38.61)
	IMD=9	2310	47248.86	48.89 (46.90 to 50.88)	4690	60780	77.16 (74.96 to 79.37)	1326	53805.02	24.64 (23.32 to 25.97)	2558	60780	42.09 (40.46 to 43.72)
	Most Deprivation	2160	40911.64	52.80 (50.57 to 55.02)	4653	53823	86.45 (83.97 to 88.93)	1188	47270.85	25.13 (23.70 to 26.56)	2403	53823	44.65 (42.86 to 46.43)
2015	Least Deprivation	2843	88259.02	32.21 (31.03 to 33.40)	4639	103744	44.72 (43.43 to 46.00)	1701	96059.22	17.71 (16.87 to 18.55)	2498	103744	24.08 (23.13 to 25.02)
	IMD=2	2940	80823.63	36.38 (35.06 to 37.69)	4709	95777	49.17 (47.76 to 50.57)	1680	87902.68	19.11 (18.20 to 20.03)	2661	95777	27.78 (26.73 to 28.84)
	IMD=3	2773	76344.82	36.32 (34.97 to 37.67)	4681	91212	51.32 (49.85 to 52.79)	1633	83504.87	19.56 (18.61 to 20.50)	2605	91212	28.56 (27.46 to 29.66)
	IMD=4	2477	67279.88	36.82 (35.37 to 38.27)	4233	80604	52.52 (50.93 to 54.10)	1478	73564.96	20.09 (19.07 to 21.12)	2381	80604	29.54 (28.35 to 30.73)
	IMD=5	2355	64712.48	36.39 (34.92 to 37.86)	4201	77954	53.89 (52.26 to 55.52)	1488	70937.32	20.98 (19.91 to 22.04)	2494	77954	31.99 (30.74 to 33.25)
	IMD=6	2406	60866.95	39.53 (37.95 to 41.11)	4370	74502	58.66 (56.92 to 60.40)	1369	67366.09	20.32 (19.25 to 21.40)	2438	74502	32.72 (31.42 to 34.02)
	IMD=7	2491	59194.99	42.08 (40.43 to 43.73)	4632	73062	63.40 (61.57 to 65.22)	1427	65924.6	21.65 (20.52 to 22.77)	2469	73062	33.79 (32.46 to 35.13)

	IMD=8	2157	48142.79	44.80 (42.91 to 46.70)	4198	60414	69.49 (67.39 to 71.59)	1221	53939.31	22.64 (21.37 to 23.91)	2367	60414	39.18 (37.60 to 40.76)
	IMD=9	2423	49065.46	49.38 (47.42 to 51.35)	4872	63065	77.25 (75.08 to 79.42)	1463	55793.22	26.22 (24.88 to 27.57)	2744	63065	43.51 (41.88 to 45.14)
	Most Deprivation	2194	42194.25	52.00 (49.82 to 54.17)	4704	55627	84.56 (82.15 to 86.98)	1268	48851.14	25.96 (24.53 to 27.39)	2528	55627	45.45 (43.67 to 47.22)
2016	Least Deprivation	2921	90446.97	32.30 (31.12 to 33.47)	4693	105932	44.30 (43.03 to 45.57)	1694	98027.3	17.28 (16.46 to 18.10)	2546	105932	24.03 (23.10 to 24.97)
	IMD=2	2948	82943.08	35.54 (34.26 to 36.83)	4721	97840	48.25 (46.88 to 49.63)	1633	89951.82	18.15 (17.27 to 19.03)	2639	97840	26.97 (25.94 to 28.00)
	IMD=3	2681	78474.4	34.16 (32.87 to 35.46)	4582	93206	49.16 (47.74 to 50.58)	1625	85440.14	19.02 (18.09 to 19.94)	2670	93206	28.65 (27.56 to 29.73)
	IMD=4	2511	69104.73	36.34 (34.91 to 37.76)	4218	82388	51.20 (49.65 to 52.74)	1469	75351.72	19.50 (18.50 to 20.49)	2445	82388	29.68 (28.50 to 30.85)
	IMD=5	2463	66856.21	36.84 (35.39 to 38.30)	4210	79978	52.64 (51.05 to 54.23)	1555	72853.77	21.34 (20.28 to 22.41)	2557	79978	31.97 (30.73 to 33.21)
	IMD=6	2553	63021.41	40.51 (38.94 to 42.08)	4463	76582	58.28 (56.57 to 59.99)	1545	69345.41	22.28 (21.17 to 23.39)	2615	76582	34.15 (32.84 to 35.46)
	IMD=7	2536	61458.95	41.26 (39.66 to 42.87)	4643	75530	61.47 (59.70 to 63.24)	1490	68278.15	21.82 (20.71 to 22.93)	2611	75530	34.57 (33.24 to 35.90)
	IMD=8	2211	50103.67	44.13 (42.29 to 45.97)	4235	62554	67.70 (65.66 to 69.74)	1322	55951.54	23.63 (22.35 to 24.90)	2439	62554	38.99 (37.44 to 40.54)
	IMD=9	2564	51142.83	50.13 (48.19 to 52.07)	5151	65513	78.63 (76.48 to 80.77)	1530	57999.34	26.38 (25.06 to 27.70)	2926	65513	44.66 (43.04 to 46.28)
	Most Deprivation	2377	43873.91	54.18 (52.00 to 56.36)	4821	57587	83.72 (81.35 to 86.08)	1363	50692.6	26.89 (25.46 to 28.32)	2739	57587	47.56 (45.78 to 49.34)
2017	Least Deprivation	3022	92887.75	32.53 (31.37 to 33.69)	4698	108488	43.30 (42.07 to 44.54)	1807	100175.2	18.04 (17.21 to 18.87)	2738	108488	25.24 (24.29 to 26.18)
	IMD=2	2865	85007.77	33.70 (32.47 to 34.94)	4517	100187	45.09 (43.77 to 46.40)	1796	91813.29	19.56 (18.66 to 20.47)	2807	100187	28.02 (26.98 to 29.05)
	IMD=3	2822	80989.16	34.84 (33.56 to 36.13)	4580	95909	47.75 (46.37 to 49.14)	1829	87634.5	20.87 (19.91 to 21.83)	2941	95909	30.66 (29.56 to 31.77)
	IMD=4	2532	71273.09	35.53 (34.14 to 36.91)	4185	84912	49.29 (47.79 to 50.78)	1542	77506.03	19.90 (18.90 to 20.89)	2510	84912	29.56 (28.40 to 30.72)
	IMD=5	2545	69012.66	36.88 (35.44 to 38.31)	4259	82510	51.62 (50.07 to 53.17)	1575	74864.34	21.04 (20.00 to 22.08)	2636	82510	31.95 (30.73 to 33.17)
	IMD=6	2547	65299.81	39.00 (37.49 to 40.52)	4443	79202	56.10 (54.45 to 57.75)	1504	71570.7	21.01 (19.95 to 22.08)	2634	79202	33.26 (31.99 to 34.53)
	IMD=7	2551	63922.25	39.91 (38.36 to 41.46)	4658	78293	59.49 (57.79 to 61.20)	1530	70581.58	21.68 (20.59 to 22.76)	2708	78293	34.59 (33.29 to 35.89)
	IMD=8	2276	52320.89	43.50 (41.71 to 45.29)	4219	65233	64.68 (62.72 to 66.63)	1437	58184.84	24.70 (23.42 to 25.97)	2598	65233	39.83 (38.30 to 41.36)
	IMD=9	2550	53537.31	47.63 (45.78 to 49.48)	5198	68662	75.70 (73.65 to 77.76)	1633	60407.6	27.03 (25.72 to 28.34)	3101	68662	45.16 (43.57 to 46.75)
	Most Deprivation	2370	45850.34	51.69 (49.61 to 53.77)	4847	59976	80.82 (78.54 to 83.09)	1445	52595.37	27.47 (26.06 to 28.89)	2851	59976	47.54 (45.79 to 49.28)
2018	Least Deprivation	2968	96286.2	30.82 (29.72 to 31.93)	4506	112107	40.19 (39.02 to 41.37)	1815	103233.7	17.58 (16.77 to 18.39)	2743	112107	24.47 (23.55 to 25.38)
	IMD=2	2771	88146.09	31.44 (30.27 to 32.61)	4406	103686	42.49 (41.24 to 43.75)	1861	94666.74	19.66 (18.77 to 20.55)	2887	103686	27.84 (26.83 to 28.86)
	IMD=3	2755	84778.99	32.50 (31.28 to 33.71)	4492	99804	45.01 (43.69 to 46.32)	1855	90817.38	20.43 (19.50 to 21.36)	3002	99804	30.08 (29.00 to 31.15)
	IMD=4	2543	74633.38	34.07 (32.75 to 35.40)	4162	88649	46.95 (45.52 to 48.38)	1585	80655.26	19.65 (18.68 to 20.62)	2604	88649	29.37 (28.25 to 30.50)
	IMD=5	2469	72452.13	34.08 (32.73 to 35.42)	4070	86154	47.24 (45.79 to 48.69)	1616	78117.49	20.69 (19.68 to 21.70)	2650	86154	30.76 (29.59 to 31.93)
	IMD=6	2473	68180.05	36.27 (34.84 to 37.70)	4322	82631	52.30 (50.75 to 53.86)	1595	74115.07	21.52 (20.46 to 22.58)	2777	82631	33.61 (32.36 to 34.86)
	IMD=7	2514	67350.23	37.33 (35.87 to 38.79)	4507	82374	54.71 (53.12 to 56.31)	1619	73742.09	21.95 (20.89 to 23.02)	2896	82374	35.16 (33.88 to 36.44)

	IMD=8	2374	55339.45	42.90 (41.17 to 44.62)	4370	68889	63.44 (61.55 to 65.32)	1413	61162.37	23.10 (21.90 to 24.31)	2626	68889	38.12 (36.66 to 39.58)
	IMD=9	2594	56786.61	45.68 (43.92 to 47.44)	5156	72573	71.05 (69.11 to 72.98)	1633	63512.56	25.71 (24.46 to 26.96)	3207	72573	44.19 (42.66 to 45.72)
	Most Deprivation	2450	48360.29	50.66 (48.66 to 52.67)	4930	62934	78.34 (76.15 to 80.52)	1515	54785.72	27.65 (26.26 to 29.05)	2923	62934	46.45 (44.76 to 48.13)
2019	Least Deprivation	3017	98576.73	30.61 (29.51 to 31.70)	4614	115528	39.94 (38.79 to 41.09)	1989	105003.6	18.94 (18.11 to 19.77)	2970	115528	25.71 (24.78 to 26.63)
	IMD=2	2855	89877.98	31.77 (30.60 to 32.93)	4450	106929	41.62 (40.39 to 42.84)	1978	95686.65	20.67 (19.76 to 21.58)	3113	106929	29.11 (28.09 to 30.14)
	IMD=3	2813	84890.78	33.14 (31.91 to 34.36)	4461	102698	43.44 (42.16 to 44.71)	1876	90427.45	20.75 (19.81 to 21.68)	3049	102698	29.69 (28.64 to 30.74)
	IMD=4	2574	74864.55	34.38 (33.05 to 35.71)	4137	91205	45.36 (43.98 to 46.74)	1774	80484.9	22.04 (21.02 to 23.07)	2838	91205	31.12 (29.97 to 32.26)
	IMD=5	2469	73875.38	33.42 (32.10 to 34.74)	4145	88943	46.60 (45.18 to 48.02)	1671	79126.95	21.12 (20.11 to 22.13)	2723	88943	30.62 (29.47 to 31.77)
	IMD=6	2574	69477.75	37.05 (35.62 to 38.48)	4338	85024	51.02 (49.50 to 52.54)	1717	75025.84	22.89 (21.80 to 23.97)	2951	85024	34.71 (33.46 to 35.96)
	IMD=7	2728	68552.79	39.79 (38.30 to 41.29)	4761	84441	56.38 (54.78 to 57.98)	1644	74444.13	22.08 (21.02 to 23.15)	2874	84441	34.04 (32.79 to 35.28)
	IMD=8	2362	56554.86	41.76 (40.08 to 43.45)	4280	71282	60.04 (58.24 to 61.84)	1544	62080.48	24.87 (23.63 to 26.11)	2789	71282	39.13 (37.67 to 40.58)
	IMD=9	2568	58438.75	43.94 (42.24 to 45.64)	5040	74765	67.41 (65.55 to 69.27)	1718	64943.29	26.45 (25.20 to 27.70)	3280	74765	43.87 (42.37 to 45.37)
	Most Deprivation	2394	48135.32	49.73 (47.74 to 51.73)	4684	62963	74.39 (72.26 to 76.52)	1434	54190.84	26.46 (25.09 to 27.83)	2922	62963	46.41 (44.73 to 48.09)
**65-74 years**													
2004	Least Deprivation	2027	55818.59	36.31 (34.73 to 37.89)	3132	64542	48.53 (46.83 to 50.23)	1687	57061.09	29.56 (28.15 to 30.98)	2926	64542	45.33 (43.69 to 46.98)
	IMD=2	1975	54368.2	36.33 (34.72 to 37.93)	3050	62827	48.55 (46.82 to 50.27)	1709	54976.97	31.09 (29.61 to 32.56)	3090	62827	49.18 (47.45 to 50.92)
	IMD=3	1988	52001.39	38.23 (36.55 to 39.91)	3025	59984	50.43 (48.63 to 52.23)	1718	52573.69	32.68 (31.13 to 34.22)	3079	59984	51.33 (49.52 to 53.14)
	IMD=4	1764	45071.18	39.14 (37.31 to 40.96)	2659	52224	50.92 (48.98 to 52.85)	1485	45585.86	32.58 (30.92 to 34.23)	2700	52224	51.70 (49.75 to 53.65)
	IMD=5	1625	43739.19	37.15 (35.35 to 38.96)	2559	50809	50.37 (48.41 to 52.32)	1322	44234.16	29.89 (28.28 to 31.50)	2608	50809	51.33 (49.36 to 53.30)
	IMD=6	1633	43655.68	37.41 (35.59 to 39.22)	2539	50565	50.21 (48.26 to 52.17)	1398	44022.46	31.76 (30.09 to 33.42)	2721	50565	53.81 (51.79 to 55.83)
	IMD=7	1729	41873.09	41.29 (39.35 to 43.24)	2620	48774	53.72 (51.66 to 55.77)	1344	42262.1	31.80 (30.10 to 33.50)	2622	48774	53.76 (51.70 to 55.82)
	IMD=8	1279	34374.57	37.21 (35.17 to 39.25)	2011	39942	50.35 (48.15 to 52.55)	1164	34628.07	33.61 (31.68 to 35.55)	2225	39942	55.71 (53.39 to 58.02)
	IMD=9	1376	35151.23	39.15 (37.08 to 41.21)	2178	40917	53.23 (50.99 to 55.47)	1129	35463.05	31.84 (29.98 to 33.69)	2171	40917	53.06 (50.83 to 55.29)
	Most Deprivation	1294	32303.19	40.06 (37.88 to 42.24)	2060	37612	54.77 (52.40 to 57.13)	1003	32590.83	30.78 (28.87 to 32.68)	1933	37612	51.39 (49.10 to 53.68)
2005	Least Deprivation	2125	55930.82	37.99 (36.38 to 39.61)	3443	65338	52.70 (50.94 to 54.46)	1588	57390.25	27.67 (26.31 to 29.03)	3029	65338	46.36 (44.71 to 48.01)
	IMD=2	2104	54024.72	38.95 (37.28 to 40.61)	3328	63053	52.78 (50.99 to 54.57)	1640	54881.79	29.88 (28.44 to 31.33)	3190	63053	50.59 (48.84 to 52.35)
	IMD=3	2055	51860.78	39.63 (37.91 to 41.34)	3296	60573	54.41 (52.56 to 56.27)	1699	52612.54	32.29 (30.76 to 33.83)	3195	60573	52.75 (50.92 to 54.58)
	IMD=4	1747	44763.09	39.03 (37.20 to 40.86)	2780	52594	52.86 (50.89 to 54.82)	1298	45611.51	28.46 (26.91 to 30.01)	2613	52594	49.68 (47.78 to 51.59)
	IMD=5	1662	43207.88	38.47 (36.62 to 40.31)	2746	50806	54.05 (52.03 to 56.07)	1386	43931.55	31.55 (29.89 to 33.21)	2751	50806	54.15 (52.12 to 56.17)
	IMD=6	1706	43024.62	39.65 (37.77 to 41.53)	2788	50661	55.03 (52.99 to 57.08)	1390	43534.42	31.93 (30.25 to 33.61)	2771	50661	54.70 (52.66 to 56.73)

	IMD=7	1681	41250.48	40.75 (38.80 to 42.70)	2725	48775	55.87 (53.77 to 57.97)	1305	41913.8	31.14 (29.45 to 32.82)	2720	48775	55.77 (53.67 to 57.86)
	IMD=8	1333	33552.46	39.73 (37.60 to 41.86)	2233	39607	56.38 (54.04 to 58.72)	1124	33871.38	33.18 (31.24 to 35.12)	2287	39607	57.74 (55.38 to 60.11)
	IMD=9	1378	34301.2	40.17 (38.05 to 42.29)	2332	40764	57.21 (54.89 to 59.53)	1121	34811.14	32.20 (30.32 to 34.09)	2319	40764	56.89 (54.57 to 59.20)
	Most Deprivation	1320	31115.03	42.42 (40.13 to 44.71)	2288	37227	61.46 (58.94 to 63.98)	1042	31781.11	32.79 (30.80 to 34.78)	2140	37227	57.49 (55.05 to 59.92)
2006	Least Deprivation	2225	56294.05	39.52 (37.88 to 41.17)	3710	66396	55.88 (54.08 to 57.67)	1583	58085.07	27.25 (25.91 to 28.60)	3069	66396	46.22 (44.59 to 47.86)
	IMD=2	2122	54217.46	39.14 (37.47 to 40.80)	3442	64032	53.75 (51.96 to 55.55)	1498	55535.73	26.97 (25.61 to 28.34)	3105	64032	48.49 (46.79 to 50.20)
	IMD=3	2027	52053.75	38.94 (37.25 to 40.64)	3378	61627	54.81 (52.97 to 56.66)	1499	53149.65	28.20 (26.78 to 29.63)	3044	61627	49.39 (47.64 to 51.15)
	IMD=4	1757	44851.11	39.17 (37.34 to 41.01)	2927	53319	54.90 (52.91 to 56.88)	1318	45946.66	28.69 (27.14 to 30.23)	2636	53319	49.44 (47.55 to 51.33)
	IMD=5	1696	43532.89	38.96 (37.10 to 40.81)	2918	51777	56.36 (54.31 to 58.40)	1249	44529.09	28.05 (26.49 to 29.60)	2655	51777	51.28 (49.33 to 53.23)
	IMD=6	1761	42819.12	41.13 (39.21 to 43.05)	2971	51174	58.06 (55.97 to 60.14)	1274	43615.39	29.21 (27.61 to 30.81)	2704	51174	52.84 (50.85 to 54.83)
	IMD=7	1638	40750.46	40.20 (38.25 to 42.14)	2845	49092	57.95 (55.82 to 60.08)	1225	41726.46	29.36 (27.71 to 31.00)	2679	49092	54.57 (52.50 to 56.64)
	IMD=8	1335	32745.68	40.77 (38.58 to 42.96)	2291	39440	58.09 (55.71 to 60.47)	974	33366.14	29.19 (27.36 to 31.02)	2139	39440	54.23 (51.94 to 56.53)
	IMD=9	1524	33580.75	45.38 (43.10 to 47.66)	2577	40579	63.51 (61.05 to 65.96)	1075	34445.53	31.21 (29.34 to 33.07)	2235	40579	55.08 (52.79 to 57.36)
	Most Deprivation	1354	30232.57	44.79 (42.40 to 47.17)	2408	36746	65.53 (62.91 to 68.15)	917	31111.26	29.47 (27.57 to 31.38)	2070	36746	56.33 (53.91 to 58.76)
2007	Least Deprivation	2211	57458.86	38.48 (36.88 to 40.08)	3697	68339	54.10 (52.35 to 55.84)	1514	59698.4	25.36 (24.08 to 26.64)	3058	68339	44.75 (43.16 to 46.33)
	IMD=2	2172	55310.89	39.27 (37.62 to 40.92)	3646	65885	55.34 (53.54 to 57.14)	1575	57155.2	27.56 (26.20 to 28.92)	3171	65885	48.13 (46.45 to 49.80)
	IMD=3	2164	52608.01	41.13 (39.40 to 42.87)	3584	62860	57.02 (55.15 to 58.88)	1545	54212.58	28.50 (27.08 to 29.92)	3105	62860	49.40 (47.66 to 51.13)
	IMD=4	1876	45463.17	41.26 (39.40 to 43.13)	3100	54498	56.88 (54.88 to 58.89)	1322	46893.78	28.19 (26.67 to 29.71)	2717	54498	49.86 (47.98 to 51.73)
	IMD=5	1776	44204.68	40.18 (38.31 to 42.05)	3052	52991	57.59 (55.55 to 59.64)	1302	45461.7	28.64 (27.08 to 30.20)	2737	52991	51.65 (49.72 to 53.59)
	IMD=6	1831	42982.46	42.60 (40.65 to 44.55)	3110	51784	60.06 (57.95 to 62.17)	1362	44205.59	30.81 (29.17 to 32.45)	2803	51784	54.13 (52.12 to 56.13)
	IMD=7	1663	41006.3	40.55 (38.61 to 42.50)	2918	49536	58.91 (56.77 to 61.04)	1247	42283.74	29.49 (27.85 to 31.13)	2742	49536	55.35 (53.28 to 57.43)
	IMD=8	1398	32620.84	42.86 (40.61 to 45.10)	2387	39510	60.42 (57.99 to 62.84)	1033	33542.29	30.80 (28.92 to 32.68)	2188	39510	55.38 (53.06 to 57.70)
	IMD=9	1435	33212.18	43.21 (40.97 to 45.44)	2607	40692	64.07 (61.61 to 66.53)	1083	34407.06	31.48 (29.60 to 33.35)	2348	40692	57.70 (55.37 to 60.04)
	Most Deprivation	1326	29515.96	44.92 (42.51 to 47.34)	2480	36496	67.95 (65.28 to 70.63)	952	30762.79	30.95 (28.98 to 32.91)	2098	36496	57.49 (55.03 to 59.95)
2008	Least Deprivation	2311	59656.91	38.74 (37.16 to 40.32)	3909	70862	55.16 (53.43 to 56.89)	1618	62315.56	25.96 (24.70 to 27.23)	3125	70862	44.10 (42.55 to 45.65)
	IMD=2	2322	57471.63	40.40 (38.76 to 42.05)	3965	68431	57.94 (56.14 to 59.75)	1682	59653.14	28.20 (26.85 to 29.54)	3362	68431	49.13 (47.47 to 50.79)
	IMD=3	2196	54769.81	40.10 (38.42 to 41.77)	3771	65329	57.72 (55.88 to 59.57)	1698	56682.92	29.96 (28.53 to 31.38)	3318	65329	50.79 (49.06 to 52.52)
	IMD=4	1860	46946.68	39.62 (37.82 to 41.42)	3177	56146	56.58 (54.62 to 58.55)	1433	48622.57	29.47 (27.95 to 31.00)	2831	56146	50.42 (48.56 to 52.28)
	IMD=5	1897	45506.03	41.69 (39.81 to 43.56)	3270	54523	59.97 (57.92 to 62.03)	1419	47042.66	30.16 (28.59 to 31.73)	2963	54523	54.34 (52.39 to 56.30)
	IMD=6	1811	43920.19	41.23 (39.33 to 43.13)	3203	53131	60.28 (58.20 to 62.37)	1419	45443.42	31.23 (29.60 to 32.85)	2951	53131	55.54 (53.54 to 57.55)

	IMD=7	1614	42090.74	38.35 (36.47 to 40.22)	3004	50900	59.02 (56.91 to 61.13)	1288	43594.27	29.55 (27.93 to 31.16)	2813	50900	55.27 (53.22 to 57.31)
	IMD=8	1409	32990.89	42.71 (40.48 to 44.94)	2450	40117	61.07 (58.65 to 63.49)	1072	34130.63	31.41 (29.53 to 33.29)	2285	40117	56.96 (54.62 to 59.29)
	IMD=9	1521	33365.51	45.59 (43.30 to 47.88)	2757	41075	67.12 (64.62 to 69.63)	1081	34755.31	31.10 (29.25 to 32.96)	2385	41075	58.06 (55.73 to 60.39)
	Most Deprivation	1344	29290.86	45.88 (43.43 to 48.34)	2569	36515	70.35 (67.63 to 73.08)	986	30729.47	32.09 (30.08 to 34.09)	2254	36515	61.73 (59.18 to 64.28)
2009	Least Deprivation	2364	62299.44	37.95 (36.42 to 39.48)	4030	74249	54.28 (52.60 to 55.95)	1719	65266.43	26.34 (25.09 to 27.58)	3288	74249	44.28 (42.77 to 45.80)
	IMD=2	2363	59883.8	39.46 (37.87 to 41.05)	4065	71607	56.77 (55.02 to 58.51)	1795	62310.82	28.81 (27.47 to 30.14)	3507	71607	48.98 (47.35 to 50.60)
	IMD=3	2427	57193.19	42.44 (40.75 to 44.12)	4094	68622	59.66 (57.83 to 61.49)	1781	59420.77	29.97 (28.58 to 31.36)	3574	68622	52.08 (50.37 to 53.79)
	IMD=4	2026	48746.91	41.56 (39.75 to 43.37)	3407	58586	58.15 (56.20 to 60.11)	1458	50615.95	28.81 (27.33 to 30.28)	2952	58586	50.39 (48.57 to 52.21)
	IMD=5	1903	46858.59	40.61 (38.79 to 42.44)	3330	56466	58.97 (56.97 to 60.98)	1449	48673.21	29.77 (28.24 to 31.30)	3016	56466	53.41 (51.51 to 55.32)
	IMD=6	1933	44643.79	43.30 (41.37 to 45.23)	3408	54417	62.63 (60.52 to 64.73)	1491	46413.02	32.12 (30.49 to 33.76)	3002	54417	55.17 (53.19 to 57.14)
	IMD=7	1841	42954.31	42.86 (40.90 to 44.82)	3323	52186	63.68 (61.51 to 65.84)	1288	44661.49	28.84 (27.26 to 30.41)	2850	52186	54.61 (52.61 to 56.62)
	IMD=8	1439	33365.89	43.13 (40.90 to 45.36)	2597	40796	63.66 (61.21 to 66.11)	1110	34560.52	32.12 (30.23 to 34.01)	2379	40796	58.31 (55.97 to 60.66)
	IMD=9	1597	33104.29	48.24 (45.88 to 50.61)	2877	41397	69.50 (66.96 to 72.04)	1152	34824.95	33.08 (31.17 to 34.99)	2516	41397	60.78 (58.40 to 63.15)
	Most Deprivation	1403	28908.21	48.53 (45.99 to 51.07)	2723	36510	74.58 (71.78 to 77.38)	935	30604.96	30.55 (28.59 to 32.51)	2246	36510	61.52 (58.97 to 64.06)
2010	Least Deprivation	2525	64450.72	39.18 (37.65 to 40.71)	4246	76804	55.28 (53.62 to 56.95)	1751	67546.7	25.92 (24.71 to 27.14)	3283	76804	42.75 (41.28 to 44.21)
	IMD=2	2439	61847.47	39.44 (37.87 to 41.00)	4155	74122	56.06 (54.35 to 57.76)	1812	64347.32	28.16 (26.86 to 29.46)	3592	74122	48.46 (46.88 to 50.05)
	IMD=3	2415	58568.58	41.23 (39.59 to 42.88)	4180	70438	59.34 (57.54 to 61.14)	1768	60912.72	29.03 (27.67 to 30.38)	3611	70438	51.26 (49.59 to 52.94)
	IMD=4	2036	50111.55	40.63 (38.86 to 42.39)	3529	60517	58.31 (56.39 to 60.24)	1533	52244.25	29.34 (27.87 to 30.81)	3058	60517	50.53 (48.74 to 52.32)
	IMD=5	1899	47974.16	39.58 (37.80 to 41.36)	3415	57928	58.95 (56.98 to 60.93)	1447	49854.43	29.02 (27.53 to 30.52)	3015	57928	52.05 (50.19 to 53.91)
	IMD=6	1877	45560.78	41.20 (39.33 to 43.06)	3390	55522	61.06 (59.00 to 63.11)	1436	47357.45	30.32 (28.75 to 31.89)	3019	55522	54.37 (52.44 to 56.31)
	IMD=7	1808	43600.36	41.47 (39.56 to 43.38)	3253	53146	61.21 (59.11 to 63.31)	1418	45333.93	31.28 (29.65 to 32.91)	2978	53146	56.03 (54.02 to 58.05)
	IMD=8	1435	33599.25	42.71 (40.50 to 44.92)	2590	41209	62.85 (60.43 to 65.27)	1103	34884.14	31.62 (29.75 to 33.48)	2421	41209	58.75 (56.41 to 61.09)
	IMD=9	1558	32978.1	47.24 (44.90 to 49.59)	2940	41547	70.76 (68.21 to 73.32)	1153	34797.22	33.13 (31.22 to 35.05)	2576	41547	62.00 (59.61 to 64.40)
	Most Deprivation	1352	28609.13	47.26 (44.74 to 49.78)	2718	36285	74.91 (72.09 to 77.72)	973	30362.91	32.05 (30.03 to 34.06)	2266	36285	62.45 (59.88 to 65.02)
2011	Least Deprivation	2713	67898.71	39.96 (38.45 to 41.46)	4628	81020	57.12 (55.48 to 58.77)	1812	71222.41	25.44 (24.27 to 26.61)	3397	81020	41.93 (40.52 to 43.34)
	IMD=2	2560	64820.49	39.49 (37.96 to 41.02)	4414	77660	56.84 (55.16 to 58.51)	1829	67557.16	27.07 (25.83 to 28.31)	3560	77660	45.84 (44.33 to 47.35)
	IMD=3	2532	61371.27	41.26 (39.65 to 42.86)	4482	73998	60.57 (58.80 to 62.34)	1831	63991.48	28.61 (27.30 to 29.92)	3689	73998	49.85 (48.24 to 51.46)
	IMD=4	2202	52541.79	41.91 (40.16 to 43.66)	3879	63529	61.06 (59.14 to 62.98)	1588	54854.01	28.95 (27.53 to 30.37)	3184	63529	50.12 (48.38 to 51.86)
	IMD=5	2090	49852.92	41.92 (40.13 to 43.72)	3593	60333	59.55 (57.61 to 61.50)	1486	51813.23	28.68 (27.22 to 30.14)	3121	60333	51.73 (49.91 to 53.54)
	IMD=6	2029	47413.55	42.79 (40.93 to 44.66)	3622	57686	62.79 (60.74 to 64.83)	1434	49376.77	29.04 (27.54 to 30.55)	3119	57686	54.07 (52.17 to 55.97)

	IMD=7	1903	45132.87	42.16 (40.27 to 44.06)	3510	55060	63.75 (61.64 to 65.86)	1409	46988.51	29.99 (28.42 to 31.55)	3008	55060	54.63 (52.68 to 56.58)
	IMD=8	1593	34573.89	46.08 (43.81 to 48.34)	2856	42575	67.08 (64.62 to 69.54)	1143	35912.83	31.83 (29.98 to 33.67)	2481	42575	58.27 (55.98 to 60.57)
	IMD=9	1590	33536.33	47.41 (45.08 to 49.74)	3042	42335	71.86 (69.30 to 74.41)	1153	35404.47	32.57 (30.69 to 34.45)	2632	42335	62.17 (59.80 to 64.55)
	Most Deprivation	1487	28650.34	51.90 (49.26 to 54.54)	2880	36612	78.66 (75.79 to 81.54)	983	30482.5	32.25 (30.23 to 34.26)	2325	36612	63.50 (60.92 to 66.09)
2012	Least Deprivation	2808	72068.34	38.96 (37.52 to 40.40)	4769	85818	55.57 (53.99 to 57.15)	1921	75799.64	25.34 (24.21 to 26.48)	3498	85818	40.76 (39.41 to 42.11)
	IMD=2	2681	68460.91	39.16 (37.68 to 40.64)	4640	81727	56.77 (55.14 to 58.41)	1920	71387.66	26.90 (25.69 to 28.10)	3668	81727	44.88 (43.43 to 46.33)
	IMD=3	2710	64542.57	41.99 (40.41 to 43.57)	4653	77759	59.84 (58.12 to 61.56)	1933	67496.22	28.64 (27.36 to 29.92)	3815	77759	49.06 (47.50 to 50.62)
	IMD=4	2235	55547.61	40.24 (38.57 to 41.90)	3957	66975	59.08 (57.24 to 60.92)	1637	58037.23	28.21 (26.84 to 29.57)	3160	66975	47.18 (45.54 to 48.83)
	IMD=5	2149	52314.57	41.08 (39.34 to 42.82)	3742	63188	59.22 (57.32 to 61.12)	1525	54631.04	27.91 (26.51 to 29.32)	3114	63188	49.28 (47.55 to 51.01)
	IMD=6	2105	49424.84	42.59 (40.77 to 44.41)	3811	60233	63.27 (61.26 to 65.28)	1525	51749.48	29.47 (27.99 to 30.95)	3221	60233	53.48 (51.63 to 55.32)
	IMD=7	1891	47022.12	40.22 (38.40 to 42.03)	3564	57289	62.21 (60.17 to 64.25)	1475	49109.85	30.03 (28.50 to 31.57)	3126	57289	54.57 (52.65 to 56.48)
	IMD=8	1590	35732.82	44.50 (42.31 to 46.68)	2928	43978	66.58 (64.17 to 68.99)	1143	37164.98	30.75 (28.97 to 32.54)	2486	43978	56.53 (54.31 to 58.75)
	IMD=9	1672	34502.52	48.46 (46.14 to 50.78)	3234	43703	74.00 (71.45 to 76.55)	1130	36678.91	30.81 (29.01 to 32.60)	2567	43703	58.74 (56.47 to 61.01)
	Most Deprivation	1534	29332.37	52.30 (49.68 to 54.91)	2993	37552	79.70 (76.85 to 82.56)	953	31324.64	30.42 (28.49 to 32.35)	2350	37552	62.58 (60.05 to 65.11)
2013	Least Deprivation	2852	74025.13	38.53 (37.11 to 39.94)	4823	88476	54.51 (52.97 to 56.05)	2045	78013.77	26.21 (25.08 to 27.35)	3579	88476	40.45 (39.13 to 41.78)
	IMD=2	2755	70374.23	39.15 (37.69 to 40.61)	4695	84420	55.61 (54.02 to 57.21)	2015	73475.94	27.42 (26.23 to 28.62)	3776	84420	44.73 (43.30 to 46.16)
	IMD=3	2676	66217.2	40.41 (38.88 to 41.94)	4649	80162	58.00 (56.33 to 59.66)	2030	69374.81	29.26 (27.99 to 30.53)	3950	80162	49.28 (47.74 to 50.81)
	IMD=4	2439	57061.28	42.74 (41.05 to 44.44)	4102	69183	59.29 (57.48 to 61.11)	1706	59727.41	28.56 (27.21 to 29.92)	3257	69183	47.08 (45.46 to 48.69)
	IMD=5	2155	53645.56	40.17 (38.48 to 41.87)	3752	65016	57.71 (55.86 to 59.56)	1670	56016.24	29.81 (28.38 to 31.24)	3157	65016	48.56 (46.86 to 50.25)
	IMD=6	2185	50316.6	43.43 (41.60 to 45.25)	3864	61541	62.79 (60.81 to 64.77)	1591	52682.56	30.20 (28.72 to 31.68)	3185	61541	51.75 (49.96 to 53.55)
	IMD=7	2039	47856.01	42.61 (40.76 to 44.46)	3634	58701	61.91 (59.89 to 63.92)	1519	49980.06	30.39 (28.86 to 31.92)	3101	58701	52.83 (50.97 to 54.69)
	IMD=8	1619	36239.36	44.68 (42.50 to 46.85)	2981	45044	66.18 (63.80 to 68.56)	1202	37883.57	31.73 (29.94 to 33.52)	2574	45044	57.14 (54.94 to 59.35)
	IMD=9	1692	35183.61	48.09 (45.80 to 50.38)	3260	44595	73.10 (70.59 to 75.61)	1238	37308.94	33.18 (31.33 to 35.03)	2637	44595	59.13 (56.88 to 61.39)
	Most Deprivation	1477	29470.99	50.12 (47.56 to 52.67)	3015	38132	79.07 (76.25 to 81.89)	1024	31615.72	32.39 (30.41 to 34.37)	2321	38132	60.87 (58.39 to 63.34)
2014	Least Deprivation	2861	75569.12	37.86 (36.47 to 39.25)	4760	90307	52.71 (51.21 to 54.21)	2238	79626.57	28.11 (26.94 to 29.27)	3817	90307	42.27 (40.93 to 43.61)
	IMD=2	2860	71539.03	39.98 (38.51 to 41.44)	4741	85963	55.15 (53.58 to 56.72)	2145	74997.8	28.60 (27.39 to 29.81)	3900	85963	45.37 (43.94 to 46.79)
	IMD=3	2849	68021.53	41.88 (40.35 to 43.42)	4838	82321	58.77 (57.11 to 60.43)	2107	71200.41	29.59 (28.33 to 30.86)	3992	82321	48.49 (46.99 to 50.00)
	IMD=4	2429	58304.74	41.66 (40.00 to 43.32)	4181	70844	59.02 (57.23 to 60.81)	1839	61270.73	30.01 (28.64 to 31.39)	3411	70844	48.15 (46.53 to 49.76)
	IMD=5	2231	54631.35	40.84 (39.14 to 42.53)	3887	66176	58.74 (56.89 to 60.58)	1739	56993.99	30.51 (29.08 to 31.95)	3257	66176	49.22 (47.53 to 50.91)
	IMD=6	2180	51223.76	42.56 (40.77 to 44.34)	3884	62705	61.94 (59.99 to 63.89)	1691	53706.34	31.49 (29.99 to 32.99)	3237	62705	51.62 (49.84 to 53.40)

	IMD=7	2077	48662.05	42.68 (40.85 to 44.52)	3768	59657	63.16 (61.14 to 65.18)	1570	50967.07	30.80 (29.28 to 32.33)	3146	59657	52.73 (50.89 to 54.58)
	IMD=8	1653	36868.81	44.83 (42.67 to 47.00)	3001	45858	65.44 (63.10 to 67.78)	1233	38719.32	31.84 (30.07 to 33.62)	2539	45858	55.37 (53.21 to 57.52)
	IMD=9	1595	35600.54	44.80 (42.60 to 47.00)	3096	45035	68.75 (66.32 to 71.17)	1295	37816.43	34.24 (32.38 to 36.11)	2588	45035	57.47 (55.25 to 59.68)
	Most Deprivation	1456	29970.86	48.58 (46.09 to 51.08)	2885	38476	74.98 (72.25 to 77.72)	1134	32020.88	35.41 (33.35 to 37.48)	2417	38476	62.82 (60.31 to 65.32)
2015	Least Deprivation	2878	77313.33	37.23 (35.87 to 38.59)	4742	91994	51.55 (50.08 to 53.01)	2364	80996.66	29.19 (28.01 to 30.36)	3973	91994	43.19 (41.84 to 44.53)
	IMD=2	2869	73356.3	39.11 (37.68 to 40.54)	4743	87871	53.98 (52.44 to 55.51)	2345	76598.65	30.61 (29.38 to 31.85)	4097	87871	46.63 (45.20 to 48.05)
	IMD=3	2691	69506.7	38.72 (37.25 to 40.18)	4535	83868	54.07 (52.50 to 55.65)	2228	72560.18	30.71 (29.43 to 31.98)	4101	83868	48.90 (47.40 to 50.39)
	IMD=4	2344	59677.09	39.28 (37.69 to 40.87)	3999	72308	55.31 (53.59 to 57.02)	1918	62587.32	30.65 (29.27 to 32.02)	3486	72308	48.21 (46.61 to 49.81)
	IMD=5	2296	55855.98	41.11 (39.42 to 42.79)	3889	67606	57.52 (55.72 to 59.33)	1814	58194.37	31.17 (29.74 to 32.61)	3366	67606	49.79 (48.11 to 51.47)
	IMD=6	2206	52395.91	42.10 (40.35 to 43.86)	3804	64203	59.25 (57.37 to 61.13)	1813	54736.28	33.12 (31.60 to 34.65)	3504	64203	54.58 (52.77 to 56.38)
	IMD=7	2102	50098.77	41.96 (40.16 to 43.75)	3614	61323	58.93 (57.01 to 60.86)	1700	52360.45	32.47 (30.92 to 34.01)	3279	61323	53.47 (51.64 to 55.30)
	IMD=8	1652	37834.27	43.66 (41.56 to 45.77)	3031	46943	64.57 (62.27 to 66.87)	1384	39642.41	34.91 (33.07 to 36.75)	2691	46943	57.32 (55.16 to 59.49)
	IMD=9	1728	36376.54	47.50 (45.26 to 49.74)	3234	46017	70.28 (67.86 to 72.70)	1317	38515.01	34.19 (32.35 to 36.04)	2722	46017	59.15 (56.93 to 61.37)
	Most Deprivation	1494	30753.31	48.58 (46.12 to 51.04)	2918	39436	73.99 (71.31 to 76.68)	1160	32736.85	35.43 (33.39 to 37.47)	2464	39436	62.48 (60.01 to 64.95)
2016	Least Deprivation	2939	79473.12	36.98 (35.64 to 38.32)	4684	94201	49.72 (48.30 to 51.15)	2433	82748.36	29.40 (28.23 to 30.57)	4116	94201	43.69 (42.36 to 45.03)
	IMD=2	2805	75265.23	37.27 (35.89 to 38.65)	4608	89690	51.38 (49.89 to 52.86)	2481	78152.28	31.75 (30.50 to 32.99)	4263	89690	47.53 (46.10 to 48.96)
	IMD=3	2663	71535.2	37.23 (35.81 to 38.64)	4493	85786	52.37 (50.84 to 53.91)	2381	74176.82	32.10 (30.81 to 33.39)	4272	85786	49.80 (48.31 to 51.29)
	IMD=4	2355	61415.02	38.35 (36.80 to 39.89)	3923	73916	53.07 (51.41 to 54.73)	2040	63959.29	31.90 (30.51 to 33.28)	3606	73916	48.79 (47.19 to 50.38)
	IMD=5	2313	57303.18	40.36 (38.72 to 42.01)	3839	69092	55.56 (53.81 to 57.32)	1866	59526.73	31.35 (29.92 to 32.77)	3405	69092	49.28 (47.63 to 50.94)
	IMD=6	2179	53674.66	40.60 (38.89 to 42.30)	3759	65315	57.55 (55.71 to 59.39)	1834	55870.7	32.83 (31.32 to 34.33)	3581	65315	54.83 (53.03 to 56.62)
	IMD=7	2048	51285.35	39.93 (38.20 to 41.66)	3580	62493	57.29 (55.41 to 59.16)	1718	53456.33	32.14 (30.62 to 33.66)	3323	62493	53.17 (51.37 to 54.98)
	IMD=8	1697	39027.67	43.48 (41.41 to 45.55)	3008	48132	62.49 (60.26 to 64.73)	1414	40696.82	34.74 (32.93 to 36.56)	2765	48132	57.45 (55.30 to 59.59)
	IMD=9	1694	37433.08	45.25 (43.10 to 47.41)	3248	47008	69.09 (66.72 to 71.47)	1340	39474.19	33.95 (32.13 to 35.76)	2814	47008	59.86 (57.65 to 62.07)
	Most Deprivation	1540	31636.73	48.68 (46.25 to 51.11)	2927	40275	72.68 (70.04 to 75.31)	1226	33435.7	36.67 (34.61 to 38.72)	2537	40275	62.99 (60.54 to 65.44)
2017	Least Deprivation	2860	80670.78	35.45 (34.15 to 36.75)	4549	95264	47.75 (46.36 to 49.14)	2422	83359.93	29.05 (27.90 to 30.21)	4197	95264	44.06 (42.72 to 45.39)
	IMD=2	2774	76285.33	36.36 (35.01 to 37.72)	4419	90595	48.78 (47.34 to 50.22)	2493	78601.24	31.72 (30.47 to 32.96)	4281	90595	47.25 (45.84 to 48.67)
	IMD=3	2624	72865.8	36.01 (34.63 to 37.39)	4267	86977	49.06 (47.59 to 50.53)	2477	74979.97	33.04 (31.73 to 34.34)	4408	86977	50.68 (49.18 to 52.18)
	IMD=4	2342	62724.38	37.34 (35.83 to 38.85)	3816	75220	50.73 (49.12 to 52.34)	2041	64886.45	31.45 (30.09 to 32.82)	3659	75220	48.64 (47.07 to 50.22)
	IMD=5	2192	58453.74	37.50 (35.93 to 39.07)	3642	70380	51.75 (50.07 to 53.43)	1854	60429.71	30.68 (29.28 to 32.08)	3461	70380	49.18 (47.54 to 50.81)
	IMD=6	2170	54767.46	39.62 (37.95 to 41.29)	3654	66580	54.88 (53.10 to 56.66)	1925	56620.93	34.00 (32.48 to 35.52)	3625	66580	54.45 (52.67 to 56.22)

	IMD=7	2008	52180.2	38.48 (36.80 to 40.17)	3417	63433	53.87 (52.06 to 55.67)	1725	54033.12	31.92 (30.42 to 33.43)	3288	63433	51.83 (50.06 to 53.61)
	IMD=8	1777	39734.96	44.72 (42.64 to 46.80)	2999	49063	61.13 (58.94 to 63.31)	1416	41380.14	34.22 (32.44 to 36.00)	2750	49063	56.05 (53.96 to 58.15)
	IMD=9	1726	38392.6	44.96 (42.84 to 47.08)	3173	48037	66.05 (63.75 to 68.35)	1363	40034.71	34.05 (32.24 to 35.85)	2867	48037	59.68 (57.50 to 61.87)
	Most Deprivation	1566	32316.23	48.46 (46.06 to 50.86)	2845	41111	69.20 (66.66 to 71.75)	1291	33928.25	38.05 (35.98 to 40.13)	2676	41111	65.09 (62.63 to 67.56)
2018	Least Deprivation	2825	82024.05	34.44 (33.17 to 35.71)	4365	96425	45.27 (43.93 to 46.61)	2567	84100.71	30.52 (29.34 to 31.70)	4229	96425	43.86 (42.54 to 45.18)
	IMD=2	2694	78018.58	34.53 (33.23 to 35.83)	4289	92571	46.33 (44.95 to 47.72)	2492	79735.32	31.25 (30.03 to 32.48)	4430	92571	47.86 (46.45 to 49.26)
	IMD=3	2636	75118.2	35.09 (33.75 to 36.43)	4194	89087	47.08 (45.65 to 48.50)	2568	76447.34	33.59 (32.29 to 34.89)	4548	89087	51.05 (49.57 to 52.53)
	IMD=4	2335	64800.04	36.03 (34.57 to 37.50)	3766	77379	48.67 (47.12 to 50.22)	2169	66342.83	32.69 (31.32 to 34.07)	3781	77379	48.86 (47.31 to 50.42)
	IMD=5	2028	60598.53	33.47 (32.01 to 34.92)	3425	72327	47.35 (45.77 to 48.94)	2072	62065.58	33.38 (31.95 to 34.82)	3620	72327	50.05 (48.42 to 51.68)
	IMD=6	2187	56111.04	38.98 (37.34 to 40.61)	3608	68142	52.95 (51.22 to 54.68)	1944	57557.9	33.77 (32.27 to 35.28)	3695	68142	54.23 (52.48 to 55.97)
	IMD=7	2024	53626.05	37.74 (36.10 to 39.39)	3346	65183	51.33 (49.59 to 53.07)	1848	55108.07	33.53 (32.01 to 35.06)	3438	65183	52.74 (50.98 to 54.51)
	IMD=8	1650	40662.31	40.58 (38.62 to 42.54)	2929	50357	58.16 (56.06 to 60.27)	1490	42115.14	35.38 (33.58 to 37.18)	2934	50357	58.26 (56.16 to 60.37)
	IMD=9	1652	39434.58	41.89 (39.87 to 43.91)	3065	49502	61.92 (59.72 to 64.11)	1470	40976.99	35.87 (34.04 to 37.71)	2925	49502	59.09 (56.95 to 61.23)
	Most Deprivation	1571	33109.83	47.45 (45.10 to 49.79)	2886	42352	68.14 (65.66 to 70.63)	1238	34708.45	35.67 (33.68 to 37.66)	2666	42352	62.95 (60.56 to 65.34)
2019	Least Deprivation	2746	81047.34	33.88 (32.61 to 35.15)	4161	96101	43.30 (41.98 to 44.61)	2663	82556.87	32.26 (31.03 to 33.48)	4461	96101	46.42 (45.06 to 47.78)
	IMD=2	2697	76664.23	35.18 (33.85 to 36.51)	4181	92205	45.34 (43.97 to 46.72)	2566	77926.79	32.93 (31.65 to 34.20)	4441	92205	48.16 (46.75 to 49.58)
	IMD=3	2561	72798.84	35.18 (33.82 to 36.54)	3985	88805	44.87 (43.48 to 46.27)	2437	73539.98	33.14 (31.82 to 34.45)	4399	88805	49.54 (48.07 to 51.00)
	IMD=4	2302	63292.39	36.37 (34.89 to 37.86)	3652	77307	47.24 (45.71 to 48.77)	2214	64286.21	34.44 (33.01 to 35.87)	3910	77307	50.58 (48.99 to 52.16)
	IMD=5	2115	60340.59	35.05 (33.56 to 36.54)	3432	73065	46.97 (45.40 to 48.54)	2175	61268.54	35.50 (34.01 to 36.99)	3763	73065	51.50 (49.86 to 53.15)
	IMD=6	2112	55340.66	38.16 (36.54 to 39.79)	3528	68173	51.75 (50.04 to 53.46)	1931	56525.63	34.16 (32.64 to 35.69)	3605	68173	52.88 (51.15 to 54.61)
	IMD=7	2024	53087.86	38.13 (36.46 to 39.79)	3376	65201	51.78 (50.03 to 53.52)	1855	54236.92	34.20 (32.65 to 35.76)	3442	65201	52.79 (51.03 to 54.55)
	IMD=8	1574	40029.17	39.32 (37.38 to 41.26)	2790	50365	55.40 (53.34 to 57.45)	1468	41189.33	35.64 (33.82 to 37.46)	2829	50365	56.17 (54.10 to 58.24)
	IMD=9	1641	39487.53	41.56 (39.55 to 43.57)	2950	49739	59.31 (57.17 to 61.45)	1479	40801.19	36.25 (34.40 to 38.10)	2898	49739	58.26 (56.14 to 60.39)
	Most Deprivation	1475	32504.05	45.38 (43.06 to 47.69)	2730	41883	65.18 (62.74 to 67.63)	1216	33860.16	35.91 (33.89 to 37.93)	2600	41883	62.08 (59.69 to 64.46)
**75-84 years**													
2004	Least Deprivation	1561	35846.9	43.55 (41.39 to 45.71)	2439	42587	57.27 (55.00 to 59.54)	1278	35765.93	35.73 (33.77 to 37.69)	2498	42587	58.66 (56.36 to 60.96)
	IMD=2	1473	36513.2	40.34 (38.28 to 42.40)	2339	43358	53.95 (51.76 to 56.13)	1373	36001.33	38.14 (36.12 to 40.15)	2698	43358	62.23 (59.88 to 64.57)
	IMD=3	1435	36432.28	39.39 (37.35 to 41.43)	2216	42937	51.61 (49.46 to 53.76)	1393	35762.94	38.95 (36.91 to 41.00)	2738	42937	63.77 (61.38 to 66.16)
	IMD=4	1334	31430.32	42.44 (40.17 to 44.72)	2030	37133	54.67 (52.29 to 57.05)	1151	30886.48	37.27 (35.11 to 39.42)	2309	37133	62.18 (59.65 to 64.72)
	IMD=5	1288	31485.24	40.91 (38.67 to 43.14)	1996	37143	53.74 (51.38 to 56.10)	1171	30738.31	38.10 (35.91 to 40.28)	2380	37143	64.08 (61.50 to 66.65)

	IMD=6	1243	32003.2	38.84 (36.68 to 41.00)	1952	37760	51.69 (49.40 to 53.99)	1256	31253.35	40.19 (37.97 to 42.41)	2452	37760	64.94 (62.37 to 67.51)
	IMD=7	1203	31368.56	38.35 (36.18 to 40.52)	1892	37068	51.04 (48.74 to 53.34)	1160	30640.37	37.86 (35.68 to 40.04)	2335	37068	62.99 (60.44 to 65.55)
	IMD=8	934	25661.68	36.40 (34.06 to 38.73)	1446	30239	47.82 (45.35 to 50.28)	997	24983.69	39.91 (37.43 to 42.38)	2015	30239	66.64 (63.73 to 69.55)
	IMD=9	1000	27192.07	36.78 (34.50 to 39.05)	1556	31958	48.69 (46.27 to 51.11)	973	26647.4	36.51 (34.22 to 38.81)	1910	31958	59.77 (57.09 to 62.45)
	Most Deprivation	832	24026.16	34.63 (32.28 to 36.98)	1328	28085	47.29 (44.74 to 49.83)	843	23495.39	35.88 (33.46 to 38.30)	1679	28085	59.78 (56.92 to 62.64)
2005	Least Deprivation	1554	35906.48	43.28 (41.13 to 45.43)	2621	43297	60.54 (58.22 to 62.85)	1224	35974.03	34.02 (32.12 to 35.93)	2602	43297	60.10 (57.79 to 62.41)
	IMD=2	1505	36086.48	41.71 (39.60 to 43.81)	2502	43295	57.79 (55.53 to 60.05)	1307	35687.54	36.62 (34.64 to 38.61)	2831	43295	65.39 (62.98 to 67.80)
	IMD=3	1454	35994.71	40.39 (38.32 to 42.47)	2309	42967	53.74 (51.55 to 55.93)	1326	35309.67	37.55 (35.53 to 39.57)	2852	42967	66.38 (63.94 to 68.81)
	IMD=4	1322	30825.07	42.89 (40.58 to 45.20)	2087	37118	56.23 (53.81 to 58.64)	1083	30500.74	35.51 (33.39 to 37.62)	2333	37118	62.85 (60.30 to 65.40)
	IMD=5	1272	30830.92	41.26 (38.99 to 43.52)	2063	36971	55.80 (53.39 to 58.21)	1166	30270.92	38.52 (36.31 to 40.73)	2498	36971	67.57 (64.92 to 70.22)
	IMD=6	1301	31063.11	41.88 (39.61 to 44.16)	2106	37224	56.58 (54.16 to 58.99)	1158	30445.34	38.04 (35.84 to 40.23)	2471	37224	66.38 (63.76 to 69.00)
	IMD=7	1168	30695.07	38.05 (35.87 to 40.23)	1954	36775	53.13 (50.78 to 55.49)	1165	30069.84	38.74 (36.52 to 40.97)	2548	36775	69.29 (66.60 to 71.98)
	IMD=8	1011	24850.14	40.68 (38.18 to 43.19)	1681	29804	56.40 (53.71 to 59.10)	926	24240.7	38.20 (35.74 to 40.66)	2052	29804	68.85 (65.87 to 71.83)
	IMD=9	1071	26206.25	40.87 (38.42 to 43.32)	1660	31371	52.92 (50.37 to 55.46)	943	25735.8	36.64 (34.30 to 38.98)	2008	31371	64.01 (61.21 to 66.81)
	Most Deprivation	902	23242.09	38.81 (36.28 to 41.34)	1462	27793	52.60 (49.91 to 55.30)	810	22779.33	35.56 (33.11 to 38.01)	1783	27793	64.15 (61.18 to 67.13)
2006	Least Deprivation	1585	36513.38	43.41 (41.27 to 45.55)	2742	44516	61.60 (59.29 to 63.90)	1276	36837.7	34.64 (32.74 to 36.54)	2688	44516	60.38 (58.10 to 62.67)
	IMD=2	1581	36508.02	43.31 (41.17 to 45.44)	2651	44312	59.83 (57.55 to 62.10)	1283	36353.29	35.29 (33.36 to 37.22)	2879	44312	64.97 (62.60 to 67.34)
	IMD=3	1545	35973.88	42.95 (40.81 to 45.09)	2560	43452	58.92 (56.63 to 61.20)	1320	35410.48	37.28 (35.27 to 39.29)	2864	43452	65.91 (63.50 to 68.33)
	IMD=4	1324	30820.48	42.96 (40.64 to 45.27)	2228	37463	59.47 (57.00 to 61.94)	1047	30760.14	34.04 (31.98 to 36.10)	2321	37463	61.95 (59.43 to 64.47)
	IMD=5	1239	30640.35	40.44 (38.19 to 42.69)	2176	37173	58.54 (56.08 to 61.00)	1077	30280.28	35.57 (33.44 to 37.69)	2456	37173	66.07 (63.46 to 68.68)
	IMD=6	1299	30815.57	42.15 (39.86 to 44.45)	2168	37486	57.83 (55.40 to 60.27)	1085	30344.19	35.76 (33.63 to 37.88)	2470	37486	65.89 (63.29 to 68.49)
	IMD=7	1203	30184.6	39.85 (37.60 to 42.11)	2077	36753	56.51 (54.08 to 58.94)	1087	29650.6	36.66 (34.48 to 38.84)	2544	36753	69.22 (66.53 to 71.91)
	IMD=8	975	24424.61	39.92 (37.41 to 42.42)	1694	29625	57.18 (54.46 to 59.90)	815	23842.66	34.18 (31.84 to 36.53)	2019	29625	68.15 (65.18 to 71.12)
	IMD=9	1041	25439.48	40.92 (38.43 to 43.41)	1753	30957	56.63 (53.98 to 59.28)	897	25128.2	35.70 (33.36 to 38.03)	1975	30957	63.80 (60.98 to 66.61)
	Most Deprivation	928	22672.54	40.93 (38.30 to 43.56)	1563	27518	56.80 (53.98 to 59.62)	749	22413.07	33.42 (31.02 to 35.81)	1758	27518	63.89 (60.90 to 66.87)
2007	Least Deprivation	1652	37063.27	44.57 (42.42 to 46.72)	2882	45553	63.27 (60.96 to 65.58)	1290	37658.12	34.26 (32.39 to 36.12)	2784	45553	61.12 (58.85 to 63.39)
	IMD=2	1615	37017.12	43.63 (41.50 to 45.76)	2789	45298	61.57 (59.28 to 63.86)	1310	37001.62	35.40 (33.49 to 37.32)	2906	45298	64.15 (61.82 to 66.49)
	IMD=3	1588	36165.53	43.91 (41.75 to 46.07)	2700	44207	61.08 (58.77 to 63.38)	1287	35894.82	35.85 (33.90 to 37.81)	2884	44207	65.24 (62.86 to 67.62)
	IMD=4	1290	30935.51	41.70 (39.42 to 43.98)	2275	38016	59.84 (57.38 to 62.30)	1027	31061.28	33.06 (31.04 to 35.09)	2337	38016	61.47 (58.98 to 63.97)
	IMD=5	1301	30634.23	42.47 (40.16 to 44.78)	2269	37528	60.46 (57.97 to 62.95)	1083	30394.15	35.63 (33.51 to 37.75)	2527	37528	67.34 (64.71 to 69.96)

	IMD=6	1342	30709.03	43.70 (41.36 to 46.04)	2286	37556	60.87 (58.37 to 63.36)	1092	30445.22	35.87 (33.74 to 38.00)	2542	37556	67.69 (65.05 to 70.32)
	IMD=7	1243	29868.17	41.62 (39.30 to 43.93)	2189	36627	59.76 (57.26 to 62.27)	1041	29525.1	35.26 (33.12 to 37.40)	2527	36627	68.99 (66.30 to 71.68)
	IMD=8	1025	24153.89	42.44 (39.84 to 45.03)	1783	29624	60.19 (57.39 to 62.98)	903	23753.07	38.02 (35.54 to 40.50)	2075	29624	70.04 (67.03 to 73.06)
	IMD=9	1060	24896.01	42.58 (40.01 to 45.14)	1849	30688	60.25 (57.51 to 63.00)	901	24715.89	36.45 (34.07 to 38.83)	2112	30688	68.82 (65.89 to 71.76)
	Most Deprivation	907	22205.85	40.85 (38.19 to 43.50)	1626	27360	59.43 (56.54 to 62.32)	777	22099.97	35.16 (32.69 to 37.63)	1831	27360	66.92 (63.86 to 69.99)
2008	Least Deprivation	1643	38080.82	43.15 (41.06 to 45.23)	2987	46652	64.03 (61.73 to 66.32)	1391	38732.7	35.91 (34.03 to 37.80)	2923	46652	62.66 (60.38 to 64.93)
	IMD=2	1644	37373.02	43.99 (41.86 to 46.12)	2861	45857	62.39 (60.10 to 64.68)	1337	37553.67	35.60 (33.69 to 37.51)	2923	45857	63.74 (61.43 to 66.05)
	IMD=3	1617	36360.23	44.47 (42.30 to 46.64)	2801	44581	62.83 (60.50 to 65.16)	1353	36420.52	37.15 (35.17 to 39.13)	3028	44581	67.92 (65.50 to 70.34)
	IMD=4	1370	31198.28	43.91 (41.59 to 46.24)	2377	38280	62.10 (59.60 to 64.59)	1104	31466.48	35.08 (33.02 to 37.15)	2401	38280	62.72 (60.21 to 65.23)
	IMD=5	1360	30765.08	44.21 (41.86 to 46.56)	2351	37674	62.40 (59.88 to 64.93)	1078	30635.28	35.19 (33.09 to 37.29)	2516	37674	66.78 (64.17 to 69.39)
	IMD=6	1301	30632.78	42.47 (40.16 to 44.78)	2359	37674	62.62 (60.09 to 65.14)	1145	30551.44	37.48 (35.31 to 39.65)	2601	37674	69.04 (66.39 to 71.69)
	IMD=7	1252	29838.64	41.96 (39.63 to 44.28)	2277	36621	62.18 (59.62 to 64.73)	1051	29552.14	35.56 (33.41 to 37.71)	2560	36621	69.91 (67.20 to 72.61)
	IMD=8	1032	23791.2	43.38 (40.73 to 46.02)	1857	29431	63.10 (60.23 to 65.97)	846	23615.66	35.82 (33.41 to 38.24)	2088	29431	70.95 (67.90 to 73.99)
	IMD=9	1123	24311.04	46.19 (43.49 to 48.89)	1964	30246	64.93 (62.06 to 67.81)	863	24302.87	35.51 (33.14 to 37.88)	2017	30246	66.69 (63.78 to 69.60)
	Most Deprivation	972	21862.02	44.46 (41.67 to 47.26)	1685	27144	62.08 (59.11 to 65.04)	743	21841.72	34.02 (31.57 to 36.46)	1855	27144	68.34 (65.23 to 71.45)
2009	Least Deprivation	1667	39016.06	42.73 (40.67 to 44.78)	3032	48001	63.17 (60.92 to 65.41)	1345	39678.52	33.90 (32.09 to 35.71)	2876	48001	59.92 (57.73 to 62.11)
	IMD=2	1636	37963.52	43.09 (41.01 to 45.18)	2993	46786	63.97 (61.68 to 66.26)	1371	38313.93	35.78 (33.89 to 37.68)	3056	46786	65.32 (63.00 to 67.63)
	IMD=3	1677	36938.86	45.40 (43.23 to 47.57)	2931	45738	64.08 (61.76 to 66.40)	1398	37220.63	37.56 (35.59 to 39.53)	3089	45738	67.54 (65.16 to 69.92)
	IMD=4	1443	31468.25	45.86 (43.49 to 48.22)	2479	38854	63.80 (61.29 to 66.31)	1163	31861.91	36.50 (34.40 to 38.60)	2438	38854	62.75 (60.26 to 65.24)
	IMD=5	1350	30877.06	43.72 (41.39 to 46.05)	2432	38183	63.69 (61.16 to 66.22)	1134	31005.07	36.57 (34.45 to 38.70)	2594	38183	67.94 (65.32 to 70.55)
	IMD=6	1459	30632.1	47.63 (45.19 to 50.07)	2548	38036	66.99 (64.39 to 69.59)	1153	30729.87	37.52 (35.35 to 39.69)	2638	38036	69.36 (66.71 to 72.00)
	IMD=7	1307	29669.86	44.05 (41.66 to 46.44)	2373	36793	64.50 (61.90 to 67.09)	1069	29594.84	36.12 (33.96 to 38.29)	2604	36793	70.77 (68.06 to 73.49)
	IMD=8	1088	23593.14	46.12 (43.37 to 48.86)	1948	29489	66.06 (63.13 to 68.99)	900	23580.47	38.17 (35.67 to 40.66)	2156	29489	73.11 (70.03 to 76.20)
	IMD=9	1122	23819.81	47.10 (44.35 to 49.86)	2065	30033	68.76 (65.79 to 71.72)	889	23960.45	37.10 (34.66 to 39.54)	2099	30033	69.89 (66.90 to 72.88)
	Most Deprivation	912	21437.58	42.54 (39.78 to 45.30)	1679	26854	62.52 (59.53 to 65.51)	786	21502.88	36.55 (34.00 to 39.11)	1904	26854	70.90 (67.72 to 74.09)
2010	Least Deprivation	1857	40111.99	46.30 (44.19 to 48.40)	3304	49504	66.74 (64.47 to 69.02)	1399	40960.15	34.16 (32.37 to 35.94)	2942	49504	59.43 (57.28 to 61.58)
	IMD=2	1703	38869.04	43.81 (41.73 to 45.89)	3078	47985	64.15 (61.88 to 66.41)	1447	39282.37	36.84 (34.94 to 38.73)	3183	47985	66.33 (64.03 to 68.64)
	IMD=3	1665	37621.02	44.26 (42.13 to 46.38)	2997	46671	64.22 (61.92 to 66.51)	1395	37874.9	36.83 (34.90 to 38.76)	3152	46671	67.54 (65.18 to 69.89)
	IMD=4	1501	31950.21	46.98 (44.60 to 49.36)	2593	39663	65.38 (62.86 to 67.89)	1163	32435.59	35.86 (33.79 to 37.92)	2450	39663	61.77 (59.32 to 64.22)
	IMD=5	1439	31135.06	46.22 (43.83 to 48.61)	2578	38763	66.51 (63.94 to 69.07)	1101	31448.09	35.01 (32.94 to 37.08)	2545	38763	65.66 (63.10 to 68.21)

	IMD=6	1389	30592.94	45.40 (43.01 to 47.79)	2603	38395	67.80 (65.19 to 70.40)	1105	30929.55	35.73 (33.62 to 37.83)	2655	38395	69.15 (66.52 to 71.78)
	IMD=7	1276	29727.02	42.92 (40.57 to 45.28)	2372	37009	64.09 (61.51 to 66.67)	1040	29848.41	34.84 (32.73 to 36.96)	2613	37009	70.60 (67.90 to 73.31)
	IMD=8	1054	23441.71	44.96 (42.25 to 47.68)	1971	29442	66.95 (63.99 to 69.90)	818	23486.76	34.83 (32.44 to 37.21)	2136	29442	72.55 (69.47 to 75.63)
	IMD=9	1107	23511.65	47.08 (44.31 to 49.86)	2078	29766	69.81 (66.81 to 72.81)	889	23749.54	37.43 (34.97 to 39.89)	2135	29766	71.73 (68.68 to 74.77)
	Most Deprivation	939	21022.09	44.67 (41.81 to 47.52)	1769	26567	66.59 (63.48 to 69.69)	727	21191.49	34.31 (31.81 to 36.80)	1883	26567	70.88 (67.68 to 74.08)
2011	Least Deprivation	1809	41370.88	43.73 (41.71 to 45.74)	3300	51071	64.62 (62.41 to 66.82)	1485	42381.42	35.04 (33.26 to 36.82)	3043	51071	59.58 (57.47 to 61.70)
	IMD=2	1863	39948.32	46.64 (44.52 to 48.75)	3326	49455	67.25 (64.97 to 69.54)	1344	40518.46	33.17 (31.40 to 34.94)	3164	49455	63.98 (61.75 to 66.21)
	IMD=3	1843	38321.5	48.09 (45.90 to 50.29)	3293	47707	69.03 (66.67 to 71.38)	1405	38707.3	36.30 (34.40 to 38.20)	3194	47707	66.95 (64.63 to 69.27)
	IMD=4	1549	32617.99	47.49 (45.12 to 49.85)	2738	40628	67.39 (64.87 to 69.92)	1208	33234.39	36.35 (34.30 to 38.40)	2587	40628	63.68 (61.22 to 66.13)
	IMD=5	1414	31646.73	44.68 (42.35 to 47.01)	2597	39490	65.76 (63.23 to 68.29)	1116	32108.54	34.76 (32.72 to 36.80)	2588	39490	65.54 (63.01 to 68.06)
	IMD=6	1494	30677.79	48.70 (46.23 to 51.17)	2753	38741	71.06 (68.41 to 73.72)	1169	31182.49	37.49 (35.34 to 39.64)	2699	38741	69.67 (67.04 to 72.30)
	IMD=7	1374	29848.54	46.03 (43.60 to 48.47)	2526	37306	67.71 (65.07 to 70.35)	1073	30120.64	35.62 (33.49 to 37.75)	2573	37306	68.97 (66.31 to 71.64)
	IMD=8	1138	23331.92	48.77 (45.94 to 51.61)	2065	29497	70.01 (66.99 to 73.03)	877	23577.56	37.20 (34.73 to 39.66)	2137	29497	72.45 (69.38 to 75.52)
	IMD=9	1163	23198.19	50.13 (47.25 to 53.01)	2201	29625	74.30 (71.19 to 77.40)	928	23547.7	39.41 (36.87 to 41.94)	2217	29625	74.84 (71.72 to 77.95)
	Most Deprivation	1037	20736.82	50.01 (46.96 to 53.05)	1937	26400	73.37 (70.10 to 76.64)	764	21014.9	36.36 (33.78 to 38.93)	1933	26400	73.22 (69.96 to 76.48)
2012	Least Deprivation	1869	42440.02	44.04 (42.04 to 46.04)	3372	52375	64.38 (62.21 to 66.55)	1452	43585.96	33.31 (31.60 to 35.03)	2975	52375	56.80 (54.76 to 58.84)
	IMD=2	1818	41025.48	44.31 (42.28 to 46.35)	3326	50787	65.49 (63.26 to 67.71)	1375	41811.68	32.89 (31.15 to 34.62)	3112	50787	61.28 (59.12 to 63.43)
	IMD=3	1801	38914.54	46.28 (44.14 to 48.42)	3346	48500	68.99 (66.65 to 71.33)	1435	39534.48	36.30 (34.42 to 38.18)	3190	48500	65.77 (63.49 to 68.06)
	IMD=4	1479	33148.64	44.62 (42.34 to 46.89)	2719	41394	65.69 (63.22 to 68.15)	1193	33867.68	35.23 (33.23 to 37.22)	2598	41394	62.76 (60.35 to 65.18)
	IMD=5	1478	32171.49	45.94 (43.60 to 48.28)	2718	40105	67.77 (65.22 to 70.32)	1111	32784.3	33.89 (31.90 to 35.88)	2606	40105	64.98 (62.48 to 67.47)
	IMD=6	1420	30999.13	45.81 (43.43 to 48.19)	2665	39189	68.00 (65.42 to 70.59)	1152	31611.7	36.44 (34.34 to 38.55)	2692	39189	68.69 (66.10 to 71.29)
	IMD=7	1381	29959.11	46.10 (43.66 to 48.53)	2623	37527	69.90 (67.22 to 72.57)	1098	30404.75	36.11 (33.98 to 38.25)	2689	37527	71.66 (68.95 to 74.36)
	IMD=8	1135	23462.47	48.38 (45.56 to 51.19)	2131	29785	71.55 (68.51 to 74.58)	945	23768.17	39.76 (37.22 to 42.29)	2226	29785	74.74 (71.63 to 77.84)
	IMD=9	1130	23026.5	49.07 (46.21 to 51.94)	2200	29513	74.54 (71.43 to 77.66)	898	23441.12	38.31 (35.80 to 40.81)	2202	29513	74.61 (71.49 to 77.73)
	Most Deprivation	937	20364.45	46.01 (43.07 to 48.96)	1900	26138	72.69 (69.42 to 75.96)	704	20811.57	33.83 (31.33 to 36.33)	1861	26138	71.20 (67.96 to 74.43)
2013	Least Deprivation	1976	43299.94	45.64 (43.62 to 47.65)	3444	53699	64.14 (61.99 to 66.28)	1534	44495.49	34.48 (32.75 to 36.20)	3106	53699	57.84 (55.81 to 59.88)
	IMD=2	1923	41588.41	46.24 (44.17 to 48.31)	3446	51880	66.42 (64.20 to 68.64)	1568	42502.13	36.89 (35.07 to 38.72)	3268	51880	62.99 (60.83 to 65.15)
	IMD=3	1837	39435.53	46.58 (44.45 to 48.71)	3333	49571	67.24 (64.95 to 69.52)	1575	40205.03	39.17 (37.24 to 41.11)	3345	49571	67.48 (65.19 to 69.77)
	IMD=4	1572	33514.66	46.90 (44.59 to 49.22)	2837	42175	67.27 (64.79 to 69.74)	1271	34314.85	37.04 (35.00 to 39.08)	2718	42175	64.45 (62.02 to 66.87)
	IMD=5	1438	32520.72	44.22 (41.93 to 46.50)	2677	40785	65.64 (63.15 to 68.12)	1260	33237.81	37.91 (35.82 to 40.00)	2670	40785	65.47 (62.98 to 67.95)

	IMD=6	1432	31187.67	45.92 (43.54 to 48.29)	2721	39638	68.65 (66.07 to 71.23)	1178	31779.03	37.07 (34.95 to 39.19)	2716	39638	68.52 (65.94 to 71.10)
	IMD=7	1366	29848.59	45.76 (43.34 to 48.19)	2585	38001	68.02 (65.40 to 70.65)	1208	30253.49	39.93 (37.68 to 42.18)	2770	38001	72.89 (70.18 to 75.61)
	IMD=8	1100	23352.97	47.10 (44.32 to 49.89)	2143	29930	71.60 (68.57 to 74.63)	943	23664.41	39.85 (37.31 to 42.39)	2198	29930	73.44 (70.37 to 76.51)
	IMD=9	1182	22628.14	52.24 (49.26 to 55.21)	2219	29447	75.36 (72.22 to 78.49)	891	23090.23	38.59 (36.05 to 41.12)	2170	29447	73.69 (70.59 to 76.79)
	Most Deprivation	940	19870.79	47.31 (44.28 to 50.33)	1938	25951	74.68 (71.35 to 78.00)	786	20328.19	38.67 (35.96 to 41.37)	1926	25951	74.22 (70.90 to 77.53)
2014	Least Deprivation	2032	44111.16	46.07 (44.06 to 48.07)	3589	54786	65.51 (63.37 to 67.65)	1705	45425.75	37.53 (35.75 to 39.32)	3246	54786	59.25 (57.21 to 61.29)
	IMD=2	2034	42176.9	48.23 (46.13 to 50.32)	3546	52773	67.19 (64.98 to 69.41)	1621	43262.08	37.47 (35.65 to 39.29)	3284	52773	62.23 (60.10 to 64.36)
	IMD=3	1890	40028.5	47.22 (45.09 to 49.35)	3404	50334	67.63 (65.36 to 69.90)	1631	40896.74	39.88 (37.95 to 41.82)	3411	50334	67.77 (65.49 to 70.04)
	IMD=4	1506	33794.64	44.56 (42.31 to 46.81)	2759	42462	64.98 (62.55 to 67.40)	1280	34645.21	36.95 (34.92 to 38.97)	2656	42462	62.55 (60.17 to 64.93)
	IMD=5	1596	32948.16	48.44 (46.06 to 50.82)	2882	41255	69.86 (67.31 to 72.41)	1291	33662.07	38.35 (36.26 to 40.44)	2654	41255	64.33 (61.88 to 66.78)
	IMD=6	1535	31452.69	48.80 (46.36 to 51.24)	2760	39956	69.08 (66.50 to 71.65)	1335	32089.71	41.60 (39.37 to 43.83)	2844	39956	71.18 (68.56 to 73.79)
	IMD=7	1476	29893.87	49.37 (46.86 to 51.89)	2647	37865	69.91 (67.24 to 72.57)	1209	30348.16	39.84 (37.59 to 42.08)	2690	37865	71.04 (68.36 to 73.73)
	IMD=8	1158	23326.79	49.64 (46.78 to 52.50)	2091	29825	70.11 (67.10 to 73.11)	952	23575.12	40.38 (37.82 to 42.95)	2202	29825	73.83 (70.75 to 76.91)
	IMD=9	1139	22568.76	50.47 (47.54 to 53.40)	2159	29336	73.60 (70.49 to 76.70)	976	23031.73	42.38 (39.72 to 45.03)	2191	29336	74.69 (71.56 to 77.81)
	Most Deprivation	1052	19400.2	54.23 (50.95 to 57.50)	1955	25292	77.30 (73.87 to 80.72)	855	19963.83	42.83 (39.96 to 45.70)	1831	25292	72.39 (69.08 to 75.71)
2015	Least Deprivation	2110	44693.66	47.21 (45.20 to 49.22)	3574	55534	64.36 (62.25 to 66.47)	1746	46014.56	37.94 (36.16 to 39.72)	3311	55534	59.62 (57.59 to 61.65)
	IMD=2	1992	42601.85	46.76 (44.71 to 48.81)	3477	53232	65.32 (63.15 to 67.49)	1689	43617.09	38.72 (36.88 to 40.57)	3432	53232	64.47 (62.32 to 66.63)
	IMD=3	1825	40564.72	44.99 (42.93 to 47.05)	3272	50873	64.32 (62.11 to 66.52)	1652	41336.32	39.96 (38.04 to 41.89)	3469	50873	68.19 (65.92 to 70.46)
	IMD=4	1632	34132.74	47.81 (45.49 to 50.13)	2808	42781	65.64 (63.21 to 68.06)	1449	34826.2	41.61 (39.46 to 43.75)	2806	42781	65.59 (63.16 to 68.02)
	IMD=5	1470	33113.29	44.39 (42.12 to 46.66)	2706	41512	65.19 (62.73 to 67.64)	1298	33715.71	38.50 (36.40 to 40.59)	2688	41512	64.75 (62.30 to 67.20)
	IMD=6	1534	31508.05	48.69 (46.25 to 51.12)	2678	39934	67.06 (64.52 to 69.60)	1307	31985.66	40.86 (38.65 to 43.08)	2786	39934	69.77 (67.17 to 72.36)
	IMD=7	1428	29765.4	47.98 (45.49 to 50.46)	2608	37794	69.01 (66.36 to 71.65)	1202	30126.66	39.90 (37.64 to 42.15)	2642	37794	69.91 (67.24 to 72.57)
	IMD=8	1105	23181.87	47.67 (44.86 to 50.48)	2009	29670	67.71 (64.75 to 70.67)	946	23404.53	40.42 (37.84 to 43.00)	2143	29670	72.23 (69.17 to 75.29)
	IMD=9	1157	22424.72	51.59 (48.62 to 54.57)	2152	29110	73.93 (70.80 to 77.05)	950	22840.21	41.59 (38.95 to 44.24)	2193	29110	75.33 (72.18 to 78.49)
	Most Deprivation	999	19074.96	52.37 (49.12 to 55.62)	1868	25054	74.56 (71.18 to 77.94)	813	19561.55	41.56 (38.70 to 44.42)	1809	25054	72.20 (68.88 to 75.53)
2016	Least Deprivation	2097	45377.77	46.21 (44.23 to 48.19)	3536	56199	62.92 (60.85 to 64.99)	1794	46478.03	38.60 (36.81 to 40.39)	3373	56199	60.02 (57.99 to 62.04)
	IMD=2	1940	43209.77	44.90 (42.90 to 46.90)	3382	53791	62.87 (60.75 to 64.99)	1763	44041.9	40.03 (38.16 to 41.90)	3491	53791	64.90 (62.75 to 67.05)
	IMD=3	1914	41288.89	46.36 (44.28 to 48.43)	3251	51547	63.07 (60.90 to 65.24)	1700	41872.58	40.60 (38.67 to 42.53)	3444	51547	66.81 (64.58 to 69.04)
	IMD=4	1615	34532	46.77 (44.49 to 49.05)	2810	43281	64.92 (62.52 to 67.33)	1443	35183	41.01 (38.90 to 43.13)	2882	43281	66.59 (64.16 to 69.02)
	IMD=5	1519	33799.19	44.94 (42.68 to 47.20)	2668	42103	63.37 (60.96 to 65.77)	1372	34263.48	40.04 (37.92 to 42.16)	2696	42103	64.03 (61.62 to 66.45)

	IMD=6	1486	31696.47	46.88 (44.50 to 49.27)	2642	40010	66.03 (63.52 to 68.55)	1302	32032.07	40.65 (38.44 to 42.85)	2740	40010	68.48 (65.92 to 71.05)
	IMD=7	1386	29891.05	46.37 (43.93 to 48.81)	2488	37817	65.79 (63.21 to 68.38)	1186	30255.29	39.20 (36.97 to 41.43)	2607	37817	68.94 (66.29 to 71.58)
	IMD=8	1083	23198.91	46.68 (43.90 to 49.46)	1926	29529	65.22 (62.31 to 68.14)	1017	23338.45	43.58 (40.90 to 46.25)	2183	29529	73.93 (70.83 to 77.03)
	IMD=9	1057	22415.86	47.15 (44.31 to 50.00)	2038	28996	70.29 (67.23 to 73.34)	1023	22764.54	44.94 (42.18 to 47.69)	2222	28996	76.63 (73.44 to 79.82)
	Most Deprivation	904	18907.56	47.81 (44.69 to 50.93)	1714	24693	69.41 (66.13 to 72.70)	826	19341.63	42.71 (39.79 to 45.62)	1826	24693	73.95 (70.56 to 77.34)
2017	Least Deprivation	2031	46869.66	43.33 (41.45 to 45.22)	3403	57890	58.78 (56.81 to 60.76)	1828	47761.11	38.27 (36.52 to 40.03)	3403	57890	58.78 (56.81 to 60.76)
	IMD=2	1966	44778.73	43.90 (41.96 to 45.85)	3319	55636	59.66 (57.63 to 61.69)	1869	45367.98	41.20 (39.33 to 43.06)	3571	55636	64.19 (62.08 to 66.29)
	IMD=3	1878	42631.05	44.05 (42.06 to 46.04)	3210	53111	60.44 (58.35 to 62.53)	1791	42896.32	41.75 (39.82 to 43.69)	3571	53111	67.24 (65.03 to 69.44)
	IMD=4	1585	35653.54	44.46 (42.27 to 46.64)	2697	44459	60.66 (58.37 to 62.95)	1457	36044.33	40.42 (38.35 to 42.50)	2856	44459	64.24 (61.88 to 66.59)
	IMD=5	1502	34506.7	43.53 (41.33 to 45.73)	2634	43181	61.00 (58.67 to 63.33)	1391	34993.45	39.75 (37.66 to 41.84)	2732	43181	63.27 (60.90 to 65.64)
	IMD=6	1457	32367.73	45.01 (42.70 to 47.33)	2542	40766	62.36 (59.93 to 64.78)	1420	32595.49	43.56 (41.30 to 45.83)	2813	40766	69.00 (66.45 to 71.55)
	IMD=7	1387	30467.74	45.52 (43.13 to 47.92)	2467	38533	64.02 (61.50 to 66.55)	1313	30743.78	42.71 (40.40 to 45.02)	2729	38533	70.82 (68.17 to 73.48)
	IMD=8	1106	23515.9	47.03 (44.26 to 49.80)	1947	29835	65.26 (62.36 to 68.16)	976	23505.6	41.52 (38.92 to 44.13)	2134	29835	71.53 (68.49 to 74.56)
	IMD=9	1156	22450.28	51.49 (48.52 to 54.46)	2072	29128	71.13 (68.07 to 74.20)	919	22863.2	40.20 (37.60 to 42.79)	2081	29128	71.44 (68.37 to 74.51)
	Most Deprivation	940	18808.2	49.98 (46.78 to 53.17)	1760	24547	71.70 (68.35 to 75.05)	866	19142.82	45.24 (42.23 to 48.25)	1884	24547	76.75 (73.28 to 80.22)
2018	Least Deprivation	2134	49420.65	43.18 (41.35 to 45.01)	3530	60752	58.11 (56.19 to 60.02)	2005	50040.31	40.07 (38.31 to 41.82)	3647	60752	60.03 (58.08 to 61.98)
	IMD=2	2042	47222.67	43.24 (41.37 to 45.12)	3390	58334	58.11 (56.16 to 60.07)	2010	47248.24	42.54 (40.68 to 44.40)	3799	58334	65.12 (63.05 to 67.20)
	IMD=3	1910	45392.45	42.08 (40.19 to 43.96)	3253	56170	57.91 (55.92 to 59.90)	1880	45261.23	41.54 (39.66 to 43.41)	3706	56170	65.98 (63.85 to 68.10)
	IMD=4	1659	37607.69	44.11 (41.99 to 46.24)	2771	46866	59.13 (56.92 to 61.33)	1537	37854.73	40.60 (38.57 to 42.63)	2962	46866	63.20 (60.93 to 65.48)
	IMD=5	1505	36366.09	41.38 (39.29 to 43.48)	2574	44972	57.24 (55.02 to 59.45)	1496	36553.49	40.93 (38.85 to 43.00)	2870	44972	63.82 (61.48 to 66.15)
	IMD=6	1498	33584.66	44.60 (42.34 to 46.86)	2533	42403	59.74 (57.41 to 62.06)	1345	33695.03	39.92 (37.78 to 42.05)	2787	42403	65.73 (63.29 to 68.17)
	IMD=7	1362	31626.92	43.06 (40.78 to 45.35)	2391	39933	59.88 (57.48 to 62.28)	1337	31631.29	42.27 (40.00 to 44.53)	2740	39933	68.61 (66.05 to 71.18)
	IMD=8	1088	24098.88	45.15 (42.46 to 47.83)	1903	30627	62.13 (59.34 to 64.93)	1102	23939.3	46.03 (43.32 to 48.75)	2185	30627	71.34 (68.35 to 74.33)
	IMD=9	1129	22764.95	49.59 (46.70 to 52.49)	2035	29674	68.58 (65.60 to 71.56)	973	23074.11	42.17 (39.52 to 44.82)	2061	29674	69.45 (66.46 to 72.45)
	Most Deprivation	946	18961.94	49.89 (46.71 to 53.07)	1676	24674	67.93 (64.67 to 71.18)	874	19049.29	45.88 (42.84 to 48.92)	1914	24674	77.57 (74.10 to 81.05)
2019	Least Deprivation	2161	51498.5	41.96 (40.19 to 43.73)	3505	63903	54.85 (53.03 to 56.66)	2191	51750.86	42.34 (40.56 to 44.11)	3994	63903	62.50 (60.56 to 64.44)
	IMD=2	2049	49051.73	41.77 (39.96 to 43.58)	3400	61291	55.47 (53.61 to 57.34)	2065	48674.33	42.42 (40.59 to 44.25)	4000	61291	65.26 (63.24 to 67.28)
	IMD=3	1840	46196.58	39.83 (38.01 to 41.65)	3109	58675	52.99 (51.12 to 54.85)	1900	45809.22	41.48 (39.61 to 43.34)	3778	58675	64.39 (62.34 to 66.44)
	IMD=4	1666	38160.25	43.66 (41.56 to 45.75)	2770	48754	56.82 (54.70 to 58.93)	1655	38166.25	43.36 (41.27 to 45.45)	3092	48754	63.42 (61.18 to 65.66)
	IMD=5	1546	37454.91	41.28 (39.22 to 43.33)	2616	46943	55.73 (53.59 to 57.86)	1582	37375.59	42.33 (40.24 to 44.41)	3044	46943	64.84 (62.54 to 67.15)

	IMD=6	1458	34220.13	42.61 (40.42 to 44.79)	2481	43416	57.14 (54.90 to 59.39)	1471	34151.83	43.07 (40.87 to 45.27)	2879	43416	66.31 (63.89 to 68.73)
	IMD=7	1324	32124.51	41.21 (38.99 to 43.43)	2336	40856	57.18 (54.86 to 59.50)	1346	32001.59	42.06 (39.81 to 44.31)	2761	40856	67.58 (65.06 to 70.10)
	IMD=8	1030	23986.9	42.94 (40.32 to 45.56)	1802	30857	58.40 (55.70 to 61.09)	1038	23793.62	43.63 (40.97 to 46.28)	2083	30857	67.50 (64.61 to 70.40)
	IMD=9	1117	22647.38	49.32 (46.43 to 52.21)	1931	29492	65.48 (62.55 to 68.40)	1011	22749.94	44.44 (41.70 to 47.18)	2131	29492	72.26 (69.19 to 75.32)
	Most Deprivation	853	18270.91	46.69 (43.55 to 49.82)	1563	23867	65.49 (62.24 to 68.73)	784	18253.2	42.95 (39.94 to 45.96)	1745	23867	73.11 (69.68 to 76.54)
**85 + years**													
2004	Least Deprivation	456	11454.94	39.81 (36.15to 43.46)	719	14377	50.01 (46.35 to 53.67)	389	11287.06	34.46 (31.04 to 37.89)	785	14377	54.60 (50.78 to 58.42)
	IMD=2	485	12589.96	38.52 (35.09to 41.95)	758	15730	48.19 (44.76 to 51.62)	365	12341.53	29.57 (26.54 to 32.61)	811	15730	51.56 (48.01 to 55.11)
	IMD=3	504	12732.03	39.59 (36.13to 43.04)	752	15803	47.59 (44.18 to 50.99)	409	12425.24	32.92 (29.73 to 36.11)	864	15803	54.67 (51.03 to 58.32)
	IMD=4	445	11173.68	39.83 (36.13to 43.53)	644	13915	46.28 (42.71 to 49.86)	344	10977.07	31.34 (28.03 to 34.65)	720	13915	51.74 (47.96 to 55.52)
	IMD=5	398	10913.91	36.47 (32.88to 40.05)	609	13471	45.21 (41.62 to 48.80)	337	10711.03	31.46 (28.10 to 34.82)	724	13471	53.75 (49.83 to 57.66)
	IMD=6	435	11451.3	37.99 (34.42to 41.56)	642	14108	45.51 (41.99 to 49.03)	399	11073.79	36.03 (32.50 to 39.57)	814	14108	57.70 (53.73 to 61.66)
	IMD=7	366	11140.91	32.85 (29.49to 36.22)	537	13636	39.38 (36.05 to 42.71)	367	10807.82	33.96 (30.48 to 37.43)	753	13636	55.22 (51.28 to 59.17)
	IMD=8	277	8973.334	30.87 (27.23to 34.50)	405	10970	36.92 (33.32 to 40.51)	262	8658.53	30.26 (26.60 to 33.92)	588	10970	53.60 (49.27 to 57.93)
	IMD=9	285	8974.971	31.75 (28.07to 35.44)	425	10970	38.74 (35.06 to 42.43)	315	8720.551	36.12 (32.13 to 40.11)	624	10970	56.88 (52.42 to 61.35)
	Most Deprivation	244	7887.231	30.94 (27.05to 34.82)	363	9547	38.02 (34.11 to 41.93)	209	7670.965	27.25 (23.55 to 30.94)	440	9547	46.09 (41.78 to 50.39)
2005	Least Deprivation	466	12157.69	38.33 (34.85to 41.81)	800	15442	51.81 (48.22 to 55.40)	397	12078.11	32.87 (29.64 to 36.10)	892	15442	57.76 (53.97 to 61.56)
	IMD=2	537	13357.68	40.20 (36.80to 43.60)	908	16881	53.79 (50.29 to 57.29)	483	13132.15	36.78 (33.50 to 40.06)	1031	16881	61.07 (57.35 to 64.80)
	IMD=3	507	13554.83	37.40 (34.15to 40.66)	818	17033	48.02 (44.73 to 51.32)	448	13260.77	33.78 (30.66 to 36.91)	1022	17033	60.00 (56.32 to 63.68)
	IMD=4	469	11777.67	39.82 (36.22to 43.43)	704	14871	47.34 (43.84 to 50.84)	399	11589.46	34.43 (31.05 to 37.81)	851	14871	57.23 (53.38 to 61.07)
	IMD=5	424	11692.08	36.26 (32.81to 39.72)	701	14618	47.95 (44.40 to 51.50)	416	11437.17	36.37 (32.88 to 39.87)	920	14618	62.94 (58.87 to 67.00)
	IMD=6	474	12004.67	39.48 (35.93to 43.04)	750	15079	49.74 (46.18 to 53.30)	386	11642.09	33.16 (29.85 to 36.46)	905	15079	60.02 (56.11 to 63.93)
	IMD=7	409	11905.42	34.35 (31.02to 37.68)	626	14697	42.59 (39.26 to 45.93)	357	11579.02	30.83 (27.63 to 34.03)	817	14697	55.59 (51.78 to 59.40)
	IMD=8	307	9500.216	32.32 (28.70to 35.93)	473	11724	40.34 (36.71 to 43.98)	319	9143.543	34.89 (31.06 to 38.72)	719	11724	61.33 (56.84 to 65.81)
	IMD=9	331	9416.15	35.15 (31.37to 38.94)	529	11724	45.12 (41.28 to 48.97)	284	9168.498	30.98 (27.37 to 34.58)	670	11724	57.15 (52.82 to 61.48)
	Most Deprivation	262	8301.632	31.56 (27.74to 35.38)	417	10232	40.75 (36.84 to 44.67)	245	8094.078	30.27 (26.48 to 34.06)	573	10232	56.00 (51.42 to 60.59)
2006	Least Deprivation	511	12936.68	39.50 (36.08to 42.92)	872	16507	52.83 (49.32 to 56.33)	394	12845.87	30.67 (27.64 to 33.70)	942	16507	57.07 (53.42 to 60.71)
	IMD=2	559	13974.71	40.00 (36.68to 43.32)	948	17747	53.42 (50.02 to 56.82)	404	13788.07	29.30 (26.44 to 32.16)	1005	17747	56.63 (53.13 to 60.13)
	IMD=3	557	14194.41	39.24 (35.98to 42.50)	917	18048	50.81 (47.52 to 54.10)	468	13953.97	33.54 (30.50 to 36.58)	1060	18048	58.73 (55.20 to 62.27)
	IMD=4	506	12272.2	41.23 (37.64to 44.82)	823	15641	52.62 (49.02 to 56.21)	380	12115.09	31.37 (28.21 to 34.52)	874	15641	55.88 (52.17 to 59.58)

	IMD=5	462	12176.11	37.94 (34.48to 41.40)	786	15392	51.07 (47.50 to 54.64)	397	11953.47	33.21 (29.95 to 36.48)	922	15392	59.90 (56.03 to 63.77)
	IMD=6	482	12515.12	38.51 (35.08to 41.95)	806	15788	51.05 (47.53 to 54.58)	373	12206.53	30.56 (27.46 to 33.66)	901	15788	57.07 (53.34 to 60.80)
	IMD=7	504	12339.44	40.84 (37.28to 44.41)	787	15538	50.65 (47.11 to 54.19)	352	12073.26	29.16 (26.11 to 32.20)	882	15538	56.76 (53.02 to 60.51)
	IMD=8	346	9850.869	35.12 (31.42to 38.82)	567	12313	46.05 (42.26 to 49.84)	288	9478.396	30.38 (26.88 to 33.89)	730	12313	59.29 (54.99 to 63.59)
	IMD=9	337	9819.816	34.32 (30.65to 37.98)	574	12322	46.58 (42.77 to 50.39)	280	9624.304	29.09 (25.69 to 32.50)	696	12322	56.48 (52.29 to 60.68)
	Most Deprivation	263	8660.592	30.37 (26.70to 34.04)	463	10686	43.33 (39.38 to 47.27)	232	8455.129	27.44 (23.91 to 30.97)	587	10686	54.93 (50.49 to 59.38)
2007	Least Deprivation	558	13601.22	41.03 (37.62to 44.43)	1005	17376	57.84 (54.26 to 61.41)	409	13610.63	30.05 (27.14 to 32.96)	965	17376	55.54 (52.03 to 59.04)
	IMD=2	606	14503.55	41.78 (38.46to 45.11)	1051	18588	56.54 (53.12 to 59.96)	437	14474.42	30.19 (27.36 to 33.02)	1061	18588	57.08 (53.65 to 60.51)
	IMD=3	632	14656.51	43.12 (39.76to 46.48)	1069	18662	57.28 (53.85 to 60.72)	469	14486.92	32.37 (29.44 to 35.30)	1171	18662	62.75 (59.15 to 66.34)
	IMD=4	554	12737.19	43.49 (39.87to 47.12)	910	16323	55.75 (52.13 to 59.37)	397	12689.98	31.28 (28.21 to 34.36)	882	16323	54.03 (50.47 to 57.60)
	IMD=5	470	12560.03	37.42 (34.04to 40.80)	847	16023	52.86 (49.30 to 56.42)	404	12356.99	32.69 (29.51 to 35.88)	961	16023	59.98 (56.18 to 63.77)
	IMD=6	514	12876.88	39.92 (36.47to 43.37)	861	16416	52.45 (48.95 to 55.95)	427	12613.92	33.85 (30.64 to 37.06)	1032	16416	62.87 (59.03 to 66.70)
	IMD=7	449	12770.83	35.16 (31.91to 38.41)	790	16088	49.10 (45.68 to 52.53)	403	12512.58	32.21 (29.06 to 35.35)	999	16088	62.10 (58.25 to 65.95)
	IMD=8	379	10030.65	37.78 (33.98to 41.59)	613	12589	48.69 (44.84 to 52.55)	330	9686.65	34.07 (30.39 to 37.74)	822	12589	65.30 (60.83 to 69.76)
	IMD=9	343	10170.17	33.73 (30.16to 37.30)	593	12840	46.18 (42.47 to 49.90)	288	9972.873	28.88 (25.54 to 32.21)	755	12840	58.80 (54.61 to 62.99)
	Most Deprivation	336	8864.87	37.90 (33.85to 41.96)	542	11060	49.01 (44.88 to 53.13)	247	8723.584	28.31 (24.78 to 31.85)	616	11060	55.70 (51.30 to 60.09)
2008	Least Deprivation	617	14294.9	43.16 (39.76to 46.57)	1071	18326	58.44 (54.94 to 61.94)	411	14361.41	28.62 (25.85 to 31.39)	999	18326	54.51 (51.13 to 57.89)
	IMD=2	595	15205.14	39.13 (35.99to 42.28)	1080	19514	55.34 (52.04 to 58.65)	448	15208.84	29.46 (26.73 to 32.18)	1136	19514	58.21 (54.83 to 61.60)
	IMD=3	582	15213.38	38.26 (35.15to 41.36)	1026	19421	52.83 (49.60 to 56.06)	488	15027.23	32.47 (29.59 to 35.36)	1207	19421	62.15 (58.64 to 65.66)
	IMD=4	553	13174.42	41.98 (38.48to 45.47)	943	16939	55.67 (52.12 to 59.22)	410	13211.15	31.03 (28.03 to 34.04)	943	16939	55.67 (52.12 to 59.22)
	IMD=5	495	12940.93	38.25 (34.88to 41.62)	895	16599	53.92 (50.39 to 57.45)	384	12809.32	29.98 (26.98 to 32.98)	978	16599	58.92 (55.23 to 62.61)
	IMD=6	527	13219.22	39.87 (36.46to 43.27)	886	16914	52.38 (48.93 to 55.83)	448	13045.46	34.34 (31.16 to 37.52)	1072	16914	63.38 (59.59 to 67.17)
	IMD=7	471	13160.22	35.79 (32.56to 39.02)	839	16598	50.55 (47.13 to 53.97)	384	12974.89	29.60 (26.64 to 32.56)	1021	16598	61.51 (57.74 to 65.29)
	IMD=8	366	10182.34	35.94 (32.26to 39.63)	642	12890	49.81 (45.95 to 53.66)	317	9856.378	32.16 (28.62 to 35.70)	844	12890	65.48 (61.06 to 69.89)
	IMD=9	349	10419.17	33.50 (29.98to 37.01)	660	13165	50.13 (46.31 to 53.96)	329	10195.47	32.27 (28.78 to 35.76)	821	13165	62.36 (58.10 to 66.63)
	Most Deprivation	299	8988.542	33.26 (29.49to 37.04)	523	11416	45.81 (41.89 to 49.74)	272	8839.986	30.77 (27.11 to 34.43)	672	11416	58.86 (54.41 to 63.32)
2009	Least Deprivation	595	14836.99	40.10 (36.88to 43.32)	1105	19197	57.56 (54.17 to 60.96)	419	15009.22	27.92 (25.24 to 30.59)	1043	19197	54.33 (51.03 to 57.63)
	IMD=2	687	15572.21	44.12 (40.82to 47.42)	1206	20103	59.99 (56.61 to 63.38)	464	15690.61	29.57 (26.88 to 32.26)	1164	20103	57.90 (54.58 to 61.23)
	IMD=3	638	15500.93	41.16 (37.97to 44.35)	1116	19977	55.86 (52.59 to 59.14)	499	15486.42	32.22 (29.39 to 35.05)	1229	19977	61.52 (58.08 to 64.96)
	IMD=4	514	13466.66	38.17 (34.87to 41.47)	922	17387	53.03 (49.61 to 56.45)	424	13610.99	31.15 (28.19 to 34.12)	998	17387	57.40 (53.84 to 60.96)

	IMD=5	536	13090.17	40.95 (37.48to 44.41)	944	16922	55.79 (52.23 to 59.34)	395	13067.59	30.23 (27.25 to 33.21)	1007	16922	59.51 (55.83 to 63.18)
	IMD=6	547	13440.26	40.70 (37.29to 44.11)	934	17275	54.07 (50.60 to 57.53)	438	13253.73	33.05 (29.95 to 36.14)	1072	17275	62.05 (58.34 to 65.77)
	IMD=7	480	13310.87	36.06 (32.83to 39.29)	850	16911	50.26 (46.88 to 53.64)	404	13142.29	30.74 (27.74 to 33.74)	1019	16911	60.26 (56.56 to 63.96)
	IMD=8	405	10110.71	40.06 (36.16to 43.96)	681	12977	52.48 (48.54 to 56.42)	321	9844.638	32.61 (29.04 to 36.17)	834	12977	64.27 (59.91 to 68.63)
	IMD=9	415	10386.24	39.96 (36.11to 43.80)	734	13266	55.33 (51.33 to 59.33)	348	10199.41	34.12 (30.53 to 37.70)	883	13266	66.56 (62.17 to 70.95)
	Most Deprivation	334	8962.842	37.26 (33.27to 41.26)	590	11395	51.78 (47.60 to 55.96)	259	8875.839	29.18 (25.63 to 32.73)	700	11395	61.43 (56.88 to 65.98)
2010	Least Deprivation	663	15473.03	42.85 (39.59to 46.11)	1211	20020	60.49 (57.08 to 63.90)	453	15700.6	28.85 (26.20 to 31.51)	1096	20020	54.75 (51.50 to 57.99)
	IMD=2	648	16179.64	40.05 (36.97to 43.13)	1233	20976	58.78 (55.50 to 62.06)	529	16368.29	32.32 (29.56 to 35.07)	1231	20976	58.69 (55.41 to 61.96)
	IMD=3	655	15906.26	41.18 (38.03to 44.33)	1217	20665	58.89 (55.58 to 62.20)	523	16004.47	32.68 (29.88 to 35.48)	1290	20665	62.42 (59.02 to 65.83)
	IMD=4	547	13686.35	39.97 (36.62to 43.32)	978	17725	55.18 (51.72 to 58.63)	438	13805.83	31.73 (28.75 to 34.70)	1042	17725	58.79 (55.22 to 62.36)
	IMD=5	539	13391.96	40.25 (36.85to 43.65)	966	17274	55.92 (52.40 to 59.45)	445	13394.36	33.22 (30.14 to 36.31)	1078	17274	62.41 (58.68 to 66.13)
	IMD=6	557	13746.57	40.52 (37.15to 43.88)	958	17707	54.10 (50.68 to 57.53)	438	13551.53	32.32 (29.29 to 35.35)	1124	17707	63.48 (59.77 to 67.19)
	IMD=7	500	13619.77	36.71 (33.49to 39.93)	908	17368	52.28 (48.88 to 55.68)	398	13440.7	29.61 (26.70 to 32.52)	1070	17368	61.61 (57.92 to 65.30)
	IMD=8	388	10275.77	37.76 (34.00to 41.52)	694	13268	52.31 (48.41 to 56.20)	321	10107.09	31.76 (28.29 to 35.23)	882	13268	66.48 (62.09 to 70.86)
	IMD=9	400	10584.72	37.79 (34.09to 41.49)	747	13581	55.00 (51.06 to 58.95)	341	10380.49	32.85 (29.36 to 36.34)	913	13581	67.23 (62.87 to 71.59)
	Most Deprivation	319	9091.469	35.09 (31.24to 38.94)	577	11636	49.59 (45.54 to 53.63)	286	9014.576	31.73 (28.05 to 35.40)	750	11636	64.46 (59.84 to 69.07)
2011	Least Deprivation	688	16054.19	42.85 (39.65to 46.06)	1287	20917	61.53 (58.17 to 64.89)	517	16381.04	31.56 (28.84 to 34.28)	1208	20917	57.75 (54.50 to 61.01)
	IMD=2	749	16483.71	45.44 (42.18to 48.69)	1386	21503	64.46 (61.06 to 67.85)	525	16738.75	31.36 (28.68 to 34.05)	1303	21503	60.60 (57.31 to 63.89)
	IMD=3	688	16391.15	41.97 (38.84to 45.11)	1247	21257	58.66 (55.41 to 61.92)	529	16432.82	32.19 (29.45 to 34.93)	1334	21257	62.76 (59.39 to 66.12)
	IMD=4	598	13975.94	42.79 (39.36to 46.22)	1026	18124	56.61 (53.15 to 60.07)	460	14104.76	32.61 (29.63 to 35.59)	1087	18124	59.98 (56.41 to 63.54)
	IMD=5	563	13697.4	41.10 (37.71to 44.50)	1044	17831	58.55 (55.00 to 62.10)	445	13785.74	32.28 (29.28 to 35.28)	1080	17831	60.57 (56.96 to 64.18)
	IMD=6	577	13981.7	41.27 (37.90to 44.64)	1020	18053	56.50 (53.03 to 59.97)	440	13876.05	31.71 (28.75 to 34.67)	1130	18053	62.59 (58.94 to 66.24)
	IMD=7	588	13771.54	42.70 (39.25to 46.15)	1056	17696	59.67 (56.08 to 63.27)	420	13667.55	30.73 (27.79 to 33.67)	1086	17696	61.37 (57.72 to 65.02)
	IMD=8	432	10408.79	41.50 (37.59to 45.42)	747	13481	55.41 (51.44 to 59.39)	323	10248.79	31.52 (28.08 to 34.95)	874	13481	64.83 (60.53 to 69.13)
	IMD=9	394	10666.52	36.94 (33.29to 40.59)	741	13738	53.94 (50.05 to 57.82)	335	10497.18	31.91 (28.50 to 35.33)	926	13738	67.40 (63.06 to 71.75)
	Most Deprivation	354	9193.539	38.51 (34.49to 42.52)	639	11808	54.12 (49.92 to 58.31)	264	9099.973	29.01 (25.51 to 32.51)	745	11808	63.09 (58.56 to 67.62)
2012	Least Deprivation	753	16623.98	45.30 (42.06to 48.53)	1352	21647	62.46 (59.13 to 65.79)	529	17056.4	31.01 (28.37 to 33.66)	1201	21647	55.48 (52.34 to 58.62)
	IMD=2	700	16920.9	41.37 (38.30to 44.43)	1275	22028	57.88 (54.70 to 61.06)	540	17205.56	31.39 (28.74 to 34.03)	1323	22028	60.06 (56.82 to 63.30)
	IMD=3	744	16733.66	44.46 (41.27to 47.66)	1339	21877	61.21 (57.93 to 64.48)	477	16847.8	28.31 (25.77 to 30.85)	1352	21877	61.80 (58.51 to 65.09)
	IMD=4	603	14289.81	42.20 (38.83to 45.57)	1079	18577	58.08 (54.62 to 61.55)	426	14403.72	29.58 (26.77 to 32.38)	1059	18577	57.01 (53.57 to 60.44)

	IMD=5	608	13888.32	43.78 (40.30to 47.26)	1077	18046	59.68 (56.12 to 63.25)	416	13980.65	29.76 (26.90 to 32.61)	1048	18046	58.07 (54.56 to 61.59)
	IMD=6	575	14033	40.97 (37.63to 44.32)	1061	18226	58.21 (54.71 to 61.72)	439	13985.54	31.39 (28.45 to 34.33)	1134	18226	62.22 (58.60 to 65.84)
	IMD=7	540	13846.81	39.00 (35.71to 42.29)	989	17848	55.41 (51.96 to 58.87)	429	13794.92	31.10 (28.16 to 34.04)	1129	17848	63.26 (59.57 to 66.95)
	IMD=8	438	10393.22	42.14 (38.20to 46.09)	794	13497	58.83 (54.74 to 62.92)	317	10339.3	30.66 (27.28 to 34.03)	869	13497	64.38 (60.10 to 68.67)
	IMD=9	441	10621.31	41.52 (37.65to 45.40)	824	13799	59.71 (55.64 to 63.79)	350	10512.03	33.30 (29.81 to 36.78)	939	13799	68.05 (63.70 to 72.40)
	Most Deprivation	361	9146.092	39.47 (35.40to 43.54)	644	11860	54.30 (50.11 to 58.49)	262	9130.319	28.70 (25.22 to 32.17)	716	11860	60.37 (55.95 to 64.79)
2013	Least Deprivation	714	16917.59	42.20 (39.11to 45.30)	1337	22461	59.53 (56.33 to 62.72)	536	17390.88	30.82 (28.21 to 33.43)	1266	22461	56.36 (53.26 to 59.47)
	IMD=2	753	17137.06	43.94 (40.80to 47.08)	1326	22733	58.33 (55.19 to 61.47)	573	17394.99	32.94 (30.24 to 35.64)	1345	22733	59.17 (56.00 to 62.33)
	IMD=3	711	16492.41	43.11 (39.94to 46.28)	1287	22074	58.30 (55.12 to 61.49)	579	16730.57	34.61 (31.79 to 37.43)	1402	22074	63.51 (60.19 to 66.84)
	IMD=4	607	14073.06	43.13 (39.70to 46.56)	1062	18707	56.77 (53.36 to 60.18)	433	14178.23	30.54 (27.66 to 33.42)	1067	18707	57.04 (53.62 to 60.46)
	IMD=5	540	13749.54	39.27 (35.96to 42.59)	996	18247	54.58 (51.19 to 57.97)	409	13912.76	29.40 (26.55 to 32.25)	1052	18247	57.65 (54.17 to 61.14)
	IMD=6	540	13766.83	39.22 (35.92to 42.53)	1001	18358	54.53 (51.15 to 57.90)	419	13765.17	30.44 (27.52 to 33.35)	1068	18358	58.18 (54.69 to 61.67)
	IMD=7	556	13381.32	41.55 (38.10to 45.00)	1013	18026	56.20 (52.74 to 59.66)	406	13420.46	30.25 (27.31 to 33.20)	1103	18026	61.19 (57.58 to 64.80)
	IMD=8	390	10139.94	38.46 (34.64to 42.28)	743	13674	54.34 (50.43 to 58.24)	330	10142.36	32.54 (29.03 to 36.05)	867	13674	63.41 (59.18 to 67.63)
	IMD=9	425	10335.07	41.12 (37.21to 45.03)	774	13863	55.83 (51.90 to 59.77)	339	10248.68	33.08 (29.56 to 36.60)	904	13863	65.21 (60.96 to 69.46)
	Most Deprivation	362	8577.44	42.20 (37.86to 46.55)	651	11738	55.46 (51.20 to 59.72)	261	8597.355	30.36 (26.68 to 34.04)	690	11738	58.78 (54.40 to 63.17)
2014	Least Deprivation	768	17401.13	44.14 (41.01to 47.26)	1361	22898	59.44 (56.28 to 62.60)	600	17848.15	33.62 (30.93 to 36.31)	1257	22898	54.90 (51.86 to 57.93)
	IMD=2	768	17421.06	44.08 (40.97to 47.20)	1353	22891	59.11 (55.96 to 62.26)	576	17700.39	32.54 (29.88 to 35.20)	1315	22891	57.45 (54.34 to 60.55)
	IMD=3	715	16624.14	43.01 (39.86to 46.16)	1275	21991	57.98 (54.80 to 61.16)	562	16879.45	33.29 (30.54 to 36.05)	1351	21991	61.43 (58.16 to 64.71)
	IMD=4	600	14105.2	42.54 (39.13to 45.94)	1050	18527	56.67 (53.25 to 60.10)	510	14250.54	35.79 (32.68 to 38.89)	1104	18527	59.59 (56.07 to 63.10)
	IMD=5	634	13852.21	45.77 (42.21to 49.33)	1077	18196	59.19 (55.65 to 62.72)	505	14006.49	36.05 (32.91 to 39.20)	1058	18196	58.14 (54.64 to 61.65)
	IMD=6	561	13764.96	40.76 (37.38to 44.13)	1040	18060	57.59 (54.09 to 61.09)	454	13776.71	32.95 (29.92 to 35.99)	1067	18060	59.08 (55.54 to 62.63)
	IMD=7	549	13123.33	41.83 (38.33to 45.33)	1001	17235	58.08 (54.48 to 61.68)	441	13114.23	33.63 (30.49 to 36.77)	1091	17235	63.30 (59.55 to 67.06)
	IMD=8	413	10046.76	41.11 (37.14to 45.07)	769	13219	58.17 (54.06 to 62.29)	367	10054.69	36.50 (32.77 to 40.23)	868	13219	65.66 (61.29 to 70.03)
	IMD=9	432	10058.97	42.95 (38.90to 47.00)	786	13323	59.00 (54.87 to 63.12)	366	9983.288	36.66 (32.91 to 40.42)	910	13323	68.30 (63.87 to 72.74)
	Most Deprivation	356	8273.752	43.03 (38.56to 47.50)	626	11030	56.75 (52.31 to 61.20)	287	8287.165	34.63 (30.63 to 38.64)	663	11030	60.11 (55.53 to 64.68)
2015	Least Deprivation	809	18090.49	44.72 (41.64to 47.80)	1492	23899	62.43 (59.26 to 65.60)	647	18552.15	34.87 (32.19 to 37.56)	1355	23899	56.70 (53.68 to 59.72)
	IMD=2	798	17773.41	44.90 (41.78to 48.01)	1379	23637	58.34 (55.26 to 61.42)	664	18054.18	36.78 (33.98 to 39.58)	1443	23637	61.05 (57.90 to 64.20)
	IMD=3	719	16932.82	42.46 (39.36to 45.57)	1291	22496	57.39 (54.26 to 60.52)	618	17084.22	36.17 (33.32 to 39.03)	1406	22496	62.50 (59.23 to 65.77)
	IMD=4	637	14295.6	44.56 (41.10to 48.02)	1099	18976	57.92 (54.49 to 61.34)	525	14441.1	36.35 (33.24 to 39.46)	1141	18976	60.13 (56.64 to 63.62)

	IMD=5	580	14017.89	41.38 (38.01to 44.74)	1006	18540	54.26 (50.91 to 57.61)	498	14194.59	35.08 (32.00 to 38.17)	1095	18540	59.06 (55.56 to 62.56)
	IMD=6	605	13831.56	43.74 (40.26to 47.23)	1043	18276	57.07 (53.61 to 60.53)	508	13794.73	36.83 (33.62 to 40.03)	1114	18276	60.95 (57.37 to 64.53)
	IMD=7	553	13180.16	41.96 (38.46to 45.45)	998	17428	57.26 (53.71 to 60.82)	455	13117.09	34.69 (31.50 to 37.87)	1115	17428	63.98 (60.22 to 67.73)
	IMD=8	424	9988.367	42.45 (38.41to 46.49)	768	13349	57.53 (53.46 to 61.60)	328	9987.953	32.84 (29.29 to 36.39)	807	13349	60.45 (56.28 to 64.63)
	IMD=9	399	9828.73	40.60 (36.61to 44.58)	735	13276	55.36 (51.36 to 59.37)	354	9732.747	36.37 (32.58 to 40.16)	893	13276	67.26 (62.85 to 71.68)
	Most Deprivation	353	8208.906	43.00 (38.52to 47.49)	616	11050	55.75 (51.34 to 60.15)	306	8183.69	37.39 (33.20 to 41.58)	689	11050	62.35 (57.70 to 67.01)
2016	Least Deprivation	847	19064.4	44.43 (41.44to 47.42)	1511	24888	60.71 (57.65 to 63.77)	661	19414.36	34.05 (31.45 to 36.64)	1373	24888	55.17 (52.25 to 58.09)
	IMD=2	838	18423.87	45.48 (42.40to 48.56)	1438	24217	59.38 (56.31 to 62.45)	718	18604.12	38.59 (35.77 to 41.42)	1470	24217	60.70 (57.60 to 63.80)
	IMD=3	773	17412.19	44.39 (41.26to 47.52)	1318	22815	57.77 (54.65 to 60.89)	611	17466.62	34.98 (32.21 to 37.75)	1363	22815	59.74 (56.57 to 62.91)
	IMD=4	696	14693.7	47.37 (43.85to 50.89)	1155	19296	59.86 (56.40 to 63.31)	511	14772.19	34.59 (31.59 to 37.59)	1121	19296	58.09 (54.69 to 61.50)
	IMD=5	590	14310	41.23 (37.90to 44.56)	1010	18729	53.93 (50.60 to 57.25)	495	14402.71	34.37 (31.34 to 37.40)	1101	18729	58.79 (55.31 to 62.26)
	IMD=6	607	14063.72	43.16 (39.73to 46.59)	1050	18431	56.97 (53.52 to 60.42)	494	14029.05	35.21 (32.11 to 38.32)	1122	18431	60.88 (57.31 to 64.44)
	IMD=7	536	13414.61	39.96 (36.57to 43.34)	971	17641	55.04 (51.58 to 58.50)	476	13353.71	35.65 (32.44 to 38.85)	1110	17641	62.92 (59.22 to 66.62)
	IMD=8	419	10071.84	41.60 (37.62to 45.58)	728	13266	54.88 (50.89 to 58.86)	364	10049.33	36.22 (32.50 to 39.94)	838	13266	63.17 (58.89 to 67.45)
	IMD=9	437	9831.201	44.45 (40.28to 48.62)	750	13047	57.48 (53.37 to 61.60)	345	9696.721	35.58 (31.82 to 39.33)	884	13047	67.76 (63.29 to 72.22)
	Most Deprivation	293	8209.358	35.69 (31.60to 39.78)	560	10958	51.10 (46.87 to 55.34)	306	8134.352	37.62 (33.40 to 41.83)	680	10958	62.06 (57.39 to 66.72)
2017	Least Deprivation	864	19749.49	43.75 (40.83to 46.67)	1479	25903	57.10 (54.19 to 60.01)	728	20123.11	36.18 (33.55 to 38.81)	1445	25903	55.79 (52.91 to 58.66)
	IMD=2	820	18940.07	43.29 (40.33to 46.26)	1396	24904	56.06 (53.11 to 59.00)	635	19173.24	33.12 (30.54 to 35.70)	1388	24904	55.73 (52.80 to 58.67)
	IMD=3	754	18086.9	41.69 (38.71to 44.66)	1283	23613	54.33 (51.36 to 57.31)	642	18072.27	35.52 (32.78 to 38.27)	1328	23613	56.24 (53.22 to 59.27)
	IMD=4	649	14948.7	43.42 (40.07to 46.76)	1108	19758	56.08 (52.78 to 59.38)	492	15103.44	32.58 (29.70 to 35.45)	1068	19758	54.05 (50.81 to 57.30)
	IMD=5	593	14509.83	40.87 (37.58to 44.16)	1006	19132	52.58 (49.33 to 55.83)	553	14594.96	37.89 (34.73 to 41.05)	1151	19132	60.16 (56.69 to 63.64)
	IMD=6	577	14155.38	40.76 (37.44to 44.09)	979	18736	52.25 (48.98 to 55.53)	500	14077.13	35.52 (32.41 to 38.63)	1092	18736	58.28 (54.83 to 61.74)
	IMD=7	571	13421.02	42.55 (39.06to 46.03)	934	17733	52.67 (49.29 to 56.05)	519	13355.52	38.86 (35.52 to 42.20)	1121	17733	63.22 (59.51 to 66.92)
	IMD=8	440	10142.05	43.38 (39.33to 47.44)	762	13388	56.92 (52.88 to 60.96)	372	10056.02	36.99 (33.23 to 40.75)	855	13388	63.86 (59.58 to 68.14)
	IMD=9	393	9740.194	40.35 (36.36to 44.34)	695	13016	53.40 (49.43 to 57.37)	315	9607.748	32.79 (29.17 to 36.41)	798	13016	61.31 (57.06 to 65.56)
	Most Deprivation	340	8196.679	41.48 (37.07to 45.89)	577	10932	52.78 (48.47 to 57.09)	314	8057.947	38.97 (34.66 to 43.28)	738	10932	67.51 (62.64 to 72.38)
2018	Least Deprivation	873	20444.69	42.70 (39.87to 45.53)	1434	26790	53.53 (50.76 to 56.30)	725	20757.98	34.93 (32.38 to 37.47)	1459	26790	54.46 (51.67 to 57.26)
	IMD=2	814	19538.61	41.66 (38.80to 44.52)	1388	25795	53.81 (50.98 to 56.64)	687	19687.3	34.90 (32.29 to 37.51)	1393	25795	54.00 (51.17 to 56.84)
	IMD=3	772	18456.41	41.83 (38.88to 44.78)	1278	24274	52.65 (49.76 to 55.54)	659	18363.13	35.89 (33.15 to 38.63)	1398	24274	57.59 (54.57 to 60.61)
	IMD=4	609	15342.95	39.69 (36.54to 42.85)	1061	20287	52.30 (49.15 to 55.45)	537	15470.1	34.71 (31.78 to 37.65)	1083	20287	53.38 (50.20 to 56.56)

	IMD=5	626	14946.58	41.88 (38.60to 45.16)	1027	19682	52.18 (48.99 to 55.37)	532	14895.98	35.71 (32.68 to 38.75)	1099	19682	55.84 (52.54 to 59.14)
	IMD=6	540	14406.04	37.48 (34.32to 40.65)	923	19028	48.51 (45.38 to 51.64)	453	14298.88	31.68 (28.76 to 34.60)	1068	19028	56.13 (52.76 to 59.49)
	IMD=7	529	13394.57	39.49 (36.13to 42.86)	896	17820	50.28 (46.99 to 53.57)	458	13282.94	34.48 (31.32 to 37.64)	1012	17820	56.79 (53.29 to 60.29)
	IMD=8	390	10147.91	38.43 (34.62to 42.25)	667	13479	49.48 (45.73 to 53.24)	409	9996.375	40.91 (36.95 to 44.88)	889	13479	65.95 (61.62 to 70.29)
	IMD=9	367	9705.347	37.81 (33.95to 41.68)	653	12983	50.30 (46.44 to 54.15)	371	9553.714	38.83 (34.88 to 42.78)	805	12983	62.00 (57.72 to 66.29)
	Most Deprivation	353	8148.191	43.32 (38.80to 47.84)	618	10828	57.07 (52.57 to 61.57)	298	7936.827	37.55 (33.28 to 41.81)	676	10828	62.43 (57.72 to 67.14)
2019	Least Deprivation	876	21116.3	41.48 (38.74to 44.23)	1462	27752	52.68 (49.98 to 55.38)	836	21310.76	39.23 (36.57 to 41.89)	1525	27752	54.95 (52.19 to 57.71)
	IMD=2	856	19927.78	42.96 (40.08to 45.83)	1388	26409	52.56 (49.79 to 55.32)	747	19995.86	37.36 (34.68 to 40.04)	1515	26409	57.37 (54.48 to 60.26)
	IMD=3	747	18538.68	40.29 (37.40to 43.18)	1200	24829	48.33 (45.60 to 51.07)	661	18354.28	36.01 (33.27 to 38.76)	1398	24829	56.31 (53.35 to 59.26)
	IMD=4	618	15322.79	40.33 (37.15to 43.51)	1030	20578	50.05 (47.00 to 53.11)	540	15342.86	35.20 (32.23 to 38.16)	1087	20578	52.82 (49.68 to 55.96)
	IMD=5	603	15147.4	39.81 (36.63to 42.99)	1001	19999	50.05 (46.95 to 53.15)	518	15066.03	34.38 (31.42 to 37.34)	1097	19999	54.85 (51.61 to 58.10)
	IMD=6	641	14446.58	44.37 (40.94to 47.81)	1016	19135	53.10 (49.83 to 56.36)	462	14413.01	32.05 (29.13 to 34.98)	1056	19135	55.19 (51.86 to 58.52)
	IMD=7	531	13523.07	39.27 (35.93to 42.61)	911	17926	50.82 (47.52 to 54.12)	469	13275.09	35.33 (32.13 to 38.53)	1013	17926	56.51 (53.03 to 59.99)
	IMD=8	422	10061.42	41.94 (37.94to 45.94)	686	13448	51.01 (47.19 to 54.83)	357	9764.947	36.56 (32.77 to 40.35)	805	13448	59.86 (55.73 to 64.00)
	IMD=9	392	9495.4	41.28 (37.20to 45.37)	672	12778	52.59 (48.61 to 56.57)	326	9345.667	34.88 (31.10 to 38.67)	768	12778	60.10 (55.85 to 64.35)
	Most Deprivation	319	7739.666	41.22 (36.69to 45.74)	532	10339	51.46 (47.08 to 55.83)	238	7522.976	31.64 (27.62 to 35.66)	577	10339	55.81 (51.25 to 60.36)

Supplementary Table S5. Slope index of inequality (SII) and relative index of inequality (RII) for incidence and prevalence of low back pain and osteoarthritis by age-strata between 2004-2019
SII indicates slope index of inequality; RII indicates relative index of inequality; CI indicates confidence interval.

Year	Linear model							
	Incidence				Prevalence			
	LBP		OA		LBP		OA	
	SII (95%CI)	RII (95% CI)	SII (95%CI)	RII (95% CI)	SII (95%CI)	RII (95% CI)	SII (95%CI)	RII (95% CI)
	15-24 years							
2004	5.44 (4.16-6.72)	1.274 (1.210-1.339)	----	----	7.37 (6.04-8.70)	1.317 (1.260-1.374)	----	----
2005	4.56 (3.28-5.85)	1.224 (1.161-1.287)	----	----	6.53 (5.20-7.86)	1.272 (1.216-1.327)	----	----
2006	3.68 (2.41-4.94)	1.180 (1.118-1.242)	----	----	5.44 (4.13-6.75)	1.225 (1.170-1.279)	----	----
2007	4.22 (2.95-5.49)	1.200 (1.140-1.261)	----	----	6.19 (4.87-7.52)	1.246 (1.194-1.299)	----	----
2008	6.44 (5.21-7.67)	1.315 (1.255-1.376)	----	----	9.02 (7.72-10.32)	1.361 (1.309-1.413)	----	----
2009	6.73 (5.49-7.98)	1.319 (1.260-1.378)	----	----	9.20 (7.91-10.50)	1.361 (1.310-1.412)	----	----
2010	6.41 (5.19-7.64)	1.307 (1.249-1.366)	----	----	9.59 (8.30-10.87)	1.376 (1.326-1.426)	----	----
2011	3.97 (2.74-5.21)	1.185 (1.128-1.242)	----	----	7.10 (5.81-8.39)	1.271 (1.222-1.320)	----	----
2012	7.50 (6.29-8.72)	1.355 (1.298-1.413)	----	----	10.13 (8.84-11.41)	1.387 (1.338-1.437)	----	----
2013	7.35 (6.15-8.55)	1.354 (1.296-1.412)	----	----	10.15 (8.89-11.41)	1.396 (1.347-1.445)	----	----
2014	7.21 (6.01-8.41)	1.346 (1.289-1.404)	----	----	10.55 (9.29-11.81)	1.411 (1.362-1.460)	----	----
2015	8.81 (7.63-9.99)	1.426 (1.369-1.483)	----	----	11.96 (10.72-13.20)	1.471 (1.422-1.520)	----	----
2016	8.73 (7.62-9.85)	1.461 (1.403-1.520)	----	----	11.69 (10.50-12.88)	1.494 (1.444-1.544)	----	----
2017	8.43 (7.35-9.50)	1.468 (1.409-1.528)	----	----	11.87 (10.74-13.00)	1.542 (1.491-1.594)	----	----
2018	7.44 (6.41-8.46)	1.434 (1.374-1.494)	----	----	10.63 (9.55-11.72)	1.509 (1.457-1.561)	----	----
2019	7.95 (6.96-8.94)	1.493 (1.431-1.554)	----	----	10.74 (9.72-11.77)	1.560 (1.507-1.614)	----	----
	25-34 years							
2004	6.33 (4.87-7.79)	1.225 (1.173-1.277)	----	----	11.88 (10.34-13.42)	1.328 (1.286-1.371)	----	----
2005	7.05 (5.59-8.52)	1.248 (1.196-1.300)	----	----	12.49 (10.93-14.04)	1.337 (1.295-1.379)	----	----
2006	6.81 (5.36-8.26)	1.245 (1.193-1.297)	----	----	11.33 (9.79-12.88)	1.307 (1.265-1.348)	----	----
2007	10.08 (8.62-11.54)	1.359 (1.307-1.411)	----	----	16.09 (14.54-17.65)	1.427 (1.386-1.469)	----	----
2008	8.31 (6.85-9.76)	1.293 (1.242-1.345)	----	----	14.77 (13.21-16.33)	1.387 (1.346-1.427)	----	----
2009	9.52 (8.05-10.99)	1.326 (1.276-1.376)	----	----	17.24 (15.67-18.82)	1.434 (1.394-1.473)	----	----
2010	10.53 (9.09-11.97)	1.362 (1.313-1.412)	----	----	18.52 (16.98-20.06)	1.471 (1.431-1.510)	----	----
2011	10.55 (9.13-11.98)	1.363 (1.314-1.412)	----	----	18.50 (16.97-20.03)	1.463 (1.425-1.502)	----	----
2012	12.59 (11.16-14.03)	1.418 (1.371-1.466)	----	----	21.12 (19.58-22.66)	1.511 (1.474-1.548)	----	----
2013	13.15 (11.71-14.58)	1.431 (1.384-1.478)	----	----	21.37 (19.85-22.90)	1.519 (1.482-1.555)	----	----
2014	13.83 (12.41-15.25)	1.458 (1.411-1.505)	----	----	22.88 (21.36-24.40)	1.557 (1.520-1.594)	----	----
2015	15.97 (14.58-17.36)	1.542 (1.495-1.589)	----	----	26.44 (24.95-27.94)	1.653 (1.616-1.690)	----	----
2016	14.83 (13.51-16.15)	1.541 (1.493-1.589)	----	----	25.91 (24.48-27.35)	1.678 (1.640-1.715)	----	----
2017	13.77 (12.50-15.05)	1.522 (1.474-1.570)	----	----	23.85 (22.47-25.23)	1.656 (1.618-1.694)	----	----
2018	14.03 (12.81-15.24)	1.563 (1.514-1.612)	----	----	23.76 (22.45-25.08)	1.696 (1.657-1.734)	----	----

**2019**	10.75 (9.58-11.93)	1.460 (1.410-1.510)	----	----	19.75 (18.50-20.99)	1.631 (1.591-1.671)	----	----
	**35-44 years**							
**2004**	8.61 (7.25-9.97)	1.247 (1.208-1.286)	----	----	16.80 (15.33-18.27)	1.355 (1.324-1.386)	----	----
**2005**	9.81 (8.46-11.17)	1.283 (1.243-1.322)	----	----	18.72 (17.24-20.20)	1.386 (1.356-1.417)	----	----
**2006**	9.45 (8.10-10.79)	1.273 (1.234-1.312)	----	----	19.14 (17.66-20.61)	1.390 (1.360-1.420)	----	----
**2007**	10.85 (9.49-12.21)	1.309 (1.270-1.348)	----	----	21.25 (19.76-22.74)	1.424 (1.394-1.454)	----	----
**2008**	10.64 (9.28-11.99)	1.305 (1.266-1.344)	----	----	22.14 (20.65-23.64)	1.438 (1.408-1.467)	----	----
**2009**	12.34 (10.96-13.73)	1.343 (1.305-1.382)	----	----	25.39 (23.86-26.93)	1.483 (1.454-1.512)	----	----
**2010**	10.79 (9.40-12.17)	1.300 (1.262-1.339)	----	----	24.85 (23.31-26.39)	1.468 (1.439-1.497)	----	----
**2011**	12.12 (10.71-13.52)	1.331 (1.293-1.370)	----	----	26.02 (24.47-27.57)	1.484 (1.455-1.513)	----	----
**2012**	15.46 (14.03-16.88)	1.418 (1.380-1.457)	----	----	30.69 (29.11-32.27)	1.561 (1.532-1.590)	----	----
**2013**	15.84 (14.39-17.29)	1.428 (1.389-1.467)	----	----	30.91 (29.33-32.50)	1.571 (1.542-1.600)	----	----
**2014**	16.08 (14.62-17.54)	1.433 (1.394-1.472)	----	----	32.81 (31.20-34.41)	1.602 (1.573-1.631)	----	----
**2015**	16.82 (15.37-18.26)	1.462 (1.422-1.501)	----	----	33.69 (32.10-35.29)	1.628 (1.599-1.658)	----	----
**2016**	14.77 (13.37-16.17)	1.427 (1.386-1.467)	----	----	31.03 (29.47-32.58)	1.606 (1.576-1.636)	----	----
**2017**	14.17 (12.82-15.51)	1.435 (1.394-1.476)	----	----	29.90 (28.41-31.40)	1.619 (1.588-1.650)	----	----
**2018**	14.84 (13.55-16.12)	1.482 (1.440-1.523)	----	----	30.14 (28.72-31.57)	1.666 (1.635-1.698)	----	----
**2019**	14.17 (12.91-15.42)	1.476 (1.434-1.518)	----	----	27.03 (25.67-28.40)	1.638 (1.606-1.670)	----	----
	**45-54 years**							
**2004**	8.69 (7.16-10.21)	1.232 (1.191-1.272)	4.07 (3.38-4.76)	1.484 (1.402-1.565)	17.97 (16.31-19.63)	1.351 (1.318-1.383)	7.74 (6.93-8.56)	1.626 (1.560-1.692)
**2005**	10.87 (9.33-12.41)	1.282 (1.243-1.322)	4.67 (4.01-5.33)	1.584 (1.501-1.667)	20.69 (19.02-22.37)	1.385 (1.354-1.416)	8.61 (7.82-9.41)	1.713 (1.647-1.779)
**2006**	11.44 (9.91-12.97)	1.295 (1.255-1.334)	3.67 (3.03-4.31)	1.480 (1.397-1.563)	23.13 (21.46-24.80)	1.418 (1.388-1.448)	7.75 (6.98-8.52)	1.661 (1.596-1.727)
**2007**	12.06 (10.55-13.58)	1.310 (1.272-1.349)	3.09 (2.46-3.71)	1.408 (1.326-1.491)	25.69 (24.03-27.35)	1.455 (1.426-1.485)	6.80 (6.05-7.55)	1.592 (1.527-1.658)
**2008**	12.12 (10.65-13.59)	1.318 (1.279-1.356)	3.79 (3.17-4.42)	1.486 (1.406-1.565)	27.25 (25.62-28.89)	1.483 (1.454-1.512)	7.77 (7.03-8.52)	1.661 (1.598-1.725)
**2009**	14.23 (12.74-15.71)	1.357 (1.320-1.394)	4.67 (4.05-5.29)	1.591 (1.513-1.669)	30.09 (28.45-31.72)	1.514 (1.486-1.542)	8.81 (8.08-9.55)	1.750 (1.688-1.813)
**2010**	15.67 (14.22-17.11)	1.398 (1.361-1.435)	4.08 (3.48-4.68)	1.516 (1.440-1.593)	33.13 (31.52-34.74)	1.561 (1.534-1.588)	8.37 (7.66-9.09)	1.711 (1.650-1.773)
**2011**	16.09 (14.64-17.54)	1.395 (1.360-1.431)	3.56 (2.98-4.15)	1.468 (1.392-1.545)	35.15 (33.54-36.76)	1.581 (1.554-1.608)	8.30 (7.60-9.00)	1.722 (1.661-1.783)
**2012**	18.59 (17.14-20.04)	1.454 (1.418-1.489)	4.37 (3.78-4.95)	1.560 (1.485-1.635)	40.04 (38.43-41.65)	1.648 (1.622-1.674)	8.70 (8.00-9.40)	1.745 (1.686-1.805)
**2013**	20.68 (19.24-22.13)	1.503 (1.468-1.538)	5.22 (4.62-5.82)	1.626 (1.554-1.699)	41.69 (40.10-43.28)	1.679 (1.653-1.705)	9.81 (9.1-10.51)	1.813 (1.755-1.872)
**2014**	19.83 (18.39-21.27)	1.481 (1.446-1.516)	5.37 (4.75-5.99)	1.603 (1.533-1.673)	41.00 (39.41-42.58)	1.668 (1.642-1.694)	10.10 (9.38-10.82)	1.795 (1.738-1.852)
**2015**	19.04 (17.63-20.45)	1.477 (1.442-1.512)	5.96 (5.34-6.59)	1.654 (1.585-1.722)	40.81 (39.26-42.37)	1.684 (1.658-1.710)	10.86 (10.12-11.59)	1.821 (1.766-1.877)
**2016**	18.25 (16.88-19.62)	1.474 (1.439-1.510)	5.11 (4.48-5.74)	1.542 (1.475-1.609)	39.33 (37.81-40.86)	1.681 (1.654-1.707)	10.82 (10.08-11.57)	1.789 (1.735-1.843)
**2017**	17.70 (16.36-19.05)	1.476 (1.440-1.512)	5.18 (4.55-5.82)	1.538 (1.472-1.604)	38.73 (37.25-40.21)	1.701 (1.674-1.728)	10.76 (10.01-11.50)	1.765 (1.712-1.818)
**2018**	17.48 (16.17-18.78)	1.488 (1.451-1.524)	5.08 (4.44-5.71)	1.525 (1.460-1.591)	36.03 (34.59-37.47)	1.686 (1.659-1.714)	10.22 (9.48-10.96)	1.731 (1.678-1.784)
**2019**	19.29 (17.99-20.60)	1.552 (1.514-1.589)	4.66 (4.01-5.32)	1.467 (1.401-1.533)	35.71 (34.30-37.12)	1.715 (1.687-1.743)	9.45 (8.70-10.20)	1.668 (1.615-1.721)
	**55-64 years**							
**2004**	10.09 (8.46-11.73)	1.260 (1.218-1.302)	6.05 (4.91-7.20)	1.299 (1.243-1.356)	15.92 (14.16-17.68)	1.303 (1.270-1.337)	12.76 (11.39-14.13)	1.402 (1.359-1.446)

**2005**	10.75 (9.12-12.39)	1.273 (1.231-1.314)	6.36 (5.24-7.47)	1.322 (1.265-1.379)	18.16 (16.38-19.94)	1.333 (1.300-1.365)	14.93 (13.56-16.3)	1.460 (1.418-1.502)
**2006**	9.04 (7.42-10.66)	1.231 (1.189-1.272)	5.88 (4.81-6.94)	1.319 (1.261-1.378)	18.35 (16.58-20.12)	1.329 (1.297-1.361)	15.32 (14.00-16.65)	1.491 (1.448-1.533)
**2007**	11.29 (9.67-12.92)	1.285 (1.244-1.326)	6.40 (5.33-7.46)	1.346 (1.288-1.403)	20.75 (18.98-22.53)	1.365 (1.334-1.397)	15.30 (13.98-16.63)	1.486 (1.444-1.528)
**2008**	9.90 (8.29-11.52)	1.251 (1.210-1.291)	7.25 (6.18-8.33)	1.377 (1.321-1.433)	23.13 (21.34-24.91)	1.398 (1.368-1.429)	17.58 (16.25-18.91)	1.543 (1.502-1.584)
**2009**	13.00 (11.37-14.62)	1.323 (1.283-1.364)	5.38 (4.29-6.47)	1.273 (1.218-1.328)	26.83 (25.04-28.62)	1.454 (1.424-1.484)	17.12 (15.77-18.46)	1.518 (1.477-1.559)
**2010**	14.30 (12.68-15.92)	1.354 (1.314-1.394)	6.98 (5.91-8.06)	1.358 (1.303-1.413)	28.01 (26.22-29.79)	1.470 (1.440-1.500)	18.17 (16.84-19.50)	1.549 (1.509-1.590)
**2011**	15.31 (13.67-16.95)	1.369 (1.329-1.409)	6.66 (5.60-7.73)	1.348 (1.292-1.403)	31.96 (30.14-33.77)	1.518 (1.488-1.547)	19.19 (17.87-20.52)	1.587 (1.547-1.627)
**2012**	14.57 (12.92-16.22)	1.350 (1.310-1.390)	7.18 (6.11-8.24)	1.375 (1.319-1.431)	33.55 (31.71-35.38)	1.535 (1.505-1.564)	19.32 (18.01-20.64)	1.596 (1.555-1.637)
**2013**	19.41 (17.73-21.10)	1.452 (1.413-1.491)	8.62 (7.52-9.72)	1.422 (1.368-1.476)	39.46 (37.61-41.30)	1.617 (1.589-1.646)	20.22 (18.9-21.54)	1.620 (1.579-1.660)
**2014**	16.91 (15.25-18.57)	1.401 (1.362-1.441)	8.85 (7.75-9.94)	1.432 (1.378-1.485)	37.56 (35.74-39.37)	1.601 (1.572-1.630)	21.12 (19.81-22.44)	1.645 (1.605-1.685)
**2015**	18.16 (16.55-19.77)	1.446 (1.406-1.486)	8.11 (7.00-9.22)	1.378 (1.326-1.429)	38.38 (36.61-40.15)	1.632 (1.603-1.661)	21.10 (19.78-22.43)	1.624 (1.585-1.663)
**2016**	19.99 (18.41-21.58)	1.491 (1.452-1.530)	10.14 (9.04-11.24)	1.466 (1.416-1.517)	38.98 (37.25-40.71)	1.652 (1.623-1.681)	23.17 (21.86-24.49)	1.675 (1.637-1.713)
**2017**	17.97 (16.44-19.51)	1.453 (1.414-1.492)	9.02 (7.93-10.12)	1.407 (1.357-1.456)	37.77 (36.10-39.44)	1.655 (1.626-1.684)	21.71 (20.41-23.00)	1.625 (1.587-1.662)
**2018**	18.49 (17.03-19.95)	1.491 (1.452-1.529)	8.68 (7.62-9.75)	1.397 (1.348-1.446)	37.12 (35.53-38.70)	1.682 (1.653-1.711)	21.37 (20.11-22.63)	1.625 (1.588-1.662)
**2019**	17.66 (16.21-19.11)	1.469 (1.430-1.507)	7.55 (6.47-8.63)	1.333 (1.285-1.38)	34.30 (32.75-35.84)	1.649 (1.620-1.678)	19.82 (18.57-21.07)	1.572 (1.536-1.609)
	**65-74 years**							
**2004**	3.52 (1.67-5.38)	1.092 (1.044-1.141)	1.60 (-0.08-3.28)	1.051 (0.997-1.104)	5.75 (3.76-7.75)	1.113 (1.073-1.152)	7.93 (5.93-9.94)	1.153 (1.114-1.192)
**2005**	3.11 (1.20-5.02)	1.078 (1.030-1.126)	4.70 (3.02-6.38)	1.151 (1.097-1.204)	7.05 (4.97-9.13)	1.127 (1.090-1.165)	11.70 (9.65-13.75)	1.217 (1.179-1.255)
**2006**	5.38 (3.44-7.33)	1.132 (1.084-1.179)	3.55 (1.93-5.16)	1.123 (1.067-1.179)	9.63 (7.51-11.75)	1.166 (1.130-1.203)	10.84 (8.83-12.85)	1.209 (1.170-1.247)
**2007**	5.47 (3.51-7.43)	1.132 (1.085-1.179)	6.01 (4.39-7.63)	1.205 (1.150-1.261)	11.71 (9.58-13.85)	1.197 (1.161-1.233)	14.16 (12.15-16.17)	1.269 (1.231-1.308)
**2008**	5.54 (3.60-7.48)	1.133 (1.087-1.180)	5.44 (3.82-7.05)	1.181 (1.127-1.235)	11.90 (9.77-14.03)	1.196 (1.161-1.231)	16.25 (14.24-18.26)	1.302 (1.264-1.339)
**2009**	9.27 (7.31-11.22)	1.216 (1.170-1.262)	5.21 (3.61-6.82)	1.173 (1.120-1.226)	17.09 (14.96-19.23)	1.274 (1.240-1.308)	16.82 (14.83-18.81)	1.310 (1.274-1.347)
**2010**	6.94 (5.02-8.86)	1.165 (1.119-1.211)	6.75 (5.16-8.34)	1.225 (1.172-1.277)	16.03 (13.92-18.14)	1.259 (1.225-1.293)	19.38 (17.41-21.35)	1.358 (1.322-1.395)
**2011**	9.44 (7.51-11.36)	1.217 (1.173-1.261)	7.04 (5.49-8.58)	1.239 (1.186-1.291)	17.66 (15.55-19.77)	1.276 (1.243-1.309)	21.37 (19.44-23.29)	1.400 (1.364-1.436)
**2012**	9.51 (7.64-11.39)	1.221 (1.177-1.265)	5.73 (4.24-7.23)	1.198 (1.147-1.250)	19.59 (17.53-21.65)	1.307 (1.275-1.339)	20.70 (18.84-22.56)	1.398 (1.363-1.434)
**2013**	10.04 (8.19-11.90)	1.233 (1.190-1.276)	6.80 (5.29-8.31)	1.227 (1.176-1.277)	20.36 (18.33-22.38)	1.323 (1.291-1.355)	19.78 (17.95-21.61)	1.385 (1.349-1.420)
**2014**	8.18 (6.35-10.01)	1.192 (1.149-1.235)	6.47 (4.95-8.00)	1.208 (1.159-1.257)	18.81 (16.82-20.80)	1.303 (1.271-1.335)	17.82 (16.00-19.63)	1.346 (1.311-1.381)
**2015**	10.26 (8.46-12.05)	1.244 (1.201-1.287)	6.13 (4.59-7.66)	1.190 (1.142-1.237)	19.78 (17.84-21.72)	1.329 (1.297-1.361)	18.28 (16.47-20.09)	1.347 (1.313-1.382)
**2016**	10.02 (8.27-11.77)	1.245 (1.202-1.288)	5.16 (3.63-6.69)	1.158 (1.111-1.204)	20.53 (18.64-22.42)	1.352 (1.320-1.385)	17.60 (15.80-19.40)	1.332 (1.298-1.366)
**2017**	11.29 (9.58-13.01)	1.282 (1.240-1.325)	5.67 (4.15-7.19)	1.172 (1.126-1.219)	19.85 (18.03-21.68)	1.358 (1.325-1.391)	17.06 (15.28-18.84)	1.322 (1.289-1.356)
**2018**	9.97 (8.32-11.62)	1.262 (1.218-1.305)	5.39 (3.87-6.91)	1.160 (1.115-1.206)	19.44 (17.68-21.20)	1.368 (1.334-1.401)	16.78 (15.02-18.55)	1.316 (1.283-1.349)
**2019**	8.98 (7.33-10.64)	1.237 (1.193-1.281)	4.06 (2.50-5.62)	1.118 (1.073-1.163)	19.11 (17.38-20.85)	1.373 (1.339-1.407)	13.73 (11.97-15.49)	1.259 (1.226-1.292)
	**75-84 years**							
**2004**	-7.69 (-9.89--5.48)	0.803 (0.747-0.860)	0.46 (-1.74-2.66)	1.012 (0.954-1.07)	-9.23 (-11.57--6.90)	0.821 (0.776-0.867)	1.58 (-1.00-4.15)	1.025 (0.984-1.066)
**2005**	-3.51 (-5.80--1.22)	0.914 (0.858-0.970)	2.27 (0.08-4.47)	1.061 (1.002-1.121)	-6.25 (-8.69--3.82)	0.887 (0.844-0.931)	4.42 (1.78-7.07)	1.067 (1.027-1.108)
**2006**	-3.92 (-6.23--1.60)	0.906 (0.850-0.962)	-0.53 (-2.67-1.62)	0.985 (0.924-1.046)	-5.26 (-7.75--2.78)	0.910 (0.867-0.952)	4.27 (1.63-6.90)	1.065 (1.025-1.106)
**2007**	-2.92 (-5.27--0.56)	0.932 (0.877-0.987)	1.97 (-0.18-4.13)	1.055 (0.995-1.116)	-3.16 (-5.69--0.63)	0.948 (0.906-0.99)	8.43 (5.78-11.08)	1.127 (1.087-1.167)

**2008**	0.52 (-1.87-2.91)	1.012 (0.957-1.066)	-0.93 (-3.09-1.22)	0.974 (0.914-1.034)	-0.15 (-2.73-2.42)	0.998 (0.957-1.039)	7.21 (4.55-9.87)	1.108 (1.068-1.147)
**2009**	2.48 (0.07-4.90)	1.055 (1.001-1.109)	2.61 (0.43-4.79)	1.071 (1.012-1.131)	2.94 (0.34-5.55)	1.045 (1.005-1.086)	11.62 (8.95-14.29)	1.171 (1.132-1.210)
**2010**	-0.13 (-2.56-2.29)	0.997 (0.944-1.051)	-0.19 (-2.33-1.95)	0.995 (0.935-1.055)	2.61 (-0.01-5.24)	1.039 (1.000-1.079)	11.94 (9.29-14.60)	1.176 (1.137-1.215)
**2011**	4.81 (2.33-7.29)	1.101 (1.049-1.153)	3.69 (1.54-5.83)	1.102 (1.043-1.161)	7.72 (5.06-10.39)	1.112 (1.073-1.150)	14.57 (11.92-17.22)	1.214 (1.175-1.253)
**2012**	4.00 (1.57-6.43)	1.087 (1.034-1.139)	4.47 (2.35-6.59)	1.125 (1.066-1.185)	9.30 (6.65-11.95)	1.135 (1.096-1.173)	18.53 (15.90-21.15)	1.275 (1.236-1.313)
**2013**	2.70 (0.26-5.15)	1.058 (1.005-1.110)	4.18 (1.99-6.36)	1.110 (1.052-1.167)	9.84 (7.20-12.48)	1.143 (1.104-1.181)	17.14 (14.52-19.77)	1.251 (1.213-1.289)
**2014**	5.78 (3.28-8.27)	1.118 (1.067-1.170)	5.27 (3.04-7.51)	1.132 (1.076-1.188)	9.21 (6.57-11.86)	1.132 (1.094-1.170)	16.26 (13.64-18.88)	1.238 (1.200-1.277)
**2015**	4.43 (1.95-6.90)	1.092 (1.041-1.144)	3.25 (1.01-5.49)	1.081 (1.025-1.137)	9.23 (6.62-11.84)	1.136 (1.098-1.175)	13.74 (11.12-16.36)	1.201 (1.163-1.239)
**2016**	1.63 (-0.80-4.05)	1.035 (0.983-1.087)	4.34 (2.07-6.60)	1.105 (1.050-1.160)	6.74 (4.18-9.30)	1.103 (1.064-1.142)	14.50 (11.88-17.13)	1.212 (1.173-1.250)
**2017**	6.45 (4.05-8.84)	1.141 (1.088-1.193)	3.92 (1.66-6.18)	1.094 (1.040-1.149)	11.86 (9.36-14.37)	1.187 (1.147-1.226)	14.74 (12.15-17.33)	1.217 (1.179-1.255)
**2018**	5.08 (2.76-7.40)	1.114 (1.062-1.166)	3.37 (1.12-5.61)	1.080 (1.027-1.133)	8.67 (6.26-11.08)	1.142 (1.103-1.182)	11.96 (9.43-14.49)	1.178 (1.140-1.216)
**2019**	4.45 (2.18-6.72)	1.103 (1.051-1.156)	1.43 (-0.83-3.69)	1.033 (0.981-1.086)	9.11 (6.78-11.44)	1.157 (1.117-1.197)	8.58 (6.09-11.08)	1.128 (1.091-1.166)
	**85+ years**							
**2004**	-10.90 (-14.50--7.31)	0.696 (0.595-0.796)	-0.31 (-3.78-3.16)	0.990 (0.883-1.098)	-14.56 (-18.13--10.99)	0.665 (0.583-0.747)	-0.48 (-4.46-3.49)	0.991 (0.917-1.065)
**2005**	-7.55 (-11.08--4.02)	0.793 (0.697-0.890)	-4.02 (-7.45--0.60)	0.880 (0.777-0.982)	-12.49 (-16.07--8.92)	0.733 (0.656-0.809)	-2.32 (-6.35-1.70)	0.961 (0.892-1.029)
**2006**	-7.24 (-10.75--3.73)	0.808 (0.714-0.901)	-2.74 (-5.94-0.45)	0.910 (0.805-1.015)	-8.75 (-12.35--5.16)	0.824 (0.752-0.896)	-0.63 (-4.50-3.24)	0.989 (0.922-1.057)
**2007**	-8.12 (-11.64--4.60)	0.792 (0.702-0.882)	0.25 (-2.94-3.44)	1.008 (0.906-1.109)	-12.89 (-16.51--9.26)	0.754 (0.685-0.823)	4.34 (0.47-8.21)	1.073 (1.008-1.138)
**2008**	-8.81 (-12.22--5.40)	0.767 (0.677-0.857)	2.58 (-0.55-5.71)	1.083 (0.982-1.183)	-10.35 (-13.91--6.79)	0.802 (0.734-0.870)	7.03 (3.20-10.87)	1.117 (1.053-1.180)
**2009**	-4.20 (-7.69--0.71)	0.895 (0.807-0.982)	3.40 (0.29-6.50)	1.109 (1.009-1.208)	-7.31 (-10.91--3.70)	0.866 (0.800-0.932)	9.06 (5.25-12.87)	1.149 (1.086-1.212)
**2010**	-6.40 (-9.81--2.98)	0.837 (0.750-0.924)	1.24 (-1.85-4.33)	1.039 (0.942-1.136)	-10.18 (-13.75--6.61)	0.815 (0.751-0.880)	10.76 (6.95-14.57)	1.173 (1.112-1.234)
**2011**	-5.59 (-9.08--2.11)	0.865 (0.781-0.949)	-1.24 (-4.28-1.80)	0.961 (0.864-1.057)	-8.91 (-12.53--5.30)	0.846 (0.783-0.908)	6.55 (2.78-10.31)	1.105 (1.045-1.166)
**2012**	-4.61 (-8.10--1.12)	0.890 (0.807-0.973)	0.62 (-2.36-3.60)	1.020 (0.923-1.118)	-4.93 (-8.55--1.31)	0.916 (0.854-0.978)	8.31 (4.60-12.02)	1.136 (1.075-1.196)
**2013**	-3.46 (-6.96-0.04)	0.916 (0.831-1.001)	-1.15 (-4.20-1.90)	0.964 (0.867-1.060)	-5.15 (-8.68--1.62)	0.908 (0.846-0.971)	4.55 (0.90-8.20)	1.076 (1.015-1.136)
**2014**	-2.42 (-6.00-1.15)	0.944 (0.860-1.027)	2.74 (-0.47-5.95)	1.079 (0.987-1.172)	-1.42 (-5.04-2.21)	0.976 (0.913-1.038)	9.78 (6.06-13.50)	1.161 (1.100-1.222)
**2015**	-3.36 (-6.93-0.21)	0.922 (0.839-1.005)	-0.25 (-3.51-3.00)	0.993 (0.902-1.084)	-5.52 (-9.09--1.95)	0.904 (0.841-0.966)	5.93 (2.22-9.64)	1.096 (1.036-1.157)
**2016**	-6.20 (-9.72--2.68)	0.855 (0.773-0.937)	0.63 (-2.60-3.86)	1.018 (0.927-1.108)	-7.41 (-10.94--3.87)	0.869 (0.807-0.932)	8.59 (4.90-12.27)	1.141 (1.080-1.201)
**2017**	-2.38 (-5.86-1.10)	0.943 (0.861-1.026)	2.62 (-0.60-5.84)	1.073 (0.983-1.163)	-4.02 (-7.46--0.58)	0.926 (0.863-0.989)	11.13 (7.51-14.75)	1.186 (1.126-1.247)
**2018**	-3.84 (-7.22--0.45)	0.905 (0.821-0.989)	2.93 (-0.29-6.15)	1.082 (0.992-1.171)	-3.00 (-6.34-0.34)	0.942 (0.878-1.007)	9.10 (5.56-12.65)	1.157 (1.096-1.218)
**2019**	-0.60 (-4.04-2.84)	0.986 (0.902-1.069)	-5.78 (-8.96--2.60)	0.835 (0.745-0.926)	-0.34 (-3.66-2.98)	0.993 (0.929-1.058)	2.53 (-0.95-6.02)	1.045 (0.983-1.107)

Supplementary Table S6. Standardised incidence and prevalence of low back pain and osteoarthritis by index of multiple derivation and geographical region between 2004-2019

For low back pain: the standard population was the population of England 2019 (ONS code: E92000001) aged 15 years and over; for osteoarthritis: the standard population was the population of England 2019 (ONS code: E92000001) aged 45 years and over.
IMD indicates index of multiple deprivation STDIR indicates standardised incidence rates; STDPR indicates standardised prevalence; CI indicates confidence interval.

Year	IMD decile	Low back pain						Osteoarthritis					
		Incidence			Prevalence			Incidence			Prevalence		
		Numerator	Denominator	STDIR (95 % CI)	Numerator	Denominator	STDIR (95 % CI)	Numerator	Denominator	STDIR (95 % CI)	Numerator	Denominator	STDIR (95 % CI)
**North East**													
2004	Least Deprivation	211	6086.596	34.48 (34.42 to 34.53)	291	6863	41.44 (41.38 to 41.50)	91	3865.936	20.59 (20.54 to 20.64)	169	4236	33.99 (33.92 to 34.05)
	IMD=2	509	16021.97	31.03 (30.98 to 31.08)	685	17912	36.90 (36.85 to 36.96)	259	9745.52	21.69 (21.63 to 21.74)	404	10732	30.11 (30.05 to 30.17)
	IMD=3	433	15760.79	26.09 (26.04 to 26.14)	590	17426	32.40 (32.35 to 32.45)	242	9707.644	19.85 (19.80 to 19.90)	363	10599	26.66 (26.61 to 26.72)
	IMD=4	428	13078.34	32.31 (32.26 to 32.36)	583	14567	38.85 (38.79 to 38.90)	193	7371.787	21.18 (21.13 to 21.23)	316	8193	30.42 (30.36 to 30.48)
	IMD=5	496	13166.03	37.00 (36.94 to 37.05)	709	14909	46.62 (46.56 to 46.69)	217	7495.212	23.60 (23.55 to 23.66)	369	8389	34.77 (34.71 to 34.83)
	IMD=6	522	14447.45	35.81 (35.75 to 35.86)	724	16278	43.70 (43.64 to 43.76)	246	8264.69	23.72 (23.66 to 23.77)	441	9322	36.49 (36.42 to 36.56)
	IMD=7	809	21803.79	36.82 (36.77 to 36.88)	1183	24783	46.94 (46.88 to 47.00)	334	11592.83	22.53 (22.48 to 22.59)	577	13059	34.18 (34.12 to 34.25)
	IMD=8	868	26181.72	33.25 (33.19 to 33.30)	1242	29379	42.12 (42.06 to 42.18)	393	14140.7	21.64 (21.59 to 21.69)	674	15951	32.25 (32.18 to 32.31)
	IMD=9	1116	30101.28	37.25 (37.19 to 37.30)	1632	34275	47.52 (47.45 to 47.58)	466	16009.11	22.84 (22.78 to 22.89)	782	18070	33.28 (33.22 to 33.35)
	Most Deprivation	1034	27484.21	38.18 (38.13 to 38.24)	1530	31399	49.06 (49.00 to 49.13)	405	14756.5	21.26 (21.21 to 21.31)	753	16777	34.22 (34.16 to 34.29)
2005	Least Deprivation	204	6132.419	32.38 (32.33 to 32.43)	307	7043	42.35 (42.29 to 42.41)	83	3957.213	17.39 (17.34 to 17.43)	175	4400	32.67 (32.61 to 32.74)
	IMD=2	484	16144.72	29.17 (29.12 to 29.22)	702	18296	36.97 (36.91 to 37.02)	244	9936.624	20.73 (20.68 to 20.78)	440	11075	32.06 (32.00 to 32.12)
	IMD=3	464	15869.94	28.40 (28.35 to 28.45)	668	17771	35.85 (35.80 to 35.91)	211	9923.948	17.02 (16.98 to 17.07)	353	10939	25.42 (25.36 to 25.47)
	IMD=4	433	12920.63	32.85 (32.80 to 32.90)	613	14684	40.23 (40.18 to 40.29)	172	7514.116	18.95 (18.91 to 19.00)	304	8393	29.00 (28.94 to 29.06)
	IMD=5	473	13116.74	35.64 (35.59 to 35.70)	701	15181	45.63 (45.57 to 45.69)	207	7589.481	22.11 (22.06 to 22.16)	387	8614	35.48 (35.41 to 35.54)
	IMD=6	463	14320.53	31.92 (31.87 to 31.97)	710	16440	42.02 (41.96 to 42.08)	217	8322.719	20.82 (20.77 to 20.87)	424	9489	34.46 (34.40 to 34.53)
	IMD=7	825	21630.86	37.92 (37.86 to 37.97)	1238	25090	48.41 (48.35 to 48.48)	346	11696.27	23.59 (23.54 to 23.65)	606	13310	35.27 (35.21 to 35.34)
	IMD=8	874	25906.71	33.75 (33.69 to 33.80)	1317	29705	44.06 (44.00 to 44.12)	449	14152.16	24.76 (24.70 to 24.81)	791	16163	37.19 (37.12 to 37.25)
	IMD=9	1022	29948.42	34.51 (34.46 to 34.57)	1597	34778	45.82 (45.76 to 45.89)	464	16073.55	22.90 (22.84 to 22.95)	865	18386	36.29 (36.22 to 36.36)
	Most Deprivation	961	27356.33	35.44 (35.39 to 35.50)	1505	31918	47.27 (47.21 to 47.34)	414	14869.04	21.85 (21.79 to 21.90)	771	17073	34.63 (34.56 to 34.69)
2006	Least Deprivation	211	6310.103	33.23 (33.17 to 33.28)	321	7259	42.89 (42.83 to 42.95)	81	4079.863	16.53 (16.48 to 16.57)	185	4537	33.57 (33.51 to 33.64)
	IMD=2	503	16402.06	29.15 (29.10 to 29.20)	725	18692	36.78 (36.73 to 36.84)	206	10281.1	16.90 (16.85 to 16.94)	390	11484	27.57 (27.51 to 27.63)

	IMD=3	455	16052.19	27.39 (27.34 to 27.44)	668	18168	35.37 (35.32 to 35.43)	206	10171.1	16.20 (16.15 to 16.24)	326	11261	22.62 (22.57 to 22.68)
	IMD=4	416	12967.2	31.63 (31.58 to 31.68)	631	14888	41.09 (41.04 to 41.15)	173	7641.032	18.78 (18.73 to 18.83)	313	8586	28.87 (28.81 to 28.93)
	IMD=5	465	13311.3	34.38 (34.33 to 34.44)	715	15528	45.18 (45.12 to 45.24)	179	7847.42	19.19 (19.14 to 19.23)	348	8886	31.54 (31.48 to 31.60)
	IMD=6	538	14367.67	36.70 (36.65 to 36.76)	809	16703	47.22 (47.16 to 47.28)	235	8441.949	22.45 (22.40 to 22.50)	439	9694	35.36 (35.29 to 35.42)
	IMD=7	829	21659.73	38.73 (38.68 to 38.79)	1311	25538	50.96 (50.90 to 51.03)	304	11969.48	20.41 (20.36 to 20.46)	602	13725	34.26 (34.20 to 34.33)
	IMD=8	956	25928.31	37.07 (37.01 to 37.12)	1447	30126	47.73 (47.67 to 47.80)	334	14305.23	18.52 (18.48 to 18.57)	675	16389	31.54 (31.48 to 31.60)
	IMD=9	1163	30160.27	39.18 (39.12 to 39.24)	1779	35366	50.47 (50.41 to 50.54)	411	16344.21	19.95 (19.90 to 20.00)	793	18775	32.49 (32.43 to 32.56)
	Most Deprivation	1071	27531.38	39.65 (39.59 to 39.70)	1655	32509	51.29 (51.23 to 51.36)	378	15066.97	20.00 (19.95 to 20.05)	728	17338	32.51 (32.45 to 32.57)
2007	Least Deprivation	232	6402.858	35.52 (35.47 to 35.58)	347	7421	45.81 (45.74 to 45.87)	96	4190.026	19.04 (18.99 to 19.08)	192	4670	33.08 (33.01 to 33.14)
	IMD=2	608	16625.73	35.59 (35.54 to 35.65)	863	19117	43.58 (43.52 to 43.64)	242	10603.66	19.13 (19.08 to 19.17)	455	11900	30.92 (30.86 to 30.99)
	IMD=3	519	16245.89	31.33 (31.28 to 31.38)	754	18554	39.63 (39.57 to 39.68)	211	10358.1	16.20 (16.16 to 16.25)	370	11507	24.79 (24.74 to 24.84)
	IMD=4	441	13078.73	33.23 (33.17 to 33.28)	643	15097	41.48 (41.42 to 41.54)	189	7839.975	19.86 (19.81 to 19.91)	335	8799	30.39 (30.33 to 30.45)
	IMD=5	458	13595.21	33.56 (33.51 to 33.61)	738	15889	45.53 (45.47 to 45.59)	192	8086.615	19.35 (19.30 to 19.40)	365	9178	31.51 (31.44 to 31.57)
	IMD=6	504	14532.71	34.26 (34.21 to 34.32)	804	16971	46.69 (46.63 to 46.75)	226	8627.288	21.46 (21.41 to 21.51)	429	9905	33.96 (33.90 to 34.03)
	IMD=7	836	21914.22	38.19 (38.13 to 38.25)	1335	26013	50.87 (50.81 to 50.94)	326	12301.28	21.57 (21.52 to 21.62)	613	14109	34.19 (34.13 to 34.26)
	IMD=8	908	26072.58	35.16 (35.11 to 35.22)	1433	30544	46.98 (46.92 to 47.04)	375	14513.3	20.64 (20.59 to 20.69)	731	16648	33.88 (33.82 to 33.95)
	IMD=9	1181	30393.13	39.26 (39.20 to 39.32)	1878	35949	52.24 (52.18 to 52.31)	479	16604.58	22.88 (22.82 to 22.93)	909	19090	36.59 (36.52 to 36.65)
	Most Deprivation	1065	27808.99	39.22 (39.16 to 39.27)	1735	33081	52.96 (52.90 to 53.03)	393	15331.84	20.33 (20.28 to 20.37)	795	17661	34.88 (34.81 to 34.94)
2008	Least Deprivation	207	6524.351	30.09 (30.04 to 30.14)	298	7553	37.25 (37.19 to 37.31)	97	4314.639	18.92 (18.87 to 18.97)	197	4803	32.91 (32.85 to 32.97)
	IMD=2	587	16813.13	34.19 (34.14 to 34.24)	869	19393	43.20 (43.14 to 43.26)	279	10903.41	21.18 (21.13 to 21.23)	490	12196	32.27 (32.21 to 32.33)
	IMD=3	468	16517.14	27.55 (27.51 to 27.60)	750	18860	38.17 (38.11 to 38.22)	248	10612.87	18.74 (18.70 to 18.79)	421	11774	27.83 (27.77 to 27.88)
	IMD=4	418	13188.05	31.33 (31.28 to 31.39)	596	15306	37.89 (37.83 to 37.94)	192	8016	19.30 (19.25 to 19.35)	342	9037	30.03 (29.97 to 30.09)
	IMD=5	505	13941.96	36.26 (36.20 to 36.31)	782	16215	47.53 (47.46 to 47.59)	191	8378.661	18.57 (18.52 to 18.62)	356	9444	29.89 (29.83 to 29.95)
	IMD=6	536	14767.68	36.43 (36.37 to 36.48)	849	17284	48.44 (48.38 to 48.51)	259	8792.192	23.77 (23.71 to 23.82)	492	10073	38.18 (38.11 to 38.25)
	IMD=7	859	22286.52	38.40 (38.35 to 38.46)	1383	26439	51.48 (51.41 to 51.54)	349	12601.67	22.22 (22.17 to 22.27)	648	14403	35.34 (35.28 to 35.41)
	IMD=8	905	26343.38	34.79 (34.74 to 34.85)	1445	31024	46.53 (46.46 to 46.59)	401	14732.68	21.85 (21.80 to 21.90)	751	16952	34.52 (34.45 to 34.58)
	IMD=9	1148	30736.09	37.59 (37.53 to 37.64)	1904	36420	52.12 (52.05 to 52.18)	481	16882.98	22.86 (22.81 to 22.92)	936	19435	37.46 (37.40 to 37.53)
	Most Deprivation	1168	27935.66	42.44 (42.38 to 42.50)	1888	33397	56.81 (56.74 to 56.88)	466	15495.81	24.42 (24.36 to 24.47)	860	17858	37.72 (37.65 to 37.79)
2009	Least Deprivation	244	6633.311	35.01 (34.95 to 35.06)	360	7707	44.32 (44.26 to 44.38)	102	4397.687	18.71 (18.66 to 18.75)	208	4920	33.16 (33.09 to 33.22)

	IMD=2	558	16961.7	32.20 (32.14 to 32.25)	870	19688	42.74 (42.68 to 42.80)	268	11172.8	19.83 (19.79 to 19.88)	508	12522	32.59 (32.53 to 32.66)
	IMD=3	530	16901.47	30.61 (30.56 to 30.66)	805	19337	40.31 (40.26 to 40.37)	256	10845.79	18.76 (18.72 to 18.81)	440	12067	28.33 (28.27 to 28.38)
	IMD=4	484	13298.21	36.19 (36.13 to 36.24)	680	15446	43.39 (43.33 to 43.45)	183	8197.454	18.05 (18.00 to 18.09)	338	9211	29.27 (29.21 to 29.33)
	IMD=5	467	14139.7	32.16 (32.11 to 32.21)	726	16528	42.89 (42.83 to 42.95)	208	8609.933	19.95 (19.90 to 20.00)	385	9719	31.72 (31.66 to 31.78)
	IMD=6	566	14983.85	37.15 (37.10 to 37.21)	914	17648	50.52 (50.46 to 50.59)	240	8945.629	21.97 (21.92 to 22.02)	494	10306	37.54 (37.47 to 37.60)
	IMD=7	969	22547.49	42.94 (42.88 to 43.00)	1525	26886	56.01 (55.95 to 56.08)	345	12938.92	21.70 (21.65 to 21.75)	664	14823	35.44 (35.38 to 35.51)
	IMD=8	1022	26459.85	39.13 (39.08 to 39.19)	1638	31296	52.59 (52.52 to 52.65)	379	14959.06	20.46 (20.41 to 20.51)	750	17181	34.29 (34.22 to 34.35)
	IMD=9	1338	30866.49	43.85 (43.79 to 43.91)	2149	36928	58.10 (58.03 to 58.17)	532	17148.09	25.12 (25.07 to 25.18)	981	19841	38.56 (38.49 to 38.62)
	Most Deprivation	1220	27892.31	44.44 (44.38 to 44.50)	2013	33720	59.93 (59.86 to 60.00)	447	15580.02	23.03 (22.98 to 23.08)	905	18082	39.23 (39.16 to 39.30)
2010	Least Deprivation	220	6823.206	31.59 (31.54 to 31.64)	336	7943	40.32 (40.27 to 40.38)	98	4505.498	17.87 (17.82 to 17.91)	205	5045	32.05 (31.99 to 32.12)
	IMD=2	600	17351.01	33.38 (33.33 to 33.43)	910	20207	43.00 (42.94 to 43.06)	233	11490.67	16.79 (16.75 to 16.84)	487	12938	29.99 (29.93 to 30.05)
	IMD=3	573	17902.97	32.16 (32.11 to 32.21)	831	20638	39.82 (39.76 to 39.88)	195	11216.97	13.90 (13.86 to 13.94)	413	12522	25.51 (25.45 to 25.56)
	IMD=4	462	13486.17	33.59 (33.54 to 33.64)	691	15709	42.82 (42.76 to 42.88)	201	8411.6	19.55 (19.51 to 19.60)	345	9461	28.79 (28.74 to 28.85)
	IMD=5	513	14356.26	35.12 (35.07 to 35.18)	784	16851	45.35 (45.29 to 45.42)	211	8886.623	19.88 (19.83 to 19.93)	378	10033	30.30 (30.24 to 30.36)
	IMD=6	556	15224.98	36.10 (36.05 to 36.16)	901	17992	49.02 (48.95 to 49.08)	239	9159.439	20.94 (20.89 to 21.00)	454	10532	33.66 (33.60 to 33.72)
	IMD=7	932	22817.96	40.78 (40.72 to 40.84)	1525	27413	54.77 (54.70 to 54.84)	366	13232.97	22.91 (22.85 to 22.96)	694	15200	36.54 (36.47 to 36.61)
	IMD=8	1084	26831.79	40.86 (40.80 to 40.92)	1740	31858	54.63 (54.56 to 54.70)	365	15176.08	19.46 (19.41 to 19.50)	739	17452	33.45 (33.39 to 33.51)
	IMD=9	1374	31046.69	44.81 (44.75 to 44.87)	2218	37424	59.16 (59.09 to 59.23)	471	17517	21.88 (21.83 to 21.93)	934	20327	35.86 (35.79 to 35.93)
	Most Deprivation	1182	27989.11	43.43 (43.37 to 43.49)	2053	34019	61.03 (60.96 to 61.10)	425	15708.43	22.16 (22.10 to 22.21)	877	18296	37.95 (37.89 to 38.02)
2011	Least Deprivation	250	6987.617	35.17 (35.12 to 35.23)	390	8155	46.00 (45.94 to 46.06)	102	4593.248	18.10 (18.05 to 18.14)	211	5170	31.59 (31.53 to 31.65)
	IMD=2	581	17662.18	31.26 (31.20 to 31.31)	903	20619	41.45 (41.39 to 41.51)	255	11731.87	17.42 (17.38 to 17.47)	503	13202	29.90 (29.84 to 29.96)
	IMD=3	580	18533.58	31.68 (31.63 to 31.73)	888	21704	40.95 (40.89 to 41.01)	237	11360.88	16.48 (16.43 to 16.52)	421	12691	25.38 (25.32 to 25.43)
	IMD=4	447	13676.57	32.17 (32.11 to 32.22)	653	15924	39.85 (39.79 to 39.91)	198	8616.684	18.76 (18.72 to 18.81)	341	9664	27.93 (27.87 to 27.98)
	IMD=5	501	14570.69	33.85 (33.80 to 33.91)	795	17120	45.43 (45.37 to 45.49)	191	9130.448	17.34 (17.29 to 17.38)	369	10300	28.74 (28.68 to 28.80)
	IMD=6	629	15376.77	40.56 (40.50 to 40.62)	998	18320	53.20 (53.13 to 53.27)	233	9303.619	20.24 (20.19 to 20.29)	465	10696	33.94 (33.87 to 34.00)
	IMD=7	956	23122.84	41.44 (41.38 to 41.49)	1570	27925	55.47 (55.41 to 55.54)	298	13529.57	18.13 (18.08 to 18.18)	596	15531	30.64 (30.58 to 30.70)
	IMD=8	1046	26753.83	39.64 (39.59 to 39.70)	1733	32197	53.95 (53.88 to 54.01)	332	15355.79	17.56 (17.51 to 17.60)	705	17648	31.49 (31.43 to 31.55)
	IMD=9	1318	31099.46	42.88 (42.82 to 42.93)	2216	37768	58.52 (58.45 to 58.59)	472	17765.93	21.54 (21.49 to 21.59)	943	20577	35.81 (35.75 to 35.88)
	Most Deprivation	1293	28046.02	47.18 (47.11 to 47.24)	2173	34399	63.78 (63.71 to 63.86)	399	15908.56	20.65 (20.60 to 20.70)	859	18513	36.93 (36.86 to 37.00)

2012	Least Deprivation	225	7009.739	30.54 (30.49 to 30.60)	373	8202	42.84 (42.78 to 42.90)	102	4653.013	17.64 (17.59 to 17.68)	201	5223	29.41 (29.35 to 29.47)
	IMD=2	568	17885.51	31.28 (31.22 to 31.33)	869	20826	40.45 (40.39 to 40.50)	279	11951.68	18.76 (18.72 to 18.81)	498	13410	28.86 (28.80 to 28.92)
	IMD=3	586	18236.31	31.77 (31.72 to 31.82)	901	21549	41.44 (41.38 to 41.50)	251	11474.44	17.13 (17.09 to 17.18)	433	12832	25.76 (25.71 to 25.82)
	IMD=4	463	13807.09	32.67 (32.61 to 32.72)	712	16060	43.18 (43.12 to 43.24)	198	8810.82	18.52 (18.47 to 18.57)	366	9886	29.32 (29.26 to 29.38)
	IMD=5	511	14665.85	34.13 (34.07 to 34.18)	805	17189	45.81 (45.75 to 45.87)	211	9316.728	18.36 (18.32 to 18.41)	375	10453	28.47 (28.41 to 28.53)
	IMD=6	569	15469.37	36.48 (36.43 to 36.54)	941	18465	50.03 (49.96 to 50.09)	235	9513.788	19.98 (19.94 to 20.03)	482	10922	34.40 (34.33 to 34.46)
	IMD=7	937	23449.13	39.87 (39.81 to 39.93)	1553	28304	53.88 (53.82 to 53.95)	373	13892.61	22.34 (22.29 to 22.40)	694	15855	34.97 (34.90 to 35.03)
	IMD=8	1020	26816.64	38.73 (38.67 to 38.78)	1684	32177	52.55 (52.49 to 52.62)	425	15441.2	22.24 (22.18 to 22.29)	779	17638	34.79 (34.73 to 34.86)
	IMD=9	1419	31183.74	45.95 (45.88 to 46.01)	2388	38015	62.73 (62.66 to 62.80)	518	17949.31	23.34 (23.28 to 23.39)	984	20770	36.95 (36.88 to 37.02)
	Most Deprivation	1236	28268.45	44.61 (44.55 to 44.67)	2183	34728	63.14 (63.07 to 63.22)	389	16153.57	19.94 (19.90 to 19.99)	810	18751	34.38 (34.32 to 34.45)
2013	Least Deprivation	219	7135.901	28.72 (28.67 to 28.77)	356	8355	39.65 (39.59 to 39.71)	113	4710.552	18.97 (18.93 to 19.02)	198	5321	28.56 (28.50 to 28.62)
	IMD=2	625	17964.32	33.54 (33.49 to 33.59)	948	21012	43.08 (43.02 to 43.14)	299	12051.47	19.92 (19.87 to 19.97)	522	13570	29.81 (29.75 to 29.87)
	IMD=3	564	18593.09	30.31 (30.26 to 30.36)	869	22012	39.23 (39.17 to 39.28)	284	11549.63	19.17 (19.13 to 19.22)	489	12954	28.61 (28.55 to 28.67)
	IMD=4	460	13812.19	33.15 (33.10 to 33.21)	715	16135	43.71 (43.65 to 43.77)	205	8918.119	18.18 (18.13 to 18.23)	356	10011	27.68 (27.62 to 27.74)
	IMD=5	474	14684.34	31.94 (31.89 to 31.99)	751	17293	42.49 (42.43 to 42.55)	198	9427.546	16.89 (16.84 to 16.93)	353	10587	26.35 (26.29 to 26.40)
	IMD=6	580	15510.15	36.94 (36.88 to 36.99)	950	18571	50.09 (50.03 to 50.15)	265	9624.372	22.03 (21.98 to 22.09)	494	11031	34.91 (34.85 to 34.98)
	IMD=7	997	23670.43	42.13 (42.07 to 42.19)	1652	28692	56.81 (56.74 to 56.87)	365	14018.77	21.43 (21.37 to 21.48)	700	16092	34.75 (34.68 to 34.81)
	IMD=8	1051	26781.39	39.77 (39.71 to 39.83)	1728	32545	53.27 (53.20 to 53.34)	363	15531.6	19.06 (19.01 to 19.10)	726	17882	32.00 (31.94 to 32.06)
	IMD=9	1338	31204.16	43.45 (43.39 to 43.51)	2288	38343	59.62 (59.55 to 59.69)	516	18081.26	23.03 (22.97 to 23.08)	966	20985	36.07 (36.00 to 36.14)
	Most Deprivation	1273	28120.39	46.38 (46.32 to 46.44)	2199	34859	63.62 (63.54 to 63.69)	442	16234.35	22.42 (22.37 to 22.47)	846	18853	35.72 (35.65 to 35.78)
2014	Least Deprivation	275	7205.785	37.08 (37.02 to 37.13)	393	8472	43.83 (43.77 to 43.89)	123	4749.952	20.76 (20.71 to 20.81)	230	5385	32.65 (32.58 to 32.71)
	IMD=2	656	18060.04	35.47 (35.41 to 35.52)	1012	21180	46.01 (45.94 to 46.07)	321	12173.26	20.80 (20.75 to 20.85)	540	13757	29.99 (29.93 to 30.05)
	IMD=3	629	18735.23	34.06 (34.01 to 34.11)	963	22385	43.18 (43.12 to 43.24)	296	11634.12	19.68 (19.63 to 19.73)	479	13098	27.46 (27.40 to 27.52)
	IMD=4	453	13863.65	31.65 (31.60 to 31.70)	700	16226	41.47 (41.41 to 41.53)	191	8990.735	17.05 (17.00 to 17.09)	362	10147	27.69 (27.63 to 27.74)
	IMD=5	548	14868.38	35.95 (35.90 to 36.01)	863	17447	47.83 (47.76 to 47.89)	255	9592.208	21.46 (21.41 to 21.51)	433	10774	31.61 (31.54 to 31.67)
	IMD=6	618	15625.36	38.86 (38.80 to 38.91)	983	18702	51.25 (51.18 to 51.32)	275	9779.132	22.50 (22.45 to 22.55)	525	11216	36.29 (36.22 to 36.36)
	IMD=7	984	23805.87	41.36 (41.30 to 41.42)	1668	28903	56.86 (56.79 to 56.93)	427	14303.69	24.61 (24.56 to 24.67)	767	16392	37.42 (37.35 to 37.49)
	IMD=8	1169	27192.83	43.55 (43.49 to 43.61)	1888	32772	57.62 (57.55 to 57.69)	408	15847.27	20.92 (20.87 to 20.98)	778	18169	33.60 (33.53 to 33.66)
	IMD=9	1413	31485.95	45.36 (45.30 to 45.42)	2402	38567	62.15 (62.08 to 62.22)	515	18334.27	22.66 (22.61 to 22.71)	968	21283	35.56 (35.49 to 35.62)

	Most Deprivation	1317	28234.19	47.87 (47.81 to 47.93)	2345	34922	67.68 (67.61 to 67.76)	462	16421.43	22.99 (22.94 to 23.05)	880	18966	36.80 (36.73 to 36.86)
2015	Least Deprivation	249	7277.221	32.54 (32.49 to 32.59)	385	8578	41.45 (41.40 to 41.51)	119	4806.53	19.61 (19.56 to 19.66)	227	5471	31.43 (31.36 to 31.49)
	IMD=2	669	18133.07	35.25 (35.19 to 35.30)	985	21311	43.80 (43.74 to 43.86)	378	12288.31	24.29 (24.24 to 24.34)	623	13957	34.04 (33.98 to 34.11)
	IMD=3	633	19017.9	33.61 (33.56 to 33.66)	934	22665	41.65 (41.59 to 41.70)	323	11725.59	21.16 (21.11 to 21.21)	530	13230	29.75 (29.69 to 29.81)
	IMD=4	423	14022.03	29.64 (29.59 to 29.69)	640	16375	38.07 (38.02 to 38.13)	245	9134.217	21.53 (21.48 to 21.58)	406	10290	30.60 (30.54 to 30.66)
	IMD=5	559	15067.9	36.59 (36.53 to 36.64)	845	17710	46.57 (46.50 to 46.63)	266	9712.066	22.18 (22.13 to 22.23)	443	10986	31.66 (31.60 to 31.72)
	IMD=6	611	15744.69	38.01 (37.96 to 38.07)	965	18830	49.48 (49.41 to 49.54)	270	9912.224	21.99 (21.94 to 22.04)	512	11400	34.79 (34.73 to 34.86)
	IMD=7	1019	24081.99	42.30 (42.24 to 42.36)	1667	29182	56.31 (56.24 to 56.38)	434	14475.82	24.70 (24.65 to 24.76)	791	16680	37.87 (37.80 to 37.94)
	IMD=8	1103	27531.04	40.56 (40.50 to 40.62)	1834	33216	55.19 (55.12 to 55.26)	492	15953.07	24.97 (24.91 to 25.02)	870	18396	37.24 (37.17 to 37.31)
	IMD=9	1335	31701.97	42.55 (42.49 to 42.61)	2342	38825	60.17 (60.09 to 60.24)	612	18483.18	26.76 (26.70 to 26.82)	1081	21481	39.54 (39.47 to 39.61)
	Most Deprivation	1315	28368.39	47.11 (47.05 to 47.18)	2315	35163	65.99 (65.92 to 66.07)	424	16511.23	20.96 (20.91 to 21.01)	879	19116	36.43 (36.36 to 36.49)
2016	Least Deprivation	246	7323.513	31.30 (31.25 to 31.35)	366	8605	39.18 (39.12 to 39.23)	126	4877.251	20.29 (20.24 to 20.34)	225	5528	30.83 (30.77 to 30.89)
	IMD=2	643	18295.8	32.96 (32.91 to 33.02)	1005	21499	43.44 (43.38 to 43.50)	338	12499.44	21.15 (21.10 to 21.20)	580	14197	30.82 (30.76 to 30.88)
	IMD=3	555	19535.32	28.73 (28.68 to 28.78)	882	22998	38.42 (38.36 to 38.47)	333	11862.69	21.68 (21.63 to 21.73)	571	13413	31.55 (31.48 to 31.61)
	IMD=4	441	14125.76	31.14 (31.09 to 31.19)	677	16434	40.43 (40.38 to 40.49)	229	9253.24	19.72 (19.67 to 19.77)	394	10429	28.96 (28.90 to 29.02)
	IMD=5	592	15242.19	38.09 (38.03 to 38.15)	920	17848	49.77 (49.71 to 49.84)	272	9921.383	22.30 (22.24 to 22.35)	460	11205	32.30 (32.24 to 32.37)
	IMD=6	590	15914.67	36.84 (36.79 to 36.90)	928	18919	47.90 (47.84 to 47.97)	278	9995.551	22.25 (22.20 to 22.30)	533	11478	35.85 (35.78 to 35.92)
	IMD=7	947	24352	38.92 (38.86 to 38.98)	1560	29495	52.18 (52.11 to 52.25)	404	14704.41	22.70 (22.65 to 22.76)	766	16978	35.96 (35.90 to 36.03)
	IMD=8	1125	27832.65	41.03 (40.97 to 41.09)	1864	33491	55.57 (55.50 to 55.63)	452	16102.76	23.14 (23.09 to 23.19)	860	18527	36.71 (36.64 to 36.77)
	IMD=9	1405	32073.16	44.33 (44.26 to 44.39)	2396	39109	61.08 (61.01 to 61.15)	580	18652.09	25.19 (25.13 to 25.24)	1110	21678	40.21 (40.14 to 40.28)
	Most Deprivation	1328	28662.47	47.24 (47.18 to 47.30)	2294	35499	64.86 (64.79 to 64.94)	487	16655.68	23.92 (23.86 to 23.97)	967	19277	39.75 (39.68 to 39.82)
2017	Least Deprivation	217	7487.507	27.48 (27.43 to 27.52)	339	8764	35.71 (35.66 to 35.76)	132	4913.027	21.11 (21.06 to 21.16)	249	5587	33.58 (33.52 to 33.64)
	IMD=2	571	18531.15	29.74 (29.69 to 29.79)	892	21800	38.71 (38.65 to 38.76)	418	12541.5	25.94 (25.89 to 26.00)	679	14377	35.28 (35.21 to 35.34)
	IMD=3	601	19921.64	30.99 (30.94 to 31.05)	893	23438	38.71 (38.65 to 38.77)	364	11891.77	23.47 (23.42 to 23.52)	602	13574	32.79 (32.73 to 32.85)
	IMD=4	473	14190.63	32.06 (32.01 to 32.11)	695	16527	40.26 (40.20 to 40.31)	241	9273.801	20.30 (20.25 to 20.35)	430	10513	31.19 (31.13 to 31.25)
	IMD=5	506	15272.59	32.18 (32.13 to 32.23)	815	18053	43.42 (43.36 to 43.48)	337	9903.91	27.41 (27.36 to 27.47)	558	11373	38.29 (38.22 to 38.36)
	IMD=6	578	16185.95	35.02 (34.97 to 35.07)	965	19238	48.67 (48.61 to 48.73)	321	10148.07	25.37 (25.31 to 25.42)	591	11707	38.87 (38.80 to 38.93)
	IMD=7	939	24760.44	37.71 (37.65 to 37.76)	1591	29955	52.27 (52.20 to 52.34)	441	14891.92	24.87 (24.82 to 24.93)	833	17269	38.45 (38.38 to 38.52)
	IMD=8	1150	28087.87	41.45 (41.39 to 41.51)	1913	33947	56.20 (56.14 to 56.27)	507	16171.03	25.46 (25.41 to 25.52)	916	18721	38.59 (38.52 to 38.66)

	IMD=9	1330	32294.69	41.68 (41.62 to 41.74)	2307	39550	58.00 (57.93 to 58.07)	620	18753.65	26.78 (26.72 to 26.84)	1155	21939	41.45 (41.38 to 41.52)
	Most Deprivation	1384	28827.09	49.18 (49.11 to 49.24)	2419	35931	67.45 (67.37 to 67.52)	534	16627.39	26.31 (26.26 to 26.37)	1033	19371	42.55 (42.48 to 42.62)
2018	Least Deprivation	211	7670.719	25.70 (25.65 to 25.74)	353	8915	36.49 (36.44 to 36.55)	142	4964.148	22.43 (22.37 to 22.48)	270	5658	35.52 (35.45 to 35.58)
	IMD=2	611	18872.64	30.78 (30.73 to 30.83)	941	22023	40.04 (39.99 to 40.10)	411	12580.26	25.22 (25.17 to 25.28)	743	14522	37.88 (37.82 to 37.95)
	IMD=3	589	20232.57	29.64 (29.59 to 29.69)	908	23698	38.67 (38.61 to 38.72)	363	11787.52	23.27 (23.22 to 23.33)	623	13561	33.64 (33.58 to 33.71)
	IMD=4	419	14255.8	27.91 (27.86 to 27.96)	634	16542	36.36 (36.30 to 36.41)	293	9234.226	24.78 (24.73 to 24.83)	497	10535	35.63 (35.57 to 35.70)
	IMD=5	555	15479.43	35.34 (35.29 to 35.40)	850	18208	45.34 (45.28 to 45.41)	359	9865.747	28.85 (28.79 to 28.91)	610	11435	41.30 (41.23 to 41.37)
	IMD=6	557	16454.76	33.24 (33.19 to 33.29)	909	19496	45.20 (45.14 to 45.27)	330	10171.33	25.67 (25.62 to 25.73)	608	11791	39.61 (39.54 to 39.68)
	IMD=7	990	25345.23	38.90 (38.84 to 38.95)	1599	30445	51.70 (51.63 to 51.76)	449	15059.06	24.50 (24.45 to 24.56)	843	17443	38.42 (38.35 to 38.49)
	IMD=8	1086	28549.88	38.40 (38.35 to 38.46)	1830	34392	52.98 (52.91 to 53.05)	511	16263.74	25.66 (25.61 to 25.72)	980	18925	40.83 (40.76 to 40.90)
	IMD=9	1415	32641.24	43.68 (43.62 to 43.74)	2369	39837	59.03 (58.96 to 59.10)	624	18745.73	26.85 (26.80 to 26.91)	1182	22066	41.94 (41.86 to 42.01)
	Most Deprivation	1228	29363.7	42.75 (42.69 to 42.81)	2183	36350	60.16 (60.09 to 60.23)	509	16576.39	25.37 (25.31 to 25.42)	997	19398	40.85 (40.78 to 40.92)
2019	Least Deprivation	239	7863.165	28.82 (28.77 to 28.87)	352	9117	36.00 (35.95 to 36.06)	156	5011.441	24.16 (24.10 to 24.21)	301	5766	38.77 (38.70 to 38.84)
	IMD=2	629	18680.57	31.79 (31.74 to 31.84)	985	22335	41.49 (41.43 to 41.55)	358	12396.81	22.05 (22.00 to 22.11)	665	14672	33.05 (32.99 to 33.11)
	IMD=3	553	20508.41	27.71 (27.66 to 27.76)	830	24083	35.15 (35.10 to 35.21)	379	11729.14	24.53 (24.48 to 24.58)	634	13607	33.95 (33.89 to 34.01)
	IMD=4	426	13618.78	30.04 (29.99 to 30.09)	644	16611	36.85 (36.79 to 36.90)	247	8753.67	21.81 (21.76 to 21.86)	446	10560	31.81 (31.75 to 31.87)
	IMD=5	525	14188.06	35.96 (35.90 to 36.01)	803	18305	41.84 (41.78 to 41.90)	291	9055.868	25.44 (25.39 to 25.50)	513	11512	34.33 (34.27 to 34.40)
	IMD=6	565	15457.34	35.34 (35.29 to 35.40)	917	19640	44.95 (44.89 to 45.01)	332	9473.215	27.55 (27.49 to 27.61)	611	11860	39.21 (39.14 to 39.28)
	IMD=7	929	24469.44	37.97 (37.91 to 38.02)	1528	30871	48.71 (48.65 to 48.78)	422	14405.14	23.91 (23.86 to 23.97)	792	17585	35.53 (35.47 to 35.60)
	IMD=8	1121	28091.57	40.24 (40.19 to 40.30)	1877	34783	53.61 (53.55 to 53.68)	530	15899.04	26.91 (26.85 to 26.97)	993	19059	40.96 (40.89 to 41.03)
	IMD=9	1325	32218.09	41.41 (41.35 to 41.47)	2255	40009	55.92 (55.85 to 55.99)	604	18380.86	26.50 (26.44 to 26.56)	1191	22074	42.20 (42.13 to 42.27)
	Most Deprivation	1243	29430.36	43.09 (43.03 to 43.15)	2199	36863	59.73 (59.66 to 59.80)	531	16245.61	26.57 (26.51 to 26.63)	1037	19459	42.05 (41.98 to 42.13)
**North West**													
2004	Least Deprivation	1948	64476.77	29.01 (28.96 to 29.06)	2989	72757	38.88 (38.82 to 38.94)	742	41687.01	14.59 (14.54 to 14.63)	1259	45269	22.17 (22.12 to 22.22)
	IMD=2	2275	66090.83	33.39 (33.34 to 33.44)	3455	75525	43.85 (43.79 to 43.91)	883	40537.19	17.39 (17.35 to 17.44)	1595	44704	27.69 (27.64 to 27.75)
	IMD=3	2627	76837.48	33.38 (33.33 to 33.43)	3958	87924	43.22 (43.16 to 43.28)	1043	47703.71	17.35 (17.30 to 17.39)	1825	52648	26.62 (26.57 to 26.68)
	IMD=4	1985	56471.26	34.28 (34.22 to 34.33)	2984	64542	44.68 (44.62 to 44.74)	721	33855.33	17.09 (17.05 to 17.14)	1318	37379	27.57 (27.51 to 27.63)
	IMD=5	1856	54816.67	33.31 (33.26 to 33.36)	2948	63031	45.54 (45.48 to 45.60)	692	32031.83	17.29 (17.24 to 17.33)	1291	35424	28.47 (28.41 to 28.53)
	IMD=6	2090	58454.96	35.35 (35.30 to 35.41)	3208	67485	46.51 (46.45 to 46.58)	869	33936.29	19.84 (19.79 to 19.89)	1430	37776	28.52 (28.46 to 28.58)

	IMD=7	2569	66232.01	38.88 (38.82 to 38.93)	3928	77290	50.43 (50.37 to 50.50)	863	36302.28	18.69 (18.65 to 18.74)	1572	40732	29.51 (29.45 to 29.57)
	IMD=8	2004	53274.35	37.98 (37.92 to 38.03)	3197	62280	51.18 (51.11 to 51.24)	760	28897.39	20.50 (20.45 to 20.55)	1387	32655	32.06 (32.00 to 32.12)
	IMD=9	2755	71344.01	39.26 (39.20 to 39.31)	4479	83456	54.03 (53.96 to 54.09)	884	38096.49	18.06 (18.02 to 18.11)	1673	42880	29.53 (29.47 to 29.59)
	Most Deprivation	4180	104461.1	41.19 (41.13 to 41.25)	6963	122875	57.76 (57.69 to 57.83)	1375	53925.59	20.36 (20.31 to 20.41)	2467	60611	31.76 (31.70 to 31.82)
2005	Least Deprivation	2054	65206.49	30.36 (30.31 to 30.41)	3159	74351	40.29 (40.23 to 40.35)	731	42440.32	14.22 (14.18 to 14.27)	1297	46412	22.30 (22.25 to 22.35)
	IMD=2	2363	66717.04	34.58 (34.53 to 34.64)	3724	77157	46.41 (46.35 to 46.48)	847	41222.19	16.48 (16.44 to 16.53)	1640	45880	27.70 (27.65 to 27.76)
	IMD=3	2708	77501.86	34.00 (33.94 to 34.05)	4212	89683	45.31 (45.25 to 45.37)	1062	48519.13	17.36 (17.31 to 17.41)	1963	53933	27.94 (27.88 to 28.00)
	IMD=4	1960	56988.16	33.69 (33.64 to 33.75)	3014	66101	44.25 (44.19 to 44.31)	719	34544.65	16.66 (16.62 to 16.71)	1408	38465	28.47 (28.41 to 28.53)
	IMD=5	1864	55934.97	33.08 (33.03 to 33.13)	3033	65024	45.57 (45.51 to 45.64)	734	33009.15	17.91 (17.86 to 17.95)	1402	36786	29.75 (29.69 to 29.81)
	IMD=6	2155	58903.65	36.40 (36.35 to 36.46)	3404	68994	48.39 (48.32 to 48.45)	823	34462.84	18.64 (18.59 to 18.68)	1472	38764	28.71 (28.65 to 28.77)
	IMD=7	2497	67150.83	37.57 (37.52 to 37.63)	3977	79247	50.15 (50.08 to 50.21)	867	37121.07	18.43 (18.38 to 18.48)	1663	41881	30.37 (30.31 to 30.43)
	IMD=8	2146	53762.05	40.32 (40.26 to 40.38)	3464	63754	54.46 (54.39 to 54.53)	746	29291.74	20.20 (20.16 to 20.25)	1423	33377	32.52 (32.46 to 32.59)
	IMD=9	2927	72088.25	41.32 (41.26 to 41.38)	4836	85638	56.86 (56.79 to 56.93)	968	38793.31	19.65 (19.60 to 19.70)	1881	44058	32.58 (32.52 to 32.64)
	Most Deprivation	4502	105227.8	44.37 (44.31 to 44.43)	7598	125967	61.68 (61.60 to 61.75)	1328	54576.8	19.64 (19.59 to 19.69)	2609	61968	33.11 (33.04 to 33.17)
2006	Least Deprivation	2170	66113.44	31.44 (31.39 to 31.49)	3433	76003	42.51 (42.45 to 42.56)	779	43661.99	14.59 (14.55 to 14.63)	1397	47848	23.21 (23.16 to 23.27)
	IMD=2	2254	67594.09	32.66 (32.61 to 32.71)	3737	78724	45.71 (45.65 to 45.77)	807	42335.72	15.39 (15.35 to 15.43)	1654	47135	27.38 (27.32 to 27.44)
	IMD=3	2758	79036.51	34.10 (34.05 to 34.15)	4493	92135	47.05 (46.99 to 47.12)	1048	49933.03	16.82 (16.78 to 16.87)	1990	55722	27.46 (27.40 to 27.52)
	IMD=4	1956	58218.23	33.05 (32.99 to 33.10)	3138	67867	44.89 (44.83 to 44.95)	756	35663.42	17.04 (17.00 to 17.09)	1474	39824	28.82 (28.76 to 28.88)
	IMD=5	1995	57704.12	34.19 (34.14 to 34.24)	3287	67430	47.65 (47.59 to 47.71)	680	34395.19	16.05 (16.00 to 16.09)	1350	38445	27.51 (27.46 to 27.57)
	IMD=6	2200	60113.63	36.18 (36.12 to 36.23)	3617	70953	49.79 (49.72 to 49.85)	823	35541.95	18.22 (18.17 to 18.27)	1515	40064	28.73 (28.67 to 28.79)
	IMD=7	2592	68453.98	38.25 (38.20 to 38.31)	4301	81507	52.62 (52.55 to 52.68)	873	38110.39	18.23 (18.19 to 18.28)	1750	43176	31.18 (31.12 to 31.24)
	IMD=8	2113	54614.43	39.11 (39.05 to 39.17)	3539	65342	54.21 (54.15 to 54.28)	686	29969.81	18.24 (18.19 to 18.29)	1412	34177	31.71 (31.65 to 31.77)
	IMD=9	3010	72832.32	42.24 (42.18 to 42.30)	5192	87440	59.91 (59.84 to 59.98)	968	39421.03	19.49 (19.44 to 19.54)	1932	44935	33.02 (32.96 to 33.09)
	Most Deprivation	4581	106680.7	44.55 (44.49 to 44.61)	8013	129250	63.41 (63.34 to 63.49)	1270	55644.99	18.57 (18.52 to 18.62)	2663	63306	33.17 (33.11 to 33.23)
2007	Least Deprivation	2107	66473.88	30.35 (30.30 to 30.40)	3456	77097	42.15 (42.09 to 42.21)	703	44511.01	12.95 (12.91 to 12.99)	1405	48853	22.82 (22.77 to 22.87)
	IMD=2	2424	68042.13	34.65 (34.59 to 34.70)	4037	79961	48.36 (48.30 to 48.43)	797	43213.15	14.94 (14.90 to 14.98)	1621	48166	26.28 (26.23 to 26.34)
	IMD=3	2864	79477.74	34.89 (34.84 to 34.95)	4718	93611	48.08 (48.01 to 48.14)	1042	50942.14	16.40 (16.36 to 16.45)	2039	56976	27.60 (27.54 to 27.66)
	IMD=4	2112	59208.76	34.85 (34.79 to 34.90)	3375	69544	46.89 (46.82 to 46.95)	766	36645.2	16.71 (16.67 to 16.76)	1528	41059	28.89 (28.84 to 28.95)
	IMD=5	2135	58002.49	36.33 (36.28 to 36.39)	3517	68455	50.19 (50.13 to 50.26)	683	35000.74	15.85 (15.81 to 15.90)	1431	39262	28.58 (28.53 to 28.64)

	IMD=6	2348	60720.21	38.41 (38.35 to 38.47)	3841	72213	52.16 (52.10 to 52.23)	857	36199.64	18.84 (18.79 to 18.89)	1683	40984	31.35 (31.29 to 31.41)
	IMD=7	2670	69090.31	39.04 (38.98 to 39.09)	4532	83034	54.57 (54.51 to 54.64)	875	38973.67	18.12 (18.08 to 18.17)	1814	44159	31.80 (31.74 to 31.86)
	IMD=8	2243	54986.72	41.35 (41.29 to 41.41)	3809	66439	57.40 (57.33 to 57.47)	691	30541.3	18.05 (18.00 to 18.10)	1440	34851	31.73 (31.67 to 31.79)
	IMD=9	2990	72682.02	41.95 (41.89 to 42.01)	5348	88703	60.81 (60.74 to 60.88)	938	39872.04	18.90 (18.86 to 18.95)	1975	45603	33.56 (33.49 to 33.62)
	Most Deprivation	4776	107223	46.34 (46.28 to 46.40)	8677	131382	67.66 (67.59 to 67.74)	1268	56334.07	18.57 (18.52 to 18.62)	2668	64355	33.05 (32.99 to 33.11)
2008	Least Deprivation	2135	67949.48	30.20 (30.15 to 30.25)	3508	78581	42.10 (42.05 to 42.16)	770	46025.64	13.55 (13.51 to 13.59)	1435	50323	22.41 (22.36 to 22.46)
	IMD=2	2381	69614.93	33.03 (32.98 to 33.08)	4005	81637	46.74 (46.68 to 46.81)	837	44859.14	15.04 (14.99 to 15.08)	1663	49702	26.11 (26.06 to 26.17)
	IMD=3	2887	81570.3	34.44 (34.38 to 34.49)	4793	95760	47.83 (47.77 to 47.89)	1028	52887.45	15.61 (15.57 to 15.65)	2018	58884	26.41 (26.35 to 26.46)
	IMD=4	2023	60308.89	32.67 (32.62 to 32.72)	3386	70650	46.23 (46.17 to 46.29)	711	37727.02	15.24 (15.20 to 15.28)	1476	42074	27.33 (27.27 to 27.39)
	IMD=5	2108	59169.55	35.41 (35.36 to 35.47)	3611	69693	50.63 (50.56 to 50.69)	758	36115.37	17.10 (17.05 to 17.14)	1512	40338	29.53 (29.47 to 29.59)
	IMD=6	2362	61866.72	37.84 (37.78 to 37.89)	4067	73590	54.05 (53.98 to 54.12)	888	37177.83	19.03 (18.98 to 19.08)	1742	42043	31.80 (31.74 to 31.86)
	IMD=7	2778	71787.32	39.04 (38.99 to 39.10)	4758	85859	55.33 (55.26 to 55.39)	928	40635.87	18.36 (18.32 to 18.41)	1941	45882	32.86 (32.80 to 32.93)
	IMD=8	2185	56077.64	39.51 (39.45 to 39.57)	3812	67731	56.31 (56.24 to 56.37)	700	31314.39	18.03 (17.99 to 18.08)	1497	35653	32.55 (32.48 to 32.61)
	IMD=9	3126	73307.91	43.60 (43.54 to 43.66)	5661	89469	63.80 (63.73 to 63.88)	918	40403.73	18.24 (18.19 to 18.29)	2025	46217	34.26 (34.19 to 34.32)
	Most Deprivation	4702	108956.3	44.86 (44.80 to 44.92)	8811	134053	67.33 (67.26 to 67.41)	1257	57785.33	17.95 (17.90 to 17.99)	2849	65834	34.71 (34.64 to 34.77)
2009	Least Deprivation	2186	68459.29	30.72 (30.66 to 30.77)	3622	79588	43.09 (43.03 to 43.15)	794	46917.16	13.64 (13.60 to 13.68)	1471	51420	22.39 (22.34 to 22.45)
	IMD=2	2309	70116.05	32.09 (32.04 to 32.14)	3984	82356	46.31 (46.25 to 46.37)	861	45758.29	15.14 (15.10 to 15.19)	1725	50697	26.54 (26.49 to 26.60)
	IMD=3	2982	81888.29	35.22 (35.16 to 35.27)	4993	96674	49.33 (49.27 to 49.39)	1096	53679.65	16.35 (16.31 to 16.40)	2115	59769	27.30 (27.25 to 27.36)
	IMD=4	2085	60849.24	33.64 (33.59 to 33.69)	3444	71567	46.45 (46.39 to 46.51)	747	38553.78	15.66 (15.61 to 15.70)	1475	43055	26.71 (26.66 to 26.77)
	IMD=5	2119	59443.44	35.45 (35.40 to 35.51)	3655	70574	50.70 (50.63 to 50.76)	689	36795.9	15.35 (15.31 to 15.39)	1443	41138	27.73 (27.67 to 27.79)
	IMD=6	2418	62274.48	38.37 (38.31 to 38.43)	4191	74627	54.79 (54.72 to 54.85)	864	37803.43	18.28 (18.24 to 18.33)	1697	42869	30.39 (30.33 to 30.45)
	IMD=7	2787	72505.34	38.92 (38.86 to 38.97)	4964	87437	56.87 (56.80 to 56.94)	890	41248.6	17.48 (17.43 to 17.53)	1911	46701	31.90 (31.83 to 31.96)
	IMD=8	2320	56717.84	41.51 (41.45 to 41.57)	4089	68851	59.56 (59.49 to 59.64)	753	31769.42	19.31 (19.26 to 19.36)	1553	36187	33.52 (33.46 to 33.58)
	IMD=9	3189	73578.98	44.64 (44.57 to 44.70)	5882	90390	65.84 (65.77 to 65.92)	930	40827.63	18.55 (18.50 to 18.60)	2070	46588	34.99 (34.92 to 35.05)
	Most Deprivation	4893	109374.3	46.45 (46.39 to 46.51)	9252	135737	69.78 (69.71 to 69.86)	1265	58487.11	17.90 (17.86 to 17.95)	2865	66700	34.70 (34.64 to 34.77)
2010	Least Deprivation	2320	69470.2	32.01 (31.96 to 32.07)	3765	80923	43.70 (43.64 to 43.76)	797	48070.89	13.41 (13.37 to 13.45)	1449	52757	21.50 (21.45 to 21.55)
	IMD=2	2583	71098.24	35.21 (35.16 to 35.27)	4314	83742	49.10 (49.04 to 49.16)	913	46858.08	15.78 (15.74 to 15.83)	1776	51938	26.73 (26.67 to 26.79)
	IMD=3	3006	82501.92	35.24 (35.18 to 35.29)	5165	97863	49.98 (49.92 to 50.05)	1169	54604.92	17.12 (17.08 to 17.17)	2192	60861	27.83 (27.77 to 27.89)
	IMD=4	2163	61435.63	34.44 (34.39 to 34.49)	3613	72462	47.97 (47.91 to 48.03)	774	39442.19	15.90 (15.85 to 15.94)	1468	43948	26.07 (26.01 to 26.12)

	IMD=5	2096	60228.43	34.42 (34.36 to 34.47)	3679	71508	50.08 (50.02 to 50.15)	824	37492.31	18.07 (18.02 to 18.12)	1566	41978	29.69 (29.63 to 29.75)
	IMD=6	2450	62834.92	38.66 (38.60 to 38.72)	4289	75560	55.70 (55.63 to 55.77)	839	38484.44	17.60 (17.55 to 17.65)	1763	43636	31.36 (31.30 to 31.42)
	IMD=7	2813	73578.95	38.67 (38.61 to 38.72)	5084	88819	57.09 (57.02 to 57.16)	969	41970.18	18.87 (18.82 to 18.92)	2064	47577	34.08 (34.02 to 34.15)
	IMD=8	2378	57445.12	42.14 (42.08 to 42.20)	4237	69861	60.68 (60.61 to 60.75)	723	32251.22	18.36 (18.31 to 18.40)	1574	36749	33.70 (33.63 to 33.76)
	IMD=9	3319	74126.69	46.10 (46.04 to 46.16)	6114	91460	67.65 (67.58 to 67.73)	980	41190.38	19.45 (19.40 to 19.50)	2206	47082	37.01 (36.95 to 37.08)
	Most Deprivation	5138	109871.1	48.76 (48.70 to 48.83)	9807	137071	73.28 (73.20 to 73.35)	1266	59336.61	17.77 (17.73 to 17.82)	2868	67608	34.54 (34.48 to 34.61)
2011	Least Deprivation	2421	70005.42	32.90 (32.84 to 32.95)	3960	81728	45.32 (45.26 to 45.38)	808	48843.53	13.24 (13.20 to 13.28)	1447	53586	20.98 (20.93 to 21.03)
	IMD=2	2606	71690.54	35.36 (35.31 to 35.42)	4474	84647	50.50 (50.43 to 50.56)	856	47778.64	14.40 (14.36 to 14.44)	1725	52988	25.36 (25.31 to 25.42)
	IMD=3	3081	83215.61	36.21 (36.16 to 36.27)	5249	98884	50.75 (50.68 to 50.81)	1110	55484.16	16.05 (16.00 to 16.09)	2197	61922	27.36 (27.31 to 27.42)
	IMD=4	2199	62056.06	34.59 (34.54 to 34.65)	3637	73327	47.61 (47.55 to 47.68)	783	40443.82	15.62 (15.58 to 15.67)	1519	45067	26.25 (26.19 to 26.30)
	IMD=5	2168	60780.52	35.28 (35.23 to 35.34)	3796	72605	50.83 (50.77 to 50.90)	651	38118.46	13.98 (13.94 to 14.02)	1377	42827	25.53 (25.48 to 25.59)
	IMD=6	2549	63313.35	39.88 (39.82 to 39.94)	4500	76386	57.56 (57.49 to 57.63)	838	39152.95	17.21 (17.16 to 17.25)	1788	44316	31.24 (31.18 to 31.30)
	IMD=7	2930	74357.93	39.77 (39.71 to 39.83)	5284	90494	58.22 (58.15 to 58.29)	943	42620.37	18.07 (18.03 to 18.12)	1998	48482	32.38 (32.31 to 32.44)
	IMD=8	2371	57965.67	41.82 (41.76 to 41.87)	4300	71089	60.80 (60.73 to 60.87)	731	32796.42	18.28 (18.23 to 18.33)	1582	37484	33.36 (33.30 to 33.43)
	IMD=9	3169	74665.16	43.71 (43.65 to 43.77)	6062	92570	66.37 (66.30 to 66.45)	933	41795.54	18.53 (18.48 to 18.57)	2114	47796	35.25 (35.19 to 35.32)
	Most Deprivation	5091	110500.2	48.06 (47.99 to 48.12)	10079	138583	74.52 (74.44 to 74.60)	1289	59971.6	18.16 (18.11 to 18.20)	3016	68544	35.92 (35.86 to 35.99)
2012	Least Deprivation	2320	70096.67	31.51 (31.46 to 31.57)	3824	82115	43.47 (43.41 to 43.53)	817	49534.75	13.13 (13.09 to 13.17)	1431	54311	20.34 (20.29 to 20.39)
	IMD=2	2613	71970.17	35.11 (35.05 to 35.16)	4492	85168	50.34 (50.27 to 50.40)	891	48519.66	14.75 (14.71 to 14.79)	1743	53732	25.13 (25.07 to 25.18)
	IMD=3	3064	83401.07	35.47 (35.41 to 35.52)	5295	99296	50.76 (50.69 to 50.82)	1053	56271.37	14.91 (14.87 to 14.95)	2095	62702	25.63 (25.57 to 25.68)
	IMD=4	2089	62671.27	32.58 (32.52 to 32.63)	3539	73856	46.11 (46.04 to 46.17)	736	41215.93	14.29 (14.25 to 14.33)	1454	45733	24.60 (24.55 to 24.65)
	IMD=5	2177	61220.91	35.06 (35.01 to 35.12)	3752	72756	50.20 (50.14 to 50.27)	762	38830.22	16.01 (15.97 to 16.06)	1408	43219	25.79 (25.74 to 25.85)
	IMD=6	2500	63749.81	38.86 (38.81 to 38.92)	4487	77019	56.84 (56.78 to 56.91)	860	39903.09	17.37 (17.33 to 17.42)	1771	45078	30.44 (30.37 to 30.50)
	IMD=7	3040	74992.28	40.98 (40.93 to 41.04)	5433	90906	59.58 (59.51 to 59.65)	916	43498.95	17.25 (17.20 to 17.29)	2013	49181	32.19 (32.13 to 32.25)
	IMD=8	2449	58344.6	42.75 (42.69 to 42.81)	4446	71472	62.42 (62.35 to 62.49)	690	33331.02	16.93 (16.88 to 16.97)	1521	37895	31.57 (31.51 to 31.63)
	IMD=9	3343	75227.36	45.62 (45.55 to 45.68)	6367	93004	69.31 (69.23 to 69.38)	950	42407.5	18.55 (18.50 to 18.60)	2136	48315	35.31 (35.24 to 35.37)
	Most Deprivation	5220	111151.2	49.14 (49.07 to 49.20)	10436	139357	76.66 (76.58 to 76.74)	1199	60865.84	16.55 (16.51 to 16.60)	2860	69106	33.94 (33.88 to 34.01)
2013	Least Deprivation	2387	69722.18	32.40 (32.35 to 32.45)	3786	82166	42.87 (42.81 to 42.93)	886	49877.74	14.02 (13.98 to 14.07)	1490	54897	20.82 (20.77 to 20.87)
	IMD=2	2646	71504.86	35.85 (35.80 to 35.91)	4436	85695	49.43 (49.37 to 49.50)	956	48808.23	15.68 (15.64 to 15.73)	1793	54558	25.36 (25.30 to 25.41)
	IMD=3	3024	82818.32	35.52 (35.46 to 35.57)	5256	99640	50.12 (50.06 to 50.19)	1197	56515.71	16.88 (16.83 to 16.92)	2238	63450	26.96 (26.91 to 27.02)

	IMD=4	2261	61735.44	35.83 (35.78 to 35.89)	3669	74304	47.46 (47.39 to 47.52)	834	41200.69	16.16 (16.12 to 16.21)	1562	46331	25.99 (25.94 to 26.05)
	IMD=5	2242	60558.8	36.48 (36.42 to 36.53)	3793	73062	50.53 (50.47 to 50.60)	817	38907.43	17.15 (17.11 to 17.20)	1493	43844	26.91 (26.85 to 26.97)
	IMD=6	2553	63549.51	39.91 (39.85 to 39.97)	4516	77406	57.01 (56.94 to 57.08)	887	40027.41	17.81 (17.77 to 17.86)	1750	45460	29.89 (29.83 to 29.95)
	IMD=7	2951	74675.79	40.05 (39.99 to 40.11)	5312	91598	57.95 (57.88 to 58.02)	960	43727.1	18.07 (18.03 to 18.12)	2035	49846	32.19 (32.13 to 32.25)
	IMD=8	2369	58247.57	41.51 (41.45 to 41.56)	4434	72160	61.70 (61.62 to 61.77)	825	33599.12	20.18 (20.13 to 20.23)	1644	38460	33.83 (33.77 to 33.90)
	IMD=9	3410	75143.22	46.84 (46.78 to 46.91)	6577	94395	70.69 (70.61 to 70.76)	931	42523.79	18.17 (18.12 to 18.21)	2146	49017	35.03 (34.96 to 35.09)
	Most Deprivation	5355	109745	50.85 (50.79 to 50.92)	10905	140950	79.22 (79.14 to 79.30)	1336	60627.52	18.72 (18.67 to 18.76)	2950	69938	34.74 (34.67 to 34.80)
2014	Least Deprivation	2372	69887.09	32.08 (32.03 to 32.13)	3762	82184	42.54 (42.48 to 42.60)	967	50305.62	15.08 (15.03 to 15.12)	1588	55273	21.87 (21.82 to 21.92)
	IMD=2	2544	71788.86	34.16 (34.11 to 34.22)	4196	85534	46.53 (46.47 to 46.59)	1045	49375.86	16.84 (16.79 to 16.88)	1819	54869	25.55 (25.50 to 25.61)
	IMD=3	2964	83378.25	34.14 (34.09 to 34.19)	5105	99560	48.51 (48.44 to 48.57)	1213	57277.77	16.75 (16.70 to 16.79)	2259	64005	26.93 (26.87 to 26.98)
	IMD=4	2230	61401.52	35.41 (35.35 to 35.46)	3701	72952	48.73 (48.67 to 48.80)	948	41349.61	18.21 (18.16 to 18.25)	1634	46205	27.21 (27.15 to 27.27)
	IMD=5	2235	60134.79	36.54 (36.49 to 36.60)	3813	72013	51.40 (51.33 to 51.46)	936	39175.49	19.55 (19.50 to 19.60)	1575	43805	28.42 (28.36 to 28.48)
	IMD=6	2609	63498.84	40.75 (40.70 to 40.81)	4564	77318	57.52 (57.45 to 57.59)	884	40399.77	17.57 (17.52 to 17.61)	1778	45766	30.21 (30.15 to 30.27)
	IMD=7	2971	74452.78	40.43 (40.38 to 40.49)	5312	90991	58.17 (58.10 to 58.24)	1031	44018.94	19.29 (19.24 to 19.34)	2091	50013	33.03 (32.97 to 33.09)
	IMD=8	2478	58593.76	43.08 (43.02 to 43.14)	4537	72023	63.12 (63.05 to 63.19)	830	33798.92	20.24 (20.19 to 20.28)	1629	38487	33.55 (33.49 to 33.61)
	IMD=9	3177	75001.67	43.71 (43.65 to 43.77)	6075	93626	65.72 (65.64 to 65.79)	1008	42740.63	19.73 (19.69 to 19.78)	2103	48823	34.54 (34.48 to 34.61)
	Most Deprivation	5197	109283.2	49.70 (49.64 to 49.77)	10416	138842	76.62 (76.54 to 76.70)	1518	60656.42	21.24 (21.19 to 21.29)	3070	69264	36.53 (36.46 to 36.59)
2015	Least Deprivation	2275	70520.12	30.74 (30.69 to 30.79)	3562	82561	40.35 (40.29 to 40.41)	1109	50692.85	17.19 (17.15 to 17.24)	1755	55855	23.85 (23.80 to 23.90)
	IMD=2	2583	72096.89	34.38 (34.33 to 34.44)	4257	85694	47.09 (47.03 to 47.15)	1105	49707.98	17.74 (17.70 to 17.79)	1956	55375	27.07 (27.01 to 27.13)
	IMD=3	2850	84053.14	32.67 (32.61 to 32.72)	4799	99975	45.56 (45.50 to 45.62)	1303	57658.07	17.85 (17.81 to 17.90)	2305	64487	27.15 (27.09 to 27.21)
	IMD=4	2169	61685.83	33.96 (33.91 to 34.01)	3610	73182	47.05 (46.99 to 47.12)	972	41609.06	18.62 (18.57 to 18.66)	1752	46641	28.82 (28.76 to 28.88)
	IMD=5	2148	60712.69	34.85 (34.79 to 34.90)	3641	72472	48.77 (48.71 to 48.84)	908	39608.86	18.71 (18.67 to 18.76)	1627	44426	28.90 (28.84 to 28.96)
	IMD=6	2444	63898.71	37.82 (37.76 to 37.88)	4292	77633	53.75 (53.68 to 53.81)	1008	40702.29	19.96 (19.91 to 20.01)	1914	46138	32.23 (32.17 to 32.29)
	IMD=7	2973	75095.3	40.10 (40.04 to 40.16)	5332	91635	58.02 (57.95 to 58.09)	1077	44392.15	19.86 (19.81 to 19.91)	2161	50512	33.79 (33.72 to 33.85)
	IMD=8	2375	58999.67	40.96 (40.90 to 41.02)	4454	72651	61.35 (61.28 to 61.42)	783	34180.22	19.01 (18.97 to 19.06)	1631	38998	33.27 (33.21 to 33.34)
	IMD=9	3307	75640.53	45.11 (45.05 to 45.17)	6184	94250	66.46 (66.38 to 66.53)	1066	43064.3	20.62 (20.57 to 20.67)	2230	49284	36.48 (36.41 to 36.54)
	Most Deprivation	5226	110253.7	49.61 (49.55 to 49.68)	10430	140246	75.85 (75.77 to 75.93)	1492	61169.86	20.78 (20.73 to 20.83)	3123	70254	36.65 (36.58 to 36.71)
2016	Least Deprivation	2345	71288.45	31.37 (31.32 to 31.42)	3656	83001	41.25 (41.19 to 41.31)	1251	50974.34	19.29 (19.24 to 19.34)	2010	56433	26.94 (26.89 to 27.00)
	IMD=2	2648	72699.01	34.71 (34.65 to 34.76)	4246	86036	46.28 (46.22 to 46.34)	1301	50097.67	20.59 (20.54 to 20.64)	2176	55932	29.67 (29.61 to 29.73)

	IMD=3	3018	84884.32	34.14 (34.08 to 34.19)	5028	100629	47.25 (47.19 to 47.31)	1413	58132.59	19.15 (19.10 to 19.20)	2510	65177	29.27 (29.21 to 29.33)
	IMD=4	2142	62436.57	33.45 (33.39 to 33.50)	3526	73765	45.84 (45.78 to 45.90)	991	42080.89	18.76 (18.71 to 18.81)	1795	47197	29.23 (29.17 to 29.28)
	IMD=5	2213	61579.31	35.14 (35.09 to 35.20)	3668	73110	48.44 (48.38 to 48.51)	962	40143.92	19.51 (19.46 to 19.56)	1692	45043	29.51 (29.45 to 29.57)
	IMD=6	2487	64637.81	38.03 (37.97 to 38.08)	4246	78072	52.97 (52.90 to 53.03)	1049	41132.41	20.51 (20.46 to 20.56)	1921	46620	32.06 (32.00 to 32.12)
	IMD=7	2889	76424.94	38.51 (38.46 to 38.57)	5103	92640	55.10 (55.03 to 55.17)	1106	45049.44	20.21 (20.16 to 20.26)	2194	51141	33.97 (33.90 to 34.03)
	IMD=8	2237	59800.39	38.14 (38.08 to 38.19)	4190	73068	57.40 (57.33 to 57.47)	839	34545.11	20.09 (20.05 to 20.14)	1647	39307	33.33 (33.26 to 33.39)
	IMD=9	3257	76489.14	43.89 (43.83 to 43.95)	6154	94846	65.69 (65.62 to 65.77)	1129	43450.68	21.76 (21.71 to 21.81)	2291	49610	37.30 (37.23 to 37.37)
	Most Deprivation	5090	112174.9	47.50 (47.43 to 47.56)	10130	141604	72.85 (72.77 to 72.93)	1638	61938.8	22.47 (22.42 to 22.53)	3285	70895	38.08 (38.01 to 38.15)
2017	Least Deprivation	2514	72145.92	32.76 (32.71 to 32.81)	3834	84013	42.20 (42.14 to 42.26)	1278	51256.81	19.43 (19.38 to 19.47)	2197	57244	28.89 (28.83 to 28.94)
	IMD=2	2526	73589.47	33.08 (33.02 to 33.13)	4102	86904	44.56 (44.50 to 44.62)	1292	50306.98	20.29 (20.24 to 20.34)	2267	56626	30.48 (30.42 to 30.54)
	IMD=3	3000	85829.88	33.42 (33.36 to 33.47)	4859	101371	45.10 (45.04 to 45.16)	1522	58269.69	20.48 (20.43 to 20.53)	2692	65695	31.01 (30.95 to 31.07)
	IMD=4	2186	63332.21	33.19 (33.14 to 33.25)	3487	74770	44.30 (44.24 to 44.36)	1033	42531.62	19.24 (19.19 to 19.29)	1895	47914	30.18 (30.12 to 30.24)
	IMD=5	2246	62381.88	35.19 (35.14 to 35.25)	3667	74144	47.69 (47.62 to 47.75)	1025	40727.88	20.36 (20.31 to 20.41)	1757	45963	29.92 (29.86 to 29.98)
	IMD=6	2497	65671.92	37.57 (37.52 to 37.63)	4243	79104	52.15 (52.08 to 52.21)	1132	41536.29	21.98 (21.93 to 22.04)	2037	47285	33.50 (33.44 to 33.57)
	IMD=7	2762	77917.03	36.06 (36.00 to 36.11)	4932	94150	52.30 (52.24 to 52.37)	1119	45545.01	20.20 (20.15 to 20.25)	2242	51897	34.20 (34.13 to 34.26)
	IMD=8	2354	61025.72	39.46 (39.40 to 39.52)	4149	74167	56.05 (55.98 to 56.12)	889	34861.06	21.10 (21.05 to 21.15)	1744	39745	35.00 (34.93 to 35.06)
	IMD=9	3202	78010.13	42.51 (42.45 to 42.56)	6103	96216	64.14 (64.07 to 64.21)	1113	43760.57	21.23 (21.18 to 21.28)	2263	50188	36.40 (36.33 to 36.47)
	Most Deprivation	5164	114627.3	47.26 (47.19 to 47.32)	10127	144209	71.59 (71.51 to 71.67)	1652	62601.41	22.85 (22.79 to 22.90)	3392	72018	39.05 (38.98 to 39.12)
2018	Least Deprivation	2282	73049.11	29.33 (29.28 to 29.38)	3511	84845	37.96 (37.90 to 38.02)	1281	51436.57	19.25 (19.20 to 19.30)	2191	57793	28.30 (28.24 to 28.36)
	IMD=2	2422	74917.4	30.76 (30.71 to 30.81)	3890	88232	41.40 (41.34 to 41.46)	1302	50790.17	20.17 (20.12 to 20.22)	2287	57469	30.04 (29.98 to 30.10)
	IMD=3	2895	87823	31.66 (31.61 to 31.71)	4707	103404	42.92 (42.86 to 42.98)	1500	59182.58	19.81 (19.76 to 19.86)	2715	66980	30.47 (30.41 to 30.53)
	IMD=4	2217	64908.07	32.59 (32.54 to 32.64)	3553	76331	43.86 (43.80 to 43.92)	1101	43341.64	19.99 (19.94 to 20.04)	1954	48976	30.22 (30.16 to 30.28)
	IMD=5	2188	63930.85	33.61 (33.55 to 33.66)	3561	75766	45.51 (45.45 to 45.58)	1002	41662.51	19.32 (19.28 to 19.37)	1762	47067	29.06 (29.00 to 29.12)
	IMD=6	2352	67618.84	34.30 (34.24 to 34.35)	4098	80997	49.16 (49.10 to 49.23)	1066	42422.42	20.13 (20.08 to 20.18)	2008	48467	32.08 (32.02 to 32.15)
	IMD=7	2813	81204.06	35.41 (35.35 to 35.46)	4890	97722	50.19 (50.12 to 50.25)	1175	46603.6	20.63 (20.58 to 20.68)	2306	53244	34.19 (34.13 to 34.26)
	IMD=8	2330	63011.29	37.98 (37.92 to 38.03)	4160	76390	54.83 (54.76 to 54.90)	948	35425.18	22.18 (22.13 to 22.23)	1831	40557	35.97 (35.90 to 36.03)
	IMD=9	3185	80210.08	41.20 (41.14 to 41.26)	5904	98662	60.71 (60.63 to 60.78)	1119	44317.03	21.11 (21.06 to 21.16)	2283	50970	36.08 (36.02 to 36.15)
	Most Deprivation	5048	118319.9	44.84 (44.78 to 44.91)	9793	148093	67.43 (67.36 to 67.51)	1727	63791.62	23.10 (23.05 to 23.16)	3511	73615	39.27 (39.20 to 39.34)
2019	Least Deprivation	2288	72577.28	29.49 (29.44 to 29.54)	3505	86158	37.42 (37.36 to 37.48)	1342	50705.72	20.31 (20.26 to 20.36)	2294	58603	28.84 (28.78 to 28.90)

	IMD=2	2419	75162.63	30.63 (30.58 to 30.68)	3919	89585	40.78 (40.72 to 40.84)	1387	50370.89	21.48 (21.43 to 21.53)	2427	58249	31.24 (31.18 to 31.30)
	IMD=3	2799	87660.36	30.50 (30.45 to 30.55)	4424	104652	39.73 (39.68 to 39.79)	1551	58577.94	20.53 (20.48 to 20.58)	2859	67735	31.55 (31.48 to 31.61)
	IMD=4	2158	65722.63	31.47 (31.42 to 31.52)	3442	77354	41.98 (41.92 to 42.04)	1178	43672.04	20.95 (20.90 to 21.00)	2098	49652	31.72 (31.66 to 31.78)
	IMD=5	2115	64600.8	31.87 (31.82 to 31.92)	3520	76562	44.32 (44.26 to 44.38)	1102	41882.81	20.89 (20.84 to 20.94)	1932	47533	31.18 (31.12 to 31.24)
	IMD=6	2471	68390.39	35.48 (35.42 to 35.53)	4071	82240	47.98 (47.91 to 48.04)	1095	42719.7	20.40 (20.35 to 20.45)	2080	49016	32.72 (32.66 to 32.78)
	IMD=7	2860	83462.71	35.12 (35.06 to 35.17)	4933	99881	49.78 (49.72 to 49.85)	1213	47076.36	21.06 (21.01 to 21.11)	2381	53838	34.84 (34.77 to 34.90)
	IMD=8	2311	64535.42	36.86 (36.80 to 36.91)	3995	77955	51.72 (51.65 to 51.79)	973	35665.46	22.53 (22.48 to 22.58)	1855	40975	35.94 (35.88 to 36.01)
	IMD=9	3048	82365.19	38.55 (38.49 to 38.60)	5585	100841	56.44 (56.37 to 56.51)	1244	44771.55	23.17 (23.12 to 23.22)	2424	51600	37.80 (37.73 to 37.86)
	Most Deprivation	5111	121880.6	44.28 (44.22 to 44.34)	9576	151561	64.66 (64.58 to 64.73)	1715	64475.26	22.64 (22.59 to 22.69)	3661	74721	40.18 (40.11 to 40.25)
**Yorkshire and the Humber**													
2004	Least Deprivation	453	12213.35	35.73 (35.68 to 35.78)	667	14064	45.17 (45.11 to 45.23)	203	7873.752	21.23 (21.18 to 21.28)	376	8772	34.12 (34.05 to 34.18)
	IMD=2	522	19310.8	26.57 (26.52 to 26.61)	814	21880	36.12 (36.06 to 36.17)	271	11834.07	18.03 (17.99 to 18.08)	535	13174	31.11 (31.05 to 31.17)
	IMD=3	772	20705.34	36.52 (36.46 to 36.57)	1196	24291	47.73 (47.66 to 47.79)	337	12750.42	21.48 (21.42 to 21.53)	683	14483	37.14 (37.07 to 37.21)
	IMD=4	467	13197.53	34.91 (34.86 to 34.96)	706	15305	44.89 (44.83 to 44.95)	180	7892.827	18.27 (18.22 to 18.31)	347	8885	30.72 (30.66 to 30.78)
	IMD=5	616	18180.63	33.26 (33.21 to 33.31)	961	20893	44.52 (44.46 to 44.58)	279	11164.74	19.33 (19.28 to 19.38)	625	12695	36.91 (36.84 to 36.98)
	IMD=6	460	13078.56	34.85 (34.80 to 34.90)	683	15058	44.51 (44.45 to 44.57)	201	7857.897	20.18 (20.13 to 20.23)	408	8915	34.80 (34.74 to 34.87)
	IMD=7	355	10115.32	34.84 (34.79 to 34.90)	551	11769	46.60 (46.54 to 46.67)	139	5453.796	20.00 (19.95 to 20.05)	288	6196	34.53 (34.46 to 34.59)
	IMD=8	439	11050.47	39.79 (39.74 to 39.85)	655	12706	50.89 (50.83 to 50.96)	184	6009.952	24.40 (24.35 to 24.46)	361	6880	40.18 (40.11 to 40.25)
	IMD=9	441	10898.2	41.45 (41.39 to 41.51)	662	12586	53.19 (53.12 to 53.26)	168	5655.579	23.42 (23.37 to 23.47)	303	6359	36.66 (36.59 to 36.73)
	Most Deprivation	772	19260.69	40.84 (40.79 to 40.90)	1152	22064	52.57 (52.50 to 52.64)	279	10178.12	21.36 (21.31 to 21.41)	470	11362	31.31 (31.25 to 31.37)
2005	Least Deprivation	421	12138.32	33.73 (33.68 to 33.78)	663	14253	44.27 (44.21 to 44.33)	184	7949.944	19.11 (19.06 to 19.15)	374	8932	33.18 (33.12 to 33.25)
	IMD=2	671	19278.38	33.96 (33.90 to 34.01)	993	22323	42.87 (42.81 to 42.93)	285	11872.9	19.32 (19.27 to 19.36)	595	13354	34.15 (34.08 to 34.21)
	IMD=3	788	20963.76	37.09 (37.04 to 37.15)	1240	24918	48.32 (48.25 to 48.38)	321	12964.04	20.51 (20.46 to 20.56)	677	14781	36.08 (36.02 to 36.15)
	IMD=4	482	13262.32	35.18 (35.12 to 35.23)	764	15638	47.29 (47.23 to 47.36)	190	7972.172	19.37 (19.32 to 19.41)	383	9010	33.42 (33.35 to 33.48)
	IMD=5	679	18337.87	36.19 (36.13 to 36.24)	1043	21398	47.28 (47.22 to 47.35)	326	11318.79	22.64 (22.58 to 22.69)	681	12996	39.35 (39.28 to 39.41)
	IMD=6	490	13139.03	37.28 (37.22 to 37.33)	770	15418	49.27 (49.21 to 49.34)	214	7927.587	21.58 (21.53 to 21.63)	471	9110	39.34 (39.27 to 39.41)
	IMD=7	390	10616.85	37.79 (37.74 to 37.85)	603	12568	49.13 (49.07 to 49.19)	144	5774.981	19.62 (19.57 to 19.66)	309	6584	35.50 (35.43 to 35.56)
	IMD=8	420	11467.35	36.53 (36.48 to 36.59)	668	13464	49.23 (49.17 to 49.29)	147	6235.25	18.98 (18.93 to 19.03)	353	7207	37.73 (37.67 to 37.80)
	IMD=9	456	11422.81	39.83 (39.77 to 39.89)	746	13464	55.61 (55.54 to 55.68)	149	5940.033	19.96 (19.91 to 20.00)	327	6784	37.08 (37.02 to 37.15)

	Most Deprivation	833	19757.69	43.44 (43.38 to 43.50)	1307	23219	57.41 (57.34 to 57.48)	238	10402.46	18.24 (18.19 to 18.29)	484	11768	31.55 (31.49 to 31.61)
2006	Least Deprivation	431	12268.13	33.97 (33.91 to 34.02)	691	14430	46.02 (45.95 to 46.08)	196	8042.94	20.25 (20.20 to 20.30)	395	9098	34.21 (34.15 to 34.28)
	IMD=2	684	19630.48	34.33 (34.28 to 34.39)	1039	22847	44.08 (44.02 to 44.14)	245	12172.07	16.12 (16.07 to 16.16)	583	13716	32.53 (32.46 to 32.59)
	IMD=3	820	21319.19	37.70 (37.64 to 37.76)	1279	25440	48.78 (48.71 to 48.84)	304	13270.13	18.74 (18.70 to 18.79)	666	15196	34.37 (34.30 to 34.43)
	IMD=4	486	13676.53	35.56 (35.51 to 35.62)	763	16155	46.58 (46.52 to 46.65)	168	8242.322	16.41 (16.36 to 16.45)	345	9271	29.38 (29.32 to 29.44)
	IMD=5	691	18581.83	36.69 (36.64 to 36.75)	1065	21927	47.23 (47.17 to 47.30)	284	11648.04	18.97 (18.92 to 19.02)	622	13365	34.90 (34.83 to 34.96)
	IMD=6	522	13376.55	38.71 (38.65 to 38.76)	826	15838	51.30 (51.24 to 51.37)	199	8156.241	19.50 (19.45 to 19.55)	453	9375	36.88 (36.82 to 36.95)
	IMD=7	394	11193.89	36.03 (35.97 to 36.08)	645	13325	49.18 (49.12 to 49.25)	141	6158.593	18.19 (18.14 to 18.24)	324	7048	35.14 (35.07 to 35.20)
	IMD=8	450	11774.95	38.53 (38.47 to 38.59)	754	13963	54.04 (53.97 to 54.11)	155	6508.616	19.55 (19.51 to 19.60)	345	7514	35.60 (35.53 to 35.66)
	IMD=9	442	11777.11	39.15 (39.09 to 39.20)	742	14145	53.77 (53.70 to 53.83)	159	6165.733	20.62 (20.57 to 20.67)	319	7100	34.85 (34.78 to 34.91)
	Most Deprivation	825	20523.87	41.46 (41.40 to 41.52)	1360	24491	56.58 (56.51 to 56.65)	244	10912.91	17.75 (17.71 to 17.80)	480	12372	29.77 (29.71 to 29.82)
2007	Least Deprivation	422	12412.03	33.19 (33.14 to 33.25)	678	14718	43.52 (43.46 to 43.58)	172	8203.598	17.22 (17.17 to 17.26)	370	9338	31.07 (31.00 to 31.13)
	IMD=2	636	19670.98	31.34 (31.29 to 31.39)	1010	23332	41.63 (41.57 to 41.69)	265	12422.32	17.07 (17.03 to 17.12)	574	14070	31.47 (31.41 to 31.53)
	IMD=3	800	21446.2	36.59 (36.53 to 36.64)	1286	25853	48.35 (48.29 to 48.42)	375	13488.23	22.80 (22.75 to 22.85)	742	15498	37.57 (37.50 to 37.64)
	IMD=4	475	13761.59	34.03 (33.97 to 34.08)	789	16466	46.70 (46.63 to 46.76)	192	8345.752	18.97 (18.92 to 19.02)	401	9462	33.54 (33.47 to 33.60)
	IMD=5	680	18691.76	35.47 (35.41 to 35.52)	1084	22230	46.78 (46.72 to 46.84)	252	11861.21	16.82 (16.78 to 16.87)	605	13669	33.63 (33.56 to 33.69)
	IMD=6	548	13409.37	39.31 (39.25 to 39.36)	851	16014	51.27 (51.21 to 51.34)	206	8296.665	19.91 (19.87 to 19.96)	458	9547	36.66 (36.59 to 36.72)
	IMD=7	460	11229.17	41.95 (41.89 to 42.01)	711	13479	53.59 (53.53 to 53.66)	137	6300.244	17.61 (17.56 to 17.65)	319	7171	34.31 (34.24 to 34.37)
	IMD=8	476	11807.89	40.89 (40.83 to 40.95)	786	14216	55.26 (55.20 to 55.33)	188	6607.496	23.07 (23.02 to 23.13)	378	7624	38.38 (38.31 to 38.45)
	IMD=9	524	11851.19	45.62 (45.56 to 45.68)	841	14303	59.91 (59.83 to 59.98)	131	6267.518	17.23 (17.19 to 17.28)	307	7227	33.16 (33.09 to 33.22)
	Most Deprivation	814	20669.94	40.36 (40.30 to 40.41)	1414	25038	57.24 (57.17 to 57.31)	244	10989.73	17.69 (17.65 to 17.74)	494	12552	30.30 (30.24 to 30.36)
2008	Least Deprivation	475	12630.26	35.90 (35.84 to 35.95)	712	14868	45.36 (45.30 to 45.42)	195	8342.951	18.66 (18.61 to 18.70)	398	9417	32.98 (32.92 to 33.04)
	IMD=2	620	19924.97	30.55 (30.50 to 30.60)	1023	23576	41.88 (41.82 to 41.94)	292	12637.78	18.68 (18.63 to 18.73)	618	14261	33.13 (33.07 to 33.19)
	IMD=3	821	21893.32	36.57 (36.51 to 36.62)	1296	26146	48.06 (48.00 to 48.12)	327	13910.77	19.60 (19.55 to 19.64)	721	15844	35.86 (35.80 to 35.93)
	IMD=4	514	14042.43	36.50 (36.44 to 36.55)	842	16694	49.45 (49.39 to 49.52)	211	8500.819	20.15 (20.10 to 20.20)	417	9631	33.80 (33.74 to 33.86)
	IMD=5	663	18925.88	34.31 (34.26 to 34.37)	1063	22479	45.81 (45.75 to 45.87)	326	12108.94	21.16 (21.11 to 21.21)	674	13900	36.45 (36.38 to 36.51)
	IMD=6	481	13524.67	35.28 (35.23 to 35.34)	797	16177	48.32 (48.26 to 48.38)	203	8422.848	19.38 (19.33 to 19.43)	462	9691	36.63 (36.57 to 36.70)
	IMD=7	410	11343.04	36.83 (36.77 to 36.88)	687	13642	50.88 (50.81 to 50.94)	166	6361.687	21.10 (21.05 to 21.15)	360	7251	38.72 (38.65 to 38.79)
	IMD=8	467	11939.32	39.76 (39.71 to 39.82)	774	14387	53.86 (53.79 to 53.92)	159	6701.312	19.42 (19.37 to 19.46)	374	7744	37.68 (37.61 to 37.74)
	IMD=9	477	12021.41	41.28 (41.22 to 41.34)	854	14553	60.05 (59.98 to 60.12)	154	6421.577	19.96 (19.91 to 20.01)	304	7363	33.00 (32.94 to 33.07)

	Most Deprivation	866	21161.18	42.32 (42.26 to 42.38)	1508	25642	59.96 (59.89 to 60.03)	264	11309.46	19.14 (19.09 to 19.19)	546	12879	33.21 (33.15 to 33.27)
2009	Least Deprivation	424	12815.09	32.04 (31.99 to 32.09)	668	15156	41.54 (41.48 to 41.60)	220	8505.147	21.29 (21.24 to 21.34)	452	9654	36.74 (36.68 to 36.81)
	IMD=2	629	20207.62	30.95 (30.90 to 31.00)	1028	23829	41.92 (41.87 to 41.98)	297	12836.55	18.53 (18.48 to 18.57)	614	14532	32.18 (32.11 to 32.24)
	IMD=3	783	22261.83	34.40 (34.35 to 34.45)	1333	26619	48.47 (48.40 to 48.53)	393	14200.41	22.76 (22.71 to 22.81)	806	16237	38.91 (38.84 to 38.98)
	IMD=4	518	14354.42	35.94 (35.88 to 35.99)	855	17038	49.46 (49.40 to 49.53)	198	8753.955	18.69 (18.64 to 18.73)	413	9886	32.96 (32.89 to 33.02)
	IMD=5	624	19189.24	32.11 (32.05 to 32.16)	1058	22790	45.00 (44.94 to 45.06)	333	12317.98	21.23 (21.18 to 21.28)	716	14124	38.11 (38.04 to 38.18)
	IMD=6	507	13762.22	36.35 (36.29 to 36.40)	838	16427	49.60 (49.54 to 49.67)	245	8616.394	23.00 (22.95 to 23.05)	506	9905	39.21 (39.14 to 39.28)
	IMD=7	413	11427.65	37.32 (37.27 to 37.38)	711	13802	52.70 (52.63 to 52.76)	174	6424.95	22.22 (22.17 to 22.28)	383	7365	40.87 (40.80 to 40.94)
	IMD=8	460	12119.35	38.61 (38.56 to 38.67)	811	14628	55.39 (55.32 to 55.46)	207	6715.97	25.35 (25.29 to 25.40)	418	7805	42.13 (42.06 to 42.20)
	IMD=9	493	12169.91	42.18 (42.12 to 42.24)	841	14839	57.93 (57.86 to 58.00)	193	6521.202	24.85 (24.79 to 24.90)	369	7482	39.29 (39.22 to 39.36)
	Most Deprivation	885	21575.89	42.43 (42.38 to 42.49)	1563	26315	60.54 (60.47 to 60.61)	277	11511.04	19.49 (19.44 to 19.54)	570	13188	33.79 (33.72 to 33.85)
2010	Least Deprivation	424	13058.2	30.95 (30.90 to 31.00)	688	15369	42.03 (41.97 to 42.09)	231	8650.836	21.69 (21.64 to 21.74)	465	9846	36.81 (36.74 to 36.87)
	IMD=2	633	20354.87	30.35 (30.30 to 30.40)	1039	24082	41.33 (41.28 to 41.39)	344	13095.41	20.85 (20.80 to 20.90)	689	14831	35.40 (35.34 to 35.47)
	IMD=3	885	22608.66	38.10 (38.04 to 38.16)	1422	27125	50.38 (50.32 to 50.45)	374	14512.51	21.16 (21.11 to 21.21)	820	16654	38.38 (38.31 to 38.45)
	IMD=4	498	14517.01	34.35 (34.30 to 34.40)	832	17373	47.40 (47.33 to 47.46)	220	8929.552	20.27 (20.22 to 20.32)	443	10114	34.54 (34.47 to 34.60)
	IMD=5	620	19479.2	30.91 (30.86 to 30.96)	1083	23034	45.32 (45.26 to 45.38)	343	12500.34	21.74 (21.69 to 21.79)	713	14382	37.63 (37.56 to 37.70)
	IMD=6	532	13991.85	37.99 (37.93 to 38.04)	876	16789	51.17 (51.10 to 51.23)	249	8795.614	22.90 (22.84 to 22.95)	535	10139	40.91 (40.84 to 40.98)
	IMD=7	417	11578.04	37.04 (36.98 to 37.09)	725	14057	52.69 (52.62 to 52.75)	169	6508.816	21.81 (21.76 to 21.86)	367	7469	38.91 (38.84 to 38.98)
	IMD=8	480	12314.46	39.68 (39.62 to 39.74)	811	14997	54.51 (54.45 to 54.58)	179	6821.7	21.66 (21.61 to 21.72)	400	7949	39.89 (39.82 to 39.96)
	IMD=9	481	12481.29	39.87 (39.81 to 39.93)	874	15249	58.04 (57.97 to 58.11)	177	6652.975	22.59 (22.54 to 22.65)	352	7680	37.11 (37.05 to 37.18)
	Most Deprivation	816	22093.2	37.98 (37.92 to 38.04)	1498	27034	56.40 (56.33 to 56.47)	306	11682.32	22.09 (22.04 to 22.14)	610	13402	36.15 (36.08 to 36.21)
2011	Least Deprivation	492	13190.4	36.71 (36.66 to 36.77)	783	15594	48.43 (48.37 to 48.50)	201	8750.023	18.70 (18.66 to 18.75)	434	10030	33.59 (33.53 to 33.65)
	IMD=2	630	20409.03	29.75 (29.70 to 29.80)	1057	24028	42.16 (42.10 to 42.22)	280	13293.75	16.64 (16.59 to 16.68)	608	15123	30.54 (30.48 to 30.60)
	IMD=3	896	22867.05	38.22 (38.17 to 38.28)	1455	27451	51.01 (50.94 to 51.07)	399	14856.1	22.04 (21.99 to 22.09)	834	17064	38.11 (38.05 to 38.18)
	IMD=4	487	14636.63	33.18 (33.13 to 33.24)	821	17532	46.46 (46.40 to 46.52)	233	9067.154	21.04 (20.99 to 21.09)	478	10337	36.42 (36.35 to 36.49)
	IMD=5	755	19660.99	37.34 (37.28 to 37.40)	1193	23283	49.24 (49.18 to 49.31)	305	12650.32	19.15 (19.10 to 19.19)	689	14651	35.56 (35.50 to 35.63)
	IMD=6	526	14060.29	37.05 (36.99 to 37.10)	906	16861	52.40 (52.34 to 52.47)	243	8850.574	22.30 (22.24 to 22.35)	524	10265	39.59 (39.52 to 39.66)
	IMD=7	460	11665.86	40.66 (40.60 to 40.72)	777	14120	56.33 (56.26 to 56.40)	178	6521.727	22.46 (22.41 to 22.51)	356	7595	37.00 (36.93 to 37.06)
	IMD=8	534	12483.63	43.82 (43.76 to 43.88)	884	15164	59.00 (58.93 to 59.07)	200	6935.307	24.16 (24.10 to 24.21)	419	8049	41.29 (41.22 to 41.36)
	IMD=9	516	12732.94	41.65 (41.60 to 41.71)	919	15572	60.29 (60.21 to 60.36)	166	6759.458	21.04 (20.99 to 21.09)	375	7835	39.07 (39.00 to 39.14)

	Most Deprivation	1034	22457.98	48.04 (47.98 to 48.11)	1766	27652	65.21 (65.14 to 65.28)	290	11792.7	20.43 (20.38 to 20.48)	610	13653	35.87 (35.80 to 35.93)
2012	Least Deprivation	503	13281.99	36.75 (36.69 to 36.80)	773	15653	46.82 (46.76 to 46.89)	248	8849.717	22.64 (22.59 to 22.69)	460	10127	34.96 (34.90 to 35.03)
	IMD=2	597	20697.63	28.18 (28.13 to 28.23)	988	24104	39.50 (39.44 to 39.55)	326	13495.84	18.97 (18.92 to 19.02)	641	15277	31.69 (31.63 to 31.75)
	IMD=3	901	23067.09	37.71 (37.66 to 37.77)	1503	27606	52.01 (51.95 to 52.08)	409	15037.11	22.07 (22.02 to 22.12)	861	17293	38.36 (38.30 to 38.43)
	IMD=4	563	14800.81	38.16 (38.10 to 38.21)	928	17600	52.15 (52.08 to 52.21)	212	9219.59	18.81 (18.76 to 18.86)	466	10504	34.85 (34.78 to 34.91)
	IMD=5	738	19664.67	36.89 (36.83 to 36.94)	1234	23330	51.16 (51.09 to 51.22)	308	12851.49	18.90 (18.85 to 18.95)	696	14778	35.47 (35.41 to 35.54)
	IMD=6	557	14210.24	38.18 (38.12 to 38.23)	914	17027	51.93 (51.86 to 51.99)	255	8967.628	23.06 (23.01 to 23.11)	552	10443	40.64 (40.57 to 40.71)
	IMD=7	468	11833.38	40.81 (40.75 to 40.86)	778	14306	55.67 (55.60 to 55.74)	198	6603.201	24.88 (24.83 to 24.94)	402	7649	41.45 (41.38 to 41.53)
	IMD=8	533	12674.66	42.88 (42.82 to 42.94)	913	15361	59.94 (59.87 to 60.01)	217	7057.552	25.56 (25.50 to 25.62)	461	8189	44.32 (44.24 to 44.39)
	IMD=9	537	13031.13	43.53 (43.47 to 43.59)	954	15846	62.00 (61.93 to 62.08)	170	6871.236	21.22 (21.17 to 21.27)	366	7940	37.42 (37.35 to 37.49)
	Most Deprivation	1014	22861.5	46.02 (45.95 to 46.08)	1903	28115	69.25 (69.17 to 69.32)	277	12087.89	19.31 (19.26 to 19.36)	622	13863	36.30 (36.24 to 36.37)
2013	Least Deprivation	443	13355.22	32.61 (32.55 to 32.66)	717	15851	43.39 (43.33 to 43.45)	254	8922.598	22.53 (22.47 to 22.58)	504	10270	37.18 (37.12 to 37.25)
	IMD=2	599	20887.17	27.81 (27.76 to 27.86)	968	24369	38.07 (38.01 to 38.12)	304	13519.9	17.44 (17.40 to 17.49)	637	15378	30.85 (30.79 to 30.91)
	IMD=3	902	22973.62	38.32 (38.26 to 38.38)	1517	27778	52.67 (52.61 to 52.74)	395	15162.21	21.18 (21.13 to 21.23)	808	17545	35.37 (35.31 to 35.44)
	IMD=4	525	14983.39	34.67 (34.61 to 34.72)	889	17913	48.74 (48.68 to 48.80)	209	9343.789	17.69 (17.64 to 17.73)	451	10684	32.89 (32.83 to 32.96)
	IMD=5	687	19668.61	34.25 (34.20 to 34.30)	1149	23435	47.42 (47.36 to 47.48)	306	12935.92	18.75 (18.70 to 18.79)	659	14933	33.35 (33.29 to 33.41)
	IMD=6	515	14269.28	36.02 (35.96 to 36.07)	876	17170	50.06 (50.00 to 50.13)	220	9002.289	19.52 (19.47 to 19.56)	516	10488	37.60 (37.53 to 37.67)
	IMD=7	451	12044.01	38.74 (38.68 to 38.79)	798	14650	55.71 (55.65 to 55.78)	171	6622.647	21.22 (21.17 to 21.27)	386	7706	39.06 (38.99 to 39.12)
	IMD=8	487	12937.74	38.92 (38.86 to 38.98)	878	15820	56.47 (56.41 to 56.54)	200	7130.051	23.08 (23.03 to 23.14)	447	8354	42.01 (41.94 to 42.08)
	IMD=9	525	13339.56	41.09 (41.04 to 41.15)	935	16250	59.12 (59.05 to 59.19)	171	7061.289	21.01 (20.96 to 21.06)	368	8129	36.92 (36.86 to 36.99)
	Most Deprivation	1041	23139.92	46.87 (46.80 to 46.93)	1938	28647	69.47 (69.39 to 69.54)	277	12273.69	18.70 (18.66 to 18.75)	619	14132	35.12 (35.05 to 35.18)
2014	Least Deprivation	483	13488.45	34.20 (34.15 to 34.25)	774	15965	45.61 (45.54 to 45.67)	244	9028.564	21.27 (21.21 to 21.32)	484	10381	34.98 (34.92 to 35.05)
	IMD=2	621	21122.49	28.61 (28.56 to 28.66)	995	24585	39.18 (39.13 to 39.24)	291	13712.47	16.42 (16.37 to 16.46)	627	15530	29.99 (29.93 to 30.05)
	IMD=3	927	22970.65	39.80 (39.75 to 39.86)	1541	27798	53.64 (53.57 to 53.71)	399	15295.42	20.85 (20.80 to 20.90)	821	17700	35.50 (35.44 to 35.57)
	IMD=4	517	15030.78	34.77 (34.71 to 34.82)	882	17975	48.53 (48.46 to 48.59)	232	9434.976	20.05 (20.00 to 20.10)	459	10792	33.09 (33.03 to 33.16)
	IMD=5	696	19651.52	34.92 (34.87 to 34.98)	1166	23442	47.88 (47.81 to 47.94)	291	13080.65	17.55 (17.51 to 17.60)	639	14971	32.28 (32.22 to 32.34)
	IMD=6	512	14384.91	34.99 (34.93 to 35.04)	894	17225	50.55 (50.48 to 50.61)	233	9097.675	20.40 (20.35 to 20.45)	530	10563	37.98 (37.91 to 38.04)
	IMD=7	465	12167.01	40.04 (39.99 to 40.10)	815	14804	56.96 (56.89 to 57.02)	168	6650.25	20.31 (20.26 to 20.36)	387	7714	38.53 (38.46 to 38.60)
	IMD=8	565	13030.06	45.43 (45.37 to 45.49)	979	15939	62.84 (62.76 to 62.91)	172	7175.288	19.72 (19.67 to 19.77)	413	8404	38.50 (38.43 to 38.56)
	IMD=9	510	13630.77	38.55 (38.50 to 38.61)	928	16642	56.85 (56.79 to 56.92)	187	7280.572	21.71 (21.66 to 21.76)	385	8346	37.66 (37.60 to 37.73)

	Most Deprivation	991	23446.59	44.06 (44.00 to 44.12)	1898	29161	66.55 (66.47 to 66.62)	291	12566.64	19.61 (19.57 to 19.66)	648	14430	36.51 (36.44 to 36.58)
2015	Least Deprivation	467	13641.97	32.16 (32.10 to 32.21)	739	16065	42.63 (42.57 to 42.69)	256	9109.281	22.08 (22.03 to 22.14)	525	10477	37.54 (37.47 to 37.60)
	IMD=2	587	21438.42	27.11 (27.07 to 27.16)	954	24920	37.75 (37.69 to 37.80)	315	13913.72	17.49 (17.45 to 17.54)	624	15750	29.39 (29.33 to 29.45)
	IMD=3	929	23336.56	39.33 (39.27 to 39.38)	1534	28136	53.18 (53.12 to 53.25)	401	15549.57	20.34 (20.29 to 20.39)	828	17856	35.22 (35.15 to 35.28)
	IMD=4	493	15186.16	32.60 (32.54 to 32.65)	848	18123	46.28 (46.22 to 46.34)	238	9499.11	20.04 (19.99 to 20.09)	488	10869	34.77 (34.70 to 34.83)
	IMD=5	652	19863.67	31.91 (31.86 to 31.96)	1096	23646	44.55 (44.49 to 44.61)	328	13219	19.59 (19.54 to 19.64)	653	15145	32.51 (32.44 to 32.57)
	IMD=6	531	14555.91	35.69 (35.63 to 35.74)	887	17421	49.57 (49.51 to 49.63)	238	9252.597	20.47 (20.42 to 20.52)	516	10701	36.36 (36.30 to 36.43)
	IMD=7	444	12300	37.66 (37.61 to 37.72)	759	14959	52.50 (52.43 to 52.56)	212	6700.539	25.32 (25.26 to 25.37)	407	7781	40.05 (39.98 to 40.12)
	IMD=8	543	13172.17	42.70 (42.64 to 42.76)	945	16097	59.69 (59.62 to 59.76)	220	7269.174	24.90 (24.84 to 24.95)	442	8515	40.66 (40.59 to 40.73)
	IMD=9	544	13974.21	40.57 (40.51 to 40.62)	998	17063	59.89 (59.82 to 59.96)	181	7464.832	20.82 (20.77 to 20.87)	365	8561	35.12 (35.06 to 35.19)
	Most Deprivation	1000	24163.3	43.24 (43.18 to 43.30)	1900	30010	64.56 (64.49 to 64.64)	337	12930.6	22.05 (22.00 to 22.10)	690	14858	37.90 (37.83 to 37.97)
2016	Least Deprivation	489	13755.03	33.25 (33.19 to 33.30)	761	16226	43.12 (43.06 to 43.18)	270	9231.425	23.20 (23.15 to 23.25)	522	10645	36.72 (36.65 to 36.78)
	IMD=2	598	21787.28	26.68 (26.63 to 26.73)	953	25163	36.93 (36.87 to 36.98)	343	14062.38	18.68 (18.63 to 18.73)	670	15891	30.89 (30.83 to 30.95)
	IMD=3	869	23622.1	35.80 (35.75 to 35.86)	1466	28409	49.65 (49.58 to 49.71)	449	15825.32	22.53 (22.48 to 22.59)	859	18141	35.78 (35.71 to 35.84)
	IMD=4	523	15352.56	33.88 (33.83 to 33.94)	852	18212	46.05 (45.99 to 46.12)	223	9599.464	18.62 (18.58 to 18.67)	483	10970	33.87 (33.81 to 33.94)
	IMD=5	639	20200.3	30.49 (30.44 to 30.54)	1082	23813	43.21 (43.15 to 43.27)	354	13363.65	20.79 (20.74 to 20.84)	690	15294	33.97 (33.91 to 34.04)
	IMD=6	528	14869.42	34.66 (34.61 to 34.71)	883	17663	48.39 (48.32 to 48.45)	228	9478.738	18.87 (18.83 to 18.92)	511	10917	35.16 (35.09 to 35.22)
	IMD=7	448	12426.55	37.37 (37.32 to 37.43)	773	15040	53.03 (52.96 to 53.09)	193	6752.03	22.74 (22.69 to 22.79)	418	7844	40.83 (40.76 to 40.90)
	IMD=8	527	13364.91	41.25 (41.19 to 41.31)	919	16280	57.71 (57.64 to 57.78)	199	7337.528	22.05 (22.00 to 22.11)	416	8508	38.06 (37.99 to 38.12)
	IMD=9	526	14352.07	38.03 (37.97 to 38.09)	998	17407	58.54 (58.47 to 58.61)	168	7618.557	19.06 (19.02 to 19.11)	367	8723	34.69 (34.62 to 34.75)
	Most Deprivation	1030	24946.98	43.20 (43.14 to 43.26)	1920	30749	63.88 (63.80 to 63.95)	321	13253.46	20.73 (20.68 to 20.78)	661	15211	35.62 (35.55 to 35.68)
2017	Least Deprivation	485	13866.67	33.15 (33.10 to 33.21)	757	16322	43.10 (43.04 to 43.16)	291	9345.626	24.55 (24.50 to 24.61)	567	10799	39.21 (39.15 to 39.28)
	IMD=2	615	22121.58	27.14 (27.09 to 27.18)	972	25649	36.69 (36.63 to 36.74)	356	14256.39	19.17 (19.12 to 19.22)	696	16169	31.79 (31.73 to 31.85)
	IMD=3	784	24071.66	31.65 (31.60 to 31.70)	1344	28856	44.96 (44.90 to 45.02)	445	16008.86	21.94 (21.89 to 22.00)	922	18419	37.64 (37.57 to 37.71)
	IMD=4	500	15447.52	32.34 (32.28 to 32.39)	806	18400	43.37 (43.31 to 43.43)	229	9628.802	18.95 (18.90 to 18.99)	478	11045	33.17 (33.11 to 33.23)
	IMD=5	636	20342.61	30.07 (30.02 to 30.12)	1068	23945	41.97 (41.91 to 42.03)	304	13444.61	17.67 (17.63 to 17.72)	672	15402	32.81 (32.75 to 32.87)
	IMD=6	477	15079.69	31.12 (31.06 to 31.17)	819	17950	44.16 (44.10 to 44.22)	244	9557.881	20.15 (20.10 to 20.19)	529	11043	35.97 (35.91 to 36.04)
	IMD=7	428	12684.6	35.66 (35.61 to 35.72)	713	15419	48.50 (48.44 to 48.57)	179	6822.839	21.10 (21.05 to 21.15)	429	7962	41.34 (41.27 to 41.41)
	IMD=8	525	13539.13	40.48 (40.42 to 40.54)	929	16595	57.66 (57.59 to 57.73)	204	7391.362	22.52 (22.47 to 22.57)	438	8599	39.69 (39.62 to 39.75)
	IMD=9	532	14766.92	37.61 (37.56 to 37.67)	1010	17844	58.16 (58.09 to 58.23)	182	7759.165	19.93 (19.88 to 19.98)	375	8893	34.70 (34.63 to 34.76)

	Most Deprivation	1018	25836.24	40.57 (40.51 to 40.63)	1926	31729	61.84 (61.77 to 61.91)	328	13542.93	20.83 (20.77 to 20.88)	716	15565	37.90 (37.83 to 37.96)
2018	Least Deprivation	491	14669.4	31.21 (31.16 to 31.26)	786	17136	42.08 (42.02 to 42.14)	305	9737.153	24.38 (24.33 to 24.44)	603	11312	39.61 (39.54 to 39.67)
	IMD=2	543	22507.23	23.31 (23.27 to 23.36)	902	26027	33.36 (33.30 to 33.41)	361	14406.08	18.82 (18.78 to 18.87)	712	16361	31.43 (31.37 to 31.49)
	IMD=3	771	24692.07	30.26 (30.21 to 30.31)	1311	29311	43.31 (43.25 to 43.37)	449	16188.13	21.61 (21.56 to 21.66)	910	18675	36.45 (36.38 to 36.51)
	IMD=4	505	16433.18	30.30 (30.25 to 30.35)	860	19357	43.57 (43.51 to 43.63)	259	10211.06	20.09 (20.04 to 20.14)	495	11635	32.54 (32.48 to 32.60)
	IMD=5	620	21144.94	28.71 (28.66 to 28.76)	1038	24733	40.32 (40.26 to 40.37)	338	13920.94	18.75 (18.70 to 18.80)	690	15909	32.36 (32.30 to 32.43)
	IMD=6	494	15257.84	31.93 (31.88 to 31.98)	814	18075	43.81 (43.75 to 43.87)	257	9647.986	21.08 (21.03 to 21.13)	566	11133	38.16 (38.09 to 38.22)
	IMD=7	396	13303.03	31.92 (31.87 to 31.97)	713	15950	47.19 (47.13 to 47.26)	173	6918.667	20.02 (19.97 to 20.07)	398	8068	37.67 (37.60 to 37.73)
	IMD=8	454	14070.36	33.80 (33.75 to 33.85)	838	17070	50.71 (50.64 to 50.77)	222	7507.6	23.90 (23.85 to 23.95)	457	8762	40.42 (40.35 to 40.49)
	IMD=9	569	15307.99	38.86 (38.80 to 38.91)	1015	18475	56.13 (56.06 to 56.19)	217	8002.275	23.06 (23.01 to 23.11)	408	9192	36.31 (36.24 to 36.37)
	Most Deprivation	982	26875.98	38.07 (38.02 to 38.13)	1857	32708	58.01 (57.94 to 58.08)	346	13904.25	21.26 (21.21 to 21.31)	705	15951	36.52 (36.45 to 36.58)
2019	Least Deprivation	497	14698.2	31.00 (30.95 to 31.05)	740	17365	38.84 (38.78 to 38.90)	355	9668.985	28.46 (28.40 to 28.52)	662	11453	42.51 (42.44 to 42.58)
	IMD=2	542	22683.86	23.39 (23.35 to 23.43)	913	26715	32.91 (32.86 to 32.96)	375	14304.12	19.57 (19.53 to 19.62)	760	16686	32.65 (32.58 to 32.71)
	IMD=3	603	18758.85	31.07 (31.02 to 31.12)	1014	29785	32.69 (32.64 to 32.74)	384	11902.94	25.19 (25.14 to 25.25)	788	18897	31.08 (31.02 to 31.14)
	IMD=4	441	14045.03	31.22 (31.17 to 31.27)	731	19990	35.95 (35.90 to 36.01)	254	8553.24	23.51 (23.46 to 23.56)	490	11986	30.97 (30.91 to 31.03)
	IMD=5	553	19309.44	27.66 (27.61 to 27.71)	943	25091	35.77 (35.72 to 35.83)	340	12682.84	20.61 (20.56 to 20.66)	675	16168	30.95 (30.89 to 31.01)
	IMD=6	453	14061.91	31.81 (31.76 to 31.87)	781	18349	41.54 (41.48 to 41.60)	256	8770.514	22.53 (22.47 to 22.58)	542	11216	36.17 (36.10 to 36.23)
	IMD=7	439	13484.13	36.17 (36.12 to 36.23)	734	16920	47.23 (47.17 to 47.29)	198	6570.992	23.66 (23.61 to 23.71)	401	8138	37.14 (37.08 to 37.21)
	IMD=8	437	13422.91	35.06 (35.01 to 35.12)	797	17735	47.51 (47.45 to 47.58)	197	6869.361	22.61 (22.55 to 22.66)	419	8826	36.65 (36.58 to 36.72)
	IMD=9	554	13813.97	41.80 (41.74 to 41.86)	1018	18950	55.02 (54.95 to 55.09)	193	7200.791	23.34 (23.29 to 23.40)	382	9338	33.51 (33.45 to 33.58)
	Most Deprivation	887	24026.73	38.38 (38.32 to 38.44)	1700	33862	51.41 (51.35 to 51.48)	300	12422.41	20.32 (20.27 to 20.37)	631	16285	31.66 (31.60 to 31.72)
**East Midlands**													
2004	Least Deprivation	452	11246.74	40.60 (40.54 to 40.66)	681	14385	46.04 (45.97 to 46.10)	117	6223.477	17.95 (17.91 to 18.00)	212	7478	26.38 (26.32 to 26.44)
	IMD=2	618	16895.18	35.41 (35.35 to 35.46)	1038	22024	44.72 (44.66 to 44.78)	210	10907.47	16.36 (16.32 to 16.41)	475	13496	28.30 (28.24 to 28.36)
	IMD=3	454	12801	34.57 (34.52 to 34.62)	769	15835	47.08 (47.02 to 47.14)	179	7897.303	19.22 (19.17 to 19.26)	351	9265	31.12 (31.06 to 31.18)
	IMD=4	284	9465.366	32.19 (32.14 to 32.24)	467	11607	42.49 (42.43 to 42.55)	93	4785.624	15.82 (15.78 to 15.86)	217	5682	30.75 (30.69 to 30.81)
	IMD=5	302	8571.962	35.88 (35.83 to 35.94)	499	10658	47.30 (47.24 to 47.36)	85	4712.038	15.49 (15.45 to 15.54)	233	5717	32.91 (32.85 to 32.98)
	IMD=6	281	7788.548	36.05 (36.00 to 36.11)	427	9022	46.57 (46.50 to 46.63)	97	4374.229	16.96 (16.92 to 17.01)	203	4915	31.09 (31.02 to 31.15)
	IMD=7	207	6856.761	33.84 (33.79 to 33.89)	340	8259	45.53 (45.47 to 45.59)	84	3233.295	20.73 (20.68 to 20.78)	156	3708	32.61 (32.54 to 32.67)
	IMD=8	230	6223.357	38.04 (37.98 to 38.09)	382	7210	53.76 (53.69 to 53.82)	70	3255.975	17.10 (17.05 to 17.14)	209	3691	42.41 (42.34 to 42.48)

	IMD=9	183	6357.229	29.94 (29.89 to 29.99)	243	7166	34.89 (34.83 to 34.94)	56	3011.012	14.39 (14.35 to 14.43)	87	3263	20.04 (19.99 to 20.09)
	Most Deprivation	194	7157.594	27.64 (27.59 to 27.69)	260	7863	33.09 (33.03 to 33.14)	48	3308.077	11.63 (11.60 to 11.67)	74	3532	16.63 (16.59 to 16.68)
2005	Least Deprivation	463	11237.04	41.42 (41.36 to 41.48)	728	13323	53.92 (53.85 to 53.98)	106	6322.795	17.19 (17.14 to 17.23)	227	6913	32.64 (32.58 to 32.70)
	IMD=2	586	16448.19	34.89 (34.83 to 34.94)	999	19373	49.49 (49.42 to 49.55)	224	10685.3	18.20 (18.15 to 18.25)	483	11906	33.16 (33.09 to 33.22)
	IMD=3	468	12753.92	36.28 (36.22 to 36.33)	773	15055	49.71 (49.65 to 49.78)	154	7954.897	16.54 (16.50 to 16.59)	356	8895	32.68 (32.62 to 32.75)
	IMD=4	292	9033.525	34.05 (34.00 to 34.10)	477	10651	47.63 (47.56 to 47.69)	83	4459.513	14.75 (14.71 to 14.80)	215	5012	33.89 (33.82 to 33.95)
	IMD=5	300	8525.259	35.88 (35.83 to 35.94)	499	10106	49.85 (49.78 to 49.91)	90	4667.784	16.31 (16.26 to 16.35)	258	5290	38.80 (38.73 to 38.86)
	IMD=6	283	7930.365	36.31 (36.26 to 36.37)	458	9334	48.80 (48.74 to 48.87)	95	4414.582	16.82 (16.78 to 16.87)	196	4974	29.73 (29.67 to 29.79)
	IMD=7	259	7014.582	42.44 (42.38 to 42.50)	403	8502	53.90 (53.83 to 53.96)	90	3218.582	22.03 (21.98 to 22.08)	186	3682	39.12 (39.05 to 39.18)
	IMD=8	232	6234.439	38.13 (38.07 to 38.19)	399	7321	55.40 (55.33 to 55.47)	89	3300.698	21.11 (21.06 to 21.16)	233	3759	46.09 (46.02 to 46.17)
	IMD=9	289	6393.615	48.26 (48.20 to 48.32)	404	7395	58.04 (57.97 to 58.11)	63	3013.993	16.18 (16.13 to 16.22)	113	3291	25.98 (25.92 to 26.04)
	Most Deprivation	282	7039.269	40.62 (40.57 to 40.68)	395	7945	50.14 (50.07 to 50.20)	54	3314.398	13.48 (13.44 to 13.52)	79	3586	17.74 (17.70 to 17.79)
2006	Least Deprivation	450	11455.72	38.58 (38.53 to 38.64)	727	13734	51.60 (51.53 to 51.66)	111	6548.413	14.89 (14.85 to 14.93)	224	7212	29.36 (29.30 to 29.42)
	IMD=2	619	16545.81	36.50 (36.45 to 36.56)	1018	19635	49.84 (49.78 to 49.90)	177	10901.38	13.84 (13.80 to 13.88)	430	12142	28.89 (28.84 to 28.95)
	IMD=3	498	12858.96	37.66 (37.60 to 37.72)	823	15274	51.73 (51.67 to 51.80)	156	8090.108	16.42 (16.37 to 16.46)	356	9063	32.10 (32.04 to 32.17)
	IMD=4	276	9231.57	32.23 (32.17 to 32.28)	454	10939	44.51 (44.45 to 44.57)	72	4552.441	12.75 (12.71 to 12.79)	210	5117	32.76 (32.69 to 32.82)
	IMD=5	306	8694.688	36.35 (36.30 to 36.41)	539	10347	53.09 (53.02 to 53.15)	112	4740.999	19.65 (19.60 to 19.70)	280	5391	40.79 (40.72 to 40.86)
	IMD=6	273	8131.967	34.12 (34.07 to 34.18)	450	9643	46.65 (46.59 to 46.71)	84	4467.206	14.57 (14.53 to 14.62)	183	5027	27.36 (27.30 to 27.42)
	IMD=7	260	7152.909	40.41 (40.35 to 40.47)	440	8736	56.63 (56.56 to 56.70)	61	3213.511	15.45 (15.40 to 15.49)	152	3690	32.01 (31.95 to 32.07)
	IMD=8	257	6253.667	42.49 (42.43 to 42.55)	433	7475	59.36 (59.29 to 59.43)	74	3327.105	17.99 (17.94 to 18.03)	237	3811	47.12 (47.05 to 47.20)
	IMD=9	242	6232.331	40.06 (40.00 to 40.11)	398	7392	56.01 (55.94 to 56.08)	49	3035.239	12.75 (12.71 to 12.79)	97	3359	21.91 (21.85 to 21.96)
	Most Deprivation	295	6987.096	43.81 (43.75 to 43.87)	465	8100	59.15 (59.08 to 59.22)	45	3369.807	11.63 (11.59 to 11.66)	81	3665	18.48 (18.43 to 18.52)
2007	Least Deprivation	442	11929.6	37.95 (37.89 to 38.01)	715	14279	49.81 (49.75 to 49.88)	118	6890.998	16.49 (16.45 to 16.54)	233	7575	29.53 (29.47 to 29.59)
	IMD=2	581	16737.79	33.46 (33.41 to 33.52)	1023	19933	48.44 (48.37 to 48.50)	202	11167.87	15.36 (15.32 to 15.40)	468	12465	30.15 (30.09 to 30.21)
	IMD=3	440	12972.39	33.71 (33.65 to 33.76)	777	15503	48.51 (48.44 to 48.57)	149	8285.897	15.41 (15.36 to 15.45)	357	9274	31.43 (31.37 to 31.49)
	IMD=4	275	9476.819	31.88 (31.83 to 31.93)	471	11375	45.22 (45.16 to 45.28)	98	4693.936	17.27 (17.22 to 17.31)	235	5249	36.12 (36.05 to 36.18)
	IMD=5	283	8812.178	33.91 (33.85 to 33.96)	528	10621	51.55 (51.48 to 51.61)	94	4873.602	16.11 (16.07 to 16.16)	271	5545	38.66 (38.59 to 38.72)
	IMD=6	313	8197.002	39.58 (39.52 to 39.64)	517	9937	53.40 (53.33 to 53.46)	118	4566.029	20.29 (20.24 to 20.34)	214	5132	31.68 (31.62 to 31.74)
	IMD=7	233	7241.062	37.46 (37.41 to 37.52)	390	8923	51.00 (50.93 to 51.06)	66	3253.552	16.82 (16.77 to 16.86)	155	3721	33.17 (33.11 to 33.24)
	IMD=8	255	6299.573	42.17 (42.11 to 42.22)	458	7671	61.40 (61.33 to 61.47)	65	3366.127	15.76 (15.71 to 15.80)	220	3845	43.15 (43.08 to 43.23)

	IMD=9	226	6138.99	39.94 (39.88 to 40.00)	378	7525	53.52 (53.46 to 53.59)	55	3058.932	14.64 (14.60 to 14.68)	118	3409	26.93 (26.87 to 26.98)
	Most Deprivation	256	7036.983	37.59 (37.54 to 37.65)	438	8393	53.46 (53.39 to 53.52)	55	3444.813	13.44 (13.40 to 13.48)	105	3756	22.90 (22.85 to 22.96)
2008	Least Deprivation	445	12714.7	35.08 (35.02 to 35.13)	715	15059	46.67 (46.61 to 46.74)	130	7418.368	16.52 (16.48 to 16.56)	246	8095	28.58 (28.52 to 28.64)
	IMD=2	646	17337.08	36.08 (36.02 to 36.13)	1089	20527	50.68 (50.61 to 50.74)	196	11676.57	14.08 (14.03 to 14.12)	482	12948	29.85 (29.79 to 29.91)
	IMD=3	479	13429.1	34.72 (34.67 to 34.77)	829	15918	49.60 (49.54 to 49.67)	185	8641.366	17.91 (17.87 to 17.96)	385	9602	32.51 (32.45 to 32.57)
	IMD=4	307	9647.365	33.91 (33.86 to 33.97)	497	11518	46.08 (46.02 to 46.15)	94	4817.815	16.07 (16.03 to 16.12)	224	5395	33.09 (33.03 to 33.16)
	IMD=5	297	9103.135	33.64 (33.58 to 33.69)	549	10866	51.56 (51.49 to 51.62)	110	5028.378	18.22 (18.17 to 18.27)	289	5717	40.39 (40.32 to 40.46)
	IMD=6	323	8370.119	39.96 (39.90 to 40.01)	530	10122	53.58 (53.51 to 53.64)	119	4665.572	20.18 (20.13 to 20.23)	225	5276	32.38 (32.32 to 32.44)
	IMD=7	222	7332.52	34.36 (34.31 to 34.42)	388	8992	49.70 (49.64 to 49.77)	79	3289.574	19.66 (19.61 to 19.71)	164	3747	34.33 (34.27 to 34.40)
	IMD=8	253	6384.567	41.76 (41.70 to 41.82)	473	7753	63.04 (62.96 to 63.11)	50	3435.061	11.43 (11.39 to 11.47)	218	3912	41.37 (41.30 to 41.45)
	IMD=9	232	6070.242	42.09 (42.03 to 42.15)	414	7455	59.96 (59.89 to 60.03)	67	3076.186	17.62 (17.58 to 17.67)	137	3438	30.79 (30.73 to 30.85)
	Most Deprivation	275	7120.682	40.65 (40.59 to 40.70)	476	8563	57.01 (56.94 to 57.08)	73	3545.555	16.87 (16.83 to 16.92)	122	3897	25.51 (25.46 to 25.57)
2009	Least Deprivation	494	13295.06	36.97 (36.92 to 37.03)	777	15691	48.46 (48.40 to 48.52)	153	7765.875	19.04 (18.99 to 19.08)	292	8501	31.93 (31.87 to 32.00)
	IMD=2	656	17587.6	36.18 (36.13 to 36.24)	1090	20855	49.68 (49.62 to 49.75)	236	12006.11	16.25 (16.20 to 16.29)	507	13267	30.57 (30.51 to 30.63)
	IMD=3	499	13672.52	35.54 (35.48 to 35.59)	870	16207	51.50 (51.43 to 51.56)	168	8864.12	16.18 (16.14 to 16.22)	375	9880	30.84 (30.78 to 30.90)
	IMD=4	295	9703.86	32.72 (32.67 to 32.77)	515	11559	47.53 (47.46 to 47.59)	109	4920.712	18.36 (18.32 to 18.41)	249	5538	36.39 (36.32 to 36.45)
	IMD=5	320	9269.958	35.78 (35.72 to 35.83)	597	11042	54.93 (54.87 to 55.00)	114	5161.539	18.71 (18.67 to 18.76)	290	5860	39.71 (39.64 to 39.78)
	IMD=6	293	8697.76	35.84 (35.78 to 35.89)	482	10507	47.72 (47.66 to 47.78)	103	4676.178	17.52 (17.48 to 17.57)	212	5308	30.61 (30.55 to 30.67)
	IMD=7	273	7389.301	43.11 (43.05 to 43.17)	457	9090	58.24 (58.17 to 58.31)	84	3308.474	20.75 (20.70 to 20.80)	177	3780	36.59 (36.52 to 36.66)
	IMD=8	220	6501.977	35.09 (35.03 to 35.14)	473	7897	61.84 (61.77 to 61.91)	79	3495.014	18.66 (18.61 to 18.71)	232	3985	44.94 (44.86 to 45.01)
	IMD=9	197	6196.693	33.27 (33.22 to 33.33)	382	7540	53.18 (53.11 to 53.25)	71	3101.221	18.01 (17.96 to 18.06)	140	3496	31.31 (31.25 to 31.37)
	Most Deprivation	232	7315.888	32.35 (32.29 to 32.40)	442	8780	51.37 (51.30 to 51.44)	75	3643.83	17.35 (17.30 to 17.39)	127	4017	25.67 (25.62 to 25.73)
2010	Least Deprivation	522	13900.4	39.17 (39.12 to 39.23)	825	16385	51.16 (51.10 to 51.23)	157	8169.555	18.48 (18.44 to 18.53)	287	8961	29.79 (29.73 to 29.85)
	IMD=2	655	17814.79	35.93 (35.87 to 35.98)	1088	21235	48.86 (48.80 to 48.92)	268	12257.12	18.28 (18.24 to 18.33)	513	13666	29.83 (29.77 to 29.89)
	IMD=3	510	14014.18	35.55 (35.50 to 35.61)	878	16636	50.83 (50.76 to 50.89)	171	9105.432	15.49 (15.44 to 15.53)	385	10173	30.41 (30.35 to 30.47)
	IMD=4	298	9877.394	32.36 (32.31 to 32.41)	529	11776	47.85 (47.79 to 47.92)	106	5027.253	17.71 (17.67 to 17.76)	257	5681	36.78 (36.72 to 36.85)
	IMD=5	303	9515.463	32.90 (32.84 to 32.95)	613	11344	55.35 (55.29 to 55.42)	91	5340.197	14.25 (14.20 to 14.29)	262	6032	34.69 (34.63 to 34.75)
	IMD=6	273	8987.641	32.99 (32.94 to 33.04)	488	10965	47.29 (47.23 to 47.35)	107	4765.856	17.97 (17.92 to 18.01)	224	5404	31.79 (31.73 to 31.85)
	IMD=7	259	7365.637	41.00 (40.94 to 41.05)	445	9078	56.30 (56.23 to 56.36)	61	3359.422	15.02 (14.98 to 15.06)	152	3859	31.20 (31.14 to 31.26)
	IMD=8	265	6619.759	42.19 (42.13 to 42.25)	505	8088	64.55 (64.48 to 64.63)	79	3521.054	18.19 (18.14 to 18.23)	231	4031	43.22 (43.15 to 43.29)

	IMD=9	230	6372.107	39.24 (39.19 to 39.30)	410	7786	56.23 (56.16 to 56.30)	58	3130.634	15.24 (15.20 to 15.29)	145	3545	31.86 (31.79 to 31.92)
	Most Deprivation	299	7473.372	41.42 (41.36 to 41.47)	520	9025	58.53 (58.46 to 58.60)	78	3737.15	18.12 (18.07 to 18.16)	139	4166	28.04 (27.98 to 28.10)
2011	Least Deprivation	546	14376.78	38.56 (38.51 to 38.62)	854	16967	50.08 (50.01 to 50.14)	176	8546.081	20.07 (20.02 to 20.12)	321	9431	31.75 (31.69 to 31.81)
	IMD=2	678	18063.85	36.70 (36.65 to 36.76)	1168	21536	51.22 (51.16 to 51.29)	275	12443.03	17.95 (17.90 to 17.99)	537	13925	30.20 (30.14 to 30.26)
	IMD=3	517	14247.33	35.11 (35.06 to 35.17)	899	16984	50.37 (50.30 to 50.43)	193	9332.668	16.90 (16.85 to 16.94)	398	10447	30.39 (30.33 to 30.45)
	IMD=4	321	9991.477	34.24 (34.19 to 34.30)	571	12002	50.18 (50.11 to 50.24)	102	5212.701	16.62 (16.57 to 16.66)	263	5891	36.19 (36.13 to 36.26)
	IMD=5	277	9639.852	29.60 (29.55 to 29.65)	579	11583	51.12 (51.06 to 51.19)	117	5504.542	17.77 (17.73 to 17.82)	288	6224	37.02 (36.96 to 37.09)
	IMD=6	302	9227	35.17 (35.12 to 35.23)	547	11259	51.75 (51.69 to 51.82)	106	4857.5	17.40 (17.35 to 17.44)	231	5510	32.40 (32.33 to 32.46)
	IMD=7	241	7362.844	38.04 (37.99 to 38.10)	434	9150	55.50 (55.44 to 55.57)	76	3395.379	18.66 (18.61 to 18.71)	175	3886	35.98 (35.91 to 36.04)
	IMD=8	305	6743.14	47.40 (47.34 to 47.47)	574	8271	71.74 (71.66 to 71.81)	74	3576.482	16.99 (16.95 to 17.04)	225	4093	42.08 (42.00 to 42.15)
	IMD=9	288	6457.7	50.85 (50.79 to 50.92)	510	8072	69.50 (69.43 to 69.58)	71	3148.898	19.08 (19.03 to 19.12)	167	3631	36.72 (36.65 to 36.79)
	Most Deprivation	378	7581.733	52.88 (52.82 to 52.95)	642	9345	70.61 (70.54 to 70.69)	95	3808.876	22.49 (22.44 to 22.54)	186	4311	36.39 (36.32 to 36.45)
2012	Least Deprivation	574	14715.38	38.55 (38.49 to 38.60)	905	17411	50.98 (50.91 to 51.05)	175	8924.731	18.95 (18.90 to 19.00)	333	9850	30.96 (30.90 to 31.02)
	IMD=2	664	18247.29	34.62 (34.57 to 34.68)	1149	21717	49.34 (49.28 to 49.41)	235	12742.65	14.91 (14.87 to 14.95)	520	14199	28.46 (28.41 to 28.52)
	IMD=3	515	14466.97	35.14 (35.09 to 35.20)	898	17177	50.29 (50.23 to 50.36)	202	9566.511	17.34 (17.30 to 17.39)	411	10690	30.46 (30.39 to 30.52)
	IMD=4	310	10167.81	33.21 (33.16 to 33.26)	582	12137	50.87 (50.80 to 50.93)	112	5268.526	17.85 (17.81 to 17.90)	240	5957	32.30 (32.24 to 32.36)
	IMD=5	351	9769.043	37.82 (37.76 to 37.87)	646	11691	56.40 (56.33 to 56.46)	124	5529.254	18.74 (18.69 to 18.79)	283	6304	35.74 (35.67 to 35.81)
	IMD=6	321	9812.674	36.47 (36.41 to 36.52)	557	11678	51.77 (51.71 to 51.84)	123	4908.545	19.93 (19.89 to 19.98)	248	5572	34.18 (34.11 to 34.24)
	IMD=7	237	7601.668	37.57 (37.52 to 37.63)	427	9329	54.30 (54.23 to 54.37)	72	3455.6	17.05 (17.01 to 17.10)	153	3934	31.03 (30.97 to 31.09)
	IMD=8	275	6764.307	42.88 (42.82 to 42.94)	554	8368	68.83 (68.75 to 68.90)	81	3574.631	18.56 (18.51 to 18.61)	253	4150	46.51 (46.44 to 46.59)
	IMD=9	274	6588.433	45.51 (45.45 to 45.57)	507	8148	66.97 (66.89 to 67.04)	86	3182.738	22.86 (22.81 to 22.92)	175	3633	38.35 (38.28 to 38.42)
	Most Deprivation	363	7597.988	51.08 (51.02 to 51.15)	653	9417	72.24 (72.16 to 72.32)	95	3822.563	21.42 (21.37 to 21.47)	167	4341	32.24 (32.18 to 32.30)
2013	Least Deprivation	592	14797.46	40.64 (40.59 to 40.70)	960	17635	53.95 (53.88 to 54.02)	157	9177.539	15.79 (15.75 to 15.83)	292	10165	25.70 (25.64 to 25.75)
	IMD=2	730	18277.39	39.02 (38.97 to 39.08)	1182	21900	51.65 (51.58 to 51.71)	286	12861.22	17.78 (17.73 to 17.82)	562	14414	30.10 (30.04 to 30.16)
	IMD=3	553	14636.4	36.44 (36.38 to 36.49)	955	17475	51.53 (51.46 to 51.59)	208	9725.155	17.41 (17.36 to 17.45)	416	10931	29.93 (29.87 to 29.99)
	IMD=4	305	10308.04	32.29 (32.24 to 32.34)	539	12417	46.64 (46.58 to 46.70)	108	5280.879	17.19 (17.15 to 17.24)	251	6017	33.52 (33.46 to 33.58)
	IMD=5	355	9750.294	38.76 (38.70 to 38.81)	602	11688	53.31 (53.25 to 53.38)	130	5499.447	19.51 (19.46 to 19.56)	264	6250	33.58 (33.52 to 33.65)
	IMD=6	371	10038.4	43.49 (43.43 to 43.55)	627	12133	58.76 (58.69 to 58.83)	105	4881.331	17.10 (17.05 to 17.14)	217	5580	29.50 (29.44 to 29.56)
	IMD=7	265	7708.753	41.61 (41.55 to 41.67)	432	9476	53.92 (53.85 to 53.98)	82	3452.446	19.76 (19.71 to 19.81)	148	3932	30.13 (30.07 to 30.19)
	IMD=8	282	6761.391	44.31 (44.25 to 44.37)	547	8395	68.02 (67.94 to 68.09)	72	3589.714	16.39 (16.35 to 16.44)	202	4142	37.48 (37.41 to 37.54)

	IMD=9	267	6669.695	45.45 (45.39 to 45.51)	492	8351	65.14 (65.06 to 65.21)	71	3184.693	18.79 (18.74 to 18.83)	148	3651	32.80 (32.74 to 32.86)
	Most Deprivation	307	7606.453	43.68 (43.62 to 43.74)	621	9571	68.20 (68.12 to 68.27)	71	3890.439	15.71 (15.66 to 15.75)	149	4422	28.20 (28.14 to 28.26)
2014	Least Deprivation	632	14926.95	42.99 (42.93 to 43.05)	954	17929	52.75 (52.68 to 52.82)	208	9491.696	20.34 (20.29 to 20.39)	369	10536	31.51 (31.45 to 31.57)
	IMD=2	694	18262.76	36.97 (36.92 to 37.03)	1124	21995	48.50 (48.44 to 48.56)	284	13018.63	17.22 (17.17 to 17.26)	540	14610	28.34 (28.28 to 28.39)
	IMD=3	567	14711.27	37.55 (37.50 to 37.61)	943	17674	50.73 (50.67 to 50.80)	220	9875.89	18.21 (18.16 to 18.26)	426	11137	29.88 (29.82 to 29.94)
	IMD=4	289	10414.43	30.37 (30.32 to 30.42)	506	12636	43.22 (43.16 to 43.28)	122	5347.518	18.51 (18.46 to 18.55)	239	6070	31.31 (31.25 to 31.37)
	IMD=5	354	9662.026	39.21 (39.15 to 39.26)	611	11723	54.24 (54.17 to 54.31)	121	5485.76	18.27 (18.22 to 18.31)	246	6286	31.07 (31.01 to 31.13)
	IMD=6	311	10146.6	37.40 (37.35 to 37.46)	563	12421	53.28 (53.21 to 53.35)	128	4914.324	20.51 (20.46 to 20.56)	237	5613	32.11 (32.05 to 32.18)
	IMD=7	236	7674.763	37.58 (37.53 to 37.64)	410	9604	51.36 (51.30 to 51.43)	85	3526.628	19.72 (19.67 to 19.77)	152	4018	30.19 (30.13 to 30.25)
	IMD=8	241	6796.126	37.25 (37.19 to 37.30)	471	8543	57.39 (57.32 to 57.46)	77	3624.619	17.29 (17.24 to 17.34)	159	4166	29.94 (29.88 to 30.00)
	IMD=9	230	6772.394	38.87 (38.82 to 38.93)	472	8548	61.63 (61.56 to 61.70)	72	3226.215	18.56 (18.51 to 18.60)	157	3714	32.94 (32.87 to 33.00)
	Most Deprivation	272	7641.766	37.39 (37.33 to 37.44)	580	9629	62.26 (62.19 to 62.34)	66	3921.418	14.92 (14.88 to 14.96)	148	4476	27.91 (27.85 to 27.97)
2015	Least Deprivation	580	15225.52	38.60 (38.54 to 38.65)	962	18219	51.71 (51.64 to 51.77)	218	9829.851	20.10 (20.05 to 20.15)	374	10880	30.65 (30.59 to 30.71)
	IMD=2	652	18372	34.04 (33.99 to 34.09)	1095	22021	46.58 (46.52 to 46.65)	304	13110.76	18.25 (18.20 to 18.29)	579	14729	29.95 (29.89 to 30.01)
	IMD=3	488	14971.36	31.20 (31.15 to 31.25)	865	17870	45.71 (45.64 to 45.77)	224	10089.68	18.17 (18.12 to 18.21)	420	11335	28.85 (28.79 to 28.91)
	IMD=4	288	10625.18	29.46 (29.41 to 29.51)	484	12688	40.93 (40.88 to 40.99)	109	5448.942	16.60 (16.55 to 16.64)	229	6145	29.55 (29.50 to 29.61)
	IMD=5	288	9810.242	30.29 (30.24 to 30.34)	522	11805	44.96 (44.90 to 45.02)	142	5573.539	20.62 (20.58 to 20.67)	273	6344	33.77 (33.70 to 33.83)
	IMD=6	304	10724.87	34.62 (34.56 to 34.67)	551	12851	50.13 (50.06 to 50.19)	136	4936.843	21.57 (21.52 to 21.62)	264	5654	35.14 (35.07 to 35.20)
	IMD=7	205	7931.756	32.35 (32.30 to 32.40)	362	9727	44.86 (44.80 to 44.92)	88	3576.75	20.66 (20.61 to 20.71)	145	4078	28.35 (28.29 to 28.41)
	IMD=8	254	6913.604	39.33 (39.28 to 39.39)	474	8567	58.15 (58.08 to 58.22)	82	3638.064	18.67 (18.62 to 18.72)	195	4192	36.71 (36.64 to 36.78)
	IMD=9	254	7133.602	41.50 (41.45 to 41.56)	481	8892	61.18 (61.11 to 61.25)	68	3315.003	17.32 (17.27 to 17.36)	156	3813	32.75 (32.69 to 32.81)
	Most Deprivation	297	8190.116	38.29 (38.23 to 38.35)	583	10111	59.38 (59.31 to 59.45)	78	4124.684	15.99 (15.94 to 16.03)	146	4647	26.16 (26.10 to 26.22)
2016	Least Deprivation	520	15563.35	32.57 (32.52 to 32.62)	903	18566	47.07 (47.00 to 47.13)	217	10201.97	19.36 (19.31 to 19.41)	400	11279	31.45 (31.39 to 31.51)
	IMD=2	625	18754.76	32.08 (32.02 to 32.13)	1084	22300	46.19 (46.12 to 46.25)	305	13335.76	18.06 (18.01 to 18.11)	553	14975	27.98 (27.92 to 28.04)
	IMD=3	491	15413.48	31.40 (31.35 to 31.45)	838	18284	43.72 (43.66 to 43.78)	247	10364.15	19.14 (19.10 to 19.19)	447	11650	29.80 (29.74 to 29.86)
	IMD=4	288	10733.53	29.80 (29.75 to 29.85)	485	12771	41.16 (41.10 to 41.22)	131	5493.312	19.78 (19.73 to 19.82)	253	6219	31.98 (31.92 to 32.04)
	IMD=5	319	10068.39	32.82 (32.77 to 32.87)	547	11989	46.56 (46.49 to 46.62)	126	5707.981	18.02 (17.98 to 18.07)	254	6465	30.59 (30.53 to 30.65)
	IMD=6	307	10951.78	34.14 (34.08 to 34.19)	546	13141	48.96 (48.89 to 49.02)	127	5015.735	20.01 (19.96 to 20.06)	250	5733	33.00 (32.94 to 33.07)
	IMD=7	219	8154.579	32.78 (32.73 to 32.83)	384	9886	47.06 (47.00 to 47.12)	83	3676.679	18.57 (18.52 to 18.62)	158	4157	30.47 (30.41 to 30.53)
	IMD=8	278	7105.495	42.06 (42.00 to 42.12)	479	8721	57.80 (57.73 to 57.87)	98	3689.949	22.10 (22.04 to 22.15)	209	4230	39.25 (39.18 to 39.32)

	IMD=9	230	7329.867	35.74 (35.69 to 35.80)	470	9001	57.89 (57.82 to 57.96)	74	3445.55	17.90 (17.85 to 17.95)	167	3916	33.47 (33.41 to 33.53)
	Most Deprivation	337	8865.698	40.52 (40.46 to 40.58)	593	10737	56.76 (56.69 to 56.83)	113	4375.548	23.27 (23.22 to 23.32)	192	4882	33.50 (33.44 to 33.57)
2017	Least Deprivation	517	16064.52	32.57 (32.51 to 32.62)	839	19003	43.67 (43.61 to 43.73)	221	10447.01	19.35 (19.30 to 19.40)	433	11634	32.59 (32.53 to 32.66)
	IMD=2	672	19189.59	33.26 (33.21 to 33.32)	1069	22707	44.17 (44.11 to 44.23)	344	13567.18	19.95 (19.90 to 20.00)	585	15317	28.79 (28.73 to 28.85)
	IMD=3	521	15871.73	31.68 (31.63 to 31.73)	831	18715	42.18 (42.12 to 42.24)	238	10625.69	18.00 (17.96 to 18.05)	452	11939	29.25 (29.19 to 29.30)
	IMD=4	306	10929.27	30.73 (30.68 to 30.78)	507	12847	42.89 (42.83 to 42.95)	104	5625.971	15.16 (15.11 to 15.20)	220	6313	27.57 (27.52 to 27.63)
	IMD=5	307	10255.42	31.00 (30.95 to 31.05)	522	12163	44.12 (44.06 to 44.18)	137	5784.471	18.49 (18.45 to 18.54)	274	6567	31.80 (31.74 to 31.87)
	IMD=6	295	11166.54	32.47 (32.42 to 32.52)	509	13198	45.61 (45.55 to 45.67)	114	5056.613	17.78 (17.74 to 17.83)	223	5793	29.11 (29.05 to 29.17)
	IMD=7	242	8455.058	35.01 (34.95 to 35.06)	397	10062	47.64 (47.58 to 47.70)	97	3728.164	21.37 (21.32 to 21.42)	161	4220	30.52 (30.46 to 30.58)
	IMD=8	258	7314.875	37.79 (37.74 to 37.85)	471	8881	55.35 (55.28 to 55.42)	103	3715.258	22.57 (22.52 to 22.62)	205	4265	37.92 (37.85 to 37.99)
	IMD=9	254	7659.039	36.74 (36.68 to 36.79)	471	9322	55.59 (55.53 to 55.66)	84	3530.073	20.22 (20.17 to 20.27)	167	4026	33.20 (33.13 to 33.26)
	Most Deprivation	313	9241.262	36.13 (36.07 to 36.18)	596	11261	55.13 (55.06 to 55.20)	106	4492.871	21.20 (21.15 to 21.25)	197	5094	33.24 (33.18 to 33.30)
2018	Least Deprivation	488	16727.78	28.61 (28.57 to 28.66)	803	19614	39.45 (39.39 to 39.51)	247	10880.76	19.44 (19.39 to 19.48)	449	12120	31.46 (31.40 to 31.52)
	IMD=2	610	19900.7	29.83 (29.78 to 29.88)	1003	23428	40.37 (40.32 to 40.43)	352	13893.77	19.61 (19.57 to 19.66)	642	15723	30.30 (30.24 to 30.36)
	IMD=3	546	17810.17	29.38 (29.33 to 29.43)	890	20702	40.88 (40.82 to 40.94)	314	11946	20.83 (20.78 to 20.88)	571	13411	32.38 (32.32 to 32.44)
	IMD=4	303	11417.55	28.98 (28.93 to 29.03)	509	13354	41.37 (41.31 to 41.43)	152	5840.608	21.38 (21.33 to 21.43)	247	6552	30.00 (29.94 to 30.06)
	IMD=5	306	10938.92	28.76 (28.71 to 28.80)	504	12794	39.97 (39.91 to 40.03)	138	6109.484	18.19 (18.15 to 18.24)	297	6941	32.67 (32.61 to 32.73)
	IMD=6	279	11527.55	30.15 (30.10 to 30.20)	470	13487	41.91 (41.85 to 41.97)	150	5167.272	22.73 (22.68 to 22.79)	272	5944	34.49 (34.42 to 34.55)
	IMD=7	227	8973.884	30.41 (30.36 to 30.46)	353	10624	39.34 (39.29 to 39.40)	95	3939.565	19.97 (19.92 to 20.02)	184	4449	32.94 (32.88 to 33.00)
	IMD=8	284	7457.851	40.89 (40.83 to 40.95)	491	9055	57.34 (57.28 to 57.41)	101	3716.865	22.63 (22.58 to 22.69)	208	4305	38.45 (38.38 to 38.52)
	IMD=9	274	7951.699	38.93 (38.88 to 38.99)	491	9649	56.62 (56.55 to 56.69)	89	3601.002	21.32 (21.27 to 21.37)	178	4117	35.50 (35.43 to 35.56)
	Most Deprivation	358	9630.056	40.00 (39.94 to 40.06)	633	11610	57.08 (57.02 to 57.15)	104	4611.869	19.76 (19.71 to 19.81)	201	5206	32.90 (32.84 to 32.96)
2019	Least Deprivation	449	15272.09	29.33 (29.29 to 29.38)	706	20189	34.00 (33.95 to 34.05)	185	9854.042	16.46 (16.41 to 16.50)	369	12518	24.80 (24.74 to 24.85)
	IMD=2	536	17921.37	28.48 (28.43 to 28.53)	880	24903	32.97 (32.91 to 33.02)	294	12560.18	17.73 (17.68 to 17.78)	544	16766	23.66 (23.60 to 23.71)
	IMD=3	419	14880.08	27.32 (27.27 to 27.37)	692	21396	30.81 (30.76 to 30.87)	195	9850.119	15.60 (15.56 to 15.65)	433	13819	23.61 (23.55 to 23.66)
	IMD=4	228	9872.865	26.45 (26.40 to 26.49)	392	14021	30.83 (30.78 to 30.88)	126	4605.363	22.28 (22.23 to 22.33)	258	6676	30.11 (30.05 to 30.18)
	IMD=5	312	10447.73	31.58 (31.53 to 31.63)	500	13857	36.87 (36.81 to 36.92)	139	5765.109	19.31 (19.26 to 19.36)	273	7565	27.43 (27.38 to 27.49)
	IMD=6	283	11949.27	30.41 (30.36 to 30.46)	480	14284	41.00 (40.94 to 41.05)	123	5028.876	19.41 (19.36 to 19.46)	247	6009	31.26 (31.20 to 31.32)
	IMD=7	214	8347.236	32.01 (31.95 to 32.06)	354	11222	37.97 (37.92 to 38.03)	70	3513.717	16.16 (16.12 to 16.21)	156	4668	26.14 (26.08 to 26.20)
	IMD=8	186	6115.956	33.63 (33.58 to 33.68)	333	9262	38.03 (37.98 to 38.09)	83	2861.101	24.98 (24.93 to 25.04)	168	4347	30.62 (30.56 to 30.68)

	IMD=9	232	8098.546	33.28 (33.22 to 33.33)	451	10075	50.59 (50.52 to 50.65)	86	3534.16	20.56 (20.51 to 20.61)	173	4200	33.00 (32.93 to 33.06)
	Most Deprivation	314	9952.566	34.10 (34.05 to 34.16)	612	12039	53.32 (53.26 to 53.39)	116	4674.946	21.70 (21.65 to 21.75)	216	5353	34.32 (34.26 to 34.39)
**West Midlands**													
2004	Least Deprivation	2091	62269.13	32.35 (32.30 to 32.40)	3040	70532	40.77 (40.71 to 40.83)	967	40476.68	19.46 (19.41 to 19.51)	1528	44337	27.19 (27.13 to 27.24)
	IMD=2	2604	76414.59	33.23 (33.18 to 33.28)	3718	86408	41.61 (41.55 to 41.67)	977	47065.09	16.81 (16.76 to 16.85)	1705	51542	25.92 (25.86 to 25.97)
	IMD=3	2484	75365.94	31.94 (31.89 to 31.99)	3664	85475	41.00 (40.94 to 41.06)	1073	47614.99	17.55 (17.51 to 17.60)	1882	52651	26.97 (26.91 to 27.02)
	IMD=4	2769	75379.07	35.70 (35.65 to 35.76)	4038	86319	45.14 (45.08 to 45.20)	1039	46849.31	17.32 (17.27 to 17.36)	1884	51954	27.38 (27.32 to 27.43)
	IMD=5	2540	75282.56	32.78 (32.73 to 32.83)	3756	85312	42.45 (42.39 to 42.51)	1056	46712.46	17.55 (17.50 to 17.60)	1881	51783	27.36 (27.30 to 27.42)
	IMD=6	2957	83271.04	35.04 (34.98 to 35.09)	4312	94522	44.62 (44.56 to 44.68)	1040	49451.82	16.45 (16.41 to 16.49)	1952	54792	27.02 (26.97 to 27.08)
	IMD=7	2375	62001.51	37.80 (37.75 to 37.86)	3549	71143	48.88 (48.82 to 48.95)	908	35730.82	19.80 (19.75 to 19.84)	1597	39938	30.16 (30.10 to 30.22)
	IMD=8	1948	57994.12	33.53 (33.47 to 33.58)	2948	65858	44.44 (44.38 to 44.50)	742	31773.93	17.92 (17.88 to 17.97)	1236	35121	26.50 (26.44 to 26.56)
	IMD=9	2407	69824.94	34.97 (34.91 to 35.02)	3595	79539	45.73 (45.67 to 45.79)	781	36347.06	16.71 (16.67 to 16.76)	1456	40372	27.39 (27.33 to 27.45)
	Most Deprivation	2108	66426.52	32.78 (32.72 to 32.83)	3107	74801	42.73 (42.67 to 42.79)	654	32577.31	15.78 (15.74 to 15.83)	1124	36131	23.83 (23.78 to 23.89)
2005	Least Deprivation	2129	62632.01	32.68 (32.63 to 32.73)	3182	72059	41.80 (41.74 to 41.86)	862	41092.08	17.10 (17.06 to 17.15)	1548	45447	26.87 (26.82 to 26.93)
	IMD=2	2441	77070.93	30.75 (30.70 to 30.80)	3798	88455	41.26 (41.20 to 41.32)	919	48036.19	15.49 (15.45 to 15.54)	1743	52994	25.75 (25.70 to 25.81)
	IMD=3	2517	75445.03	32.21 (32.15 to 32.26)	3881	86855	42.73 (42.67 to 42.79)	1046	48096.12	17.15 (17.10 to 17.19)	1980	53657	27.93 (27.87 to 27.98)
	IMD=4	2751	75804.75	35.53 (35.48 to 35.59)	4225	88048	46.37 (46.31 to 46.43)	975	47671.28	16.06 (16.02 to 16.11)	1919	53222	27.18 (27.13 to 27.24)
	IMD=5	2521	75581.57	32.59 (32.53 to 32.64)	3903	86906	43.42 (43.36 to 43.48)	1081	47411.61	17.86 (17.82 to 17.91)	2038	52946	29.13 (29.07 to 29.19)
	IMD=6	2898	83436.88	34.25 (34.20 to 34.31)	4535	96225	45.93 (45.87 to 45.99)	1162	49968.13	18.28 (18.23 to 18.33)	2193	55758	29.88 (29.82 to 29.94)
	IMD=7	2416	62590.75	38.29 (38.24 to 38.35)	3803	73074	51.04 (50.97 to 51.10)	920	36565.09	19.74 (19.69 to 19.79)	1750	41178	32.07 (32.01 to 32.13)
	IMD=8	2107	57890.66	36.52 (36.47 to 36.58)	3238	66797	48.20 (48.14 to 48.27)	701	31885.19	16.95 (16.90 to 17.00)	1270	35563	26.87 (26.81 to 26.92)
	IMD=9	2390	69932.93	34.92 (34.87 to 34.97)	3867	81083	48.44 (48.38 to 48.50)	792	36670.85	16.91 (16.86 to 16.95)	1553	41015	28.82 (28.77 to 28.88)
	Most Deprivation	2159	67541.21	32.98 (32.93 to 33.03)	3418	77371	45.43 (45.37 to 45.49)	715	32896.78	17.18 (17.13 to 17.22)	1343	36773	28.15 (28.09 to 28.21)
2006	Least Deprivation	2155	63305.1	32.76 (32.71 to 32.81)	3362	73538	43.34 (43.28 to 43.40)	844	41981.51	16.50 (16.45 to 16.54)	1562	46689	26.40 (26.35 to 26.46)
	IMD=2	2608	78498.6	32.36 (32.31 to 32.41)	4093	90797	43.37 (43.31 to 43.43)	909	49507.31	14.81 (14.77 to 14.85)	1747	54731	24.96 (24.91 to 25.02)
	IMD=3	2531	75761.74	32.49 (32.44 to 32.54)	3969	88124	43.15 (43.09 to 43.21)	955	48976.64	15.29 (15.25 to 15.33)	1906	54741	26.33 (26.28 to 26.39)
	IMD=4	2695	76349.29	34.22 (34.16 to 34.27)	4354	89676	46.51 (46.45 to 46.58)	945	48656.94	15.29 (15.24 to 15.33)	1881	54441	26.12 (26.06 to 26.17)
	IMD=5	2541	76052.45	32.68 (32.62 to 32.73)	4056	88457	44.19 (44.13 to 44.25)	921	48447.12	14.90 (14.86 to 14.94)	1905	54248	26.49 (26.43 to 26.54)
	IMD=6	2900	83952.66	33.91 (33.85 to 33.96)	4649	98040	46.12 (46.06 to 46.19)	1127	50762.01	17.54 (17.49 to 17.58)	2219	56982	29.70 (29.64 to 29.76)
	IMD=7	2289	62848.17	36.23 (36.17 to 36.28)	3838	74317	50.65 (50.58 to 50.71)	839	37209.94	17.89 (17.85 to 17.94)	1699	42085	30.73 (30.67 to 30.79)

	IMD=8	2112	58033.19	36.75 (36.69 to 36.80)	3390	68010	49.80 (49.74 to 49.87)	670	32140.89	16.37 (16.33 to 16.42)	1297	36022	27.42 (27.36 to 27.48)
	IMD=9	2499	69825.82	36.70 (36.64 to 36.75)	4100	82054	50.79 (50.73 to 50.86)	800	36764.66	17.14 (17.09 to 17.18)	1587	41358	29.39 (29.33 to 29.44)
	Most Deprivation	2279	67928.28	35.15 (35.09 to 35.20)	3669	79011	48.23 (48.17 to 48.30)	638	33075.79	15.67 (15.62 to 15.71)	1250	37126	26.51 (26.46 to 26.57)
2007	Least Deprivation	2107	63824.88	31.99 (31.94 to 32.04)	3299	74567	42.06 (42.00 to 42.12)	775	42794.06	14.77 (14.73 to 14.82)	1467	47628	24.15 (24.09 to 24.20)
	IMD=2	2566	79458.05	31.16 (31.11 to 31.21)	4053	92542	41.87 (41.81 to 41.93)	964	50531.39	15.32 (15.28 to 15.37)	1813	56046	25.11 (25.06 to 25.17)
	IMD=3	2572	75839.88	32.84 (32.79 to 32.90)	4137	88933	44.40 (44.34 to 44.46)	927	49612.68	14.74 (14.70 to 14.78)	1920	55557	26.11 (26.06 to 26.17)
	IMD=4	2754	76576.11	35.11 (35.06 to 35.17)	4570	90583	48.58 (48.52 to 48.65)	999	49430.64	15.87 (15.83 to 15.92)	1934	55352	26.39 (26.33 to 26.45)
	IMD=5	2522	76751.63	31.95 (31.90 to 32.01)	4143	89902	44.33 (44.27 to 44.39)	1037	49432.17	16.59 (16.54 to 16.63)	2031	55469	27.79 (27.73 to 27.84)
	IMD=6	2961	84319.17	34.54 (34.48 to 34.59)	4856	99400	47.50 (47.43 to 47.56)	1032	51636.54	15.88 (15.84 to 15.93)	2113	58138	27.80 (27.74 to 27.85)
	IMD=7	2333	63079.97	36.81 (36.75 to 36.86)	3895	75262	50.79 (50.72 to 50.85)	844	37782.66	17.71 (17.66 to 17.75)	1676	42873	29.66 (29.60 to 29.72)
	IMD=8	2166	58443.27	37.25 (37.19 to 37.30)	3568	69220	51.42 (51.35 to 51.48)	689	32599.92	16.58 (16.53 to 16.62)	1304	36677	27.13 (27.07 to 27.19)
	IMD=9	2482	70318.23	36.19 (36.13 to 36.24)	4203	83549	51.32 (51.25 to 51.38)	807	37154.8	17.29 (17.24 to 17.33)	1706	42007	31.20 (31.14 to 31.26)
	Most Deprivation	2396	68277.26	36.53 (36.47 to 36.58)	3966	80553	51.01 (50.95 to 51.08)	713	33505.14	17.28 (17.23 to 17.32)	1354	37767	28.26 (28.20 to 28.32)
2008	Least Deprivation	2202	64521.76	32.74 (32.69 to 32.79)	3513	75308	43.82 (43.76 to 43.88)	889	43725.25	16.41 (16.36 to 16.45)	1580	48450	25.38 (25.32 to 25.43)
	IMD=2	2693	80417.79	32.57 (32.52 to 32.63)	4226	93646	43.25 (43.19 to 43.31)	975	51796.44	15.05 (15.01 to 15.10)	1872	57156	25.39 (25.34 to 25.45)
	IMD=3	2489	76254.88	31.45 (31.40 to 31.50)	4141	89338	43.98 (43.92 to 44.04)	1028	50427.48	15.99 (15.95 to 16.04)	2017	56231	27.06 (27.00 to 27.12)
	IMD=4	2778	77514.39	35.02 (34.97 to 35.08)	4577	91452	48.16 (48.10 to 48.22)	1048	50535.69	16.28 (16.24 to 16.33)	2016	56270	27.03 (26.97 to 27.09)
	IMD=5	2621	77625.84	32.91 (32.86 to 32.97)	4230	90770	44.98 (44.92 to 45.04)	1034	50335.92	16.16 (16.12 to 16.20)	2089	56308	28.14 (28.09 to 28.20)
	IMD=6	2948	85229.23	34.19 (34.14 to 34.24)	4955	100436	47.99 (47.92 to 48.05)	1136	52647.37	17.10 (17.05 to 17.14)	2293	59048	29.65 (29.59 to 29.71)
	IMD=7	2398	63790.04	37.59 (37.53 to 37.65)	4024	76090	52.08 (52.02 to 52.15)	837	38525.62	17.35 (17.30 to 17.39)	1722	43570	30.14 (30.08 to 30.20)
	IMD=8	2172	58935.12	37.20 (37.15 to 37.26)	3690	70352	52.46 (52.40 to 52.53)	751	33138.92	18.02 (17.97 to 18.06)	1406	37234	29.03 (28.97 to 29.09)
	IMD=9	2694	71237.63	38.85 (38.80 to 38.91)	4492	84949	53.88 (53.81 to 53.95)	850	37697.78	18.08 (18.03 to 18.13)	1766	42641	32.04 (31.98 to 32.10)
	Most Deprivation	2387	68045.6	36.92 (36.87 to 36.98)	4120	81651	52.62 (52.55 to 52.69)	717	33585.57	17.48 (17.44 to 17.53)	1421	38200	29.47 (29.42 to 29.53)
2009	Least Deprivation	2305	65269.15	34.19 (34.13 to 34.24)	3583	76379	44.47 (44.41 to 44.53)	880	44731.18	15.77 (15.72 to 15.81)	1593	49648	24.82 (24.76 to 24.87)
	IMD=2	2804	81171.77	33.39 (33.33 to 33.44)	4490	94848	45.28 (45.22 to 45.34)	1013	53010.61	15.28 (15.23 to 15.32)	1886	58708	24.80 (24.75 to 24.85)
	IMD=3	2724	78701.04	33.46 (33.41 to 33.51)	4379	92364	45.04 (44.98 to 45.10)	1143	52571.83	16.93 (16.89 to 16.98)	2240	58771	28.55 (28.49 to 28.61)
	IMD=4	3007	78143.09	37.48 (37.42 to 37.53)	4886	92796	50.62 (50.55 to 50.68)	1109	51424.27	16.96 (16.92 to 17.01)	2144	57530	28.23 (28.18 to 28.29)
	IMD=5	2696	78374.23	33.46 (33.41 to 33.51)	4392	92061	45.85 (45.79 to 45.92)	1079	51261.83	16.57 (16.53 to 16.62)	2108	57453	27.87 (27.81 to 27.92)
	IMD=6	3244	85906.68	37.20 (37.15 to 37.26)	5374	101710	51.36 (51.29 to 51.42)	1157	53408.3	17.21 (17.16 to 17.25)	2376	60087	30.22 (30.16 to 30.28)
	IMD=7	2450	64274.01	37.99 (37.93 to 38.04)	4191	76816	53.68 (53.61 to 53.74)	919	39192.01	18.68 (18.63 to 18.73)	1813	44379	31.21 (31.15 to 31.27)

	IMD=8	2213	59021.69	38.02 (37.96 to 38.08)	3766	70661	53.31 (53.24 to 53.37)	792	33389.26	18.84 (18.79 to 18.88)	1507	37697	30.83 (30.77 to 30.89)
	IMD=9	2787	71214.65	40.53 (40.47 to 40.59)	4767	85853	56.67 (56.60 to 56.74)	887	37890.94	19.10 (19.05 to 19.15)	1906	43216	34.57 (34.50 to 34.63)
	Most Deprivation	2587	65997.61	41.40 (41.34 to 41.46)	4571	79600	59.92 (59.85 to 59.99)	753	33061	18.69 (18.65 to 18.74)	1534	37593	32.49 (32.42 to 32.55)
2010	Least Deprivation	2296	66309.47	33.45 (33.40 to 33.50)	3634	77747	44.36 (44.30 to 44.42)	868	45857.37	14.98 (14.94 to 15.02)	1594	50794	24.10 (24.05 to 24.16)
	IMD=2	2830	82877.89	33.21 (33.16 to 33.26)	4597	97153	45.40 (45.34 to 45.46)	996	54639.37	14.54 (14.50 to 14.59)	1918	60610	24.42 (24.37 to 24.47)
	IMD=3	2692	79681.74	32.65 (32.60 to 32.70)	4497	93832	45.38 (45.32 to 45.44)	1074	53674.75	15.68 (15.64 to 15.72)	2156	60104	26.94 (26.88 to 26.99)
	IMD=4	2889	78810.99	35.65 (35.60 to 35.71)	4787	94093	48.89 (48.82 to 48.95)	1012	52365.28	15.29 (15.25 to 15.34)	2033	58678	26.33 (26.27 to 26.38)
	IMD=5	2782	79235.37	34.57 (34.52 to 34.63)	4508	93485	46.59 (46.53 to 46.65)	1089	52204.47	16.45 (16.40 to 16.49)	2108	58574	27.34 (27.29 to 27.40)
	IMD=6	3083	86762.01	34.87 (34.81 to 34.92)	5219	103255	48.91 (48.84 to 48.97)	1171	54400.77	17.12 (17.07 to 17.16)	2388	61289	29.82 (29.76 to 29.88)
	IMD=7	2457	65299.21	37.67 (37.61 to 37.72)	4236	78361	53.27 (53.21 to 53.34)	918	40049.08	18.31 (18.26 to 18.35)	1846	45424	31.14 (31.08 to 31.20)
	IMD=8	2191	59761.89	37.00 (36.94 to 37.06)	3825	71952	53.16 (53.09 to 53.22)	760	34033.62	17.93 (17.88 to 17.97)	1538	38491	31.05 (30.99 to 31.11)
	IMD=9	2768	71436.15	39.96 (39.90 to 40.02)	4940	86596	58.30 (58.23 to 58.37)	855	38185.42	18.21 (18.17 to 18.26)	1866	43595	33.72 (33.66 to 33.78)
	Most Deprivation	2688	65866.08	42.69 (42.63 to 42.75)	4827	80302	62.34 (62.27 to 62.42)	799	33310.26	19.95 (19.91 to 20.00)	1640	38056	34.53 (34.47 to 34.60)
2011	Least Deprivation	2253	67088.64	32.49 (32.43 to 32.54)	3634	78905	43.66 (43.60 to 43.72)	937	46772.92	15.80 (15.76 to 15.85)	1668	51931	24.53 (24.47 to 24.58)
	IMD=2	2922	83568.08	34.15 (34.09 to 34.20)	4770	98269	46.78 (46.72 to 46.85)	1073	55700.36	15.31 (15.26 to 15.35)	1972	61761	24.51 (24.46 to 24.57)
	IMD=3	2817	80362.48	33.72 (33.67 to 33.77)	4640	94832	46.20 (46.14 to 46.26)	1053	54652.19	15.06 (15.02 to 15.11)	2101	61210	25.69 (25.64 to 25.75)
	IMD=4	2972	79453.74	36.60 (36.54 to 36.65)	4990	94859	50.39 (50.33 to 50.46)	1136	53201.48	16.82 (16.78 to 16.87)	2174	59608	27.66 (27.60 to 27.72)
	IMD=5	2769	79754.33	33.89 (33.84 to 33.94)	4446	94237	45.39 (45.32 to 45.45)	1077	53072.02	15.95 (15.91 to 16.00)	2159	59557	27.40 (27.34 to 27.45)
	IMD=6	3202	87646.45	35.99 (35.93 to 36.04)	5434	104521	50.54 (50.47 to 50.60)	1137	55276.89	16.37 (16.33 to 16.42)	2407	62315	29.60 (29.54 to 29.66)
	IMD=7	2565	65743.81	38.87 (38.82 to 38.93)	4452	79065	55.37 (55.31 to 55.44)	916	40586.71	18.13 (18.08 to 18.17)	1853	45993	30.92 (30.86 to 30.98)
	IMD=8	2419	60116.58	40.70 (40.64 to 40.76)	4140	72645	57.03 (56.96 to 57.10)	791	34355.39	18.45 (18.40 to 18.50)	1558	38975	31.07 (31.01 to 31.13)
	IMD=9	2848	70964.05	41.34 (41.28 to 41.40)	5134	86757	60.29 (60.22 to 60.36)	886	38264.07	19.03 (18.99 to 19.08)	1924	43777	34.79 (34.72 to 34.85)
	Most Deprivation	2628	65525.14	42.49 (42.43 to 42.55)	4875	80460	63.20 (63.13 to 63.28)	785	33408.36	19.60 (19.55 to 19.65)	1673	38241	35.26 (35.19 to 35.32)
2012	Least Deprivation	2366	68094.76	33.02 (32.96 to 33.07)	3757	79838	44.06 (44.00 to 44.12)	888	47734.22	14.59 (14.55 to 14.64)	1633	52795	23.40 (23.34 to 23.45)
	IMD=2	2977	84599.73	34.03 (33.98 to 34.09)	4898	99391	47.15 (47.08 to 47.21)	987	57062.07	13.67 (13.63 to 13.71)	1870	63047	22.64 (22.59 to 22.70)
	IMD=3	2829	81339.49	33.28 (33.23 to 33.33)	4714	95787	46.43 (46.37 to 46.49)	1052	55694.88	14.69 (14.65 to 14.73)	2057	62123	24.58 (24.53 to 24.63)
	IMD=4	3008	80085.08	36.58 (36.52 to 36.63)	5119	95591	51.28 (51.22 to 51.35)	1123	53969.15	16.34 (16.30 to 16.38)	2162	60456	26.99 (26.94 to 27.05)
	IMD=5	2903	80509.75	35.29 (35.24 to 35.35)	4763	95013	48.11 (48.05 to 48.18)	1036	54065.13	15.04 (14.99 to 15.08)	2100	60461	26.17 (26.11 to 26.23)
	IMD=6	3287	88510.37	36.86 (36.80 to 36.92)	5679	105430	52.57 (52.51 to 52.64)	1171	56275.14	16.52 (16.47 to 16.56)	2495	63277	30.16 (30.10 to 30.22)
	IMD=7	2653	66246.73	39.91 (39.86 to 39.97)	4643	79648	57.11 (57.04 to 57.18)	913	41324.59	17.66 (17.61 to 17.71)	1860	46644	30.56 (30.50 to 30.62)

	IMD=8	2414	60978.94	40.12 (40.07 to 40.18)	4274	73549	58.07 (58.00 to 58.14)	789	34940.38	18.28 (18.23 to 18.33)	1576	39661	31.02 (30.96 to 31.08)
	IMD=9	2952	71393.56	42.62 (42.56 to 42.68)	5366	86940	62.76 (62.69 to 62.83)	870	38746.47	18.46 (18.42 to 18.51)	1911	44207	34.20 (34.13 to 34.26)
	Most Deprivation	2886	65965.69	46.03 (45.96 to 46.09)	5304	80960	67.97 (67.89 to 68.04)	799	33808.98	19.73 (19.68 to 19.78)	1733	38657	36.18 (36.12 to 36.25)
2013	Least Deprivation	2282	68267.12	32.47 (32.41 to 32.52)	3631	80421	43.10 (43.04 to 43.16)	1003	48141.29	16.24 (16.20 to 16.28)	1766	53488	24.75 (24.70 to 24.81)
	IMD=2	2911	84658.28	33.51 (33.46 to 33.56)	4776	100169	45.77 (45.71 to 45.83)	1174	57660.67	16.06 (16.02 to 16.10)	2008	64044	23.83 (23.78 to 23.88)
	IMD=3	2759	81087.28	32.83 (32.77 to 32.88)	4540	96276	44.78 (44.71 to 44.84)	1110	56115.41	15.34 (15.30 to 15.38)	2088	62872	24.67 (24.62 to 24.73)
	IMD=4	2962	79933.95	36.12 (36.07 to 36.18)	5011	95951	50.09 (50.03 to 50.16)	1119	54198.85	16.21 (16.16 to 16.25)	2131	61000	26.29 (26.23 to 26.34)
	IMD=5	2790	80444.82	33.84 (33.79 to 33.89)	4584	95494	46.01 (45.95 to 46.07)	1168	54437.85	16.78 (16.73 to 16.82)	2196	61135	27.05 (26.99 to 27.10)
	IMD=6	3247	88358.75	36.50 (36.44 to 36.55)	5573	105949	51.46 (51.39 to 51.52)	1254	56584.9	17.60 (17.55 to 17.64)	2486	63994	29.66 (29.61 to 29.72)
	IMD=7	2638	66466.25	39.65 (39.59 to 39.70)	4554	80469	55.72 (55.65 to 55.78)	955	41653.23	18.24 (18.20 to 18.29)	1847	47247	29.91 (29.85 to 29.97)
	IMD=8	2340	60886.87	39.08 (39.02 to 39.13)	4143	74023	56.16 (56.09 to 56.23)	826	35032.65	18.99 (18.94 to 19.04)	1557	39929	30.51 (30.45 to 30.58)
	IMD=9	2978	71132.66	43.23 (43.17 to 43.29)	5401	87471	62.81 (62.74 to 62.88)	960	38883.2	20.38 (20.33 to 20.43)	1888	44524	33.63 (33.57 to 33.70)
	Most Deprivation	2837	65485.45	45.73 (45.67 to 45.80)	5424	81399	69.16 (69.09 to 69.24)	878	33649.97	22.08 (22.03 to 22.13)	1722	38853	35.97 (35.90 to 36.03)
2014	Least Deprivation	2371	68376.77	33.04 (32.99 to 33.09)	3770	80547	44.18 (44.12 to 44.24)	1074	48414.77	17.23 (17.19 to 17.28)	1833	53922	25.36 (25.30 to 25.41)
	IMD=2	3044	84839.96	34.45 (34.40 to 34.50)	4876	100388	46.25 (46.19 to 46.31)	1251	58273.57	16.89 (16.84 to 16.93)	2145	64771	25.17 (25.12 to 25.23)
	IMD=3	2914	81217	34.34 (34.29 to 34.40)	4640	96178	45.66 (45.60 to 45.72)	1268	56500.41	17.38 (17.34 to 17.43)	2218	63119	26.09 (26.04 to 26.15)
	IMD=4	2964	80377.57	35.60 (35.55 to 35.66)	4997	96358	49.44 (49.37 to 49.50)	1223	54816.62	17.55 (17.50 to 17.59)	2233	61632	27.36 (27.30 to 27.42)
	IMD=5	2843	80906.63	34.20 (34.14 to 34.25)	4673	95854	46.82 (46.76 to 46.88)	1176	54956.58	16.71 (16.66 to 16.75)	2097	61670	25.52 (25.46 to 25.57)
	IMD=6	3320	89146.35	36.85 (36.80 to 36.91)	5661	106678	51.84 (51.78 to 51.91)	1323	57424.82	18.38 (18.34 to 18.43)	2462	64839	29.09 (29.03 to 29.15)
	IMD=7	2614	66736.47	39.16 (39.11 to 39.22)	4509	80845	54.95 (54.88 to 55.02)	1050	42121.98	19.85 (19.81 to 19.90)	1958	47799	31.40 (31.34 to 31.46)
	IMD=8	2349	61256.34	38.90 (38.85 to 38.96)	4134	74505	55.65 (55.58 to 55.72)	874	35411.5	19.92 (19.88 to 19.97)	1638	40312	31.81 (31.75 to 31.87)
	IMD=9	2829	71838.43	40.73 (40.67 to 40.79)	5196	88365	59.89 (59.82 to 59.96)	1042	39295.01	22.01 (21.95 to 22.06)	2011	45076	35.58 (35.51 to 35.64)
	Most Deprivation	2895	66451.71	45.80 (45.74 to 45.86)	5508	82382	69.06 (68.98 to 69.13)	890	34090.87	22.12 (22.07 to 22.18)	1801	39276	37.42 (37.35 to 37.49)
2015	Least Deprivation	2369	68955.04	32.81 (32.76 to 32.87)	3739	80960	43.38 (43.32 to 43.44)	1131	48984.72	17.89 (17.85 to 17.94)	1932	54540	26.32 (26.26 to 26.38)
	IMD=2	3019	85545.72	33.98 (33.93 to 34.04)	4894	101140	45.92 (45.86 to 45.98)	1350	59003.87	17.98 (17.93 to 18.02)	2316	65724	26.70 (26.64 to 26.76)
	IMD=3	2793	81876.41	33.01 (32.96 to 33.06)	4507	96720	44.39 (44.33 to 44.45)	1312	57049.11	17.81 (17.76 to 17.85)	2298	63865	26.72 (26.66 to 26.78)
	IMD=4	2887	80808.04	34.79 (34.73 to 34.84)	4874	96643	48.29 (48.23 to 48.36)	1319	55361.09	18.72 (18.67 to 18.77)	2316	62306	28.10 (28.04 to 28.16)
	IMD=5	2826	81675.37	33.67 (33.62 to 33.73)	4678	96678	46.32 (46.26 to 46.38)	1314	55650.6	18.40 (18.35 to 18.45)	2345	62497	28.16 (28.10 to 28.22)
	IMD=6	3233	89928.97	35.61 (35.55 to 35.66)	5450	107718	49.52 (49.45 to 49.58)	1399	57996.78	19.23 (19.18 to 19.28)	2629	65682	30.73 (30.67 to 30.79)
	IMD=7	2747	67591.71	40.72 (40.66 to 40.77)	4637	81781	55.98 (55.91 to 56.05)	1136	42588.59	21.34 (21.29 to 21.39)	2109	48462	33.43 (33.37 to 33.50)

	IMD=8	2337	62393.7	38.11 (38.05 to 38.16)	4099	75449	54.55 (54.48 to 54.61)	862	35963.6	19.56 (19.51 to 19.61)	1627	40910	31.47 (31.41 to 31.53)
	IMD=9	2945	74083.7	40.84 (40.78 to 40.89)	5265	90346	59.15 (59.08 to 59.22)	1049	40046.65	21.66 (21.61 to 21.72)	2032	45989	35.24 (35.18 to 35.31)
	Most Deprivation	2807	68461.55	42.84 (42.78 to 42.90)	5450	84683	66.36 (66.29 to 66.44)	915	34808.47	22.44 (22.39 to 22.49)	1872	40195	38.20 (38.13 to 38.27)
2016	Least Deprivation	2268	70184.89	30.82 (30.77 to 30.87)	3570	82007	40.96 (40.90 to 41.02)	1180	49683.23	18.31 (18.26 to 18.36)	1993	55459	26.62 (26.56 to 26.67)
	IMD=2	2892	86984.97	32.02 (31.97 to 32.07)	4692	102291	43.66 (43.60 to 43.72)	1399	59897.69	18.29 (18.25 to 18.34)	2383	66816	26.96 (26.90 to 27.02)
	IMD=3	2775	83200.89	32.12 (32.07 to 32.17)	4442	97818	43.21 (43.15 to 43.27)	1338	57709.04	17.89 (17.84 to 17.94)	2353	64669	27.02 (26.96 to 27.08)
	IMD=4	2860	82318.96	33.63 (33.57 to 33.68)	4660	97763	45.39 (45.33 to 45.45)	1382	56167.18	19.32 (19.27 to 19.37)	2416	63136	28.96 (28.90 to 29.02)
	IMD=5	2747	82970.8	32.04 (31.98 to 32.09)	4516	97717	44.32 (44.26 to 44.38)	1374	56333.4	18.96 (18.91 to 19.00)	2386	63421	28.23 (28.17 to 28.29)
	IMD=6	3333	91524.16	35.98 (35.93 to 36.04)	5517	109004	49.41 (49.34 to 49.47)	1443	58905.08	19.52 (19.47 to 19.57)	2727	66698	31.42 (31.36 to 31.48)
	IMD=7	2527	69000.09	36.73 (36.68 to 36.79)	4376	82987	52.15 (52.08 to 52.21)	1065	43285.9	19.68 (19.63 to 19.73)	2081	49211	32.48 (32.42 to 32.54)
	IMD=8	2338	63923.93	37.26 (37.20 to 37.31)	4053	76799	53.04 (52.98 to 53.11)	990	36605.01	22.09 (22.03 to 22.14)	1774	41610	33.63 (33.57 to 33.70)
	IMD=9	2783	76388.58	37.85 (37.80 to 37.91)	5163	92728	56.85 (56.78 to 56.92)	1127	40876.35	22.83 (22.78 to 22.88)	2185	46932	37.24 (37.17 to 37.30)
	Most Deprivation	2782	70772.26	41.38 (41.32 to 41.43)	5253	87068	62.48 (62.41 to 62.55)	948	35587.16	22.88 (22.83 to 22.93)	1901	41039	38.18 (38.11 to 38.25)
2017	Least Deprivation	2305	71043.14	30.39 (30.34 to 30.44)	3547	82997	39.80 (39.74 to 39.86)	1170	50098.47	17.91 (17.86 to 17.95)	2011	56212	26.32 (26.27 to 26.38)
	IMD=2	2896	88013.64	31.87 (31.81 to 31.92)	4656	103606	43.08 (43.02 to 43.14)	1398	60563.35	17.99 (17.94 to 18.03)	2419	67956	26.76 (26.70 to 26.81)
	IMD=3	2572	84338.2	29.56 (29.51 to 29.61)	4181	99225	40.29 (40.23 to 40.35)	1417	58050.31	18.86 (18.82 to 18.91)	2415	65523	27.43 (27.37 to 27.49)
	IMD=4	2664	83574.22	31.00 (30.95 to 31.05)	4443	99110	43.17 (43.11 to 43.22)	1381	56852.92	19.03 (18.98 to 19.08)	2402	64275	28.23 (28.17 to 28.28)
	IMD=5	2705	83666.7	31.40 (31.35 to 31.45)	4391	98751	42.50 (42.44 to 42.56)	1387	56799.44	18.93 (18.88 to 18.97)	2440	64302	28.38 (28.32 to 28.44)
	IMD=6	3129	93009.5	33.13 (33.08 to 33.19)	5245	110976	46.01 (45.95 to 46.07)	1489	59685.17	19.81 (19.76 to 19.86)	2775	67992	31.30 (31.24 to 31.36)
	IMD=7	2492	69892.08	35.56 (35.51 to 35.62)	4257	84257	49.93 (49.87 to 50.00)	1181	43579.54	21.63 (21.57 to 21.68)	2212	49970	34.03 (33.96 to 34.09)
	IMD=8	2212	64978.42	34.70 (34.64 to 34.75)	3876	78246	49.77 (49.71 to 49.84)	970	36872.2	21.51 (21.46 to 21.56)	1783	42329	33.32 (33.25 to 33.38)
	IMD=9	2761	78381.14	36.55 (36.50 to 36.61)	4983	95250	53.45 (53.38 to 53.52)	1066	41381.36	21.33 (21.28 to 21.38)	2169	47926	36.37 (36.30 to 36.44)
	Most Deprivation	2769	73194.02	40.00 (39.94 to 40.06)	5220	89824	60.25 (60.18 to 60.32)	1044	36213.86	24.46 (24.41 to 24.52)	2048	42047	40.00 (39.94 to 40.07)
2018	Least Deprivation	2149	73286.59	27.47 (27.43 to 27.52)	3362	85293	36.59 (36.53 to 36.64)	1259	51294.89	18.65 (18.61 to 18.70)	2028	57730	25.57 (25.52 to 25.63)
	IMD=2	2850	90868.88	29.98 (29.93 to 30.03)	4545	106533	40.30 (40.24 to 40.36)	1422	62212.79	17.73 (17.68 to 17.77)	2444	70049	26.09 (26.04 to 26.15)
	IMD=3	2520	87473.29	27.78 (27.73 to 27.82)	4040	102257	37.55 (37.49 to 37.60)	1505	59588.13	19.33 (19.29 to 19.38)	2600	67496	28.26 (28.20 to 28.32)
	IMD=4	2737	88075.16	30.08 (30.03 to 30.13)	4421	103641	40.82 (40.76 to 40.88)	1434	59363.19	18.79 (18.74 to 18.84)	2519	67303	28.11 (28.05 to 28.17)
	IMD=5	2554	86609.02	28.52 (28.47 to 28.57)	4151	101593	39.13 (39.07 to 39.18)	1450	58229.35	19.19 (19.15 to 19.24)	2484	66069	27.98 (27.92 to 28.03)
	IMD=6	3097	96709	31.63 (31.58 to 31.68)	5111	114571	43.69 (43.63 to 43.75)	1522	61550.22	19.51 (19.46 to 19.56)	2866	70231	31.12 (31.06 to 31.18)
	IMD=7	2402	72997.19	32.80 (32.75 to 32.86)	4095	87346	46.22 (46.15 to 46.28)	1208	44979.13	21.30 (21.25 to 21.35)	2272	51730	33.65 (33.58 to 33.71)

	IMD=8	2195	67709.79	33.16 (33.11 to 33.21)	3791	81191	47.02 (46.96 to 47.08)	1056	37853.14	22.98 (22.93 to 23.03)	1883	43632	34.13 (34.06 to 34.19)
	IMD=9	2917	82351.75	36.69 (36.64 to 36.75)	5103	99641	52.23 (52.16 to 52.29)	1172	43071.45	22.68 (22.63 to 22.73)	2254	49982	36.31 (36.24 to 36.38)
	Most Deprivation	2809	75389.91	39.43 (39.38 to 39.49)	5206	93587	57.75 (57.68 to 57.82)	991	36844.45	22.88 (22.83 to 22.93)	1985	43505	37.68 (37.61 to 37.75)
2019	Least Deprivation	2046	73911.32	25.88 (25.83 to 25.92)	3185	85961	34.31 (34.26 to 34.37)	1295	51353.81	18.90 (18.86 to 18.95)	2108	58229	26.10 (26.04 to 26.15)
	IMD=2	2807	91730.95	29.15 (29.10 to 29.20)	4394	107252	38.87 (38.81 to 38.93)	1577	62536.34	19.32 (19.27 to 19.37)	2653	70578	27.90 (27.84 to 27.96)
	IMD=3	2521	88842.42	27.23 (27.18 to 27.27)	3912	103209	35.86 (35.80 to 35.91)	1450	60049.02	18.35 (18.30 to 18.40)	2565	68115	27.58 (27.52 to 27.63)
	IMD=4	2711	89544.62	29.26 (29.21 to 29.31)	4375	104706	39.94 (39.88 to 40.00)	1462	59756.86	18.91 (18.87 to 18.96)	2601	67716	28.70 (28.64 to 28.76)
	IMD=5	2589	87373.15	28.63 (28.58 to 28.68)	4136	101941	38.55 (38.49 to 38.61)	1482	58201.59	19.52 (19.47 to 19.57)	2523	66143	28.25 (28.19 to 28.31)
	IMD=6	2987	97849.99	29.96 (29.91 to 30.01)	4951	115179	41.86 (41.80 to 41.92)	1605	61739.55	20.42 (20.37 to 20.47)	2843	70444	30.67 (30.61 to 30.73)
	IMD=7	2421	73414.24	32.78 (32.73 to 32.83)	4038	86905	45.66 (45.59 to 45.72)	1182	44849.2	20.76 (20.71 to 20.81)	2224	51494	32.85 (32.79 to 32.91)
	IMD=8	2185	67806.91	32.67 (32.62 to 32.73)	3681	80538	45.81 (45.75 to 45.87)	957	37888.18	20.65 (20.60 to 20.70)	1777	43572	32.21 (32.15 to 32.27)
	IMD=9	2690	81475.84	34.49 (34.43 to 34.54)	4701	97179	49.55 (49.48 to 49.61)	1075	42011.85	21.14 (21.09 to 21.19)	2104	48397	34.92 (34.86 to 34.99)
	Most Deprivation	2367	67400.35	36.66 (36.60 to 36.71)	4300	81366	54.43 (54.36 to 54.50)	835	33141.59	21.37 (21.32 to 21.42)	1645	38347	35.25 (35.18 to 35.31)
**East of England**													
2004	Least Deprivation	1899	54476.42	33.98 (33.93 to 34.03)	2916	63014	44.35 (44.29 to 44.41)	659	34809.42	15.91 (15.87 to 15.95)	1138	38242	24.13 (24.08 to 24.19)
	IMD=2	1013	28975.83	33.99 (33.93 to 34.04)	1565	33753	44.66 (44.60 to 44.72)	372	17534.96	17.60 (17.56 to 17.65)	662	19373	27.36 (27.30 to 27.42)
	IMD=3	1169	29657.49	38.68 (38.62 to 38.74)	1714	34098	48.90 (48.84 to 48.97)	410	18317.74	18.29 (18.25 to 18.34)	728	20240	28.20 (28.15 to 28.26)
	IMD=4	916	25275.45	35.76 (35.70 to 35.81)	1336	29035	44.98 (44.91 to 45.04)	363	15461.27	18.43 (18.39 to 18.48)	672	17192	29.38 (29.32 to 29.44)
	IMD=5	696	20088.93	33.85 (33.80 to 33.90)	967	22647	41.50 (41.44 to 41.56)	278	12158.6	18.20 (18.15 to 18.24)	434	13296	25.40 (25.34 to 25.45)
	IMD=6	679	20315.94	32.98 (32.92 to 33.03)	1056	23305	44.29 (44.23 to 44.35)	275	11858.68	18.23 (18.19 to 18.28)	506	13202	29.09 (29.03 to 29.15)
	IMD=7	718	23640	29.85 (29.80 to 29.90)	1188	27137	42.78 (42.72 to 42.84)	339	15287.76	16.21 (16.17 to 16.25)	743	17162	30.49 (30.43 to 30.55)
	IMD=8	162	5108.236	31.02 (30.97 to 31.07)	227	5733	38.43 (38.37 to 38.49)	45	2786.565	13.29 (13.25 to 13.33)	80	3025	20.72 (20.67 to 20.77)
	IMD=9	197	4872.561	40.59 (40.53 to 40.65)	293	5591	52.63 (52.56 to 52.70)	73	2889.903	19.85 (19.80 to 19.90)	131	3240	29.71 (29.65 to 29.77)
	Most Deprivation	65	1804.947	35.95 (35.89 to 36.00)	93	2031	45.40 (45.34 to 45.46)	28	911.4497	25.14 (25.09 to 25.20)	38	991	30.01 (29.95 to 30.07)
2005	Least Deprivation	1987	54839.9	35.09 (35.03 to 35.14)	3189	64144	47.36 (47.30 to 47.42)	656	35363.2	15.75 (15.71 to 15.80)	1208	39032	25.16 (25.11 to 25.22)
	IMD=2	1082	29134.95	36.80 (36.74 to 36.85)	1703	34290	48.18 (48.12 to 48.24)	373	17807.44	17.67 (17.62 to 17.71)	684	19798	28.02 (27.97 to 28.08)
	IMD=3	1173	29610.83	38.94 (38.88 to 38.99)	1789	34681	50.24 (50.18 to 50.31)	358	18534.79	15.60 (15.56 to 15.65)	729	20668	27.48 (27.42 to 27.53)
	IMD=4	1007	25186.54	38.83 (38.77 to 38.89)	1487	29457	48.59 (48.52 to 48.65)	347	15635.11	17.33 (17.28 to 17.37)	659	17526	28.20 (28.14 to 28.26)
	IMD=5	796	19969.59	39.32 (39.26 to 39.37)	1147	23021	48.64 (48.58 to 48.70)	318	12190.75	21.05 (21.00 to 21.10)	537	13532	30.93 (30.87 to 31.00)
	IMD=6	784	20372.22	38.24 (38.19 to 38.30)	1226	23750	50.68 (50.62 to 50.75)	251	11942.89	16.70 (16.66 to 16.75)	508	13406	29.06 (29.00 to 29.12)

	IMD=7	821	23704.91	33.76 (33.71 to 33.81)	1362	27539	48.03 (47.96 to 48.09)	340	15409.33	16.32 (16.28 to 16.37)	801	17431	32.76 (32.70 to 32.83)
	IMD=8	196	5260.493	36.97 (36.92 to 37.03)	287	6009	47.59 (47.53 to 47.65)	52	2915.554	14.37 (14.33 to 14.41)	102	3194	25.14 (25.08 to 25.19)
	IMD=9	198	4840.92	41.07 (41.01 to 41.13)	306	5669	53.87 (53.81 to 53.94)	84	2870.68	22.01 (21.96 to 22.06)	166	3256	37.29 (37.22 to 37.35)
	Most Deprivation	79	1755.455	45.33 (45.27 to 45.39)	108	2043	52.51 (52.45 to 52.58)	25	916.5695	22.05 (22.00 to 22.10)	44	1020	33.90 (33.84 to 33.97)
2006	Least Deprivation	2042	55588.57	35.24 (35.18 to 35.29)	3273	65481	47.33 (47.26 to 47.39)	698	36181.77	16.37 (16.33 to 16.42)	1294	40021	26.45 (26.39 to 26.50)
	IMD=2	1113	29623.08	36.77 (36.72 to 36.83)	1816	35086	49.89 (49.83 to 49.96)	329	18258.99	14.91 (14.87 to 14.95)	690	20373	27.40 (27.35 to 27.46)
	IMD=3	1150	29708.45	37.67 (37.62 to 37.73)	1906	35246	52.36 (52.29 to 52.42)	332	18924.98	14.57 (14.52 to 14.61)	662	21133	24.78 (24.72 to 24.83)
	IMD=4	960	25305.48	37.16 (37.11 to 37.22)	1506	30017	48.14 (48.08 to 48.20)	318	15883.31	15.86 (15.82 to 15.90)	636	17841	27.08 (27.02 to 27.13)
	IMD=5	804	20220.61	39.22 (39.16 to 39.28)	1203	23693	49.58 (49.52 to 49.65)	269	12507.4	17.50 (17.46 to 17.55)	486	13972	27.23 (27.17 to 27.29)
	IMD=6	766	20711.54	36.73 (36.67 to 36.78)	1202	24431	48.17 (48.11 to 48.23)	249	12272.7	16.11 (16.07 to 16.16)	501	13814	27.93 (27.87 to 27.99)
	IMD=7	865	23954.83	34.93 (34.88 to 34.99)	1397	28092	48.21 (48.15 to 48.27)	312	15657.65	14.79 (14.74 to 14.83)	795	17772	31.86 (31.80 to 31.92)
	IMD=8	213	5426.511	39.95 (39.89 to 40.01)	313	6318	49.83 (49.76 to 49.89)	76	3038.565	20.32 (20.27 to 20.37)	122	3354	28.51 (28.45 to 28.57)
	IMD=9	219	4882.806	45.48 (45.41 to 45.54)	351	5802	60.50 (60.43 to 60.58)	77	2921.533	20.35 (20.30 to 20.40)	144	3336	31.43 (31.37 to 31.49)
	Most Deprivation	70	1748.276	40.12 (40.07 to 40.18)	109	2076	51.64 (51.57 to 51.70)	22	924.0027	19.86 (19.81 to 19.91)	49	1051	36.57 (36.50 to 36.64)
2007	Least Deprivation	2003	56090.53	34.51 (34.45 to 34.56)	3352	66605	47.70 (47.64 to 47.76)	656	37016.93	15.09 (15.04 to 15.13)	1256	41032	25.07 (25.02 to 25.13)
	IMD=2	1113	30640.39	35.17 (35.12 to 35.23)	1845	36475	48.46 (48.40 to 48.52)	364	19199.67	15.99 (15.95 to 16.03)	711	21390	27.03 (26.97 to 27.08)
	IMD=3	1113	30015.74	36.19 (36.13 to 36.24)	1893	35927	50.86 (50.79 to 50.92)	407	19441.68	17.14 (17.09 to 17.18)	764	21724	27.66 (27.60 to 27.71)
	IMD=4	998	25470.58	38.74 (38.68 to 38.80)	1585	30438	50.71 (50.65 to 50.78)	323	16212.12	16.01 (15.96 to 16.05)	626	18286	26.17 (26.11 to 26.23)
	IMD=5	842	21071.35	39.25 (39.19 to 39.31)	1320	25080	51.14 (51.07 to 51.20)	294	13160.68	18.17 (18.12 to 18.22)	531	14807	28.00 (27.95 to 28.06)
	IMD=6	818	21090.57	38.36 (38.31 to 38.42)	1324	25177	51.52 (51.46 to 51.59)	266	12697.85	17.01 (16.97 to 17.06)	508	14326	27.48 (27.42 to 27.53)
	IMD=7	865	24131.21	35.74 (35.68 to 35.79)	1449	28582	49.81 (49.74 to 49.87)	303	15918.2	14.22 (14.18 to 14.26)	770	18080	30.30 (30.24 to 30.36)
	IMD=8	207	5585.908	37.61 (37.55 to 37.66)	336	6595	51.01 (50.94 to 51.07)	59	3172.835	15.11 (15.07 to 15.15)	105	3518	23.66 (23.61 to 23.71)
	IMD=9	193	4870.546	39.97 (39.91 to 40.02)	343	5922	57.66 (57.59 to 57.73)	58	2973.624	15.10 (15.06 to 15.15)	127	3410	27.08 (27.02 to 27.13)
	Most Deprivation	74	1758.177	41.52 (41.46 to 41.58)	118	2107	55.40 (55.33 to 55.47)	29	925.2156	27.53 (27.47 to 27.58)	55	1068	41.59 (41.52 to 41.66)
2008	Least Deprivation	2097	57263.21	35.50 (35.45 to 35.56)	3501	67712	49.11 (49.05 to 49.17)	718	38151.61	16.09 (16.05 to 16.14)	1326	42074	25.87 (25.81 to 25.93)
	IMD=2	1123	31400.77	35.39 (35.34 to 35.44)	1859	37350	48.28 (48.22 to 48.35)	388	19853.97	16.51 (16.46 to 16.55)	733	22103	26.96 (26.90 to 27.01)
	IMD=3	1179	30791.6	37.72 (37.67 to 37.78)	1999	36765	52.68 (52.62 to 52.75)	421	20099.5	17.35 (17.30 to 17.40)	827	22359	29.18 (29.12 to 29.24)
	IMD=4	940	25996.35	35.82 (35.77 to 35.88)	1569	30938	49.24 (49.17 to 49.30)	333	16705.17	15.90 (15.85 to 15.94)	629	18680	25.81 (25.76 to 25.87)
	IMD=5	832	21387.35	38.60 (38.54 to 38.65)	1321	25528	50.36 (50.30 to 50.43)	254	13484.48	15.44 (15.40 to 15.49)	496	15094	25.87 (25.81 to 25.92)
	IMD=6	843	21528.67	38.82 (38.76 to 38.87)	1394	25671	53.25 (53.19 to 53.32)	262	13023.31	16.37 (16.33 to 16.41)	518	14628	27.59 (27.53 to 27.65)

	IMD=7	855	24438.18	34.73 (34.68 to 34.79)	1468	28874	49.76 (49.69 to 49.82)	289	16269.8	13.48 (13.44 to 13.52)	752	18369	29.59 (29.53 to 29.65)
	IMD=8	224	5773.583	38.84 (38.78 to 38.90)	359	6827	52.31 (52.25 to 52.38)	56	3299.781	13.72 (13.67 to 13.76)	104	3662	22.50 (22.45 to 22.55)
	IMD=9	227	4902.179	46.64 (46.58 to 46.71)	371	5973	62.23 (62.16 to 62.30)	62	3015.672	16.16 (16.12 to 16.20)	132	3455	28.44 (28.39 to 28.50)
	Most Deprivation	67	1789.596	39.63 (39.57 to 39.68)	112	2153	52.27 (52.21 to 52.34)	18	950.8337	17.12 (17.08 to 17.17)	39	1092	29.93 (29.87 to 29.99)
2009	Least Deprivation	2116	58527.51	34.96 (34.90 to 35.01)	3518	69454	47.87 (47.81 to 47.93)	667	39208.86	14.59 (14.55 to 14.63)	1290	43323	24.52 (24.46 to 24.57)
	IMD=2	1236	31798.07	37.62 (37.57 to 37.68)	2013	37970	50.88 (50.82 to 50.95)	411	20313.71	17.23 (17.19 to 17.28)	781	22647	28.32 (28.26 to 28.38)
	IMD=3	1202	31414.49	37.10 (37.04 to 37.15)	2007	37571	51.07 (51.00 to 51.13)	431	20683.98	17.11 (17.06 to 17.15)	819	23037	27.98 (27.92 to 28.04)
	IMD=4	1010	26549.17	37.81 (37.75 to 37.86)	1660	31650	51.23 (51.17 to 51.30)	350	17149.97	16.32 (16.28 to 16.36)	620	19175	24.87 (24.82 to 24.93)
	IMD=5	821	21851.31	37.09 (37.04 to 37.15)	1336	26116	50.09 (50.02 to 50.15)	272	13942.76	15.96 (15.92 to 16.00)	504	15546	25.55 (25.50 to 25.61)
	IMD=6	828	21923.33	37.59 (37.54 to 37.65)	1395	26184	52.17 (52.11 to 52.24)	280	13408.02	16.81 (16.77 to 16.86)	529	15030	27.42 (27.36 to 27.48)
	IMD=7	885	24780.04	34.88 (34.83 to 34.93)	1569	29477	51.76 (51.70 to 51.83)	307	16650.45	13.76 (13.72 to 13.81)	740	18778	28.12 (28.06 to 28.18)
	IMD=8	230	6014.584	38.29 (38.23 to 38.35)	370	7180	50.65 (50.59 to 50.72)	53	3489.949	12.19 (12.15 to 12.22)	96	3858	19.70 (19.65 to 19.75)
	IMD=9	198	4972.109	40.82 (40.76 to 40.87)	341	6118	55.79 (55.72 to 55.85)	58	3080.879	14.83 (14.79 to 14.87)	115	3515	24.86 (24.81 to 24.92)
	Most Deprivation	80	1811.584	45.68 (45.62 to 45.74)	129	2197	59.28 (59.21 to 59.35)	17	966.8391	15.70 (15.66 to 15.75)	41	1112	30.25 (30.19 to 30.31)
2010	Least Deprivation	2230	59872.48	36.07 (36.01 to 36.12)	3722	71353	49.30 (49.23 to 49.36)	738	40433.07	15.68 (15.63 to 15.72)	1376	44785	25.49 (25.44 to 25.55)
	IMD=2	1204	32607.29	36.23 (36.17 to 36.28)	1980	39040	48.91 (48.85 to 48.97)	455	20986.1	18.50 (18.45 to 18.54)	861	23471	29.97 (29.91 to 30.03)
	IMD=3	1247	32244.15	37.55 (37.49 to 37.61)	2119	38719	52.24 (52.17 to 52.31)	440	21328.14	17.02 (16.97 to 17.06)	839	23905	27.73 (27.68 to 27.79)
	IMD=4	977	27110.7	35.15 (35.10 to 35.20)	1637	32448	48.50 (48.43 to 48.56)	352	17638.6	16.05 (16.00 to 16.09)	649	19782	25.22 (25.16 to 25.27)
	IMD=5	823	22419.38	36.07 (36.02 to 36.13)	1366	26915	49.29 (49.23 to 49.36)	268	14478.31	15.30 (15.25 to 15.34)	493	16177	24.13 (24.08 to 24.19)
	IMD=6	760	22408.56	33.38 (33.33 to 33.43)	1321	26899	47.86 (47.80 to 47.92)	273	13809.06	16.02 (15.98 to 16.06)	518	15527	26.17 (26.12 to 26.23)
	IMD=7	905	25082.77	35.63 (35.58 to 35.69)	1559	29975	50.74 (50.68 to 50.81)	333	16965.44	14.89 (14.85 to 14.93)	773	19155	29.18 (29.12 to 29.24)
	IMD=8	229	6225.818	36.79 (36.74 to 36.85)	364	7487	48.14 (48.07 to 48.20)	54	3677.394	11.82 (11.79 to 11.86)	86	4061	16.94 (16.90 to 16.99)
	IMD=9	211	5054.439	41.48 (41.43 to 41.54)	351	6240	56.05 (55.98 to 56.12)	72	3168.419	17.85 (17.80 to 17.90)	124	3580	25.91 (25.85 to 25.97)
	Most Deprivation	77	1817.599	42.61 (42.55 to 42.67)	129	2255	56.83 (56.76 to 56.90)	27	986.3737	23.44 (23.38 to 23.49)	50	1135	36.10 (36.04 to 36.17)
2011	Least Deprivation	2179	60999.99	34.55 (34.49 to 34.60)	3697	72554	48.27 (48.21 to 48.34)	732	41492.01	15.08 (15.03 to 15.12)	1378	45795	24.77 (24.72 to 24.83)
	IMD=2	1282	33180.9	37.58 (37.53 to 37.64)	2135	39691	51.65 (51.58 to 51.71)	371	21529.31	14.57 (14.53 to 14.61)	763	24028	25.74 (25.68 to 25.79)
	IMD=3	1329	32939.29	39.16 (39.10 to 39.22)	2210	39619	53.16 (53.10 to 53.23)	409	21970.83	15.31 (15.27 to 15.36)	804	24637	25.58 (25.53 to 25.64)
	IMD=4	1082	27673.42	38.19 (38.14 to 38.25)	1785	33086	52.16 (52.10 to 52.23)	348	18103.9	15.35 (15.30 to 15.39)	628	20224	23.76 (23.70 to 23.81)
	IMD=5	892	22991.03	37.69 (37.63 to 37.74)	1422	27476	49.65 (49.59 to 49.72)	304	14957.69	16.62 (16.57 to 16.66)	504	16646	23.78 (23.72 to 23.83)
	IMD=6	872	22923.98	37.50 (37.45 to 37.56)	1472	27396	52.36 (52.29 to 52.42)	255	14256.85	14.56 (14.51 to 14.60)	536	15944	26.34 (26.29 to 26.40)

	IMD=7	919	25506.75	35.47 (35.41 to 35.52)	1593	30383	51.25 (51.18 to 51.31)	281	17326.57	12.21 (12.17 to 12.24)	725	19489	26.82 (26.76 to 26.88)
	IMD=8	251	6349.508	40.02 (39.96 to 40.08)	402	7627	52.47 (52.40 to 52.54)	69	3790.801	14.99 (14.95 to 15.04)	114	4184	21.85 (21.80 to 21.90)
	IMD=9	250	5137.342	48.89 (48.83 to 48.96)	413	6333	64.43 (64.36 to 64.50)	70	3259.617	16.90 (16.86 to 16.95)	121	3673	24.71 (24.65 to 24.76)
	Most Deprivation	90	1856.698	49.36 (49.29 to 49.42)	155	2275	68.77 (68.69 to 68.84)	23	1005.963	20.22 (20.17 to 20.27)	49	1146	34.50 (34.44 to 34.57)
2012	Least Deprivation	2234	62142.64	34.79 (34.74 to 34.85)	3726	73677	48.17 (48.11 to 48.23)	753	42505.42	15.07 (15.03 to 15.11)	1391	46795	24.25 (24.20 to 24.31)
	IMD=2	1304	33681.57	38.04 (37.98 to 38.09)	2197	40283	52.77 (52.71 to 52.84)	411	21987.03	15.77 (15.73 to 15.82)	827	24517	27.13 (27.07 to 27.19)
	IMD=3	1329	33354.05	38.90 (38.85 to 38.96)	2232	40116	53.42 (53.35 to 53.49)	489	22460.78	17.79 (17.75 to 17.84)	858	25075	26.76 (26.70 to 26.81)
	IMD=4	1066	28020.18	37.35 (37.30 to 37.41)	1778	33489	51.52 (51.45 to 51.59)	344	18483.37	14.90 (14.86 to 14.94)	609	20602	22.75 (22.70 to 22.80)
	IMD=5	942	23600.62	39.27 (39.21 to 39.32)	1508	28213	51.91 (51.84 to 51.98)	305	15371.36	16.12 (16.08 to 16.17)	505	17063	23.21 (23.15 to 23.26)
	IMD=6	875	23578.15	36.98 (36.93 to 37.04)	1536	28031	53.81 (53.75 to 53.88)	256	14655.16	14.16 (14.12 to 14.20)	514	16327	24.59 (24.54 to 24.65)
	IMD=7	983	25769.67	37.88 (37.82 to 37.93)	1703	30730	54.61 (54.54 to 54.68)	339	17568.27	14.60 (14.56 to 14.64)	756	19701	27.46 (27.41 to 27.52)
	IMD=8	241	6492.616	37.36 (37.30 to 37.41)	387	7808	49.38 (49.32 to 49.45)	67	3884.058	14.27 (14.23 to 14.32)	103	4282	19.51 (19.46 to 19.56)
	IMD=9	226	5205.906	44.13 (44.07 to 44.19)	400	6423	61.87 (61.80 to 61.94)	80	3307.283	18.99 (18.94 to 19.04)	127	3725	25.86 (25.80 to 25.91)
	Most Deprivation	86	1892.518	47.16 (47.09 to 47.22)	153	2361	65.80 (65.73 to 65.87)	26	1018.634	22.21 (22.16 to 22.27)	43	1186	29.36 (29.30 to 29.42)
2013	Least Deprivation	2238	62789.36	34.51 (34.45 to 34.56)	3746	74720	47.53 (47.46 to 47.59)	775	43061.49	15.18 (15.13 to 15.22)	1388	47635	23.50 (23.44 to 23.55)
	IMD=2	1228	33578.29	35.77 (35.72 to 35.83)	2079	40685	49.25 (49.19 to 49.32)	441	22264.71	16.70 (16.65 to 16.74)	833	24959	26.90 (26.84 to 26.95)
	IMD=3	1355	33524.42	39.24 (39.18 to 39.29)	2309	40681	54.03 (53.96 to 54.10)	493	22761.88	17.76 (17.71 to 17.80)	871	25552	26.66 (26.61 to 26.72)
	IMD=4	1041	28258.06	35.81 (35.76 to 35.87)	1727	33837	48.86 (48.79 to 48.92)	395	18607.5	16.91 (16.86 to 16.95)	680	20825	24.99 (24.93 to 25.04)
	IMD=5	900	23908.87	37.25 (37.20 to 37.31)	1466	28689	49.83 (49.77 to 49.90)	311	15550.66	16.23 (16.18 to 16.27)	519	17315	23.36 (23.31 to 23.41)
	IMD=6	905	23600.97	37.98 (37.93 to 38.04)	1536	28484	52.97 (52.90 to 53.03)	278	14763.72	15.32 (15.28 to 15.36)	502	16583	23.65 (23.59 to 23.70)
	IMD=7	1006	25014.72	39.07 (39.02 to 39.13)	1674	30999	52.22 (52.16 to 52.29)	346	17069.51	15.13 (15.08 to 15.17)	712	19862	25.41 (25.35 to 25.46)
	IMD=8	263	6593.029	40.30 (40.24 to 40.35)	410	7963	51.14 (51.07 to 51.20)	64	3940.515	13.17 (13.13 to 13.21)	105	4374	19.14 (19.09 to 19.19)
	IMD=9	234	5230.453	44.59 (44.53 to 44.65)	410	6541	61.98 (61.91 to 62.06)	75	3315.329	17.78 (17.73 to 17.82)	130	3781	26.35 (26.29 to 26.41)
	Most Deprivation	87	1897.697	49.05 (48.99 to 49.12)	162	2377	70.52 (70.45 to 70.60)	27	1037.196	21.88 (21.83 to 21.94)	52	1196	34.35 (34.29 to 34.41)
2014	Least Deprivation	2317	63506.48	35.13 (35.08 to 35.18)	3800	75402	47.68 (47.61 to 47.74)	790	43698.58	15.30 (15.26 to 15.35)	1411	48274	23.65 (23.59 to 23.70)
	IMD=2	1318	33853.35	37.99 (37.93 to 38.05)	2152	40689	50.83 (50.77 to 50.90)	418	22681.05	15.43 (15.39 to 15.48)	805	25313	25.48 (25.42 to 25.53)
	IMD=3	1367	33645.46	39.69 (39.63 to 39.74)	2274	40820	53.66 (53.59 to 53.72)	495	23054.09	17.53 (17.49 to 17.58)	876	25847	26.45 (26.39 to 26.51)
	IMD=4	1088	28192.24	38.11 (38.06 to 38.17)	1809	33956	51.68 (51.61 to 51.74)	372	18809.7	15.85 (15.81 to 15.90)	625	21033	22.77 (22.72 to 22.82)
	IMD=5	934	24254.44	37.94 (37.88 to 37.99)	1571	29051	52.69 (52.62 to 52.75)	335	15821.24	17.13 (17.09 to 17.18)	552	17611	24.53 (24.48 to 24.59)
	IMD=6	918	23567.45	38.86 (38.80 to 38.91)	1533	28456	52.98 (52.91 to 53.04)	318	14858.24	17.40 (17.35 to 17.44)	549	16603	25.84 (25.79 to 25.90)

	IMD=7	1110	24855.32	43.75 (43.69 to 43.81)	1830	30135	58.98 (58.91 to 59.05)	333	17122.79	14.58 (14.54 to 14.62)	661	19369	24.20 (24.15 to 24.25)
	IMD=8	289	6683.239	43.80 (43.74 to 43.86)	452	8042	56.13 (56.06 to 56.20)	83	3996.03	17.17 (17.13 to 17.22)	136	4429	24.61 (24.56 to 24.67)
	IMD=9	234	5220.282	45.01 (44.95 to 45.08)	414	6513	62.76 (62.69 to 62.84)	67	3349.136	15.86 (15.81 to 15.90)	119	3808	24.20 (24.14 to 24.25)
	Most Deprivation	92	1897.363	49.96 (49.89 to 50.02)	157	2381	67.02 (66.94 to 67.09)	22	1040.862	18.90 (18.86 to 18.95)	41	1189	28.26 (28.20 to 28.32)
2015	Least Deprivation	2259	64704.12	33.67 (33.62 to 33.73)	3757	76851	46.31 (46.25 to 46.38)	846	44538.81	15.99 (15.95 to 16.04)	1424	49224	23.40 (23.35 to 23.45)
	IMD=2	1305	34469.18	36.42 (36.36 to 36.47)	2088	41354	48.20 (48.13 to 48.26)	464	23140.64	16.88 (16.83 to 16.92)	818	25809	25.52 (25.47 to 25.58)
	IMD=3	1323	33958.8	38.09 (38.03 to 38.14)	2270	41349	52.64 (52.58 to 52.71)	465	23304.14	16.26 (16.22 to 16.31)	910	26179	27.16 (27.10 to 27.21)
	IMD=4	1086	28518.07	37.28 (37.22 to 37.33)	1798	34290	50.74 (50.67 to 50.80)	395	18960.83	16.57 (16.53 to 16.62)	660	21224	23.74 (23.69 to 23.79)
	IMD=5	841	24510.74	33.70 (33.64 to 33.75)	1419	29365	47.06 (46.99 to 47.12)	347	15967.59	17.55 (17.50 to 17.59)	585	17839	25.63 (25.57 to 25.68)
	IMD=6	943	23971.95	39.26 (39.20 to 39.32)	1531	28947	52.15 (52.08 to 52.22)	321	15071.72	17.36 (17.31 to 17.40)	583	16843	27.04 (26.98 to 27.09)
	IMD=7	1019	24947.21	40.24 (40.18 to 40.30)	1712	30444	55.07 (55.00 to 55.14)	344	17353.69	14.90 (14.86 to 14.94)	668	19596	24.51 (24.46 to 24.57)
	IMD=8	268	6879.814	39.33 (39.28 to 39.39)	448	8278	53.83 (53.76 to 53.90)	80	4109.812	16.28 (16.24 to 16.33)	125	4549	22.29 (22.24 to 22.34)
	IMD=9	234	5365.317	43.94 (43.88 to 44.00)	439	6629	65.66 (65.58 to 65.73)	82	3376.824	19.44 (19.40 to 19.49)	129	3830	25.85 (25.80 to 25.91)
	Most Deprivation	90	1922.834	48.14 (48.08 to 48.21)	159	2402	67.78 (67.70 to 67.85)	22	1038.62	18.46 (18.41 to 18.51)	38	1190	26.75 (26.69 to 26.81)
2016	Least Deprivation	2227	66247.85	32.15 (32.10 to 32.20)	3664	78116	43.96 (43.90 to 44.02)	864	45627.84	15.78 (15.74 to 15.83)	1488	50232	23.83 (23.78 to 23.88)
	IMD=2	1216	35292.18	33.73 (33.68 to 33.78)	1985	42013	45.25 (45.19 to 45.31)	487	23563.35	17.33 (17.28 to 17.37)	862	26276	26.32 (26.27 to 26.38)
	IMD=3	1264	34636.38	35.69 (35.64 to 35.75)	2132	41796	48.87 (48.81 to 48.94)	498	23791.91	17.02 (16.97 to 17.06)	878	26593	25.76 (25.71 to 25.82)
	IMD=4	1084	29109.91	36.22 (36.16 to 36.27)	1751	34808	48.44 (48.38 to 48.51)	420	19247.99	17.45 (17.41 to 17.50)	701	21476	25.06 (25.00 to 25.11)
	IMD=5	870	25129.86	33.92 (33.86 to 33.97)	1399	29912	45.49 (45.43 to 45.55)	341	16317.62	17.00 (16.95 to 17.04)	592	18183	25.57 (25.52 to 25.63)
	IMD=6	906	25020.87	36.28 (36.23 to 36.34)	1531	29961	50.52 (50.45 to 50.58)	315	15658.75	16.31 (16.26 to 16.35)	580	17433	26.04 (25.98 to 26.09)
	IMD=7	968	25676.12	36.87 (36.81 to 36.92)	1672	31104	52.27 (52.20 to 52.34)	372	17797.57	15.70 (15.66 to 15.74)	702	19974	25.36 (25.30 to 25.41)
	IMD=8	278	7185.782	38.87 (38.81 to 38.93)	472	8634	54.27 (54.21 to 54.34)	73	4258.494	14.17 (14.12 to 14.21)	148	4721	25.19 (25.14 to 25.25)
	IMD=9	234	5497.635	42.96 (42.90 to 43.02)	415	6791	60.46 (60.39 to 60.53)	88	3463.294	19.83 (19.78 to 19.88)	148	3889	29.19 (29.13 to 29.25)
	Most Deprivation	58	2030.051	29.86 (29.81 to 29.91)	137	2496	55.90 (55.83 to 55.97)	29	1106.743	21.46 (21.40 to 21.51)	52	1255	32.89 (32.83 to 32.95)
2017	Least Deprivation	2079	67655.14	29.56 (29.51 to 29.61)	3388	79565	40.17 (40.11 to 40.23)	880	46260.04	15.94 (15.90 to 15.99)	1478	51102	23.33 (23.28 to 23.39)
	IMD=2	1150	36054.68	30.87 (30.82 to 30.92)	1890	42783	42.06 (42.00 to 42.12)	533	24054.68	18.37 (18.32 to 18.41)	907	26837	26.96 (26.90 to 27.01)
	IMD=3	1184	35565.92	32.19 (32.14 to 32.24)	1988	42670	44.35 (44.29 to 44.41)	539	24112.71	18.12 (18.07 to 18.16)	951	27063	27.46 (27.40 to 27.51)
	IMD=4	1025	29645.62	33.85 (33.80 to 33.91)	1692	35466	46.23 (46.16 to 46.29)	398	19500.16	16.26 (16.22 to 16.31)	689	21802	24.26 (24.21 to 24.32)
	IMD=5	905	25477.52	35.27 (35.22 to 35.33)	1459	30364	46.96 (46.89 to 47.02)	339	16510.42	16.54 (16.49 to 16.58)	576	18506	24.21 (24.15 to 24.26)
	IMD=6	912	25687.68	35.42 (35.37 to 35.48)	1580	30746	50.63 (50.56 to 50.69)	338	15925.43	17.23 (17.19 to 17.28)	571	17858	25.05 (24.99 to 25.10)

	IMD=7	1012	26136.55	37.81 (37.75 to 37.86)	1703	31675	51.73 (51.67 to 51.80)	404	18008.51	16.95 (16.90 to 16.99)	708	20268	25.44 (25.39 to 25.50)
	IMD=8	297	7362.757	40.57 (40.52 to 40.63)	489	8883	54.73 (54.66 to 54.80)	75	4348.391	14.06 (14.02 to 14.10)	137	4838	22.57 (22.52 to 22.62)
	IMD=9	210	5613.04	37.67 (37.61 to 37.72)	386	6947	55.13 (55.06 to 55.19)	74	3510.765	17.15 (17.11 to 17.20)	152	3972	29.60 (29.54 to 29.66)
	Most Deprivation	70	2023.814	36.09 (36.04 to 36.15)	140	2488	57.00 (56.93 to 57.07)	26	1097.287	19.67 (19.63 to 19.72)	51	1245	31.54 (31.48 to 31.61)
2018	Least Deprivation	2050	70030.52	28.19 (28.14 to 28.24)	3215	81663	37.36 (37.30 to 37.42)	962	47371.86	17.01 (16.96 to 17.05)	1494	52315	22.97 (22.92 to 23.03)
	IMD=2	1144	37296.83	29.96 (29.91 to 30.01)	1834	43865	40.02 (39.96 to 40.08)	498	24550.97	16.83 (16.78 to 16.87)	856	27414	24.86 (24.80 to 24.91)
	IMD=3	1183	36737.04	31.25 (31.20 to 31.30)	1942	43646	42.73 (42.67 to 42.79)	503	24596.73	16.47 (16.42 to 16.51)	938	27634	26.20 (26.14 to 26.25)
	IMD=4	969	30362.43	31.37 (31.32 to 31.42)	1602	36062	43.15 (43.09 to 43.21)	400	19820.24	16.10 (16.06 to 16.15)	657	22130	22.91 (22.85 to 22.96)
	IMD=5	868	26107.96	32.62 (32.57 to 32.67)	1371	30853	42.95 (42.89 to 43.01)	382	16744.94	18.28 (18.24 to 18.33)	646	18785	26.65 (26.59 to 26.71)
	IMD=6	863	26033.31	32.89 (32.84 to 32.95)	1445	31926	44.33 (44.27 to 44.39)	299	16118.89	14.91 (14.87 to 14.96)	525	18643	22.01 (21.96 to 22.06)
	IMD=7	830	25058.15	32.31 (32.26 to 32.36)	1467	32135	44.03 (43.97 to 44.09)	363	16976.82	15.94 (15.90 to 15.98)	668	20499	23.38 (23.33 to 23.44)
	IMD=8	228	7265.613	31.22 (31.17 to 31.27)	438	9168	47.31 (47.24 to 47.37)	81	4266.201	15.99 (15.95 to 16.04)	130	5022	20.74 (20.69 to 20.79)
	IMD=9	169	4858.524	35.65 (35.60 to 35.71)	299	7178	41.56 (41.50 to 41.62)	68	2862.639	18.64 (18.60 to 18.69)	112	4072	21.30 (21.25 to 21.35)
	Most Deprivation	77	2073.476	38.08 (38.02 to 38.13)	128	2517	51.43 (51.37 to 51.50)	22	1103.121	16.36 (16.32 to 16.41)	38	1257	23.26 (23.21 to 23.32)
2019	Least Deprivation	2004	71032.34	27.35 (27.30 to 27.39)	3129	83475	35.54 (35.48 to 35.59)	1004	47490.78	17.55 (17.50 to 17.59)	1585	53388	23.70 (23.64 to 23.75)
	IMD=2	1156	37981.38	29.84 (29.79 to 29.89)	1813	45214	38.63 (38.58 to 38.69)	542	24623.36	18.21 (18.16 to 18.25)	943	28092	26.66 (26.60 to 26.72)
	IMD=3	1102	35668.35	29.68 (29.63 to 29.73)	1770	44421	37.89 (37.83 to 37.95)	510	23592.6	17.44 (17.39 to 17.48)	897	28055	24.53 (24.48 to 24.59)
	IMD=4	837	28767.26	28.43 (28.38 to 28.48)	1386	36859	36.00 (35.94 to 36.05)	416	18404.02	18.27 (18.22 to 18.31)	637	22505	21.74 (21.69 to 21.80)
	IMD=5	768	25593.9	29.52 (29.47 to 29.57)	1300	31495	40.01 (39.95 to 40.07)	386	16231.06	19.15 (19.10 to 19.20)	684	19160	27.59 (27.53 to 27.64)
	IMD=6	779	23933.95	32.48 (32.43 to 32.53)	1301	31939	39.95 (39.89 to 40.00)	295	14597.27	16.38 (16.34 to 16.43)	501	18727	20.84 (20.79 to 20.89)
	IMD=7	756	23167.78	31.99 (31.94 to 32.04)	1273	30820	40.23 (40.18 to 40.29)	335	15501.95	16.29 (16.25 to 16.33)	626	19603	23.12 (23.07 to 23.17)
	IMD=8	210	6425.684	32.96 (32.91 to 33.02)	345	8906	38.41 (38.35 to 38.47)	81	3704.936	17.78 (17.74 to 17.83)	122	4811	19.98 (19.93 to 20.03)
	IMD=9	143	4011.97	36.84 (36.78 to 36.89)	244	5865	41.69 (41.63 to 41.75)	38	2277.049	13.05 (13.01 to 13.09)	89	3147	21.99 (21.94 to 22.04)
	Most Deprivation	46	1784.257	27.30 (27.25 to 27.34)	100	2535	39.78 (39.72 to 39.83)	20	954.8172	17.45 (17.41 to 17.50)	30	1289	17.89 (17.84 to 17.93)
**South West**													
2004	Least Deprivation	1805	54554.33	31.87 (31.82 to 31.92)	2812	62832	42.70 (42.64 to 42.76)	753	34377.77	18.06 (18.02 to 18.11)	1460	38101	30.28 (30.22 to 30.34)
	IMD=2	1920	57953.44	31.98 (31.93 to 32.04)	2929	66888	41.79 (41.73 to 41.85)	821	37421.26	17.23 (17.18 to 17.27)	1542	41646	28.14 (28.08 to 28.20)
	IMD=3	1824	52299.12	34.06 (34.01 to 34.11)	2671	60191	42.98 (42.92 to 43.04)	681	31497.39	17.42 (17.37 to 17.46)	1376	35006	30.45 (30.39 to 30.51)
	IMD=4	2014	64365.52	30.70 (30.65 to 30.75)	3081	73877	40.51 (40.45 to 40.56)	859	38690.24	18.04 (17.99 to 18.09)	1542	42822	28.38 (28.32 to 28.43)
	IMD=5	2034	60094.23	33.43 (33.38 to 33.48)	3175	69417	44.60 (44.54 to 44.67)	741	36723.55	15.89 (15.85 to 15.94)	1485	40845	27.65 (27.60 to 27.71)

	IMD=6	1935	56407.62	33.51 (33.46 to 33.56)	3120	65794	46.09 (46.02 to 46.15)	794	35632.51	17.07 (17.03 to 17.12)	1629	39864	30.19 (30.13 to 30.25)
	IMD=7	1965	63145.49	30.70 (30.65 to 30.75)	3199	72444	43.30 (43.24 to 43.36)	765	38263.63	15.21 (15.17 to 15.25)	1630	42517	28.24 (28.19 to 28.30)
	IMD=8	1635	45529.24	35.78 (35.73 to 35.84)	2706	53027	50.52 (50.46 to 50.59)	628	26113.55	18.58 (18.53 to 18.63)	1275	29099	32.76 (32.70 to 32.83)
	IMD=9	1312	35998.98	36.76 (36.70 to 36.81)	2250	41989	53.53 (53.46 to 53.59)	448	20234.18	16.87 (16.82 to 16.91)	1008	22614	32.76 (32.70 to 32.82)
	Most Deprivation	1654	43270.05	39.10 (39.05 to 39.16)	3033	50888	60.37 (60.30 to 60.45)	530	23293.94	17.85 (17.81 to 17.90)	1324	26400	37.81 (37.75 to 37.88)
2005	Least Deprivation	1832	55370.3	32.21 (32.16 to 32.27)	2957	64220	43.77 (43.71 to 43.83)	659	35112.83	15.25 (15.21 to 15.29)	1448	39119	29.27 (29.21 to 29.33)
	IMD=2	2025	58532.47	33.09 (33.04 to 33.14)	3133	68352	43.51 (43.45 to 43.57)	818	38000.03	16.96 (16.92 to 17.01)	1634	42582	29.17 (29.11 to 29.23)
	IMD=3	1698	52816.87	31.10 (31.04 to 31.15)	2682	61332	42.13 (42.07 to 42.19)	670	31955.31	16.85 (16.81 to 16.90)	1399	35776	30.25 (30.19 to 30.31)
	IMD=4	2101	65263.39	31.60 (31.55 to 31.65)	3325	75720	42.61 (42.55 to 42.67)	805	39586.9	16.55 (16.51 to 16.60)	1598	44171	28.57 (28.51 to 28.63)
	IMD=5	2009	60745.56	32.30 (32.25 to 32.35)	3307	70739	45.24 (45.17 to 45.30)	744	37389.52	15.72 (15.67 to 15.76)	1533	41736	28.09 (28.04 to 28.15)
	IMD=6	1958	56830.21	33.69 (33.64 to 33.74)	3252	66872	47.05 (46.99 to 47.11)	784	36013.68	16.80 (16.76 to 16.85)	1653	40574	30.08 (30.02 to 30.14)
	IMD=7	1902	63653.96	29.43 (29.38 to 29.48)	3238	73942	42.59 (42.53 to 42.65)	785	38706.56	15.64 (15.60 to 15.68)	1716	43317	29.30 (29.24 to 29.36)
	IMD=8	1619	45901.75	35.07 (35.01 to 35.12)	2809	54104	51.13 (51.06 to 51.19)	595	26289.92	17.67 (17.62 to 17.71)	1267	29577	32.17 (32.11 to 32.24)
	IMD=9	1278	35871.79	35.89 (35.83 to 35.94)	2305	42598	54.26 (54.20 to 54.33)	461	20167.73	18.06 (18.02 to 18.11)	1026	22856	33.84 (33.78 to 33.90)
	Most Deprivation	1791	43206.96	42.41 (42.35 to 42.47)	3332	51986	64.98 (64.91 to 65.05)	573	23230.43	19.65 (19.60 to 19.70)	1497	26691	42.66 (42.59 to 42.74)
2006	Least Deprivation	1977	56096.37	34.33 (34.28 to 34.39)	3191	65575	46.62 (46.56 to 46.68)	708	36038.74	16.04 (15.99 to 16.08)	1481	40183	29.22 (29.16 to 29.28)
	IMD=2	2028	59266.35	32.96 (32.91 to 33.02)	3197	69789	43.66 (43.60 to 43.72)	848	38786.44	17.37 (17.33 to 17.42)	1651	43567	28.82 (28.76 to 28.88)
	IMD=3	1713	53341.89	31.38 (31.33 to 31.43)	2737	62370	42.33 (42.27 to 42.39)	650	32529.35	16.09 (16.04 to 16.13)	1352	36532	28.70 (28.64 to 28.75)
	IMD=4	2085	66501.38	30.60 (30.55 to 30.65)	3414	77496	42.53 (42.47 to 42.59)	749	40662.13	15.09 (15.05 to 15.13)	1574	45480	27.41 (27.35 to 27.47)
	IMD=5	2109	61676.55	33.62 (33.56 to 33.67)	3440	72350	46.15 (46.09 to 46.21)	742	38240.53	15.42 (15.38 to 15.47)	1556	42785	27.85 (27.80 to 27.91)
	IMD=6	1951	57872.51	32.99 (32.94 to 33.04)	3292	68316	46.55 (46.49 to 46.61)	690	36931.17	14.46 (14.41 to 14.50)	1517	41575	27.08 (27.03 to 27.14)
	IMD=7	1996	64928.83	30.34 (30.29 to 30.39)	3444	75726	44.39 (44.33 to 44.45)	752	39437.91	14.75 (14.71 to 14.80)	1751	44257	29.32 (29.26 to 29.38)
	IMD=8	1552	46159.86	33.53 (33.48 to 33.58)	2780	54894	50.00 (49.93 to 50.06)	550	26515.59	16.12 (16.07 to 16.16)	1306	30035	32.69 (32.63 to 32.75)
	IMD=9	1264	36269.34	35.27 (35.21 to 35.32)	2370	43257	54.86 (54.79 to 54.92)	432	20364.45	16.68 (16.64 to 16.73)	1032	23083	33.70 (33.64 to 33.77)
	Most Deprivation	1676	43545.59	39.57 (39.52 to 39.63)	3390	52931	65.02 (64.95 to 65.10)	479	23263.13	16.24 (16.20 to 16.29)	1447	26776	41.24 (41.17 to 41.31)
2007	Least Deprivation	1856	56556.57	31.96 (31.91 to 32.01)	3073	66399	44.23 (44.16 to 44.29)	764	36756.69	17.07 (17.03 to 17.12)	1548	41084	29.87 (29.81 to 29.93)
	IMD=2	2064	59676.96	33.35 (33.30 to 33.41)	3324	70366	44.87 (44.81 to 44.93)	871	39239.24	17.49 (17.45 to 17.54)	1705	44185	29.18 (29.13 to 29.24)
	IMD=3	1816	53844.17	33.04 (32.98 to 33.09)	2849	63256	43.54 (43.48 to 43.60)	676	33134.88	16.38 (16.34 to 16.43)	1392	37257	28.81 (28.75 to 28.87)
	IMD=4	2078	67383.01	30.19 (30.14 to 30.24)	3408	78786	41.92 (41.86 to 41.98)	744	41653.4	14.53 (14.49 to 14.57)	1574	46565	26.76 (26.70 to 26.81)
	IMD=5	2029	62193.92	31.79 (31.74 to 31.84)	3384	73445	44.58 (44.52 to 44.65)	776	38940.47	15.89 (15.85 to 15.93)	1610	43683	28.31 (28.25 to 28.37)

	IMD=6	2046	58275.92	33.97 (33.92 to 34.03)	3383	68969	47.12 (47.06 to 47.18)	745	37494.31	15.33 (15.28 to 15.37)	1646	42244	29.00 (28.94 to 29.06)
	IMD=7	2023	65505.97	30.65 (30.60 to 30.70)	3528	76726	45.09 (45.03 to 45.15)	738	39981.2	14.33 (14.29 to 14.37)	1729	44950	28.51 (28.45 to 28.57)
	IMD=8	1640	46465.61	35.53 (35.48 to 35.59)	2900	55393	52.02 (51.95 to 52.08)	560	26855.78	16.43 (16.38 to 16.47)	1265	30461	31.45 (31.39 to 31.52)
	IMD=9	1295	36496.21	35.70 (35.65 to 35.75)	2349	43866	53.54 (53.48 to 53.61)	422	20529.95	16.20 (16.16 to 16.25)	1024	23403	33.02 (32.95 to 33.08)
	Most Deprivation	1759	44039.97	41.20 (41.14 to 41.25)	3551	54017	67.03 (66.96 to 67.11)	453	23630.85	15.66 (15.62 to 15.70)	1380	27172	39.22 (39.16 to 39.29)
2008	Least Deprivation	1923	57605.94	32.40 (32.35 to 32.45)	3212	67383	45.63 (45.57 to 45.69)	778	37758.79	16.85 (16.80 to 16.89)	1612	42037	30.21 (30.15 to 30.27)
	IMD=2	2064	60597.25	32.98 (32.92 to 33.03)	3358	71196	45.01 (44.95 to 45.07)	887	40027.87	17.62 (17.57 to 17.66)	1754	44943	29.60 (29.54 to 29.66)
	IMD=3	1802	54871.99	32.38 (32.32 to 32.43)	2950	64167	44.89 (44.83 to 44.96)	724	34049.07	17.17 (17.13 to 17.22)	1499	38070	30.48 (30.42 to 30.54)
	IMD=4	2227	68301.71	32.00 (31.95 to 32.05)	3604	79607	43.90 (43.84 to 43.96)	849	42698.91	16.25 (16.21 to 16.29)	1663	47417	27.74 (27.68 to 27.79)
	IMD=5	2161	63296.33	33.50 (33.45 to 33.55)	3579	74403	46.58 (46.52 to 46.64)	817	39970.2	16.30 (16.25 to 16.34)	1650	44546	28.54 (28.48 to 28.60)
	IMD=6	1961	59013.79	32.72 (32.67 to 32.77)	3349	69634	46.57 (46.50 to 46.63)	869	38214.94	17.65 (17.60 to 17.69)	1783	42922	31.10 (31.04 to 31.16)
	IMD=7	1985	66303.04	29.47 (29.42 to 29.52)	3553	77200	45.06 (44.99 to 45.12)	738	40713.18	14.02 (13.98 to 14.06)	1696	45417	27.65 (27.59 to 27.71)
	IMD=8	1629	47117.54	34.45 (34.40 to 34.50)	2944	55968	51.89 (51.82 to 51.95)	585	27466.98	16.84 (16.79 to 16.88)	1349	31006	33.05 (32.98 to 33.11)
	IMD=9	1282	37037.67	35.13 (35.08 to 35.19)	2483	44294	56.25 (56.18 to 56.31)	418	20975.07	15.86 (15.81 to 15.90)	991	23757	31.67 (31.61 to 31.73)
	Most Deprivation	1680	44807.97	38.23 (38.17 to 38.28)	3570	54820	66.15 (66.07 to 66.22)	501	24071.28	17.15 (17.10 to 17.19)	1440	27578	41.13 (41.06 to 41.20)
2009	Least Deprivation	1883	58445.35	31.28 (31.23 to 31.33)	3178	68402	44.17 (44.11 to 44.24)	772	38672.93	16.18 (16.14 to 16.22)	1594	43135	28.99 (28.93 to 29.05)
	IMD=2	2195	60991.75	34.89 (34.84 to 34.95)	3537	71948	47.04 (46.98 to 47.10)	899	40711.3	17.42 (17.37 to 17.46)	1784	45771	29.45 (29.39 to 29.51)
	IMD=3	1872	55492.74	33.10 (33.04 to 33.15)	3039	65118	45.19 (45.13 to 45.25)	719	34862.16	16.48 (16.44 to 16.52)	1510	39088	29.82 (29.76 to 29.88)
	IMD=4	2271	68867.64	32.63 (32.58 to 32.68)	3747	80614	45.30 (45.24 to 45.36)	889	43497.45	16.63 (16.59 to 16.68)	1754	48427	28.46 (28.41 to 28.52)
	IMD=5	2149	63900.54	33.03 (32.98 to 33.09)	3572	75356	45.96 (45.90 to 46.02)	839	40712.75	16.45 (16.41 to 16.50)	1705	45512	28.75 (28.69 to 28.81)
	IMD=6	2035	59321.31	33.50 (33.44 to 33.55)	3441	70161	47.41 (47.35 to 47.48)	809	38641.75	16.23 (16.18 to 16.27)	1682	43543	28.88 (28.83 to 28.94)
	IMD=7	2089	66986.64	31.07 (31.02 to 31.12)	3698	78390	46.54 (46.47 to 46.60)	746	41362.03	14.07 (14.03 to 14.11)	1675	46312	27.07 (27.01 to 27.12)
	IMD=8	1739	47334.83	36.71 (36.66 to 36.77)	3043	56443	53.26 (53.19 to 53.32)	567	27819.49	16.37 (16.32 to 16.41)	1342	31503	32.79 (32.73 to 32.85)
	IMD=9	1339	37770.73	36.06 (36.01 to 36.12)	2541	45284	56.35 (56.28 to 56.41)	411	21445.32	15.58 (15.53 to 15.62)	1029	24269	32.73 (32.66 to 32.79)
	Most Deprivation	1854	45723.57	41.70 (41.64 to 41.76)	3837	56106	69.94 (69.87 to 70.02)	481	24603.37	15.90 (15.85 to 15.94)	1437	28163	40.27 (40.20 to 40.34)
2010	Least Deprivation	1989	59491.71	32.29 (32.24 to 32.35)	3358	69657	45.76 (45.70 to 45.82)	843	39662.03	17.24 (17.19 to 17.28)	1666	44395	29.33 (29.27 to 29.39)
	IMD=2	2051	61942.45	31.82 (31.77 to 31.87)	3393	73435	43.70 (43.64 to 43.76)	959	41618.65	18.10 (18.06 to 18.15)	1823	46926	29.28 (29.22 to 29.34)
	IMD=3	1877	56184.57	32.83 (32.77 to 32.88)	3113	66302	45.52 (45.46 to 45.58)	736	35581.5	16.67 (16.62 to 16.71)	1523	40063	29.40 (29.34 to 29.46)
	IMD=4	2289	69824.59	32.21 (32.16 to 32.26)	3805	81947	45.02 (44.96 to 45.08)	963	44528.23	17.53 (17.48 to 17.57)	1840	49735	29.10 (29.04 to 29.16)
	IMD=5	2044	64894.01	30.89 (30.84 to 30.94)	3537	76633	44.80 (44.74 to 44.86)	794	41593.27	15.18 (15.14 to 15.23)	1674	46612	27.56 (27.50 to 27.62)

	IMD=6	2065	60025.14	33.67 (33.62 to 33.73)	3524	71168	47.92 (47.86 to 47.98)	837	39307.36	16.52 (16.48 to 16.57)	1755	44392	29.48 (29.42 to 29.54)
	IMD=7	1980	67443.29	29.11 (29.06 to 29.16)	3565	79284	44.31 (44.25 to 44.38)	738	41994.39	13.74 (13.70 to 13.78)	1646	47103	26.19 (26.14 to 26.25)
	IMD=8	1591	47874.89	33.33 (33.28 to 33.39)	2936	57320	50.68 (50.61 to 50.74)	550	28331.67	15.51 (15.47 to 15.55)	1336	32085	32.01 (31.95 to 32.07)
	IMD=9	1375	38389.95	36.41 (36.36 to 36.47)	2596	46118	56.62 (56.56 to 56.69)	445	21816.88	16.48 (16.44 to 16.53)	1049	24729	33.04 (32.98 to 33.10)
	Most Deprivation	1693	46552.45	37.60 (37.55 to 37.66)	3784	57310	67.84 (67.77 to 67.92)	484	25264.97	15.87 (15.82 to 15.91)	1376	28850	37.97 (37.90 to 38.03)
2011	Least Deprivation	2111	60481.12	33.62 (33.56 to 33.67)	3536	70969	47.19 (47.13 to 47.25)	772	40586.78	15.38 (15.33 to 15.42)	1610	45394	27.56 (27.50 to 27.62)
	IMD=2	2162	62738.84	33.00 (32.94 to 33.05)	3566	74126	45.60 (45.54 to 45.67)	963	42335.62	17.78 (17.73 to 17.83)	1931	47831	30.28 (30.22 to 30.34)
	IMD=3	2054	57010.71	35.24 (35.19 to 35.29)	3326	67352	47.75 (47.69 to 47.81)	739	36393.12	16.36 (16.31 to 16.40)	1551	40934	29.27 (29.21 to 29.33)
	IMD=4	2430	71073.53	33.50 (33.45 to 33.55)	4014	83530	46.64 (46.58 to 46.70)	901	45641.01	15.99 (15.95 to 16.04)	1855	51008	28.51 (28.45 to 28.57)
	IMD=5	2158	65984.88	32.35 (32.30 to 32.40)	3666	77857	45.81 (45.74 to 45.87)	870	42576.07	16.22 (16.18 to 16.27)	1734	47619	27.92 (27.86 to 27.98)
	IMD=6	2109	60812.32	33.79 (33.74 to 33.84)	3594	72154	47.99 (47.92 to 48.05)	818	40038.65	15.85 (15.81 to 15.90)	1709	45120	28.27 (28.21 to 28.32)
	IMD=7	2130	68377.45	30.82 (30.77 to 30.87)	3824	80122	46.72 (46.66 to 46.78)	757	42933.46	13.76 (13.72 to 13.81)	1645	47899	25.88 (25.83 to 25.94)
	IMD=8	1723	48858.85	35.58 (35.53 to 35.63)	3147	58342	53.67 (53.61 to 53.74)	552	28905.18	15.29 (15.25 to 15.33)	1353	32692	31.90 (31.83 to 31.96)
	IMD=9	1343	38923.79	34.94 (34.89 to 35.00)	2661	46774	57.11 (57.04 to 57.18)	453	22291.79	16.68 (16.63 to 16.72)	1058	25171	32.94 (32.88 to 33.01)
	Most Deprivation	1811	47428.79	39.56 (39.51 to 39.62)	3903	58276	68.72 (68.64 to 68.80)	504	25793.89	16.74 (16.70 to 16.79)	1411	29412	39.12 (39.05 to 39.19)
2012	Least Deprivation	2011	61146.03	31.78 (31.73 to 31.83)	3395	71599	44.99 (44.93 to 45.05)	789	41607.86	15.22 (15.18 to 15.27)	1618	46275	27.00 (26.94 to 27.05)
	IMD=2	2134	63214.07	32.49 (32.44 to 32.54)	3480	74551	44.29 (44.23 to 44.35)	897	42991.14	16.19 (16.15 to 16.23)	1791	48371	27.52 (27.46 to 27.57)
	IMD=3	2068	57303.93	35.24 (35.19 to 35.29)	3397	67899	48.26 (48.19 to 48.32)	800	36961.79	17.24 (17.20 to 17.29)	1609	41464	29.80 (29.74 to 29.86)
	IMD=4	2420	71769.75	33.21 (33.16 to 33.26)	4036	84492	46.52 (46.45 to 46.58)	915	46390.21	15.84 (15.80 to 15.88)	1799	51803	27.03 (26.97 to 27.08)
	IMD=5	2177	66670.6	32.22 (32.16 to 32.27)	3701	78525	45.95 (45.89 to 46.01)	870	43171.59	15.99 (15.95 to 16.03)	1784	48193	28.28 (28.22 to 28.34)
	IMD=6	2085	61320.59	33.43 (33.38 to 33.49)	3609	72647	48.28 (48.21 to 48.34)	825	40615.97	15.78 (15.74 to 15.83)	1705	45660	27.83 (27.78 to 27.89)
	IMD=7	2047	68898.17	29.55 (29.50 to 29.60)	3704	80711	45.18 (45.11 to 45.24)	724	43609.46	12.95 (12.91 to 12.99)	1615	48416	25.04 (24.98 to 25.09)
	IMD=8	1724	49616.91	34.93 (34.87 to 34.98)	3140	59078	52.97 (52.90 to 53.04)	552	29459.25	15.00 (14.95 to 15.04)	1282	33073	29.92 (29.86 to 29.98)
	IMD=9	1448	39334.46	37.62 (37.57 to 37.68)	2798	47300	59.62 (59.55 to 59.69)	437	22597.23	15.68 (15.64 to 15.72)	1023	25460	31.29 (31.23 to 31.35)
	Most Deprivation	1805	48056.43	38.96 (38.91 to 39.02)	3971	59060	68.80 (68.72 to 68.87)	429	26300.77	13.93 (13.89 to 13.97)	1331	29875	36.42 (36.35 to 36.48)
2013	Least Deprivation	1996	61212.91	31.50 (31.45 to 31.55)	3322	72608	43.39 (43.33 to 43.45)	847	41948.73	16.14 (16.10 to 16.19)	1630	47130	26.59 (26.54 to 26.65)
	IMD=2	2108	63456.59	31.98 (31.93 to 32.03)	3407	75444	42.96 (42.90 to 43.02)	936	43180.14	16.76 (16.72 to 16.81)	1742	48986	26.37 (26.31 to 26.43)
	IMD=3	1912	56755.87	32.66 (32.61 to 32.71)	3228	67793	46.01 (45.94 to 46.07)	835	37125.4	17.90 (17.86 to 17.95)	1583	41906	28.90 (28.84 to 28.96)
	IMD=4	2399	71720.02	32.62 (32.57 to 32.67)	3914	85087	44.44 (44.38 to 44.50)	1015	46675.62	17.46 (17.42 to 17.51)	1893	52601	27.84 (27.78 to 27.90)
	IMD=5	2168	66252.92	32.14 (32.09 to 32.19)	3638	78618	44.92 (44.86 to 44.98)	904	43308.73	16.52 (16.47 to 16.56)	1707	48701	26.68 (26.62 to 26.73)

	IMD=6	2098	60664.89	34.10 (34.05 to 34.15)	3548	72913	47.37 (47.30 to 47.43)	896	40414.12	17.19 (17.15 to 17.24)	1720	46017	27.73 (27.67 to 27.79)
	IMD=7	2032	68017.23	29.61 (29.56 to 29.66)	3629	81348	43.80 (43.74 to 43.86)	837	43307.32	14.98 (14.94 to 15.03)	1687	48987	25.78 (25.72 to 25.84)
	IMD=8	1697	49120.61	34.70 (34.65 to 34.76)	3116	59702	51.89 (51.82 to 51.95)	592	29342.72	16.13 (16.08 to 16.17)	1368	33555	31.41 (31.35 to 31.47)
	IMD=9	1421	39189.27	37.06 (37.00 to 37.12)	2730	47723	57.75 (57.68 to 57.82)	457	22673.69	16.29 (16.25 to 16.34)	1026	25834	31.05 (30.98 to 31.11)
	Most Deprivation	1897	47967.77	40.88 (40.82 to 40.94)	3983	59606	68.06 (67.98 to 68.13)	506	26501.83	16.34 (16.29 to 16.38)	1348	30346	36.42 (36.36 to 36.49)
2014	Least Deprivation	1951	61289.2	30.62 (30.57 to 30.67)	3203	72476	42.18 (42.12 to 42.24)	859	42403.1	16.13 (16.09 to 16.18)	1565	47453	25.29 (25.23 to 25.34)
	IMD=2	2109	63137.53	32.02 (31.97 to 32.07)	3367	75612	42.42 (42.36 to 42.48)	999	43172.51	17.75 (17.71 to 17.80)	1798	49070	27.11 (27.05 to 27.17)
	IMD=3	1955	56638.24	33.40 (33.35 to 33.46)	3189	68274	44.94 (44.88 to 45.00)	816	37377.93	17.31 (17.26 to 17.36)	1552	42466	27.90 (27.84 to 27.95)
	IMD=4	2338	71769.02	31.78 (31.73 to 31.84)	3817	85509	43.10 (43.04 to 43.16)	1035	46928.12	17.55 (17.51 to 17.60)	1857	52987	27.11 (27.05 to 27.17)
	IMD=5	2173	66336.46	31.96 (31.91 to 32.01)	3606	78719	44.26 (44.20 to 44.32)	939	43469.69	17.00 (16.95 to 17.04)	1684	48959	26.17 (26.11 to 26.23)
	IMD=6	2064	60458.14	33.41 (33.36 to 33.47)	3474	72282	46.50 (46.44 to 46.56)	955	40519.65	18.14 (18.10 to 18.19)	1711	45859	27.73 (27.68 to 27.79)
	IMD=7	2147	67137.63	31.64 (31.59 to 31.69)	3567	79659	43.91 (43.85 to 43.97)	809	42977.75	14.72 (14.68 to 14.77)	1494	48152	23.52 (23.46 to 23.57)
	IMD=8	1752	48546.77	35.98 (35.93 to 36.04)	3036	58553	51.31 (51.25 to 51.38)	618	29270.12	16.82 (16.78 to 16.87)	1265	33193	29.44 (29.38 to 29.50)
	IMD=9	1429	38924.19	37.18 (37.13 to 37.24)	2605	47477	55.06 (54.99 to 55.13)	478	22677.96	17.17 (17.12 to 17.22)	950	25771	29.07 (29.01 to 29.13)
	Most Deprivation	1864	47465.29	40.27 (40.22 to 40.33)	3784	59189	64.84 (64.77 to 64.91)	529	26479.72	17.27 (17.23 to 17.32)	1144	30237	31.41 (31.35 to 31.47)
2015	Least Deprivation	1927	61927.12	29.75 (29.70 to 29.80)	3076	72729	40.09 (40.03 to 40.15)	906	42801.12	16.80 (16.75 to 16.84)	1615	47876	25.68 (25.62 to 25.73)
	IMD=2	2117	63064.45	32.46 (32.41 to 32.52)	3376	74687	43.12 (43.06 to 43.18)	1029	43379.54	18.17 (18.13 to 18.22)	1861	49130	27.85 (27.79 to 27.90)
	IMD=3	1880	56831.5	31.89 (31.84 to 31.94)	3181	67801	45.02 (44.96 to 45.08)	938	37644.7	19.64 (19.60 to 19.69)	1711	42690	30.30 (30.24 to 30.36)
	IMD=4	2378	72013.95	32.34 (32.29 to 32.39)	3880	85176	43.96 (43.90 to 44.02)	1126	47229.46	19.00 (18.95 to 19.04)	1928	53220	27.84 (27.78 to 27.89)
	IMD=5	2136	66171.24	31.36 (31.31 to 31.41)	3532	78479	43.53 (43.47 to 43.59)	983	43660.09	17.65 (17.60 to 17.69)	1810	49261	27.81 (27.76 to 27.87)
	IMD=6	2142	60065.85	35.01 (34.95 to 35.06)	3488	72018	47.00 (46.94 to 47.06)	887	40484.67	16.77 (16.72 to 16.81)	1703	46155	27.30 (27.25 to 27.36)
	IMD=7	2227	66525.14	33.26 (33.21 to 33.32)	3696	78747	46.12 (46.06 to 46.18)	840	43064.2	15.20 (15.16 to 15.25)	1519	48284	23.72 (23.66 to 23.77)
	IMD=8	1924	48166.63	40.16 (40.10 to 40.21)	3223	58338	54.96 (54.89 to 55.03)	677	29279.89	18.41 (18.36 to 18.45)	1297	33379	29.99 (29.93 to 30.05)
	IMD=9	1528	38171.77	40.59 (40.53 to 40.64)	2757	47216	58.55 (58.48 to 58.62)	477	22461.81	17.33 (17.28 to 17.37)	960	25865	29.39 (29.33 to 29.45)
	Most Deprivation	1993	47423.8	43.06 (43.00 to 43.12)	3865	59044	66.11 (66.03 to 66.18)	608	26614.27	19.52 (19.47 to 19.57)	1191	30425	32.07 (32.01 to 32.13)
2016	Least Deprivation	2022	62829.14	30.71 (30.66 to 30.76)	3174	73239	40.90 (40.84 to 40.96)	869	43479.53	15.74 (15.70 to 15.78)	1581	48385	24.68 (24.63 to 24.74)
	IMD=2	2043	63974.58	30.45 (30.40 to 30.50)	3285	75172	41.36 (41.30 to 41.41)	961	44118.45	16.56 (16.51 to 16.60)	1749	49654	25.75 (25.70 to 25.81)
	IMD=3	2036	57643.8	34.02 (33.96 to 34.07)	3263	68279	45.83 (45.77 to 45.89)	866	38118.65	17.77 (17.72 to 17.81)	1656	43143	28.88 (28.82 to 28.94)
	IMD=4	2393	72992.95	31.97 (31.92 to 32.02)	3890	85821	43.70 (43.64 to 43.76)	1079	47870.49	17.84 (17.80 to 17.89)	1956	53845	27.86 (27.81 to 27.92)
	IMD=5	2204	66959.68	32.34 (32.29 to 32.40)	3606	78700	44.69 (44.62 to 44.75)	957	44184.58	16.87 (16.83 to 16.92)	1770	49584	26.88 (26.82 to 26.94)

	IMD=6	2058	60667.25	33.06 (33.01 to 33.12)	3406	71984	45.78 (45.72 to 45.84)	948	40862.85	17.71 (17.66 to 17.76)	1747	46214	27.88 (27.82 to 27.93)
	IMD=7	2278	67378.77	33.39 (33.34 to 33.44)	3744	79286	46.31 (46.25 to 46.37)	895	43565.32	15.98 (15.94 to 16.03)	1597	48637	24.78 (24.72 to 24.83)
	IMD=8	1872	48539.46	38.63 (38.57 to 38.69)	3192	58336	54.30 (54.24 to 54.37)	653	29632.81	17.60 (17.55 to 17.64)	1310	33510	30.15 (30.09 to 30.21)
	IMD=9	1471	38595.03	38.70 (38.65 to 38.76)	2662	46838	56.96 (56.90 to 57.03)	534	22563.19	19.40 (19.35 to 19.45)	1023	25521	31.67 (31.61 to 31.74)
	Most Deprivation	2175	47977.08	46.28 (46.22 to 46.34)	4094	59417	69.44 (69.36 to 69.51)	589	26821.58	18.95 (18.90 to 19.00)	1237	30478	33.37 (33.31 to 33.44)
2017	Least Deprivation	2033	63782.44	30.08 (30.03 to 30.13)	3197	74206	40.27 (40.21 to 40.33)	962	44086.4	17.13 (17.09 to 17.18)	1631	49149	25.03 (24.97 to 25.08)
	IMD=2	2185	65121.15	32.02 (31.96 to 32.07)	3363	76392	41.72 (41.66 to 41.78)	996	44475.22	17.00 (16.95 to 17.04)	1786	50157	26.03 (25.97 to 26.08)
	IMD=3	1988	58719.3	32.78 (32.72 to 32.83)	3217	69347	44.33 (44.27 to 44.39)	900	38688.88	18.10 (18.06 to 18.15)	1696	43798	29.02 (28.96 to 29.08)
	IMD=4	2403	74456.52	31.33 (31.27 to 31.38)	3772	87318	41.36 (41.30 to 41.41)	1072	48701.82	17.37 (17.33 to 17.42)	1947	54865	27.05 (26.99 to 27.11)
	IMD=5	2155	68288.14	30.54 (30.49 to 30.59)	3507	80193	42.31 (42.25 to 42.37)	994	44792.91	17.23 (17.19 to 17.28)	1863	50430	27.69 (27.63 to 27.74)
	IMD=6	2104	61597.09	33.15 (33.10 to 33.21)	3426	73014	45.38 (45.31 to 45.44)	934	41084.17	17.13 (17.08 to 17.18)	1722	46597	27.02 (26.96 to 27.08)
	IMD=7	2211	68599.05	31.71 (31.65 to 31.76)	3614	80856	43.75 (43.69 to 43.81)	916	43967.46	16.05 (16.01 to 16.09)	1618	49208	24.54 (24.49 to 24.60)
	IMD=8	1820	49518.57	36.76 (36.71 to 36.82)	3053	59598	50.77 (50.71 to 50.84)	672	30049.68	17.83 (17.78 to 17.87)	1311	34105	29.60 (29.54 to 29.66)
	IMD=9	1502	39604.41	38.57 (38.51 to 38.62)	2672	47999	55.97 (55.90 to 56.04)	488	22862.33	17.46 (17.41 to 17.51)	1000	25924	30.60 (30.54 to 30.66)
	Most Deprivation	2079	49083.38	43.18 (43.12 to 43.24)	3972	60973	65.45 (65.38 to 65.52)	616	27276.09	19.40 (19.35 to 19.45)	1243	31042	32.90 (32.83 to 32.96)
2018	Least Deprivation	2113	65122.12	31.15 (31.10 to 31.20)	3203	76106	39.62 (39.56 to 39.68)	995	44710.75	17.31 (17.26 to 17.36)	1743	50293	25.88 (25.83 to 25.94)
	IMD=2	2085	64674.92	30.85 (30.80 to 30.90)	3293	78239	39.94 (39.88 to 40.00)	1042	44027.78	17.91 (17.86 to 17.95)	1893	51128	26.88 (26.83 to 26.94)
	IMD=3	2019	59591.04	32.89 (32.84 to 32.94)	3238	71329	43.43 (43.37 to 43.49)	960	38970.45	19.11 (19.07 to 19.16)	1791	44885	29.69 (29.63 to 29.75)
	IMD=4	2353	75422.11	30.34 (30.29 to 30.39)	3795	89581	40.72 (40.66 to 40.78)	1126	48863.5	18.13 (18.09 to 18.18)	2055	56068	27.81 (27.75 to 27.87)
	IMD=5	1955	69369.68	27.51 (27.46 to 27.56)	3203	81909	37.78 (37.72 to 37.84)	1015	45071.09	17.36 (17.31 to 17.41)	1867	51175	27.22 (27.16 to 27.28)
	IMD=6	2096	61334.36	33.15 (33.09 to 33.20)	3353	73995	43.66 (43.60 to 43.72)	933	40701.87	17.34 (17.29 to 17.39)	1761	47103	27.29 (27.24 to 27.35)
	IMD=7	2107	69727.96	29.87 (29.82 to 29.92)	3507	82504	41.77 (41.71 to 41.82)	893	44354.04	15.56 (15.52 to 15.60)	1597	49960	24.00 (23.95 to 24.05)
	IMD=8	1725	49926.05	34.57 (34.51 to 34.62)	2978	60581	48.76 (48.69 to 48.82)	653	30110.62	17.34 (17.29 to 17.38)	1296	34523	28.91 (28.86 to 28.97)
	IMD=9	1472	40622.52	36.94 (36.88 to 37.00)	2609	49250	53.44 (53.37 to 53.51)	497	23050.87	17.62 (17.57 to 17.66)	964	26231	29.05 (28.99 to 29.11)
	Most Deprivation	2165	49510.63	44.86 (44.80 to 44.93)	4074	62253	66.01 (65.94 to 66.09)	590	27205.94	18.71 (18.66 to 18.76)	1263	31428	33.21 (33.15 to 33.28)
2019	Least Deprivation	2002	65233.38	28.93 (28.88 to 28.98)	3153	76673	38.26 (38.20 to 38.31)	1026	44638.33	17.72 (17.67 to 17.77)	1823	50509	26.75 (26.69 to 26.81)
	IMD=2	1874	63434.04	28.39 (28.34 to 28.44)	2978	76613	36.87 (36.82 to 36.93)	1014	42613.95	17.92 (17.87 to 17.97)	1894	49635	27.60 (27.54 to 27.66)
	IMD=3	1888	58647	31.12 (31.07 to 31.17)	3026	70670	40.83 (40.77 to 40.89)	879	38108.09	17.78 (17.73 to 17.82)	1761	44208	29.50 (29.44 to 29.56)
	IMD=4	2287	72774.6	30.73 (30.68 to 30.78)	3634	88790	39.40 (39.35 to 39.46)	1105	46636.3	18.59 (18.54 to 18.64)	2025	55059	27.74 (27.68 to 27.80)
	IMD=5	2030	69853.61	28.42 (28.37 to 28.47)	3255	81967	38.58 (38.52 to 38.64)	1024	44930.56	17.43 (17.39 to 17.48)	1884	51009	27.30 (27.24 to 27.35)

	IMD=6	1929	60873.34	30.59 (30.54 to 30.64)	3122	72844	41.19 (41.13 to 41.24)	917	39999.74	17.20 (17.16 to 17.25)	1767	45936	28.03 (27.98 to 28.09)
	IMD=7	2105	69848.76	29.61 (29.56 to 29.66)	3473	82821	40.89 (40.83 to 40.95)	902	43636.22	15.85 (15.81 to 15.90)	1617	49502	24.36 (24.31 to 24.42)
	IMD=8	1616	48716.98	33.25 (33.20 to 33.31)	2778	58584	47.09 (47.03 to 47.16)	623	28870.96	17.15 (17.10 to 17.19)	1265	32957	29.57 (29.51 to 29.63)
	IMD=9	1467	40920.08	36.52 (36.47 to 36.58)	2527	49557	51.35 (51.28 to 51.42)	527	22867.18	18.85 (18.80 to 18.89)	1019	26145	30.82 (30.76 to 30.88)
	Most Deprivation	1929	47409.89	42.03 (41.97 to 42.09)	3682	58831	63.47 (63.39 to 63.54)	517	25666.28	17.25 (17.20 to 17.29)	1137	29369	31.91 (31.85 to 31.97)
**South Central**													
2004	Least Deprivation	3718	121200.7	29.90 (29.85 to 29.95)	5619	139382	38.95 (38.89 to 39.01)	1213	74767.56	13.52 (13.48 to 13.56)	2031	81554	20.24 (20.19 to 20.29)
	IMD=2	2397	82093.3	28.56 (28.51 to 28.61)	3631	94384	37.30 (37.25 to 37.36)	934	49632.75	15.43 (15.38 to 15.47)	1543	54764	22.38 (22.33 to 22.43)
	IMD=3	1669	52733.43	30.69 (30.64 to 30.74)	2470	60453	39.41 (39.35 to 39.46)	606	31823.32	15.42 (15.38 to 15.46)	1018	34943	23.08 (23.03 to 23.13)
	IMD=4	1736	53449.71	32.12 (32.07 to 32.17)	2666	62229	42.23 (42.17 to 42.29)	541	30883.75	14.39 (14.35 to 14.43)	923	34013	21.48 (21.43 to 21.53)
	IMD=5	1279	38003.81	33.67 (33.62 to 33.73)	1982	44321	44.41 (44.35 to 44.47)	366	20610.76	14.48 (14.44 to 14.52)	728	22774	25.12 (25.07 to 25.18)
	IMD=6	1008	29088.28	34.85 (34.80 to 34.91)	1631	34126	47.47 (47.41 to 47.53)	317	16019.84	15.48 (15.44 to 15.52)	603	17797	25.73 (25.68 to 25.79)
	IMD=7	1011	29178.14	35.97 (35.92 to 36.03)	1625	34166	48.70 (48.63 to 48.76)	295	14810.77	15.75 (15.70 to 15.79)	559	16528	25.95 (25.89 to 26.00)
	IMD=8	746	22280.7	34.81 (34.76 to 34.87)	1207	26045	47.72 (47.66 to 47.78)	182	10858.93	13.49 (13.45 to 13.53)	397	12063	25.87 (25.82 to 25.93)
	IMD=9	578	15865.12	37.21 (37.16 to 37.27)	967	18473	52.91 (52.85 to 52.98)	159	8080.143	15.81 (15.76 to 15.85)	288	8923	25.06 (25.00 to 25.11)
	Most Deprivation	251	6846.442	36.69 (36.63 to 36.74)	383	7837	48.78 (48.72 to 48.85)	70	3413.744	16.46 (16.41 to 16.50)	121	3749	25.10 (25.05 to 25.16)
2005	Least Deprivation	3871	123216.4	30.94 (30.88 to 30.99)	5872	142991	39.86 (39.80 to 39.92)	1264	76625.33	13.90 (13.86 to 13.94)	2173	83975	21.16 (21.11 to 21.21)
	IMD=2	2496	82400.1	29.93 (29.88 to 29.98)	3830	95870	38.98 (38.92 to 39.03)	876	50349.73	14.26 (14.22 to 14.30)	1547	55840	21.99 (21.94 to 22.04)
	IMD=3	1597	53614.11	29.35 (29.30 to 29.40)	2524	62101	39.51 (39.45 to 39.57)	654	32479.84	16.54 (16.49 to 16.58)	1111	35941	24.58 (24.52 to 24.63)
	IMD=4	1729	53413.82	32.38 (32.33 to 32.43)	2746	62670	43.39 (43.33 to 43.45)	527	31192.14	13.85 (13.81 to 13.89)	949	34559	21.78 (21.73 to 21.84)
	IMD=5	1235	38930.87	32.09 (32.04 to 32.14)	2054	45755	45.11 (45.05 to 45.17)	388	20859.71	15.11 (15.06 to 15.15)	739	23197	25.07 (25.01 to 25.12)
	IMD=6	1028	29484.01	35.11 (35.06 to 35.17)	1727	34915	49.30 (49.23 to 49.36)	271	16168.17	13.28 (13.24 to 13.32)	558	18073	23.53 (23.48 to 23.59)
	IMD=7	1048	29439.21	36.90 (36.85 to 36.96)	1747	34860	51.59 (51.53 to 51.66)	281	14948.38	14.97 (14.93 to 15.02)	567	16751	26.19 (26.13 to 26.24)
	IMD=8	775	22612.79	35.26 (35.20 to 35.31)	1277	26711	49.02 (48.96 to 49.09)	169	11062.45	12.43 (12.39 to 12.47)	395	12310	25.55 (25.49 to 25.60)
	IMD=9	582	15918.24	37.57 (37.51 to 37.62)	990	18847	52.89 (52.83 to 52.96)	154	8132.2	15.06 (15.02 to 15.10)	287	9068	24.61 (24.55 to 24.66)
	Most Deprivation	240	6860.871	34.98 (34.93 to 35.04)	413	8046	51.46 (51.40 to 51.53)	56	3445.736	12.98 (12.94 to 13.02)	122	3849	24.31 (24.25 to 24.36)
2006	Least Deprivation	3862	126494.4	29.90 (29.85 to 29.95)	6023	147265	39.55 (39.50 to 39.61)	1284	79601.02	13.54 (13.50 to 13.58)	2174	87219	20.35 (20.30 to 20.40)
	IMD=2	2630	82969.25	31.04 (30.99 to 31.10)	4077	96776	40.94 (40.88 to 40.99)	872	51724.99	13.78 (13.74 to 13.82)	1576	57222	21.81 (21.76 to 21.86)
	IMD=3	1691	54619.26	30.42 (30.37 to 30.47)	2684	63724	40.73 (40.67 to 40.79)	643	33521.36	15.91 (15.87 to 15.96)	1146	37224	24.54 (24.48 to 24.59)
	IMD=4	1789	53781.3	32.87 (32.82 to 32.92)	2819	63533	43.56 (43.50 to 43.62)	538	31968.37	13.89 (13.85 to 13.93)	964	35416	21.75 (21.70 to 21.80)

	IMD=5	1303	39259.39	33.43 (33.37 to 33.48)	2189	46326	47.18 (47.12 to 47.25)	352	21416.87	13.63 (13.59 to 13.67)	710	23827	23.74 (23.68 to 23.79)
	IMD=6	1056	29978.02	35.65 (35.60 to 35.71)	1796	35704	50.49 (50.43 to 50.56)	290	16587.59	14.04 (14.00 to 14.08)	581	18494	24.36 (24.31 to 24.41)
	IMD=7	1044	29591.44	36.24 (36.18 to 36.29)	1810	35469	52.13 (52.07 to 52.20)	256	15352.5	13.41 (13.37 to 13.45)	541	17192	24.39 (24.33 to 24.44)
	IMD=8	799	22989.77	35.99 (35.93 to 36.04)	1361	27276	51.19 (51.13 to 51.26)	174	11358.13	12.55 (12.51 to 12.59)	378	12621	23.84 (23.79 to 23.90)
	IMD=9	536	16240.72	33.94 (33.89 to 33.99)	964	19387	50.44 (50.37 to 50.50)	139	8356.843	13.40 (13.36 to 13.44)	263	9314	22.17 (22.12 to 22.22)
	Most Deprivation	238	7012.055	34.30 (34.24 to 34.35)	428	8313	52.02 (51.95 to 52.08)	61	3533.788	14.15 (14.11 to 14.19)	129	3942	25.85 (25.79 to 25.90)
2007	Least Deprivation	4134	129115.1	31.37 (31.32 to 31.42)	6392	150448	41.14 (41.08 to 41.19)	1260	82013.72	12.93 (12.89 to 12.97)	2248	89937	20.42 (20.37 to 20.47)
	IMD=2	2600	84228.89	30.36 (30.31 to 30.41)	4150	98330	40.96 (40.90 to 41.02)	870	53141.23	13.34 (13.30 to 13.38)	1534	58712	20.74 (20.69 to 20.79)
	IMD=3	1781	55300.96	31.51 (31.46 to 31.57)	2791	64573	41.72 (41.66 to 41.78)	624	34396.16	15.01 (14.97 to 15.06)	1166	38180	24.41 (24.35 to 24.46)
	IMD=4	1920	54289.56	35.07 (35.02 to 35.12)	3026	64438	46.28 (46.22 to 46.34)	558	32772.01	14.06 (14.02 to 14.10)	968	36289	21.49 (21.44 to 21.54)
	IMD=5	1311	39882.62	33.18 (33.13 to 33.23)	2270	47212	48.22 (48.16 to 48.29)	403	22030.1	15.08 (15.03 to 15.12)	761	24454	24.83 (24.78 to 24.89)
	IMD=6	1184	30431.36	39.22 (39.17 to 39.28)	1977	36493	54.24 (54.17 to 54.30)	317	17058.17	15.06 (15.02 to 15.11)	605	19005	24.73 (24.68 to 24.78)
	IMD=7	1057	30041.11	36.58 (36.53 to 36.64)	1877	36062	53.29 (53.22 to 53.35)	319	15815.78	16.23 (16.18 to 16.27)	592	17702	26.15 (26.10 to 26.21)
	IMD=8	808	23715.15	35.15 (35.10 to 35.21)	1413	28246	51.19 (51.12 to 51.25)	214	11896.1	15.16 (15.12 to 15.20)	378	13186	23.41 (23.36 to 23.47)
	IMD=9	630	17012.17	38.46 (38.40 to 38.52)	1117	20232	56.31 (56.24 to 56.38)	157	8839.061	14.59 (14.54 to 14.63)	285	9825	23.04 (22.99 to 23.09)
	Most Deprivation	239	7561.706	31.73 (31.68 to 31.79)	456	8951	51.59 (51.52 to 51.65)	56	3876.986	12.16 (12.12 to 12.20)	123	4292	23.14 (23.08 to 23.19)
2008	Least Deprivation	4156	132131.9	30.73 (30.68 to 30.78)	6422	153021	40.54 (40.48 to 40.60)	1306	84529.61	12.97 (12.93 to 13.01)	2273	92097	20.15 (20.10 to 20.19)
	IMD=2	2657	85835.09	30.26 (30.21 to 30.31)	4246	100041	41.06 (41.00 to 41.12)	933	54594.69	13.90 (13.85 to 13.94)	1626	60045	21.49 (21.44 to 21.54)
	IMD=3	1792	56732.65	31.03 (30.98 to 31.09)	2905	66016	42.70 (42.64 to 42.76)	603	35457.64	14.00 (13.96 to 14.04)	1108	39164	22.49 (22.44 to 22.54)
	IMD=4	1815	55202.66	32.79 (32.74 to 32.84)	2934	65214	44.53 (44.47 to 44.59)	549	33623.05	13.73 (13.69 to 13.77)	983	37006	21.61 (21.56 to 21.66)
	IMD=5	1286	41054.69	31.51 (31.45 to 31.56)	2253	48357	46.52 (46.46 to 46.58)	394	22762.57	14.43 (14.39 to 14.47)	732	25158	23.48 (23.42 to 23.53)
	IMD=6	1126	30765.95	37.33 (37.27 to 37.38)	2001	36871	54.45 (54.38 to 54.52)	312	17437.46	14.41 (14.37 to 14.45)	631	19360	25.39 (25.33 to 25.44)
	IMD=7	1000	30881.75	33.45 (33.40 to 33.51)	1835	36934	50.95 (50.88 to 51.01)	332	16342.13	16.50 (16.46 to 16.55)	604	18206	26.03 (25.98 to 26.09)
	IMD=8	788	24377.39	33.32 (33.27 to 33.37)	1374	29054	48.59 (48.53 to 48.66)	228	12372.58	15.57 (15.53 to 15.62)	417	13694	24.89 (24.84 to 24.95)
	IMD=9	585	17852.92	34.00 (33.95 to 34.05)	1051	21197	50.87 (50.80 to 50.93)	189	9324.244	16.66 (16.62 to 16.71)	309	10334	24.03 (23.97 to 24.08)
	Most Deprivation	261	8016.173	33.02 (32.97 to 33.07)	487	9467	52.01 (51.95 to 52.08)	70	4132.077	13.99 (13.95 to 14.04)	150	4586	26.35 (26.30 to 26.41)
2009	Least Deprivation	4051	134525	29.64 (29.59 to 29.69)	6427	156394	39.87 (39.81 to 39.93)	1393	86998.2	13.36 (13.32 to 13.40)	2330	95059	19.96 (19.91 to 20.01)
	IMD=2	2637	87113.5	29.72 (29.67 to 29.77)	4252	101921	40.46 (40.40 to 40.52)	1003	55995.26	14.61 (14.57 to 14.66)	1707	61655	21.90 (21.85 to 21.95)
	IMD=3	1818	57914.28	30.89 (30.84 to 30.94)	3012	67868	43.00 (42.94 to 43.06)	702	36611.15	15.86 (15.82 to 15.90)	1263	40501	24.90 (24.84 to 24.95)
	IMD=4	1857	56167.04	32.96 (32.91 to 33.01)	3093	66732	45.86 (45.80 to 45.92)	612	34535.09	14.79 (14.74 to 14.83)	1013	38077	21.57 (21.52 to 21.62)

	IMD=5	1420	41887.93	34.36 (34.30 to 34.41)	2450	49688	49.37 (49.30 to 49.43)	404	23473.7	14.27 (14.23 to 14.32)	730	25940	22.71 (22.66 to 22.77)
	IMD=6	1139	31199.13	36.85 (36.79 to 36.90)	1984	37652	52.86 (52.80 to 52.93)	331	17814.26	15.15 (15.11 to 15.19)	619	19871	24.49 (24.44 to 24.55)
	IMD=7	1055	31590.25	34.56 (34.51 to 34.61)	1865	37815	50.59 (50.53 to 50.66)	276	16723.23	13.49 (13.45 to 13.53)	547	18657	23.23 (23.17 to 23.28)
	IMD=8	828	24839.17	34.34 (34.28 to 34.39)	1424	29780	48.75 (48.69 to 48.82)	232	12813.98	15.68 (15.64 to 15.73)	406	14260	23.58 (23.53 to 23.63)
	IMD=9	617	18217.83	34.85 (34.80 to 34.90)	1099	21678	51.96 (51.90 to 52.03)	173	9622.754	15.07 (15.03 to 15.12)	288	10658	21.97 (21.91 to 22.02)
	Most Deprivation	258	8166.727	32.03 (31.97 to 32.08)	479	9634	50.24 (50.17 to 50.30)	77	4263.704	14.65 (14.61 to 14.69)	148	4725	25.55 (25.50 to 25.61)
2010	Least Deprivation	4204	137993	29.66 (29.61 to 29.71)	6614	160337	39.63 (39.57 to 39.68)	1362	89851.24	12.69 (12.65 to 12.73)	2339	98078	19.39 (19.34 to 19.44)
	IMD=2	2768	89272.65	30.71 (30.66 to 30.76)	4475	104377	41.83 (41.77 to 41.88)	1072	57648.34	15.18 (15.14 to 15.22)	1808	63566	22.49 (22.44 to 22.54)
	IMD=3	1849	59394.11	30.62 (30.57 to 30.67)	3012	69657	42.02 (41.96 to 42.08)	649	37835.62	14.17 (14.13 to 14.22)	1200	41928	22.88 (22.83 to 22.93)
	IMD=4	1879	57451.62	32.57 (32.52 to 32.62)	3120	68071	45.13 (45.06 to 45.19)	647	35589.94	15.22 (15.18 to 15.26)	1103	39251	22.81 (22.76 to 22.87)
	IMD=5	1420	43185.57	33.39 (33.34 to 33.44)	2507	51370	49.13 (49.06 to 49.19)	412	24470.47	14.14 (14.10 to 14.18)	763	27028	22.95 (22.90 to 23.00)
	IMD=6	1113	32096.05	35.03 (34.98 to 35.09)	2042	38790	52.67 (52.60 to 52.73)	314	18456.77	13.92 (13.88 to 13.96)	637	20551	24.45 (24.39 to 24.50)
	IMD=7	1048	32716.81	33.18 (33.13 to 33.23)	1895	39147	49.67 (49.61 to 49.73)	350	17382.14	16.58 (16.54 to 16.63)	636	19414	26.26 (26.21 to 26.32)
	IMD=8	858	25762.38	34.51 (34.45 to 34.56)	1478	30772	49.12 (49.06 to 49.19)	236	13401.9	15.01 (14.96 to 15.05)	431	14875	24.09 (24.04 to 24.15)
	IMD=9	573	18824.73	30.88 (30.83 to 30.93)	1071	22383	48.45 (48.39 to 48.52)	195	10034.6	16.29 (16.25 to 16.34)	324	11100	23.65 (23.60 to 23.70)
	Most Deprivation	286	8362.815	35.43 (35.38 to 35.49)	491	9930	50.13 (50.06 to 50.19)	84	4409.733	16.10 (16.06 to 16.15)	152	4899	25.54 (25.48 to 25.59)
2011	Least Deprivation	4310	140750.3	29.86 (29.81 to 29.91)	6812	163464	40.18 (40.12 to 40.23)	1470	92487.45	13.28 (13.24 to 13.32)	2417	100947	19.38 (19.34 to 19.43)
	IMD=2	2895	90997.11	30.99 (30.94 to 31.04)	4680	106278	42.44 (42.38 to 42.50)	974	59086.74	13.41 (13.37 to 13.45)	1724	65246	20.83 (20.78 to 20.88)
	IMD=3	1894	60537.97	30.78 (30.72 to 30.83)	3093	70879	42.43 (42.37 to 42.49)	678	38792.72	14.34 (14.30 to 14.38)	1250	42890	23.17 (23.12 to 23.22)
	IMD=4	2037	58554.61	34.67 (34.62 to 34.73)	3338	69295	47.34 (47.28 to 47.41)	639	36600.92	14.70 (14.66 to 14.75)	1115	40390	22.54 (22.48 to 22.59)
	IMD=5	1471	43765.94	33.74 (33.69 to 33.80)	2528	52167	48.29 (48.23 to 48.36)	448	25141.32	15.05 (15.01 to 15.09)	825	27891	24.16 (24.10 to 24.21)
	IMD=6	1200	32809.22	36.87 (36.81 to 36.92)	2082	39459	52.62 (52.56 to 52.69)	340	19027.48	14.74 (14.70 to 14.78)	646	21154	24.23 (24.17 to 24.28)
	IMD=7	1086	33398.81	33.60 (33.55 to 33.65)	1940	39994	49.60 (49.53 to 49.66)	340	17806.45	15.76 (15.72 to 15.80)	631	19900	25.38 (25.33 to 25.44)
	IMD=8	961	26403.08	37.85 (37.79 to 37.90)	1634	31368	53.20 (53.14 to 53.27)	279	13740.79	17.65 (17.61 to 17.70)	462	15245	25.48 (25.43 to 25.54)
	IMD=9	755	19379.21	40.08 (40.02 to 40.14)	1269	22971	56.01 (55.94 to 56.07)	212	10346.39	17.38 (17.33 to 17.42)	357	11513	25.44 (25.39 to 25.50)
	Most Deprivation	351	8623.072	41.69 (41.63 to 41.75)	580	10263	57.21 (57.14 to 57.28)	89	4589.993	16.29 (16.25 to 16.34)	154	5106	24.84 (24.78 to 24.89)
2012	Least Deprivation	4403	142430.4	30.06 (30.01 to 30.11)	6884	165169	40.03 (39.97 to 40.09)	1538	94649.18	13.47 (13.43 to 13.51)	2478	103098	19.34 (19.29 to 19.39)
	IMD=2	2846	92607.19	30.13 (30.08 to 30.18)	4586	108176	40.93 (40.87 to 40.99)	1091	60490.79	14.62 (14.58 to 14.66)	1812	66590	21.27 (21.22 to 21.32)
	IMD=3	1896	61148.52	30.61 (30.56 to 30.66)	3125	71633	42.45 (42.39 to 42.51)	722	39535.95	14.95 (14.90 to 14.99)	1288	43690	23.35 (23.30 to 23.40)
	IMD=4	2035	59735.55	33.78 (33.73 to 33.83)	3342	71288	46.05 (45.99 to 46.11)	650	37978.03	14.40 (14.36 to 14.45)	1140	41887	22.16 (22.11 to 22.21)

	IMD=5	1411	44247.15	32.15 (32.10 to 32.20)	2450	52688	46.34 (46.28 to 46.40)	454	25651.7	14.86 (14.82 to 14.90)	817	28324	23.53 (23.47 to 23.58)
	IMD=6	1241	33130.34	37.82 (37.77 to 37.88)	2184	40027	54.51 (54.44 to 54.57)	336	19389.47	14.37 (14.33 to 14.41)	635	21526	23.59 (23.54 to 23.64)
	IMD=7	1194	33567.2	36.61 (36.56 to 36.67)	2082	40268	52.68 (52.61 to 52.75)	408	18139.23	18.56 (18.51 to 18.61)	669	20274	26.44 (26.38 to 26.49)
	IMD=8	1053	26832.69	40.50 (40.44 to 40.56)	1794	32046	57.17 (57.10 to 57.23)	273	14124.59	16.79 (16.75 to 16.84)	467	15639	25.16 (25.11 to 25.22)
	IMD=9	834	19434.62	43.65 (43.59 to 43.71)	1391	23322	60.04 (59.97 to 60.11)	233	10539.79	18.62 (18.57 to 18.66)	396	11722	27.65 (27.59 to 27.71)
	Most Deprivation	333	8721.657	38.70 (38.64 to 38.75)	567	10412	54.52 (54.45 to 54.59)	118	4671.192	21.55 (21.50 to 21.60)	193	5219	30.64 (30.58 to 30.71)
2013	Least Deprivation	4497	142924.1	30.65 (30.60 to 30.70)	7174	166356	41.48 (41.42 to 41.54)	1572	95672.31	13.55 (13.51 to 13.59)	2559	104698	19.52 (19.48 to 19.57)
	IMD=2	2898	93006.82	30.48 (30.43 to 30.53)	4710	109069	41.67 (41.61 to 41.73)	1151	61135.84	15.22 (15.18 to 15.26)	1923	67633	22.13 (22.08 to 22.18)
	IMD=3	1922	61128.84	30.95 (30.90 to 31.00)	3143	72015	42.37 (42.31 to 42.43)	812	39783.09	16.74 (16.70 to 16.79)	1386	44218	24.77 (24.72 to 24.83)
	IMD=4	2043	59746.9	33.94 (33.89 to 34.00)	3386	71166	46.68 (46.62 to 46.74)	700	38208.3	15.28 (15.24 to 15.32)	1210	42328	23.17 (23.11 to 23.22)
	IMD=5	1537	44546.93	34.71 (34.66 to 34.77)	2568	53147	48.11 (48.04 to 48.17)	486	26003.12	15.78 (15.74 to 15.82)	815	28854	23.04 (22.99 to 23.09)
	IMD=6	1287	33125.52	39.51 (39.45 to 39.57)	2240	40095	55.98 (55.91 to 56.05)	367	19534.59	15.66 (15.61 to 15.70)	673	21777	24.92 (24.87 to 24.98)
	IMD=7	1145	33545.73	35.19 (35.14 to 35.25)	2019	40621	50.64 (50.57 to 50.70)	411	18300.55	18.76 (18.72 to 18.81)	726	20619	28.36 (28.30 to 28.42)
	IMD=8	980	26895.67	37.32 (37.26 to 37.38)	1756	32534	54.95 (54.88 to 55.02)	277	14271.04	16.80 (16.76 to 16.85)	504	16004	26.57 (26.51 to 26.62)
	IMD=9	721	19484.29	38.08 (38.02 to 38.14)	1297	23526	55.89 (55.82 to 55.96)	278	10610.61	22.01 (21.96 to 22.06)	466	11950	31.89 (31.83 to 31.95)
	Most Deprivation	288	8796.915	33.22 (33.17 to 33.27)	538	10568	51.14 (51.08 to 51.21)	150	4650.713	27.20 (27.14 to 27.25)	237	5276	36.94 (36.87 to 37.01)
2014	Least Deprivation	4424	143458.5	30.12 (30.07 to 30.17)	7071	167579	40.50 (40.44 to 40.56)	1665	96788.71	14.07 (14.03 to 14.11)	2683	106212	20.04 (20.00 to 20.09)
	IMD=2	3056	94016.49	31.96 (31.91 to 32.01)	4882	110520	42.79 (42.73 to 42.85)	1160	61766.73	15.16 (15.12 to 15.20)	1983	68525	22.51 (22.45 to 22.56)
	IMD=3	1971	61411.67	31.30 (31.24 to 31.35)	3149	72291	42.04 (41.98 to 42.10)	800	39970.51	16.40 (16.36 to 16.45)	1420	44564	25.09 (25.03 to 25.14)
	IMD=4	2035	60036.76	33.52 (33.47 to 33.57)	3441	71699	47.00 (46.93 to 47.06)	734	38565.85	15.84 (15.79 to 15.88)	1241	42768	23.34 (23.29 to 23.40)
	IMD=5	1592	44848.75	35.60 (35.54 to 35.65)	2655	53549	49.18 (49.12 to 49.25)	529	26266.64	16.90 (16.86 to 16.95)	873	29229	24.29 (24.23 to 24.34)
	IMD=6	1267	33173	38.42 (38.36 to 38.47)	2210	40348	54.39 (54.32 to 54.46)	355	19695.66	14.97 (14.93 to 15.01)	707	22023	25.73 (25.67 to 25.79)
	IMD=7	1097	33764.78	33.63 (33.58 to 33.68)	2011	40929	50.18 (50.12 to 50.25)	388	18488.37	17.52 (17.47 to 17.57)	681	20871	26.21 (26.15 to 26.27)
	IMD=8	1039	26818.44	39.51 (39.45 to 39.56)	1771	32814	54.62 (54.56 to 54.69)	328	14455.13	19.49 (19.44 to 19.54)	530	16279	27.29 (27.23 to 27.35)
	IMD=9	765	19602.93	40.08 (40.02 to 40.14)	1343	23811	56.87 (56.80 to 56.94)	288	10641.76	22.96 (22.91 to 23.01)	473	12100	32.26 (32.20 to 32.32)
	Most Deprivation	318	8889.865	36.46 (36.40 to 36.51)	566	10669	53.42 (53.36 to 53.49)	141	4665.654	25.58 (25.52 to 25.63)	227	5322	35.22 (35.15 to 35.28)
2015	Least Deprivation	4340	145512.9	29.02 (28.97 to 29.07)	6902	169006	39.07 (39.02 to 39.13)	1760	98235.95	14.63 (14.58 to 14.67)	2834	107646	20.81 (20.76 to 20.86)
	IMD=2	3062	95038.51	31.43 (31.38 to 31.49)	4840	111706	41.91 (41.85 to 41.97)	1235	62469.38	15.82 (15.77 to 15.86)	2100	69303	23.44 (23.38 to 23.49)
	IMD=3	1899	62219.78	29.93 (29.88 to 29.98)	3051	73121	40.36 (40.30 to 40.42)	798	40641.03	15.96 (15.91 to 16.00)	1391	45299	24.08 (24.02 to 24.13)
	IMD=4	2082	61043.19	33.70 (33.65 to 33.75)	3434	72353	46.42 (46.35 to 46.48)	745	39142.21	15.69 (15.65 to 15.73)	1330	43397	24.58 (24.53 to 24.64)

	IMD=5	1573	44894.63	35.28 (35.22 to 35.33)	2579	54107	47.48 (47.41 to 47.54)	522	26702.43	16.31 (16.26 to 16.35)	852	29787	23.31 (23.25 to 23.36)
	IMD=6	1171	33605.54	35.25 (35.20 to 35.30)	2131	40682	52.17 (52.10 to 52.23)	421	19916.51	17.62 (17.58 to 17.67)	776	22214	28.05 (27.99 to 28.11)
	IMD=7	1141	34213.69	34.04 (33.99 to 34.09)	1993	41157	49.01 (48.94 to 49.07)	379	18842.28	16.76 (16.71 to 16.80)	661	21231	25.02 (24.96 to 25.07)
	IMD=8	1056	27208.19	39.60 (39.54 to 39.66)	1827	32932	55.96 (55.89 to 56.03)	317	14743.48	18.56 (18.51 to 18.61)	559	16516	28.36 (28.30 to 28.42)
	IMD=9	804	19856.29	41.54 (41.48 to 41.60)	1409	24092	59.00 (58.93 to 59.07)	265	10748.57	21.26 (21.21 to 21.32)	478	12239	32.44 (32.38 to 32.50)
	Most Deprivation	309	9050.606	34.36 (34.31 to 34.42)	517	10798	47.89 (47.83 to 47.95)	120	4703.551	21.90 (21.85 to 21.95)	208	5403	31.97 (31.91 to 32.04)
2016	Least Deprivation	4118	147832.7	27.00 (26.96 to 27.05)	6580	171764	36.55 (36.50 to 36.61)	1807	99979.34	14.64 (14.60 to 14.68)	2905	110010	20.77 (20.72 to 20.82)
	IMD=2	2954	96583.71	29.81 (29.76 to 29.86)	4641	113382	39.53 (39.47 to 39.59)	1332	63527.69	16.79 (16.74 to 16.83)	2204	70436	24.19 (24.13 to 24.24)
	IMD=3	1922	63541.21	29.89 (29.84 to 29.94)	3039	74117	39.94 (39.88 to 40.00)	861	41667.18	16.83 (16.78 to 16.87)	1438	46282	24.30 (24.24 to 24.35)
	IMD=4	2034	62119.44	32.35 (32.30 to 32.40)	3428	73600	45.58 (45.52 to 45.64)	750	39904.7	15.56 (15.51 to 15.60)	1355	44220	24.48 (24.43 to 24.54)
	IMD=5	1434	45449.99	31.93 (31.88 to 31.98)	2436	54145	44.78 (44.71 to 44.84)	557	27139.35	17.14 (17.10 to 17.19)	906	30141	24.29 (24.23 to 24.34)
	IMD=6	1178	34224.18	34.54 (34.49 to 34.59)	2117	41065	51.05 (50.98 to 51.11)	427	20278.89	17.60 (17.55 to 17.64)	781	22608	27.83 (27.77 to 27.89)
	IMD=7	1103	34788.75	32.59 (32.54 to 32.64)	1901	41557	46.51 (46.45 to 46.57)	442	19172.18	19.44 (19.40 to 19.49)	704	21547	26.35 (26.29 to 26.40)
	IMD=8	988	27840.95	36.70 (36.65 to 36.76)	1719	33613	51.78 (51.71 to 51.84)	341	15036.9	19.94 (19.89 to 19.99)	620	16922	30.81 (30.75 to 30.87)
	IMD=9	756	20287.45	38.20 (38.14 to 38.25)	1329	24465	54.88 (54.81 to 54.94)	263	10906.45	20.52 (20.47 to 20.57)	489	12380	32.62 (32.56 to 32.68)
	Most Deprivation	277	9404.003	30.15 (30.10 to 30.20)	487	11050	44.42 (44.36 to 44.48)	110	4820.55	19.20 (19.16 to 19.25)	205	5508	30.55 (30.49 to 30.61)
2017	Least Deprivation	4067	150551.3	26.32 (26.27 to 26.36)	6295	173591	34.87 (34.81 to 34.92)	1828	101322.8	14.61 (14.57 to 14.66)	2958	111547	20.76 (20.71 to 20.81)
	IMD=2	2727	98324.5	27.17 (27.12 to 27.22)	4321	114738	36.59 (36.54 to 36.65)	1291	64546.12	15.99 (15.94 to 16.03)	2131	71774	22.90 (22.85 to 22.96)
	IMD=3	1879	65228.7	28.34 (28.29 to 28.39)	2963	75810	37.95 (37.89 to 38.00)	965	42653.06	18.23 (18.18 to 18.27)	1598	47513	26.16 (26.11 to 26.22)
	IMD=4	2067	63573.58	32.17 (32.12 to 32.22)	3366	74962	43.89 (43.83 to 43.96)	819	40627.41	16.67 (16.62 to 16.71)	1400	45138	24.72 (24.66 to 24.77)
	IMD=5	1542	46374.6	33.40 (33.35 to 33.45)	2452	55013	44.22 (44.16 to 44.28)	511	27596.19	15.43 (15.38 to 15.47)	879	30735	23.14 (23.09 to 23.19)
	IMD=6	1236	34895.24	35.82 (35.76 to 35.87)	2081	41809	49.48 (49.42 to 49.54)	416	20450.18	16.91 (16.86 to 16.95)	773	23009	27.01 (26.95 to 27.07)
	IMD=7	1182	35443.35	34.23 (34.18 to 34.28)	1974	42026	47.63 (47.56 to 47.69)	432	19401.87	18.47 (18.42 to 18.52)	733	21794	26.93 (26.87 to 26.99)
	IMD=8	1014	28543	36.29 (36.24 to 36.35)	1722	34172	50.95 (50.89 to 51.02)	330	15347.75	18.70 (18.65 to 18.75)	587	17202	28.82 (28.76 to 28.88)
	IMD=9	715	20670.54	35.43 (35.38 to 35.49)	1238	24891	50.08 (50.01 to 50.14)	268	11129.89	20.61 (20.56 to 20.66)	471	12664	30.77 (30.71 to 30.83)
	Most Deprivation	330	9777.779	34.71 (34.65 to 34.76)	522	11474	46.15 (46.09 to 46.22)	123	4983.858	20.92 (20.87 to 20.97)	222	5682	32.10 (32.04 to 32.16)
2018	Least Deprivation	3976	153420.2	24.95 (24.90 to 24.99)	6003	176541	32.43 (32.38 to 32.48)	1819	102737.3	14.22 (14.18 to 14.26)	2932	113482	20.06 (20.01 to 20.11)
	IMD=2	2823	102338.5	26.93 (26.88 to 26.98)	4311	118534	35.12 (35.07 to 35.17)	1373	66357.34	16.46 (16.41 to 16.50)	2303	73968	23.89 (23.84 to 23.94)
	IMD=3	1872	68109.61	26.94 (26.89 to 26.99)	2959	79147	36.34 (36.29 to 36.40)	910	44310.68	16.38 (16.34 to 16.43)	1547	49734	24.07 (24.01 to 24.12)
	IMD=4	1956	66394.69	29.24 (29.19 to 29.29)	3111	78205	39.01 (38.96 to 39.07)	790	42032.24	15.40 (15.36 to 15.44)	1372	46918	23.24 (23.19 to 23.30)

	IMD=5	1474	48473.63	30.56 (30.51 to 30.61)	2397	57160	41.59 (41.53 to 41.65)	550	28698.29	15.85 (15.81 to 15.90)	917	31967	23.01 (22.96 to 23.06)
	IMD=6	1196	35886.26	33.71 (33.65 to 33.76)	2033	42939	47.23 (47.17 to 47.29)	395	20806.46	15.74 (15.70 to 15.78)	748	23456	25.63 (25.57 to 25.68)
	IMD=7	1131	36283.35	32.05 (31.99 to 32.10)	1854	43387	43.51 (43.45 to 43.57)	433	19596.1	18.16 (18.11 to 18.21)	751	22213	26.97 (26.91 to 27.02)
	IMD=8	967	29316.65	33.93 (33.88 to 33.98)	1679	35083	48.61 (48.55 to 48.67)	329	15617.28	18.32 (18.28 to 18.37)	597	17614	28.33 (28.27 to 28.38)
	IMD=9	793	21146.66	38.48 (38.43 to 38.54)	1342	25439	53.27 (53.20 to 53.33)	292	11252.29	22.34 (22.28 to 22.39)	495	12876	31.57 (31.51 to 31.64)
	Most Deprivation	387	10033.02	39.49 (39.43 to 39.54)	611	11805	52.25 (52.18 to 52.32)	140	5086.979	23.87 (23.81 to 23.92)	222	5797	31.76 (31.69 to 31.82)
2019	Least Deprivation	3930	154064.6	24.67 (24.62 to 24.71)	5816	177836	31.15 (31.10 to 31.20)	2132	102466.1	16.62 (16.58 to 16.67)	3276	114311	22.15 (22.10 to 22.20)
	IMD=2	2757	103667.1	25.83 (25.78 to 25.88)	4225	120806	33.68 (33.63 to 33.74)	1512	66527.01	17.89 (17.84 to 17.94)	2492	75077	25.21 (25.15 to 25.26)
	IMD=3	1914	68862.15	27.11 (27.07 to 27.16)	2917	79684	35.41 (35.36 to 35.47)	1005	44423.82	17.96 (17.91 to 18.01)	1669	49932	25.59 (25.54 to 25.65)
	IMD=4	2113	67312.35	31.15 (31.10 to 31.20)	3314	79464	40.91 (40.85 to 40.97)	907	42060.58	17.57 (17.52 to 17.61)	1510	47399	25.09 (25.03 to 25.14)
	IMD=5	1509	49442.88	30.53 (30.48 to 30.58)	2392	57964	40.78 (40.72 to 40.83)	663	28990.92	18.84 (18.79 to 18.89)	1057	32315	26.11 (26.05 to 26.16)
	IMD=6	1220	36575.06	33.81 (33.76 to 33.86)	2042	43557	46.83 (46.77 to 46.90)	458	21037.52	18.09 (18.05 to 18.14)	810	23670	27.45 (27.40 to 27.51)
	IMD=7	1152	36943.43	32.19 (32.14 to 32.24)	1867	43654	43.70 (43.64 to 43.76)	441	19724.03	18.41 (18.36 to 18.46)	757	22220	27.04 (26.98 to 27.10)
	IMD=8	980	30157.1	33.18 (33.13 to 33.23)	1648	35841	46.44 (46.38 to 46.50)	368	15905.62	20.26 (20.21 to 20.31)	632	17955	29.37 (29.32 to 29.43)
	IMD=9	752	21673.51	36.05 (36.00 to 36.11)	1273	25805	50.06 (50.00 to 50.13)	298	11295.27	22.01 (21.96 to 22.06)	490	12891	30.80 (30.74 to 30.86)
	Most Deprivation	331	10211.25	33.27 (33.22 to 33.33)	544	11995	45.80 (45.74 to 45.86)	120	5145.084	19.79 (19.74 to 19.84)	210	5882	29.42 (29.36 to 29.48)
**London**													
2004	Least Deprivation	603	22305.87	25.93 (25.88 to 25.98)	829	25079	31.43 (31.38 to 31.48)	218	13509.36	13.25 (13.21 to 13.29)	333	14635	18.18 (18.13 to 18.22)
	IMD=2	995	36019.13	27.40 (27.36 to 27.45)	1399	40826	33.52 (33.46 to 33.57)	349	20267.28	14.33 (14.29 to 14.37)	614	22296	22.14 (22.09 to 22.19)
	IMD=3	1254	44894.91	28.03 (27.98 to 28.08)	1799	51096	35.03 (34.97 to 35.08)	473	24894.94	15.96 (15.92 to 16.00)	841	27542	24.66 (24.61 to 24.72)
	IMD=4	1357	45771.12	29.92 (29.87 to 29.97)	2017	53346	37.51 (37.46 to 37.57)	510	25224.1	17.04 (17.00 to 17.09)	870	28162	25.11 (25.06 to 25.17)
	IMD=5	1574	52707.39	30.11 (30.06 to 30.16)	2389	61230	38.84 (38.78 to 38.90)	575	28198.9	16.94 (16.90 to 16.99)	1053	31594	26.58 (26.53 to 26.64)
	IMD=6	1759	60713.52	29.94 (29.89 to 29.99)	2686	70238	38.95 (38.90 to 39.01)	596	30907.44	17.01 (16.96 to 17.05)	1111	34299	27.25 (27.19 to 27.30)
	IMD=7	1946	69085.91	29.21 (29.16 to 29.26)	2925	79859	37.33 (37.27 to 37.39)	638	33772.45	16.29 (16.25 to 16.34)	1108	37728	24.32 (24.27 to 24.38)
	IMD=8	2238	76932.45	30.49 (30.44 to 30.54)	3378	89201	39.19 (39.14 to 39.25)	737	35870.91	17.47 (17.43 to 17.52)	1357	40313	27.57 (27.51 to 27.63)
	IMD=9	2557	85373.35	31.81 (31.76 to 31.86)	4050	99043	42.54 (42.48 to 42.60)	778	38372.64	17.46 (17.42 to 17.51)	1434	43246	27.39 (27.33 to 27.44)
	Most Deprivation	665	23863.14	30.12 (30.07 to 30.17)	1041	27456	39.68 (39.62 to 39.73)	188	10166.2	15.64 (15.60 to 15.69)	316	11373	23.04 (22.99 to 23.09)
2005	Least Deprivation	565	21944.39	25.13 (25.09 to 25.18)	839	24906	32.50 (32.45 to 32.55)	197	13448.43	12.08 (12.04 to 12.12)	321	14619	17.50 (17.46 to 17.55)
	IMD=2	1032	35531.64	29.04 (28.99 to 29.09)	1509	40590	36.48 (36.43 to 36.54)	351	20158.01	14.45 (14.41 to 14.49)	653	22215	23.48 (23.43 to 23.54)
	IMD=3	1337	44577.32	30.12 (30.07 to 30.17)	1971	51070	38.35 (38.30 to 38.41)	476	24829.92	16.28 (16.24 to 16.33)	880	27480	26.19 (26.13 to 26.25)

	IMD=4	1348	45503.31	30.13 (30.08 to 30.18)	2149	53012	40.65 (40.59 to 40.71)	420	25303.46	14.12 (14.08 to 14.16)	796	28091	23.17 (23.12 to 23.22)
	IMD=5	1528	52822.38	29.59 (29.54 to 29.64)	2467	61621	40.11 (40.05 to 40.16)	550	28342.6	16.19 (16.15 to 16.24)	1035	31829	25.88 (25.82 to 25.93)
	IMD=6	1759	60545.83	30.30 (30.25 to 30.35)	2744	70679	39.95 (39.89 to 40.01)	510	31014.14	14.54 (14.49 to 14.58)	1003	34628	24.75 (24.70 to 24.81)
	IMD=7	1907	68843.09	28.80 (28.75 to 28.85)	3009	80091	38.50 (38.45 to 38.56)	516	33846.24	13.38 (13.34 to 13.42)	1044	37738	23.29 (23.24 to 23.34)
	IMD=8	2257	77053.7	30.90 (30.85 to 30.95)	3667	90181	42.39 (42.33 to 42.45)	668	35963.73	16.15 (16.11 to 16.20)	1402	40525	28.88 (28.82 to 28.94)
	IMD=9	2795	85191.46	35.08 (35.03 to 35.13)	4482	100429	46.76 (46.70 to 46.82)	695	38696.85	15.51 (15.46 to 15.55)	1456	43812	27.89 (27.83 to 27.95)
	Most Deprivation	789	23755.77	36.03 (35.97 to 36.08)	1243	27845	47.51 (47.44 to 47.57)	171	10179.41	14.91 (14.87 to 14.96)	334	11458	24.99 (24.93 to 25.04)
2006	Least Deprivation	639	22061.16	28.45 (28.40 to 28.50)	931	25249	35.73 (35.67 to 35.78)	181	13584.41	11.04 (11.00 to 11.07)	320	14836	17.23 (17.18 to 17.27)
	IMD=2	1057	35702.13	29.59 (29.54 to 29.63)	1603	41275	38.11 (38.05 to 38.17)	292	20463.53	11.77 (11.73 to 11.81)	620	22684	21.98 (21.93 to 22.03)
	IMD=3	1297	44587.68	29.18 (29.13 to 29.23)	2044	51803	39.15 (39.09 to 39.21)	408	25066.13	13.91 (13.87 to 13.95)	850	28015	24.89 (24.83 to 24.94)
	IMD=4	1415	45541.43	31.43 (31.38 to 31.48)	2283	53806	42.38 (42.32 to 42.44)	414	25518.12	13.95 (13.91 to 13.99)	792	28586	22.77 (22.72 to 22.82)
	IMD=5	1520	53168.71	29.39 (29.34 to 29.44)	2550	62883	40.99 (40.93 to 41.05)	499	28477.43	14.67 (14.63 to 14.72)	975	32228	24.51 (24.46 to 24.56)
	IMD=6	1731	60242.42	30.13 (30.08 to 30.18)	2915	72428	41.68 (41.62 to 41.74)	472	30671.66	13.58 (13.54 to 13.62)	1044	35276	25.38 (25.32 to 25.43)
	IMD=7	1943	68087.73	30.05 (30.00 to 30.10)	3160	82017	39.69 (39.63 to 39.74)	540	33416.94	14.27 (14.23 to 14.31)	1042	38536	22.90 (22.85 to 22.95)
	IMD=8	2234	77073.96	31.11 (31.06 to 31.16)	3853	92371	43.81 (43.75 to 43.87)	536	35861.62	13.21 (13.17 to 13.25)	1248	41353	25.44 (25.38 to 25.49)
	IMD=9	2868	86552.36	35.52 (35.46 to 35.57)	4872	103271	49.51 (49.45 to 49.57)	687	39371.7	15.37 (15.32 to 15.41)	1430	44761	27.14 (27.08 to 27.20)
	Most Deprivation	711	24081.75	32.55 (32.50 to 32.60)	1270	28564	47.53 (47.47 to 47.59)	156	10427.2	13.53 (13.49 to 13.57)	327	11781	24.29 (24.24 to 24.34)
2007	Least Deprivation	622	22087.65	27.24 (27.20 to 27.29)	964	25462	36.34 (36.29 to 36.40)	202	13782.47	12.14 (12.10 to 12.18)	364	15035	19.51 (19.46 to 19.55)
	IMD=2	1039	35756.87	28.89 (28.84 to 28.94)	1638	41572	38.55 (38.49 to 38.60)	317	20715.21	12.69 (12.66 to 12.73)	660	22973	23.01 (22.95 to 23.06)
	IMD=3	1350	44588.49	30.32 (30.27 to 30.37)	2149	52119	40.71 (40.65 to 40.77)	437	25442.55	14.68 (14.64 to 14.73)	936	28412	27.20 (27.14 to 27.26)
	IMD=4	1378	45441.46	30.77 (30.72 to 30.82)	2345	53671	43.71 (43.65 to 43.77)	404	25774.18	13.42 (13.38 to 13.46)	834	28767	23.94 (23.89 to 23.99)
	IMD=5	1566	53306.64	30.23 (30.18 to 30.28)	2630	63164	42.11 (42.05 to 42.17)	461	28792.79	13.61 (13.57 to 13.65)	972	32475	24.31 (24.25 to 24.36)
	IMD=6	1774	61023.53	30.48 (30.43 to 30.53)	3007	72043	43.00 (42.94 to 43.06)	499	31374.9	14.14 (14.10 to 14.19)	1103	35141	26.80 (26.75 to 26.86)
	IMD=7	1879	68797.26	28.66 (28.61 to 28.71)	3154	81180	40.38 (40.32 to 40.43)	526	33789.44	13.72 (13.68 to 13.77)	1056	37852	23.84 (23.78 to 23.89)
	IMD=8	2305	78144.56	31.15 (31.10 to 31.21)	3919	92588	44.07 (44.01 to 44.13)	656	36514.65	15.95 (15.91 to 16.00)	1387	41355	28.50 (28.44 to 28.56)
	IMD=9	2916	88014.01	35.63 (35.58 to 35.69)	5242	105737	52.19 (52.12 to 52.25)	659	40296.48	14.68 (14.64 to 14.72)	1462	45673	27.64 (27.58 to 27.70)
	Most Deprivation	802	24283.72	36.03 (35.97 to 36.08)	1400	28837	51.51 (51.44 to 51.57)	196	10686.98	16.57 (16.52 to 16.61)	380	11983	27.72 (27.66 to 27.78)
2008	Least Deprivation	595	22247.2	26.01 (25.96 to 26.06)	942	25601	35.30 (35.24 to 35.35)	224	13989.78	13.35 (13.31 to 13.39)	386	15242	20.42 (20.37 to 20.47)
	IMD=2	1062	36189.59	29.08 (29.03 to 29.13)	1714	42198	39.82 (39.76 to 39.87)	325	21084.96	13.18 (13.14 to 13.22)	658	23351	22.97 (22.92 to 23.02)
	IMD=3	1343	45068.75	29.83 (29.78 to 29.88)	2217	52727	41.40 (41.34 to 41.46)	433	26020.23	14.35 (14.31 to 14.39)	907	28947	26.00 (25.94 to 26.05)

	IMD=4	1450	46095.75	31.68 (31.63 to 31.73)	2412	54566	44.05 (43.99 to 44.12)	453	26324.16	14.73 (14.69 to 14.77)	874	29259	24.71 (24.65 to 24.76)
	IMD=5	1699	54103.16	32.28 (32.23 to 32.33)	2851	64048	44.77 (44.71 to 44.83)	512	29376.32	14.89 (14.85 to 14.93)	1025	32877	25.61 (25.56 to 25.67)
	IMD=6	1859	62755.28	30.80 (30.75 to 30.85)	3166	74461	43.86 (43.80 to 43.92)	489	32446.7	13.48 (13.44 to 13.52)	1065	36325	25.36 (25.31 to 25.42)
	IMD=7	2066	70657.77	30.52 (30.47 to 30.57)	3514	83424	43.64 (43.58 to 43.70)	557	34875.84	14.38 (14.34 to 14.42)	1129	38873	25.25 (25.19 to 25.30)
	IMD=8	2438	80781.89	32.36 (32.31 to 32.41)	4322	96102	47.30 (47.23 to 47.36)	666	37837.8	15.68 (15.63 to 15.72)	1436	42697	29.04 (28.98 to 29.10)
	IMD=9	3009	90122.86	35.73 (35.67 to 35.78)	5559	108987	53.60 (53.53 to 53.67)	731	41687.14	15.71 (15.67 to 15.75)	1513	47073	27.95 (27.89 to 28.01)
	Most Deprivation	794	24582.7	35.57 (35.51 to 35.62)	1470	29682	52.63 (52.57 to 52.70)	159	11086.75	13.45 (13.41 to 13.49)	376	12468	27.34 (27.28 to 27.40)
2009	Least Deprivation	632	22132.18	27.74 (27.69 to 27.78)	992	25875	36.70 (36.65 to 36.76)	230	14086.58	13.63 (13.59 to 13.67)	395	15493	20.58 (20.53 to 20.63)
	IMD=2	1079	35904.01	30.10 (30.05 to 30.15)	1751	42718	40.48 (40.42 to 40.54)	341	21291.3	13.41 (13.37 to 13.45)	684	23788	23.28 (23.23 to 23.34)
	IMD=3	1462	44960.7	32.59 (32.53 to 32.64)	2356	53354	43.64 (43.58 to 43.70)	425	26261.81	13.89 (13.85 to 13.93)	913	29403	25.69 (25.63 to 25.75)
	IMD=4	1489	46492.75	32.30 (32.25 to 32.35)	2544	55006	45.90 (45.83 to 45.96)	431	26680.13	13.57 (13.53 to 13.61)	887	29557	24.56 (24.50 to 24.61)
	IMD=5	1772	54851.63	33.14 (33.09 to 33.19)	3037	65130	47.02 (46.96 to 47.08)	541	30015.36	15.42 (15.38 to 15.46)	1098	33472	27.04 (26.98 to 27.09)
	IMD=6	2075	63958.38	33.90 (33.85 to 33.95)	3511	75689	47.86 (47.79 to 47.92)	578	33276.21	15.71 (15.67 to 15.76)	1199	37056	28.25 (28.19 to 28.31)
	IMD=7	2243	72170	32.82 (32.76 to 32.87)	3762	85579	45.70 (45.64 to 45.77)	558	35726.96	14.20 (14.16 to 14.25)	1172	39817	25.70 (25.64 to 25.75)
	IMD=8	2688	82635.35	34.56 (34.51 to 34.61)	4806	98496	51.06 (50.99 to 51.12)	669	39086.06	15.70 (15.66 to 15.74)	1452	43987	29.03 (28.97 to 29.09)
	IMD=9	3370	91909.62	39.23 (39.18 to 39.29)	6135	111308	57.59 (57.52 to 57.66)	808	43132.95	17.09 (17.05 to 17.14)	1643	48703	29.78 (29.72 to 29.84)
	Most Deprivation	837	25113.75	35.25 (35.19 to 35.30)	1610	30404	54.66 (54.59 to 54.73)	209	11581	16.55 (16.51 to 16.60)	414	13024	28.48 (28.42 to 28.54)
2010	Least Deprivation	630	22426.24	27.25 (27.20 to 27.29)	985	25954	36.33 (36.28 to 36.39)	207	14251.09	12.11 (12.07 to 12.15)	390	15607	20.02 (19.97 to 20.07)
	IMD=2	1038	36318.19	28.32 (28.27 to 28.37)	1798	42487	41.17 (41.11 to 41.23)	309	21740.04	12.09 (12.05 to 12.13)	673	23972	22.94 (22.88 to 22.99)
	IMD=3	1493	45513.7	32.98 (32.92 to 33.03)	2514	53663	46.41 (46.35 to 46.47)	425	26853.55	13.74 (13.70 to 13.78)	985	29805	27.67 (27.61 to 27.72)
	IMD=4	1541	47539.73	32.80 (32.74 to 32.85)	2695	56589	47.37 (47.31 to 47.44)	443	27351.76	13.85 (13.81 to 13.89)	941	30440	25.51 (25.46 to 25.57)
	IMD=5	1835	56116.32	33.65 (33.60 to 33.71)	3157	67035	47.64 (47.58 to 47.70)	488	30748.04	13.63 (13.59 to 13.67)	1085	34387	26.10 (26.04 to 26.15)
	IMD=6	2193	66037.7	34.61 (34.55 to 34.66)	3767	78787	49.21 (49.14 to 49.27)	537	34450.69	13.93 (13.89 to 13.98)	1202	38438	27.32 (27.26 to 27.38)
	IMD=7	2398	74539.1	33.96 (33.90 to 34.01)	4108	88655	48.20 (48.14 to 48.26)	640	37076.65	15.53 (15.48 to 15.57)	1261	41313	26.75 (26.69 to 26.80)
	IMD=8	2800	85609.58	34.62 (34.57 to 34.68)	5024	102411	51.29 (51.23 to 51.36)	777	40532.38	17.65 (17.60 to 17.69)	1659	45625	32.36 (32.30 to 32.42)
	IMD=9	3439	94977.19	38.59 (38.53 to 38.65)	6461	115842	58.48 (58.41 to 58.55)	793	44854.32	16.32 (16.28 to 16.37)	1724	50670	30.49 (30.43 to 30.55)
	Most Deprivation	935	25542.23	38.41 (38.35 to 38.46)	1794	31346	59.10 (59.03 to 59.17)	216	11950.6	16.25 (16.21 to 16.30)	447	13515	29.80 (29.74 to 29.86)
2011	Least Deprivation	643	22825.92	27.42 (27.37 to 27.47)	960	26518	34.86 (34.80 to 34.91)	230	14546.08	13.18 (13.14 to 13.22)	409	15991	20.52 (20.47 to 20.57)
	IMD=2	1103	36769.55	30.03 (29.98 to 30.08)	1872	43417	42.40 (42.34 to 42.46)	345	22193.86	13.19 (13.15 to 13.23)	715	24532	23.80 (23.74 to 23.85)
	IMD=3	1471	46183.65	31.88 (31.83 to 31.93)	2548	54964	45.83 (45.77 to 45.90)	446	27383.95	14.12 (14.08 to 14.16)	1004	30543	27.48 (27.43 to 27.54)

	IMD=4	1613	47896.73	33.99 (33.94 to 34.05)	2885	57866	49.59 (49.52 to 49.65)	435	27918.13	13.31 (13.27 to 13.35)	914	31156	24.23 (24.17 to 24.28)
	IMD=5	1890	56729.48	33.95 (33.89 to 34.00)	3358	68960	49.00 (48.94 to 49.07)	583	31422.32	15.98 (15.94 to 16.03)	1169	35378	27.44 (27.39 to 27.50)
	IMD=6	2198	67042.55	34.23 (34.18 to 34.29)	3877	81024	49.46 (49.40 to 49.53)	604	35389.95	15.62 (15.58 to 15.66)	1258	39644	28.02 (27.96 to 28.07)
	IMD=7	2450	76254.21	33.91 (33.86 to 33.97)	4396	92335	49.57 (49.50 to 49.63)	634	38430.43	15.19 (15.15 to 15.23)	1300	43070	26.93 (26.87 to 26.98)
	IMD=8	3012	87509.79	36.75 (36.70 to 36.81)	5440	106148	53.93 (53.86 to 53.99)	778	41703.76	17.11 (17.07 to 17.16)	1617	47146	30.62 (30.56 to 30.68)
	IMD=9	3639	97568.68	40.09 (40.03 to 40.15)	6905	119970	60.72 (60.65 to 60.79)	815	46619.69	16.51 (16.47 to 16.56)	1830	52620	31.72 (31.66 to 31.78)
	Most Deprivation	1010	25821.75	42.69 (42.63 to 42.75)	1965	31807	65.13 (65.05 to 65.20)	206	12346.83	15.21 (15.17 to 15.26)	441	13916	28.61 (28.55 to 28.67)
2012	Least Deprivation	638	22957.86	26.91 (26.87 to 26.96)	1018	26468	36.70 (36.65 to 36.76)	201	14713.03	11.29 (11.25 to 11.32)	365	16094	18.00 (17.95 to 18.05)
	IMD=2	1125	36877.42	30.13 (30.08 to 30.18)	1883	43121	42.42 (42.36 to 42.48)	286	22460.21	10.71 (10.67 to 10.75)	676	24667	22.17 (22.12 to 22.22)
	IMD=3	1409	46767.22	30.11 (30.06 to 30.16)	2496	55195	44.53 (44.47 to 44.59)	395	27923.35	12.15 (12.11 to 12.18)	971	30879	26.00 (25.94 to 26.06)
	IMD=4	1613	48147.79	33.49 (33.44 to 33.54)	2848	57420	48.85 (48.79 to 48.92)	479	28284.19	14.42 (14.38 to 14.46)	965	31306	25.08 (25.02 to 25.13)
	IMD=5	1953	57169.14	34.75 (34.70 to 34.81)	3423	68563	49.96 (49.90 to 50.03)	527	31915.83	14.14 (14.10 to 14.18)	1166	35521	27.04 (26.98 to 27.10)
	IMD=6	2369	67694.18	36.19 (36.13 to 36.24)	4163	81398	52.29 (52.22 to 52.36)	583	35929.38	14.47 (14.42 to 14.51)	1255	40027	27.29 (27.23 to 27.35)
	IMD=7	2623	76993.53	35.40 (35.34 to 35.45)	4605	92553	51.25 (51.18 to 51.31)	674	39129.35	15.77 (15.72 to 15.81)	1366	43540	27.85 (27.80 to 27.91)
	IMD=8	3073	89949.23	36.55 (36.50 to 36.61)	5626	108628	54.60 (54.54 to 54.67)	789	43047.03	16.99 (16.94 to 17.03)	1642	48368	30.42 (30.36 to 30.48)
	IMD=9	3741	99666.45	39.72 (39.66 to 39.78)	7333	122632	62.52 (62.45 to 62.59)	879	48068.92	16.91 (16.87 to 16.96)	1913	54104	32.00 (31.94 to 32.06)
	Most Deprivation	987	26462.73	39.06 (39.01 to 39.12)	1954	32544	62.01 (61.94 to 62.08)	226	12788.64	15.73 (15.69 to 15.77)	450	14373	28.08 (28.02 to 28.14)
2013	Least Deprivation	647	23151.26	27.13 (27.08 to 27.17)	1005	26805	36.18 (36.12 to 36.23)	218	14852.39	12.06 (12.03 to 12.10)	395	16252	19.20 (19.15 to 19.25)
	IMD=2	1095	37127.01	29.28 (29.23 to 29.33)	1911	44067	42.41 (42.35 to 42.47)	314	22703.34	11.70 (11.66 to 11.74)	706	25072	22.92 (22.87 to 22.98)
	IMD=3	1430	47411.12	29.86 (29.81 to 29.91)	2514	56446	43.64 (43.58 to 43.70)	439	28294.33	13.25 (13.21 to 13.29)	965	31449	25.36 (25.30 to 25.42)
	IMD=4	1651	49157.96	33.27 (33.22 to 33.32)	2940	59218	48.91 (48.84 to 48.97)	488	28761.02	14.38 (14.33 to 14.42)	986	32009	25.19 (25.14 to 25.25)
	IMD=5	2038	57606.64	35.79 (35.73 to 35.84)	3628	70792	51.56 (51.50 to 51.63)	617	32286.17	16.28 (16.24 to 16.33)	1225	36324	27.80 (27.74 to 27.86)
	IMD=6	2310	66648.56	35.42 (35.36 to 35.47)	4203	83230	51.53 (51.46 to 51.59)	616	36257.54	15.10 (15.05 to 15.14)	1276	41006	27.03 (26.98 to 27.09)
	IMD=7	2742	75744.34	37.16 (37.10 to 37.21)	4946	94957	53.45 (53.38 to 53.51)	712	39598.01	16.24 (16.19 to 16.28)	1450	44936	28.70 (28.64 to 28.75)
	IMD=8	3383	88999.86	39.87 (39.82 to 39.93)	6168	112860	57.28 (57.22 to 57.35)	895	43628.41	18.83 (18.78 to 18.87)	1761	50264	31.44 (31.37 to 31.50)
	IMD=9	3971	96009.97	43.36 (43.30 to 43.42)	7727	125421	64.30 (64.23 to 64.37)	1010	47867.05	19.13 (19.08 to 19.18)	2041	55793	33.10 (33.03 to 33.16)
	Most Deprivation	1091	25166.85	45.67 (45.61 to 45.73)	2176	33221	67.57 (67.49 to 67.64)	261	12764.88	18.64 (18.59 to 18.69)	509	14928	30.75 (30.69 to 30.81)
2014	Least Deprivation	613	23450.4	25.12 (25.08 to 25.17)	968	27094	34.05 (33.99 to 34.10)	221	15085.68	12.13 (12.09 to 12.17)	412	16489	19.88 (19.83 to 19.93)
	IMD=2	1094	37113.42	29.23 (29.19 to 29.28)	1868	44047	41.36 (41.30 to 41.42)	345	22786.97	12.65 (12.61 to 12.69)	712	25163	22.77 (22.72 to 22.82)
	IMD=3	1503	47731.06	31.35 (31.30 to 31.40)	2595	56976	44.54 (44.48 to 44.60)	448	28527.01	13.37 (13.33 to 13.41)	950	31795	24.61 (24.55 to 24.66)

	IMD=4	1670	49793.61	33.99 (33.94 to 34.04)	2990	60111	49.22 (49.15 to 49.28)	539	29073.45	15.60 (15.56 to 15.64)	1053	32430	26.30 (26.25 to 26.36)
	IMD=5	2059	58453.99	35.74 (35.69 to 35.80)	3692	71156	51.88 (51.81 to 51.95)	589	32606.39	15.39 (15.35 to 15.44)	1229	36681	27.61 (27.56 to 27.67)
	IMD=6	2468	67770.22	37.42 (37.36 to 37.47)	4392	82862	53.61 (53.55 to 53.68)	705	36779.77	17.35 (17.30 to 17.39)	1377	41289	28.94 (28.88 to 29.00)
	IMD=7	2872	77994.45	38.26 (38.20 to 38.31)	5133	95318	55.22 (55.15 to 55.29)	754	40673.65	16.78 (16.74 to 16.83)	1513	45602	29.32 (29.26 to 29.38)
	IMD=8	3417	91289.46	39.90 (39.84 to 39.96)	6332	112455	58.85 (58.78 to 58.92)	865	44779.79	17.92 (17.87 to 17.96)	1774	50783	31.34 (31.28 to 31.40)
	IMD=9	4038	97878.77	43.32 (43.26 to 43.38)	7820	122448	65.74 (65.67 to 65.82)	1086	48805.88	20.48 (20.43 to 20.53)	2208	55550	35.62 (35.55 to 35.68)
	Most Deprivation	1115	25311.12	46.49 (46.43 to 46.56)	2150	31993	68.50 (68.43 to 68.58)	291	12944.07	21.17 (21.12 to 21.22)	554	14726	33.58 (33.52 to 33.65)
2015	Least Deprivation	655	23785	26.66 (26.61 to 26.71)	999	27380	34.76 (34.71 to 34.82)	243	15295.12	13.16 (13.12 to 13.20)	408	16702	19.36 (19.31 to 19.41)
	IMD=2	1134	37715.78	29.76 (29.71 to 29.81)	1843	44297	40.41 (40.35 to 40.46)	384	23020.3	13.99 (13.95 to 14.03)	744	25441	23.40 (23.35 to 23.45)
	IMD=3	1411	48578.32	28.98 (28.93 to 29.03)	2365	57287	40.56 (40.51 to 40.62)	487	28940.26	14.42 (14.37 to 14.46)	929	32124	23.88 (23.83 to 23.94)
	IMD=4	1623	51354.31	32.03 (31.98 to 32.08)	2763	61131	44.78 (44.72 to 44.84)	517	29820.65	14.84 (14.80 to 14.89)	975	33107	24.05 (23.99 to 24.10)
	IMD=5	1964	60515.93	33.17 (33.12 to 33.22)	3513	72851	48.48 (48.41 to 48.54)	594	33318.69	15.28 (15.24 to 15.32)	1186	37309	26.28 (26.23 to 26.34)
	IMD=6	2357	69957.4	34.68 (34.63 to 34.74)	4173	84863	49.92 (49.85 to 49.98)	705	37781.38	16.81 (16.77 to 16.86)	1338	42285	27.47 (27.41 to 27.52)
	IMD=7	2788	81593.2	36.01 (35.95 to 36.06)	5031	98914	52.39 (52.32 to 52.46)	812	42134.35	17.62 (17.57 to 17.66)	1506	47075	28.33 (28.27 to 28.39)
	IMD=8	3495	95887.25	38.60 (38.54 to 38.66)	6484	117239	57.82 (57.75 to 57.89)	901	46747.9	17.84 (17.79 to 17.89)	1882	52606	32.06 (32.00 to 32.12)
	IMD=9	4141	102575	42.63 (42.57 to 42.69)	8089	127699	65.34 (65.26 to 65.41)	1045	51029.62	19.24 (19.19 to 19.29)	2265	57815	35.63 (35.57 to 35.70)
	Most Deprivation	1079	26758.82	42.83 (42.77 to 42.89)	2229	33475	68.40 (68.33 to 68.48)	352	13606.74	24.28 (24.23 to 24.33)	635	15384	36.98 (36.91 to 37.04)
2016	Least Deprivation	650	24372.48	25.81 (25.76 to 25.85)	980	27898	33.51 (33.46 to 33.56)	263	15582.61	13.95 (13.90 to 13.99)	411	16949	19.23 (19.18 to 19.27)
	IMD=2	1067	38897.07	27.06 (27.01 to 27.10)	1735	45291	37.25 (37.20 to 37.31)	351	23583.8	12.50 (12.46 to 12.53)	688	25934	21.32 (21.27 to 21.37)
	IMD=3	1351	50397.2	26.73 (26.68 to 26.77)	2205	58640	36.86 (36.80 to 36.91)	484	29821.3	13.79 (13.75 to 13.83)	928	32822	23.28 (23.23 to 23.33)
	IMD=4	1592	53690.8	30.04 (29.99 to 30.09)	2680	63362	42.19 (42.13 to 42.25)	555	30908.75	15.36 (15.32 to 15.40)	976	34173	23.43 (23.38 to 23.48)
	IMD=5	1892	63496.51	30.79 (30.74 to 30.84)	3293	75684	44.08 (44.02 to 44.14)	650	34637.2	16.18 (16.14 to 16.22)	1220	38579	26.23 (26.17 to 26.29)
	IMD=6	2231	73169.84	31.66 (31.61 to 31.71)	3992	87401	46.53 (46.47 to 46.59)	795	39053.02	18.28 (18.23 to 18.33)	1448	43526	28.94 (28.89 to 29.00)
	IMD=7	2760	85828.73	33.79 (33.74 to 33.85)	4931	103081	49.52 (49.46 to 49.59)	836	43985.4	17.59 (17.54 to 17.64)	1569	48996	28.55 (28.49 to 28.61)
	IMD=8	3344	101721	35.24 (35.18 to 35.29)	6289	123024	53.61 (53.54 to 53.68)	1012	49278.1	19.57 (19.52 to 19.61)	1921	55163	31.66 (31.60 to 31.72)
	IMD=9	4142	108580.2	40.63 (40.57 to 40.69)	8139	133863	62.88 (62.81 to 62.95)	1151	53551.9	19.78 (19.73 to 19.83)	2404	60548	35.77 (35.70 to 35.83)
	Most Deprivation	1101	28620.79	41.23 (41.18 to 41.29)	2214	35311	64.69 (64.62 to 64.76)	322	14360.21	21.16 (21.11 to 21.21)	654	16227	36.17 (36.10 to 36.23)
2017	Least Deprivation	625	24893.62	24.48 (24.43 to 24.52)	914	28463	30.65 (30.60 to 30.70)	260	15849.65	13.75 (13.71 to 13.80)	414	17319	19.09 (19.04 to 19.13)
	IMD=2	1033	39948.65	25.49 (25.45 to 25.54)	1666	46318	34.95 (34.89 to 35.00)	399	24053.88	14.03 (13.98 to 14.07)	745	26463	22.76 (22.70 to 22.81)
	IMD=3	1238	51817.38	23.92 (23.87 to 23.96)	2045	60066	33.39 (33.34 to 33.44)	524	30446.54	14.57 (14.52 to 14.61)	960	33591	23.54 (23.48 to 23.59)

	IMD=4	1506	55536.59	27.51 (27.46 to 27.55)	2533	65102	38.74 (38.69 to 38.80)	585	31625.48	15.80 (15.76 to 15.84)	1033	35050	24.20 (24.14 to 24.25)
	IMD=5	1852	66000.59	28.85 (28.80 to 28.89)	3154	77909	40.82 (40.77 to 40.88)	637	35716.46	15.48 (15.44 to 15.52)	1233	39753	25.88 (25.82 to 25.93)
	IMD=6	2149	76610.64	29.04 (28.99 to 29.09)	3760	90762	42.28 (42.22 to 42.34)	788	40475.5	17.90 (17.85 to 17.94)	1484	45128	28.93 (28.87 to 28.99)
	IMD=7	2760	90193.5	32.54 (32.49 to 32.59)	4873	107598	47.09 (47.02 to 47.15)	891	45793.81	18.23 (18.18 to 18.28)	1637	51150	28.71 (28.65 to 28.76)
	IMD=8	3414	107501.5	34.72 (34.66 to 34.77)	6298	129247	51.66 (51.59 to 51.72)	1034	51600.86	18.59 (18.54 to 18.64)	2039	57911	31.67 (31.61 to 31.74)
	IMD=9	4157	115131.7	38.69 (38.63 to 38.74)	8103	141099	59.81 (59.74 to 59.88)	1137	56070.62	18.68 (18.63 to 18.73)	2423	63587	34.25 (34.19 to 34.31)
	Most Deprivation	1112	30276.94	39.35 (39.30 to 39.41)	2218	37112	61.87 (61.79 to 61.94)	345	14956.83	21.67 (21.62 to 21.72)	699	16944	37.51 (37.44 to 37.58)
2018	Least Deprivation	642	25680.22	24.20 (24.15 to 24.24)	924	29302	30.15 (30.10 to 30.20)	284	16279.76	14.52 (14.47 to 14.56)	460	17800	20.56 (20.51 to 20.61)
	IMD=2	1068	42178.32	25.24 (25.20 to 25.29)	1627	48404	32.87 (32.82 to 32.93)	427	25082.71	14.27 (14.23 to 14.32)	724	27530	21.27 (21.22 to 21.32)
	IMD=3	1310	54363.18	24.08 (24.04 to 24.13)	2041	62288	32.19 (32.13 to 32.24)	489	31523.95	13.20 (13.16 to 13.24)	859	34736	20.28 (20.23 to 20.33)
	IMD=4	1592	58492.63	27.64 (27.59 to 27.69)	2566	67897	37.73 (37.67 to 37.79)	590	32857.85	15.23 (15.19 to 15.28)	1033	36259	23.25 (23.19 to 23.30)
	IMD=5	1895	70571.94	27.39 (27.34 to 27.44)	3279	82472	39.94 (39.88 to 39.99)	745	37858.57	17.13 (17.08 to 17.17)	1285	42024	25.65 (25.60 to 25.71)
	IMD=6	2324	81463.11	29.74 (29.69 to 29.79)	3824	95693	40.85 (40.79 to 40.91)	840	42409.84	18.09 (18.04 to 18.13)	1515	47336	28.09 (28.04 to 28.15)
	IMD=7	2663	96414.22	29.17 (29.12 to 29.22)	4713	114107	42.81 (42.75 to 42.87)	1036	48371.34	20.00 (19.95 to 20.05)	1778	54098	29.20 (29.14 to 29.26)
	IMD=8	3408	114848.2	32.35 (32.30 to 32.41)	6253	137257	48.44 (48.38 to 48.51)	1093	54508.37	18.78 (18.73 to 18.82)	2086	61348	30.61 (30.55 to 30.67)
	IMD=9	4060	122677.5	35.69 (35.64 to 35.75)	7833	149195	55.03 (54.97 to 55.10)	1214	59209.05	19.43 (19.38 to 19.48)	2489	67133	33.68 (33.62 to 33.74)
	Most Deprivation	1147	32656.03	37.40 (37.35 to 37.46)	2246	39680	58.54 (58.47 to 58.61)	323	15839.58	18.63 (18.59 to 18.68)	695	18018	34.73 (34.66 to 34.79)
2019	Least Deprivation	595	27126.69	21.00 (20.96 to 21.04)	891	30728	27.51 (27.46 to 27.56)	297	17163.55	14.21 (14.17 to 14.25)	495	18778	20.81 (20.76 to 20.86)
	IMD=2	1046	44440.1	23.21 (23.17 to 23.25)	1576	50855	30.08 (30.03 to 30.13)	431	26246.91	13.65 (13.61 to 13.70)	727	28884	20.19 (20.14 to 20.24)
	IMD=3	1312	57399.04	22.74 (22.70 to 22.78)	1994	65444	29.85 (29.80 to 29.90)	537	33180.1	13.58 (13.54 to 13.62)	898	36546	19.99 (19.94 to 20.04)
	IMD=4	1588	61197.6	26.40 (26.35 to 26.45)	2519	70883	35.54 (35.49 to 35.60)	687	33956.64	17.19 (17.15 to 17.24)	1131	37624	24.55 (24.50 to 24.61)
	IMD=5	1922	75149.53	26.26 (26.21 to 26.31)	3161	87571	36.54 (36.49 to 36.60)	773	39850.96	16.84 (16.80 to 16.89)	1349	44412	25.46 (25.40 to 25.52)
	IMD=6	2271	86538.7	27.36 (27.31 to 27.41)	3779	100946	38.34 (38.29 to 38.40)	901	44582.99	18.54 (18.50 to 18.59)	1549	49793	27.39 (27.34 to 27.45)
	IMD=7	2705	100179.8	28.59 (28.54 to 28.64)	4667	119808	40.67 (40.62 to 40.73)	1011	49749.16	18.94 (18.89 to 18.99)	1748	56529	27.74 (27.68 to 27.79)
	IMD=8	3352	117317.4	31.09 (31.04 to 31.14)	5968	145068	43.94 (43.88 to 44.01)	1121	54952.49	19.25 (19.20 to 19.30)	2062	64325	28.93 (28.87 to 28.99)
	IMD=9	4160	127415.6	35.08 (35.03 to 35.14)	7602	156381	50.76 (50.69 to 50.82)	1276	60769.86	20.09 (20.04 to 20.14)	2458	69972	31.84 (31.77 to 31.90)
	Most Deprivation	1143	33161.1	37.07 (37.01 to 37.12)	2138	41803	53.18 (53.11 to 53.24)	350	15924.55	20.54 (20.49 to 20.59)	703	18843	33.42 (33.36 to 33.49)
**South East Coast**													
2004	Least Deprivation	1552	54840.98	27.18 (27.13 to 27.22)	2401	62947	36.33 (36.28 to 36.39)	545	34302.28	13.31 (13.27 to 13.35)	998	37265	21.72 (21.67 to 21.77)
	IMD=2	1599	47585.45	32.17 (32.12 to 32.22)	2350	54585	40.83 (40.77 to 40.89)	539	30236.55	13.99 (13.95 to 14.03)	972	33251	22.15 (22.09 to 22.20)

	IMD=3	1569	47298.21	32.08 (32.03 to 32.13)	2341	54251	41.15 (41.09 to 41.21)	564	30247.98	14.23 (14.19 to 14.27)	960	33258	21.28 (21.23 to 21.34)
	IMD=4	1029	29873.34	33.87 (33.82 to 33.92)	1509	34564	42.17 (42.11 to 42.23)	370	18634.16	15.31 (15.27 to 15.35)	604	20612	21.94 (21.89 to 21.99)
	IMD=5	1075	30795.75	34.34 (34.29 to 34.40)	1691	36062	45.62 (45.56 to 45.68)	378	19101.23	15.39 (15.34 to 15.43)	695	21213	24.65 (24.60 to 24.71)
	IMD=6	953	27023.19	35.33 (35.27 to 35.38)	1564	31837	48.22 (48.16 to 48.29)	388	16758.17	17.23 (17.19 to 17.28)	668	18889	25.27 (25.21 to 25.32)
	IMD=7	800	23086.92	34.66 (34.60 to 34.71)	1261	26968	46.13 (46.06 to 46.19)	255	13554.02	14.80 (14.76 to 14.84)	421	14880	21.69 (21.64 to 21.74)
	IMD=8	624	17126.69	36.57 (36.52 to 36.63)	1036	20200	51.23 (51.16 to 51.29)	222	9936.353	16.40 (16.35 to 16.44)	433	11157	27.17 (27.11 to 27.23)
	IMD=9	447	11493.5	39.32 (39.26 to 39.38)	702	13488	51.88 (51.82 to 51.95)	117	6302.667	14.80 (14.76 to 14.84)	202	6956	22.76 (22.71 to 22.81)
	Most Deprivation	136	4822.366	28.76 (28.71 to 28.81)	209	5561	37.49 (37.43 to 37.54)	42	2666.993	12.31 (12.27 to 12.35)	72	2965	18.00 (17.96 to 18.05)
2005	Least Deprivation	1639	54215.77	29.28 (29.23 to 29.33)	2627	62922	40.05 (39.99 to 40.11)	510	34613.23	12.36 (12.32 to 12.40)	1023	37843	22.07 (22.02 to 22.12)
	IMD=2	1569	47043.09	32.18 (32.13 to 32.23)	2446	54571	42.65 (42.59 to 42.71)	573	30235.7	15.15 (15.11 to 15.19)	1054	33400	24.14 (24.08 to 24.19)
	IMD=3	1656	47562.43	33.56 (33.50 to 33.61)	2533	55125	43.86 (43.80 to 43.92)	615	30710	15.49 (15.45 to 15.53)	1063	33956	23.18 (23.13 to 23.24)
	IMD=4	1019	29656.71	33.26 (33.20 to 33.31)	1599	34536	44.58 (44.52 to 44.65)	373	18781.98	15.50 (15.45 to 15.54)	638	20771	23.17 (23.12 to 23.22)
	IMD=5	1120	30427.54	35.84 (35.79 to 35.90)	1818	35784	49.27 (49.21 to 49.34)	364	19038.23	15.04 (15.00 to 15.09)	713	21100	25.56 (25.51 to 25.62)
	IMD=6	1059	26712.15	39.52 (39.46 to 39.58)	1696	31665	52.24 (52.18 to 52.31)	371	16637.69	16.97 (16.92 to 17.01)	690	18769	26.63 (26.57 to 26.68)
	IMD=7	842	23180.17	36.17 (36.11 to 36.22)	1413	27470	50.78 (50.71 to 50.84)	275	13708.09	16.01 (15.97 to 16.06)	505	15208	25.56 (25.51 to 25.62)
	IMD=8	641	17219.44	36.98 (36.92 to 37.03)	1089	20527	52.42 (52.35 to 52.49)	228	10002.94	16.78 (16.73 to 16.82)	480	11353	29.79 (29.73 to 29.85)
	IMD=9	444	11542.65	39.00 (38.94 to 39.06)	739	13783	54.11 (54.04 to 54.18)	119	6397.215	14.61 (14.57 to 14.65)	224	7123	24.17 (24.11 to 24.22)
	Most Deprivation	132	4842.634	27.67 (27.62 to 27.72)	230	5677	41.05 (40.99 to 41.10)	53	2645.81	15.19 (15.14 to 15.23)	92	2998	22.66 (22.61 to 22.71)
2006	Least Deprivation	1694	54422.17	30.30 (30.25 to 30.35)	2794	63204	42.43 (42.37 to 42.49)	459	35018.06	11.04 (11.00 to 11.08)	956	38316	20.41 (20.36 to 20.46)
	IMD=2	1640	47137.46	33.46 (33.40 to 33.51)	2595	55224	44.78 (44.72 to 44.84)	537	30693.2	13.88 (13.84 to 13.92)	1078	34004	24.20 (24.14 to 24.25)
	IMD=3	1605	48005.45	32.87 (32.82 to 32.93)	2650	56138	45.53 (45.47 to 45.59)	542	31311.34	13.47 (13.43 to 13.51)	1044	34763	22.41 (22.36 to 22.47)
	IMD=4	1060	29896.87	34.43 (34.38 to 34.49)	1749	35220	47.73 (47.66 to 47.79)	372	19059.25	15.34 (15.29 to 15.38)	678	21214	24.23 (24.18 to 24.28)
	IMD=5	1100	30901.32	34.88 (34.82 to 34.93)	1861	36585	49.26 (49.19 to 49.32)	367	19468.45	14.86 (14.82 to 14.90)	754	21702	26.45 (26.40 to 26.51)
	IMD=6	949	26802.73	35.03 (34.98 to 35.09)	1639	32091	49.79 (49.72 to 49.85)	330	16801.88	14.94 (14.90 to 14.99)	698	19034	26.68 (26.63 to 26.74)
	IMD=7	829	23601.05	34.78 (34.73 to 34.84)	1447	28024	50.62 (50.56 to 50.69)	263	13974.68	15.10 (15.06 to 15.15)	591	15598	29.38 (29.32 to 29.44)
	IMD=8	650	17425.24	36.97 (36.92 to 37.03)	1125	20893	52.88 (52.82 to 52.95)	221	10183.06	16.47 (16.43 to 16.52)	511	11571	31.69 (31.63 to 31.76)
	IMD=9	461	11780.08	39.54 (39.49 to 39.60)	815	14201	57.54 (57.47 to 57.61)	122	6579.726	15.14 (15.10 to 15.19)	243	7343	26.20 (26.14 to 26.25)
	Most Deprivation	155	4955.203	32.02 (31.96 to 32.07)	263	5839	45.73 (45.67 to 45.79)	39	2689.079	10.91 (10.87 to 10.95)	99	3024	24.47 (24.42 to 24.52)
2007	Least Deprivation	1609	54235.16	28.85 (28.80 to 28.90)	2743	64214	41.01 (40.95 to 41.07)	495	35501.27	11.66 (11.62 to 11.70)	996	38901	20.96 (20.91 to 21.01)
	IMD=2	1575	47162.67	32.17 (32.12 to 32.22)	2687	55668	45.63 (45.57 to 45.69)	512	31091.92	13.15 (13.11 to 13.19)	1079	34494	23.96 (23.91 to 24.01)

	IMD=3	1678	47748.46	34.39 (34.33 to 34.44)	2766	56510	47.08 (47.02 to 47.14)	521	31648.42	12.78 (12.75 to 12.82)	1041	35234	22.06 (22.00 to 22.11)
	IMD=4	1145	29826.38	37.56 (37.51 to 37.62)	1811	35636	49.27 (49.21 to 49.34)	345	19198.29	14.24 (14.20 to 14.28)	643	21476	22.79 (22.73 to 22.84)
	IMD=5	1137	30874.67	36.42 (36.36 to 36.47)	1957	37031	51.18 (51.12 to 51.25)	367	19653.87	14.76 (14.72 to 14.80)	774	22078	26.73 (26.67 to 26.79)
	IMD=6	978	26667.96	36.16 (36.11 to 36.22)	1731	32134	52.47 (52.40 to 52.53)	356	16885.11	16.09 (16.05 to 16.14)	707	19132	26.85 (26.79 to 26.91)
	IMD=7	878	23687.04	36.74 (36.68 to 36.79)	1546	28384	53.47 (53.40 to 53.54)	253	14192.46	14.48 (14.44 to 14.52)	605	15913	29.71 (29.65 to 29.77)
	IMD=8	717	17451.64	41.31 (41.26 to 41.37)	1274	21155	60.25 (60.18 to 60.32)	252	10284.61	19.01 (18.96 to 19.06)	560	11796	34.83 (34.76 to 34.89)
	IMD=9	492	11785.87	41.96 (41.90 to 42.02)	841	14469	58.18 (58.11 to 58.25)	115	6652.164	14.03 (13.99 to 14.07)	257	7484	26.93 (26.87 to 26.99)
	Most Deprivation	157	4983.926	32.19 (32.14 to 32.24)	267	5946	45.00 (44.94 to 45.06)	31	2749.629	9.07 (9.03 to 9.10)	75	3089	18.65 (18.60 to 18.70)
2008	Least Deprivation	1652	54796.86	29.40 (29.35 to 29.45)	2854	64438	42.54 (42.48 to 42.60)	510	36367.13	11.68 (11.64 to 11.72)	1011	39557	20.74 (20.69 to 20.79)
	IMD=2	1621	47630.06	33.04 (32.99 to 33.09)	2722	56176	46.29 (46.22 to 46.35)	491	31654.68	12.31 (12.28 to 12.35)	1051	35005	22.87 (22.82 to 22.93)
	IMD=3	1668	48242.23	33.26 (33.21 to 33.31)	2793	57061	46.40 (46.34 to 46.46)	612	32253.56	14.73 (14.69 to 14.78)	1161	35756	24.29 (24.24 to 24.35)
	IMD=4	1069	30224.51	34.33 (34.28 to 34.39)	1829	36083	48.74 (48.68 to 48.81)	354	19677.62	14.17 (14.13 to 14.21)	684	21919	23.65 (23.60 to 23.71)
	IMD=5	1179	31212.13	37.10 (37.04 to 37.16)	2070	37410	53.78 (53.71 to 53.85)	382	20132.24	15.08 (15.03 to 15.12)	815	22468	27.68 (27.62 to 27.74)
	IMD=6	963	26969.68	35.82 (35.77 to 35.88)	1735	32357	52.55 (52.49 to 52.62)	326	17198.86	14.60 (14.56 to 14.64)	694	19278	26.30 (26.24 to 26.35)
	IMD=7	892	24209.07	37.05 (36.99 to 37.10)	1631	28998	55.78 (55.71 to 55.84)	282	14569.67	15.62 (15.57 to 15.66)	669	16309	32.29 (32.23 to 32.35)
	IMD=8	701	17702.32	39.72 (39.66 to 39.78)	1308	21461	60.42 (60.35 to 60.49)	226	10500.61	16.88 (16.83 to 16.92)	550	11989	33.92 (33.86 to 33.99)
	IMD=9	520	12007.11	43.76 (43.70 to 43.82)	981	14642	67.47 (67.39 to 67.54)	138	6813.646	16.25 (16.21 to 16.30)	285	7647	29.33 (29.27 to 29.39)
	Most Deprivation	209	5124.06	42.15 (42.09 to 42.21)	347	6110	57.82 (57.75 to 57.89)	57	2827.231	15.74 (15.70 to 15.79)	105	3164	25.43 (25.37 to 25.48)
2009	Least Deprivation	1663	55193.01	29.25 (29.20 to 29.30)	2932	64958	43.06 (43.00 to 43.12)	545	37039.48	12.22 (12.18 to 12.26)	1055	40424	21.13 (21.08 to 21.18)
	IMD=2	1726	48174.53	34.85 (34.80 to 34.90)	2940	56964	49.32 (49.26 to 49.39)	528	32354.58	13.02 (12.98 to 13.06)	1088	35645	23.25 (23.19 to 23.30)
	IMD=3	1770	48780.74	35.34 (35.29 to 35.40)	2993	57849	49.67 (49.61 to 49.74)	590	32795.47	14.09 (14.04 to 14.13)	1144	36343	23.78 (23.72 to 23.83)
	IMD=4	1141	30690.58	36.57 (36.51 to 36.62)	1960	36739	51.75 (51.68 to 51.81)	381	20155.42	14.89 (14.85 to 14.93)	706	22384	23.96 (23.91 to 24.01)
	IMD=5	1265	31610.12	39.28 (39.22 to 39.33)	2233	38049	56.81 (56.74 to 56.88)	395	20591.78	15.39 (15.35 to 15.44)	860	23005	28.78 (28.72 to 28.84)
	IMD=6	1026	27492.48	36.72 (36.66 to 36.77)	1754	32971	51.80 (51.74 to 51.87)	366	17530.61	16.16 (16.11 to 16.20)	736	19709	27.71 (27.65 to 27.77)
	IMD=7	991	24555.48	40.56 (40.50 to 40.62)	1793	29640	59.92 (59.85 to 60.00)	290	14907.82	15.97 (15.93 to 16.02)	700	16716	33.17 (33.11 to 33.24)
	IMD=8	712	17801.98	40.57 (40.51 to 40.63)	1363	21768	62.65 (62.57 to 62.72)	230	10659.91	16.80 (16.75 to 16.84)	593	12254	35.75 (35.69 to 35.82)
	IMD=9	536	11978.49	45.34 (45.28 to 45.40)	1001	14819	68.02 (67.95 to 68.10)	145	6885.188	17.06 (17.02 to 17.11)	320	7756	32.51 (32.45 to 32.57)
	Most Deprivation	178	5239.296	34.35 (34.30 to 34.40)	344	6330	55.45 (55.38 to 55.51)	33	2900.972	9.44 (9.40 to 9.47)	84	3249	20.48 (20.43 to 20.53)
2010	Least Deprivation	1674	56186.05	28.52 (28.47 to 28.57)	2953	66032	42.07 (42.01 to 42.13)	550	37847.79	12.25 (12.21 to 12.28)	1077	41359	21.24 (21.19 to 21.29)
	IMD=2	1666	49028.49	33.25 (33.20 to 33.31)	2899	58330	47.67 (47.61 to 47.73)	532	33105.71	12.77 (12.73 to 12.81)	1138	36595	23.75 (23.70 to 23.80)

	IMD=3	1824	50012.76	35.16 (35.11 to 35.22)	3150	59578	50.31 (50.25 to 50.38)	613	33644.83	14.25 (14.21 to 14.29)	1217	37356	24.55 (24.50 to 24.61)
	IMD=4	1135	31477.63	34.87 (34.82 to 34.93)	2021	37744	51.09 (51.02 to 51.15)	354	20772.75	13.51 (13.47 to 13.55)	699	23087	23.17 (23.12 to 23.23)
	IMD=5	1239	32458.09	37.60 (37.54 to 37.65)	2258	39200	55.84 (55.77 to 55.90)	399	21222.63	15.11 (15.07 to 15.15)	897	23728	29.08 (29.02 to 29.14)
	IMD=6	1081	28022.84	37.96 (37.90 to 38.02)	1922	33751	55.18 (55.11 to 55.25)	337	17926.94	14.65 (14.60 to 14.69)	725	20154	26.67 (26.61 to 26.72)
	IMD=7	935	25020.55	37.38 (37.32 to 37.43)	1792	30356	58.20 (58.13 to 58.27)	271	15313.63	14.35 (14.31 to 14.39)	727	17235	33.25 (33.19 to 33.32)
	IMD=8	720	18097.96	40.05 (39.99 to 40.11)	1429	22278	63.90 (63.83 to 63.97)	212	10908.19	15.32 (15.27 to 15.36)	571	12503	34.22 (34.16 to 34.28)
	IMD=9	487	12103.98	40.86 (40.80 to 40.91)	1016	15062	67.49 (67.42 to 67.57)	132	7025.845	15.61 (15.56 to 15.65)	338	7954	34.26 (34.20 to 34.33)
	Most Deprivation	171	5404.413	31.56 (31.51 to 31.62)	345	6577	52.77 (52.70 to 52.84)	34	2982.338	9.40 (9.37 to 9.43)	98	3333	23.61 (23.56 to 23.67)
2011	Least Deprivation	1883	56549.4	32.12 (32.07 to 32.17)	3215	66739	45.55 (45.49 to 45.61)	614	38395.32	13.28 (13.24 to 13.32)	1152	42045	22.03 (21.97 to 22.08)
	IMD=2	1803	49558.8	35.13 (35.07 to 35.18)	3060	59076	49.05 (48.98 to 49.11)	569	33741.16	13.33 (13.29 to 13.37)	1187	37311	24.10 (24.04 to 24.15)
	IMD=3	1847	50440.23	35.72 (35.67 to 35.78)	3268	60527	51.71 (51.64 to 51.77)	642	34351.17	14.48 (14.43 to 14.52)	1294	38255	25.10 (25.04 to 25.15)
	IMD=4	1233	32109.68	37.23 (37.17 to 37.29)	2127	38648	52.81 (52.74 to 52.88)	409	21301.57	15.25 (15.20 to 15.29)	773	23696	24.87 (24.81 to 24.92)
	IMD=5	1294	33142.98	38.62 (38.57 to 38.68)	2346	40195	56.71 (56.65 to 56.78)	377	21910.37	13.78 (13.74 to 13.83)	870	24440	27.44 (27.38 to 27.50)
	IMD=6	1079	28511.11	37.51 (37.45 to 37.56)	2015	34418	57.12 (57.05 to 57.18)	359	18256.76	15.43 (15.38 to 15.47)	731	20521	26.49 (26.43 to 26.55)
	IMD=7	990	25567.51	38.71 (38.66 to 38.77)	1889	31147	59.65 (59.58 to 59.72)	301	15619.9	15.95 (15.91 to 15.99)	782	17696	35.12 (35.05 to 35.18)
	IMD=8	766	18395.79	41.58 (41.52 to 41.64)	1501	22751	65.29 (65.22 to 65.36)	252	11115.07	17.72 (17.67 to 17.76)	639	12806	37.45 (37.38 to 37.52)
	IMD=9	536	12244.17	44.08 (44.02 to 44.14)	1088	15342	70.96 (70.88 to 71.04)	141	7128.69	16.31 (16.26 to 16.35)	367	8132	36.04 (35.97 to 36.10)
	Most Deprivation	181	5496.682	33.73 (33.68 to 33.78)	339	6644	51.56 (51.49 to 51.62)	43	3057.098	11.55 (11.52 to 11.59)	103	3374	24.39 (24.34 to 24.45)
2012	Least Deprivation	1836	56496.17	31.42 (31.37 to 31.47)	3201	66666	45.23 (45.16 to 45.29)	563	39038.4	11.79 (11.75 to 11.83)	1114	42624	20.76 (20.71 to 20.81)
	IMD=2	1690	49938.02	32.90 (32.85 to 32.95)	2998	59465	47.79 (47.72 to 47.85)	583	34323.11	13.40 (13.36 to 13.44)	1224	37880	24.34 (24.28 to 24.39)
	IMD=3	1734	50689.2	33.22 (33.17 to 33.27)	3173	60762	49.69 (49.62 to 49.75)	649	34853.9	14.38 (14.34 to 14.42)	1344	38693	25.64 (25.58 to 25.69)
	IMD=4	1168	32486.68	34.93 (34.88 to 34.99)	2100	39058	51.61 (51.54 to 51.67)	408	21720.45	14.87 (14.83 to 14.91)	796	24092	25.12 (25.06 to 25.17)
	IMD=5	1285	33774.76	37.49 (37.43 to 37.54)	2390	40934	56.21 (56.14 to 56.28)	410	22516.42	14.50 (14.46 to 14.54)	911	25062	27.80 (27.74 to 27.85)
	IMD=6	1149	28682.02	39.74 (39.68 to 39.80)	2127	34762	59.48 (59.41 to 59.55)	369	18591.41	15.65 (15.60 to 15.69)	775	20869	27.83 (27.78 to 27.89)
	IMD=7	1064	26000.04	41.13 (41.07 to 41.19)	2091	31629	65.27 (65.20 to 65.35)	325	15985.93	16.64 (16.59 to 16.68)	844	18032	36.90 (36.83 to 36.97)
	IMD=8	815	18636.61	43.71 (43.65 to 43.77)	1635	22943	70.52 (70.45 to 70.60)	245	11282.71	17.48 (17.44 to 17.53)	672	12926	39.85 (39.79 to 39.92)
	IMD=9	562	12584.08	45.51 (45.44 to 45.57)	1135	15699	72.51 (72.43 to 72.59)	115	7347.075	12.94 (12.90 to 12.98)	332	8277	31.93 (31.87 to 31.99)
	Most Deprivation	204	5829.865	35.44 (35.39 to 35.50)	384	7047	54.56 (54.49 to 54.62)	45	3195.195	11.54 (11.51 to 11.58)	109	3545	24.87 (24.82 to 24.93)
2013	Least Deprivation	1706	56925.65	28.72 (28.67 to 28.76)	3009	67317	41.99 (41.93 to 42.05)	577	39598.19	11.94 (11.90 to 11.98)	1107	43340	20.15 (20.10 to 20.20)
	IMD=2	1761	50722.67	33.81 (33.75 to 33.86)	3094	60443	48.63 (48.57 to 48.69)	548	35019.47	12.27 (12.23 to 12.30)	1193	38726	23.05 (23.00 to 23.11)

	IMD=3	1832	51302.63	34.72 (34.66 to 34.77)	3278	61603	50.92 (50.85 to 50.98)	679	35392.51	14.78 (14.74 to 14.83)	1409	39472	26.08 (26.03 to 26.14)
	IMD=4	1248	33076.75	36.52 (36.46 to 36.57)	2217	39919	53.00 (52.94 to 53.07)	393	22130.1	13.99 (13.95 to 14.03)	762	24652	23.45 (23.39 to 23.50)
	IMD=5	1323	34375.67	37.62 (37.56 to 37.68)	2477	41931	56.60 (56.53 to 56.67)	393	23085.28	13.58 (13.53 to 13.62)	916	25817	27.05 (26.99 to 27.10)
	IMD=6	1150	28969.97	39.29 (39.24 to 39.35)	2161	35347	59.51 (59.44 to 59.58)	349	18897.89	14.55 (14.51 to 14.59)	735	21258	26.07 (26.02 to 26.13)
	IMD=7	1029	26261.34	39.40 (39.34 to 39.46)	2054	32223	62.88 (62.80 to 62.95)	288	16251.67	14.62 (14.58 to 14.67)	797	18389	34.30 (34.24 to 34.36)
	IMD=8	746	18871.56	40.12 (40.06 to 40.18)	1615	23440	68.82 (68.74 to 68.90)	267	11455.84	18.53 (18.49 to 18.58)	694	13153	39.97 (39.90 to 40.04)
	IMD=9	617	12892.87	48.80 (48.74 to 48.87)	1244	16219	77.20 (77.12 to 77.28)	126	7568.988	13.89 (13.85 to 13.93)	347	8537	32.64 (32.57 to 32.70)
	Most Deprivation	212	6013.421	35.89 (35.84 to 35.95)	436	7341	60.23 (60.16 to 60.30)	51	3242.798	12.99 (12.95 to 13.03)	113	3627	25.35 (25.30 to 25.41)
2014	Least Deprivation	1697	57812.92	28.27 (28.22 to 28.32)	2889	68016	40.11 (40.05 to 40.17)	673	40149.12	13.61 (13.57 to 13.65)	1129	43950	20.20 (20.15 to 20.25)
	IMD=2	1631	51552.81	30.59 (30.54 to 30.64)	2838	61220	43.94 (43.88 to 44.01)	608	35440.16	13.40 (13.36 to 13.44)	1175	39203	22.45 (22.40 to 22.50)
	IMD=3	1793	52270.62	33.13 (33.07 to 33.18)	3188	62365	48.55 (48.48 to 48.61)	672	35963.38	14.44 (14.39 to 14.48)	1374	40061	25.13 (25.07 to 25.18)
	IMD=4	1172	33739.75	33.99 (33.94 to 34.05)	2176	40529	51.75 (51.68 to 51.82)	385	22626.72	13.37 (13.33 to 13.41)	780	25123	23.51 (23.46 to 23.57)
	IMD=5	1285	35263.12	35.86 (35.81 to 35.92)	2461	42639	55.64 (55.57 to 55.71)	463	23657.74	15.56 (15.52 to 15.60)	995	26373	28.72 (28.66 to 28.78)
	IMD=6	1127	29310.19	38.24 (38.18 to 38.29)	2187	35720	59.89 (59.82 to 59.96)	371	19219.84	15.22 (15.18 to 15.26)	776	21544	27.28 (27.22 to 27.33)
	IMD=7	1024	26625.64	38.49 (38.44 to 38.55)	2072	32553	62.64 (62.57 to 62.71)	297	16600	14.78 (14.73 to 14.82)	837	18718	35.46 (35.39 to 35.53)
	IMD=8	773	19179.87	40.93 (40.87 to 40.99)	1630	23838	68.34 (68.27 to 68.42)	239	11685.08	16.35 (16.30 to 16.39)	653	13410	36.91 (36.84 to 36.98)
	IMD=9	551	13067.99	42.80 (42.74 to 42.85)	1161	16453	70.76 (70.68 to 70.84)	124	7787.548	13.27 (13.23 to 13.31)	351	8748	32.43 (32.36 to 32.49)
	Most Deprivation	202	6162.626	33.44 (33.39 to 33.49)	411	7478	56.07 (56.00 to 56.13)	55	3318.957	13.96 (13.92 to 14.00)	122	3698	27.11 (27.05 to 27.17)
2015	Least Deprivation	1650	59035.26	26.92 (26.87 to 26.97)	2784	68945	37.78 (37.72 to 37.84)	651	40676.52	13.06 (13.02 to 13.10)	1102	44561	19.42 (19.37 to 19.46)
	IMD=2	1640	52330.86	30.36 (30.31 to 30.41)	2831	61938	43.25 (43.19 to 43.31)	610	35882.05	13.24 (13.20 to 13.28)	1161	39697	21.81 (21.76 to 21.86)
	IMD=3	1735	53188.97	31.67 (31.62 to 31.72)	3099	63310	46.79 (46.73 to 46.85)	698	36425.11	14.86 (14.82 to 14.90)	1423	40678	25.82 (25.76 to 25.87)
	IMD=4	1168	34348.97	33.33 (33.28 to 33.39)	2083	41054	48.72 (48.66 to 48.79)	458	23022.34	15.65 (15.61 to 15.70)	818	25559	24.35 (24.30 to 24.41)
	IMD=5	1251	35753.39	34.45 (34.40 to 34.51)	2364	43239	52.71 (52.64 to 52.77)	448	23841.76	14.93 (14.89 to 14.97)	990	26681	28.26 (28.20 to 28.31)
	IMD=6	1132	29636.28	38.18 (38.12 to 38.24)	2163	36133	58.53 (58.46 to 58.60)	410	19361.31	16.59 (16.55 to 16.64)	815	21760	28.23 (28.17 to 28.29)
	IMD=7	1018	26930.71	38.07 (38.01 to 38.12)	2084	33085	62.23 (62.16 to 62.31)	345	16811.77	16.77 (16.72 to 16.81)	856	19057	35.55 (35.49 to 35.62)
	IMD=8	754	19421.47	39.43 (39.37 to 39.48)	1562	24105	64.80 (64.72 to 64.87)	257	11884.37	17.04 (16.99 to 17.08)	633	13626	35.13 (35.06 to 35.19)
	IMD=9	541	13216.8	41.72 (41.66 to 41.78)	1167	16632	70.33 (70.26 to 70.41)	149	7897.328	15.51 (15.47 to 15.55)	376	8863	34.00 (33.93 to 34.06)
	Most Deprivation	213	6157.125	35.15 (35.09 to 35.20)	441	7475	59.79 (59.71 to 59.86)	52	3391.055	12.46 (12.42 to 12.50)	115	3776	24.71 (24.66 to 24.77)
2016	Least Deprivation	1668	60646.42	26.14 (26.09 to 26.18)	2704	70430	35.83 (35.78 to 35.89)	638	41264.86	12.57 (12.53 to 12.61)	1085	45238	18.79 (18.74 to 18.83)
	IMD=2	1562	53442.44	28.19 (28.15 to 28.24)	2680	62842	40.11 (40.05 to 40.16)	654	36378.61	13.97 (13.93 to 14.01)	1177	40161	21.88 (21.82 to 21.93)

	IMD=3	1601	54034.14	28.37 (28.33 to 28.42)	2866	64087	42.28 (42.22 to 42.34)	721	36915.35	15.01 (14.97 to 15.05)	1365	41155	24.49 (24.44 to 24.55)
	IMD=4	1157	35147.88	32.11 (32.06 to 32.16)	2107	41886	48.07 (48.00 to 48.13)	473	23477.11	15.76 (15.71 to 15.80)	843	26068	24.47 (24.42 to 24.53)
	IMD=5	1196	36923.05	31.87 (31.82 to 31.93)	2261	44144	49.35 (49.28 to 49.41)	453	24546.5	14.66 (14.62 to 14.71)	903	27323	25.19 (25.13 to 25.24)
	IMD=6	1083	30256.89	35.52 (35.47 to 35.58)	2081	36686	55.18 (55.11 to 55.24)	417	19696.38	16.70 (16.66 to 16.75)	825	22096	28.35 (28.29 to 28.40)
	IMD=7	908	27733.73	32.91 (32.85 to 32.96)	1906	33681	55.84 (55.77 to 55.91)	319	17267.86	15.24 (15.20 to 15.28)	782	19424	32.01 (31.95 to 32.07)
	IMD=8	751	20087.73	37.73 (37.67 to 37.78)	1556	24655	62.60 (62.53 to 62.68)	251	12223.6	16.55 (16.50 to 16.59)	614	13937	33.84 (33.78 to 33.91)
	IMD=9	514	13583.13	38.35 (38.29 to 38.41)	1112	16944	65.83 (65.75 to 65.90)	136	8092.583	13.75 (13.71 to 13.80)	356	9082	31.30 (31.24 to 31.36)
	Most Deprivation	204	6330.99	32.51 (32.46 to 32.57)	410	7676	53.80 (53.73 to 53.87)	37	3496.542	8.98 (8.94 to 9.01)	109	3900	22.83 (22.78 to 22.89)
2017	Least Deprivation	1559	62195.75	24.05 (24.00 to 24.09)	2451	72118	32.11 (32.06 to 32.16)	632	41944.83	12.19 (12.15 to 12.23)	1043	45898	17.76 (17.71 to 17.80)
	IMD=2	1486	54530.65	25.91 (25.86 to 25.95)	2365	63640	34.98 (34.92 to 35.03)	703	36800.21	14.79 (14.74 to 14.83)	1114	40673	20.52 (20.47 to 20.56)
	IMD=3	1646	55335.27	28.67 (28.62 to 28.72)	2641	65042	38.48 (38.43 to 38.54)	768	37556.25	15.72 (15.67 to 15.76)	1270	41951	22.41 (22.36 to 22.46)
	IMD=4	1036	36115.38	28.02 (27.97 to 28.07)	1804	42858	40.49 (40.43 to 40.54)	448	23952.16	14.62 (14.58 to 14.67)	739	26618	21.14 (21.09 to 21.19)
	IMD=5	1175	37970.81	30.34 (30.29 to 30.39)	2068	45296	44.14 (44.07 to 44.20)	498	24996.05	15.77 (15.73 to 15.82)	884	27967	23.99 (23.94 to 24.05)
	IMD=6	1042	30741.45	33.53 (33.48 to 33.58)	1838	37199	48.28 (48.21 to 48.34)	405	19938.03	16.02 (15.98 to 16.07)	746	22443	25.41 (25.36 to 25.47)
	IMD=7	889	28333.97	31.67 (31.62 to 31.72)	1656	34276	47.83 (47.76 to 47.89)	320	17494.02	14.83 (14.79 to 14.87)	698	19724	27.98 (27.92 to 28.04)
	IMD=8	731	20670.46	35.59 (35.54 to 35.65)	1320	25285	51.88 (51.82 to 51.95)	282	12593.35	18.20 (18.16 to 18.25)	537	14308	29.01 (28.95 to 29.07)
	IMD=9	492	13925.01	36.05 (36.00 to 36.11)	932	17183	54.42 (54.35 to 54.49)	185	8169.185	18.40 (18.36 to 18.45)	353	9201	30.38 (30.32 to 30.44)
	Most Deprivation	186	6379.436	29.75 (29.70 to 29.80)	332	7700	43.83 (43.77 to 43.89)	49	3529.574	11.51 (11.47 to 11.55)	86	3930	17.81 (17.76 to 17.86)
2018	Least Deprivation	1611	64251.45	24.14 (24.10 to 24.19)	2387	73640	30.62 (30.57 to 30.67)	755	42825.51	14.24 (14.20 to 14.28)	1161	46797	19.33 (19.28 to 19.37)
	IMD=2	1406	56160.54	24.17 (24.13 to 24.22)	2215	65070	32.34 (32.29 to 32.39)	771	37491.05	15.91 (15.87 to 15.95)	1161	41539	20.93 (20.88 to 20.98)
	IMD=3	1589	56779.22	26.66 (26.61 to 26.71)	2529	66256	35.97 (35.91 to 36.02)	824	38182.03	16.48 (16.44 to 16.53)	1363	42736	23.54 (23.49 to 23.59)
	IMD=4	1011	37179.1	26.40 (26.35 to 26.45)	1700	43661	37.32 (37.26 to 37.37)	520	24269.9	16.56 (16.52 to 16.61)	818	27044	22.78 (22.73 to 22.83)
	IMD=5	1159	38843.52	29.23 (29.18 to 29.28)	1958	45878	41.28 (41.23 to 41.34)	540	25353.67	16.75 (16.70 to 16.79)	877	28315	23.60 (23.55 to 23.66)
	IMD=6	1055	31355.95	33.23 (33.18 to 33.28)	1840	37722	47.52 (47.45 to 47.58)	417	20121.77	16.28 (16.24 to 16.33)	770	22703	25.84 (25.78 to 25.90)
	IMD=7	899	29278.31	30.72 (30.67 to 30.77)	1574	35004	44.41 (44.35 to 44.47)	366	17885.4	16.72 (16.68 to 16.77)	683	20094	26.81 (26.75 to 26.86)
	IMD=8	719	21369.37	33.94 (33.89 to 34.00)	1261	25886	48.59 (48.53 to 48.65)	289	12789.28	18.17 (18.12 to 18.21)	533	14556	28.40 (28.34 to 28.45)
	IMD=9	507	14194.5	36.54 (36.49 to 36.60)	937	17367	54.21 (54.15 to 54.28)	153	8237.996	15.17 (15.12 to 15.21)	311	9339	26.19 (26.13 to 26.24)
	Most Deprivation	196	6352.238	31.61 (31.56 to 31.67)	322	7602	42.83 (42.77 to 42.89)	69	3537.823	16.57 (16.52 to 16.61)	94	3930	19.68 (19.63 to 19.73)
2019	Least Deprivation	1637	65775.55	23.96 (23.92 to 24.01)	2422	75423	30.45 (30.40 to 30.50)	867	43261.29	16.04 (15.99 to 16.08)	1352	47543	22.05 (21.99 to 22.10)
	IMD=2	1481	55967.1	25.15 (25.11 to 25.20)	2241	65766	32.20 (32.14 to 32.25)	807	37041.42	16.76 (16.71 to 16.80)	1270	42135	22.29 (22.23 to 22.34)

	IMD=3	1538	56724.9	25.74 (25.69 to 25.78)	2459	67077	34.38 (34.33 to 34.43)	862	37668.24	17.49 (17.44 to 17.53)	1399	43230	23.83 (23.78 to 23.88)
	IMD=4	1017	36359.9	27.17 (27.12 to 27.22)	1584	44376	34.27 (34.22 to 34.33)	585	23410.75	19.31 (19.26 to 19.36)	920	27494	25.08 (25.03 to 25.14)
	IMD=5	1140	38610.02	28.91 (28.86 to 28.96)	1864	47002	38.28 (38.22 to 38.33)	597	24752.71	18.94 (18.89 to 18.98)	966	28957	25.17 (25.11 to 25.22)
	IMD=6	1035	30855.14	33.24 (33.19 to 33.29)	1746	38210	44.67 (44.61 to 44.73)	484	19521.98	19.36 (19.31 to 19.41)	862	22992	28.44 (28.38 to 28.50)
	IMD=7	923	29525.63	31.22 (31.17 to 31.27)	1544	35787	42.51 (42.45 to 42.57)	393	17736.62	17.76 (17.72 to 17.81)	713	20513	27.14 (27.08 to 27.20)
	IMD=8	736	21880	33.98 (33.93 to 34.03)	1289	26410	48.51 (48.45 to 48.57)	324	12955.62	19.96 (19.91 to 20.01)	589	14818	30.65 (30.59 to 30.71)
	IMD=9	482	14495.76	33.74 (33.69 to 33.79)	841	17540	47.95 (47.89 to 48.02)	205	8313.278	19.93 (19.88 to 19.98)	377	9447	31.17 (31.11 to 31.23)
	Most Deprivation	197	6316.17	31.20 (31.15 to 31.25)	314	7523	41.35 (41.29 to 41.41)	59	3497.454	13.83 (13.79 to 13.87)	86	3908	17.75 (17.71 to 17.80)

Supplementary Table S7. Slope index of inequality (SII) and relative index of inequality (RII) for standardised incidence and prevalence of low back pain and osteoarthritis by geographical region between 2004-2019

SII indicates slope index of inequality; RII indicates relative index of inequality; CI indicates confidence interval.

Year	Linear model							
	Incidence				Prevalence			
	LBP		OA		LBP		OA	
	SII (95%CI)	RII (95% CI)	SII (95%CI)	RII (95% CI)	SII (95%CI)	RII (95% CI)	SII (95%CI)	RII (95% CI)
	**North East**							
2004	8.48 (8.43-8.53)	1.247 (1.246-1.249)	0.85 (0.80-0.90)	1.039 (1.036-1.041)	13.82 (13.76-13.88)	1.324 (1.323-1.325)	4.31 (4.25-4.38)	1.132 (1.130-1.134)
2005	5.96 (5.91-6.01)	1.180 (1.178-1.181)	5.18 (5.13-5.23)	1.246 (1.243-1.248)	10.51 (10.46-10.57)	1.245 (1.244-1.247)	7.50 (7.43-7.56)	1.225 (1.223-1.227)
2006	12.55 (12.49-12.60)	1.360 (1.359-1.362)	3.51 (3.46-3.56)	1.185 (1.182-1.187)	16.27 (16.21-16.33)	1.361 (1.360-1.363)	6.19 (6.12-6.25)	1.199 (1.197-1.201)
2007	7.13 (7.08-7.19)	1.201 (1.199-1.202)	3.97 (3.92-4.02)	1.198 (1.195-1.200)	13.20 (13.13-13.26)	1.283 (1.282-1.284)	8.14 (8.08-8.20)	1.251 (1.249-1.253)
2008	11.45 (11.39-11.50)	1.327 (1.325-1.328)	5.40 (5.35-5.45)	1.254 (1.251-1.256)	18.67 (18.61-18.73)	1.404 (1.403-1.405)	9.12 (9.05-9.18)	1.270 (1.268-1.272)
2009	15.56 (15.51-15.62)	1.416 (1.415-1.418)	6.14 (6.09-6.19)	1.295 (1.292-1.297)	22.80 (22.74-22.86)	1.463 (1.462-1.465)	10.30 (10.23-10.36)	1.301 (1.300-1.303)
2010	15.51 (15.45-15.56)	1.416 (1.414-1.417)	6.89 (6.84-6.94)	1.352 (1.349-1.354)	25.45 (25.39-25.51)	1.517 (1.516-1.519)	10.94 (10.88-11.00)	1.337 (1.335-1.339)
2011	17.53 (17.47-17.59)	1.465 (1.463-1.466)	4.28 (4.23-4.32)	1.229 (1.227-1.232)	26.42 (26.36-26.49)	1.527 (1.526-1.529)	10.15 (10.08-10.21)	1.324 (1.322-1.326)
2012	18.20 (18.14-18.25)	1.495 (1.494-1.497)	5.08 (5.03-5.13)	1.256 (1.253-1.258)	28.39 (28.33-28.46)	1.570 (1.569-1.572)	10.36 (10.29-10.42)	1.325 (1.323-1.327)
2013	18.50 (18.45-18.56)	1.503 (1.502-1.505)	4.41 (4.36-4.46)	1.219 (1.216-1.221)	27.91 (27.85-27.98)	1.566 (1.564-1.567)	9.51 (9.45-9.57)	1.301 (1.299-1.303)
2014	16.89 (16.83-16.95)	1.431 (1.429-1.432)	3.68 (3.63-3.73)	1.172 (1.170-1.174)	29.08 (29.02-29.15)	1.559 (1.558-1.561)	8.97 (8.91-9.03)	1.271 (1.270-1.273)
2015	15.79 (15.73-15.85)	1.416 (1.414-1.417)	2.38 (2.33-2.43)	1.104 (1.102-1.106)	29.36 (29.30-29.43)	1.586 (1.585-1.587)	8.65 (8.58-8.71)	1.251 (1.249-1.253)
2016	19.65 (19.59-19.70)	1.528 (1.526-1.529)	4.69 (4.63-4.74)	1.210 (1.208-1.212)	30.11 (30.05-30.18)	1.608 (1.607-1.609)	12.44 (12.37-12.5)	1.361 (1.359-1.363)
2017	22.04 (21.98-22.09)	1.614 (1.613-1.616)	3.54 (3.48-3.59)	1.142 (1.140-1.145)	34.27 (34.20-34.33)	1.711 (1.710-1.712)	10.73 (10.67-10.8)	1.288 (1.286-1.290)
2018	18.66 (18.60-18.71)	1.536 (1.534-1.537)	1.92 (1.86-1.98)	1.076 (1.074-1.078)	28.68 (28.62-28.75)	1.612 (1.611-1.614)	6.85 (6.78-6.92)	1.177 (1.175-1.179)
2019	17.13 (17.07-17.18)	1.484 (1.482-1.485)	4.58 (4.52-4.63)	1.183 (1.18-1.185)	28.13 (28.07-28.19)	1.616 (1.615-1.618)	11.16 (11.09-11.23)	1.299 (1.298-1.301)
	**North West**							
2004	12.23 (12.18-12.29)	1.343 (1.341-1.344)	5.07 (5.03-5.12)	1.279 (1.276-1.281)	19.84 (19.78-19.91)	1.416 (1.414-1.417)	8.39 (8.34-8.45)	1.295 (1.293-1.297)
2005	14.50 (14.45-14.56)	1.395 (1.394-1.397)	5.60 (5.56-5.65)	1.311 (1.309-1.314)	22.21 (22.15-22.28)	1.449 (1.447-1.450)	10.06 (10.00-10.11)	1.341 (1.339-1.343)
2006	15.05 (15.00-15.11)	1.410 (1.409-1.412)	4.77 (4.72-4.81)	1.275 (1.272-1.278)	23.33 (23.26-23.40)	1.458 (1.457-1.459)	10.04 (9.98-10.10)	1.343 (1.341-1.345)
2007	15.97 (15.91-16.03)	1.421 (1.419-1.422)	5.87 (5.83-5.92)	1.345 (1.342-1.347)	26.18 (26.12-26.25)	1.494 (1.493-1.495)	10.98 (10.92-11.04)	1.369 (1.367-1.371)
2008	16.33 (16.28-16.39)	1.439 (1.437-1.440)	5.26 (5.22-5.31)	1.311 (1.308-1.313)	27.78 (27.72-27.85)	1.521 (1.520-1.522)	13.69 (13.63-13.75)	1.457 (1.455-1.459)
2009	18.05 (17.99-18.10)	1.477 (1.475-1.478)	5.07 (5.02-5.11)	1.300 (1.298-1.303)	30.46 (30.39-30.52)	1.558 (1.557-1.560)	13.58 (13.53-13.64)	1.456 (1.454-1.458)
2010	18.41 (18.35-18.47)	1.476 (1.474-1.477)	4.80 (4.76-4.85)	1.277 (1.275-1.28)	32.27 (32.21-32.34)	1.579 (1.577-1.580)	14.95 (14.89-15.01)	1.491 (1.489-1.493)
2011	16.14 (16.08-16.20)	1.415 (1.413-1.416)	5.77 (5.72-5.81)	1.351 (1.348-1.354)	31.30 (31.23-31.37)	1.554 (1.552-1.555)	16.09 (16.03-16.15)	1.544 (1.542-1.546)
2012	19.44 (19.38-19.50)	1.500 (1.499-1.501)	4.63 (4.59-4.68)	1.288 (1.286-1.291)	35.89 (35.82-35.95)	1.631 (1.630-1.632)	15.50 (15.45-15.56)	1.540 (1.538-1.542)
2013	19.46 (19.41-19.52)	1.491 (1.489-1.492)	5.02 (4.97-5.06)	1.289 (1.286-1.292)	38.82 (38.76-38.89)	1.681 (1.680-1.682)	15.35 (15.30-15.41)	1.523 (1.521-1.525)
2014	19.19 (19.14-19.25)	1.490 (1.488-1.491)	6.15 (6.11-6.20)	1.333 (1.330-1.336)	36.81 (36.74-36.88)	1.655 (1.654-1.656)	15.66 (15.60-15.72)	1.523 (1.521-1.525)
2015	20.57 (20.52-20.63)	1.539 (1.538-1.541)	4.10 (4.06-4.15)	1.215 (1.212-1.217)	38.47 (38.40-38.54)	1.704 (1.702-1.705)	14.53 (14.47-14.60)	1.469 (1.467-1.471)
2016	16.78 (16.73-16.84)	1.446 (1.445-1.448)	3.07 (3.02-3.12)	1.151 (1.149-1.154)	34.14 (34.07-34.21)	1.638 (1.636-1.639)	12.16 (12.10-12.22)	1.379 (1.378-1.381)
2017	16.26 (16.21-16.32)	1.438 (1.436-1.439)	2.96 (2.91-3.01)	1.142 (1.140-1.145)	33.24 (33.18-33.31)	1.636 (1.635-1.638)	10.8 (10.74-10.86)	1.328 (1.326-1.330)
2018	17.02 (16.97-17.08)	1.483 (1.481-1.484)	3.61 (3.56-3.65)	1.175 (1.173-1.178)	32.53 (32.47-32.60)	1.656 (1.654-1.657)	11.69 (11.62-11.75)	1.358 (1.356-1.360)
2019	16.08 (16.02-16.13)	1.465 (1.464-1.467)	2.60 (2.55-2.65)	1.122 (1.119-1.124)	30.12 (30.06-30.19)	1.632 (1.630-1.633)	11.64 (11.57-11.70)	1.346 (1.344-1.348)
	**Yorkshire and the Humber**							

2004	10.84 (10.78-10.89)	1.302 (1.301-1.304)	2.97 (2.92-3.02)	1.143 (1.141-1.145)	13.00 (12.94-13.06)	1.279 (1.278-1.280)	1.63 (1.56-1.69)	1.047 (1.045-1.049)
2005	8.71 (8.65-8.76)	1.234 (1.233-1.236)	-0.40 (-0.45--0.35)	0.980 (0.978-0.983)	13.90 (13.83-13.96)	1.283 (1.281-1.284)	1.09 (1.02-1.16)	1.030 (1.029-1.032)
2006	6.90 (6.84-6.95)	1.185 (1.183-1.186)	1.08 (1.03-1.12)	1.058 (1.055-1.060)	12.64 (12.57-12.70)	1.253 (1.252-1.255)	-0.41 (-0.47--0.35)	0.988 (0.986-0.990)
2007	12.24 (12.19-12.30)	1.322 (1.320-1.323)	0.05 (0.00-0.09)	1.002 (1.000-1.005)	18.57 (18.50-18.63)	1.367 (1.366-1.368)	0.47 (0.41-0.53)	1.014 (1.012-1.016)
2008	9.77 (9.71-9.82)	1.265 (1.263-1.266)	0.97 (0.92-1.01)	1.049 (1.046-1.051)	18.38 (18.31-18.44)	1.365 (1.363-1.366)	1.65 (1.58-1.71)	1.047 (1.045-1.049)
2009	12.45 (12.39-12.50)	1.343 (1.342-1.345)	2.47 (2.42-2.52)	1.113 (1.111-1.115)	20.45 (20.38-20.51)	1.406 (1.405-1.407)	3.31 (3.24-3.38)	1.088 (1.086-1.090)
2010	8.57 (8.52-8.63)	1.239 (1.238-1.241)	1.50 (1.45-1.55)	1.069 (1.067-1.071)	17.07 (17.01-17.14)	1.341 (1.340-1.342)	1.78 (1.71-1.85)	1.047 (1.045-1.049)
2011	15.49 (15.44-15.55)	1.400 (1.399-1.402)	3.93 (3.88-3.98)	1.189 (1.186-1.191)	22.41 (22.35-22.48)	1.422 (1.420-1.423)	6.09 (6.03-6.16)	1.165 (1.164-1.167)
2012	15.01 (14.95-15.06)	1.386 (1.384-1.387)	1.07 (1.02-1.12)	1.049 (1.047-1.052)	27.02 (26.95-27.09)	1.499 (1.498-1.501)	6.25 (6.19-6.32)	1.166 (1.164-1.168)
2013	15.77 (15.72-15.83)	1.427 (1.425-1.428)	0.25 (0.20-0.30)	1.012 (1.010-1.015)	27.93 (27.86-27.99)	1.535 (1.534-1.536)	4.57 (4.51-4.64)	1.127 (1.125-1.129)
2014	12.85 (12.8-12.91)	1.342 (1.341-1.344)	1.27 (1.22-1.32)	1.064 (1.062-1.067)	24.89 (24.83-24.96)	1.470 (1.469-1.472)	6.25 (6.18-6.31)	1.175 (1.174-1.177)
2015	14.25 (14.19-14.30)	1.392 (1.390-1.393)	3.87 (3.81-3.92)	1.182 (1.179-1.184)	25.67 (25.60-25.73)	1.501 (1.500-1.503)	5.99 (5.92-6.05)	1.167 (1.165-1.169)
2016	13.97 (13.91-14.02)	1.394 (1.392-1.395)	-0.65 (-0.70--0.60)	0.969 (0.966-0.971)	26.00 (25.94-26.07)	1.519 (1.517-1.520)	3.17 (3.11-3.24)	1.089 (1.087-1.091)
2017	12.78 (12.73-12.84)	1.377 (1.375-1.378)	-1.14 (-1.19--1.09)	0.945 (0.942-0.947)	25.83 (25.77-25.90)	1.538 (1.536-1.539)	2.89 (2.82-2.95)	1.079 (1.078-1.081)
2018	13.07 (13.02-13.12)	1.410 (1.409-1.412)	0.63 (0.57-0.68)	1.029 (1.027-1.032)	23.36 (23.30-23.42)	1.509 (1.508-1.511)	2.75 (2.68-2.81)	1.076 (1.074-1.078)
2019	15.03 (14.98-15.08)	1.459 (1.457-1.460)	-3.40 (-3.46--3.35)	0.851 (0.849-0.854)	24.03 (23.97-24.09)	1.572 (1.571-1.574)	-2.50 (-2.56--2.44)	0.927 (0.925-0.929)
	**East Midlands**							
2004	-7.74 (-7.80--7.69)	0.775 (0.773-0.777)	-2.68 (-2.73--2.64)	0.838 (0.835-0.840)	-5.72 (-5.78--5.66)	0.871 (0.869-0.872)	0.88 (0.82-0.94)	1.030 (1.028-1.032)
2005	5.23 (5.17-5.28)	1.135 (1.133-1.136)	-0.42 (-0.47--0.38)	0.976 (0.973-0.978)	2.68 (2.61-2.74)	1.052 (1.051-1.053)	-2.23 (-2.29--2.16)	0.932 (0.930-0.934)
2006	4.72 (4.66-4.77)	1.124 (1.122-1.125)	0.10 (0.05-0.14)	1.006 (1.004-1.009)	8.22 (8.16-8.29)	1.156 (1.154-1.157)	0.50 (0.44-0.56)	1.016 (1.014-1.018)
2007	5.13 (5.07-5.18)	1.139 (1.138-1.141)	-0.01 (-0.06-0.03)	0.999 (0.997-1.002)	8.08 (8.02-8.15)	1.156 (1.155-1.157)	2.62 (2.55-2.68)	1.081 (1.079-1.083)
2008	6.24 (6.18-6.30)	1.167 (1.165-1.168)	2.22 (2.18-2.27)	1.131 (1.128-1.134)	12.16 (12.10-12.23)	1.229 (1.228-1.231)	5.43 (5.37-5.49)	1.165 (1.163-1.167)
2009	-1.62 (-1.68--1.57)	0.954 (0.953-0.956)	1.20 (1.16-1.25)	1.067 (1.064-1.069)	7.69 (7.62-7.75)	1.147 (1.145-1.148)	4.15 (4.09-4.21)	1.123 (1.121-1.125)
2010	2.95 (2.90-3.01)	1.079 (1.078-1.081)	-2.06 (-2.10--2.01)	0.878 (0.875-0.881)	10.48 (10.42-10.55)	1.195 (1.194-1.197)	5.16 (5.10-5.22)	1.157 (1.156-1.159)
2011	11.95 (11.89-12.01)	1.300 (1.299-1.302)	0.18 (0.13-0.23)	1.010 (1.007-1.012)	21.28 (21.21-21.35)	1.372 (1.370-1.373)	8.97 (8.91-9.04)	1.257 (1.255-1.259)
2012	10.54 (10.48-10.60)	1.268 (1.267-1.270)	4.32 (4.28-4.37)	1.230 (1.227-1.232)	21.02 (20.95-21.09)	1.367 (1.366-1.368)	9.71 (9.64-9.77)	1.284 (1.282-1.286)
2013	6.08 (6.02-6.14)	1.149 (1.148-1.151)	1.24 (1.20-1.29)	1.071 (1.068-1.073)	15.89 (15.82-15.96)	1.277 (1.276-1.278)	5.92 (5.86-5.98)	1.190 (1.188-1.192)
2014	-3.21 (-3.27--3.16)	0.914 (0.913-0.916)	-1.53 (-1.58--1.49)	0.917 (0.914-0.919)	10.83 (10.76-10.90)	1.202 (1.200-1.203)	0.64 (0.58-0.70)	1.021 (1.019-1.023)
2015	2.17 (2.11-2.22)	1.062 (1.06-1.063)	-0.95 (-1.00--0.91)	0.949 (0.947-0.952)	9.84 (9.78-9.91)	1.195 (1.194-1.196)	2.02 (1.96-2.08)	1.064 (1.062-1.066)
2016	7.79 (7.74-7.84)	1.226 (1.224-1.228)	2.38 (2.33-2.43)	1.121 (1.119-1.124)	12.06 (11.99-12.12)	1.244 (1.242-1.245)	5.60 (5.53-5.66)	1.174 (1.172-1.176)
2017	4.40 (4.35-4.45)	1.130 (1.129-1.132)	1.17 (1.13-1.22)	1.060 (1.058-1.063)	13.24 (13.18-13.30)	1.278 (1.276-1.279)	2.82 (2.76-2.88)	1.090 (1.088-1.092)
2018	11.04 (10.99-11.09)	1.338 (1.337-1.340)	1.82 (1.77-1.87)	1.088 (1.086-1.090)	17.34 (17.28-17.40)	1.381 (1.380-1.382)	4.89 (4.82-4.95)	1.147 (1.145-1.149)
2019	6.50 (6.44-6.55)	1.211 (1.210-1.213)	5.69 (5.65-5.74)	1.293 (1.290-1.295)	18.69 (18.63-18.74)	1.481 (1.479-1.482)	10.32 (10.26-10.38)	1.361 (1.359-1.363)
	**West Midlands**							
2004	2.08 (2.02-2.13)	1.061 (1.059-1.063)	-1.31 (-1.36--1.27)	0.925 (0.922-0.928)	4.75 (4.69-4.81)	1.109 (1.107-1.110)	-0.30 (-0.36--0.24)	0.989 (0.987-0.991)
2005	3.91 (3.86-3.96)	1.115 (1.113-1.116)	1.62 (1.58-1.67)	1.094 (1.091-1.096)	7.68 (7.62-7.74)	1.169 (1.167-1.170)	3.13 (3.08-3.19)	1.111 (1.109-1.113)
2006	4.77 (4.72-4.82)	1.139 (1.137-1.140)	1.75 (1.70-1.79)	1.108 (1.105-1.111)	8.57 (8.50-8.63)	1.184 (1.182-1.185)	3.81 (3.75-3.87)	1.139 (1.137-1.141)
2007	6.30 (6.25-6.35)	1.183 (1.181-1.184)	3.00 (2.95-3.04)	1.185 (1.182-1.188)	11.39 (11.33-11.46)	1.240 (1.239-1.242)	5.80 (5.75-5.86)	1.212 (1.209-1.214)
2008	7.11 (7.05-7.16)	1.203 (1.202-1.205)	2.70 (2.66-2.75)	1.161 (1.158-1.163)	12.57 (12.51-12.64)	1.260 (1.259-1.261)	6.41 (6.35-6.46)	1.225 (1.223-1.227)

2009	8.51 (8.46-8.57)	1.232 (1.230-1.233)	4.00 (3.95-4.05)	1.230 (1.227-1.232)	16.28 (16.22-16.35)	1.321 (1.320-1.322)	9.45 (9.39-9.50)	1.321 (1.319-1.323)
2010	9.26 (9.20-9.31)	1.256 (1.254-1.257)	5.24 (5.20-5.29)	1.310 (1.308-1.313)	17.96 (17.89-18.02)	1.354 (1.353-1.355)	11.30 (11.24-11.36)	1.389 (1.387-1.391)
2011	10.64 (10.59-10.70)	1.287 (1.286-1.289)	4.51 (4.47-4.56)	1.265 (1.262-1.267)	20.05 (19.98-20.11)	1.386 (1.385-1.387)	11.83 (11.77-11.89)	1.405 (1.403-1.407)
2012	12.95 (12.89-13.01)	1.342 (1.341-1.344)	5.85 (5.81-5.90)	1.354 (1.352-1.357)	23.61 (23.55-23.68)	1.440 (1.439-1.441)	13.95 (13.89-14.01)	1.486 (1.484-1.488)
2013	13.47 (13.41-13.52)	1.361 (1.359-1.362)	5.81 (5.76-5.85)	1.326 (1.323-1.328)	25.27 (25.21-25.34)	1.480 (1.479-1.482)	12.01 (11.95-12.06)	1.418 (1.416-1.420)
2014	11.22 (11.17-11.28)	1.300 (1.299-1.302)	5.46 (5.41-5.51)	1.290 (1.287-1.293)	22.82 (22.75-22.88)	1.435 (1.434-1.436)	12.37 (12.31-12.43)	1.419 (1.417-1.421)
2015	10.74 (10.68-10.79)	1.293 (1.291-1.294)	4.70 (4.65-4.75)	1.241 (1.238-1.243)	22.08 (22.02-22.15)	1.429 (1.428-1.430)	11.71 (11.65-11.77)	1.383 (1.381-1.385)
2016	10.27 (10.22-10.33)	1.293 (1.291-1.294)	5.20 (5.15-5.24)	1.260 (1.257-1.262)	21.28 (21.22-21.35)	1.432 (1.430-1.433)	12.47 (12.41-12.53)	1.400 (1.398-1.402)
2017	9.42 (9.37-9.47)	1.281 (1.280-1.283)	5.74 (5.69-5.79)	1.285 (1.282-1.287)	19.35 (19.29-19.42)	1.412 (1.411-1.414)	13.41 (13.35-13.47)	1.428 (1.426-1.430)
2018	11.26 (11.21-11.32)	1.354 (1.352-1.356)	5.50 (5.45-5.55)	1.271 (1.268-1.273)	20.25 (20.19-20.31)	1.458 (1.456-1.459)	13.02 (12.96-13.08)	1.420 (1.418-1.422)
2019	9.88 (9.83-9.93)	1.322 (1.320-1.323)	3.01 (2.96-3.06)	1.151 (1.148-1.153)	18.73 (18.67-18.79)	1.440 (1.438-1.441)	9.47 (9.41-9.53)	1.310 (1.308-1.312)
	**East of England**							
2004	-2.07 (-2.12--2.01)	0.940 (0.939-0.942)	1.75 (1.70-1.80)	1.096 (1.094-1.099)	-1.4 (-1.46--1.34)	0.969 (0.967-0.970)	5.61 (5.55-5.67)	1.204 (1.202-1.206)
2005	2.57 (2.51-2.63)	1.067 (1.065-1.068)	2.40 (2.36-2.45)	1.134 (1.132-1.137)	2.64 (2.58-2.71)	1.053 (1.052-1.055)	8.23 (8.17-8.29)	1.276 (1.274-1.278)
2006	3.11 (3.06-3.17)	1.081 (1.080-1.083)	0.88 (0.83-0.92)	1.051 (1.049-1.054)	2.59 (2.52-2.65)	1.051 (1.050-1.052)	4.94 (4.88-5.00)	1.171 (1.169-1.173)
2007	4.73 (4.68-4.79)	1.125 (1.124-1.127)	1.13 (1.08-1.17)	1.066 (1.063-1.068)	5.28 (5.21-5.34)	1.102 (1.101-1.104)	4.54 (4.48-4.60)	1.160 (1.158-1.162)
2008	3.48 (3.42-3.53)	1.091 (1.089-1.092)	-1.96 (-2.00--1.91)	0.876 (0.874-0.879)	4.41 (4.35-4.48)	1.085 (1.083-1.086)	2.16 (2.10-2.21)	1.079 (1.077-1.081)
2009	2.52 (2.46-2.58)	1.066 (1.064-1.067)	-0.30 (-0.35--0.26)	0.980 (0.978-0.983)	5.28 (5.22-5.35)	1.101 (1.100-1.103)	1.55 (1.50-1.61)	1.059 (1.057-1.061)
2010	-0.29 (-0.34--0.23)	0.992 (0.991-0.994)	-1.17 (-1.22--1.13)	0.930 (0.927-0.932)	1.15 (1.09-1.22)	1.023 (1.021-1.024)	-0.21 (-0.27--0.16)	0.992 (0.99-0.994)
2011	4.80 (4.74-4.85)	1.120 (1.119-1.122)	-0.76 (-0.80--0.71)	0.951 (0.949-0.954)	6.11 (6.04-6.17)	1.112 (1.111-1.113)	0.91 (0.85-0.96)	1.035 (1.033-1.037)
2012	4.59 (4.54-4.65)	1.117 (1.116-1.119)	-0.02 (-0.06-0.02)	0.999 (0.996-1.001)	7.58 (7.51-7.64)	1.139 (1.138-1.140)	0.17 (0.12-0.23)	1.007 (1.005-1.009)
2013	6.47 (6.42-6.53)	1.164 (1.163-1.166)	0.27 (0.23-0.32)	1.016 (1.014-1.019)	7.56 (7.49-7.62)	1.140 (1.139-1.141)	0.28 (0.22-0.33)	1.011 (1.009-1.013)
2014	8.75 (8.69-8.81)	1.213 (1.212-1.215)	1.18 (1.14-1.23)	1.071 (1.069-1.074)	11.98 (11.92-12.05)	1.216 (1.215-1.217)	0.83 (0.77-0.88)	1.033 (1.031-1.035)
2015	7.43 (7.38-7.49)	1.190 (1.189-1.192)	0.65 (0.61-0.70)	1.038 (1.036-1.041)	10.85 (10.78-10.91)	1.201 (1.200-1.202)	1.87 (1.81-1.92)	1.074 (1.072-1.076)
2016	6.34 (6.29-6.39)	1.177 (1.176-1.179)	0.66 (0.62-0.71)	1.039 (1.036-1.041)	10.97 (10.91-11.04)	1.217 (1.215-1.218)	2.53 (2.47-2.59)	1.095 (1.093-1.097)
2017	10.52 (10.47-10.58)	1.300 (1.299-1.302)	0.39 (0.34-0.43)	1.023 (1.020-1.025)	15.94 (15.87-16.00)	1.325 (1.323-1.326)	1.75 (1.70-1.81)	1.067 (1.065-1.069)
2018	6.29 (6.24-6.34)	1.194 (1.192-1.195)	-1.01 (-1.06--0.97)	0.939 (0.936-0.942)	9.41 (9.35-9.47)	1.216 (1.215-1.217)	-0.46 (-0.52--0.41)	0.980 (0.978-0.983)
2019	5.69 (5.64-5.74)	1.185 (1.183-1.187)	-0.95 (-0.99--0.90)	0.945 (0.942-0.947)	5.28 (5.22-5.34)	1.136 (1.134-1.137)	-2.39 (-2.44--2.33)	0.895 (0.893-0.897)
	**South West**							
2004	5.43 (5.38-5.48)	1.160 (1.159-1.162)	-0.77 (-0.81--0.72)	0.955 (0.953-0.958)	15.33 (15.27-15.39)	1.328 (1.326-1.329)	5.30 (5.24-5.36)	1.173 (1.171-1.175)
2005	6.02 (5.96-6.07)	1.178 (1.177-1.180)	2.27 (2.22-2.31)	1.134 (1.131-1.137)	16.22 (16.16-16.28)	1.339 (1.338-1.340)	7.82 (7.76-7.88)	1.249 (1.247-1.251)
2006	3.18 (3.13-3.23)	1.095 (1.093-1.097)	-0.86 (-0.90--0.81)	0.946 (0.943-0.949)	14.78 (14.72-14.85)	1.306 (1.305-1.308)	7.64 (7.58-7.70)	1.250 (1.248-1.252)
2007	5.69 (5.63-5.74)	1.168 (1.167-1.170)	-1.78 (-1.82--1.73)	0.888 (0.886-0.891)	16.86 (16.79-16.92)	1.348 (1.346-1.349)	5.77 (5.71-5.83)	1.190 (1.188-1.192)
2008	3.07 (3.02-3.12)	1.092 (1.090-1.094)	-1.33 (-1.38--1.29)	0.920 (0.917-0.923)	15.54 (15.48-15.61)	1.316 (1.314-1.317)	5.65 (5.59-5.71)	1.181 (1.179-1.183)
2009	5.7 (5.65-5.75)	1.165 (1.164-1.167)	-1.66 (-1.70--1.61)	0.897 (0.895-0.900)	17.94 (17.88-18.01)	1.358 (1.356-1.359)	5.84 (5.78-5.90)	1.190 (1.188-1.192)
2010	3.34 (3.29-3.40)	1.101 (1.100-1.103)	-2.89 (-2.93--2.84)	0.822 (0.820-0.825)	16.30 (16.24-16.36)	1.331 (1.329-1.332)	4.44 (4.38-4.50)	1.146 (1.144-1.148)
2011	2.78 (2.73-2.84)	1.081 (1.080-1.083)	-1.04 (-1.08--0.99)	0.935 (0.932-0.938)	15.89 (15.83-15.96)	1.313 (1.312-1.315)	5.27 (5.21-5.33)	1.175 (1.173-1.177)
2012	4.06 (4.01-4.11)	1.120 (1.118-1.121)	-2.20 (-2.24--2.16)	0.857 (0.854-0.860)	17.82 (17.76-17.89)	1.353 (1.351-1.354)	4.47 (4.42-4.53)	1.154 (1.152-1.156)
2013	6.04 (5.98-6.09)	1.179 (1.177-1.180)	-1.07 (-1.12--1.03)	0.935 (0.933-0.938)	18.64 (18.57-18.70)	1.379 (1.378-1.380)	5.98 (5.92-6.04)	1.207 (1.205-1.209)

2014	7.13 (7.08-7.18)	1.210 (1.209-1.212)	-0.45 (-0.49--0.40)	0.974 (0.971-0.976)	17.49 (17.43-17.55)	1.365 (1.363-1.366)	2.85 (2.80-2.91)	1.104 (1.102-1.106)
2015	12.06 (12.00-12.11)	1.344 (1.342-1.345)	-0.53 (-0.57--0.48)	0.970 (0.968-0.973)	21.73 (21.66-21.79)	1.444 (1.442-1.445)	2.06 (2.00-2.12)	1.073 (1.071-1.075)
2016	12.06 (12.00-12.11)	1.344 (1.343-1.346)	2.06 (2.02-2.11)	1.118 (1.115-1.121)	22.61 (22.55-22.67)	1.461 (1.460-1.463)	5.65 (5.59-5.70)	1.200 (1.198-1.202)
2017	9.70 (9.65-9.75)	1.285 (1.283-1.286)	0.63 (0.58-0.67)	1.036 (1.033-1.038)	19.72 (19.66-19.78)	1.417 (1.416-1.419)	4.55 (4.49-4.60)	1.162 (1.160-1.164)
2018	8.81 (8.76-8.87)	1.265 (1.263-1.267)	-0.80 (-0.85--0.76)	0.954 (0.952-0.957)	19.39 (19.33-19.45)	1.425 (1.424-1.427)	2.63 (2.58-2.69)	1.094 (1.092-1.096)
2019	9.28 (9.23-9.33)	1.290 (1.289-1.292)	-0.98 (-1.03--0.93)	0.944 (0.942-0.947)	19.21 (19.15-19.27)	1.438 (1.437-1.440)	2.19 (2.13-2.25)	1.077 (1.075-1.079)
	**South Central**							
2004	8.13 (8.07-8.18)	1.242 (1.241-1.244)	1.76 (1.71-1.80)	1.117 (1.114-1.120)	13.46 (13.40-13.52)	1.299 (1.298-1.301)	6.54 (6.49-6.60)	1.271 (1.269-1.273)
2005	6.45 (6.39-6.50)	1.192 (1.191-1.194)	0.29 (0.25-0.33)	1.021 (1.018-1.023)	14.10 (14.03-14.16)	1.304 (1.303-1.306)	4.93 (4.88-4.99)	1.206 (1.204-1.208)
2006	7.12 (7.07-7.18)	1.213 (1.211-1.214)	0.09 (0.04-0.13)	1.006 (1.003-1.009)	14.98 (14.91-15.04)	1.318 (1.317-1.320)	4.69 (4.63-4.74)	1.201 (1.198-1.203)
2007	7.31 (7.26-7.36)	1.212 (1.211-1.214)	2.98 (2.94-3.02)	1.207 (1.204-1.210)	16.25 (16.19-16.31)	1.333 (1.332-1.334)	5.51 (5.45-5.56)	1.236 (1.234-1.238)
2008	4.65 (4.60-4.70)	1.141 (1.140-1.143)	3.02 (2.98-3.06)	1.206 (1.203-1.209)	13.84 (13.78-13.90)	1.291 (1.290-1.293)	6.16 (6.11-6.21)	1.260 (1.258-1.262)
2009	7.41 (7.36-7.47)	1.223 (1.222-1.225)	1.81 (1.77-1.85)	1.123 (1.120-1.126)	15.35 (15.29-15.42)	1.323 (1.321-1.324)	4.49 (4.44-4.54)	1.195 (1.192-1.197)
2010	5.51 (5.46-5.56)	1.168 (1.167-1.170)	3.21 (3.17-3.25)	1.215 (1.212-1.218)	14.07 (14.01-14.13)	1.299 (1.298-1.300)	6.52 (6.46-6.57)	1.277 (1.275-1.279)
2011	9.69 (9.64-9.75)	1.276 (1.274-1.277)	3.86 (3.82-3.90)	1.252 (1.250-1.255)	16.83 (16.77-16.89)	1.342 (1.341-1.343)	7.55 (7.50-7.60)	1.319 (1.317-1.322)
2012	11.43 (11.38-11.48)	1.321 (1.320-1.323)	4.36 (4.32-4.41)	1.269 (1.266-1.272)	19.82 (19.75-19.88)	1.398 (1.397-1.399)	8.24 (8.19-8.30)	1.338 (1.336-1.34)
2013	8.61 (8.56-8.66)	1.249 (1.247-1.250)	6.13 (6.09-6.18)	1.346 (1.343-1.349)	16.26 (16.20-16.33)	1.330 (1.329-1.332)	10.50 (10.44-10.55)	1.400 (1.398-1.403)
2014	9.58 (9.52-9.63)	1.272 (1.270-1.273)	6.03 (5.98-6.08)	1.337 (1.334-1.339)	17.25 (17.19-17.32)	1.349 (1.348-1.350)	9.81 (9.75-9.86)	1.373 (1.371-1.375)
2015	10.54 (10.48-10.59)	1.305 (1.303-1.306)	4.51 (4.46-4.55)	1.257 (1.255-1.260)	18.14 (18.08-18.20)	1.376 (1.375-1.377)	8.78 (8.72-8.83)	1.333 (1.331-1.335)
2016	9.97 (9.92-10.02)	1.307 (1.305-1.309)	5.12 (5.07-5.17)	1.287 (1.285-1.290)	17.79 (17.73-17.85)	1.388 (1.387-1.390)	9.67 (9.61-9.72)	1.361 (1.359-1.363)
2017	12.45 (12.4-12.5)	1.382 (1.381-1.384)	4.72 (4.68-4.77)	1.267 (1.264-1.270)	19.19 (19.13-19.25)	1.431 (1.430-1.433)	9.16 (9.10-9.22)	1.346 (1.344-1.349)
2018	12.37 (12.32-12.42)	1.389 (1.387-1.390)	4.90 (4.85-4.95)	1.277 (1.274-1.279)	20.10 (20.04-20.16)	1.464 (1.463-1.466)	8.91 (8.86-8.97)	1.343 (1.341-1.346)
2019	12.09 (12.04-12.14)	1.391 (1.389-1.392)	3.46 (3.41-3.51)	1.184 (1.182-1.187)	20.22 (20.16-20.28)	1.484 (1.482-1.485)	7.52 (7.46-7.58)	1.279 (1.277-1.281)
	**London**							
2004	4.15 (4.10-4.20)	1.141 (1.140-1.143)	2.62 (2.57-2.66)	1.161 (1.159-1.164)	8.39 (8.34-8.45)	1.223 (1.222-1.225)	5.02 (4.96-5.07)	1.202 (1.200-1.204)
2005	7.15 (7.10-7.20)	1.234 (1.232-1.235)	1.16 (1.12-1.21)	1.079 (1.076-1.081)	11.31 (11.25-11.37)	1.280 (1.278-1.281)	5.97 (5.91-6.02)	1.241 (1.239-1.243)
2006	5.83 (5.78-5.88)	1.190 (1.188-1.191)	2.27 (2.23-2.31)	1.167 (1.164-1.170)	11.64 (11.58-11.70)	1.277 (1.276-1.279)	5.28 (5.22-5.33)	1.222 (1.219-1.224)
2007	6.86 (6.81-6.91)	1.221 (1.220-1.223)	2.83 (2.79-2.87)	1.199 (1.197-1.202)	13.47 (13.41-13.53)	1.311 (1.309-1.312)	5.62 (5.56-5.67)	1.221 (1.219-1.223)
2008	7.17 (7.12-7.22)	1.228 (1.226-1.230)	1.86 (1.81-1.90)	1.130 (1.127-1.133)	15.24 (15.18-15.31)	1.340 (1.339-1.342)	5.99 (5.94-6.05)	1.235 (1.232-1.237)
2009	8.44 (8.39-8.50)	1.254 (1.252-1.255)	3.72 (3.68-3.77)	1.249 (1.246-1.251)	17.78 (17.72-17.84)	1.376 (1.375-1.377)	7.30 (7.25-7.36)	1.277 (1.274-1.279)
2010	9.19 (9.14-9.25)	1.273 (1.272-1.275)	5.36 (5.32-5.41)	1.369 (1.366-1.372)	18.13 (18.07-18.20)	1.372 (1.371-1.373)	9.07 (9.01-9.13)	1.336 (1.333-1.338)
2011	11.73 (11.67-11.78)	1.339 (1.337-1.340)	3.79 (3.75-3.83)	1.252 (1.250-1.255)	21.77 (21.70-21.83)	1.433 (1.432-1.434)	8.69 (8.63-8.75)	1.321 (1.318-1.323)
2012	11.01 (10.96-11.07)	1.320 (1.319-1.322)	6.50 (6.46-6.54)	1.454 (1.451-1.457)	22.51 (22.44-22.57)	1.443 (1.442-1.444)	10.53 (10.48-10.59)	1.396 (1.394-1.399)
2013	17.27 (17.21-17.32)	1.482 (1.480-1.483)	8.22 (8.18-8.27)	1.526 (1.524-1.529)	26.70 (26.63-26.76)	1.514 (1.513-1.515)	11.96 (11.90-12.02)	1.438 (1.436-1.440)
2014	17.09 (17.04-17.15)	1.471 (1.469-1.472)	8.83 (8.79-8.88)	1.539 (1.536-1.542)	28.87 (28.80-28.94)	1.548 (1.547-1.550)	14.33 (14.27-14.39)	1.509 (1.507-1.511)
2015	16.53 (16.48-16.59)	1.477 (1.475-1.478)	7.88 (7.84-7.93)	1.469 (1.466-1.471)	31.84 (31.77-31.90)	1.629 (1.628-1.631)	16.47 (16.41-16.53)	1.590 (1.588-1.592)
2016	16.31 (16.26-16.36)	1.503 (1.502-1.505)	8.35 (8.30-8.39)	1.494 (1.491-1.496)	31.77 (31.70-31.83)	1.671 (1.669-1.672)	17.34 (17.28-17.39)	1.626 (1.624-1.628)
2017	17.24 (17.19-17.29)	1.564 (1.563-1.566)	6.59 (6.55-6.64)	1.389 (1.386-1.392)	32.47 (32.40-32.53)	1.732 (1.731-1.733)	15.85 (15.79-15.91)	1.569 (1.567-1.571)
2018	13.47 (13.42-13.52)	1.458 (1.456-1.460)	6.69 (6.64-6.73)	1.393 (1.39-1.396)	28.04 (27.98-28.10)	1.667 (1.665-1.668)	16.00 (15.95-16.06)	1.595 (1.593-1.597)

2019	15.03 (14.99-15.08)	1.537 (1.535-1.539)	7.38 (7.34-7.43)	1.425 (1.423-1.428)	25.25 (25.20-25.31)	1.650 (1.649-1.652)	13.59 (13.53-13.64)	1.519 (1.517-1.522)
	**South East Coast**							
2004	9.54 (9.49-9.59)	1.284 (1.282-1.286)	3.01 (2.97-3.06)	1.203 (1.200-1.206)	14.41 (14.35-14.47)	1.325 (1.323-1.326)	3.08 (3.03-3.14)	1.135 (1.133-1.138)
2005	9.30 (9.24-9.35)	1.269 (1.267-1.270)	3.78 (3.74-3.82)	1.246 (1.243-1.249)	14.61 (14.55-14.67)	1.308 (1.307-1.309)	5.03 (4.97-5.08)	1.203 (1.200-1.205)
2006	6.54 (6.49-6.59)	1.189 (1.188-1.191)	4.53 (4.49-4.57)	1.319 (1.316-1.322)	12.03 (11.97-12.09)	1.247 (1.245-1.248)	9.32 (9.26-9.37)	1.362 (1.360-1.364)
2007	11.62 (11.56-11.67)	1.324 (1.322-1.325)	4.72 (4.68-4.76)	1.336 (1.333-1.339)	17.24 (17.18-17.31)	1.341 (1.339-1.342)	9.61 (9.56-9.67)	1.377 (1.375-1.379)
2008	11.76 (11.71-11.82)	1.320 (1.319-1.322)	5.27 (5.23-5.31)	1.357 (1.354-1.360)	20.47 (20.40-20.53)	1.383 (1.382-1.384)	11.83 (11.78-11.89)	1.442 (1.440-1.444)
2009	12.56 (12.50-12.62)	1.336 (1.334-1.337)	4.82 (4.78-4.86)	1.330 (1.327-1.333)	20.60 (20.53-20.67)	1.374 (1.373-1.376)	13.11 (13.05-13.16)	1.482 (1.479-1.484)
2010	11.32 (11.26-11.37)	1.315 (1.314-1.317)	2.98 (2.94-3.02)	1.216 (1.213-1.219)	22.20 (22.13-22.27)	1.406 (1.404-1.407)	12.56 (12.50-12.62)	1.456 (1.454-1.458)
2011	9.17 (9.11-9.22)	1.244 (1.243-1.246)	3.34 (3.30-3.39)	1.227 (1.224-1.230)	20.62 (20.55-20.69)	1.366 (1.365-1.367)	13.33 (13.27-13.39)	1.469 (1.467-1.471)
2012	13.44 (13.39-13.50)	1.356 (1.355-1.358)	4.39 (4.35-4.43)	1.305 (1.302-1.308)	26.53 (26.46-26.60)	1.460 (1.459-1.462)	15.27 (15.21-15.33)	1.533 (1.531-1.535)
2013	14.33 (14.28-14.39)	1.380 (1.379-1.382)	4.03 (3.99-4.08)	1.285 (1.282-1.288)	29.36 (29.29-29.43)	1.503 (1.502-1.504)	14.86 (14.80-14.91)	1.532 (1.530-1.534)
2014	13.96 (13.90-14.01)	1.390 (1.388-1.391)	2.01 (1.97-2.06)	1.139 (1.137-1.142)	31.26 (31.19-31.33)	1.556 (1.555-1.557)	15.62 (15.56-15.68)	1.556 (1.554-1.559)
2015	14.52 (14.47-14.57)	1.413 (1.411-1.414)	4.17 (4.13-4.21)	1.277 (1.274-1.280)	31.92 (31.85-31.99)	1.581 (1.580-1.582)	16.41 (16.35-16.47)	1.588 (1.586-1.590)
2016	12.11 (12.06-12.16)	1.372 (1.370-1.373)	2.90 (2.85-2.94)	1.201 (1.198-1.204)	29.70 (29.63-29.76)	1.579 (1.578-1.580)	14.20 (14.14-14.25)	1.536 (1.534-1.538)
2017	11.99 (11.94-12.04)	1.392 (1.391-1.394)	4.26 (4.22-4.31)	1.279 (1.276-1.281)	22.87 (22.81-22.93)	1.520 (1.518-1.521)	11.27 (11.22-11.32)	1.473 (1.471-1.475)
2018	12.12 (12.07-12.17)	1.405 (1.404-1.407)	2.65 (2.61-2.70)	1.163 (1.160-1.165)	22.57 (22.51-22.63)	1.538 (1.537-1.540)	8.39 (8.34-8.44)	1.352 (1.350-1.354)
2019	11.50 (11.45-11.55)	1.388 (1.386-1.390)	3.70 (3.66-3.75)	1.206 (1.203-1.208)	19.22 (19.16-19.28)	1.483 (1.481-1.484)	8.17 (8.12-8.23)	1.320 (1.318-1.322)
