# Supplementary material for: Persistent inequalities in consultation incidence and prevalence of low back pain and osteoarthritis in England between 2004 and 2019
Source: Rheumatol Adv Pract. 2022 Dec 2;7(1):rkac106. doi: 10.1093/rap/rkac106 (PMC9800855; doi:10.1093/rap/rkac106)
Supplement: rkac106_Supplementary_Data [file rkac106_supplementary_data.zip › 22-113 Supplementary Data S1. Technical note.pdf]

Supplemental Technical Note. Technical notes for slope index of inequality and relative index of inequality

The slope index of inequality (SII) and relative index of inequality (RII) are population-weighted and regression-based inequality measurements, which are interpreted as the effect on the health of moving from the least to the most deprived group.

Regress the mortality on the midpoint of IMD categories, weighted by proportion in the population:

$$Prevalence = \beta_0 + \beta_1(IMD\ midpoint) + \varepsilon$$

– Slope Index of Inequality (SII) = β_1
– Relative Index of Inequality (RII) = 1 + (SII/ average of prevalence in the whole population IMD decile 1-5

Where:

β_0 is the intercept of the regression line and the Y-axis
β_1 is the coefficient that relates to the midpoint of the range of the distribution of IMD;
ε is an error term.

SII has the value zero when there is no inequality. Greater values indicate higher levels of inequality. Positive values indicate a higher concentration of a condition among the most deprived group and negative values indicate a higher concentration among the least deprived. RII has the value one when there is no inequality. Values larger than one indicate a higher concentration of a condition among the most deprived group and values smaller than one indicate a higher concentration among the least deprived. SIIs and RIIs were calculated using a standard analytical tool provided by England Office for Health Improvement and Disparities.
